# Recent advances in the discovery and development of drugs targeting the kallikrein-kinin system

**DOI:** 10.1186/s12967-024-05216-5

**Published:** 2024-04-26

**Authors:** Petra Wisniewski, Tanja Gangnus, Bjoern B. Burckhardt

**Affiliations:** Individualized Pharmacotherapy, Institute of Pharmaceutical and Medicinal Chemistry, University of Münster, Corrensstr. 48, 48149 Münster, Germany

**Keywords:** Kallikrein-kinin system, Tissue kallikrein, Plasma kallikrein, Bradykinin, B1 receptor, B2 receptor, Coagulation factor XII, Thromboprophylaxis, Diabetic retinopathy, Diabetic macular edema

## Abstract

**Background:**

The kallikrein-kinin system is a key regulatory cascade involved in blood pressure maintenance, hemostasis, inflammation and renal function. Currently, approved drugs remain limited to the rare disease hereditary angioedema. However, growing interest in this system is indicated by an increasing number of promising drug candidates for further indications.

**Methods:**

To provide an overview of current drug development, a two-stage literature search was conducted between March and December 2023 to identify drug candidates with targets in the kallikrein-kinin system. First, drug candidates were identified using PubMed and Clinicaltrials.gov. Second, the latest publications/results for these compounds were searched in PubMed, Clinicaltrials.gov and Google Scholar. The findings were categorized by target, stage of development, and intended indication.

**Results:**

The search identified 68 drugs, of which 10 are approved, 25 are in clinical development, and 33 in preclinical development. The three most studied indications included diabetic retinopathy, thromboprophylaxis and hereditary angioedema. The latter is still an indication for most of the drug candidates close to regulatory approval (3 out of 4). For the emerging indications, promising new drug candidates in clinical development are ixodes ricinus-contact phase inhibitor for thromboprophylaxis and RZ402 and THR-149 for the treatment of diabetic macular edema (all phase 2).

**Conclusion:**

The therapeutic impact of targeting the kallikrein-kinin system is no longer limited to the treatment of hereditary angioedema. Ongoing research on other diseases demonstrates the potential of therapeutic interventions targeting the kallikrein-kinin system and will provide further treatment options for patients in the future.

**Supplementary Information:**

The online version contains supplementary material available at 10.1186/s12967-024-05216-5.

## Introduction

**Scheme 1:**
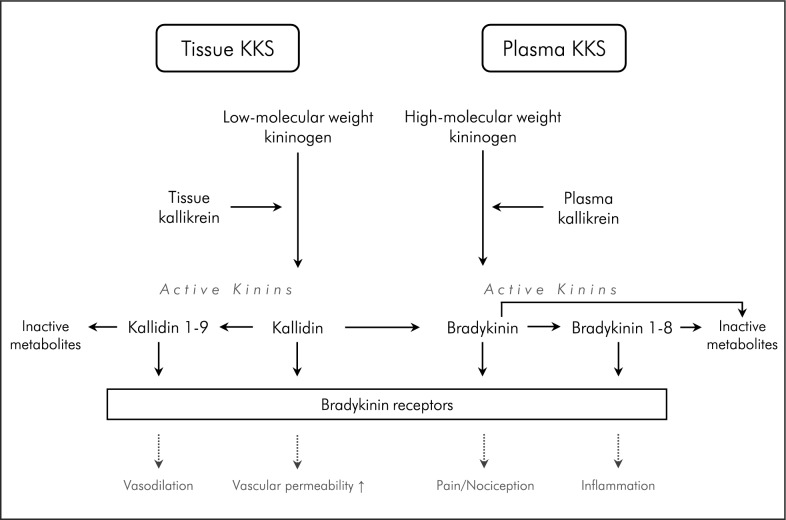
Overview of the kallikrein-kinin system (KKS) in plasma and tissue

The kallikrein-kinin system (KKS) is an endogenous cascade well known for its direct and indirect effects on hemostasis, renal function, blood pressure regulation and on the innate immune system [1–7]. Within the ubiquitously distributed KKS, active kinins are cleaved by the activity of plasma kallikrein (PKa) and tissue kallikrein (TK) from kininogen (Scheme 1) [8]. The active kinin bradykinin (BK) is the most prominent peptide hormone of the KKS; however, there are multiple other active kinins that act on bradykinin receptors [9–11]. Their effects are mediated by the constitutively expressed bradykinin B2 receptor (B2R) and/or the bradykinin B1 receptor (B1R), which is inducible during immunopathology [1, 12]. Its housekeeping functions endow the KKS with dual roles in physiological/pathophysiological conditions [4]. The cascade is subclassified into a plasma- and a tissue-arm which act mostly independently (Scheme 1) [13]. Its complex nature is further attributed to its tight interaction with the intrinsic coagulation cascade via coagulation factor XII (FXII) [14], the renin–angiotensin–aldosterone system and the complement system [2, 3].

Despite its important endogenous role and therefore promising drug target, the predominant therapeutic focus is limited to hereditary angioedema (HAE), a rare disease affecting 1 in 50,000 to 100,000 people [15]. However, recent research has demonstrated the relevance of the KKS in numerous other major diseases, such as cancer, sepsis, cardiovascular disease and pain [6, 16–19]. The KKS is a potential promising target for developing new therapies for diseases in which optimal therapeutic strategies have not yet been established due to factors such as resistance or escape mechanisms [20, 21]. The growing interest in this key regulatory system is indicated by an increasing number of drug candidates being used in preclinical and clinical development.

Therefore, this systematic review aims to provide a comprehensive overview of advances in the discovery and development of drugs targeting the KKS. Besides the approved drugs, it will focus on promising new drug candidates, their mode of action, new indications and their potential for approval in near future.

## Methods/Data collection

For the present review, a two-stage literature search for drugs targeting the KKS was conducted between March and December 2023. The first step was to identify drug candidates targeting the KKS. This was accomplished by searching clinicaltrials.gov for “kallikrein”, “kinin”, “prekallikrein”, “prolylcarboxypeptidase”, “FXII”, “bradykinin receptor” “B1 receptor”, “B2 receptor” and “C1 inhibitor” in “other terms” to identify compounds currently in clinical development. To additionally cover drug candidates in preclinical development, the following MeSH terms were searched in Pubmed: ((((((((prolylcarboxypeptidase[Title/Abstract]) OR (FXII[Title/Abstract])) OR (prekallikein[Title/Abstract])) OR (kallikrein[Title/Abstract])) OR (bradykinin receptor[Title/Abstract])) OR (b1 receptor[Title/Abstract])) OR (b2 receptor[Title/Abstract])) OR (c1 inhibitor[Title/Abstract])) AND (preclin*[Title/Abstract]). In addition, the websites of the U.S. Hereditary Angioedema Association and pharmaceutical companies developing drugs with a target in the KKS were searched. Identified publications and trials were screened for drugs/drug candidates and checked to ensure that they met the inclusion criteria (clinical or preclinical development in the last 25 years or already approved for use in humans). The second step was to search Pubmed and Clinicaltrials.gov for the most recent publications of clinical and preclinical trials for the identified compounds. For compounds for which no publications were available in these two databases, a search in Google Scholar was additionally performed.

This review does not include the well-established angiotensin-converting enzyme and neprilysin inhibitors. Their connection to the KKS and the therapeutic value in hypertension and heart failure have been well summarized in several recent reviews [22, 23]. Within the group of promising new DNA/RNA aptamers, there are drug candidates under development that target PK, FXII, and other coagulation factors. Their state of development and their therapeutic use for the regulation of hemostasis were discussed also in a recent review [24].

## Results and discussion

### Search results

**Fig. 1:**
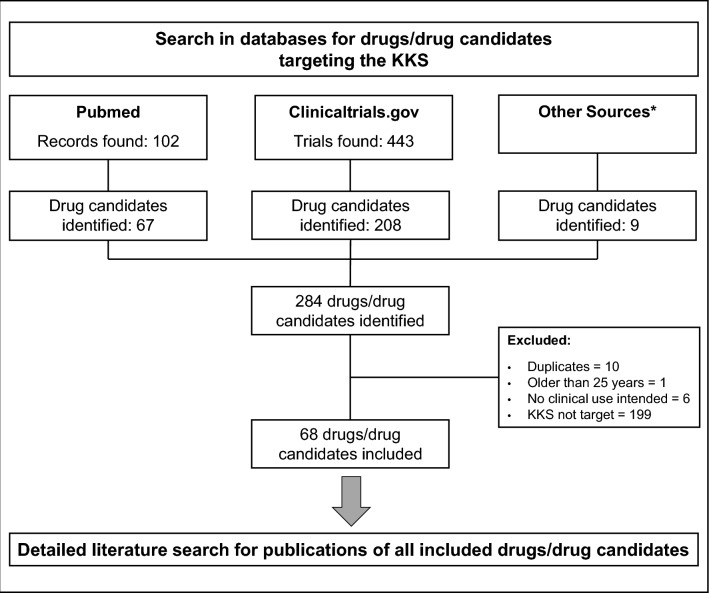
Flow diagram of the conducted literature search. *Other sources are the websites of the US Hereditary Angioedema Association and of pharmaceutical companies developing drugs with a target in the kallikrein-kinin system (KKS)

The literature search yielded 102 articles and 443 trials, all of which were included in the final analysis (Fig. 1). Those articles and trials included a total of 68 different drugs, 10 of which are approved, 25 of which are in clinical development, and 33 of which are in preclinical development (see Tables 1, 2, 3; some drugs are in several stages of development regarding different indications). The three most studied indications are hereditary angioedema (HAE), diabetic retinopathy including diabetic macular edema (DME), and thromboprophylaxis. The identified compounds are sorted by target, stage of development and intended indication.

**Table 1:** Approved drugs with targets in the kallikrein-kinin system

Drug	Class	Approved indication	Ref
**C1 esterase inhibitor**			
Human C1 esterase inhibitor	Protein replacement therapy	Hereditary angioedema (on-demand and prophylaxis)	[110–112]
Conestat alfa	Protein replacement therapy	Hereditary angioedema (on-demand)	[117]
**Coagulation factor XII**			
Garadacimab/CSL312	Monoclonal antibody (fully human)	Hereditary angioedema (prophylaxis)	[27]
**Plasma (P) and tissue (T) kallikrein**			
Berotralstat (P)	Small molecule inhibitor	Hereditary angioedema (prophylaxis)	[57, 58]
Ecallantide (P)	Inhibitor, protein	Hereditary angioedema (on-demand)	[53, 54]
Lanadelumab (P)	Monoclonal antibody (fully human)	Hereditary angioedema (prophylaxis)	[55, 56]
Aprotinin (P, T)	Inhibitor, protein	Reduction of perioperative blood loss	[87–92]
Ulinastatin (P, T)	Inhibitor, protein	Sepsis, pancreatitis	[95, 96]
Human urinary kallidinogenase (T)	Enzyme replacement therapy	Acute ischemic stroke	[101]
**Bradykinin B2 receptor**			
Icatibant	Peptide antagonist	Hereditary angioedema (on-demand)	[121, 122]

(P) and (T) indicate whether plasma (P) or tissue (T) kallikrein is the drug target

**Table 2:** Drug candidates in clinical development with targets in the kallikrein-kinin system

Drug candidate	Class	Intended indication	Current trial phase	Year of latest publication	Refs.
**C1 esterase inhibitor**					
BMN 331	AAV5-based gene transfer	Hereditary angioedema (prophylaxis)	1/2	Study ongoing	[120]
Human C1 esterase inhibitor	Protein replacement therapy	ACEi-induced angioedema	3	2022	[116]
		Acute antibody-mediated rejection after kidney transplant	3	2020	[115]
		Delayed graft function after kidney transplant*	1/2	2020	[114]
		Neuromyelitis optica	1	2014	[162]
Conestat alfa	Protein replacement therapy	Stroke prevention after transcatheter aortic valve transplantation	2	Study ongoing	[119]
		COVID-19*	2	2021	
		Amoreliate adverse events of intravenous immunoglobulin therapy	4	2021	[118]
**Coagulation factor XII**					
Garadacimab/CSL312	Monoclonal antibody (fully human)	COVID-19	2	2023	[28]
		Idiopathic pulmonary fibrosis	2	(2024)	[29]
		Catheter-associated blood clot formation in subjects with cancer who receive chemotherapy through a PICC line*	1/2	(2020)	[30]
Ixodes ricinus-contact phase inhibitor (Ir-CPI)	Inhibitor, protein	Thromboprophylaxis during cardiopulmonary bypass surgery	1	(2023)	[31–33]
**Plasma (P) and tissue (T) kallikrein**					
ADX-324 (P)	siRNA	Hereditary angioedema (prophylaxis)	1	(2023)	[70]
ATN-249 (P)	Small molecule inhibitor	Hereditary angioedema (prophylaxis)	1	2017	[63]
Avoralstat/BCX4161 (P)	Small molecule inhibitor	Hereditary angioedema (prophylaxis)	3	2018	[59]
Donidalorsen/IONIS-PKK-L_RX_ (P)	Antisense oligonucleotide	Hereditary angioedema (prophylaxis)	3	2022	[66, 68, 69]
		COVID-19	2	(2020)	[163]
IONIS-PKK_RX_ (P)	Antisense oligonucleotide	Hereditary angioedema (prophylaxis)	2	2020	[66]
		Chronic migraine	2	2020	[67]
KVD001 (P)	Small molecule inhibitor	Diabetic macular edema	2	2019	[72]
KVD824 (P)	Small molecule inhibitor	Hereditary angioedema (prophylaxis)*	2	(2022)	[62]
Lanadelumab/DX-2930 (P)	Monoclonal antibody (fully human)	Dialysis-induced hypotension	2	Study ongoing	[76]
		FXII-associated cold autoinflammatory syndrome	2	Study ongoing	[78]
		Lung injury	1	Study ongoing	[77]
NTLA-2002 (P)	CRISPR/CAS 9 gene editing	Hereditary angioedema (prophylaxis)	1/2	Study ongoing	[64, 65]
RZ402 (P)	Small molecule inhibitor	Diabetic macular edema	2	Study ongoing	[75]
Sebetralstat/KVD900 (P)	Small molecule inhibitor	Hereditary angioedema (on-demand)	3	2023	[60, 61]
STAR-0215 (P)	Monoclonal antibody (humanized)	Hereditary angioedema (prophylaxis)	2	Study ongoing	-
THR-149 (P)	Peptide inhibitor	Diabetic macular edema	2	Study ongoing	[73]
MDCO-2010 (P, T)	Small molecule inhibitor	Reduction of blood loss during coronary artery bypass graft surgery*	2	2014	[97, 98]
Aprotinin (P, T)	Inhibitor, protein	COVID-19	3	2022	[93, 94]
DM199 (recombinant tissue kallikrein) (T)	Enzyme replacement therapy	Acute ischemic stroke	2/3	(2022)	[103, 104]
		Kidney disease	2	(2022)	[106]
		Diabetes mellitus type 2	2	(2014)	[105]
Porcine pancreatic pig tissue kallikrein (T)	Enzyme replacement therapy	Restenosis after stenting of symptomatic middle cerebral artery (MCA) atherosclerotic stenosis	2	2016	[102]
**Bradykinin B2 receptor**					
Anatibant/LF 16-0687	Small molecule antagonist	Traumatic brain injury*	2	2009	[129]
Deltibant/CP-1027/Bradycor	Peptide antagonist	Traumatic brain injury	2	1999	[128]
Deucrictibant/PHA-022121	Small molecule antagonist	Hereditary angioedema (on-demand)	2	2022	[127]
Fasitibant/MEN16132	Small molecule antagonist	Knee osteoarthritis*	2	2015	[130]
Icatibant/HOE 140	Peptide antagonist	Dialysis-induced hypotension	2	2023	[125, 126]
		ACEi-induced upper airway angioedema	3	2017	[123, 124]
Labradimil/RMP-7	Peptide agonist	Primary brain tumors*	2	2006	[134, 135]
**Bradykinin B1 receptor**					
BAY 2395840	Antagonist	Diabetic neuropathic pain*	2	(2023)	[146, 147]
BI 1026706	Small molecule antagonist	Diabetic macular edema	2	2020	[144]
Safotibant/LF22-0542	Small molecule antagonist	Diabetic macular edema*	2	2012	[145]

Year of latest publication: Years are those of the most recent publications found in peer-reviewed journals

Years in parentheses indicate press releases, poster presentations at conferences or announcements on clinicaltrials.gov

Study ongoing means that the study is currently ongoing and there are no publications from previous studies

^*^Indicates that development of the drug candidate for this indication was discontinued during clinical trials

(P) and (T) indicate whether plasma (P) or tissue (T) kallikrein is the target of the active pharmaceutical ingredient

AAV = adeno-associated virus; ACEi = angiotensin-converting enzyme inhibitor; COVID-19 = coronavirus disease 2019; CRISPR/Cas9 = clustered regularly interspaced short palindromic repeats/CRISPR associated protein 9; FXII = coagulation factor XII; PICC = Peripherally inserted central catheter; siRNA = small interfering ribonucleic acid

**Table 3:** Identified drug candidates in preclinical development with targets in the kallikrein-kinin system

Drug candidate	Class	Intended indication	Year of latest publication	Refs.
**Prolylcarboxypeptidase**				
UM8190	Small molecule inhibitor	Thromboprophylaxis, obesity	2012	[51]
**Coagulation factor XII**				
3F7	Recombinant antibody (fully human)	Thromboprophylaxis	2022	[26]
ALN-F12	siRNA	Hereditary angioedema (prophylaxis)	2019	[38]
		Thromboprophylaxis	2020	[37]
ARC-F12	siRNA	Hereditary angioedema (prophylaxis)	(2016)	[39]
		Thromboprophylaxis	(2016)	[39]
COU254	Small molecule inhibitor	Acute ischemic stroke	2010	[42]
FXII900	Peptide inhibitor	Thromboprophylaxis	2021	[34, 35]
KV998086	Small molecule inhibitor	Hereditary angioedema (on-demand)	(2022)	[40, 41]
rHA-Infestin-4	Inhibitor, protein	Acute ischemic stroke	2016	[43–46]
DX-4012	Monoclonal antibody (fully human)	Thromboprophylaxis	(2015)	[36]
Sylvestin	Peptide inhibitor	Acute ischemic stroke	2022	[47]
**Plasma (P) and tissue (T) kallikrein**				
CU-2010 (P)	Small molecule inhibitor	Prevention of blood loss in cardiac surgery	2009	[99]
Ecallantide/DX-88 (P)	Inhibitor, protein	Cerebral ischemia	2006	[85]
KV998052 (P)	Small molecule inhibitor	Acute respiratory distress syndrome	(2021)	[79]
		Retinal edema	(2022)	[80]
KV998054 (P)	Small molecule inhibitor	Retinal edema	(2019)	[80]
VA999272 (P)	Small molecule inhibitor	Retinal edema	2016	[81]
VE-3539 (P)	Small molecule inhibitor	Diabetic retinopathy	(2018)	[83]
VE-4840 (P)	Small molecule inhibitor	Retinal edema	(2019)	[82]
DX-2300 (T)	Monoclonal antibody (fully human)	Airway diseases	2009	[86]
**Bradykinin B2 receptor**				
Bradyzide	Small molecule inhibitor	Inflammatory hyperalgesia	2009	[133]
FR173657	Small molecule inhibitor	Skin inflammatory diseases	2009	[132, 153]
LF 18-1505T	Small molecule inhibitor	Closed head trauma	2006	[131]
FR-190997	Small molecule partial agonist	Ocular hypertension	2014	[139]
NG291	Peptide agonist	Disruption of blood brain barrier	2021	[136, 137]
R523	Peptide agonist	Glioma	2010	[138]
**Bradykinin B1 receptor**				
B 9430	Peptide antagonist	Cerebral ischemia	2000	[156]
B 9858	Peptide antagonist	Inflammatory hyperalgesia	2002	[151]
BI 113823	Small molecule antagonist	Chronic liver fibrosis	2022	[155]
ELN441958	Small molecule antagonist	Inflammatory hyperalgesia	2007	[152]
MK-0686	Small molecule antagonist	Chronic pain	2007	[149]
NVP-SAA164	Small molecule antagonist	Inflammatory hyperalgesia	2005	[150]
R-715	Peptide antagonist	Skin inflammatory diseases	2009	[153]
R-954	Peptide antagonist	Diabetic retinopathy	2018	[148]
SSR240612	Small molecule antagonist	Skin inflammatory diseases	2009	[132, 153]
NG29	Peptide agonist	Glioma	2016	[157, 158]

Year of latest publication: Years are those of the most recent publications found in peer-reviewed journals

Years in parentheses indicate press releases or poster presentations at conferences

“-” means there are no publications or press releases available

(P) and (T) indicate whether plasma; (P) or tissue (T) kallikrein is the target of the active pharmaceutical ingredient; siRNA = small interfering ribonucleic acid

### Coagulation factor XII

**Fig. 2:**
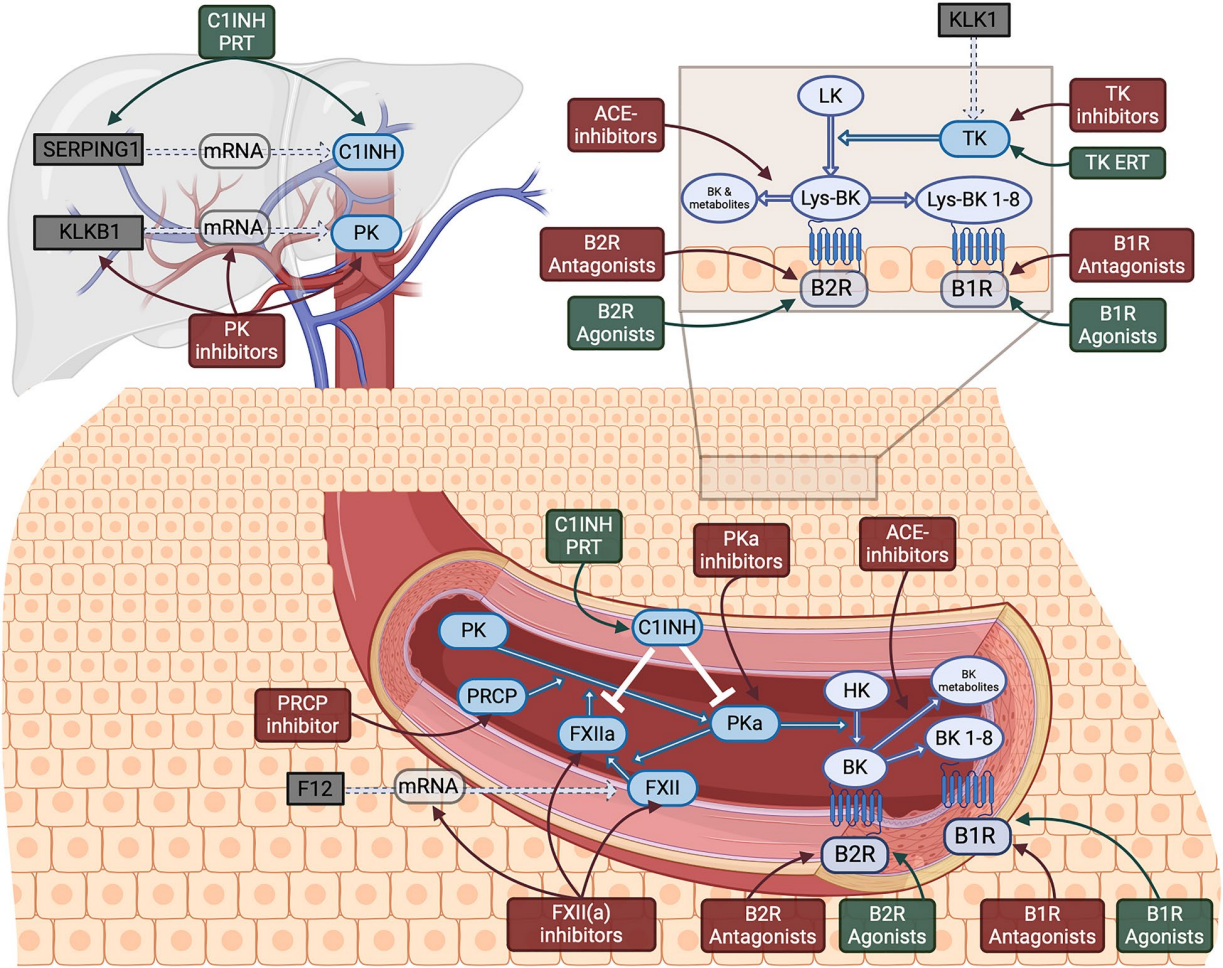
Representation of the human kallikrein-kinin system in plasma and tissue and identified drug targets. ACE: Angiotensin-converting enzyme, B1R: Bradykinin B1 receptor, B2R: Bradykinin B2 receptor, BK: Bradykinin, BK 1–8: Bradykinin 1–8, C1INH: C1 esterase inhibitor, ERT: Enzyme replacement therapy, F12: Coagulation factor XII gene, FXII: Coagulation factor XII, HK: High molecular weight kininogen, KLK1: Tissue kallikrein gene, KLKB1: Prekallikrein gene, LK: Low molecular weight kininogen, Lys-BK: Lysin-Bradykinin/Kallidin, Lys-BK 1–8: Lysin-Bradykinin 1–8/Kallidin 1–9, mRNA: Messenger ribonucleic acid, PK: Plasma prekallikrein, PKa: Plasma kallikrein, PRCP: Prolylcarboxypeptidase, PRT: Protein replacement therapy, SERPING1: C1 esterase inhibitor gene, TK: Tissue kallikrein. Created with BioRender.com

FXII is found at the crosstalk site between the KKS and the intrinsic coagulation cascade (Fig. 2). Hence, FXII/FXIIa inhibition is emerging not only as a therapeutic strategy for patients with HAE (one approved and one FXIIa inhibitor under development) but also for investigation of their ability to affect hemostasis in various diseases (e.g., stroke, thromboprophylaxis) [25]. Additionally, two drugs from the new group of small interfering RNAs (siRNAs) are being developed for both indications.

#### Approved drugs

Garadacimab (CSL312) is an FDA-approved fully human monoclonal IgG4 antibody against FXIIa. It is an affinity-matured, recombinant variant of the monoclonal antibody 3F7 which was shown to reduce the severity of potentially life-threatening abdominal aortic aneurysms, inhibit the development of atherosclerosis, stabilize vulnerable plaques, and reduce systemic and local proinflammatory marker levels in three different mouse models of cardiovascular disease [26]. Monthly subcutaneous administration of garadacimab significantly reduced the incidence of HAE attacks per month (0.27 vs. 2.01; p < 0.0001) in a randomized, double-blind, placebo-controlled phase 3 study in 2022. No association with an increased risk of bleeding or thromboembolic events was observed [27]. In contrast, a phase 2 study in patients with severe coronavirus disease 2019 (COVID-19) did not show any effect [28]. Garadacimab was investigated for the treatment of idiopathic pulmonary fibrosis in a phase 2 study which ended in November 2023 [29]. Results have not yet been published. A phase 1b study for preventing catheter-associated blood clot formation in subjects with cancer who receive chemotherapy through a peripherally inserted central venous catheter has been withdrawn in 2020 due to non-safety related company decisions [30].

#### Drug candidates in clinical development

For the treatment of thrombo-inflammatory diseases and hemorrhagic stroke, Ixodes ricinus-contact phase inhibitor (Ir-CPI), a recombinant protein expressed in the salivary glands of the tick Ixodes Ricinus, is in clinical development, and a phase 2 trial was announced in August 2023 [31]. A phase 1 study confirmed preclinical findings on dose-dependent prolongation of activated partial thromboplastin time [32, 33].

#### Drug candidates in preclinical development

For FXIIa inhibitors that have not yet entered clinical development, the majority of potential indications are thromboprophylaxis, stroke and HAE. Using FXII900 in animal models of thrombosis (mice and rabbits) reduced clot formation by half in treated mice (42.8% treatment vs. 87.5% placebo, p = 0.02) and the resistance measured at the inlet of an artificial lung used in rabbits remained at baseline in 75% of the treated animals [34, 35]. No changes in bleeding tendency or blood loss were detected in either animal species, suggesting that FXII900 is a promising candidate for thromboprophylaxis. DX-4012 is a fully human monoclonal antibody in preclinical development for thromboprophylaxis. The manufacturer presented data on its anti-thrombotic activity in various animal models at a conference in 2015 [36].

Two of the new compounds belong to the new group of siRNAs and are being investigated for use in thromboprotection and HAE treatment. ALN-F12 targets a 23-nucleotide region of FXII mRNA and is still in preclinical development. In a study in mice on the prevention of thrombosis, a decrease in FXII plasma levels (55% at 0.3 mg/kg and 93% at 1 mg/kg) was observed, as was a dose-dependent reduction in fibrin accumulation (up to tenfold at 10 mg/kg) [37]. In animal studies evaluating the potential of treatment of HAE via FXII-inhibition, similar dose-dependent reductions in FXII plasma levels were observed in female C57BL/6 mice 10 days after a single subcutaneous administration (51% at 0.3 mg/kg and 93% at 1 mg/kg) [38]. The levels returned to baseline approximately 64 days after termination of dosing. ARC-F12 is another FXII-targeting siRNA drug being investigated in animal studies for HAE and thromboprophylaxis. A single subcutaneous injection resulted in an even higher reduction in FXII plasma levels (86% at 1 mg/kg and 96% at 3 mg/kg) as with ALN-F12 [39].

Moreover, KV998086, a small molecule FXIIa inhibitor, is in preclinical development as an oral prophylactic treatment for HAE by preventing edema [40]. First clinical trial was planned for 2023 [41]**.**

The remaining three drug candidates are being developed for the prevention and treatment of stroke. The selective nonpeptidic FXIIa inhibitor COU254 did not influence regional cerebral blood flow or reduce ischemic brain damage following transient middle cerebral artery filament occlusion compared to that in untreated mice [42]. rHA-Infestin-4 is a FXIIa inhibiting protein (infestin-4) produced from the blood-sucking bug *Triatoma infestans* coupled to recombinant human albumin [43]. Three animal studies have demonstrated its thrombolytic effect. Therapeutic administration after transient middle cerebral artery occlusion (tMCAO) in rats had no significant effect on the infarct area or edema formation but still improved neurological scores and reduced mortality [7% vs 32% (controls); p < 0.05] [44]. In traumatic brain injury in mice, rHA-infestin-4 was able to decrease the volume of brain lesions by 50% when administered one hour after injury, which was comparable to the effects observed in FXII deficient mice [45]. Another study showed dose-dependent protection against thrombosis in an arteriovenous shunt model in rats and rabbits (clot weight reduction up to 88%, p < 0.001) and a less than twofold increase in bleeding time in a cuticle bleeding model [46]. Nonetheless, high doses cause off-target effects, and as a derivative of an insect-derived protein -infestin-4- potential immunogenicity in humans cannot be excluded [46]. Sylvestin is an inhibitor of FXIIa and PKa derived from forest leeches (*Haemadipsa sylvestris*) [47]. Dose-dependent decreases in infarct volume compared to those in controls were shown after one hour of middle cerebral artery occlusion (MCAO), and the inhibition of thrombus formation was demonstrated in mice [47]. In three different bleeding models, sylvestin did not increase bleeding, but it had a modest inhibitory effect on FXa [47].

The current focus of research on FXIIa inhibitors pertains predominantly on influencing hemostasis in various diseases, followed by HAE. All currently approved anticoagulants, regardless of their mode of action, increase the risk of bleeding [48]. As a result, safe therapy is not yet available for many multimorbid patients putting them at risk for lethal or disabling thromboembolic events. The high demand for safe anticoagulants is reflected in the large number of FXIIa inhibitors under development. If the promising thromboprophylaxis that has been shown in animal studies can be translated to humans without increasing the risk of bleeding, it could be a safe therapeutic option for a large number of patients.

### Prolylcarboxypeptidase (PRCP)

PRCP is a carboxypeptidase that affects the KKS by activating PK and is involved in the regulation of the cardiovascular system by degrading BK 1–8, angiotensin II and angiotensin III [49, 50].

#### Drug candidates in preclinical development

UM8190 blocks PRCP-dependent PK activation and thus also blocks PKa-dependent FXII activation, reversing the escalating feedback mechanism [51]. It was found that UM8190 prevented vessel occlusion in the ferric chloride-induced mouse carotid artery thrombosis model in FXII-deficient mice which could not be confirmed in wild-type mice. However, the prothrombotic effect of PRCP does not appear to be dependent on PKa, as it could not be demonstrated in wild-type mice. This effect is likely mediated by PRCP-dependent increased generation of reactive oxygen species, as treatment of PRCP-deficient mice with antioxidants abolishes their prothrombotic phenotype [52]. Moreover, after oral administration in mice, a time- and dose-dependent anorectic effect was observed, indicating a potential application in treating obesity [51].

PRCP is the least studied target associated with the KKS, and additional research is needed to better assess its therapeutic potential.

### Plasma and tissue kallikrein

Drug candidates targeting plasma and tissue kallikrein are the focus of current research within the KKS. Emerging applications include chronic ocular diseases and influencing hemostasis during certain surgical procedures. The approved drugs and drug candidates are either selective inhibitors of plasma and/or tissue kallikrein or broad-spectrum protease inhibitors that inhibit a number of serine proteases including PKa. Additionally, activation of the KKS by TK agonists is used to favorably influence ischemic diseases.

#### Selective inhibitors of plasma kallikrein

Most selective PKa inhibitors are intended for use in the treatment of HAE, with three being approved for the prevention or treatment of acute HAE attacks. The direct inhibition of PKa is intended to compensate for the lack of inhibition by C1INH.

*Approved drugs *Ecallantide is a recombinant PKa inhibitor approved by the FDA for the on-demand treatment of HAE attacks. In a double-blind, placebo-controlled phase 3 study in patients with moderate or severe HAE attacks, subcutaneous administration of ecallantide significantly improved symptoms 4 h after administration, as measured by the mean symptom complex severity (MSCS) score, compared to that of placebo [53]. An initial improvement in symptoms was seen at 2 h, and the effect was sustained over the 24-h observation period [53]. A subsequent open-label follow-up study showed comparable results (change in MSCS score: − 1.04 ± 0.77 open-label study vs. − 0.8 ± 0.6 phase 3 study); however, anaphylactic reactions occurred in 4.1% of treated subjects [54]. The human monoclonal antibody against PKa, lanadelumab, is investigated in a variety of diseases in addition to its approved use in the prevention of HAE. A randomized, double-blind, placebo-controlled phase 3 trial showed a significant improvement in quality of life in patients with HAE as measured by the Angioedema Quality of Life Questionnaire (AE-QoL) [55]. A subsequent open-label extension study demonstrated a reduction from 3.1 attacks to 0.4 attacks per month one month after treatment initiation [56]. Berotralstat is the first oral PKa inhibitor approved for the prevention of HAE attacks [57]. In a phase 3 study a significant reduction in attacks per month was demonstrated, but less than that with subcutaneous C1INH prophylaxis (attacks per month: placebo: 2.35; 150 mg berotralstat per day: 1.31; 60 IU/kg C1INH twice weekly: 0.017–0.09 attacks) [58].

*Drug candidates in clinical development *Five additional PKa inhibitors for the treatment or prophylaxis of HAE attacks are in clinical development. Avoralstat, an oral HAE prophylaxis, is the most advanced in development, but failed to show a reduction in HAE attacks in a phase 3 trial [59]. Sebetralstat, an oral on-demand treatment, significantly increased the time to conventional treatment after study drug administration in a phase 2 study [60]. A phase 3 trial is currently underway [61]. The phase 2 trial of KVD824 was terminated early due to safety concerns after elevated liver enzymes were observed in all treatment groups [62]. For ATN-249, promising results from a phase 1 trial were announced in 2019, and currently enrollment in a phase 2 trial for once-daily oral prophylaxis of HAE has been started [63]. STAR-0215 is subcutaneously administered once every three months. The phase 1b/2 trial in HAE patients started in early 2023.

Additional approaches for influencing PKa activity include intervening at the DNA or RNA level. NTLA-2002 is a CRISPR/CAS 9-based gene editing therapy used to reduce PKa activity by removing the gene encoding PK (KLKB1) from hepatocytes. A phase 1/2 trial investigating the use of NTLA-2002 in HAE patients is still ongoing; however, the first positive interim results regarding efficacy, tolerability and safety have been indicated [64, 65]. With IONIS-PKK_RX_ and donidalorsen (formerly IONIS-PKK-L_RX_) two antisense oligonucleotides are being investigated [66, 67]. These compounds inhibit PK production in hepatocytes through ribonuclease H1-mediated degradation of PK mRNA, thus reducing PKa activity. According to the company's website, only donidalorsen will be further developed due to the higher target specificity of the ligand, resulting in better efficacy and tolerability [68]. Donidalorsen is a ligand conjugated form of IONIS-PKK_RX_ [69]. The conjugated N-acetylgalactosamine-moiety increases receptor-mediated uptake into liver hepatocytes, allowing targeted drug delivery and increasing potency by a factor of 30, which allows for less frequent and lower-dose administration. The results from the phase 2 study showed a 90% reduction in HAE attacks per month compared to placebo, with very good tolerability [69]. A phase 3 study is currently ongoing. Finally, a further treatment approach for HAE bases on siRNAs; yet the sole drug candidate is ADX-324 [70]. Enrollment in a phase 1 study started in early 2023. No preclinical results have been formally reported.

Diabetic macular edema (DME) is another area where PKa inhibitors are being extensively researched. Studies in DME patients have shown that PKa levels in the vitreous are more elevated than vascular endothelial growth factor (VEGF) levels are, and this difference is thought to contribute to the incomplete or delayed response to anti-VEGF therapy in many patients [71]. KVD001 and THR-149 are intravitreally administered PKa inhibitors. KVD001 failed to meet the primary endpoint of the conducted phase 2 study, as indicated by the lack of significant improvement in best corrected visual acuity (BCVA) [72]. A randomized, multicenter phase 2 study with THR-149 is still ongoing after the phase 1 study showed no dose-limiting toxicity and an improvement in BCVA [73]. Additionally, an orally administered PKa inhibitor against DME, RZ402 (formerly ASP440 and ASP-440 [74]), is under development. A phase 2 trial with RZ402 in participants with DME who are naive to or have received limited anti-VEGF injections, was initiated in 2023. However earlier results from the phase 1 trial (RZ402-101) have not yet been published [75].

Clinical trials are also exploring the use of lanadelumab for its potential use in dialysis-induced hypotension (DIH) (phase 2) [76] and lung injury (phase 1) [77]. BK-mediated vasodilation and increased vascular permeability are hypothesized to play important roles in the pathomechanisms of both conditions. The objective of a third study is to investigate whether inhibiting PKa has a positive effect on the rare disease FXII-associated cold autoinflammatory disease (FACAS) in a small cohort [78]. The results of studies investigating the use of lanadelumab for DIH, lung injury and FACAS have not yet been published.

*Drug candidates in preclinical development *Moreover, PKa inhibitors are under preclinical investigation as potential treatments for acute respiratory distress syndrome (ARDS), retinopathies, and brain ischemia. KV998052 was shown to be associated with improved blood oxygenation in an acid-aspiration lung injury model in mice [79]. The efficacy of the treatment of ARDS is achieved through the inhibition of PKa, which ultimately reduces the release of BK and its pro-inflammatory and edema-inducing effects. Additionally, it was studied in mouse models of VEGF-induced retinal edema, alongside three other PKa inhibitors (KV998054, VA999272, and VE-4840). KV998052 and KV998054 were both tested in a mouse model of retinal edema and reduced retinal thickening after VEGF injection by 37% (p = 0.0018) and 59% (p = 0.008), respectively [80]. PKa inhibition by VA999272 also reduced retinal thickening (57%, p < 0.001) induced by VEGF application [81]. The effect was comparable to that in KLKB1-deficient mice and was also demonstrated in rats (53%, p < 0.001). Among the PKa inhibitors tested, VE-4840 had the least effect on VEGF-induced retinal thickening (30.8%, p = 0.0055) [82]. Moreover, VE-3539, an orally administered PKa inhibitor, was tested in a human PKa injection model in diabetic rats and was shown to reduce vascular leakage and improve intravascular blood flow [83].

A 2006 study investigated the use of the PKa inhibitor ecallantide in the treatment of cerebral ischemia, since BK has been shown to increase brain edema after ischemic stroke [84]. Dose-dependent reductions in ischemic volume (up to 61%) and brain swelling (up to 68%) were shown in a placebo-controlled model of transient and permanent focal brain ischemia in mice. An effect was observed only if the drug was administered before ischemia or before reperfusion [85].

#### Selective inhibitors of tissue kallikrein

*Drug candidates in preclinical development *DX-2300 is a fully human monoclonal antibody that inhibits TK. Although no immediate effects were observed in a sheep model of asthma, a 91% reduction in late-phase kininogen-induced bronchoconstriction was observed following inhalation administration of DX-2300 [86]. This late-phase airway response is triggered by inflammatory processes associated with increased TK activity; and therefore, TK inhibitors such as DX-2300 could be a therapeutic agent for patients suffering from allergic asthma [86]. However, as no further data have been published since this study was conducted in 2009, this approach does not appear to be promising.

#### Broad-spectrum protease inhibitors

In addition to the selective kallikrein inhibitors, there are four broad-spectrum protease inhibitors that inhibit a number of serine proteases, including PKa. Since PKa activates FXII, its inhibition is also a potential target for influencing hemostasis in various diseases.

*Approved drugs *Aprotinin was approved by the FDA and EMA to reduce blood loss during bypass surgery. After being temporarily withdrawn from the market by the manufacturer in 2008, due to serious adverse events, it is now being reapproved for use in high-risk patients [87–92]. All the studies demonstrated comparable or greater reductions in blood loss with aprotinin than with tranexamic acid. In patients with moderate or severe COVID-19 controversial results were observed in phase 3 studies [93, 94]. Ulinastatin is approved in some Asian countries for the treatment of sepsis and acute pancreatitis. In sepsis, ulinastatin has been shown to significantly reduce inflammatory signs (body temperature, white blood cell count, inflammatory cytokines) and mortality [95]. In pancreatitis, ulinastatin showed a significant decrease in mortality only in patients with severe pancreatitis [96].

*Drug candidates in clinical development *MDCO-2010 is an active site inhibitor of PKa as well as of plasmin, of coagulation factors Xa, XIa and of activated protein C [97]. It has been investigated as an alternative to aprotinin for the prevention of blood loss in coronary artery bypass grafting during cardiopulmonary bypass (CPB) surgery. Two phase 2 studies were conducted, with one of which showed no significant benefit, while the other was terminated due to unspecified safety concerns [97, 98].

*Drug candidates in preclinical development *CU-2010 is a synthetic small molecule serine protease inhibitor, that was also developed as a substitute for aprotinin. Currently, there is only evidence of decreased blood loss during CPB surgery in preclinical canine studies [99].

Overall, plasma and tissue kallikrein are promising targets with most candidates in preclinical and clinical development. DME therapy could benefit greatly from potential new drug candidates. Furthermore, the search for a substitute for aprotinin highlights the necessity for safe therapies to prevent blood loss during surgery.

#### Enzyme replacement of tissue kallikrein

##### Approved drugs

Human urinary kallidinogenase (HUK) is a glycoprotein found in human urine that belongs to the TK family. It can cleave kallidin from LK, making it an agonist of the KKS. Kallidin may reduce the incidence of stroke by promoting local vasodilation and long-term vascularization. HUK has been approved for use in China as a treatment for acute ischemic stroke. While phase 3 data are available only upon request from trial investigators, the data of a phase 4 trial have been published [100]. The multi-center study in 1202 patients with acute ischemic stroke evaluated safety and efficacy [101]. However, in the absence of a control group, it is not possible to assess superiority over the standard of care.

##### Drug candidates in clinical development

Two additional TK agonists are presently undergoing clinical development. The first is a porcine pancreas-derived TK that yielded a 71% reduction in in-stent restenosis after stenting of symptomatic atherosclerotic middle cerebral artery stenosis in a phase 2 trial by daily peroral administration [102]. The second drug candidate, DM199, is a recombinant TK and has been developed for use in acute ischemic infarction [103]. In 2022, the FDA halted a phase 2/3 trial due to safety concerns [103]. After the hold was lifted in June 2023, the manufacturer announced the continuation of the study [104]. Phase 2 studies with DM199 have been conducted in patients with type 2 diabetes mellitus in 2014 as well as kidney disease [105, 106]. Nevertheless, no outcomes have been published thus far.

The use of TK and its analogs indicated a protective effect of KKS activation in ischemic disease. To determine the clinical relevance of these findings, further studies are needed.

#### C1 esterase inhibitor

The KKS is tightly controlled by C1INH, which irreversibly inhibits PKa and FXIIa to limit the production of active kinins [107]. This protease inhibitor is synthesized mainly in the liver and monocytes [108, 109]. Targeting C1INH is approved for the treatment and prophylaxis of acute attacks of HAE by blood-derived or recombinant replacement therapy and under clinical investigation for gene therapy. A number of other indications are currently being explored in clinical trials.

##### Approved drugs

Blood-derived C1INH for intravenous application is approved for on-demand treatment and prophylaxis of acute HAE attacks [110]. In a long-term study in which C1INH was intravenously administered twice a week as prophylaxis, one-third of the patients were attack-free during the treatment period (mean duration of 9.2 months per subject) [111]. The remaining two thirds experienced an average of 0.57 attacks per month. An approved subcutaneously administered formulation even improved this finding by showing even lower attack rates (0.09 attacks per month) and a higher percentage of attack-free participants (44%) [112]. Among patients treated for more than 12 months, the attack rate was 0.017 per month, and the proportion of attack-free patients was 50%. These low attack rates, combined with a comparably low adverse event rate, easier subcutaneous administration, and a significant improvement in health-related quality of life [113] compared to intravenous C1INH, represent an improvement in HAE therapy. The complement system plays a significant role in inflammation and graft rejection after transplantation, making C1INH a potential therapeutic option. The use of C1INH in a phase 1/2 study after kidney transplantation showed a significantly lower incidence of graft failure compared to placebo (3% in the treatment group vs. 21% in the placebo group) [114], while a phase 3 study of acute antibody-mediated graft rejection after kidney transplantation was terminated early because the predefined criteria for futility were met during a prescheduled interim analysis [115]. In a phase 3 trial in patients suffering from ACEi-induced angioedema, C1INH showed no superiority to the application of steroids or antihistamines [116].

Conestat alfa is an approved recombinant human C1INH for on-demand treatment of HAE attacks. A total of 99.8% of HAE attacks were adequately treated with a single dose [117]. A phase 4 open-label pilot study evaluated the effect of conestat alfa on adverse events of intravenous immunoglobulin (IVIG) therapy for autoimmune diseases associated with immunodeficiency or polyneuropathy [118]. In the small study population (n = 19), a significant reduction in adverse events such as headache, fatigue and migraine was observed compared to treatment with IVIG alone. The benefit of conestat alfa in the prevention of acute cerebral and renal ischemic events following transcatheter aortic valve implantation is being evaluated in a phase 2 study [119]. Recruitment is ongoing and the study is expected to end in 2025. Phase 1 data have not yet been published.

##### Drug candidates in clinical development

In clinical development, a phase 1/2 study with the investigational single administration gene therapy BMN331 has been ongoing since early 2022 [120]. BMN331 is an adeno-associated virus 5-based vector and designed to deliver the functional gene for C1INH (SERPING1) into the patient’s hepatocytes. Corresponding results might not be available before 2028 [120].

C1INH is a well-established first-line therapy for treating HAE. Other indications currently under investigation also affect small patient populations. However, they are in clinical development and may provide valuable contributions in the near future.

### B2 receptor

B2R is ubiquitously and constitutively expressed in healthy tissues. Its activation has proinflammatory effects caused by B2R-mediated vasodilation and increased vascular permeability with leukocyte migration [1]. Both receptor antagonists and agonists are currently in development.

#### Antagonists

##### Approved drugs

Icatibant is the first approved subcutaneous B2R antagonist for the on-demand treatment of HAE attacks and is also under investigation for its use in ACEi-induced upper airway angioedema and DIH. In a phase 3 study, icatibant demonstrated a significantly faster onset of symptom relief in acute HAE attacks than did placebo (2 h vs. 19.8 h) [121]. Overall, icatibant is an equally efficient alternative to C1INH in acute cases. A post hoc analysis of data from an observational study confirmed that a large proportion of HAE attacks (89.1%) can be treated with a single injection of icatibant in a real-world setting [122]. Regarding ACEi-induced upper airway angioedema conflicting results were reported. While a phase 2 study showed a significant reduction in the time to onset of symptom relief (2.0 h vs 11.7 h; p = 0.03) and complete resolution of symptoms (8.0 h vs 27.1 h; p = 0.002) [123], a phase 3 study showed no superiority to placebo [124]. Using icatibant in patients undergoing dialysis, a significantly lower incidence of DIH was shown in a small cohort of 22 patients (treatment group: one out of 11 patients vs placebo group: 7 out of 11 patients) [125]. A subsequent phase 3 study is planned to start in December 2023 [126].

##### Drug candidates in clinical development

Deucrictibant is intended for the on-demand treatment of HAE attacks. One advantage is that it can be administered orally rather than subcutaneously. No results have been published yet, but an FDA hold on the phase 2 trial was lifted in June 2023, and the manufacturer announced preparation for a phase 3 trial [127].

Two B2R antagonists, deltibant and anatibant, were developed for use in traumatic brain injury but did not proceed to phase 3 of clinical development. Deltibant failed to significantly reduce intracranial pressure [128]. A phase 2 trial using anatibant in patients with traumatic brain injury lost its funding during the course of the trial resulting in a reduced sample size and study power [129]. Fasitibant was developed for the treatment of patients with knee osteoarthritis but showed no superiority in a phase 2 study [130].

##### Drug candidates in preclinical development

A third B2R antagonist is still in preclinical investigation for the treatment of closed head trauma. In preclinical studies, compared with untreated animals, LF 18-15057 dose-dependently reduced brain edema by 4.5% and the neurological severity score improved when administered one hour after closed head trauma in rats [131].

The potential anti-inflammatory effects of three further B2R antagonists were investigated in various diseases in preclinical settings. FR173657 showed morphological and histological improvements in a psoriasis model in mice with a normalizing influence on keratinocyte proliferation. Furthermore, the results of the study indicate the involvement of both B2R and B1R. Knockout mice lacking one or both receptors were analyzed for comparison, and a single deletion of either receptor showed no effect [132]. Bradyzide is an orally available, nonpeptide B2R antagonist that shows long-lasting effects on reversing Freund´s complete adjuvant (FCA)-induced mechanical hyperalgesia in rodent models [133]. The potency of bradyzide is higher in rodents than in humans but it could be a therapeutic option for diseases that are associated with inflammatory hyperalgesia that require a continuous therapy (e. g. rheumatoid arthritis).

#### Agonists

##### Drug candidates in clinical development

Labradimil is a B2R agonist that increases the permeability of the blood–brain barrier (BBB). It has been studied in combination with carboplatin in various brain tumors to potentially increase carboplatin concentrations in the brain, which could improve efficacy [134]. Two phase 2 studies failed to observe enhanced responses in the treated group compared to carboplatin alone [134], and there was no significant improvement in the time to tumor progression [135].

##### Drug candidates in preclinical development

NG291 was tested in mice in comparison to labradimil and the endogenous agonist BK [136]. NG291 and labradimil both had stronger hypotensive, antithrombotic, and profibrinolytic effects than BK and may therefore have cardioprotective potential [136]. Another group showed a dose-dependent, reversible BBB disruption of NG291 in rats, as well as an activation of P-glycoprotein efflux transporters in mice, suggesting it can increase the concentration of therapeutics in the central nervous system if they are not P-glycoprotein substrates [137]. R523 was also tested in a glioma rat model and increased BBB permeability in a dose-dependent manner, as evidenced by a twofold increase in the amount of contrast agent in tumor cells and the peritumoral vasculature [138]. Considering the negative study results of labradimil, clinical relevance of this therapeutic approach has yet to be demonstrated.

Although promising for ocular hypotension, BK has poor ocular bioavailability and metabolic instability [139]. These challenges might be appropriately addressed by the development of the partial B2R agonist FR-190997. Topical application significantly decreased intraocular pressure in cynomolgus monkeys by 37.7 ± 5.4% 24 h after application and may therefore be a viable therapeutic option for treating glaucoma [139].

Compared to the targets discussed thus far, the exploration of antagonists and agonists of the B2R has been limited. Receptor internalization and desensitization curtail long-term effects via B2R, especially under conditions of inflammation [140]. The use of B2 agonists to disrupt the BBB in order to increase the concentration of other therapeutics in the brain seems conceivable but requires further preclinical and clinical research before actual implementation. Novel lead structures might be introduced by the determination of the crystal structure of B2R in 2022 empowering structure-based drug design [141].

### B1 receptor

B1R is synthesized de novo after tissue injury and its activation induces pain and inflammation, making it a potentially safe drug target in inflammatory diseases [142, 143]. To date, there are no approved drugs for this target, and new developments are generally less advanced than the targets discussed thus far. The main areas of research are diabetic retinopathies and pain. A total of 3 compounds (BI 1026706, safotibant and BAY 2395840) have entered clinical trials, with the remainder in preclinical development.

#### Antagonists

##### Drug candidates in clinical development

For the treatment of diabetic macular edema, the B1R antagonist BI 1026706 was evaluated in a 12-week, randomized, double-blind, placebo-controlled, twice-daily oral phase 2 study. No superiority over the placebo was shown in the primary endpoint, the reduction in central subfield foveal thickness [144]. Another B1R antagonist, safotibant, has shown promising results in diabetic Wistar rats by decreasing retinal plasma extravasation, leukostasis, elevated mRNA levels of B1R, and proinflammatory markers [145]. However, further development was discontinued after a phase 2 trial in humans without explanation. BAY 2395840 was developed as an oral treatment for diabetic neuropathic pain [146]. In April 2022, the manufacturer announced the termination of development on clinicaltrials.gov, as no superiority compared to placebo could be shown [146, 147].

##### Drug candidates in preclinical development

Another topical B1R antagonist is R-954. In preclinical studies in streptozotocin diabetic rats, it reversed the increase in B1R expression, leukostasis and vascular permeability and may be a therapeutic option for diabetic retinopathy if those findings can be confirmed in clinical trials [148].

Four other B1R antagonists for pain are in preclinical development. MK-0686, NVP-SAA164 and B9858 have all been shown to reduce FCA-induced hyperalgesia in animal models [149–151]. The first two are orally available and have been tested in mice. B9858 was tested in rabbits and administered intravenously. The fourth compound is ELN441958. It is orally administered and was able to reduce carrageenan-induced thermal hyperalgesia in the rhesus monkey tail withdrawal model [152].

In addition to diabetic retinopathy and pain, B1R antagonists have been studied in animal models of inflammatory skin diseases, liver fibrosis and cerebral ischemia. R-715 and SSR240612 have been developed as B1R antagonists for use in treating inflammatory skin diseases. Topical and intraperitoneal application of the combination of R-715 or SSR240612 with FR173657, a B2R antagonist, resulted in a significant reduction in cutaneous inflammation models in mice [153]. B1R and B2R play a role in the cutaneous neurogenic inflammatory response, as only double B1R and B2R knockout mice showed reduction in cutaneous inflammation, whereas single deletion of one of the receptors does not change their responses [153].

Since the B1R is involved in the pathogenesis of liver fibrosis [154], BI 113823 was tested in a mouse model of liver fibrosis [155]. It significantly attenuated liver fibrosis, normalized portal hypertension and reduced the expression of fibrotic proteins.

B 9430 is a B1R antagonist tested in the Mongolian gerbil for its effect on cerebral microcirculation after global cerebral ischemia [156]. Despite a decrease in the number of leukocytes rolling along the venular endothelium and the number of leukocytes adhering to the endothelial surface, no difference in the number of viable neurons was observed.

#### Agonists

##### Drug candidates in preclinical development

NG29 is a B1R agonist with potential application in neurological and ischemic cardiovascular diseases and brain tumors. Favorable toxicity and pharmacokinetics of NG29-TFacetate have been demonstrated in rats [157]. It has been shown, that B1R is overexpressed in rat brain gliomas and that the BBB disruption by B1R agonism can significantly increase the local concentration of platinum by a factor of 2 in cerebral tumors [158].

Most of the preclinical studies and the phase 2 study for safotibant are more than 10 years old, and the more recent studies have shown a lack of efficacy (BAY 2395840, BI 1026706). Overall, the B1R has been studied less extensively than the B2R, mainly due to the low homology between the human B1R and the rodent B1R [68% in mice [159], 71% in rats [160]], leading to limited transferability of results from animal models to humans. The identification of the crystal structure of the B1R in 2021 [161] could lead to significant advancements through structure-based drug design.

## Conclusion

In this review more than 65 drug candidates targeting the kallikrein-kinin system were identified. The latest (pre)clinical investigations indicate a far broader therapeutic significance of the kallikrein-kinin system beyond hereditary angioedema. Four new compounds are currently in phase 3 clinical development, with the potential for approval in the near future. However, only one of these compounds covers an indication other than HAE. Other research fields with drug candidates in phase 2 mainly encompass thromboprophylaxis and DME.

The recent unraveling of the human bradykinin receptor (B-R) structures holds the promise that improved development of drug candidates can now be initiated by structure-based drug design. Thus, further optimized drug candidates might become available for (pre)clinical testing and add to the current interest in drugs targeting the KKS.

### Supplementary Information

**Additional file 1.** References not indexed in databases

## Data Availability

References not indexed in databases are complied in Supplement 1. Further data available upon request.

## Abbreviations

AAV: Adeno-associated virus
ACE: Angiotensin-converting enzyme
ACEi: Angiotensin-converting enzyme inhibitor
AE-QoL: Angioedema Quality of Life Questionnaire
B1R: Bradykinin B1 receptor
B2R: Bradykinin B2 receptor
BBB: Blood–brain barrier
BCVA: Best corrected visual acuity
BK: Bradykinin
C1INH: C1 esterase inhibitor
CBP: Cardiopulmonary bypass
COVID-19: Coronavirus disease 2019
CRISPR/Cas9: Clustered regularly interspaced short palindromic repeats/CRISPR associated protein 9
DIH: Dialysis-induced hypotension
DME: Diabetic macular edema
EMA: European Medicines Agency
ERT: Enzyme replacement therapy
F12: Coagulation factor XII gene
FACAS: Coagulation factor XII-associated cold autoinflammatory disease
FCA: Freund’s complete adjuvant
FDA: U.S. Food and Drug Administration
FXII: Coagulation factor XII
FXIIa: Activated coagulation factor XII
HAE: Hereditary angioedema
HK: High molecular weight kininogen
HUK: Human urinary kallidinogenase
IVIG: Intravenous immunoglobulin
Ir-CPI: Ixodes ricinus-contact phase inhibitor
KKS: Kallikrein-kinin system
KLK1: Tissue kallikrein gene
KLKB1: Prekallikrein gene
LK: Low molecular weight kininogen
Lys-BK: Lysin-bradykinin/kallidin
Lys-BK 1–8: Lysin-bradykinin 1–8/kallidin 1–9
MCAO: Middle cerebral artery occlusion
MSCS score: Mean symptom complex severity score
PK: Plasma prekallikrein
PKa: Plasma kallikrein
PRCP: Prolylcarboxypeptidase
PRT: Protein replacement therapy
SERPING1: C1 esterase inhibitor gene
siRNA: Small interfering ribonucleic acid
TK: Tissue kallikrein
tMCAO: Transient middle cerebral artery
VEGF: Vascular endothelial growth factor
