# Supplementary material for: Recent advances in the discovery and development of drugs targeting the kallikrein-kinin system
Source: J Transl Med. 2024 Apr 26;22:388. doi: 10.1186/s12967-024-05216-5 (PMC11046790; doi:10.1186/s12967-024-05216-5)
Supplement: Supplementary file 1 — Additional file 1. References not indexed in databases [file 12967_2024_5216_MOESM1_ESM.pdf]

Table of Content

Ref.	Title	Page
29	CSL312 Safety, Pharmacokinetics, and Pharmacodynamics in Idiopathic Pulmonary Fibrosis	1
30	A Clinical Study to Test the Efficacy and Safety of CSL312 on Catheter-associated Blood Clot Formation in Subjects With Cancer Who Receive Chemotherapy Through a PICC Line	2
31	A Study to Evaluate the Safety, Tolerability and the Effects of Ixodes Ricinus-Contact Phase Inhibitor (Ir-CPI) in Adult Patients With Spontaneous Intracerebral Haemorrhage (BIRCH)	3
32	A Study to Evaluate the Safety, Tolerability, Pharmacokinetics, and Pharmacodynamics of Ir-CPI in Healthy Male Subjects	4-5
36	A MONOCLONAL ANTIBODY INHIBITOR OF FACTOR XIIA – European Patent for DX-4012	6-146
39	Arrowhead Pharmaceuticals Presents New Data on ARC-F12 and ARC-LPA Using DPCsqTM Subcutaneous RNAi Delivery Vehicle	147
41	Factor XIIa	148
57	BioCryst Announces FDA Approval of ORLADEYO™ (berotralstat), First Oral, Once-daily Therapy to Prevent Attacks in Hereditary Angioedema Patients	149
61	Sebetralstat for HAE	150
62	KalVista Pharmaceuticals Announces Termination of KVD824 Phase 2 KOMPLETE Trial for Prophylactic Treatment of Hereditary Angioedema	151
63	Attune Pharmaceuticals Announces Positive Phase 1 Results for ATN-249, an Oral Plasma Kallikrein Inhibitor for the Treatment of HAE	152-153
64	NTLA-2002 in Adults With Hereditary Angioedema (HAE) (NTLA-2002)	154
65	Intellia Therapeutics Announces Positive Interim Clinical Data for its Second Systemically Delivered Investigational CRISPR Candidate, NTLA-2002 for the Treatment of Hereditary Angioedema (HAE)	155
67	Efficacy and Safety of IONIS-PKKRx for Preventive Treatment of Chronic Migraine	156-157
68	The Ionis antisense pipeline	158
70	Safety, Tolerability, PK, PD of ADX-324 in Healthy Volunteers and Hereditary Angioedema Patients	159
72	KalVista Pharmaceuticals Reports Phase 2 Clinical Trial Results in Patients with Diabetic Macular Edema	160
74	Nonclinical safety and pharmacology of RZ402, a plasma kallikrein inhibitor, for the treatment of diabetic macular edema as a daily oral therapy	161
75	REZOLUTE ANNOUNCES INITIATION OF A PHASE 2 STUDY OF RZ402 IN PATIENTS WITH DIABETIC MACULAR EDEMA	162
76	Hemodialysis-Induced Hypotension Therapy for End Stage Kidney Disease (Hinder)	163
77	Inhibition of Plasma Kallikrein as a New Therapy for Lung Injury	164
79	Oral plasma kallikrein inhibitor KV998052 improves arterial blood oxygenation in a murine model of acute respiratory distress syndrome (ARDS)	165

88	Aprotinin Injection (marketed as Trasylol) Information	166
98	Phase II Study to Compare MDCO-2010 vs Placebo and Tranexamic Acid in Patients Undergoing Cardiac Surgery	167-168
103	DiaMedica Therapeutics Announces Clinical Hold of its Phase 2/3 ReMEDy2 Clinical Trial for DM199	169
104	DiaMedica Therapeutics Announces that the FDA Has Removed The Clinical Hold On DM199 Phase 2/3 Trial For Ischemic Stroke	170
105	A Study to Evaluate the Safety and Effectiveness of DM199 in Healthy Subjects and Type 2 Diabetes Patients	171
106	Multiple Doses of DM199 in Patients With Chronic Kidney Disease (REDUX)	172
115	A Multicenter Study to Evaluate the Efficacy and Safety of Cynrize® for the Treatment of Acute Antibody-mediated Rejection in Participants With Kidney Transplant	173
119	Recombinant Human C1 Esterase Inhibitor (Conestat Alfa) in the Prevention of Acute Ischemic Cerebral and Renal Events After Transcatheter Aortic Valve Implantation (PAIR-TAVI)	174
120	A Gene Therapy Study of BMN 331 in Subjects With Hereditary Angioedema (HAErmony-1)	175
126	Prevention of Intradialytic Hypotension by Inhibiting Bradykinin B2 Receptor	176
127	Pharvaris Announces FDA Removal of Clinical Hold of Deucrictibant for the On-Demand Treatment of HAE	177
130	Fasitibant Intra-articular Injection in Patients With Symptomatic Osteoarthritis of the Knee (ALBATROSS-3)	178-179
146	A Study to Learn How the Study Treatment BAY2395840 Moves Into, Through and Out of the Body, How Safe it is, and How it Affects the Body in Participants With Moderate Reduced Kidney Function and in Healthy Male and Female Participants With Normal Kidney Function	180
147	Clinical Study Results Synopsis: A randomized, double-blind, cross-over, placebo-controlled, multi-center, Phase 2a study to assess the safety and efficacy of BAY 2395840 in patients with diabetic neuropathic pain	181-187
163	Antisense Therapy to Block the Kallikrein-kinin Pathway in COVID-19 (ASKCOV)	188

This is the classic website, which will be retired eventually. Please visit the modernized ClinicalTrials.gov instead.

CSL312 Safety, Pharmacokinetics, and Pharmacodynamics in Idiopathic Pulmonary Fibrosis

The safety and scientific validity of this study is the responsibility of the study sponsor and investigators. Listing a study does not mean it has been evaluated by the U.S. Federal Government. Read our disclaimer for details.

ClinicalTrials.gov Identifier: NCT05130970

Recruitment Status : Completed
First Posted : November 23, 2021
Last Update Posted : February 9, 2024

View this study on the modernized ClinicalTrials.gov

Sponsor:
CSL Behring

Information provided by (Responsible Party):
CSL Behring

Study Details | Tabular View | No Results Posted | Disclaimer | How to Read a Study Record

Study Description

Brief Summary:
This is a prospective, phase 2a, multicenter, randomized, double-blind, placebo-controlled, parallel-group study to assess the safety, pharmacokinetics (PK), and pharmacodynamics (PD) of CSL312 in subjects with idiopathic pulmonary fibrosis (IPF).

Condition or disease	Intervention/treatment	Phase
Idiopathic Pulmonary Fibrosis	Drug: CSL312<br>Drug: Placebo	Phase 2

Study Design

Study Type : Interventional (Clinical Trial)
Actual Enrollment : 81 participants
Allocation: Randomized
Intervention Model: Parallel Assignment
Masking: Quadruple (Participant, Care Provider, Investigator, Outcomes Assessor)
Primary Purpose: Treatment
Official Title: A Randomized, Double-blind, Placebo-controlled, Study to Investigate the Safety, Pharmacokinetics, and Pharmacodynamics of CSL312 in Subjects With Idiopathic Pulmonary Fibrosis
Actual Study Start Date : January 27, 2022
Actual Primary Completion Date : January 2, 2024
Actual Study Completion Date : January 2, 2024

Resource links provided by the National Library of Medicine NIH NLM

MedlinePlus Genetics related topics: Idiopathic pulmonary fibrosis

MedlinePlus related topics: Pulmonary Fibrosis

Genetic and Rare Diseases Information Center resources: Idiopathic Pulmonary Fibrosis

U.S. FDA Resources

Arms and Interventions

Arm	Intervention/treatment
Experimental: CSL312<br>Administered IV and SC	Drug: CSL312<br>Fully human immunoglobulin G subclass 4/lambda recombinant monoclonal antibody<br>Other Names:<br>• Factor XIIa antagonist monoclonal antibody<br>• Garadacimab
Placebo Comparator: Placebo<br>Administered IV and SC	Drug: Placebo<br>Same as the CSL312 formulation buffer

Outcome Measures

Primary Outcome Measures :

1. Number of participants with treatment-emergent serious adverse events (SAEs) for CSL312 or placebo [Time Frame: Up to 22 weeks]
2. Percent of participants with SAEs for CSL312 or placebo [Time Frame: Up to 22 weeks]
3. Number of participants with treatment-emergent adverse events of special interest (AESIs) for CSL312 or placebo [Time Frame: Up to 22 weeks]
4. Percent of participants with AESIs for CSL312 or placebo [Time Frame: Up to 22 weeks]
5. Number of participants with treatment-emergent CSL312 induced antidrug antibodies (ADAs) [Time Frame: Up to 14 weeks]
6. Percent of participants with CSL312 induced ADAs [Time Frame: Up to 14 weeks]
7. Number of participants with treatment-emergent clinically significant abnormalities in laboratory assessments that are reported as adverse events (AEs) for CSL312 or placebo [Time Frame: Up to 14 weeks]
8. Percent of participants with treatment-emergent clinically significant abnormalities in laboratory assessments that are reported as adverse events (AEs) for CSL312 or placebo [Time Frame: Up to 14 weeks]

Secondary Outcome Measures :

1. Trough plasma concentration (Ctrough) after subcutaneous (SC) administration of CSL312 [Time Frame: Up to 14 weeks]
2. Maximum plasma concentration (Cmax) (last SC dosing interval only) of CSL312 [Time Frame: Up to 14 weeks]
3. Time to maximum plasma concentration (Tmax) (last SC dosing interval only) of CSL312 [Time Frame: Up to 14 weeks]
4. Area under the plasma concentration-time curve after the first dose interval (AUC0-tau) (last SC dosing interval only) of CSL312 [Time Frame: Up to 14 weeks]
5. Ctrough after intravenous (IV) administration of CSL312 [Time Frame: Up to 8 days]
6. Cmax after IV administration of CSL312 [Time Frame: Up to 8 days]
7. Tmax after IV administration of CSL312 [Time Frame: Up to 8 days]
8. Mean change from Baseline in FXIIa-mediated kallikrein activity of CSL312 [Time Frame: Up to 14 weeks]
9. Mean percentage of Baseline in FXIIa-mediated kallikrein activity of CSL312 [Time Frame: Up to 14 weeks]

Eligibility Criteria

Information from the National Library of Medicine NIH NLM

Choosing to participate in a study is an important personal decision. Talk with your doctor and family members or friends about deciding to join a study. To learn more about this study, you or your doctor may contact the study research staff using the contacts provided below. For general information, Learn About Clinical Studies.

Ages Eligible for Study: 40 Years and older (Adult, Older Adult)
Sexes Eligible for Study: All
Accepts Healthy Volunteers: No

Criteria

Inclusion Criteria:

- Male or female patients ≥ 40 years of age
- Documented diagnosis of IPF

Exclusion Criteria:

- History of clinically significant cardiovascular disease, including myocardial infarction, unstable ischemic heart disease, congestive heart failure, or angina during the 6 months before screening
- Sinoatrial or atrioventricular block, uncontrolled hypertension
- Active bleeding or current clinically significant coagulopathy

Contacts and Locations

Information from the National Library of Medicine NIH NLM

To learn more about this study, you or your doctor may contact the study research staff using the contact information provided by the sponsor.

Please refer to this study by its ClinicalTrials.gov identifier (NCT number): ***NCT05130970***

Locations

▸ Show 47 study locations

Sponsors and Collaborators

CSL Behring

Investigators

Study Director: Study Director CSL Behring

More Information

Responsible Party: CSL Behring
ClinicalTrials.gov Identifier: NCT05130970 History of Changes
Other Study ID Numbers: CSL312_2002
2021 003162 12 (EudraCT Number)
First Posted: November 23, 2021 Key Record Dates
Last Update Posted: February 9, 2024
Last Verified: February 2024

Individual Participant Data (IPD) Sharing Statement:
Plan to Share IPD: Yes
Plan Description: CSL will consider requests to share Individual Patient Data (IPD) from systematic review groups or bona-fide researchers. For information on the process and requirements for submitting a voluntary data sharing request for IPD, please contact CSL at clinicaltrials@cslbehring.com.

Applicable country specific privacy and other laws and regulations will be considered and may prevent sharing of IPD.

If the request is approved and the researcher has executed an appropriate data sharing agreement, IPD that has been appropriately anonymized will be available.

Supporting Materials: Study Protocol
Statistical Analysis Plan (SAP)
Time Frame: IPD requests may be submitted to CSL no earlier than 12 months after publication of the results of this study via an article made available on a public website.
Access Criteria: Requests may only be made by systematic review groups or bona-fide researchers whose proposed use of the IPD is non-commercial in nature and has been approved by an internal review committee.

An IPD request will not be considered by CSL unless the proposed research question seeks to answer a significant and unknown medical science or patient care question as determined by CSL's internal review committee.

The requesting party must execute an appropriate data sharing agreement before IPD will be made available.

Studies a U.S. FDA-regulated Drug Product: Yes
Studies a U.S. FDA-regulated Device Product: No

Additional relevant MeSH terms:

Pulmonary Fibrosis	Lung Diseases, Interstitial
Idiopathic Pulmonary Fibrosis	Lung Diseases
Fibrosis	Respiratory Tract Diseases
Pathologic Processes	

This is the classic website, which will be retired eventually. Please visit the modernized ClinicalTrials.gov instead.

A Clinical Study to Test the Efficacy and Safety of CSL312 on Catheter-associated Blood Clot Formation in Subjects With Cancer Who Receive Chemotherapy Through a PICC Line

The safety and scientific validity of this study is the responsibility of the study sponsor and investigators. Listing a study does not mean it has been evaluated by the U.S. Federal Government. Read our disclaimer for details.

ClinicalTrials.gov Identifier: NCT04281524

Recruitment Status : Withdrawn (Business decision non-safety related.)
First Posted : February 24, 2020
Last Update Posted : March 25, 2020

View this study on the modernized ClinicalTrials.gov

Sponsor:
CSL Behring

Information provided by (Responsible Party):
CSL Behring

Study Description

Brief Summary:
Peripherally Inserted Central Catheters (PICCs) are commonly used in patients with cancer to administer chemotherapy and supportive care medication. However, PICCs and other medical devices that come into contact with blood increase the risk of blood clots (thrombosis) inside the blood vessels. Conventional blood thinners (anticoagulants) may reduce the risk of thrombosis but they also increase the risk of bleeding. CSL312, a monoclonal antibody that inhibits the activated blood clotting factor 12 (FXIIa) will be assessed for its potential to prevent thrombus formation in subjects with cancer at risk of PICC-associated thrombosis.

Condition or disease	Intervention/treatment	Phase
PICC-associated Thrombosis	Drug: CSL312<br>Drug: Placebo	Phase 1<br>Phase 2

Study Design

Study Type : Interventional (Clinical Trial)
Actual Enrollment : 0 participants
Allocation: Randomized
Intervention Model: Parallel Assignment
Masking: Quadruple (Participant, Care Provider, Investigator, Outcomes Assessor)
Primary Purpose: Prevention
Official Title: A Phase 1b, Multi-center, Randomized, Double-blind, Placebo-controlled Study to Investigate the Efficacy, Safety, and Pharmacokinetics of CSL312 in the Prevention of Peripherally Inserted Central Catheter (PICC)-Associated Thrombosis in Subjects With Cancer
Estimated Study Start Date : March 2020
Estimated Primary Completion Date : August 2021
Estimated Study Completion Date : October 2021

Resource links provided by the National Library of Medicine

MedlinePlus related topics: Blood Clots

U.S. FDA Resources

Arms and Interventions

Arm	Intervention/treatment
Experimental: CSL312 Cohort 1 (Dose 1)<br>CSL312 administered as IV infusion	Drug: CSL312<br>CSL312 administered as an IV infusion<br>Other Name: **Garadacimab**
Experimental: CSL312 Cohort 2 (Dose 2)<br>CSL312 administered as IV infusion	Drug: CSL312<br>CSL312 administered as an IV infusion<br>Other Name: **Garadacimab**
Experimental: CSL312 Cohort 3 (Dose 3)<br>CSL312 administered as IV infusion	Drug: CSL312<br>CSL312 administered as an IV infusion<br>Other Name: **Garadacimab**
Experimental: CSL312 Cohort 4 (Dose 4)<br>CSL312 administered as IV infusion	Drug: CSL312<br>CSL312 administered as an IV infusion<br>Other Name: **Garadacimab**
Placebo Comparator: Placebo<br>Placebo administered as IV infusion	Drug: Placebo<br>Solution of 70% 0.9% saline / 30% CSL312 diluent

Outcome Measures

Primary Outcome Measures :

1. Number of subjects with PICC-associated thrombosis [Time Frame: Up to 29 days after PICC insertion]

   PICC-associated thrombosis which can be either:

   a. Asymptomatic PICC associated thrombosis detected by Duplex ultrasound (DUS) or venography at Day 15 or Day 29 after PICC insertion or
   b. Symptomatic PICC associated thrombosis up to Day 29 after PICC insertion, suspected clinically due to symptoms of the upper limb or neck, and objectively confirmed by DUS or venography

2. Percent of subjects with PICC-associated thrombosis [Time Frame: Up to 29 days after PICC insertion]

   PICC-associated thrombosis which can be either:

   a. Asymptomatic PICC associated thrombosis detected by Duplex ultrasound (DUS) or venography at Day 15 or Day 29 after PICC insertion or
   b. Symptomatic PICC associated thrombosis up to Day 29 after PICC insertion, suspected clinically due to symptoms of the upper limb or neck, and objectively confirmed by DUS or venography

Secondary Outcome Measures :

1. Overall percentage of subjects with treatment emergent adverse events (TEAEs) and serious adverse events (SAEs) [Time Frame: Up to 110 days after first dose of CSL312]
2. Percent of subjects with related TEAEs [Time Frame: Up to 110 days after first dose of CSL312]
3. Percent of subjects with TEAEs by severity [Time Frame: Up to 110 days after first dose of CSL312]
4. Number of subjects treated with CSL312 with detectable antibodies to CSL312 [Time Frame: Up to 110 days after first dose of CSL312]
5. Percent of subjects treated with CSL312 with detectable antibodies to CSL312 [Time Frame: Up to 110 days after first dose of CSL312]
6. Maximum plasma concentration (Cmax) of CSL312 [Time Frame: Up to 110 days after first dose of CSL312]
7. Area under the concentration-time curve (AUC0-t) of CSL312 [Time Frame: Up to 110 days after first dose of CSL312]
8. Time of maximum plasma concentration (Tmax) of CSL312 [Time Frame: Up to 110 days after first dose of CSL312]
9. Terminal elimination half-life (T1/2) of CSL312 [Time Frame: Up to 110 days after first dose of CSL312]
10. Total systemic clearance (CLtot) of CSL312 [Time Frame: Up to 110 days after first dose of CSL312]
11. Volume of distribution during the elimination phase (Vz) of CSL312 [Time Frame: Up to 110 days after first dose of CSL312]
12. Accumulation Ratio (AR) of CSL312 [Time Frame: Up to 110 days after first dose of CSL312]
13. Number of subjects with thrombosis-associated catheter occlusion [Time Frame: Up to 29 days after first dose of CSL312]
14. Percent of subjects with thrombosis-associated catheter occlusion [Time Frame: Up to 29 days after first dose of CSL312]
15. Number of subjects with PICC removal or replacement [Time Frame: Up to 29 days after first dose of CSL312]
16. Percent of subjects with PICC removal or replacement [Time Frame: Up to 29 days after first dose of CSL312]
17. Number of subjects with central line-associated blood stream infections (CLABSI) [Time Frame: Up to 29 days after first dose of CSL312]
18. Percent of subjects with CLABSI [Time Frame: Up to 29 days after first dose of CSL312]

Eligibility Criteria

Information from the National Library of Medicine

Choosing to participate in a study is an important personal decision. Talk with your doctor and family members or friends about deciding to join a study. To learn more about this study, you or your doctor may contact the study research staff using the contacts provided below. For general information, Learn About Clinical Studies.

Ages Eligible for Study: 18 Years and older (Adult, Older Adult)
Sexes Eligible for Study: All
Gender Based Eligibility: Yes
Accepts Healthy Volunteers: No

Criteria

Inclusion Criteria:

- Aged 18 years or older at the time of providing written informed consent
- Diagnosis of malignancy that requires placement of a PICC within the next 3 weeks for administration of chemotherapy (PICC anticipated to be required for at least 1 month)
- Eastern Cooperative Oncology Group (ECOG) performance status of 0, 1, or 2 [Oken et al, 1982], and investigator's expectation that performance status will remain 0, 1, or 2 for the duration of the study

Exclusion Criteria:

- Active bleeding or with a current clinically significant coagulopathy (eg, international normalized ratio [INR] > 1.5) or clinically significant risk for bleeding (eg, recent intracranial hemorrhage or bleeding peptic ulcer within the last 4 weeks)
- History of venous thrombosis, myocardial infarction or cerebrovascular event within 3 months, or a prothrombotic disorder (eg, antithrombin III, protein C or S deficiency)
- Life expectancy less than study duration (110 days)
- Platelet count of < 20 × 109/L on the day of dose 1 (Day 1) or within 7 days before first dosing
- Estimated glomerular filtration rate (eGFR) < 30 mL/min/1.73 m2
- Treatment with antiplatelet or anticoagulant medication, including thrombosis prophylaxis, within 10 days prior to insertion of the PICC
- Chemotherapy regimen that would be expected to drop the platelet count to < 20 × 109/L
- Chemotherapy regimen with heparin mixed into IV bags (eg, dalteparin 2500 IU/day)
- Difficult IV access that would prevent infusion of the IP
- In situ central venous catheter (CVC) or PICC in the 3 months before the Screening Visit. The study PICC must be inserted in the contralateral side, which must be PICC / CVC naïve
- Undergoing dialysis or have another inserted intravascular foreign surface device
- Serum aspartate aminotransferase (AST) and serum alanine aminotransferase (ALT) ≥ 4 × upper limit of normal

Contacts and Locations

Information from the National Library of Medicine

To learn more about this study, you or your doctor may contact the study research staff using the contact information provided by the sponsor.

*Please refer to this study by its ClinicalTrials.gov identifier (NCT number): **NCT04281524***

Sponsors and Collaborators

CSL Behring

Investigators

Study Director: Study Director CSL Behring

More Information

Responsible Party: CSL Behring
ClinicalTrials.gov Identifier: NCT04281524 History of Changes
Other Study ID Numbers: CSL312_1002
First Posted: February 24, 2020 Key Record Dates
Last Update Posted: March 25, 2020
Last Verified: March 2020

Individual Participant Data (IPD) Sharing Statement:
Plan to Share IPD: Yes
Plan Description: CSL will consider requests to share Individual Patient Data (IPD) from systematic review groups or bona-fide researchers. For information on the process and requirements for submitting a voluntary data sharing request for IPD, please contact CSL at clinicaltrials@cslbehring.com.

Applicable country specific privacy and other laws and regulations will be considered and may prevent sharing of IPD.

If the request is approved and the researcher has executed an appropriate data sharing agreement, IPD that has been appropriately anonymized will be available.

Supporting Materials: Study Protocol
Statistical Analysis Plan (SAP)
Time Frame: IPD requests may be submitted to CSL no earlier than 12 months after publication of the results of this study via an article made available on a public website.
Access Criteria: Requests may only be made by systematic review groups or bona-fide researchers whose proposed use of the IPD is non-commercial in nature and has been approved by an internal review committee.

An IPD request will not be considered by CSL unless the proposed research question seeks to answer a significant and unknown medical science or patient care question as determined by CSL's internal review committee.

The requesting party must execute an appropriate data sharing agreement before IPD will be made available.

Studies a U.S. FDA-regulated Drug Product: No
Studies a U.S. FDA-regulated Device Product: No

Additional relevant MeSH terms:
Thrombosis
Embolism and Thrombosis
Vascular Diseases
Cardiovascular Diseases

This is the classic website, which will be retired eventually. Please visit the modernized ClinicalTrials.gov instead.

Notice to API users: Read about imminent changes impacting usage of the ClinicalTrials.gov API.

A Study to Evaluate the Safety, Tolerability and the Effects of Ixodes Ricinus-Contact Phase Inhibitor (Ir-CPI) in Adult Patients With Spontaneous Intracerebral Haemorrhage (BIRCH)

The safety and scientific validity of this study is the responsibility of the study sponsor and investigators. Listing a study does not mean it has been evaluated by the U.S. Federal Government. Know the risks and potential benefits of clinical studies and talk to your health care provider before participating. Read our disclaimer for details.

ClinicalTrials.gov Identifier: NCT05970224

Recruitment Status : Not yet recruiting
First Posted : August 1, 2023
Last Update Posted : August 1, 2023
See **Contacts and Locations**

View this study on the modernized ClinicalTrials.gov

Sponsor:
Bioxodes S.A.

Information provided by (Responsible Party):
Bioxodes S.A.

Study Description

Brief Summary:
The purpose of the study is to provide a first assessment of safety, tolerability and efficacy of **Ir-CPI**, administered on top of standard-of-care, on secondary brain injury in patients with spontaneous intracerebral haemorrhage.

Condition or disease	Intervention/treatment	Phase
Intracerebral Hemorrhage	Drug: **Ir-CPI**	Phase 2

Study Design

Study Type : Interventional (Clinical Trial)
Estimated Enrollment : 32 participants
Allocation: Randomized
Intervention Model: Parallel Assignment
Masking: None (Open Label)
Primary Purpose: Treatment
Official Title: A Phase IIa, Randomized, Open-label, Proof-of-Concept Study to Evaluate Safety, Tolerability and Efficacy of **Ir-CPI** in Patients With Spontaneous Intracerebral Haemorrhage
Estimated Study Start Date : July 31, 2023
Estimated Primary Completion Date : August 2025
Estimated Study Completion Date : July 2027

Resource links provided by the National Library of Medicine

MedlinePlus related topics: Bleeding

U.S. FDA Resources

Arms and Interventions

Arm	Intervention/treatment
Experimental: **Ir-CPI**<br>**Ir-CPI** will be administered on top of standard of care	Drug: **Ir-CPI**<br>Participants receive a single intravenous dose of **Ir-CPI** during 48 hours
No Intervention: Standard care<br>Only standard of care	

Outcome Measures

Primary Outcome Measures :

1. Adverse events monitoring [Time Frame: 360 days post-randomization]
2. Incidence of abnormalities in physical examination [Time Frame: 7 days post-randomization]

   A complete physical examination will include, at a minimum, assessments of the cardiovascular, respiratory, gastrointestinal, dermatological, neurological (including basic neurological testing for isocoria, light reflexes, gait and balance), musculoskeletal and lymphatic systems, in addition to head, eyes, ears, nose, throat, and neck.

3. Change from baseline in HR interval [Time Frame: 7 days post-randomization]

   Measured by standard 12-lead ECG

4. Change from baseline in PR interval [Time Frame: 7 days post-randomization]

   Measured by standard 12-lead ECG

5. Change from baseline in QRS duration [Time Frame: 7 days post-randomization]

   Measured by standard 12-lead ECG

6. Change from baseline in QRS axis [Time Frame: 7 days post-randomization]

   Measured by standard 12-lead ECG

7. Change from baseline in QT interval [Time Frame: 7 days post-randomization]

   Measured by standard 12-lead ECG. Two corrections of the QT interval will be investigated: Fridericia's correction (QTcF) and Bazett's correction (QTcB)

8. Change from baseline in blood pressure [Time Frame: 7 days post-randomization]

   Blood pressure (systolic and diastolic) is measured using an automatic device

9. Change from baseline in heart rate [Time Frame: 7 days post-randomization]

   Heart rate is measured using an automatic device

10. Change from baseline in body temperature [Time Frame: 7 days post-randomization]

    Measurement of tympanic temperature

Secondary Outcome Measures :

1. Change from baseline in perihematomal oedema (PHO) and haemorrhage volumes [Time Frame: 10 days post-randomization]

   CT scans will be acquired by volumetric CT acquisition with reconstructions in 3 planes, in order to assess hematoma volume and perihematomal volume. Assessment of hematoma expansion will be performed by comparing follow-up CT scans with baseline CT.

2. Measurement of the effect of **Ir-CPI** on the activated Partial Thromboplastin Time (aPTT) [Time Frame: 7 days post-randomization]

   Activated partial thromboplastin time (aPTT) will be used as a pharmacodynamic marker

3. Measurement of the effect of **Ir-CPI** on the inhibition of Factor XI (FXI) and Factor XII (FXII) procoagulant activities [Time Frame: 7 days post-randomization]

   The inhibition of Factor XI (FXI) and Factor XII (FXII) procoagulant activities will be assessed to support the aPTT dynamics

4. Change from baseline in **Ir-CPI** plasma concentrations [Time Frame: 7 days post-randomization]

Eligibility Criteria

Information from the National Library of Medicine

Choosing to participate in a study is an important personal decision. Talk with your doctor and family members or friends about deciding to join a study. To learn more about this study, you or your doctor may contact the study research staff using the contacts provided below. For general information, Learn About Clinical Studies.

Ages Eligible for Study: 18 Years and older (Adult, Older Adult)
Sexes Eligible for Study: All
Accepts Healthy Volunteers: No

Criteria

Inclusion Criteria:

- Male or female patients aged ≥ 18 years.
- Written informed consent obtained before any study assessment. If the patient is not able to give the informed consent personally, consent by a legal representative as defined by local law and regulation is acceptable.
- First-ever, spontaneous, supratentorial intracerebral haemorrhage in cerebral cortex or deep brain structures (putamen, thalamus, caudate, and associated deep white matter tracts) with a volume ≥ 5 mL and ≤ 60 mL determined by non-contrast CT scan.
- Patients with Glasgow Coma Scale (GCS) best motor score no less than 5.
- Modified Rankin Scale (mRS) score 0-2 prior to ICH symptom onset.

Exclusion Criteria:

- History of personal or familial bleeding disorders; including prolonged or unusual bleeding.
- Known deficiency in factor XII (FXII) or haemophilia type A (FVII) or type B (FIX) or type C (FXI).
- Infratentorial (midbrain, pons, medulla, or cerebellum) ICH.
- Secondary ICH due to aneurysm, brain tumour, arteriovenous malformation, thrombocytopenia, coagulopathy, acute sepsis, traumatic brain injury (TBI), or disseminated intravascular coagulation (DIC).
- Planned neurosurgical hematoma evacuation or other urgent surgical intervention (i.e., surgical relief of increased intracranial pressure) on initial presentation.
- Planned anticoagulation reversal treatment.
- Patients with intraventricular haemorrhage (IVH) having a Graeb score of >3 on initial presentation. Patients must not have blood in the 4th ventricle and may only have blood in the 3rd ventricle in the absence of ventricular expansion. Trace or mild haemorrhage in either or both lateral ventricles is permitted. Patients with hydrocephalus determined radiologically on initial presentation are excluded regardless of Graeb score.
- Use of immunosuppressive or immune-modulating therapy at admission (e.g., steroids, methotrexate, monoclonal antibodies, etc).
- Patients with active systemic bacterial, viral or fungal infections.
- Women of childbearing potential.
- Have a body weight > 120 kg at screening.
- Severe renal impairment (eGFR < 30 mL/min/1.73 m2).

Contacts and Locations

Information from the National Library of Medicine

To learn more about this study, you or your doctor may contact the study research staff using the contact information provided by the sponsor.

*Please refer to this study by its ClinicalTrials.gov identifier (NCT number): **NCT05970224***

Contacts

Contact: Charlotte Corbisier +32 (0)472 21 01 20 charlotte.corbisier@bioxodes.com

Locations

Belgium

UZ Gent
Gent, East Flanders, Belgium, 9000
Contact: Wendy Stoop +32 (0) 93320449 wendy.stoop@uzgent.be

CHU Ambroise Paré
Mons, Hainaut, Belgium, 7000
Contact: Virginie Vanderhaegen + 32 (0) 65 41 75 17 virginie.vanderhaegen@hap.be

AZ Groeninge
Kortrijk, West Flanders, Belgium, 8500
Contact: Isabelle Vanpantghem +32 (0)56 63 34 26 isabelle.vanpanteghem@azgroeninge.be

Sponsors and Collaborators

Bioxodes S.A.

More Information

Responsible Party: Bioxodes S.A.
ClinicalTrials.gov Identifier: NCT05970224 History of Changes
Other Study ID Numbers: Clin_IrCPI_201
2022-500491-53-00 (Registry Identifier: CTIS)
First Posted: August 1, 2023 Key Record Dates
Last Update Posted: August 1, 2023
Last Verified: July 2023

Individual Participant Data (IPD) Sharing Statement:
Plan to Share IPD: No

Studies a U.S. FDA-regulated Drug Product: No
Studies a U.S. FDA-regulated Device Product: No

Keywords provided by Bioxodes S.A.:
Ir-CPI
ICH
Secondary brain injury
Ixodes ricinus-Contact Phase Inhibitor
Neutrophil
Antithrombotic
Intracranial hemorrhage
Stroke
Brain
Inflammation
Neuroinflammation
Cerebrovascular disorders

Additional relevant MeSH terms:
Cerebral Hemorrhage
Hemorrhage
Pathologic Processes
Intracranial Hemorrhages
Cerebrovascular Disorders
Brain Diseases
Central Nervous System Diseases
Nervous System Diseases
Vascular Diseases
Cardiovascular Diseases

This is the classic website, which will be retired eventually. Please visit the modernized ClinicalTrials.gov instead.

A Study to Evaluate the Safety, Tolerability, Pharmacokinetics, and Pharmacodynamics of Ir-CPI in Healthy Male Subjects

The safety and scientific validity of this study is the responsibility of the study sponsor and investigators. Listing a study does not mean it has been evaluated by the U.S. Federal Government. Read our disclaimer for details.

ClinicalTrials.gov Identifier: NCT04653766

Recruitment Status : Completed
First Posted : December 4, 2020
Results First Posted : May 27, 2021
Last Update Posted : February 23, 2023

View this study on the modernized ClinicalTrials.gov

Sponsor:
Bioxodes S.A.

Information provided by (Responsible Party):
Bioxodes S.A.

Study Details | Tabular View | Study Results | Disclaimer | How to Read a Study Record

Study Description

Brief Summary:
The purpose of this First-in-Human study is to assess the safety, tolerability, pharmacokinetics, and pharmacodynamics of **Ir-CPI**, a novel dual inhibitor of FXIIa and FXIa, following IV administration of single ascending doses in healthy male volunteers.

Condition or disease	Intervention/treatment	Phase
Healthy	Drug: **Ir-CPI** - Dose 1<br>Drug: **Ir-CPI** - Dose 2<br>Drug: **Ir-CPI** - Dose 3<br>Drug: **Ir-CPI** - Dose 4<br>Drug: Placebo	Phase 1

Study Design

Study Type :	Interventional (Clinical Trial)
Actual Enrollment :	32 participants
Allocation:	Randomized
Intervention Model:	Sequential Assignment
Masking:	Double (Participant, Investigator)
Primary Purpose:	Other
Official Title:	A Phase I, Double Blind, Placebo Controlled, Single Ascending Dose Study of Intravenously Administered **Ir-CPI** to Evaluate Pharmacokinetics, Pharmacodynamics, Safety and Tolerability in Healthy Male Volunteers
Actual Study Start Date :	September 12, 2019
Actual Primary Completion Date :	July 18, 2020
Actual Study Completion Date :	January 4, 2023

Resource links provided by the National Library of Medicine

MedlinePlus related topics: Safety

U.S. FDA Resources

Arms and Interventions

Arm	Intervention/treatment
Experimental: **Ir-CPI** - Dose 1<br>Participants received a single intravenous dose of 1.5 mg/kg of **Ir-CPI** during 6 hours	Drug: **Ir-CPI** - Dose 1<br>6 participants received a single intravenous dose of 1.5 mg/kg of **Ir-CPI** during 6 hours
Experimental: **Ir-CPI** - Dose 2<br>Participants received a single intravenous dose of 3.0 mg/kg of **Ir-CPI** during 6 hours	Drug: **Ir-CPI** - Dose 2<br>6 participants received a single intravenous dose of 3.0 mg/kg of **Ir-CPI** during 6 hours
Experimental: **Ir-CPI** - Dose 3<br>Participants received a single intravenous dose of 6.0 mg/kg of **Ir-CPI** during 6 hours	Drug: **Ir-CPI** - Dose 3<br>6 participants received a single intravenous dose of 6.0 mg/kg of **Ir-CPI** during 6 hours
Experimental: **Ir-CPI** - Dose 4<br>Participants received a single intravenous dose of 9.0 mg/kg of **Ir-CPI** during 6 hours	Drug: **Ir-CPI** - Dose 4<br>6 participants received a single intravenous dose of 9.0 mg/kg of **Ir-CPI** during 6 hours
Placebo Comparator: Placebo<br>Participants received a single intravenous dose of placebo during 6 hours	Drug: Placebo<br>For each dose group, 2 additional participants received a single intravenous dose of placebo during 6 hours (8 in total).

Outcome Measures

Primary Outcome Measures :

1. Measurement of the Safety Lab Parameter Activated Partial Thromboplastin Time (aPTT) [Time Frame: At pre-dose (baseline), 2, 4, 6, 8, 12, 24, 48, 72 hours post-dose and at the discharge visit (Day 10 ± 2 days)]

   These safety aPTT results were readily available to the PI for safety follow-up during and after the study drug administration. Safety aPTT was followed up "at the bedside" at regular time-points (as opposed to the PD biomarker aPTT, which was assessed by the central laboratory alongside the PK time-points). An aPTT ratio was calculated by dividing the aPTT value (in sec) of the specific time-point (aPTTt) by the baseline aPTT value (in sec) (aPTTbaseline) of the same participant (aPTT ratio = aPTTt/aPTTbaseline). The baseline time-point was considered as having an aPTT ratio of 1.

   Concerning the row titles, for example, Day 1 H02:00 corresponds to aPTT ratio results obtained on Day 1, 2 hours after the start of the infusion.

Secondary Outcome Measures :

1. Measurement of the Maximum Plasma Concentration (Cmax) of **Ir-CPI** [Time Frame: Pre-dose, 0.5, 1, 1.5, 2, 4, 6, 6.5, 7, 7.5, 8, 10, 12, 16, 24, 48, 72, 96, 144 hours (144 hours for Dose 4 only)]
2. Measurement of the Time to Reach Maximum Plasma Concentration (Tmax) of **Ir-CPI** [Time Frame: Pre-dose, 0.5, 1, 1.5, 2, 4, 6, 6.5, 7, 7.5, 8, 10, 12, 16, 24, 48, 72, 96, 144 hours (144 hours for Dose 4 only)]
3. Measurement of the Area Under the Plasma Concentration-time Curve From Time Zero to 6h (AUC0-6) and From Time Zero to Time of Infinity (AUCinf) [Time Frame: Pre-dose, 0.5, 1, 1.5, 2, 4, 6, 6.5, 7, 7.5, 8, 10, 12, 16, 24, 48, 72, 96, 144 hours (144 hours for Dose 4 only)]
4. Measurement of the Effect of **Ir-CPI** on the Activated Partial Thromboplastin Time (aPTT) [Time Frame: Pre-dose (baseline), 0.5, 1, 1.5, 2, 4, 6, 6.5, 7, 7.5, 8, 10, 12, 16, 24, 48, 72, 96 hours post-dose and at the discharge visit (Day 10 ± 2 days)]

   The activated partial thromboplastin time (aPTT) is used as a pharmacodynamic marker. No data were available for the **Ir-CPI** 1.5 mg/kg group because the results were not usable due to plasma preparation and method repeatability problems. An aPTT ratio was calculated by dividing the aPTT value (in sec) of the specific time-point (aPTTt) by the baseline aPTT value (in sec) (aPTTbaseline) of the same participant (aPTT ratio = aPTTt/aPTTbaseline). The baseline time-point (Day 1 H00:00) was considered as having an aPTT ratio of 1.

   Concerning the row titles, for example, Day 1 H02:00 corresponds to aPTT ratio results obtained on Day 1, 2 hours after the start of the infusion.

5. Measurement of the Effect of **Ir-CPI** on Factor XI Activity [Time Frame: Pre-dose (baseline), 0.5, 1, 1.5, 2, 4, 6, 6.5, 7, 7.5, 8, 10, 12, 16, 24, 48, 72, 96 hours post-dose and at the discharge visit (Day 10 ± 2 days)]

   The method is a clotting assay where all the clotting factors are present (constant and in excess, brought by the deficient plasma), excepted for FXI, which is brought by the diluted tested plasma, and clotting is triggered with cephalin, activator and calcium (aPTT). FXI is the limiting factor and clotting time is inversely proportional to the concentration of FXI. There is an inverse linear relationship between the percentage of FXI activity and the corresponding clotting time. Central laboratory normal ranges for Factor XI activity are between 65 and 150 %.

   Concerning the row titles, for example, Day 1 H02:00 corresponds to the percentage of FXI activity obtained on Day 1, 2 hours after the start of the infusion.

6. Measurement of the Effect of **Ir-CPI** on Factor XII Activity [Time Frame: Pre-dose (baseline), 0.5, 1, 1.5, 2, 4, 6, 6.5, 7, 7.5, 8, 10, 12, 16, 24, 48, 72, 96 hours post-dose and at the discharge visit (Day 10 ± 2 days)]

   The method is a clotting assay where all the clotting factors are present (constant and in excess, brought by the deficient plasma), excepted for FXII, which is brought by the diluted tested plasma, and clotting is triggered with cephalin, activator and calcium (aPTT). FXII is the limiting factor and clotting time is inversely proportional to the concentration of FXII. There is an inverse linear relationship between the percentage of FXII activity and the corresponding clotting time. Central laboratory normal ranges for Factor XII activity are between 50 and 150 %.

   Concerning the row titles, for example, Day 1 H02:00 corresponds to the percentage of FXII activity obtained on Day 1, 2 hours after the start of the infusion.

Eligibility Criteria

Information from the National Library of Medicine

Choosing to participate in a study is an important personal decision. Talk with your doctor and family members or friends about deciding to join a study. To learn more about this study, you or your doctor may contact the study research staff using the contacts provided below. For general information, Learn About Clinical Studies.

Ages Eligible for Study:	18 Years to 55 Years (Adult)
Sexes Eligible for Study:	Male
Accepts Healthy Volunteers:	Yes

Criteria

Inclusion Criteria:

Participants must satisfy all of the following inclusion criteria before being allowed to enter the study:

1. Have given written informed consent approved by the relevant Ethics Committee (EC) governing the site, indicating that they understand the purpose of and procedures required for the study and are willing to participate in the study.
2. Male participants between 18 and 55 years of age, inclusive at screening.
3. Otherwise healthy with no clinically significant abnormalities as determined by medical history, physical examination, blood chemistry assessments, haematological assessments, coagulation and urinalysis, measurement of vital signs, and ECG. Isolated out-of-range values judged by the PI (or designated physician) to be of no clinical significance can be allowed. This determination must be recorded in the participant's source documents.
4. Have a body weight in the range of 50 to 90 kg inclusive at screening. Have a body mass index (BMI) of 19 to 28 kg/m2 inclusive at screening.
5. Agree to abstain from alcohol intake 24 hours before administration of study drug, during the in-patient period of the study and 24 hours prior to all other ambulatory visits, up until and including the discharge visit.
6. Agree not to use prescription medications within 14 days prior to study drug administration and through the duration of the study, unless approved by the PI and Sponsor medical monitor.
7. Non-smokers or abstinence from tobacco or nicotine-containing products for at least 3 months prior to screening.
8. Agree not to use over-the-counter (OTC) medications [including decongestants, antihistamines, and herbal medication (including herbal tea and St. John's Wort)], within 14 days prior to study drug administration through the discharge visit, unless approved by the PI and Sponsor medical monitor. Occasional use of paracetamol at recommended doses is allowed. Special rules apply for aspirin, corticosteroids and nonsteroidal anti-inflammatory drugs (NSAIDS (see exclusion criteria 6-7-8-9)
9. Venous access (both arms) sufficient to allow blood sampling and study drug administration as per protocol.
10. Participants and their partners of childbearing potential [meaning who are not surgically sterile (tubal ligation/obstruction or removal of ovaries or uterus) or post-menopausal (absence of menstrual periods for at least 12 consecutive months)] must be willing to use 2 methods of contraception: - a highly effective method of birth control starting at screening. Highly effective methods of birth control are defined as those that result in a low failure rate (i.e. Pearl Index less than 1% per year) when used consistently and correctly, such as implants, rings, patches, injectable or combined oral contraceptives, intrauterine devices (IUDs), or sexual abstinence (periodic abstinence e.g. calendar, ovulation, symptothermal, postovulation methods, declaration of abstinence for the duration of the trial, and withdrawal are not acceptable methods of contraception) . - and a local barrier form of contraception. Acceptable barrier methods are either the participant's use of a condom or the partner's use of an occlusive cap or diaphragm, or spermicides. Participants will not donate sperm from the selection visit and up to 90 days after the infusion. In case of sterile or vasectomised participants, no contraception will be required for their partners of childbearing potential.
11. Willing/able to adhere to the study visit schedule and other requirements, prohibitions and restrictions specified in this protocol.

Exclusion Criteria:

If any of the following exclusion criteria apply, the participant must not enter the study:

1. Currently have or have a history of any clinically significant medical illness or medical disorders the PI considers should exclude the participant, including (but not limited to) cardiovascular disease, neuromuscular, haematological disease, immune deficiency state, respiratory disease, hepatic or gastrointestinal disease, neurological or psychiatric disease, ophthalmological disorders, neoplastic disease, renal or urinary tract diseases, or dermatological disease.
2. History of personal or familial bleeding disorders; including prolonged or unusual bleeding.
3. History of deficiency in factor XII (FXII) or haemophilia type A (FVII) or type B (FIX) or type C (FXI).
4. History of cerebral bleeding (e.g. after a car accident), stroke and cerebrovascular accident (CVA).
5. Anamnestic history of Lyme disease or tick-borne encephalitis.
6. Use of Acetylsalicylic-Acid (ASA)-containing OTC medications within 1 month prior to screening.
7. Chronic administration of NSAIDs, chronic use of corticosteroids within 1 month prior to screening.
8. Chronic administration of clopidogrel, ticlopidin, dipyridamole, Coumadin-like anticoagulants, new oral anticoagulant dabigatran, rivaroxaban, apixaban or edoxaban within 3 months prior to screening.
9. Administration of unfractionated heparin, low molecular weight heparin, fibrinolytic agents and anti-FXa within 3 months prior to screening.
10. Have an active acute or chronic infection or diagnosed latent infection.
11. Systolic blood pressure (SBP) greater than 150 or less than 90 mmHg, diastolic blood pressure (DBP) greater than 90 or less than 50 mmHg, and heart rate (HR) greater than 100 or less than 40 bpm.
12. Acute clinically relevant illness within 7 days prior to study drug administration or have had a major illness or hospitalisation within 1 month prior to study drug administration.
13. Major or traumatic surgery within 12 weeks of screening.
14. Any participant who plans to undergo elective surgery within 4 weeks prior to study drug administration and through the discharge visit.
15. Positive serology test for HIV antibodies, hepatitis B surface antigen (HBsAg), or hepatitis C virus (HCV) antibodies at screening.
16. Recent history (within previous 6 months) of alcohol or drug abuse.
17. Have positive urine toxicology screen at screening or D -1 for substances of abuse including amphetamines, benzodiazepine, cocaine, opiates, barbiturate and cannabinoids.
18. Have a positive alcohol breath test at screening or D-1.
19. Consumes, on average, more than approximately 500 mg/day of caffeine (as contained in 5 cups of tea or coffee or 8 cans of caffeine-containing soda or other caffeinated products per day).
20. Donated blood (i.e. 500 mL) within 3 months before D1.
21. Have a history of active drug and/or food allergy or other active allergic disease requiring the constant use of medications, or a history of severe allergic reaction, angioedema or anaphylaxis.
22. Received any other experimental therapy or new investigational drug within 30 days or 5 half-lives (whichever is longer) of study drug administration.

Contacts and Locations

Information from the National Library of Medicine

To learn more about this study, you or your doctor may contact the study research staff using the contact information provided by the sponsor.

Please refer to this study by its ClinicalTrials.gov identifier (NCT number): ***NCT04653766***

Locations

Belgium
A.T.C. Pharma
Liège, Belgium, 4000

Sponsors and Collaborators
Bioxodes S.A.

Study Documents (Full-Text)

Documents provided by Bioxodes S.A.:

Study Protocol [PDF] October 15, 2020

Statistical Analysis Plan [PDF] July 9, 2020

More Information

Responsible Party:	Bioxodes S.A.
ClinicalTrials.gov Identifier:	NCT04653766 History of Changes
Other Study ID Numbers:	Clin_IrCPI_101<br>2019-002305-22 (EudraCT Number)
First Posted:	December 4, 2020 Key Record Dates
Results First Posted:	May 27, 2021
Last Update Posted:	February 23, 2023
Last Verified:	February 2023

Individual Participant Data (IPD) Sharing Statement:
Plan to Share IPD: No

Studies a U.S. FDA-regulated Drug Product: No
Studies a U.S. FDA-regulated Device Product: No

Keywords provided by Bioxodes S.A.:
Antithrombotic
Intrinsic coagulation pathway

This is the classic website, which will be retired eventually. Please visit the modernized ClinicalTrials.gov instead.

A Study to Evaluate the Safety, Tolerability, Pharmacokinetics, and Pharmacodynamics of Ir-CPI in Healthy Male Subjects

The safety and scientific validity of this study is the responsibility of the study sponsor and investigators. Listing a study does not mean it has been evaluated by the U.S. Federal Government. Read our disclaimer for details.

ClinicalTrials.gov Identifier: NCT04653766

Recruitment Status : Completed
First Posted : December 4, 2020
Results First Posted : May 27, 2021
Last Update Posted : February 23, 2023

View this study on the modernized ClinicalTrials.gov

Sponsor:
Bioxodes S.A.

Information provided by (Responsible Party):
Bioxodes S.A.

Study Details | Tabular View | Study Results | Disclaimer | How to Read a Study Record

**Study Type**	Interventional
**Study Design**	Allocation: Randomized; Intervention Model: Sequential Assignment; Masking: Double (Participant, Investigator); Primary Purpose: Other
**Condition**	Healthy
**Interventions**	Drug: Ir-CPI - Dose 1<br>Drug: Ir-CPI - Dose 2<br>Drug: Ir-CPI - Dose 3<br>Drug: Ir-CPI - Dose 4<br>Drug: Placebo
**Enrollment**	32

Participant Flow

Go to

Recruitment Details	
Pre-assignment Details	

Arm/Group Title	Ir-CPI - Dose 1	Ir-CPI - Dose 2	Ir-CPI - Dose 3	Ir-CPI - Dose 4	Placebo
Arm/Group Description	6 participants received a single intravenous dose of 1.5 mg/kg of Ir-CPI during 6 hours	6 participants received a single intravenous dose of 3.0 mg/kg of Ir-CPI during 6 hours	6 participants received a single intravenous dose of 6.0 mg/kg of Ir-CPI during 6 hours	6 participants received a single intravenous dose of 9.0 mg/kg of Ir-CPI during 6 hours	For each dose group, 2 additional participants received a single intravenous dose of placebo during 6 hours (8 in total).
Period Title: **Overall Study**					
Started	6	6	6	6	8
Completed	6	6	6	6	8
Not Completed	0	0	0	0	0

Baseline Characteristics

Go to

Arm/Group Title		Ir-CPI - Dose 1	Ir-CPI - Dose 2	Ir-CPI - Dose 3	Ir-CPI - Dose 4	Placebo	Total
Arm/Group Description		Ir-CPI - Dose 1: 6 participants received a single intravenous dose of 1.5 mg/kg of Ir-CPI during 6 hours	Ir-CPI - Dose 2: 6 participants received a single intravenous dose of 3.0 mg/kg of Ir-CPI during 6 hours	Ir-CPI - Dose 3: 6 participants received a single intravenous dose of 6.0 mg/kg of Ir-CPI during 6 hours	Ir-CPI - Dose 4: 6 participants received a single intravenous dose of 9.0 mg/kg of Ir-CPI during 6 hours	For each dose group, 2 additional participants received a single intravenous dose of placebo during 6 hours (8 in total).	Total of all reporting groups
Overall Number of Baseline Participants		6	6	6	6	8	32
Baseline Analysis Population Description		[Not Specified]					
Age, Categorical Measure Type: Count of Participants Unit of measure: Participants							
	Number Analyzed	6 participants	6 participants	6 participants	6 participants	8 participants	32 participants
	<=18 years	0 0.0%	0 0.0%	0 0.0%	0 0.0%	0 0.0%	0 0.0%
	Between 18 and 65 years	6 100.0%	6 100.0%	6 100.0%	6 100.0%	8 100.0%	32 100.0%
	>=65 years	0 0.0%	0 0.0%	0 0.0%	0 0.0%	0 0.0%	0 0.0%
Age, Continuous Mean (Standard Deviation) Unit of measure: Years							
	Number Analyzed	6 participants	6 participants	6 participants	6 participants	8 participants	32 participants
		36.0 (12.2)	35.2 (11.8)	27.3 (7.6)	34.8 (10.0)	35.8 (11.4)	33.9 (10.6)
Sex: Female, Male Measure Type: Count of Participants Unit of measure: Participants							
	Number Analyzed	6 participants	6 participants	6 participants	6 participants	8 participants	32 participants
	Female	0 0.0%	0 0.0%	0 0.0%	0 0.0%	0 0.0%	0 0.0%
	Male	6 100.0%	6 100.0%	6 100.0%	6 100.0%	8 100.0%	32 100.0%
Ethnicity (NIH/OMB) Measure Type: Count of Participants Unit of measure: Participants							
	Number Analyzed	6 participants	6 participants	6 participants	6 participants	8 participants	32 participants
	Hispanic or Latino	0 0.0%	0 0.0%	0 0.0%	0 0.0%	0 0.0%	0 0.0%
	Not Hispanic or Latino	6 100.0%	6 100.0%	6 100.0%	6 100.0%	8 100.0%	32 100.0%
	Unknown or Not Reported	0 0.0%	0 0.0%	0 0.0%	0 0.0%	0 0.0%	0 0.0%
Race (NIH/OMB) Measure Type: Count of Participants Unit of measure: Participants							
	Number Analyzed	6 participants	6 participants	6 participants	6 participants	8 participants	32 participants
	American Indian or Alaska Native	0 0.0%	0 0.0%	0 0.0%	0 0.0%	0 0.0%	0 0.0%
	Asian	0 0.0%	0 0.0%	0 0.0%	0 0.0%	0 0.0%	0 0.0%
	Native Hawaiian or Other Pacific Islander	0 0.0%	0 0.0%	0 0.0%	0 0.0%	0 0.0%	0 0.0%
	Black or African American	0 0.0%	0 0.0%	0 0.0%	0 0.0%	0 0.0%	0 0.0%
	White	6 100.0%	6 100.0%	6 100.0%	6 100.0%	8 100.0%	32 100.0%
	More than one race	0 0.0%	0 0.0%	0 0.0%	0 0.0%	0 0.0%	0 0.0%
	Unknown or Not Reported	0 0.0%	0 0.0%	0 0.0%	0 0.0%	0 0.0%	0 0.0%
Region of Enrollment Measure Type: Number Unit of measure: Participants							
Belgium	Number Analyzed	6 participants	6 participants	6 participants	6 participants	8 participants	32 participants
		6	6	6	6	8	32
Height Mean (Standard Deviation) Unit of measure: Cm							
	Number Analyzed	6 participants	6 participants	6 participants	6 participants	8 participants	32 participants
		178.8 (4.4)	175.3 (4.5)	177.2 (4.9)	174.8 (6.8)	179.5 (6.9)	177.3 (5.7)
Weight Mean (Standard Deviation) Unit of measure: Kg							
	Number Analyzed	6 participants	6 participants	6 participants	6 participants	8 participants	32 participants
		78.43 (4.28)	76.22 (9.24)	77.25 (6.34)	66.80 (7.93)	72.96 (6.99)	74.25 (7.84)
Body Mass Index (BMI) Mean (Standard Deviation) Unit of measure: Kg/m²							
	Number Analyzed	6 participants	6 participants	6 participants	6 participants	8 participants	32 participants
		24.52 (0.95)	24.75 (2.47)	24.65 (2.32)	21.81 (1.63)	22.68 (2.23)	23.62 (2.24)

Outcome Measures

Go to

1. Primary Outcome

Title	Measurement of the Safety Lab Parameter Activated Partial Thromboplastin Time (aPTT)
Description	These safety aPTT results were readily available to the PI for safety follow-up during and after the study drug administration. Safety aPTT was followed up "at the bedside" at regular time-points (as opposed to the PD biomarker aPTT, which was assessed by the central laboratory alongside the PK time-points). An aPTT ratio was calculated by dividing the aPTT value (in sec) of the specific time-point (aPTTt) by the baseline aPTT value (in sec) (aPTTbaseline) of the same participant (aPTT ratio = aPTTt/aPTTbaseline). The baseline time-point was considered as having an aPTT ratio of 1.<br>Concerning the row titles, for example, Day 1 H02:00 corresponds to aPTT ratio results obtained on Day 1, 2 hours after the start of the infusion.
Time Frame	At pre-dose (baseline), 2, 4, 6, 8, 12, 24, 48, 72 hours post-dose and at the discharge visit (Day 10 ± 2 days)

Outcome Measure Data

Analysis Population Description
[Not Specified]

Arm/Group Title	Ir-CPI - Dose 1	Ir-CPI - Dose 2	Ir-CPI - Dose 3	Ir-CPI - Dose 4	Placebo
Arm/Group Description:	Ir-CPI - Dose 1: 6 participants received a single intravenous dose of 1.5 mg/kg of Ir-CPI during 6 hours	Ir-CPI - Dose 2: 6 participants received a single intravenous dose of 3.0 mg/kg of Ir-CPI during 6 hours	Ir-CPI - Dose 3: 6 participants received a single intravenous dose of 6.0 mg/kg of Ir-CPI during 6 hours	Ir-CPI - Dose 4: 6 participants received a single intravenous dose of 9.0 mg/kg of Ir-CPI during 6 hours	Placebo: For each dose group, 2 additional participants received a single intravenous dose of placebo during 6 hours (8 in total).
Overall Number of Participants Analyzed	6	6	6	6	8
Mean (Standard Deviation) Unit of Measure: aPTT ratio					
Day 1 H00:00 (baseline)	1.000 (0.000)	1.000 (0.000)	1.000 (0.000)	1.000 (0.000)	1.000 (0.000)
Day 1 H02:00	1.600 (0.080)	1.978 (0.068)	2.405 (0.214)	2.627 (0.126)	0.986 (0.088)
Day 1 H04:00	1.768 (0.123)	2.262 (0.085)	2.628 (0.260)	2.875 (0.129)	0.981 (0.077)
Day 1 H06:00	1.860 (0.103)	2.313 (0.060)	2.775 (0.269)	2.993 (0.196)	0.994 (0.086)
Day 1 H08:00	1.520 (0.100)	1.832 (0.072)	2.135 (0.208)	2.367 (0.096)	0.960 (0.091)
Day 1 H12:00	1.308 (0.070)	1.600 (0.102)	1.848 (0.075)	2.077 (0.046)	0.970 (0.075)
Day 2 H24:00	1.162 (0.056)	1.352 (0.122)	1.627 (0.114)	1.725 (0.056)	0.973 (0.077)
Day 3 H48:00	1.033 (0.041)	1.123 (0.132)	1.313 (0.063)	1.377 (0.064)	0.983 (0.108)
Day 4 H72:00	1.020 (0.036)	1.083 (0.056)	1.143 (0.067)	1.182 (0.046)	0.974 (0.071)
Day 10 (discharge visit)	1.003 (0.029)	1.017 (0.072)	1.027 (0.046)	0.993 (0.043)	0.993 (0.093)

2. Secondary Outcome

Title	Measurement of the Maximum Plasma Concentration (Cmax) of Ir-CPI
Description	[Not Specified]
Time Frame	Pre-dose, 0.5, 1, 1.5, 2, 4, 6, 6.5, 7, 7.5, 8, 10, 12, 16, 24, 48, 72, 96, 144 hours (144 hours for Dose 4 only)

Outcome Measure Data

Analysis Population Description
Pharmacokinetic set (PKS): all of the included participants who were administered a complete infusion of study drug without major protocol deviation affecting PK evaluation.

Arm/Group Title	Ir-CPI - Dose 1	Ir-CPI - Dose 2	Ir-CPI - Dose 3	Ir-CPI - Dose 4
Arm/Group Description:	Ir-CPI - Dose 1: 6 participants received a single intravenous dose of 1.5 mg/kg of Ir-CPI during 6 hours	Ir-CPI - Dose 2: 6 participants received a single intravenous dose of 3.0 mg/kg of Ir-CPI during 6 hours	Ir-CPI - Dose 3: 6 participants received a single intravenous dose of 6.0 mg/kg of Ir-CPI during 6 hours	Ir-CPI - Dose 4: 6 participants received a single intravenous dose of 9.0 mg/kg of Ir-CPI during 6 hours
Overall Number of Participants Analyzed	6	6	6	6
Mean (Standard Deviation) Unit of Measure: ng/mL				
	958.8 (98.6)	2013.2 (228.2)	3752.5 (422.3)	6288.3 (757)

3. Secondary Outcome

Title	Measurement of the Time to Reach Maximum Plasma Concentration (Tmax) of Ir-CPI
Description	[Not Specified]
Time Frame	Pre-dose, 0.5, 1, 1.5, 2, 4, 6, 6.5, 7, 7.5, 8, 10, 12, 16, 24, 48, 72, 96, 144 hours (144 hours for Dose 4 only)

Outcome Measure Data

Analysis Population Description
Pharmacokinetic set (PKS): all of the included participants who were administered a complete infusion of study drug without major protocol deviation affecting PK evaluation.

Arm/Group Title	Ir-CPI - Dose 1	Ir-CPI - Dose 2	Ir-CPI - Dose 3	Ir-CPI - Dose 4
Arm/Group Description:	Ir-CPI - Dose 1: 6 participants received a single intravenous dose of 1.5 mg/kg of Ir-CPI during 6 hours	Ir-CPI - Dose 2: 6 participants received a single intravenous dose of 3.0 mg/kg of Ir-CPI during 6 hours	Ir-CPI - Dose 3: 6 participants received a single intravenous dose of 6.0 mg/kg of Ir-CPI during 6 hours	Ir-CPI - Dose 4: 6 participants received a single intravenous dose of 9.0 mg/kg of Ir-CPI during 6 hours
Overall Number of Participants Analyzed	6	6	6	6
Mean (Standard Deviation) Unit of Measure: hours				
	6 (0)	6 (0)	6 (0)	6 (0)

4. Secondary Outcome

Title	Measurement of the Area Under the Plasma Concentration-time Curve From Time Zero to 6h (AUC0-6) and From Time Zero to Time of Infinity (AUCinf)
Description	[Not Specified]
Time Frame	Pre-dose, 0.5, 1, 1.5, 2, 4, 6, 6.5, 7, 7.5, 8, 10, 12, 16, 24, 48, 72, 96, 144 hours (144 hours for Dose 4 only)

Outcome Measure Data

Analysis Population Description
Pharmacokinetic set (PKS): all of the included participants who were administered a complete infusion of study drug without major protocol deviation affecting PK evaluation.

Arm/Group Title	Ir-CPI - Dose 1	Ir-CPI - Dose 2	Ir-CPI - Dose 3	Ir-CPI - Dose 4
Arm/Group Description:	Ir-CPI - Dose 1: 6 participants received a single intravenous dose of 1.5 mg/kg of Ir-CPI during 6 hours	Ir-CPI - Dose 2: 6 participants received a single intravenous dose of 3.0 mg/kg of Ir-CPI during 6 hours	Ir-CPI - Dose 3: 6 participants received a single intravenous dose of 6.0 mg/kg of Ir-CPI during 6 hours	Ir-CPI - Dose 4: 6 participants received a single intravenous dose of 9.0 mg/kg of Ir-CPI during 6 hours
Overall Number of Participants Analyzed	6	6	6	6
Mean (Standard Deviation) Unit of Measure: h*ng/mL				
AUC0-6h	3648.0 (250.7)	7915.5 (1210.2)	15184.7 (1863.9)	24683.2 (5069.5)
AUC0-inf	13446.3 (1208.2)	26573.3 (2037.3)	53541.5 (3832.6)	104509.8 (10124.8)

5. Secondary Outcome

Title	Measurement of the Effect of Ir-CPI on the Activated Partial Thromboplastin Time (aPTT)
Description	The activated partial thromboplastin time (aPTT) is used as a pharmacodynamic marker. No data were available for the Ir-CPI 1.5 mg/kg group because the results were not usable due to plasma preparation and method repeatability problems. An aPTT ratio was calculated by dividing the aPTT value (in sec) of the specific time-point (aPTTt) by the baseline aPTT value (in sec) (aPTTbaseline) of the same participant (aPTT ratio = aPTTt/aPTTbaseline). The baseline time-point (Day 1 H00:00) was considered as having an aPTT ratio of 1.<br>Concerning the row titles, for example, Day 1 H02:00 corresponds to aPTT ratio results obtained on Day 1, 2 hours after the start of the infusion.
Time Frame	Pre-dose (baseline), 0.5, 1, 1.5, 2, 4, 6, 6.5, 7, 7.5, 8, 10, 12, 16, 24, 48, 72, 96 hours post-dose and at the discharge visit (Day 10 ± 2 days)

Outcome Measure Data

Analysis Population Description
This analysis was performed on the Pharmacodyamic Set (PDS): all of the included participants who completed the study without any protocol deviation affecting PD evaluation and with at least one available post-baseline PD data point.

Arm/Group Title	Ir-CPI - Dose 2	Ir-CPI - Dose 3	Ir-CPI - Dose 4	Placebo
Arm/Group Description:	Ir-CPI - Dose 2: 6 participants received a single intravenous dose of 3.0 mg/kg of Ir-CPI during 6 hours	Ir-CPI - Dose 3: 6 participants received a single intravenous dose of 6.0 mg/kg of Ir-CPI during 6 hours	Ir-CPI - Dose 4: 6 participants received a single intravenous dose of 9.0 mg/kg of Ir-CPI during 6 hours	Placebo: For each dose group, 2 additional participants received a single intravenous dose of placebo during 6 hours (8 in total).
Overall Number of Participants Analyzed	6	6	6	8
Mean (Standard Deviation) Unit of Measure: aPTT ratio				
Day 1 H00:00 (baseline)	1.000 (0.000)	1.000 (0.000)	1.000 (0.000)	1.000 (0.000)
Day 1 H00:30	1.535 (0.093)	1.825 (0.133)	2.218 (0.358)	0.982 (0.115)
Day 1 H01:00	1.707 (0.073)	2.085 (0.131)	2.397 (0.296)	0.962 (0.097)
Day 1 H01:30	1.827 (0.091)	2.225 (0.122)	2.533 (0.185)	0.982 (0.102)
Day 1 H02:00	1.915 (0.079)	2.367 (0.143)	2.692 (0.214)	0.972 (0.094)
Day 1 H04:00	2.148 (0.141)	2.583 (0.218)	2.822 (0.096)	0.970 (0.094)
Day 1 H06:00	2.207 (0.119)	2.652 (0.260)	3.050 (0.189)	0.973 (0.116)
Day 1 H06:30	2.045 (0.109)	2.442 (0.222)	2.798 (0.184)	0.982 (0.109)
Day 1 H07:00	1.917 (0.105)	2.240 (0.154)	2.558 (0.113)	0.972 (0.101)
Day 1 H07:30	1.842 (0.093)	2.103 (0.131)	2.402 (0.206)	0.950 (0.121)
Day 1 H08:00	1.775 (0.097)	2.098 (0.161)	1.098 (0.161)	0.945 (0.102)
Day 1 H10:00	1.638 (0.113)	1.942 (0.154)	2.230 (0.108)	0.922 (0.117)
Day 1 H12:00	1.540 (0.120)	1.842 (0.119)	2.137 (0.116)	0.952 (0.106)
Day 1 H16:00	1.428 (0.115)	1.705 (0.102)	1.947 (0.096)	0.987 (0.075)
Day 2 H24:00	1.312 (0.151)	1.585 (0.107)	1.718 (0.116)	0.965 (0.103)
Day 3 H48:00	1.125 (0.109)	1.277 (0.106)	1.390 (0.064)	0.945 (0.117)
Day 4 H72:00	1.025 (0.107)	1.113 (0.118)	1.172 (0.053)	0.988 (0.133)
Day 5 H96:00	0.990 (0.094)	1.037 (0.088)	1.062 (0.049)	0.942 (0.098)
Day 10 (discharge visit)	0.972 (0.078)	0.967 (0.072)	0.978 (0.067)	0.985 (0.128)

6. Secondary Outcome

Title	Measurement of the Effect of Ir-CPI on Factor XI Activity
Description	The method is a clotting assay where all the clotting factors are present (constant and in excess, brought by the deficient plasma), excepted for FXI, which is brought by the diluted tested plasma, and clotting is triggered with cephalin, activator and calcium (aPTT). FXI is the limiting factor and clotting time is inversely proportional to the concentration of FXI. There is an inverse linear relationship between the percentage of FXI activity and the corresponding clotting time. Central laboratory normal ranges for Factor XI activity are between 65 and 150 %.<br>Concerning the row titles, for example, Day 1 H02:00 corresponds to the percentage of FXI activity obtained on Day 1, 2 hours after the start of the infusion.
Time Frame	Pre-dose (baseline), 0.5, 1, 1.5, 2, 4, 6, 6.5, 7, 7.5, 8, 10, 12, 16, 24, 48, 72, 96 hours post-dose and at the discharge visit (Day 10 ± 2 days)

Outcome Measure Data

Analysis Population Description
This analysis was performed on the Pharmacodyamic Set (PDS): all of the included participants who completed the study without any protocol deviation affecting PD evaluation and with at least one available post-baseline PD data point.

Arm/Group Title	Ir-CPI - Dose 1	Ir-CPI - Dose 2	Ir-CPI - Dose 3	Ir-CPI - Dose 4	Placebo
Arm/Group Description:	Participants received a single intravenous dose of 1.5 mg/kg of Ir-CPI during 6 hours	Participants received a single intravenous dose of 3.0 mg/kg or Ir-CPI during 6 hours	Participants received a single intravenous dose of 6.0 mg/kg of Ir-CPI during 6 hours	Participants received a single intravenous dose of 9.0 mg/kg of Ir-CPI during 6 hours	Participants received a single intravenous dose of placebo during 6 hours
Overall Number of Participants Analyzed	6	6	6	6	8
Mean (Standard Deviation) Unit of Measure: Percentage of Factor XI activity					
Day 1 H00:00 (baseline)	112.3 (23.9)	103.7 (22.2)	89.2 (9.8)	94.5 (8.4)	95.9 (12.4)
Day 1 H00:30	98.7 (20.1)	85.5 (15.6)	61.8 (8.3)	58.8 (8.1)	99.5 (10.2)
Day 1 H01:00	94.8 (23.4)	79.5 (14.3)	53.3 (7.1)	54.7 (7.6)	104.8 (12.2)
Day 1 H01:30	98.0 (18.2)	73.3 (11.3)	49.8 (6.3)	50.0 (7.6)	99.3 (14.2)
Day 1 H02:00	91.5 (19.1)	70.8 (12.8)	46.2 (6.0)	47.2 (7.2)	100.1 (10.8)
Day 1 H04:00	88.8 (19.1)	64.0 (10.8)	42.5 (5.5)	41.7 (4.1)	106.6 (13.8)
Day 1 H06:00	85.5 (16.5)	61.7 (12.3)	41.3 (3.3)	40.5 (5.5)	106.6 (12.7)
Day 1 H06:30	88.7 (19.5)	67.7 (10.8)	49.5 (6.7)	45.3 (7.0)	104.9 (13.0)
Day 1 H07:00	91.3 (25.9)	70.7 (13.9)	49.2 (4.4)	47.0 (8.4)	101.6 (10.9)
Day 1 H07:30	91.7 (23.9)	74.5 (11.7)	51.0 (5.6)	47.8 (8.0)	100.5 (14.5)
Day 1 H08:00	92.8 (20.8)	73.8 (12.0)	54.8 (4.3)	50.7 (9.1)	106.4 (15.4)
Day 1 H10:00	99.3 (28.1)	83.2 (14.5)	61.0 (6.9)	56.7 (11.2)	106.4 (14.9)
Day 1 H12:00	102.7 (25.0)	87.0 (16.1)	61.5 (7.1)	59.5 (9.7)	101.0 (11.5)
Day 1 H16:00	101.8 (25.7)	89.0 (15.2)	65.3 (8.0)	63.5 (11.0)	98.4 (11.1)
Day 2 H24:00	116.0 (35.8)	92.7 (15.8)	73.2 (9.9)	71.2 (11.3)	104.8 (8.5)
Day 3 H48:00	117.8 (21.9)	104.7 (24.8)	85.8 (13.8)	81.5 (12.7)	104.0 (9.5)
Day 4 H72:00	136.8 (81.0)	104.5 (20.9)	92.8 (6.9)	86.7 (17.3)	105.5 (7.8)
Day 5 H96:00	110.8 (23.0)	105.5 (24.2)	90.0 (11.0)	97.2 (19.1)	107.4 (14.0)
Day 10 (discharge visit)	111.3 (28.6)	107.7 (17.9)	93.2 (9.3)	99.3 (16.8)	103.4 (11.7)

7. Secondary Outcome

Title	Measurement of the Effect of Ir-CPI on Factor XII Activity
Description	The method is a clotting assay where all the clotting factors are present (constant and in excess, brought by the deficient plasma), excepted for FXII, which is brought by the diluted tested plasma, and clotting is triggered with cephalin, activator and calcium (aPTT). FXII is the limiting factor and clotting time is inversely proportional to the concentration of FXII. There is an inverse linear relationship between the percentage of FXII activity and the corresponding clotting time. Central laboratory normal ranges for Factor XII activity are between 50 and 150 %.<br>Concerning the row titles, for example, Day 1 H02:00 corresponds to the percentage of FXII activity obtained on Day 1, 2 hours after the start of the infusion.
Time Frame	Pre-dose (baseline), 0.5, 1, 1.5, 2, 4, 6, 6.5, 7, 7.5, 8, 10, 12, 16, 24, 48, 72, 96 hours post-dose and at the discharge visit (Day 10 ± 2 days)

Outcome Measure Data

Analysis Population Description
This analysis was performed on the Pharmacodyamic Set (PDS): all of the included participants who completed the study without any protocol deviation affecting PD evaluation and with at least one available post-baseline PD data point.

Arm/Group Title	Ir-CPI - Dose 1	Ir-CPI - Dose 2	Ir-CPI - Dose 3	Ir-CPI - Dose 4	Placebo
Arm/Group Description:	Participants received a single intravenous dose of 1.5 mg/kg of Ir-CPI during 6 hours	Participants received a single intravenous dose of 3.0 mg/kg or Ir-CPI during 6 hours	Participants received a single intravenous dose of 6.0 mg/kg of Ir-CPI during 6 hours	Participants received a single intravenous dose of 9.0 mg/kg of Ir-CPI during 6 hours	Participants received a single intravenous dose of placebo during 6 hours
Overall Number of Participants Analyzed	6	6	6	6	8
Mean (Standard Deviation) Unit of Measure: Percentage of Factor XII activity					
Day 1 H00:00 (baseline)	105.0 (24.9)	94.8 (20.8)	101.5 (36.9)	100.0 (15.9)	111.0 (28.3)
Day 1 H00:30	93.0 (12.8)	68.8 (18.7)	60.8 (26.1)	42.3 (9.7)	114.0 (29.6)
Day 1 H01:00	83.5 (21.2)	62.2 (17.7)	45.8 (21.7)	35.0 (5.3)	119.1 (20.4)
Day 1 H01:30	82.7 (23.6)	53.3 (15.0)	43.8 (22.6)	29.3 (5.3)	111.4 (25.6)
Day 1 H02:00	76.0 (16.3)	49.3 (12.5)	32.7 (13.7)	26.3 (5.0)	113.5 (26.7)
Day 1 H04:00	68.5 (16.4)	40.5 (10.6)	27.5 (12.8)	24.8 (4.6)	117.0 (29.1)
Day 1 H06:00	64.8 (12.8)	38.2 (5.7)	26.7 (11.9)	24.2 (7.4)	116.4 (26.3)
Day 1 H06:30	69.5 (16.8)	45.8 (11.8)	33.3 (13.6)	25.8 (5.9)	116.3 (25.2)
Day 1 H07:00	75.0 (21.1)	47.0 (11.0)	35.8 (14.7)	29.8 (8.2)	116.8 (29.2)
Day 1 H07:30	75.2 (18.2)	53.3 (14.4)	39.0 (17.1)	31.2 (8.1)	115.9 (25.5)
Day 1 H08:00	77.7 (20.2)	54.7 (12.1)	44.3 (18.3)	34.0 (7.6)	117.4 (26.7)
Day 1 H10:00	90.3 (22.9)	65.5 (15.7)	54.2 (21.7)	40.5 (8.8)	118.3 (24.3)
Day 1 H12:00	91.3 (21.6)	71.3 (14.5)	58.5 (23.8)	42.3 (9.7)	113.6 (27.4)
Day 1 H16:00	89.7 (23.0)	73.8 (18.6)	61.0 (24.2)	49.8 (11.7)	112.8 (29.0)
Day 2 H24:00	104.8 (25.9)	80.3 (16.4)	78.8 (31.3)	59.3 (14.6)	116.5 (27.0)
Day 3 H48:00	128.7 (27.6)	96.7 (15.4)	92.0 (35.8)	79.7 (21.0)	118.1 (28.6)
Day 4 H72:00	121.5 (29.2)	98.3 (15.9)	112.0 (13.3)	89.5 (24.2)	120.4 (29.6)
Day 5 H96:00	123.5 (27.3)	100.5 (17.4)	102.5 (36.5)	100.2 (23.8)	121.6 (29.3)
Day 10 (discharge visit)	122.0 (26.9)	102.2 (20.2)	104.0 (35.3)	107.2 (23.8)	114.3 (27.5)

Adverse Events

Go to

Time Frame	From screening visit through last extra-ambulatory visit [Day 90 or Day 180 (in case of anti-drug antibodies at Day 90)]
Adverse Event Reporting Description	[Not Specified]

Arm/Group Title	Ir-CPI - Dose 1	Ir-CPI - Dose 2	Ir-CPI - Dose 3	Ir-CPI - Dose 4	Placebo
Arm/Group Description	6 participants received a single intravenous dose of 1.5 mg/kg of Ir-CPI during 6 hours	6 participants received a single intravenous dose of 3.0 mg/kg of Ir-CPI during 6 hours	6 participants received a single intravenous dose of 6.0 mg/kg of Ir-CPI during 6 hours	6 participants received a single intravenous dose of 9.0 mg/kg of Ir-CPI during 6 hours	For each dose group, 2 additional participants received a single intravenous dose of placebo during 6 hours (8 in total).

All-Cause Mortality

	Ir-CPI - Dose 1		Ir-CPI - Dose 2		Ir-CPI - Dose 3		Ir-CPI - Dose 4		Placebo	
	Affected / at Risk (%)		Affected / at Risk (%)		Affected / at Risk (%)		Affected / at Risk (%)		Affected / at Risk (%)	
Total	0/6 (0.00%)		0/6 (0.00%)		0/6 (0.00%)		0/6 (0.00%)		0/8 (0.00%)	

Serious Adverse Events

	Ir-CPI - Dose 1		Ir-CPI - Dose 2		Ir-CPI - Dose 3		Ir-CPI - Dose 4		Placebo	
	Affected / at Risk (%)	# Events	Affected / at Risk (%)	# Events	Affected / at Risk (%)	# Events	Affected / at Risk (%)	# Events	Affected / at Risk (%)	# Events
Total	1/6 (16.67%)		0/6 (0.00%)		0/6 (0.00%)		1/6 (16.67%)		0/8 (0.00%)	
Infections and infestations										
Appendicitis † 1	0/6 (0.00%)	0	0/6 (0.00%)	0	0/6 (0.00%)	0	1/6 (16.67%)	1	0/8 (0.00%)	0
Musculoskeletal and connective tissue disorders										
Polyarthritis acute * 2	1/6 (16.67%)	1	0/6 (0.00%)	0	0/6 (0.00%)	0	0/6 (0.00%)	0	0/8 (0.00%)	0

1 Term from vocabulary, MedDRA (22.0)
2 Term from vocabulary, MedDRA (24.1)
† Indicates events were collected by systematic assessment
* Indicates events were collected by non-systematic assessment

Other (Not Including Serious) Adverse Events

Frequency Threshold for Reporting Other Adverse Events	0%

	Ir-CPI - Dose 1		Ir-CPI - Dose 2		Ir-CPI - Dose 3		Ir-CPI - Dose 4		Placebo	
	Affected / at Risk (%)	# Events	Affected / at Risk (%)	# Events	Affected / at Risk (%)	# Events	Affected / at Risk (%)	# Events	Affected / at Risk (%)	# Events
Total	4/6 (66.67%)		5/6 (83.33%)		5/6 (83.33%)		6/6 (100.00%)		7/8 (87.50%)	
Gastrointestinal disorders										
Abdominal pain † 1	0/6 (0.00%)	0	0/6 (0.00%)	0	1/6 (16.67%)	1	1/6 (16.67%)	1	0/8 (0.00%)	0
Diarrhoea † 1	1/6 (16.67%)	1	1/6 (16.67%)	1	0/6 (0.00%)	0	0/6 (0.00%)	0	0/8 (0.00%)	0
Gastrointestinal pain † 1	0/6 (0.00%)	0	0/6 (0.00%)	0	0/6 (0.00%)	0	1/6 (16.67%)	1	0/8 (0.00%)	0
General disorders										
Catheter site haematoma † 1	0/6 (0.00%)	0	0/6 (0.00%)	0	0/6 (0.00%)	0	0/6 (0.00%)	0	1/8 (12.50%)	2
Fatigue † 1	0/6 (0.00%)	0	0/6 (0.00%)	0	1/6 (16.67%)	1	0/6 (0.00%)	0	0/8 (0.00%)	0
Vessel puncture site haematoma † 1	0/6 (0.00%)	0	1/6 (16.67%)	3	1/6 (16.67%)	2	3/6 (50.00%)	3	1/8 (12.50%)	2
Infections and infestations										
Acarodermatitis † 1	0/6 (0.00%)	0	0/6 (0.00%)	0	0/6 (0.00%)	0	0/6 (0.00%)	0	1/8 (12.50%)	1
Appendicitis † 1	0/6 (0.00%)	0	0/6 (0.00%)	0	0/6 (0.00%)	0	1/6 (16.67%)	1	0/8 (0.00%)	0
Diverticulitis † 1	0/6 (0.00%)	0	0/6 (0.00%)	0	1/6 (16.67%)	1	0/6 (0.00%)	0	0/8 (0.00%)	0
Fungal infection † 1	0/6 (0.00%)	0	1/6 (16.67%)	1	0/6 (0.00%)	0	0/6 (0.00%)	0	0/8 (0.00%)	0
Influenza † 1	0/6 (0.00%)	0	0/6 (0.00%)	0	1/6 (16.67%)	1	0/6 (0.00%)	0	0/8 (0.00%)	0
Laryngitis † 1	1/6 (16.67%)	1	0/6 (0.00%)	0	0/6 (0.00%)	0	0/6 (0.00%)	0	0/8 (0.00%)	0
Nasopharyngitis † 1	0/6 (0.00%)	0	0/6 (0.00%)	0	0/6 (0.00%)	0	2/6 (33.33%)	2	0/8 (0.00%)	0
Onychomycosis † 1	0/6 (0.00%)	0	0/6 (0.00%)	0	0/6 (0.00%)	0	1/6 (16.67%)	1	0/8 (0.00%)	0
Pharyngitis † 1	0/6 (0.00%)	0	1/6 (16.67%)	1	0/6 (0.00%)	0	0/6 (0.00%)	0	0/8 (0.00%)	0
Urinary tract infection † 1	0/6 (0.00%)	0	0/6 (0.00%)	0	1/6 (16.67%)	1	0/6 (0.00%)	0	0/8 (0.00%)	0
Injury, poisoning and procedural complications										
Arthropod bite † 1	0/6 (0.00%)	0	0/6 (0.00%)	0	0/6 (0.00%)	0	0/6 (0.00%)	0	1/8 (12.50%)	1
Contusion † 1	0/6 (0.00%)	0	0/6 (0.00%)	0	1/6 (16.67%)	1	0/6 (0.00%)	0	0/8 (0.00%)	0
Ligament injury † 1	1/6 (16.67%)	1	0/6 (0.00%)	0	0/6 (0.00%)	0	0/6 (0.00%)	0	0/8 (0.00%)	0
Investigations										
Fibrin D dimer increased † 1	0/6 (0.00%)	0	0/6 (0.00%)	0	0/6 (0.00%)	0	1/6 (16.67%)	2	1/8 (12.50%)	1
Musculoskeletal and connective tissue disorders										
Back pain † 1	0/6 (0.00%)	0	0/6 (0.00%)	0	1/6 (16.67%)	1	0/6 (0.00%)	0	0/8 (0.00%)	0
Bursitis † 1	0/6 (0.00%)	0	1/6 (16.67%)	1	0/6 (0.00%)	0	0/6 (0.00%)	0	0/8 (0.00%)	0
Myalgia † 1	0/6 (0.00%)	0	0/6 (0.00%)	0	0/6 (0.00%)	0	1/6 (16.67%)	1	1/8 (12.50%)	1
Nervous system disorders										
Headache † 1	0/6 (0.00%)	0	1/6 (16.67%)	1	3/6 (50.00%)	7	3/6 (50.00%)	6	1/8 (12.50%)	1
Respiratory, thoracic and mediastinal disorders										
Oropharyngeal pain † 1	1/6 (16.67%)	1	0/6 (0.00%)	0	1/6 (16.67%)	1	0/6 (0.00%)	0	0/8 (0.00%)	0
Rhinitis allergic † 1	0/6 (0.00%)	0	0/6 (0.00%)	0	0/6 (0.00%)	0	1/6 (16.67%)	1	2/8 (25.00%)	2
Surgical and medical procedures										
Sebaceous cyst excision † 1	0/6 (0.00%)	0	1/6 (16.67%)	1	0/6 (0.00%)	0	0/6 (0.00%)	0	0/8 (0.00%)	0
Varicose vein operation † 1	1/6 (16.67%)	1	0/6 (0.00%)	0	0/6 (0.00%)	0	0/6 (0.00%)	0	0/8 (0.00%)	0
Vascular disorders										
Haematoma † 1	0/6 (0.00%)	0	0/6 (0.00%)	0	1/6 (16.67%)	1	0/6 (0.00%)	0	0/8 (0.00%)	0

1 Term from vocabulary, MedDRA (22.0)
† Indicates events were collected by systematic assessment

Limitations and Caveats

Go to

[Not Specified]

More Information

Go to

Certain Agreements

Principal Investigators are NOT employed by the organization sponsoring the study.

There IS an agreement between Principal Investigators and the Sponsor (or its agents) that restricts the PI's rights to discuss or publish trial results after the trial is completed.

Results Point of Contact

Name/Title: Medical Writer
Organization: Bioxodes
Phone: +32 (0)71 239 602
EMail: stephanie.demoulin@bioxodes.com

Responsible Party: Bioxodes S.A.
ClinicalTrials.gov Identifier: NCT04653766 History of Changes
Other Study ID Numbers: Clin_IrCPI_101
2019-002305-22 (EudraCT Number)
First Submitted: October 29, 2020
First Posted: December 4, 2020
Results First Submitted: May 4, 2021
Results First Posted: May 27, 2021
Last Update Posted: February 23, 2023

(19) Europäisches Patentamt
European Patent Office
Office européen des brevets

(11) EP 3 325 516 B1

(12) EUROPEAN PATENT SPECIFICATION

(45) Date of publication and mention of the grant of the patent:
15.12.2021 Bulletin 2021/50

(21) Application number: **16745341.4**

(22) Date of filing: **21.07.2016**

(51) Int Cl.:
C07K 16/36 *(2006.01)*

(86) International application number:
PCT/US2016/043265

(87) International publication number:
WO 2017/015431 (26.01.2017 Gazette 2017/04)

(54) A MONOCLONAL ANTIBODY INHIBITOR OF FACTOR XIIA

MONOKLONALER INHIBITORISCHER ANTIKÖRPER GEGEN FAKTOR XIIA

ANTICORPS MONOCLONAL INHIBITEUR, DIRIGÉ CONTRE LE FACTEUR XIIA

(84) Designated Contracting States:
AL AT BE BG CH CY CZ DE DK EE ES FI FR GB GR HR HU IE IS IT LI LT LU LV MC MK MT NL NO PL PT RO RS SE SI SK SM TR

(30) Priority: **21.07.2015 US 201562194957 P**
31.12.2015 US 201562273657 P
31.03.2016 US 201662316310 P

(43) Date of publication of application:
30.05.2018 Bulletin 2018/22

(60) Divisional application:
21204197.4

(73) Proprietor: **Takeda Pharmaceutical Company Limited**
Osaka-shi, Osaka (JP)

(72) Inventors:
- **MASON, Shauna**
  Arlington, MA 02474 (US)
- **KENNISTON, Jon, A.**
  Hingham, MA 02043 (US)
- **NIXON, Andrew**
  Hanover, MA 02339 (US)
- **SEXTON, Daniel, J.**
  Melrose, MA 02176 (US)
- **COMEAU, Stephen, R.**
  Avon, New York 14414 (US)
- **ADELMAN, Burt**
  Concord, MA 01742 (US)

(74) Representative: **Hoffmann Eitle**
Patent- und Rechtsanwälte PartmbB
Arabellastraße 30
81925 München (DE)

(56) References cited:
WO-A1-90/08835
WO-A1-2013/014092
WO-A1-2016/109774

- **ESNOUF M PETER ET AL: "A monoclonal antibody raised against human beta-factor XIIa which also recognizes alpha-factor XIIa but not factor XII or complexes of factor XIIa with C1 esterase inhibitor", THROMBOSIS AND HAEMOSTASIS, SCHATTAUER GMBH, DE, vol. 83, no. 6, 1 June 2000 (2000-06-01), pages 874-881, XP009154609, ISSN: 0340-6245**
- **LARSSON MAGNUS ET AL: "A Factor XIIa Inhibitory Antibody Provides Thromboprotection in Extracorporeal Circulation Without Increasing Bleeding Risk", SCIENCE TRANSLATIONAL MEDICINE, vol. 6, no. 222, February 2014 (2014-02), XP002762081,**

Note: Within nine months of the publication of the mention of the grant of the European patent in the European Patent Bulletin, any person may give notice to the European Patent Office of opposition to that patent, in accordance with the Implementing Regulations. Notice of opposition shall not be deemed to have been filed until the opposition fee has been paid. (Art. 99(1) European Patent Convention).

Printed by Jouve, 75001 PARIS (FR)

Description

CROSS REFERENCE TO RELATED APPLICATIONS

[0001] This application claims the benefit of the filing date of U.S. Provisional Application No. 62/194957 filed July 21, 2015, U.S. Provisional Application No. 62/273657, filed December 31, 2015, and U.S. Provisional Application No. 62/316310, filed March 31, 2016.

BACKGROUND OF INVENTION

[0002] Factor XII (FXII) is a component of the plasma contact activation system responsible for both the initiation of intrinsic coagulation and the conversion of pre-kallikrein into plasma kallikrein (pKal). Active pKal cleaves high molecular weight kininogen (HMWK) to release bradykinin which is a potent activator of edema and pain. Activation of the intrinsic pathway for blood coagulation leads to the generation of fibrin and formation of blood clots (thrombus).

[0003] Esnouf et al; (2000), Thrombosis and Haemostasis , Vol 83, No 6, pages 874-881 disclosed the anti-Factor XIIa antibody mAb 2/215. WO2013/014092 disclosed the anti-Factor XIIa antibody 3F7.

SUMMARY OF INVENTION

[0004] The present disclosure is based on the identification of a number of antibodies that specifically bind Factor XIIa (FXIIa) but not Factor XII (FXII). These antibodies have high binding affinity to FXIIa (e.g., Ki^{app} < 50 pM); successfully reduced the activation of plasma kallikrein; and delayed activated partial thromboplastin time (APTT) but had no effect on prothrombin time (PT). The antibodies are further defined in the claims.

[0005] Accordingly, one aspect of the present disclosure features a monoclonal antibody that binds to Factor XIIa (FXIIa) and docs not bind to Factor XII (FXII). In some embodiments, the antibody is a full length antibody or an antigen-binding fragment thereof, such as an Fab. In some embodiments, the antibody is a human antibody or a humanized antibody.

[0006] In some embodiments, the antibody interacts with one or more amino acid residues in as further defined in the claims. chain C of FXIIa, as further defined in the claims. In some examples, the amino acid residues are selected from L390, Y391, W392, G393, H394, S395, F396, C397, H412, C413, L414, Q415, D416, R432, N433, V456, Y458, H507, F509, E510, G511, A512, E513, Y515, D557, A558, C559, Q560, G561, D562, S563, 1584, S585, W586, G587, S588, G589, C590, G591, D592, and G597 in SEQ ID NO: 128. In some embodiments, the antibody binds to a fragment of FXIIa comprising residues 390-397, 412-416, 432-433, 456-458, 507-515, 557-563 or 584-592 of SEQ ID NO: 128.

[0007] In some examples, the anti-FXIIa antibodies described herein comprises (a) a heavy chain comprising a heavy chain variable region that comprises a heavy chain complementarity determining region 1 (CDR1), a heavy chain complementarity determining region 2 (CDR2), and a heavy chain complementarity determining region 3 (CDR3), and optionally (b) a light chain comprising a light chain variable region that comprises a light chain CDR1, a light chain CDR2, and a light chain CDR3. This is further defined in the claims.

[0008] The heavy chain of the anti-FXIIa antibodies described herein comprises a heavy chain variable region that comprises an amino acid sequence of any of SEQ ID NO: 125.

[0009] Any of the heavy chains of the anti-FXIIa antibodies described herein may further comprise a heavy chain constant region. In some embodiments, the heavy chain constant region comprises at least one mutation (e.g., amino acid substitution) that enhances half-life of the antibody as compared to the wild-type counterpart. Such a mutated heavy chain constant region may comprise at least one mutation at a position corresponding to position 145, 147, or 149 of SEQ ID NO:119, which represents the amino acid sequence of an exemplary wild-type human heavy chain constant region. The at least one mutation may be an amino acid substitution of M145Y, S147T, and/or T149E. In some examples, the mutated heavy chain constant region comprises amino acid substitutions at positions 145, 147, and 149 of SEQ ID NO:119 *(e.g.,* M145Y, S147T, and T149E). Such a mutated heavy chain may comprise the amino acid sequence of SEQ ID NO:120.

[0010] The light chain of the anti-FXIIa antibodies described herein comprises a light chain variable region that comprises the amino acid sequence of SEQ ID NO:126.

[0011] Any of the anti-FXIIa antibodies described herein can be full length antibodies or antigen-binding fragments thereof. In some examples, the antibodies can be human antibodies or humanized antibodies.

[0012] Also within the scope of the present disclosure are an isolated nucleic acid or a set of nucleic acids, which collectively encode any of the anti-FXIIa antibodies (e.g., encoding at least the heavy and light chain variable regions of an antibody) described herein, a vector or vector set (e.g., expression vectors) that comprise the nucleic acid or the set of nucleic acids, and a host cell or host cell set that comprises the vector or vector set. Exemplary host cells include, but are not limited to, bacterial cells, yeast cells, insect cells, plant cells, and mammalian cells such as CHO cells.

[0013] Further, the present disclosure provides a method of producing any of the anti-FXIIa antibodies described herein. The method comprises culturing a host cell or host cell set that comprises a vector or vector set, which collectively encodes the antibody, and collecting the cultured cells or the culture medium for isolation of the antibody. Such a method can further comprise isolating the antibody from the cultured cells or the culture medium.

[0014] In another aspect, the present disclosure provides a method for treating a disease associated with Factor XII, namely diseases associated contact system activation, diseases associated with the pKal signaling pathway such as hereditary angioedema or HAE, or ocular diseases (e.g., macular edema, diabetic retinopathy, hypertensive retinopathy, age-related macular degeneration, or retinal vein occlusion) in a subjcct. Such methods may comprise administering to a subject in need thereof an effective amount of a composition comprising an antibody that binds to FXIIa and may not bind to FXII *(e.g.,* any of the anti-FXIIa antibodies described herein), one or more nucleic acids that, collectively encode the antibody, or expression vectors comprising such nucleic acid(s). In some embodiments, the antibody specifically binds the catalytic domain of FXIIa. In other embodiments, the antibody inhibits the activation of FXI to FXIa. In yet other embodiments, the Ki^{app} of the antibody is lower than about 110 pM *(e.g.,* lower than about 50 pM or 10 pM).

[0015] The subject to be treated by the method described herein may be a human subject having, suspected of having, or at risk for the pKal-associated disease (e.g., HAE). In some examples, the subject has, is suspected of having, or is at risk for Type I, Type II, or Type III HAE.

[0016] In some embodiments, the subject to be treated by the methods described herein may be a human subject having, suspected of having, or at risk for a disease associated with Factor XII such as those disclosed herein (namely a disease associated with contact system activation). For example, the subject may have, suspected of have, or at risk for a disease associated with contact system activation, such as thrombosis, including thrombosis associated with atrial fibrillation, deep vein thrombosis (DVT), pulmonary embolism, stroke, or an arterial or venous thrombotic event. In another example, the subject may have, suspected of having, or at risk for a disease associated with the pKal signaling pathway, e.g., HAE such as Type I, Type II, or Type III HAE. In yet another example, the subject may have, suspected of having, or at risk for an ocular disease, which can be macular edema, diabetic retinopathy, hypertensive retinopathy, age-related macular degeneration, or retinal vein occlusions.

[0017] Also within the scope of the present disclosure are pharmaceutical compositions comprising any of the anti-FXIIa antibodies described herein and a pharmaceutically acceptable carrier. Such pharmaceutical composition can be for use in treating a disease associated with the pKal signaling pathway (e.g., HAE such as Type I, Type II, or Type III HAE), or for use in manufacturing medicaments for use in treating such a disease.

[0018] The details of one or more embodiments of the invention are set forth in the description below. Other features or advantages of the present invention will be apparent from the following drawing and detailed description of several embodiments, and also from the appended claims.

BRIEF DESCRIPTION OF DRAWINGS

[0019] The following drawing forms part of the present specification and is included to further demonstrate certain aspects of the present disclosure, which can be better understood by reference to the drawing in combination with the detailed description of specific embodiments presented herein.

Figure 1 is a schematic illustration showing the coagulation cascade, in which Factor XII is involved.

Figure 2 is a schematic illustration showing the antibody selection strategy.

Figure 3 is a schematic illustration showing the bioassays used for characterizing activity of FXIIa inhibitors, including *in vitro* activity assay, plasma activity assay, and binding preference assay.

Figure 4 is a diagram showing the inhibitory activity of parent antibody 559C-M71-F06 (a human IgG1 antibody) against human (left panel) and mouse FXIIa (right panel).

Figure 5 is a diagram showing species cross-reactivity of M71-F06 as determined by APTT.

Figure 6 shows the concentration of the exemplary anti-FXIIa antibodies M191-E09 and M192-H11 in the plasma of rats at various time points following injection of the anti-FXIIa antibodies.

Figure 7 is a chart showing the time course of APTT and PT increase (sec) in the presence of anti-FXIIa antibody 559C-X211-A01 (A01). A: APTT kinetics after 10 mg/kg A01. B: time course of APTT in the presence of 10 mg/kg A01 over baesline. C: time course of PT (sec) in the presence of 10 mg/kg A01.

Figure 8 is a diagram showing APTT activity of antibody DX-4012 at the indicated time points prior to administration of the indicated treatment (Pre Drug, gray bars), 5 minutes prior to initiation of the study (Pre Study, black bars), and 5 minutes prior the end of the study (Post Study, white bars). The values indicate the average APTT +/- standard error in seconds. A: APTT time. B: fold increase in APTT over the baseline value (the average of the pre and post study measurement on the same animal on the control study day(s))

Figure 9 is a diagram showing PT activity of antibody DX-4012 at the indicated time points prior to administration of the indicated treatment (Pre Drug, gray bars), 5 minutes prior to initiation of the study (Pre Study, black bars),

and 5 minutes prior the end of the study (Post Study, white bars). The values indicate the average PT +/- standard error in seconds. A: PT time. B: fold increase in PT over the baseline value (the average of the pre and post study measurement on the same animal on the control study day(s)).

Figure 10 is a schematic of the external loop of a chronic arteriovenous (AV) shunt during thrombosis studies in non-human primates. The external loop is extended over the top of a gamma camera for real time measurement of platelet deposition.

Figure 11 is a chart showing the time course of platelet deposition on a collagen-coated graft. A: Thrombus head. B: Thrombus tail.

Figure 12 is a chart showing the time course of platelet deposition on a collagen-coated graft.

Figure 13 is a chart showing the time course of platelet deposition (total thrombus) on a collagen-coated graft.

Figure 14 is a chart showing the thrombus growth rate on a collagen-coated graft measured as platelet deposition per 5 minute interval.

Figure 15 is a chart showing the time course of platelet deposition on a tissue factor-coated graft. A: Thrombus head. B: Thrombus tail.

Figure 16 is a chart showing the time course of platelet deposition on a tissue factor-coated graft.

Figure 17 is a chart showing the time course of platelet deposition on a tissue factor-coated graft.

Figure 18 is a chart showing the thrombus growth rate on a tissue factor-coated graft measured as platelet deposition per 5 minute interval.

Figure 19 is a diagram showing terminal fibrin content and platelet deposition in the whole thrombus (head and tail) in collagen-coated grafts (gray bars) and tissue factor-coated grafts (white bars). A: Platelet deposition. B: Fibrin content.

Figure 20 is a chart showing the complex formation of the Fab fragment of DX-4012 and FXIIa by SEC analysis. A: FXIIa and Fab. B: FXIIa, Fab, and FXIIa-Fab complex of 50 μM FXIIa and 30 μM Fab. C: FXIIa, Fab, and FXIIa-Fab complex of 50 μM FXIIa and 40 μM Fab. D: FXIIa, Fab, and FXIIa-Fab complex of 50 μM FXIIa and 50 μM Fab. E: FXIIa, Fab, and FXIIa-Fab complex of 50 μM FXIIa and 60 μM Fab.

Figure 21 is a diagram showing the inhibitory activity of the anti-FXIIa antibody M192-H11 against human FXIIa.

Figure 22 is a chart showing hemostasis measurements associated with DX-4012. A: Bleeding time. B: Bleeding volume.

DETAILED DESCRIPTION OF INVENTION

[0020] Components of the contact system initiate the intrinsic pathway of coagulation and promote inflammation through the release of the proinflammatory peptide bradykinin. Factor XII (FXII), also known as Hageman Factor, is a serine protease that plays a role in activation of the intrinsic pathway of coagulation as well as the kallikrein-kinin system. FXII is activated by negatively charged surfaces (e.g., polyanionic surfaces, glass, polyphosphate, ellagic acid) to produce the active form FXIIa. Activated FXIIa has the ability to cleave pre-kallikrein, generating active pKal. Subsequently, activated pKal is able to cleave FXII into FXIIa, resulting in a positive feedback loop in which FXIIa generates even more pKal, which further activates additional FXII into FXIIa. Activated pKal is also able to cleave high molecular weight kininogen (HMWK) to release bradykinin. The coagulation cascade, in which FXII is involved, is illustrated in Figure 1.

[0021] In diseases associated with contact system activation, such as HAE, increased levels of bradykinin can induce vasodilation and inflammation that result in edematous HAE attacks. Accordingly, it is desired to develop novel therapeutics for treating a range of diseases that are potentially mediated by the activation of the contact system.

[0022] Described herein are antibodies that bind to and inhibit FXIIa, and uses thereof in inhibiting FXIIa and treating diseases associated with contact system activation. As shown in the Examples below, a number of exemplary anti-FXIIa antibodies were generated and shown to specifically bind to and inhibit the activity of FXIIa. Such antibodies as exemplified herein are expected to exhibit enhanced therapeutic effects in treating disease associated with contact system activation, particularly reducing the production of bradykinin, vasodilation, and pathological thrombi formation associated with disease symptoms.

Antibodies binding to FXIIa

[0023] The present disclosure provides antibodies that bind FXIIa, for example, the catalytic domain of FXIIa. Such antibodies may not bind to FXII.

[0024] An antibody (interchangeably used in plural form) is an immunoglobulin molecule capable of specific binding to a target, such as a carbohydrate, polynucleotide, lipid, polypeptide, etc., through at least one antigen recognition site, located in the variable region of the immunoglobulin molecule. As used herein, the term "antibody" encompasses not only intact (*i.e.*, full-length) polyclonal or monoclonal antibodies, but also antigen-binding fragments thereof (such as Fab, Fab', F(ab')2, Fv), single chain (scFv), mutants thereof, fusion proteins comprising an antibody portion, humanized

antibodies, chimeric antibodies, diabodies, nanobodies, linear antibodies, single chain antibodies, multispecific antibodies (e.g., bispecific antibodies) and any other modified configuration of the immunoglobulin molecule that comprises an antigen recognition site of the required specificity, including glycosylation variants of antibodies, amino acid sequence variants of antibodies, and covalently modified antibodies. An antibody includes an antibody of any class, such as IgD, IgE, IgG, IgA, or IgM (or sub-class thereof), and the antibody need not be of any particular class. Depending on the antibody amino acid sequence of the constant domain of its heavy chains, immunoglobulins can be assigned to different classes. There are five major classes of immunoglobulins: IgA, IgD, IgE, IgG, and IgM, and several of these may be further divided into subclasses (isotypes), e.g., IgG1, IgG2, IgG3, IgG4, IgA1 and IgA2. The heavy-chain constant domains that correspond to the different classes of immunoglobulins are called alpha, delta, epsilon, gamma, and mu, respectively. The subunit structures and three-dimensional configurations of different classes of immunoglobulins are well known.

[0025] In some embodiments, the anti-FXIIa antibody as described herein can bind and inhibit the activity of FXIIa by at least 50% *(e.g.,* 60%, 70%, 80%, 90%, 95% or greater). The apparent inhibition constant (Ki^{app} or $K_{i,app}$), which provides a measure of inhibitor potency, is related to the concentration of inhibitor required to reduce enzyme activity and is not dependent on enzyme concentrations. The inhibitory activity of an anti-FXIIa antibody described herein can be determined by routine methods known in the art.

[0026] The $K_{i,}^{app}$ value of an antibody may be determined by measuring the inhibitory effect of different concentrations of the antibody on the extent of the reaction (e.g., enzyme activity); fitting the change in pseudo-first order rate constant (v) as a function of inhibitor concentration to the modified Morrison equation (Equation 1) yields an estimate of the apparent Ki value. For a competitive inhibitor, the Ki^{app} can be obtained from the y-intercept extracted from a linear regression analysis of a plot of $K_{i,}^{app}$ versus substrate concentration.

$$v = A \cdot \frac{([E]-[I]-K_i^{app}) + \sqrt{([E]-[I]-K_i^{app})^2 + 4[E]\cdot K_i^{app}}}{2} \qquad \text{(Equation 1)}$$

[0027] Where A is equivalent to v_o/E, the initial velocity (v_o) of the enzymatic reaction in the absence of inhibitor (*I*) divided by the total enzyme concentration (E).

[0028] In some embodiments, the anti-FXIIa antibody described herein may have a Ki^{app} value of 1000, 900, 800, 700, 600, 500, 400, 300, 200, 100, 50, 40, 30, 20, 19, 18, 17, 16, 15, 14, 13, 12, 11, 10, 9, 8, 7, 6, 5 pM or less for the target antigen or antigen epitope. In some embodiments, the anti-FXIIa antibody may have a lower Ki^{app} for a first target *(e.g.,* FXIIa) relative to a second target *(e.g.,* FXII). Differences in Ki^{app} (e.g., for specificity or other comparisons) can be at least 1.5, 2, 3, 4, 5, 10, 15, 20, 37.5, 50, 70, 80, 91, 100, 500, 1000, 10,000 or 10^5 fold. In some examples, the anti-FXIIa antibody inhibits a first antigen (e.g., a first protein in a first conformation or mimic thereof) better relative to a second antigen (e.g., the same first protein in a second conformation or mimic thereof; or a second protein). In some embodiments, any of the anti-FXIIa antibodies may be further affinity matured to reduce the Ki^{app} of the antibody to the target antigen or antigenic epitope thereof.

[0029] The antibodies described herein can be murine, rat, human, or any other origin (including chimeric or humanized antibodies). Such antibodies are non-naturally occurring, *i.e.*, would not be produced in an animal without human act (e.g., immunizing such an animal with a desired antigen or fragment thereof).

[0030] Any of the antibodies described herein can be either monoclonal or polyclonal. A "monoclonal antibody" refers to a homogenous antibody population and a "polyclonal antibody" refers to a heterogeneous antibody population. These two terms do not limit the source of an antibody or the manner in which it is made.

[0031] In one example, the antibody used in the methods described herein is a humanized antibody. Humanized antibodies refer to forms of non-human (e.g. murine) antibodies that are specific chimeric immunoglobulins, immunoglobulin chains, or antigen-binding fragments thereof that contain minimal sequence derived from non-human immunoglobulin. For the most part, humanized antibodies are human immunoglobulins (recipient antibody) in which residues from a complementary determining region (CDR) of the recipient are replaced by residues from a CDR of a non-human species (donor antibody) such as mouse, rat, or rabbit having the desired specificity, affinity, and capacity. In some instances, Fv framework region (FR) residues of the human immunoglobulin are replaced by corresponding non-human residues. Furthermore, the humanized antibody may comprise residues that are found neither in the recipient antibody nor in the imported CDR or framework sequences, but are included to further refine and optimize antibody performance. In general, the humanized antibody will comprise substantially all of at least one, and typically two, variable domains, in which all or substantially all of the CDR regions correspond to those of a non-human immunoglobulin and all or substantially all of the FR regions are those of a human immunoglobulin consensus sequence. The humanized antibody optimally also will comprise at least a portion of an immunoglobulin constant region or domain (Fc), typically that of a human immunoglobulin. Antibodies may have Fc regions modified as described in WO 99/58572. Other forms of hu-

manized antibodies have one or more CDRs (one, two, three, four, five, six) which are altered with respect to the original antibody, which are also termed one or more CDRs "derived from" one or more CDRs from the original antibody. Humanized antibodies may also involve affinity maturation.

[0032] In another example, the antibody described herein is a chimeric antibody, which can include a heavy constant region and a light constant region from a human antibody. Chimeric antibodies refer to antibodies having a variable region or part of variable region from a first species and a constant region from a second species. Typically, in these chimeric antibodies, the variable region of both light and heavy chains mimics the variable regions of antibodies derived from one species of mammals (e.g., a non-human mammal such as mouse, rabbit, and rat), while the constant portions are homologous to the sequences in antibodies derived from another mammal such as human. In some embodiments, amino acid modifications can be made in the constant region.

[0033] In some embodiments, the anti-FXIIa antibodies described herein specifically bind to the corresponding target antigen or an epitope thereof. An antibody that "specifically binds" to an antigen or an epitope is a term well understood in the art. A molecule is said to exhibit "specific binding" if it reacts more frequently, more rapidly, with greater duration and/or with greater affinity with a particular target antigen than it does with alternative targets. An antibody "specifically binds" to a target antigen or epitope if it binds with greater affinity, avidity, more readily, and/or with greater duration than it binds to other substances. For example, an antibody that specifically (or preferentially) binds to an antigen (FXIIa) or an antigenic epitope therein is an antibody that binds this target antigen with greater affinity, avidity, more readily, and/or with greater duration than it binds to other antigens or other epitopes in the same antigen. It is also understood with this definition that, for example, an antibody that specifically binds to a first target antigen may or may not specifically or preferentially bind to a second target antigen. As such, "specific binding" or "preferential binding" does not necessarily require (although it can include) exclusive binding. In some examples, an antibody that "specifically binds" to a target antigen or an epitope thereof may not bind to other antigens or other epitopes in the same antigen. The antibodies described herein specifically bind to FXIIa. The antibodies described herein specifically bind to FXIIa and do not bind to FXII.

[0034] As used herein, the term "Factor XIIa" or "FXIIa" refers to the active form of Factor XII and the term "Factor XII" or "FXII" refers to the proenzyme or zymogen of Factor XII (inactive form). FXII is a single chain glycoprotein with a molecule weight of about 80 kD. Once activated, FXII is concerted into the active form, FXIIa, which contains two chains, the heavy chain (353 residues of the human FXIIa heavy chain) and the light chain (243 residues of the human FXIIa light chain), held by a disulfide bond. Human FXII is encoded by gene *F12.* The amino acid sequence of human FXII is well known in the art, *e.g.,* GenBank Accession Number NP_000496.2.

[0035] In some embodiments, an anti-FXIIa antibody as described herein has a suitable binding affinity for the target antigen (e.g., FXIIa) or antigenic epitopes thereof. As used herein, "binding affinity" refers to the apparent association constant or K_A. The K_A is the reciprocal of the dissociation constant (K_D). The anti-FXIIa antibody described herein may have a binding affinity (K_D) of at least 10^{-5}, 10^{-6}, 10^{-7}, 10^{-8}, 10^{-9}, 10^{-10} M, or lower for the target antigen or antigenic epitope. An increased binding affinity corresponds to a decreased K_D. Higher affinity binding of an antibody for a first antigen relative to a second antigen can be indicated by a higher K_A (or a smaller numerical value K_D) for binding the first antigen than the K_A (or numerical value K_D) for binding the second antigen. In such cases, the antibody has specificity for the first antigen (e.g., a first protein in a first conformation or mimic thereof) relative to the second antigen (e.g., the same first protein in a second conformation or mimic thereof; or a second protein). In some embodiments, the anti-FXIIa antibodies described herein have a higher binding affinity (a higher K_A or smaller K_D) to FXIIa as compared to the binding affinity to the zymogen FXII or another protein in the pKal signaling system. Differences in binding affinity (e.g., for specificity or other comparisons) can be at least 1.5, 2, 3, 4, 5, 10, 15, 20, 37.5, 50, 70, 80, 91, 100, 500, 1000, 10,000 or 10^5 fold. In some embodiments, any of the anti-FXIIa antibodies may be further affinity matured to increase the binding affinity of the antibody to the target antigen or antigenic epitope thereof.

[0036] Binding affinity (or binding specificity) can be determined by a variety of methods including equilibrium dialysis, equilibrium binding, gel filtration, ELISA, surface plasmon resonance, or spectroscopy (e.g., using a fluorescence assay). Exemplary conditions for evaluating binding affinity are in HBS-P buffer (10 mM HEPES pH7.4, 150 mM NaCl, 0.005% (v/v) Surfactant P20). These techniques can be used to measure the concentration of bound binding protein as a function of target protein concentration. The concentration of bound binding protein ([Bound]) is generally related to the concentration of free target protein ([Free]) by the following equation:

$$[\mathrm{Bound}] = [\mathrm{Free}]/(\mathrm{Kd}+[\mathrm{Free}])$$

[0037] It is not always necessary to make an exact determination of K_A, though, since sometimes it is sufficient to obtain a quantitative measurement of affinity, e.g., determined using a method such as ELISA or FACS analysis, is proportional to K_A, and thus can be used for comparisons, such as determining whether a higher affinity is, e.g., 2-fold higher, to obtain a qualitative measurement of affinity, or to obtain an inference of affinity, e.g., by activity in a functional

assay, e.g., an in vitro or in vivo assay.

[0038] The anti-FXIIa antibody comprises a heavy chain comprising a heavy chain variable region that comprises a heavy chain CDR1, a heavy chain CDR2, and a heavy chain CDR3. In some instances, the heavy chain CDR3 region comprises the amino acid sequence of SEQ ID NO: 111, 112 or 113 (see Table 1). In a reference embodiment, the heavy chain variable region of any of the anti-FXIIa antibodies described herein may further comprise a CDR1 region comprising the formula $X_1YX_3MX_5$ (SEQ ID NO: 117) as described herein, and/or a CDR2 region comprising the formula $X_1IX_3PSGX_7X_8TX_{10}YX_{12}DSVKG$, (SEQ ID NO: 118), which is also described herein. In some examples, the anti-FXIIa antibody comprises a heavy chain CDR1 region selected from SEQ ID NO: 41-73 and 121 (see Table 1) and/or a CDR2 region selected from SEQ ID NO: 74-110 and 122-124. In some specific examples, the heavy chain variable region of an anti-FXIIa antibody as described herein comprises the amino acid sequence of any of SEQ ID NOs:1-39.

[0039] Table 1 provides the amino acid sequences of the heavy chain CDRs for exemplary anti-FXIIa antibodies. Antibodies having the same heavy chain CDR1, CDR2, and CDR3 regions as those exemplary anti-FXIIa antibodies are also within the scope of the present disclosure.

Table 1: Heavy chain CDR sequences of anti-FXIIa antibodies (for reference purposes)

Exemplary Antibody	CDR1	CDR2	CDR3
M0191-E09	WYSMH (SEQ ID NO: 41)	VIYPSGGKTRYADSVKG (SEQ ID NO: 74)	QRYRGPKYYYYMDV (SEQ ID NO: 111)
M0192-H11	QYVMH (SEQ ID NO: 42)	SIWPSGGHTRYADSVKG (SEQ ID NO: 75)	QRYRGPKYYYYMDV (SEQ ID NO: 111)
M0184-F12	WYVMH (SEQ ID NO: 43)	GIWPSGGRTKYADSVKG (SEQ ID NO: 76)	QRYRGPKYYYYMDV (SEQ ID NO: 111)
M0292-D07	NYVMH (SEQ ID NO: 44)	SIWPSGGKTKYADSVKG (SEQ ID NO: 77)	QRYRGPKYYYYMDA (SEQ ID NO: 112)
M0182-D04	MYTMN (SEQ ID NO: 45)	RIYPSGGKTLYADSVKG (SEQ ID NO: 78)	QRYRGPKYYYYMDV (SEQ ID NO: 111)
M0192-F07	QYVMS (SEQ ID NO: 46)	RIYPSGGVTKYADSVKG (SEQ ID NO: 79)	QRYRGPKYYYYMDV (SEQ ID NO: 111)
M0183-C03	WYNMH (SEQ ID NO: 47)	YISPSGGKTKYTDSVKG (SEQ ID NO: 80)	QRYRGPKYYYYMDV (SEQ ID NO: 111)
M0183-H08	RYIMH (SEQ ID NO: 48)	SIYPSGGVTKYADSVKG (SEQ ID NO: 81)	QRYRGPKYYYYMDV (SEQ ID NO: 111)
M0183-D08	RYIMG (SEQ ID NO: 49)	SIYPSGGVTRYADSVKG (SEQ ID NO: 82)	QRYRGPKYYYYMDV (SEQ ID NO: 111)
M0192-G03	QYNMV (SEQ ID NO: 50)	RIWPSGGKTTYADSVKG (SEQ ID NO: 83)	QRYRGPKYYYYMDV (SEQ ID NO: 111)
M0310-C06	RYVMV (SEQ ID NO: 51)	RIYPSGGMTQYADSVKG (SEQ ID NO: 84)	QRYRGPKYYYYMDV (SEQ ID NO: 111)
M0192-F01	WYNMA (SEQ ID NO: 52)	RIYPSGGMTQYADSVKG (SEQ ID NO: 84)	QRYRGPKYYYYMDV (SEQ ID NO: 111)
M0191-A03	QYIMH (SEQ ID NO: 53)	SIYPSGGNTKYADSVKG (SEQ ID NO: 85)	QRYRGPKYYYYMDV (SEQ ID NO: 111)
M0192-A01	HYVMH (SEQ ID NO: 54)	SIYPSGGLTKYADSVKG (SEQ ID NO: 86)	QRYRGPKYYYYMDV (SEQ ID NO: 111)
M0191-H10	WYTMH (SEQ ID NO: 55)	SIYPSGGFTRYADSVKG (SEQ ID NO: 87)	QRYRGPKYYYYMDV (SEQ ID NO: 111)
M0177-C12	FYHMH (SEQ ID NO: 56)	RIVPSGGMTRYADSVKG (SEQ ID NO: 88)	QRYRGPKYYYYMDV (SEQ ID NO: 111)

(continued)

Exemplary Antibody	CDR1	CDR2	CDR3
M0184-B04	FYSMH (SEQ ID NO: 57)	RIYPSGGVTKYADSVKG (SEQ ID NO: 89)	QRYRGPKYYYYMDV (SEQ ID NO: 111)
M0308-H03	QYVMH (SEQ ID NO: 58)	SIWPSGGKTTYADSVKG (SEQ ID NO: 90)	QRYRGPKYYYYMDV (SEQ ID NO: 111)
M0192-D02	QYVMH (SEQ ID NO: 58)	SIWPSGGFTKYADSVKG (SEQ ID NO: 91)	QRYRGPKYYYYMDV (SEQ ID NO: 111)
M0310-G07	FYNMH (SEQ ID NO: 59)	SIYPSGGVTRYADSVKG (SEQ ID NO: 92)	QRYRGPKYYYYMDV (SEQ ID NO: 111)
M0192-F06	WYVMH (SEQ ID NO: 43)	SIYPSGGKTSYADSVKG (SEQ ID NO: 93)	QRYRGPKYYYYMDV (SEQ ID NO: 111)
M0310-A04	PYIMH (SEQ ID NO: 60)	VIYPSGSKTNYADSVKG (SEQ ID NO: 94)	QRYRGPKYYYYMDV (SEQ ID NO: 111)
M0310-F02	RYTMR (SEQ ID NO: 61)	SIWPSGGMTRYADSVKG (SEQ ID NO: 95)	QRYRGPKYYYYMDV (SEQ ID NO: 111)
M0310-B09	QYVMH (SEQ ID NO: 58)	SIYPSGGLTRYADSVKG (SEQ ID NO: 96)	QRYRGPKYYYYMDV (SEQ ID NO: 111)
M0310-G06	WYIMG (SEQ ID NO: 62)	YIYPSGGNTRYADSVKG (SEQ ID NO: 97)	QRYRGPKYYYYMDV (SEQ ID NO: 111)
M0182-H01	RYVMH (SEQ ID NO: 63)	SIWPSGGMTKYADSVKG (SEQ ID NO: 98)	QRYRGPKYYYYMDV (SEQ ID NO: 111)
M0178-A08	FYIMG (SEQ ID NO: 64)	RIYPSGGATQYADSVKG (SEQ ID NO: 99)	QRYRGPKYYYYMDV (SEQ ID NO: 111)
M0184-D01	FYVMG (SEQ ID NO: 65)	RIYPSGGLTQYADSVKG (SEQ ID NO: 100)	QRYRGPKYYYYMDV (SEQ ID NO: 111)
M0177-A06	FYSMH (SEQ ID NO: 66)	RIYPSGGITSYADSVKG (SEQ ID NO: 101)	QRYRGPKYYYYMDV (SEQ ID NO: 111)
M0191-E04	MYIMH (SEQ ID NO: 67)	SIYPSGGMTKYADSVKG (SEQ ID NO: 102)	QRYRGPKYYYYMDV (SEQ ID NO: 111)
M0191-H09	MYVMH (SEQ ID NO: 68)	SIYPSGGLTKYADSVKG (SEQ ID NO: 103)	QRYRGPKYYYYMDV (SEQ ID NO: 111)
M0192-H04	QYVMH (SEQ ID NO: 58)	RIYPSGGLTNYADSVKG (SEQ ID NO: 104)	QRYRGPKYYYYMDV (SEQ ID NO: 111)
M0192-A03	WYVMQ (SEQ ID NO: 69)	SIYPSGGMTKYADSVKG (SEQ ID NO: 102)	QRYRGPKYYYYMDV (SEQ ID NO: 111)
M0310-G08	WYIMG (SEQ ID NO: 62)	RIYPSGGSTHYADSVKG (SEQ ID NO: 105)	QRYRGPRYYYYIDA (SEQ ID NO: 113)
M0192-G05	QYTMV (SEQ ID NO: 70)	RIYPSGGVTQYADSVKG (SEQ ID NO: 106)	QRYRGPKYYYYMDV (SEQ ID NO: 111)
M0183-B12	WYVMY (SEQ ID NO: 71)	RIYPSGGITHYADSVKG (SEQ ID NO: 107)	QRYRGPKYYYYMDV (SEQ ID NO: 111)
M0308-F04	FYVML (SEQ ID NO: 72)	SIWPSGGVTKYADSVKG (SEQ ID NO: 108)	QRYRGPKYYYYMDV (SEQ ID NO: 111)
M0182-B04	WYVMQ (SEQ ID NO: 69)	YIYPSGGHTKYADSVKG (SEQ ID NO: 109)	QRYRGPKYYYYMDV (SEQ ID NO: 111)

(continued)

Exemplary Antibody	CDR1	CDR2	CDR3
M0310-F04	RYSMN (SEQ ID NO: 73)	GIYPSGGKTKYADSVKG (SEQ ID NO: 110)	QRYRGPKYYYYMDV (SEQ ID NO: 111)
X211-A01 (DX-4012)	QYVMH (SEQ ID NO:58)	SIWPSGGHTRYADSVKG (SEQ ID NO:75)	QRYRGPKYYYYMDV (SEQ ID NO:111)

[0040] The heavy chain variable regions of the exemplary anti-FXIIa antibodies listed in Table 1 are provided below (CDR regions are boldfaced). In other aspects, the HC CDR1 of the anti-FXIIa antibody can be QYSMH (SEQ ID NO:121), which may be in combination with HC CDR2 of SEQ ID NO:74 and/or HC CDR3 of SEQ ID NO:111. In yet other embodiments, the HC CDR2 of the anti-FXIIa antibody can be SIYPSGGKTRYADSVKG (SEQ ID NO:122), VIWPSG-GKTRYADSVKG (SEQ ID NO:123), or VIYPSGGHTRYADSVKG (SEQ ID NO:124), which may be in combination with HC CDR1 of SEQ ID NO:41 and/or HC CDR3 of SEQ ID NO:111. In still another embodiment, the HC CDR2 of the anti-FXIIa antibody can be SIWPSGGHTRYADSVHG (SEQ ID NO:127), which may be in combination with HC CDR1 of SEQ ID NO:58 and/or HC CDR3 of SEQ ID NO:111.

Heavy chain variable region sequence of M0191-E09 (SEQ ID NO: 1):

EVQLLESGGGLVQPGGSLRLSCAASGFTFS**WYSMH**WVRQAPGKGLEWVS**VIYPSGGKTRYADSVKG**RFTISRD NSKNTLYLQMNSLRAEDTAVYYCTR**QRYRGPKYYYYMDV**WGKGTTVTVSS

Heavy chain variable region sequence of M0192-H11 (SEQ ID NO: 2):

EVQLLESGGGLVQPGGSLRLSCAASGFTFS**QYVMH**WVRQAPGKGLEWVS**SIWPSGGHTRYADSVKG**RFTISRD NSKNTLYLQMNSLRAEDTAVYYCTR**QRYRGPKYYYYMDV**WGKGTTVTVSS

Heavy chain variable region sequence of M0184-F12 (SEQ ID NO: 3):

EVQLLESGGGLVQPGGSLRLSCAASGFTFS**WYVMH**WVRQAPGKGLEWVS**GIWPSGGRTKYADSVKG**RFTISRD NSKNTLYLQMNSLRAEDTAVYYCTR**QRYRGPKYYYYMDV**WGKGTTVTVSS

Heavy chain variable region sequence of M0292-D07 (SEQ ID NO: 4):

EVQLLESGGGLVQPGGSLRLSCAASGFTFS**NYVMH**WVRQAPGKGLEWVS**SIWPSGGKTKYADSVKG**RFTISRD NSKNTLYLQMNSLRAEDTAVYYCTR**QRYRGPKYYYYMDA**WGQGTTVTVSS

Heavy chain variable region sequence of M0182-D04(SEQ ID NO: 5):

EVQLLESGGGLVQPGGSLRLSCAASGFTFS**MYTMN**WVRQAPGKGLEWVS**RIYPSGGKTLYADSVKG**RFTISRD NSKNTLYLQMNSLRAEDTAVYYCTR**QRYRGPKYYYYMDV**WGKGTTVTVSS

Heavy chain variable region sequence of M192-F07 (SEQ ID NO: 6):

EVQLLESGGGLVQPGGSLRLSCAASGFTFS**QYVMS**WVRQAPGKGLEWVS**RIYPSGGVTKYADSVKG**RFTISRD NSKNTLYLQMNSLRAEDTAVYYCTR**QRYRGPKYYYYMDV**WGKGTTVTVSS

Heavy chain variable region sequence of M0183-C03 (SEQ ID NO: 7):

EVQLLESGGGLVQPGGSLRLSCAASGFTFS**WYNMH**WVRQAPGKGLEWVS**YISPSGGKTKYTDSVKG**RFTISRD
NSKNTLYLQMNSLRAEDTAVYYCTR**QRYRGPKYYYYMDV**WGKGTTVTVSS

Heavy chain variable region sequence of M0183-H08 (SEQ ID NO: 8):

EVQLLESGGGLVQPGGSLRLSCAASGFTFS**RYIMH**WVRQAPGKGLEWVS**SIYPSGGVTKYADSVKG**RFTISRD
NSKNTLYLQMNSLRAEDTAVYYCTR**QRYRGPKYYYYMDV**WGKGTTVTVSS

Heavy chain variable region sequence of M0183-D08 (SEQ ID NO: 9):

EVQLLESGGGLVQPGGSLRLSCAASGFTFS**RYIMG**WVRQAPGKGLEWVS**SIYPSGGVTRYADSVKG**RFTISRD
NSKNTLYLQMNSLRAEDTAVYYCTR**QRYRGPKYYYYMDV**WGKGTTVTVSS

Heavy chain variable region sequence of M0192-G03 (SEQ ID NO: 10):

EVQLLESGGGLVQPGGSLRLSCAASGFTFS**QYNMV**WVRQAPGKGLEWVS**RIWPSGGKTTYADSVKG**RFTISRD
NSKNTLYLQMNSLRAEDTAVYYCTR**QRYRGPKYYYYMDV**WGKGTTVTVSS

Heavy chain variable region sequence of M0310-C06 (SEQ ID NO: 11):

EVQLLESGGGLVQPGGSLRLSCAASGFTFS**RYVMV**WVRQAPGKGLEWVS**RIYPSGGMTQYADSVKG**RFTISRD
NSKNTLYLQMNSLRAEDTAVYYCTR**QRYRGPKYYYYMDV**WGKGTTVTVSS

Heavy chain variable region sequence of M0192-F01 (SEQ ID NO: 12):

EVQLLESGGGLVQPGGSLRLSCAASGFTFS**WYNMA**WVRQAPGKGLEWVS**RIYPSGGMTQYADSVKG**RFTISRD
NSKNTLYLQMNSLRAEDTAVYYCTR**QRYRGPKYYYYMDV**WGKGTTVTVSS

Heavy chain variable region sequence of M0191-A03 (SEQ ID NO: 13):

EVQLLESGGGLVQPGGSLRLSCAASGFTFS**QYIMH**WVRQAPGKGLEWVS**SIYPSGGNTKYADSVKG**RFTISRD
NSKNTLYLQMNSLRAEDTAVYYCTR**QRYRGPKYYYYMDV**WGKGTTVTVSS

Heavy chain variable region sequence of M0192-A01 (SEQ ID NO: 14):

EVQLLESGGGLVQPGGSLRLSCAASGFTFS**HYVMH**WVRQAPGKGLEWVS**SIYPSGGLTKYADSVKG**RFTISRD
NSKNTLYLQMNSLRAEDTAVYYCTR**QRYRGPKYYYYMDV**WGKGTTVTVSS

Heavy chain variable region sequence of M0191-H10 (SEQ ID NO: 15):

EVQLLESGGGLVQPGGSLRLSCAASGFTFS**WYTMH**WVRQAPGKGLEWVS**SIYPSGGFTRYADSVKG**RFTISRD
NSKNTLYLQMNSLRAEDTAVYYCTR**QRYRGPKYYYYMDV**WGKGTTVTVSS

Heavy chain variable region sequence of M0177-C12 (SEQ ID NO: 16):

EVQLLESGGGLVQPGGSLRLSCAASGFTFS**FYHMH**WVRQAPGKGLEWVS**RIVPSGGMTRYADSVKG**RFTISRD
NSKNTLYLQMNSLRAEDTAVYYCTR**QRYRGPKYYYYMDV**WGKGTTVTVSS

Heavy chain variable region sequence of M0184-B04 (SEQ ID NO: 17):

EVQLLESGGGLVQPGGSLRLSCAASGFTFS**FYSMH**WVRQAPGKGLEWVS**RIYPSGGVTKYADSVKG**RFTISRD NSKNTLYLQMNSLRAEDTAVYYCTR**QRYRGPKYYYYMDV**WGKGTTVTVSS

Heavy chain variable region sequence of M0308-H03 (SEQ ID NO: 18):

EVQLLESGGGLVQPGGSLRLSCAASGFTFS**QYVMH**WVRQAPGKGLEWVS**SIWPSGGKTTYADSVKG**RFTISRD NSKNTLYLQMNSLRAEDTAVYYCTR**QRYRGPKYYYYMDV**WGKGTTVTVSS

Heavy chain variable region sequence of M0192-D02 (SEQ ID NO: 19):

EVQLLESGGGLVQPGGSLRLSCAASGFTFS**QYVMH**WVRQAPGKGLEWVS**SIWPSGGFTKYADSVKG**RFTISRD NSKNTLYLQMNSLRAEDTAVYYCTR**QRYRGPKYYYYMDV**WGKGTTVTVSS

Heavy chain variable region sequence of M0310- G07 (SEQ ID NO: 20):

EVQLLESGGGLVQPGGSLRLSCAASGFTFS**FYNMH**WVRQAPGKGLEWVS**SIYPSGGVTRYADSVKG**RFTISRD NSKNTLYLQMNSLRAEDTAVYYCTR**QRYRGPKYYYYMDV**WGKGTTVTVSS

Heavy chain variable region sequence of M0192-F06 (SEQ ID NO: 21):

EVQLLESGGGLVQPGGSLRLSCAASGFTFS**WYVMH**WVRQAPGKGLEWVS**SIYPSGGKTSYADSVKG**RFTISRD NSKNTLYLQMNSLRAEDTAVYYCTR**QRYRGPKYYYYMDV**WGKGTTVTVSS

Heavy chain variable region sequence of M0310-A04 (SEQ ID NO: 22):

EVQLLESGGGLVQPGGSLRLSCAASGFTFS**PYIMH**WVRQAPGKGLEWVS**VIYPSGSKTNYADSVKG**RFTISRD NSKNTLYLQMNSLRAEDTAVYYCTR**QRYRGPKYYYYMDV**WGKGTTVTVSS

Heavy chain variable region sequence of M0310-F02 (SEQ ID NO: 23):

EVQLLESGGGLVQPGGSLRLSCAASGFTFS**RYTMR**WVRQAPGKGLEWVS**SIWPSGGMTRYADSVKG**RFTISRD NSKNTLYLQMNSLRAEDTAVYYCTR**QRYRGPKYYYYMDV**WGQGTTVTVSS

Heavy chain variable region sequence of M0310-B09 (SEQ ID NO: 24):

EVQLLESGGGLVQPGGSLRLSCAASGFTFS**QYVMH**WVRQAPGKGLEWVS**SIYPSGGLTRYADSVKG**RFTISRD NSKNTLYLQMNSLRAEDTAVYYCTR**QRYRGPKYYYYMDV**WGQGTTVTVSS

Heavy chain variable region sequence of M0310-G06 (SEQ ID NO: 25):

EVQLLESGGGLVQPGGSLRLSCAASGFTFS**WYIMG**WVRQAPGKGLEWVS**YIYPSGGNTRYADSVKG**RFTISRD NSKNTLYLQMNSLRAEDTAVYYCTR**QRYRGPKYYYYMDV**WGKGTTVTVSS

Heavy chain variable region sequence of M0182-H01 (SEQ ID NO: 26):

EVQLLESGGGLVQPGGSLRLSCAASGFTFS**RYVMH**WVRQAPGKGLEWVS**SIWPSGGMTKYADSVKG**RFTISRDNSKN
TLYLQMNSLRAEDTAVYYCTR**QRYRGPKYYYYMDV**WGKGTTVTVSS

Heavy chain variable region sequence of M0178-A08 (SEQ ID NO: 27):

EVQLLESGGGLVQPGGSLRLSCAASGFTFS**FYIMG**WVRQAPGKGLEWVS**RIYPSGGATQYADSVKG**RFTISRD
NSKNTLYLQMNSLRAEDTAVYYCTR**QRYRGPKYYYYMDV**WGKGTTVTVSS

Heavy chain variable region sequence of M0184-D01 (SEQ ID NO: 28):

EVQLLESGGGLVQPGGSLRLSCAASGFTFS**FYVMG**WVRQAPGKGLEWVS**RIYPSGGLTQYADSVKG**RFTISRD
NSKNTLYLQMNSLRAEDTAVYYCTR**QRYRGPKYYYYMDV**WGKGTTVTVSS

Heavy chain variable region sequence of M0177-A06 (SEQ ID NO: 29):

EVQLLESGGGLVQPGGSLRLSCAASGFTFS**FYSMH**WVRQAPGKGLEWVS**RIYPSGGITSYADSVKG**RFTISRD
NSKNTLYLQMNSLRAEDTAVYYCTR**QRYRGPKYYYYMDV**WGKGTTVTVSS

Heavy chain variable region sequence of M0191-E04 (SEQ ID NO: 30):

EVQLLESGGGLVQPGGSLRLSCAASGFTFS**MYIMH**WVRQAPGKGLEWVS**SIYPSGGMTKYADSVKG**RFTISRD
NSKNTLYLQMNSLRAEDTAVYYCTR**QRYRGPKYYYYMDV**WGKGTTVTVSS

Heavy chain variable region sequence of M0191-H09 (SEQ ID NO: 31):

EVQLLESGGGLVQPGGSLRLSCAASGFTFS**MYVMH**WVRQAPGKGLEWVS**SIYPSGGLTKYADSVKG**RFTISRD
NSKNTLYLQMNSLRAEDTAVYYCTR**QRYRGPKYYYYMDV**WGKGTTVTVSS

Heavy chain variable region sequence of M0192-H04 (SEQ ID NO: 32):

EVQLLESGGGLVQPGGSLRLSCAASGFTFS**QYVMH**WVRQAPGKGLEWVS**RIYPSGGLTNYADSVKG**RFTISRD
NSKNTLYLQMNSLRAEDTAVYYCTR**QRYRGPKYYYYMDV**WGKGTTVTVSS

Heavy chain variable region sequence of M0192-A03 (SEQ ID NO: 33):

EVQLLESGGGLVQPGGSLRLSCAASGFTFS**WYVMQ**WVRQAPGKGLEWVS**SIYPSGGMTKYADSVKG**RFTISRD
NSKNTLYLQMNSLRAEDTAVYYCTR**QRYRGPKYYYYMDV**WGKGTTVTVSS

Heavy chain variable region sequence of M0310-G08 (SEQ ID NO: 34):

EVQLLESGGGLVQPGGSLRLSCAASGFTFS**WYIMG**WVRQAPGKGLEWVS**RIYPSGGSTHYADSVKG**RFTISRD
NSKNTLYLQMNSLRAEDTAVYYCTR**QRYRGPRYYYYIDA**WGQGTTVTVSS

Heavy chain variable region sequence of M0192-G05 (SEQ ID NO: 35):

EVQLLESGGGLVQPGGSLRLSCAASGFTFS**QYTMV**WVRQAPGKGLEWVS**RIYPSGGVTQYADSVKG**RFTISRD
NSKNTLYLQMNSLRAEDTAVYYCTR**QRYRGPKYYYYMDV**WGKGTTVTVSS

Heavy chain variable region sequence of M0183-B12 (SEQ ID NO: 36):

EVQLLESGGGLVQPGGSLRLSCAASGFTFS**WYVMY**WVRQAPGKGLEWVS**RIYPSGGITHYADSVKG**RFTISRD
NSKNTLYLQMNSLRAEDTAVYYCTR**QRYRGPKYYYYMDV**WGKGTTVTVSS

Heavy chain variable region sequence of M0308-F04 (SEQ ID NO: 37):

EVQLLESGGGLVQPGGSLRLSCAASGFTFS**FYVML**WVRQAPGKGLEWVS**SIWPSGGVTKYADSVKG**RFTISRD
NSKNTLYLQMNSLRAEDTAVYYCTR**QRYRGPKYYYYMDV**WGKGTTVTVSS

Heavy chain variable region sequence of M0182-B04 (SEQ ID NO: 38):

EVQLLESGGGLVQPGGSLRLSCAASGFTFS**WYVMQ**WVRQAPGKGLEWVS**YIYPSGGHTKYADSVKG**RFTISRD
NSKNTLYLQMNSLRAEDTAVYYCTR**QRYRGPKYYYYMDV**WGKGTTVTVSS

Heavy chain variable region sequence of M0310-F04 (SEQ ID NO: 39):

EVQLLESGGGLVQPGGSLRLSCAASGFTFS**RYSMN**WVRQAPGKGLEWVS**GIYPSGGKTKYADSVKG**RFTISRD
NSKNTLYLQMNSLRAEDTAVYYCTR**QRYRGPKYYYYMDV**WGKGTTVTVSS

Heavy chain variable region sequence of X211-A01 (SEQ ID NO:125)

EVQLLESGGGLVQPGGSLRLSCAASGFTFS**QYVMH**WVRQAPGKGLEWVS**SIWPSGGHTRYADSVKG**RFTISRD
NSK NTLYLQMNSLRAEDTAVYYCTR**QRYRGPKYYYYMDV**WGKGTTVTVSS

[0041] The present disclosure also provides germlined variants of any of the exemplary anti-FXIIa antibodies disclosed herein. A germlined variant contains one or more mutations in the framework regions as relative to its parent antibody towards the corresponding germline sequence. To make a germline variant, the heavy or light chain variable region sequence of the parent antibody or a portion thereof *(e.g.,* a framework sequence) can be used as a query against an antibody germline sequence database (e.g., www.bioinfo.org.uk/abs/, www.vbase2.org, or www.imgt.org) to identify the corresponding germline sequence used by the parent antibody and amino acid residue variations in one or more of the framework regions between the germline sequence and the parent antibody. One or more amino acid substitutions can then be introduced into the parent antibody based on the germline sequence to produce a germlined variant. As examples, clone X211-A01 (DX-4012) is a germline variant of clone M192-H11 As described herein, the anti-FXIIa antibody can be in any antibody form, including, but not limited to, intact (*i.e.*, full-length) antibodies, antigen-binding fragments thereof (such as Fab, Fab', F(ab')2, Fv), and single chain antibodies.

[0042] In some embodiments, the heavy chain of any of the anti-FXIIa antibodies as described herein may further comprise a heavy chain constant region (CH) or a portion thereof *(e.g.,* CH1, CH2, CH3, or a combination thereof). The heavy chain constant region can of any suitable origin, e.g., human, mouse, rat, or rabbit. In one specific example, the heavy chain constant region is from a human IgG (a gamma heavy chain). One exemplary human heavy chain constant region is provided below (SEQ ID NO:119):

ASTKGPSVFP LAPSSKSTSG GTAALGCLVK DYFPEPVTVS WNSGALTSGV HTFPAVLQSS
GLYSLSSVVT VPSSSLGTQT YICNVNHKPS NTKVDKRVEP KSCDKTHTCP PCPAPELLGG
PSVFLFPPKP KDTL**M**I**S**R**T**P EVTCVVVDVS HEDPEVKFNW YVDGVEVHNA KTKPREEQYN
STYRVVSVLT VLHQDWLNGK EYKCKVSNKA LPAPIEKTIS KAKGQPREPQ VYTLPPSREE
MTKNQVSLTC LVKGFYPSDI AVEWESNGQP ENNYKTTPPV LDSDGSFFLY SKLTVDKSRW
QQGNVFSCSV MHEALHNHYT QKSLSLSPG

[0043] The boldfaced/underlined residues represent those where mutations can be introduced to enhance FcRn binding and then serum half-life.

[0044] In some embodiments, the anti-FXIIa antibody comprises the heavy chain constant region of SEQ ID NO:119.

Alternatively, the heavy chain constant region of the antibodies described herein may comprise a single domain (e.g., CH1, CH2, or CH3) or a combination of any of the single domains, of a constant region (e.g., SEQ ID NO:119).

[0045] When needed, the anti-FXIIa antibody as described herein may comprise a modified constant region. For example, it may comprise a modified constant region that is immunologically inert, *e.g.,* does not trigger complement mediated lysis, or does not stimulate antibody-dependent cell mediated cytotoxicity (ADCC). ADCC activity can be assessed using methods disclosed in U.S. Pat. No. 5,500,362. In other embodiments, the constant region is modified as described in Eur. J. Immunol. (1999) 29:2613-2624; PCT Application No. PCT/GB99/01441; and/or UK Patent Application No. 9809951.8.

[0046] In some embodiments, the heavy chain constant region used in the anti-FXIIa antibodies described herein may comprise mutations (e.g., amino acid residue substitutions) to enhance a desired characteristic of the antibody, for example, increasing the binding activity to the neonatal Fc receptor (FcRn) and thus the serum half-life of the antibodies. It was known that binding to FcRn is critical for maintaining antibody homeostasis and regulating the serum half-life of antibodies. One or more *(e.g.,* 1, 2, 3, 4, 5, or more) mutations *(e.g.,* amino acid residue substitutions) may be introduced into the constant region at suitable positions (e.g., in CH2 region) to enhance FcRn binding and enhance the half-life of the antibody. Such mutations may be at a position(s) corresponding to 145, 147, and/or 149 of SEQ ID NO:119 (corresponding to positions 252, 254, and 256 based on the numbering system reported in Kabat et al., 1991, Sequences of Proteins of Immunological Interest, U.S. Public Health Service, National Institutes of Health, Washington, D. C.). See also Dall'Acqua et al., J.B.C., 2006, 281:23514-23524; Robbie et al., Antimicrob. Agents Chemother, 2013, 57(12):6147; and Dall'Acqua et al., J. Immunol. 2002 169:5171-5180. If a constant region other than SEQ ID NO:119 is used, the positions where mutations may be introduced can be determined either by aligning the amino acid sequence of that constant region with SEQ ID NO:119 to identify the positions corresponding to a position of interest (e.g., positions 145, 147, or 149 in SEQ ID NO:119). Alternatively, such positions can be determined by a well-recognized numbering system such as the Kabat numbering system noted above.

[0047] In some examples, a substitution mutation is introduced at the amino acid residue *(e.g.,* a methionine residue) at position 252 (position 145 of SEQ ID NO:119) of a heavy chain constant region, e.g., M→Y substitution. In some examples, a substitution mutation is introduced at position 254 (position 147 of SEQ ID NO:119) of a heavy chain constant region, *e.g.,* S→T substitution. In some examples, a substitution mutation is introduced at position 256 (position 149 of SEQ ID NO:119) of a heavy chain constant region, *e.g.,* T→E. In some examples, the threonine at position 256 is mutated to a glutamic acid residue. When desired, multiple mutations (e.g., amino acid substitutions) may be introduced into a heavy chain constant region, *e.g.,* any combination of the above-noted substitutions. In a specific example, a modified heavy chain constant region used in any of the anti-FXIIa antibodies can contain amino acid substitutions at positions 252, 254, and 256 (e.g., M252Y, S254T, and T256E; also known as a YTE variant). See, e.g., Dall'Acqua et al., J.B.C., 2006, 281:23514-23524; Robbie et al., Antimicrob. Agents Chemother, 2013, 57(12):6147; and Dall'Acqua et al., J. Immunol. 2002 169:5171-5180. The amino acid sequence below (SEQ ID NO:120) represents an exemplary modified heavy chain constant region having enhanced FcRn binding activity and thus serum half-life:

Exemplary heavy chain constant region sequence of a YTE variant (SEQ ID NO: 120):

ASTKGPSVFP LAPSSKSTSG GTAALGCLVK DYFPEPVTVS WNSGALTSGV HTFPAVLQSS
GLYSLSSVVT VPSSSLGTQT YICNVNHKPS NTKVDKRVEP KSCDKTHTCP PCPAPELLGG
PSVFLFPPKP KDTL**Y**I**T**R**E**P EVTCVVVDVS HEDPEVKFNW YVDGVEVHNA KTKPREEQYN
STYRVVSVLT VLHQDWLNGK EYKCKVSNKA LPAPIEKTIS KAKGQPREPQ VYTLPPSREE
MTKNQVSLTC LVKGFYPSDI AVEWESNGQP ENNYKTTPPV LDSDGSFFLY SKLTVDKSRW
QQGNVFSCSV MHEALHNHYT QKSLSLSPG

[0048] Amino acid residues that are mutated in SEQ ID NO: 120 relative to SEQ ID NO: 119 are shown in boldface.

[0049] Any of the anti-FXIIa antibodies described herein may further comprise a light chain that includes a light chain variable region and optionally, a light chain constant region, which can be any CL known in the art. In some examples, the CL is a kappa light chain. In other examples, the CL is a lambda light chain. Antibody heavy and light chain constant regions are well known in the art, *e.g.,* those provided in the IMGT database (www.imgt.org) or at www.vbase2.org/vbstat.php.

[0050] The anti-FXIIa antibody described herein may comprise a light chain variable region that comprises a light chain CDR3 set forth as SEQ ID NO: 116 (see Table 2). In some embodiments, the light chain variable region of the anti-FXIIa antibody may further comprise a light chain CDR1 region set forth as SEQ ID NO: 114 (see Table 2) and/or a light chain CDR2 region set forth as SEQ ID NO: 115 (see Table 2). For example, the anti-FXIIa antibody light chain variable region may comprise the light chain CDR1 of SEQ ID NO:114, the light chain CDR2 of SEQ ID NO:115, and the light chain CDR3 of SEQ ID NO: 116. In one example, the light chain variable region comprises the amino acid sequence of SEQ ID NO: 40. Alternatively, the light chain variable region is a germline variant of SEQ ID NO:40, which may contain one or more mutations in one or more frameworks toward the corresponding germline sequence. The

corresponding germline sequence can be identified by methods known in the art and those described herein.

[0051] An exemplary light chain variable region for anti-FXIIa antibodies is provided below (CDR regions are boldfaced).

[0052] Exemplary Light chain variable region for anti-FXIIa antibodies (SEQ ID NO: 40):

DIQMTQSPLSLPVTPGEPASISC**RSSQSLLHSNGYNYLD**WYLQKPGQSPQLLIY**LGSNRAS**GVPDRFSGSGSG
TDFTLKISRVEAEDVGVYYC**MQALQTPWT**FGQGTKVEIK

[0053] Example Light chain variable region for anti-FXIIa antibodies (SEQ ID NO:126)

DIVMTQSPLSLPVTPGEPASISC**RSSQSLLHSNGYNYLD**WYLQKPGQSPQLLIY**LGSNRAS**
GVPDRFSGSGSGTDFTLKISRVEAEDVGVYYC**MQALQTPWT**FGQGTKVEIK

Table 2: Light chain CDR sequences of anti-FXIIa antibodies (for reference)

	CDR1	CDR2	CD3
Exemplary antibody	RSSQSLLHSNGYNYLD (SEQ ID NO: 114)	LGSNRAS (SEQ ID NO: 115)	MQALQTPWT (SEQ ID NO: 116)

[0054] In some embodiments, the anti-FXIIa antibody described herein comprises a heavy chain variable region comprising the amino acid sequence of any of SEQ ID NO: 1-39; and a light chain variable region comprising the amino acid sequence of SEQ ID NO: 40.

[0055] The anti-FXIIa antibody described herein *(e.g.,* X211-A01; *aka* DX-4012) comprises a heavy chain variable region comprising the amino acid sequence of SEQ ID NO:125 and a light chain variable region comprising the amino acid sequence of SEQ ID NO:126.

[0056] Also disclosed are functional variants of any of the exemplary anti-FXIIa antibodies as disclosed herein *(e.g.,* the 44 antibodies clones described above). In some examples, the anti-FXIIa antibody is a functional variant of an antibody comprising a heavy chain variable region provided by any one of SEQ ID NO: 1-39,and a light chain variable region provided by SEQ ID NO: 40. A functional variant can comprise up to 5 *(e.g.,* 4, 3, 2, or 1) amino acid residue variations in one or more of the CDR regions of the antibody and binds the same epitope of FXIIa with substantially similar affinity *(e.g.,* having a K_D value in the same order). In one example, the amino acid residue variations are conservative amino acid residue substitutions. As used herein, a "conservative amino acid substitution" refers to an amino acid substitution that does not alter the relative charge or size characteristics of the protein in which the amino acid substitution is made. Variants can be prepared according to methods for altering polypeptide sequence known to one of ordinary skill in the art such as are found in references which compile such methods, e.g. Molecular Cloning: A Laboratory Manual, J. Sambrook, et al., eds., Second Edition, Cold Spring Harbor Laboratory Press, Cold Spring Harbor, New York, 1989, or Current Protocols in Molecular Biology, F.M. Ausubel, et al., eds., John Wiley & Sons, Inc., New York. Conservative substitutions of amino acids include substitutions made amongst amino acids within the following groups: (a) M, I, L, V; (b) F, Y, W; (c) K, R, H; (d) A, G; (e) S, T; (f) Q, N; and (g) E, D.

[0057] In some aspects the anti-FXIIa antibody comprises heavy chain CDRs that are at least 80% (e.g., 85%, 90%, 95%, or 98%) identical to any one of the heavy chain CDR sequence provided by SEQ ID NO: 41-113,SEQ ID NO: 121-124, and SEQ ID NO: 127 and/or light chain CDR sequences that are at least 80% (e.g., 85%, 90%, 95%, or 98%) identical to at least one of the corresponding light chain CDRs provided by SEQ ID NO: 114-116. In some aspects the anti-FXIIa antibody comprises a heavy chain variable region that is at least 80% (*e.g.*, 85%, 90%, 95%, or 98%) identical to the heavy chain variable region of any of SEQ ID NO: 1-39 and/or light chain variable region that is at least 80% *(e.g.,* 85%, 90%, 95%, or 98%) identical to the light chain variable region provided by SEQ ID NO:40.

[0058] The "percent identity" of two amino acid sequences is determined using the algorithm of Karlin and Altschul Proc. Natl. Acad. Sci. USA 87:2264-68, 1990, modified as in Karlin and Altschul Proc. Natl. Acad. Sci. USA 90:5873-77, 1993. Such an algorithm is incorporated into the NBLAST and XBLAST programs (version 2.0) of Altschul, et al. J. Mol. Biol. 215:403-10, 1990. BLAST protein searches can be performed with the XBLAST program, score=50, wordlength=3 to obtain amino acid sequences homologous to the protein molecules of interest. Where gaps exist between two sequences, Gapped BLAST can be utilized as described in Altschul et al., Nucleic Acids Res. 25(17):3389-3402, 1997. When utilizing BLAST and Gapped BLAST programs, the default parameters of the respective programs (e.g., XBLAST and NBLAST) can be used.

[0059] In addition, the anti-FXIIa antibody can include one or more other residues that are identified based on the

crystal structures discussed in Example 5 herein as being involved in interacting with the C chain of FXIIa. These residues can be located in the VH or the VL chain. Examples include T28, S30, Q31, W52, P53, S54, G55, G56, H57, R59, N74, R100, Y101, R102, G103, P104, K105, Y106, Y107, and Y108 in the heavy chain variable region, and H31, N33, Y35, Y54, L55, N58, and T99 in the light chain variable region.

[0060] Also provided herein are antibodies that target specific residues in FXIIa. The antibody preferentially binds to FXIIa but not bind to FXII. In some embodiments, the antibody that specifically binds to FXIIa interacts with one or more of the residues (e.g., at least 3, 5, 8, 10, 15, 20, 25, 30, 35, 40 or 45) of the C chain of FXIIa, including L390, Y391, W392, G393, H394, S395, F396, C397, H412, C413, L414, Q415, D416, R432, N433, V456, Y458, H507, F509, E510, G511, A512, E513, Y515, D557, A558, C559, Q560, G561, D562, S563, 1584, S585, W586, G587, S588, G589, C590, G591, D592, and G597. These residues are identified as interacting with one or more residues in of the heavy chain variable region and the light chain variable region of the anti-FXIIa antibody, according to the crystal structures described in Example 5 below.

[0061] The amino acid sequence (SEQ ID NO:128) of an exemplary human FXIIa is provided below and the above noted residues are highlighted in boldface:

1 mrallllgfl lvslestlsi ppweapkehk ykaeehtvvl tvtgepchfp fqyhrqlyhk
61 cthkgrpgpq pwcattpnfd qdqrwgycle pkkvkdhcsk hspcqkggtc vnmpsgphcl
121 cpqhltgnhc qkekcfepql lrffhkneiw yrteqaavar cqckgpdahc qrlasqacrt
181 npclhggrcl eveghrlchc pvgytgafcd vdtkascydg rglsyrglar ttlsgapcqp
241 waseatyrnv taeqarnwgl gghafcrnpd ndirpwcfvl nrdrlsweyc dlaqcqtptq
301 aapptpvspr lhvplmpaqp appkpqpttr tppqsqtpga lpakreqpps ltrngplscg
361 qrlrkslssm trvvgglval rgahpyiaa**l** **ywghsfc**ags liapcwvlta a**hclqd**rpap
421 edltvvlgqe r**rn**hscepcq tlavrsyrlh eafsp**v**s**y**qh dlallrlqed adgscallsp
481 yvqpvclpsg aarpsettlc qvagwg**hqfe** **gae**e**y**asflq eaqvpflsle rcsapdvhgs
541 silpgmlcag fleggt**dacq** **gds**ggplvce dqaaerrltl qgi**iswgsgc** **gd**rnkp**g**vyt
601 dvayylawir ehtvs

[0062] Other exemplary FXIIa sequences can include human, mouse, or rat FXIIa sequences, a sequence that is 80%, 85%, 90%, 95%, 96%, 97%, 98%, or 99% identical to one of these sequences, or a fragment thereof.

[0063] Interacting means that the distance between two residues in a complex formed by two binding partners is lower than a predetermined value, e.g., < 6 Å, < 4 Å, or < 2 Å. For example, an interacting residue in one binding partner can have has at least 1 atom within a given threshold (e.g., < 6 Å, < 4 Å, or < 2 Å) of at least 1 atom from a residue of the other binding partner on the complexed structure. Interacting does not require actual binding. Interacting residues are suggested as involved in antibody recognition.

[0064] In some embodiments, the antibodies described herein bind human active FXIIa at an epitope comprising one or more of the residues listed above. An "epitope" refers to the site on a target compound that is bound by an antibody such as a Fab or full length antibody. An epitope can be linear, which is typically 6-15 aa in length. Alternatively, the epitope can be conformational.

[0065] In some examples, the antibody that specifically binds to FXIIa described herein binds an epitope that comprises the following segments of SEQ ID NO: 128: residues 390-397, residues 412-416, residues 432-433, residues 456-458, residues 507-515, residues 557-563, and/or residues 584-592.

Preparation of anti-FXIIa antibodies

[0066] Antibodies capable of binding FXIIa as described herein can be made by any method known in the art. See, for example, Harlow and Lane, (1998) Antibodies: A Laboratory Manual, Cold Spring Harbor Laboratory, New York.

[0067] In some embodiments, antibodies specific to a target antigen (e.g., FXIIa or the catalytic domain thereof) can be made by the conventional hybridoma technology. The full-length target antigen or a fragment thereof, optionally coupled to a carrier protein such as KLH, can be used to immunize a host animal for generating antibodies binding to that antigen. The route and schedule of immunization of the host animal are generally in keeping with established and conventional techniques for antibody stimulation and production, as further described herein. General techniques for production of mouse, humanized, and human antibodies are known in the art and are described herein. It is contemplated that any mammalian subject including humans or antibody producing cells therefrom can be manipulated to serve as the basis for production of mammalian, including human hybridoma cell lines. Typically, the host animal is inoculated intraperitoneally, intramuscularly, orally, subcutaneously, intraplantar, and/or intradermally with an amount of immunogen, including as described herein.

[0068] Hybridomas can be prepared from the lymphocytes and immortalized myeloma cells using the general somatic

cell hybridization technique of Kohler, B. and Milstein, C. (1975) Nature 256:495-497 or as modified by Buck, D. W., et al., In Vitro, 18:377-381 (1982). Available myeloma lines, including but not limited to X63-Ag8.653 and those from the Salk Institute, Cell Distribution Center, San Diego, Calif., USA, may be used in the hybridization. Generally, the technique involves fusing myeloma cells and lymphoid cells using a fusogen such as polyethylene glycol, or by electrical means well known to those skilled in the art. After the fusion, the cells are separated from the fusion medium and grown in a selective growth medium, such as hypoxanthine-aminopterin-thymidine (HAT) medium, to eliminate unhybridized parent cells. Any of the media described herein, supplemented with or without serum, can be used for culturing hybridomas that secrete monoclonal antibodies. As another alternative to the cell fusion technique, EBV immortalized B cells may be used to produce the anti-FXIIa monoclonal antibodies described herein. The hybridomas are expanded and subcloned, if desired, and supernatants are assayed for anti-immunogen activity by conventional immunoassay procedures (e.g., radioimmunoassay, enzyme immunoassay, or fluorescence immunoassay).

[0069] Hybridomas that may be used as source of antibodies encompass all derivatives, progeny cells of the parent hybridomas that produce monoclonal antibodies capable of interfering with the FXIIa activity. Hybridomas that produce such antibodies may be grown in vitro or in vivo using known procedures. The monoclonal antibodies may be isolated from the culture media or body fluids, by conventional immunoglobulin purification procedures such as ammonium sulfate precipitation, gel electrophoresis, dialysis, chromatography, and ultrafiltration, if desired. Undesired activity if present, can be removed, for example, by running the preparation over adsorbents made of the immunogen attached to a solid phase and eluting or releasing the desired antibodies off the immunogen. Immunization of a host animal with a target antigen or a fragment containing the target amino acid sequence conjugated to a protein that is immunogenic in the species to be immunized, e.g., keyhole limpet hemocyanin, serum albumin, bovine thyroglobulin, or soybean trypsin inhibitor using a bifunctional or derivatizing agent, for example maleimidobenzoyl sulfosuccinimide ester (conjugation through cysteine residues), N-hydroxysuccinimide (through lysine residues), glutaraldehyde, succinic anhydride, SOC1, or R1N=C=NR, where R and R1 are different alkyl groups, can yield a population of antibodies (e.g., monoclonal antibodies).

[0070] If desired, an antibody (monoclonal or polyclonal) of interest (e.g., produced by a hybridoma) may be sequenced and the polynucleotide sequence may then be cloned into a vector for expression or propagation. The sequence encoding the antibody of interest may be maintained in vector in a host cell and the host cell can then be expanded and frozen for future use. In an alternative, the polynucleotide sequence may be used for genetic manipulation to "humanize" the antibody or to improve the affinity (affinity maturation), or other characteristics of the antibody. For example, the constant region may be engineered to more resemble human constant regions to avoid immune response if the antibody is used in clinical trials and treatments in humans. It may be desirable to genetically manipulate the antibody sequence to obtain greater affinity to the target antigen and greater efficacy in inhibiting the activity of FXIIa. It will be apparent to one of skill in the art that one or more polynucleotide changes can be made to the antibody and still maintain its binding specificity to the target antigen.

[0071] In other embodiments, fully human antibodies can be obtained by using commercially available mice that have been engineered to express specific human immunoglobulin proteins. Transgenic animals that are designed to produce a more desirable (e.g., fully human antibodies) or more robust immune response may also be used for generation of humanized or human antibodies. Examples of such technology are Xenomouse^{RTM} from Amgen, Inc. (Fremont, Calif.) and HuMAb-Mouse^{RTM} and TC MouseTM from Medarex, Inc. (Princeton, N.J.). In another alternative, antibodies may be made recombinantly by phage display or yeast technology. See, for example, U.S. Pat. Nos. 5,565,332; 5,580,717; 5,733,743; and 6,265,150; and Winter et al., (1994) Annu. Rev. Immunol. 12:433-455, and. Alternatively, the phage display technology (McCafferty et al., (1990) Nature 348:552-553) can be used to produce human antibodies and antibody fragments in vitro, from immunoglobulin variable (V) domain gene repertoires from unimmunized donors.

[0072] Antigen-binding fragments of an intact antibody (full-length antibody) can be prepared via routine methods. For example, F(ab')2 fragments can be produced by pepsin digestion of an antibody molecule, and Fab fragments that can be generated by reducing the disulfide bridges of F(ab')2 fragments.

[0073] Genetically engineered antibodies, such as humanized antibodies, chimeric antibodies, single-chain antibodies, and bi-specific antibodies, can be produced via, e.g., conventional recombinant technology. In one example, DNA encoding a monoclonal antibodies specific to a target antigen can be readily isolated and sequenced using conventional procedures (e.g., by using oligonucleotide probes that are capable of binding specifically to genes encoding the heavy and light chains of the monoclonal antibodies). The hybridoma cells serve as a preferred source of such DNA. Once isolated, the DNA may be placed into one or more expression vectors, which are then transfected into host cells such as E. coli cells, simian COS cells, Chinese hamster ovary (CHO) cells, or myeloma cells that do not otherwise produce immunoglobulin protein, to obtain the synthesis of monoclonal antibodies in the recombinant host cells. See, *e.g.,* PCT Publication No. WO 87/04462. The DNA can then be modified, for example, by substituting the coding sequence for human heavy and light chain constant domains in place of the homologous murine sequences, Morrison et al., (1984) Proc. Nat. Acad. Sci. 81:6851, or by covalently joining to the immunoglobulin coding sequence all or part of the coding sequence for a non-immunoglobulin polypeptide. In that manner, genetically engineered antibodies, such as "chimeric"

or "hybrid" antibodies; can be prepared that have the binding specificity of a target antigen.

[0074] Techniques developed for the production of "chimeric antibodies" are well known in the art. See, e.g., Morrison et al. (1984) Proc. Natl. Acad. Sci. USA 81, 6851; Neuberger et al. (1984) Nature 312, 604; and Takeda et al. (1984) Nature 314:452.

[0075] Methods for constructing humanized antibodies are also well known in the art. See, e.g., Queen et al., Proc. Natl. Acad. Sci. USA, 86:10029-10033 (1989). In one example, variable regions of VH and VL of a parent non-human antibody are subjected to three-dimensional molecular modeling analysis following methods known in the art. Next, framework amino acid residues predicted to be important for the formation of the correct CDR structures are identified using the same molecular modeling analysis. In parallel, human VH and VL chains having amino acid sequences that are homologous to those of the parent non-human antibody are identified from any antibody gene database using the parent VH and VL sequences as search queries. Human VH and VL acceptor genes are then selected.

[0076] The CDR regions within the selected human acceptor genes can be replaced with the CDR regions from the parent non-human antibody or functional variants thereof. When necessary, residues within the framework regions of the parent chain that are predicted to be important in interacting with the CDR regions (see above description) can be used to substitute for the corresponding residues in the human acceptor genes.

[0077] A single-chain antibody can be prepared via recombinant technology by linking a nucleotide sequence coding for a heavy chain variable region and a nucleotide sequence coding for a light chain variable region. Preferably, a flexible linker is incorporated between the two variable regions. Alternatively, techniques described for the production of single chain antibodies (U.S. Patent Nos. 4,946,778 and 4,704,692) can be adapted to produce a phage or yeast scFv library and scFv clones specific to a FXIIa can be identified from the library following routine procedures. Positive clones can be subjected to further screening to identify those that inhibit FXIIa activity.

[0078] Antibodies obtained following a method known in the art and described herein can be characterized using methods well known in the art. For example, one method is to identify the epitope to which the antigen binds, or "epitope mapping." There are many methods known in the art for mapping and characterizing the location of epitopes on proteins, including solving the crystal structure of an antibody-antigen complex, competition assays, gene fragment expression assays, and synthetic peptide-based assays, as described, for example, in Chapter 11 of Harlow and Lane, Using Antibodies, a Laboratory Manual, Cold Spring Harbor Laboratory Press, Cold Spring Harbor, N.Y., 1999. In an additional example, epitope mapping can be used to determine the sequence to which an antibody binds. The epitope can be a linear epitope, i.e., contained in a single stretch of amino acids, or a conformational epitope formed by a three-dimensional interaction of amino acids that may not necessarily be contained in a single stretch (primary structure linear sequence). Peptides of varying lengths (e.g., at least 4-6 amino acids long) can be isolated or synthesized (e.g., recombinantly) and used for binding assays with an antibody. In another example, the epitope to which the antibody binds can be determined in a systematic screening by using overlapping peptides derived from the target antigen sequence and determining binding by the antibody. According to the gene fragment expression assays, the open reading frame encoding the target antigen is fragmented either randomly or by specific genetic constructions and the reactivity of the expressed fragments of the antigen with the antibody to be tested is determined. The gene fragments may, for example, be produced by PCR and then transcribed and translated into protein in vitro, in the presence of radioactive amino acids. The binding of the antibody to the radioactively labeled antigen fragments is then determined by immunoprecipitation and gel electrophoresis. Certain epitopes can also be identified by using large libraries of random peptide sequences displayed on the surface of phage particles (phage libraries). Alternatively, a defined library of overlapping peptide fragments can be tested for binding to the test antibody in simple binding assays. In an additional example, mutagenesis of an antigen binding domain, domain swapping experiments and alanine scanning mutagenesis can be performed to identify residues required, sufficient, and/or necessary for epitope binding. For example, domain swapping experiments can be performed using a mutant of a target antigen in which various fragments of the FXIIa polypeptide have been replaced (swapped) with sequences from a closely related, but antigenically distinct protein (such as another member of the neurotrophin protein family). By assessing binding of the antibody to the mutant FXIIa, the importance of the particular antigen fragment to antibody binding can be assessed.

[0079] Alternatively, competition assays can be performed using other antibodies known to bind to the same antigen to determine whether an antibody binds to the same epitope as the other antibodies. Competition assays are well known to those of skill in the art.

[0080] In some examples, an anti-FXIIa antibody is prepared by recombinant technology as exemplified below.

[0081] Nucleic acids encoding the heavy and light chain of an anti-FXIIa antibody as described herein can be cloned into one expression vector, each nucleotide sequence being in operable linkage to a suitable promoter. In one example, each of the nucleotide sequences encoding the heavy chain and light chain is in operable linkage to a distinct prompter. Alternatively, the nucleotide sequences encoding the heavy chain and the light chain can be in operable linkage with a single promoter, such that both heavy and light chains are expressed from the same promoter. When necessary, an internal ribosomal entry site (IRES) can be inserted between the heavy chain and light chain encoding sequences.

[0082] In some examples, the nucleotide sequences encoding the two chains of the antibody are cloned into two

vectors, which can be introduced into the same or different cells. When the two chains are expressed in different cells, each of them can be isolated from the host cells expressing such and the isolated heavy chains and light chains can be mixed and incubated under suitable conditions allowing for the formation of the antibody.

[0083] Generally, a nucleic acid sequence encoding one or all chains of an antibody can be cloned into a suitable expression vector in operable linkage with a suitable promoter using methods known in the art. For example, the nucleotide sequence and vector can be contacted, under suitable conditions, with a restriction enzyme to create complementary ends on each molecule that can pair with each other and be joined together with a ligase. Alternatively, synthetic nucleic acid linkers can be ligated to the termini of a gene. These synthetic linkers contain nucleic acid sequences that correspond to a particular restriction site in the vector. The selection of expression vectors/promoter would depend on the type of host cells for use in producing the antibodies.

[0084] A variety of promoters can be used for expression of the antibodies described herein, including, but not limited to, cytomegalovirus (CMV) intermediate early promoter, a viral LTR such as the *Rous sarcoma* virus LTR, HIV-LTR, HTLV-1 LTR, the simian virus 40 (SV40) early promoter, *E. coli* lac UV5 promoter, and the herpes simplex tk virus promoter.

[0085] Regulatable promoters can also be used. Such regulatable promoters include those using the lac repressor from *E. coli* as a transcription modulator to regulate transcription from lac operator-bearing mammalian cell promoters [Brown, M. et al., Cell, 49:603-612 (1987)], those using the tetracycline repressor (tetR) [Gossen, M., and Bujard, H., Proc. Natl. Acad. Sci. USA 89:5547-5551 (1992); Yao, F. et al., Human Gene Therapy, 9:1939-1950 (1998); Shockelt, P., et al., Proc. Natl. Acad. Sci. USA, 92:6522-6526 (1995)]. Other systems include FK506 dimer, VP16 or p65 using astradiol, RU486, diphenol murislerone, or rapamycin. Inducible systems are available from Invitrogen, Clontech and Ariad.

[0086] Regulatable promoters that include a repressor with the operon can be used. In one embodiment, the lac repressor from *E. coli* can function as a transcriptional modulator to regulate transcription from lac operator-bearing mammalian cell promoters [M. Brown et al., Cell, 49:603-612 (1987)]; Gossen and Bujard (1992); [M. Gossen et al., Natl. Acad. Sci. USA, 89:5547-5551 (1992)] combined the tetracycline repressor (tetR) with the transcription activator (VP 16) to create a tetR-mammalian cell transcription activator fusion protein, tTa (tetR-VP 16), with the tetO-bearing minimal promoter derived from the human cytomegalovirus (hCMV) major immediate-early promoter to create a tetR-tet operator system to control gene expression in mammalian cells. In one embodiment, a tetracycline inducible switch is used. The tetracycline repressor (tetR) alone, rather than the tetR-mammalian cell transcription factor fusion derivatives can function as potent trans-modulator to regulate gene expression in mammalian cells when the tetracycline operator is properly positioned downstream for the TATA element of the CMVIE promoter (Yao et al., Human Gene Therapy). One particular advantage of this tetracycline inducible switch is that it does not require the use of a tetracycline repressor-mammalian cells transactivator or repressor fusion protein, which in some instances can be toxic to cells (Gossen et al., Natl. Acad. Sci. USA, 89:5547-5551 (1992); Shockett et al., Proc. Natl. Acad. Sci. USA, 92:6522-6526 (1995)), to achieve its regulatable effects.

[0087] Additionally, the vector can contain, for example, some or all of the following: a selectable marker gene, such as the neomycin gene for selection of stable or transient transfectants in mammalian cells; enhancer/promoter sequences from the immediate early gene of human CMV for high levels of transcription; transcription termination and RNA processing signals from SV40 for mRNA stability; SV40 polyoma origins of replication and ColE1 for proper episomal replication; internal ribosome binding sites (IRESes), versatile multiple cloning sites; and T7 and SP6 RNA promoters for in vitro transcription of sense and antisense RNA. Suitable vectors and methods for producing vectors containing transgenes are well known and available in the art.

[0088] Examples of polyadenylation signals useful to practice the methods described herein include, but are not limited to, human collagen I polyadenylation signal, human collagen II polyadenylation signal, and SV40 polyadenylation signal.

[0089] One or more vectors (e.g., expression vectors) comprising nucleic acids encoding any of the antibodies may be introduced into suitable host cells for producing the antibodies. The host cells can be cultured under suitable conditions for expression of the antibody or any polypeptide chain thereof. Such antibodies or polypeptide chains thereof can be recovered by the cultured cells *(e.g.,* from the cells or the culture supernatant) via a conventional method, *e.g.,* affinity purification. If necessary, polypeptide chains of the antibody can be incubated under suitable conditions for a suitable period of time allowing for production of the antibody.

[0090] In some embodiments, methods for preparing an antibody described herein involve a recombinant expression vector that encodes both the heavy chain and the light chain of an anti-FXIIa antibody, as also described herein. The recombinant expression vector can be introduced into a suitable host cell *(e.g.,* a dhfr- CHO cell) by a conventional method, *e.g.,* calcium phosphate-mediated transfection. Positive transformant host cells can be selected and cultured under suitable conditions allowing for the expression of the two polypeptide chains that form the antibody, which can be recovered from the cells or from the culture medium. When necessary, the two chains recovered from the host cells can be incubated under suitable conditions allowing for the formation of the antibody.

[0091] In one example, two recombinant expression vectors are provided, one encoding the heavy chain of the anti-

FXIIa antibody and the other encoding the light chain of the anti-FXIIa antibody. Both of the two recombinant expression vectors can be introduced into a suitable host cell *(e.g.,* dhfr- CHO cell) by a conventional method, *e.g.,* calcium phosphate-mediated transfection. Alternatively, each of the expression vectors can be introduced into a suitable host cells. Positive transformants can be selected and cultured under suitable conditions allowing for the expression of the polypeptide chains of the antibody. When the two expression vectors are introduced into the same host cells, the antibody produced therein can be recovered from the host cells or from the culture medium. If necessary, the polypeptide chains can be recovered from the host cells or from the culture medium and then incubated under suitable conditions allowing for formation of the antibody. When the two expression vectors are introduced into different host cells, each of them can be recovered from the corresponding host cells or from the corresponding culture media. The two polypeptide chains can then be incubated under suitable conditions for formation of the antibody.

[0092] Standard molecular biology techniques are used to prepare the recombinant expression vector, transfect the host cells, select for transformants, culture the host cells and recovery of the antibodies from the culture medium. For example, some antibodies can be isolated by affinity chromatography with a Protein A or Protein G coupled matrix.

[0093] Any of the nucleic acids encoding the heavy chain, the light chain, or both of an anti-FXIIa antibody as described herein, vectors (e.g., expression vectors) containing such; and host cells comprising the vectors are within the scope of the present disclosure.

Pharmaceutical compositions

[0094] The antibodies, as well as the encoding nucleic acids or nucleic acid sets, vectors comprising such, or host cells comprising the vectors, as described herein can be mixed with a pharmaceutically acceptable carrier (excipient) to form a pharmaceutical composition for use in treating a target disease. "Acceptable" means that the carrier must be compatible with the active ingredient of the composition (and preferably, capable of stabilizing the active ingredient) and not deleterious to the subject to be treated. Pharmaceutically acceptable excipients (carriers) including buffers, which are well known in the art. See, e.g., Remington: The Science and Practice of Pharmacy 20th Ed. (2000) Lippincott Williams and Wilkins, Ed. K. E. Hoover.

[0095] The pharmaceutical compositions to be used in the present methods can comprise pharmaceutically acceptable carriers, excipients, or stabilizers in the form of lyophilized formulations or aqueous solutions. (Remington: The Science and Practice of Pharmacy 20th Ed. (2000) Lippincott Williams and Wilkins, Ed. K. E. Hoover). Acceptable carriers, excipients, or stabilizers are nontoxic to recipients at the dosages and concentrations used, and may comprise buffers such as phosphate, citrate, and other organic acids; antioxidants including ascorbic acid and methionine; preservatives (such as octadecyldimethylbenzyl ammonium chloride; hexamethonium chloride; benzalkonium chloride, benzethonium chloride; phenol, butyl or benzyl alcohol; alkyl parabens such as methyl or propyl paraben; catechol; resorcinol; cyclohexanol; 3-pentanol; and m-cresol); low molecular weight (less than about 10 residues) polypeptides; proteins, such as serum albumin, gelatin, or immunoglobulins; hydrophilic polymers such as polyvinylpyrrolidone; amino acids such as glycine, glutamine, asparagine, histidine, arginine, or lysine; monosaccharides, disaccharides, and other carbohydrates including glucose, mannose, or dextrans; chelating agents such as EDTA; sugars such as sucrose, mannitol, trehalose or sorbitol; salt-forming counter-ions such as sodium; metal complexes (e.g. Zn-protein complexes); and/or non-ionic surfactants such as TWEEN™, PLURONICS™ or polyethylene glycol (PEG).

[0096] In some examples, the pharmaceutical composition described herein comprises liposomes containing the antibodies (or the encoding nucleic acids) which can be prepared by methods known in the art, such as described in Epstein, et al., Proc. Natl. Acad. Sci. USA 82:3688 (1985); Hwang, et al., Proc. Natl. Acad. Sci. USA 77:4030 (1980); and U.S. Pat. Nos. 4,485,045 and 4,544,545. Liposomes with enhanced circulation time are disclosed in U.S. Pat. No. 5,013,556. Particularly useful liposomes can be generated by the reverse phase evaporation method with a lipid composition comprising phosphatidylcholine, cholesterol and PEGderivatized phosphatidylethanolamine (PEG-PE). Liposomes are extruded through filters of defined pore size to yield liposomes with the desired diameter.

[0097] The antibodies, or the encoding nucleic acid(s), may also be entrapped in microcapsules prepared, for example, by coacervation techniques or by interfacial polymerization, for example, hydroxymethylcellulose or gelatin-microcapsules and poly-(methylmethacylate) microcapsules, respectively, in colloidal drug delivery systems (for example, liposomes, albumin microspheres, microemulsions, nano-particles and nanocapsules) or in macroemulsions. Such techniques are known in the art, see, e.g., Remington, The Science and Practice of Pharmacy 20th Ed. Mack Publishing (2000).

[0098] In other examples, the pharmaceutical composition described herein can be formulated in sustained-release format. Suitable examples of sustained-release preparations include semipermeable matrices of solid hydrophobic polymers containing the antibody, which matrices are in the form of shaped articles, e.g. films, or microcapsules. Examples of sustained-release matrices include polyesters, hydrogels (for example, poly(2-hydroxyethyl-methacrylate), or poly(v nylalcohol)), polylactides (U.S. Pat. No. 3,773,919), copolymers of L-glutamic acid and 7 ethyl-L-glutamate, non-degradable ethylene-vinyl acetate, degradable lactic acid-glycolic acid copolymers such as the LUPRON DEPOT™ (injectable microspheres composed of lactic acid-glycolic acid copolymer and leuprolide acetate), sucrose acetate isobutyrate, and

poly-D-(-)-3-hydroxybutyric acid.

[0099] The pharmaceutical compositions to be used for in vivo administration must be sterile. This is readily accomplished by, for example, filtration through sterile filtration membranes. Therapeutic antibody compositions are generally placed into a container having a sterile access port, for example, an intravenous solution bag or vial having a stopper pierceable by a hypodermic injection needle.

[0100] The pharmaceutical compositions described herein can be in unit dosage forms such as tablets, pills, capsules, powders, granules, solutions or suspensions, or suppositories, for oral, parenteral or rectal administration, or administration by inhalation or insufflation.

[0101] For preparing solid compositions such as tablets, the principal active ingredient can be mixed with a pharmaceutical carrier, e.g., conventional tableting ingredients such as corn starch, lactose, sucrose, sorbitol, talc, stearic acid, magnesium stearate, dicalcium phosphate or gums, and other pharmaceutical diluents, e.g., water, to form a solid preformulation composition containing a homogeneous mixture of a compound of the present invention, or a non-toxic pharmaceutically acceptable salt thereof. When referring to these preformulation compositions as homogeneous, it is meant that the active ingredient is dispersed evenly throughout the composition so that the composition may be readily subdivided into equally effective unit dosage forms such as tablets, pills and capsules. This solid preformulation composition is then subdivided into unit dosage forms of the type described above containing from 0.1 to about 500 mg of the active ingredient of the present invention. The tablets or pills of the novel composition can be coated or otherwise compounded to provide a dosage form affording the advantage of prolonged action. For example, the tablet or pill can comprise an inner dosage and an outer dosage component, the latter being in the form of an envelope over the former. The two components can be separated by an enteric layer that serves to resist disintegration in the stomach and permits the inner component to pass intact into the duodenum or to be delayed in release. A variety of materials can be used for such enteric layers or coatings, such materials including a number of polymeric acids and mixtures of polymeric acids with such materials as shellac, cetyl alcohol and cellulose acetate.

[0102] Suitable surface-active agents include, in particular, non-ionic agents, such as polyoxyethylenesorbitans *(e.g.,* Tween™ 20, 40, 60, 80 or 85) and other sorbitans *(e.g.,* Span™ 20, 40, 60, 80 or 85). Compositions with a surface-active agent will conveniently comprise between 0.05 and 5% surface-active agent, and can be between 0.1 and 2.5%. It will be appreciated that other ingredients may be added, for example mannitol or other pharmaceutically acceptable vehicles, if necessary.

[0103] Suitable emulsions may be prepared using commercially available fat emulsions, such as Intralipid™, Liposyn™, Infonutrol™, Lipofundin™ and Lipiphysan™. The active ingredient may be either dissolved in a pre-mixed emulsion composition or alternatively it may be dissolved in an oil (e.g., soybean oil, safflower oil, cottonseed oil, sesame oil, corn oil or almond oil) and an emulsion formed upon mixing with a phospholipid (e.g. egg phospholipids, soybean phospholipids or soybean lecithin) and water. It will be appreciated that other ingredients may be added, for example glycerol or glucose, to adjust the tonicity of the emulsion. Suitable emulsions will typically contain up to 20% oil, for example, between 5 and 20%. The fat emulsion can comprise fat droplets between 0.1 and 1.0 .im, particularly 0.1 and 0.5 .im, and have a pH in the range of 5.5 to 8.0.

[0104] The emulsion compositions can be those prepared by mixing an antibody with Intralipid™ or the components thereof (soybean oil, egg phospholipids, glycerol and water).

[0105] Pharmaceutical compositions for inhalation or insufflation include solutions and suspensions in pharmaceutically acceptable, aqueous or organic solvents, or mixtures thereof, and powders. The liquid or solid compositions may contain suitable pharmaceutically acceptable excipients as set out above. In some embodiments, the compositions are administered by the oral or nasal respiratory route for local or systemic effect.

[0106] Compositions in preferably sterile pharmaceutically acceptable solvents may be nebulised by use of gases. Nebulised solutions may be breathed directly from the nebulising device or the nebulising device may be attached to a face mask, tent or intermittent positive pressure breathing machine. Solution, suspension or powder compositions may be administered, preferably orally or nasally, from devices which deliver the formulation in an appropriate manner.

Methods of treatment

[0107] Any of the antibodies, as well as the encoding nucleic acids or nucleic acid sets, vectors comprising such, or host cells comprising the vectors, described herein are useful for treating a namely disease or disorder associated with aberrant contact system activation, namely diseases associated with contact system activation, diseases associated with aberrant contact system activation (e.g., HAE) or ocular diseases.

[0108] To practice the method disclosed herein, an effective amount of the pharmaceutical composition described herein can be administered to a subject *(e.g.,* a human) in need of the treatment via a suitable route, such as intravenous administration, e.g., as a bolus or by continuous infusion over a period of time, by intramuscular, intraperitoneal, intracerebrospinal, subcutaneous, intra-articular, intrasynovial, intrathecal, oral, inhalation or topical routes. Commercially available nebulizers for liquid formulations, including jet nebulizers and ultrasonic nebulizers are useful for administration.

Liquid formulations can be directly nebulized and lyophilized powder can be nebulized after reconstitution. Alternatively, the antibodies as described herein can be aerosolized using a fluorocarbon formulation and a metered dose inhaler, or inhaled as a lyophilized and milled powder.

[0109] The subject to be treated by the methods described herein can be a mammal, more preferably a human. Mammals include, but are not limited to, farm animals, sport animals, pets, primates, horses, dogs, cats, mice and rats. A human subject who needs the treatment may be a human patient having, at risk for, or suspected of having a target disease/disorder, such as hereditary angioedema (HAE), thrombosis, or ocular diseases. A subject having a target disease or disorder can be identified by routine medical examination, e.g., laboratory tests, organ functional tests, CT scans, or ultrasounds. A subject suspected of having any of such target disease/disorder might show one or more symptoms of the disease/disorder. A subject at risk for the disease/disorder can be a subject having one or more of the risk factors for that disease/disorder.

[0110] The methods and compositions described herein may be used to treat any disease or disorder associated with contact system activation and/or pKal signaling pathways. In some embodiments, the target disease is thrombosis, including thrombosis associated with atrial fibrillation, DVT, pulmonary embolism, stroke, or other arterial or venous thrombotic events. Thrombosis (e.g., venous thrombosis or arterial thrombosis) refers to the formation of blood clots inside a blood vessel, which may obstruct the flow of blood through the circulation system. Subjects having or at risk for thrombosis can be identified by routine medical procedures.

[0111] In other embodiments, the disease that can be treated by the anti-FXIIa antibodies described herein can be a disease associated with the kallikrein system (e.g., the pKal system), including, but not limited to, macular edema, diabetic retinopathy, hypertensive retinopathy, age-related macular degeneration, and retinal vein occlusions. Examples of diseases or disorders associated with the contact activation system include, without limitation, rheumatoid arthritis, osteoarthritis, psoriatic arthritis, psoriasis, systemic lupus erythematosus, systemic lupus erythematosus nephritis, systemic mastocytosis, gout, intestinal bowel disease, oral mucositis, neuropathic pain, inflammatory pain, spinal stenosis-degenerative spine disease, arterial or venous thrombosis, post-operative ileus, aortic aneurysm, vasculitis, edema, acquired angioedema, idiopathic angioedema, anaphylasxis, idiopathic anaphylaxis, cerebral edema, pulmonary embolism, stroke, clotting on ventricular assistance devices or stents, clotting associated with the use of in-dwelling catheters or peripherally inserted central catheters, clotting associated with the use of an extracorporeal membrane oxygenation device, clotting associated with the use of a graft or fistula for dialysis, head trauma or peri-tumor brain edema, sepsis, acute middle cerebral artery (MCA), ischemic event (stroke), restenosis (e.g., after angioplasty), or burn injury.

[0112] In some embodiments, the disease that can be treated with the anti-FXIIa antibodies described herein is an ocular disease associated with contact activation system, including, but not limited to, macular edema, diabetic retinopathy, hypertensive retinopathy, age-related macular degeneration, and retinal vein occlusions.

[0113] In one example, the disease or condition that involves the contact system activation is hereditary angioedema (HAE). Hereditary angioedema (HAE) is also known as "Quincke edema," C1 esterase inhibitor deficiency, C1 inhibitor deficiency, and hereditary angioneurotic edema (HANE). HAE is characterized by recurrent episodes of severe swelling (angioedema), which can affect, *e.g.,* the limbs, face, genitals, gastrointestinal tract, and airway. Symptoms of HAE include, e.g., swelling in the arms, legs, lips, eyes, tongue, and/or throat; airway blockage that can involve throat swelling and sudden hoarseness; repeat episodes of abdominal cramping without obvious cause; and/or swelling of the intestines, which can be severe and can lead to abdominal cramping, vomiting, dehydration, diarrhea, pain, and/or shock. About one-third of individuals with this HAE develop a non-itchy rash called erythema marginatum during an attack.

[0114] Swelling of the airway can be life threatening and causes death in some patients. Mortality rates are estimated at 15-33%. HAE leads to about 15,000-30,000 emergency department visits per year.

[0115] Trauma or stress, e.g., dental procedures, sickness (e.g., viral illnesses such as colds and the flu), menstruation, and surgery can trigger an attack of angioedema. To prevent acute attacks of HAE, patients can attempt to avoid specific stimuli that have previously caused attacks. However, in many cases, an attack occurs without a known trigger. Typically, HAE symptoms first appear in childhood and worsen during puberty. On average, untreated individuals have an attack every 1 to 2 weeks, and most episodes last for about 3 to 4 days (ghr.nlm.nih.gov/condition/hereditary-angioedema). The frequency and duration of attacks vary greatly among people with hereditary angioedema, even among people in the same family.

[0116] There are three types of HAE, known as types I, II, and III. It is estimated that HAE affects 1 in 50,000 people, that type I accounts for about 85 percent of cases, type II accounts for about 15 percent of cases, and type III is very rare. Type III is the most newly described form and was originally thought to occur only in women, but families with affected males have been identified.

[0117] HAE is inherited in an autosomal dominant pattern, such that an affected person can inherit the mutation from one affected parent. New mutations in the gene can also occur, and thus HAE can also occur in people with no history of the disorder in their family. It is estimated that 20-25% of cases result from a new spontaneous mutation.

[0118] Mutations in the SERPING1 gene cause hereditary angioedema type I and type II. The SERPING1 gene provides instructions for making the C1 inhibitor protein, which is important for controlling inflammation. C1 inhibitor

blocks the activity of certain proteins that promote inflammation. Mutations that cause hereditary angioedema type I lead to reduced levels of C1 inhibitor in the blood. In contrast, mutations that cause type II result in the production of a C1 inhibitor that functions abnormally. Without the proper levels of functional C1 inhibitor, excessive amounts of bradykinin are generated. Bradykinin promotes inflammation by increasing the leakage of fluid through the walls of blood vessels into body tissues. Excessive accumulation of fluids in body tissues causes the episodes of swelling seen in individuals with hereditary angioedema type I and type II.

[0119] Mutations in the F12 gene are associated with some cases of hereditary angioedema type III, also known as HAE with normal C1 inhibitor. The F12 gene provides instructions for making coagulation FXII. In addition to playing a critical role in blood clotting (coagulation), factor XII is also an important stimulator of inflammation and is involved in the production of bradykinin. Certain mutations in the F12 gene result in the production of factor XII with increased activity. As a result, more bradykinin is generated and blood vessel walls become more leaky, which leads to episodes of swelling. The cause of other cases of hereditary angioedema type III remains unknown. Mutations in one or more as-yet unidentified genes may be responsible for the disorder in these cases.

[0120] HAE can present similarly to other forms of angioedema resulting from allergies or other medical conditions, but it differs significantly in cause and treatment. When hereditary angioedema is misdiagnosed as an allergy, it is most commonly treated with antihistamines, steroids, and/or epinephrine, which are typically ineffective in HAE, although epinephrine can be used for life-threatening reactions. Misdiagnoses have also resulted in unnecessary exploratory surgery for patients with abdominal swelling, and in some HAE patients abdominal pain has been incorrectly diagnosed as psychosomatic.

[0121] Symptoms of HAE can be assessed, for example, using questionnaires, e.g., questionnaires that are completed by patients, clinicians, or family members. Such questionnaires are known in the art and include, for example, visual analog scales. See, *e.g.,* McMillan, C.V. et al. Patient. 2012;5(2): 113-26.

[0122] As used herein, "an effective amount" refers to the amount of each active agent required to confer therapeutic effect on the subject, either alone or in combination with one or more other active agents. In some embodiments, the therapeutic effect is reduced FXIIa activity, reduced amounts of pKal or bradykinin or reduced vasodilation. Antibody inhibitors that specifically bind FXIIa but do not bind FXII may require a lower efficacious dose than antibody inhibitors that also bind FXII. Determination of whether an amount of the antibody achieved the therapeutic effect would be evident to one of skill in the art. Effective amounts vary, as recognized by those skilled in the art, depending on the particular condition being treated, the severity of the condition, the individual patient parameters including age, physical condition, size, gender and weight, the duration of the treatment, the nature of concurrent therapy (if any), the specific route of administration and like factors within the knowledge and expertise of the health practitioner. These factors are well known to those of ordinary skill in the art and can be addressed with no more than routine experimentation. It is generally preferred that a maximum dose of the individual components or combinations thereof be used, that is, the highest safe dose according to sound medical judgment.

[0123] Empirical considerations, such as the half-life, generally will contribute to the determination of the dosage. For example, antibodies that are compatible with the human immune system, such as humanized antibodies or fully human antibodies, may be used to prolong half-life of the antibody and to prevent the antibody being attacked by the host's immune system. Frequency of administration may be determined and adjusted over the course of therapy, and is generally, but not necessarily, based on treatment and/or suppression and/or amelioration and/or delay of a target disease/disorder. Alternatively, sustained continuous release formulations of an antibody may be appropriate. Various formulations and devices for achieving sustained release are known in the art.

[0124] In one example, dosages for an antibody as described herein may be determined empirically in individuals who have been given one or more administration(s) of the antibody. Individuals are given incremental dosages of the antagonist. To assess efficacy of the antagonist, an indicator of the disease/disorder can be followed.

[0125] Generally, for administration of any of the antibodies described herein, an initial candidate dosage can be about 2 mg/kg. For the purpose of the present disclosure, a typical daily dosage might range from about any of 0.1 μg/kg to 3 μg/kg to 30 μg/kg to 300 μg/kg to 3 mg/kg, to 30 mg/kg to 100 mg/kg or more, depending on the factors mentioned above. For repeated administrations over several days or longer, depending on the condition, the treatment is sustained until a desired suppression of symptoms occurs or until sufficient therapeutic levels are achieved to alleviate a target disease or disorder, or a symptom thereof. An exemplary dosing regimen comprises administering an initial dose of about 2 mg/kg, followed by a weekly maintenance dose of about 1 mg/kg of the antibody, or followed by a maintenance dose of about 1 mg/kg every other week. However, other dosage regimens may be useful, depending on the pattern of pharmacokinetic decay that the practitioner wishes to achieve. For example, dosing from one-four times a week is contemplated. In some embodiments, dosing ranging from about 3 μg/mg to about 2 mg/kg (such as about 3 μg/mg, about 10 μg/mg, about 30 μg/mg, about 100 μg/mg, about 300 μg/mg, about 1 mg/kg, and about 2 mg/kg) may be used. In some embodiments, dosing frequency is once every week, every 2 weeks, every 4 weeks, every 5 weeks, every 6 weeks, every 7 weeks, every 8 weeks, every 9 weeks, or every 10 weeks; or once every month, every 2 months, or every 3 months, or longer. The progress of this therapy is easily monitored by conventional techniques and assays. The

dosing regimen (including the antibody used) can vary over time.

[0126] In some embodiments, for an adult patient of normal weight, doses ranging from about 0.3 to 5.00 mg/kg may be administered. In some examples, the dosage of the anti-FXII antibody described herein *(e.g.,* DX-4012) can be 10 mg/kg. The particular dosage regimen, *i.e.*, dose, timing and repetition, will depend on the particular individual and that individual's medical history, as well as the properties of the individual agents (such as the half-life of the agent, and other considerations well known in the art).

[0127] For the purpose of the present disclosure, the appropriate dosage of an antibody as described herein will depend on the specific antibody, antibodies, and/or non-antibody peptide (or compositions thereof) employed, the type and severity of the disease/disorder, whether the antibody is administered for preventive or therapeutic purposes, previous therapy, the patient's clinical history and response to the antagonist, and the discretion of the attending physician. Typically the clinician will administer an antibody, until a dosage is reached that achieves the desired result. In some embodiments, the desired result is a decrease in thrombosis. Methods of determining whether a dosage resulted in the desired result would be evident to one of skill in the art. Administration of one or more antibodies can be continuous or intermittent, depending, for example, upon the recipient's physiological condition, whether the purpose of the administration is therapeutic or prophylactic, and other factors known to skilled practitioners. The administration of an antibody may be essentially continuous over a preselected period of time or may be in a series of spaced dose, e.g., either before, during, or after developing a target disease or disorder.

[0128] As used herein, the term "treating" refers to the application or administration of a composition including one or more active agents to a subject, who has a target disease or disorder, a symptom of the disease/disorder, or a predisposition toward the disease/disorder, with the purpose to cure, heal, alleviate, relieve, alter, remedy, ameliorate, improve, or affect the disorder, the symptom of the disease, or the predisposition toward the disease or disorder.

[0129] Alleviating a target disease/disorder includes delaying the development or progression of the disease, or reducing disease severity. Alleviating the disease does not necessarily require curative results. As used therein, "delaying" the development of a target disease or disorder means to defer, hinder, slow, retard, stabilize, and/or postpone progression of the disease. This delay can be of varying lengths of time, depending on the history of the disease and/or individuals being treated. A method that "delays" or alleviates the development of a disease, or delays the onset of the disease, is a method that reduces probability of developing one or more symptoms of the disease in a given time frame and/or reduces extent of the symptoms in a given time frame, when compared to not using the method. Such comparisons are typically based on clinical studies, using a number of subjects sufficient to give a statistically significant result.

[0130] "Development" or "progression" of a disease means initial manifestations and/or ensuing progression of the disease. Development of the disease can be detectable and assessed using standard clinical techniques as well known in the art. However, development also refers to progression that may be undetectable. For purpose of this disclosure, development or progression refers to the biological course of the symptoms. "Development" includes occurrence, recurrence, and onset. As used herein "onset" or "occurrence" of a target disease or disorder includes initial onset and/or recurrence.

[0131] In some embodiments, the antibodies described herein are administered to a subject in need of the treatment at an amount sufficient to inhibit the activity of one or both of the target antigen by at least 20% (e.g., 30%, 40%, 50%, 60%, 70%, 80%, 90% or greater) in vivo. In other embodiments, the antibodies are administered in an amount effective in reducing the activity level of a target antigens by at least 20% (e.g., 30%, 40%, 50%, 60%, 70%, 80%, 90% or greater).

[0132] Conventional methods, known to those of ordinary skill in the art of medicine, can be used to administer the pharmaceutical composition to the subject, depending upon the type of disease to be treated or the site of the disease. This composition can also be administered via other conventional routes, e.g., administered orally, parenterally, by inhalation spray, topically, rectally, nasally, buccally, vaginally or via an implanted reservoir. The term "parenteral" as used herein includes subcutaneous, intracutaneous, intravenous, intramuscular, intraarticular, intraarterial, intrasynovial, intrasternal, intrathecal, intralesional, and intracranial injection or infusion techniques. In addition, it can be administered to the subject via injectable depot routes of administration such as using 1-, 3-, or 6-month depot injectable or biodegradable materials and methods. In some examples, the pharmaceutical composition is administered intraocularly or intravitreally.

[0133] Injectable compositions may contain various carriers such as vegetable oils, dimethylactamide, dimethyformamide, ethyl lactate, ethyl carbonate, isopropyl myristate, ethanol, and polyols (glycerol, propylene glycol, liquid polyethylene glycol, and the like). For intravenous injection, water soluble antibodies can be administered by the drip method, whereby a pharmaceutical formulation containing the antibody and a physiologically acceptable excipient is infused. Physiologically acceptable excipients may include, for example, 5% dextrose, 0.9% saline, Ringer's solution or other suitable excipients. Intramuscular preparations, e.g., a sterile formulation of a suitable soluble salt form of the antibody, can be dissolved and administered in a pharmaceutical excipient such as Water-for-Injection, 0.9% saline, or 5% glucose solution.

[0134] In one embodiment, an antibody is administered via site-specific or targeted local delivery techniques. Examples of site-specific or targeted local delivery techniques include various implantable depot sources of the antibody or local

delivery catheters, such as infusion catheters, an indwelling catheter, or a needle catheter, synthetic grafts, adventitial wraps, shunts and stents or other implantable devices, site specific carriers, direct injection, or direct application. See, *e.g.,* PCT Publication No. WO 00/53211 and U.S. Pat. No. 5,981,568.

[0135] Targeted delivery of therapeutic compositions containing an antisense polynucleotide, expression vector, or subgenomic polynucleotides can also be used. Receptor-mediated DNA delivery techniques are described in, for example, Findeis et al., Trends Biotechnol. (1993) 11:202; Chiou et al., Gene Therapeutics: Methods And Applications Of Direct Gene Transfer (J. A. Wolff, ed.) (1994); Wu et al., J. Biol. Chem. (1988) 263:621; Wu et al., J. Biol. Chem. (1994) 269:542; Zenke et al., Proc. Natl. Acad. Sci. USA (1990) 87:3655; Wu et al., J. Biol. Chem. (1991) 266:338.

[0136] Therapeutic compositions containing a polynucleotide (e.g., those encoding the antibodies described herein) are administered in a range of about 100 ng to about 200 mg of DNA for local administration in a gene therapy protocol. In some embodiments, concentration ranges of about 500 ng to about 50 mg, about 1 μg to about 2 mg, about 5 μg to about 500 μg, and about 20 μg to about 100 μg of DNA or more can also be used during a gene therapy protocol.

[0137] The therapeutic polynucleotides and polypeptides described herein can be delivered using gene delivery vehicles. The gene delivery vehicle can be of viral or non-viral origin (see generally, Jolly, Cancer Gene Therapy (1994) 1:51; Kimura, Human Gene Therapy (1994) 5:845; Connelly, Human Gene Therapy (1995) 1:185; and Kaplitt, Nature Genetics (1994) 6:148). Expression of such coding sequences can be induced using endogenous mammalian or heterologous promoters and/or enhancers. Expression of the coding sequence can be either constitutive or regulated.

[0138] Viral-based vectors for delivery of a desired polynucleotide and expression in a desired cell are well known in the art. Exemplary viral-based vehicles include, but are not limited to, recombinant retroviruses (see, *e.g.,* PCT Publication Nos. WO 90/07936; WO 94/03622; WO 93/25698; WO 93/25234; WO 93/11230; WO 93/10218; WO 91/02805; U.S. Pat. Nos. 5,219,740 and 4,777,127; GB Patent No. 2,200,651; and EP Patent No. 0 345 242), alphavirusbased vectors (e.g., Sindbis virus vectors, Semliki forest virus (ATCC VR-67; ATCC VR-1247), Ross River virus (ATCC VR-373; ATCC VR-1246) and Venezuelan equine encephalitis virus (ATCC VR-923; ATCC VR-1250; ATCC VR 1249; ATCC VR-532)), and adeno-associated virus (AAV) vectors (see, e.g., PCT Publication Nos. WO 94/12649, WO 93/03769; WO 93/19191; WO 94/28938; WO 95/11984 and WO 95/00655). Administration of DNA linked to killed adenovirus as described in Curiel, Hum. Gene Ther. (1992) 3:147 can also be employed.

[0139] Non-viral delivery vehicles and methods can also be employed, including, but not limited to, polycationic condensed DNA linked or unlinked to killed adenovirus alone (see, *e.g.,* Curiel, Hum. Gene Ther. (1992) 3:147); ligand-linked DNA (see, e.g., Wu, J. Biol. Chem. (1989) 264:16985); eukaryotic cell delivery vehicles cells (see, *e.g.,* U.S. Pat. No. 5,814,482; PCT Publication Nos. WO 95/07994; WO 96/17072; WO 95/30763; and WO 97/42338) and nucleic charge neutralization or fusion with cell membranes. Naked DNA can also be employed. Exemplary naked DNA introduction methods are described in PCT Publication No. WO 90/11092 and U.S. Pat. No. 5,580,859. Liposomes that can act as gene delivery vehicles are described in U.S. Pat. No. 5,422,120; PCT Publication Nos. WO 95/13796; WO 94/23697; WO 91/14445; and EP Patent No. 0524968. Additional approaches are described in Philip, Mol. Cell. Biol. (1994) 14:2411, and in Woffendin, Proc. Natl. Acad. Sci. (1994) 91:1581.

[0140] The particular dosage regimen, i.e., dose, timing and repetition, used in the method described herein will depend on the particular subject and that subject's medical history.

[0141] In some embodiments, more than one antibody, or a combination of an antibody and another suitable therapeutic agent, may be administered to a subject in need of the treatment. The antibody can also be used in conjunction with other agents that serve to enhance and/or complement the effectiveness of the agents.

[0142] Treatment efficacy for a target disease/disorder can be assessed by methods well-known in the art.

Kits for use in alleviating diseases associated with contact system activation

[0143] The present disclosure also provides kits for use in alleviating diseases/disorders associated with contact system activation, such as HAE. Such kits can include one or more containers comprising an anti-FXIIa antibody, e.g., any of those described herein.

[0144] In some embodiments, the kit can comprise instructions for use in accordance with any of the methods described herein. The included instructions can comprise a description of administration of the anti-FXIIa antibody to treat, delay the onset, or alleviate a target disease as those described herein. The kit may further comprise a description of selecting an individual suitable for treatment based on identifying whether that individual has the target disease. In still other embodiments, the instructions comprise a description of administering an antibody to an individual at risk of the target disease.

[0145] The instructions relating to the use of an anti-FXIIa antibody generally include information as to dosage, dosing schedule, and route of administration for the intended treatment. The containers may be unit doses, bulk packages (e.g., multi-dose packages) or subunit doses. Instructions supplied in the kits of the invention are typically written instructions on a label or package insert *(e.g.,* a paper sheet included in the kit), but machine-readable instructions (e.g., instructions carried on a magnetic or optical storage disk) are also acceptable.

[0146] The label or package insert indicates that the composition is used for treating, delaying the onset and/or alleviating a disease or disorder associated with the pKal signaling pathway, such as HAE. Instructions may be provided for practicing any of the methods described herein.

[0147] The kits of this invention are in suitable packaging. Suitable packaging includes, but is not limited to, vials, bottles, jars, flexible packaging (e.g., sealed Mylar or plastic bags), and the like. Also contemplated are packages for use in combination with a specific device, such as an inhaler, nasal administration device (e.g., an atomizer) or an infusion device such as a minipump. A kit may have a sterile access port (for example the container may be an intravenous solution bag or a vial having a stopper pierceable by a hypodermic injection needle). The container may also have a sterile access port (for example the container may be an intravenous solution bag or a vial having a stopper pierceable by a hypodermic injection needle). At least one active agent in the composition is an anti-FXIIa antibody as those described herein.

[0148] Kits may optionally provide additional components such as buffers and interpretive information. Normally, the kit comprises a container and a label or package insert(s) on or associated with the container. In some embodiments, the invention provides articles of manufacture comprising contents of the kits described above.

General Techniques

[0149] The practice of the present invention will employ, unless otherwise indicated, conventional techniques of molecular biology (including recombinant techniques), microbiology, cell biology, biochemistry and immunology, which are within the skill of the art. Such techniques are explained fully in the literature, such as, Molecular Cloning: A Laboratory Manual, second edition (Sambrook, et al., 1989) Cold Spring Harbor Press; Oligonucleotide Synthesis (M. J. Gait, ed., 1984); Methods in Molecular Biology, Humana Press; Cell Biology: A Laboratory Notebook (J. E. Cellis, ed., 1998) Academic Press; Animal Cell Culture (R. I. Freshney, ed., 1987); Introduction to Cell and Tissue Culture (J. P. Mather and P. E. Roberts, 1998) Plenum Press; Cell and Tissue Culture: Laboratory Procedures (A. Doyle, J. B. Griffiths, and D. G. Newell, eds., 1993-8) J. Wiley and Sons; Methods in Enzymology (Academic Press, Inc.); Handbook of Experimental Immunology (D. M. Weir and C. C. Blackwell, eds.); Gene Transfer Vectors for Mammalian Cells (J. M. Miller and M. P. Calos, eds., 1987); Current Protocols in Molecular Biology (F. M. Ausubel, et al., eds., 1987); PCR: The Polymerase Chain Reaction, (Mullis, et al., eds., 1994); Current Protocols in Immunology (J. E. Coligan et al., eds., 1991); Short Protocols in Molecular Biology (Wiley and Sons, 1999); Immunobiology (C. A. Janeway and P. Travers, 1997); Antibodies (P. Finch, 1997); Antibodies: a practical approach (D. Catty., ed., IRL Press, 1988-1989); Monoclonal antibodies: a practical approach (P. Shepherd and C. Dean, eds., Oxford University Press, 2000); Using antibodies: a laboratory manual (E. Harlow and D. Lane (Cold Spring Harbor Laboratory Press, 1999); The Antibodies (M. Zanetti and J. D. Capra, eds., Harwood Academic Publishers, 1995).

[0150] Without further elaboration, it is believed that one skilled in the art can, based on the above description, utilize the present invention to its fullest extent. The following specific embodiments are, therefore, to be construed as merely illustrative, and not limitative of the remainder of the disclosure in any way whatsoever.

EXAMPLES

Example 1: Generation of anti-FXIIa antibodies

[0151] A phage display selection using Dyax's FAB310 phagemid library was performed using the biotinylated beta fragment of FXIIa immobilized on streptavidin coated beads. Fab molecules that bound to the immobilized FXIIa were isolated. The selection produced a selective Fab inhibitor that specifically bound to the catalytic domain of FXIIa with a Ki^{app} of 800pM. The isolated Fab showed no cross-reactivity towards the following proteases tested at a concentration of 1μM: urokinase plasminogen activator, hepatocyte growth factor activator, activated protein C, cathepsin G, C1 s, elastase, factor VIIa, factor Xa, factor XIa, plasmin, thrombin alpha, , trypsin, urokinase, plasma kallikrein, hepatocyte growth factor activator, and urokinase-type plasminogen activator.

[0152] The isolated Fab, M71-F06, was affinity matured via multiple strategies, including heavy chain CDR1/2 shuffling, light chain shuffling, heavy chain CDR3 wobble, and a combination of heavy chain CDR3 wobble and heavy chain CDR1/2 shuffle. The heavy chain CDR1/2 shuffle library was constructed by removing the CDR1/2 region of M71-F06 and replacing it with the full library diversity to make an affinity maturation library with a diversity of $\sim 10^8$. The heavy chain CDR3 was varied at each amino acid position from the parental CDR3 sequence of M71-F06 to create a new library, which was also subsequently combined with the HC CDR1/2 shuffle diversity. A second selection was performed using these libraries. As opposed to the selection performed with immobilized FXIIa, as used for the naive library selection, the affinity maturation libraries were incubated with the biotinylated target in solution at concentrations lower than the parental Ki^{app}. The biotinylated FXIIa and any bound Fabs from the libraries were subsequently pulled down on streptavidin coated beads. Figure 2 presents a schematic of the selection strategy for the identification and generation of anti-

FXIIa antibodies.

[0153] The inhibitory activities of clone M71-F06 against human and mouse FXIIa, as determined by the "in vitro" activity assay described in Example 2 below, are shown in Figure 4. The APTT value of clone M71-F06 (see Example 2 below) as compared to DX-2930 is shown in Figure 5. Results also indicate that clone M71-F06 has no inhibitory activity against activated protein C, CCC Is, Cathepsin G, Elastase, Factor VIIa, Factor Xa, Factor Xia, activated plasma kallikrein, plasmin, thrombin alpha, trypsin, urokinase, HGFA, and uPA, indicating that its inhibitory activity is specific to FXIIa.

[0154] Any isolated Fabs from the selection were screened by ELISA, resulting in 39 unique isolates. The amino acid sequences of the heavy chain variable regions and light chain variable regions are provided above. These clones are expected to have the same antigen-binding activity and specificity as the parent clone with higher binding affinity.

Example 2: Characterization of anti-FXIIa antibodies

Methods:

Activated partial thromboplastin time (APTT) Assay

[0155] Inhibitors (or control dilution buffer = 25 mM HEPES, pH 7.5, 125 mM NaCl) were added to neat plasma in a 1:1 mixture, and pre-equilibrated at 37°C for 5 minutes. 2 x 50 μl of this mix was dispensed to 2 separate KC4 Delta assay cuvettes (with metal ball). After 60 seconds, 50 μl of aPTT reagent (activator, Pacific Hemostasis APTT-XL) was added to the rotating cuvettes, and 180 seconds after aPTT addition (at t = 0 secs), 50 μl of $CaCl_2$ was added. The KC4 Delta instrument recorded the time of coagulation in seconds.

Prothrombin time (PT) Assay

[0156] Inhibitors (or control dilution buffer = 25 mM HEPES, pH 7.5, 125 mM NaCl) were added to neat plasma in a 1:1 mixture, and pre-equilibrated at 37°C for 5 minutes. 2 x 50 μl of this mix was dispensed to 2 separate KC4 Delta assay cuvettes (with metal ball). After 4 minutes, the PT activator (Pacific Hemostasis Thromboplastin D) was added (at t = 0 secs). Coagulation time was automatically recorded by the KC4 Delta instrument.

Purified component inhibition assay

[0157] The purified component inhibition assay is depicted in Figure 3, panel 1. 20 pM FXIIa was incubated with inhibitors at varying concentration for 1 hour at 30°C in a 96-well microplate. 10 nM Prekallikrein was added for 20 minutes at 30°C, followed by a 5 min incubation with 100 nM corn trypsin inhibitor (CTI). Proteolysis was then assessed over time by addition of 10uM final fluorogenic peptide substrate (PFR-AMC), with initial rate of substrate proteolysis (y-axis) plotted against inhibitor concentration (x-axis) and the resulting data fit to the modified Morrison equation (Equation 1) for tight binding inhibitors. All reagents were diluted into Assay Buffer = 20 mM Tris-HCl pH 7.5, 150 mM NaCl, 1 mM EDTA, 0.1% PEG-8000 and 0.1% Triton X-100.

Plasma Inhibition Assay

[0158] The plasma inhibition assay is depicted in Figure 3, panel 2. Pooled normal human plasma was diluted 1:40 in Assay Buffer (above), and inhibitors were added at varying concentrations in a 96-well microplate at room temperature. Contact activation was then initiated by the addition of 25% (2.5% final) ellagic acid, the microplate was mixed by gentle shaking, and allowed to proceed for 2 minutes at room temperature, whereby 100 nM of CTI was added. 10 μl of this mixture was then removed to a replicate microplate containing 80 μl of assay buffer at pre-equilibrated at 30C. This dilution plate was then incubated a further 5 minutes at 30C, and proteolysis of PFR-AMC assessed as above, but with back-calculated concentrations of inhibitor used in the X-axis for curve-fitting to a standard IC_{50} equation (plasma was diluted 1:400 in final assay read).

[0159] One or more of the anti-FXIIa antibodies disclosed herein are also tested in a non-human primate model at two different dosages. The study runs for 30 days after which blood samples are taken to evaluate the pharmacokinetics, effect on APTT, and effect on plasma kallikrein activation via western blot analysis.

Binding preference assay

[0160] To determine the binding preference of the anti-FXIIa antibodies, each antibody was incubated with the FXII zymogen other related proteases of the contact/coagulation system. The binding affinity was then assessed by surface

plasmon resonance (SPR) analysis using a Biacore biosensor. See Figure 3, panel 3.

Flow model/capillary occlusion assay

[0161] The antibodies are tested in an *ex vivo* flow model in which human blood supplemented with the antibody is passed at different flow rates through collagen coated capillary tubes. Platelet and fibrin deposition are assessed by fluorescence. Platelet and fibrin deposition is inhibited in this system by antibodies that bind zymogen factor XI or factor XII. In a typical experiment, the antibodies are allowed to bind to their targets before blood flow is initiated.

Mouse thrombosis model

[0162] The antibodies is tested in a mouse model of ferric chloride-induced carotid artery thrombosis. The model incorporates different $FeCl_3$ concentrations, starting at the lowest concentration that consistently induces thrombus formation in C57Bl/6 mice (3.5%). If the antibody demonstrates an antithrombotic effect at low $FeCl_3$ concentration, progressively higher concentrations are tested until the antithrombotic effect of the antibody is overwhelmed.

Results:

[0163] The 39 isolate anti-FXIIa antibodies were tested in various *in vitro* activity assays to determine the following properties: Ki apparent, IC_{50}, binding preference, such as binding to the FXII zymogen, cross-reactivity with other related proteases in the contact/coagulation system, cross-reactivity with closely related sequence homologs, effect on plasma kallikrein (pKal) generation, activity in human plasma, partial thromboplastin time (PT), activated partial thromboplastin time (APTT), and lag time to thrombin generation.

[0164] Results of the assays are presented in Table 3. All 39 isolates were found to delay APTT, while having no effect on PT. Each of the 39 affinity matured isolates all demonstrated Ki^{app} increases 10-100 fold over the parental isolate. The antibodies reduced pKal generation as measured by two independent inhibition assays: purified component inhibition assay and plasma inhibition assay.

[0165] According to SPR analysis (Biacore), the antibodies did not bind the FXII zymogen, and showed specificity toward the catalytic domain when tested against full length FXIIa. The antibodies also prevented the activation of FXI to FXIa.

Table 3

Isolate	Plasma Ki ^{app} (nM)	APTT (fold delay)	Ki ^{app} (pM)
M0191-E09	31	3.70	5
M0183-C03	33	3.17	6
M0184-F12	38	3.74	4
M0183-H08	76	3.36	5
M0182-D04	96	3.13	11
M0192-H11	101	3.33	6
M0292-D07	115	3.11	14
M0183-D08	118	2.08	11
M0192-F07	125	3.00	7
M0192-G03	125	3.14	18
M0310-C06	135	2.46	57
M0192-F01	174	3.18	8
M0191-A03	176	2.88	13
M0192-A01	198	3.11	17
M0191-H10	216	3.81	13
M0177-C12	217	3.86	18
M0184-B04	269	3.76	8

(continued)

Isolate	Plasma Ki ^{app} (nM)	APTT (fold delay)	Ki ^{app} (pM)
M0308-H03	275	3.38	18
M0192-D02	279	3.30	5
M0310-G07	295	4.48	22
M0192-F06	304	3.49	9
M0310-A04	316	3.48	15
M0310-F02	319	1.86	106
M0310-B09	331	3.73	12
M0310-G06	353	3.18	7
M0182-H01	359	N/A	N/A
M0178-A08	381	3.26	7
M0184-D01	435	3.32	4
M0177-A06	458	4.08	17
M0191-E04	460	3.59	7
M0191-H09	463	3.61	16
M0192-H04	485	3.51	9
M0192-A03	514	3.34	13
M0310-G08	796	2.76	24
M0192-G05	852	3.38	15
M0183-B12	1004	3.06	11
M0308-F04	1570	3.29	17
M0182-B04	N/A	N/A	N/A
M0310-F04	N/A	N/A	N/A

[0166] The inhibitory activities of clone M0192-H11 against human FXIIa, is shown in Figure 21, and was found to be approximately 4.7 +/-0.6 pM.

[0167] Anti-FXIIa antibodies M192-H11 and M192-E09 were also tested in *in vivo* pharmacokinetic experiments in rats. Groups of rats were injected with 20 mg/kg of either anti-FXIIa antibody M191-E09 or M192-H11. At various days following injection, samples were collected from the rats and assess for concentration of the anti-FXIIa antibody and pharmacokinetic parameters (Figure 6 and Table 4).

Table 4: Pharmacokinetic Characteristics of Antibodies M192-H11 and M191-E09:

Exemplary Antibody	Cmax (μg/mL)	AUClast (hr*μg/mL)	Cl (ml/hr/kg)	Vss (mL/kg)	$t_{1/2}$ (hr)
M192-H11	360	18375	0.92	242	220
M191-E09	269	7612	2.28	489	218

Example 3: Development and Characterization of Anti-FXIIa Antibody 559C-X211-A01

[0168] 559C-X211-A01 is an affinity matured, partially germlined, version of the previously described parent antibody 559C-M71-F06. The amino acid sequences of the heavy chain variable region and light chain variable region of this antibody are shown below:

559C-X211-A01_HV (CDRs underlined and in boldface)

EVQLLESGGGLVQPGGSLRLSCAASGFTFS **QYVMH** WVRQAPGKGLEWVS **SIWPSGGHTRYADSVKG** RFTISRDNSKNTLYLQMNSLRAEDTAVYYCTR **QRYRGPKYYYYMDV** WGKGTTVTVSS (SEQ ID NO:125)

559C-X211-A01_LV (CDRs underlined and in boldface)

DIVMTQSPLSLPVTPGEPASISC **RSSQSLLHSNGYNYLD** WYLQKPGQSPQLLIY **LGSNRAS** GVPDRFSGSGSGTDFTLKISRVEAEDVGVYYC **MQALQTPWT** FGQGTKVEIK (SEQ ID NO:126)

[0169] 559C-X211-A01 was tested in various *in vitro* activity assays to determine the following properties; K_i apparent, IC_{50}, binding to the FXII zymogen, cross-reactivity with other related proteases in the contact/coagulation system, cross-reactivity with closely related sequence homologs, effect on plasma kallikrein (pKal) generation, and activity in human plasma. Partial thromboplastin time (PT), and activated partial thromboplastin time (APTT) in mouse plasma has also been tested *in vitro.*

Cross-reactivity Assay

[0170] 559C-X211-A01 showed no cross-reactivity towards the following proteases tested at 1μM: urokinase plasminogen activator, human growth factor activator, activated protein C, cathepsin G, C1s, elastase, factor VIIa, factor Xa, factor XIa, plasmin, thrombin alpha, trypsin, urokinase, and plasma kallikrein.

Activated Partial Thromboplastin Time (APTT) and Prothrombin time Assays

[0171] Inhibitors (or control dilution buffer = 25 mM HEPES, pH 7.5, 125 mM NaCl) were added to both mouse, and human, neat plasma in a 1:1 mixture, and pre-equilibrated at 37C for 5 minutes. 2 x 50 μl of this mix was dispensed to 2 separate KC4 Delta assay cuvettes (with metal ball). After 60 seconds, 50 μl of aPTT reagent (activator, Pacific Hemostasis APTT-XL) was added to the rotating cuvettes, and 180 seconds after this (at t = 0 secs) 50 μl of CaCl2 was added. The KC4 Delta instrument recorded the time of coagulation in seconds.

[0172] As above, except 4 minutes after the 2 x 50 μl of inhibitor/plasma mix was dispensed to 2 separate KC4 Delta assay cuvettes, the PT activator (Pacific Hemostasis Thromboplastin D) was added at t = 0 secs. Coagulation time was automatically recorded by the KC4 Delta instrument.

[0173] 559C-X211-A01 delays APTT in mouse plasma, while having no effect on PT. Figure 7. This data supports species cross-reactivity with mouse factor XII.

Purified component inhibition assay:

[0174] 20 pM FXIIa is incubated with inhibitors at varying concentration for 1 hour at 30°C in a 96-well microplate. 10 nM Prekallikrein is then added for 20 minutes at 30°C, followed by a 5 min incubation with 100 nM corn trypsin inhibitor (CTI). Proteolysis is then assessed over time by addition of 10uM final fluorogenic peptide substrate (PFR-AMC), with initial rate of substrate proteolysis (y-axis) plotted against inhibitor concentration (x-axis) and the resulting data fit to the modified Morrison equation for tight binding inhibitors. All reagents were diluted into Assay Buffer = 20 mM Tris-HCl pH 7.5, 150 mM NaCl, 1 mM EDTA, 0.1% PEG-8000 and 0.1% Triton X-100

Plasma Inhibition Assay:

[0175] Pooled normal human plasma is diluted 1:40 in Assay Buffer (above), and inhibitors are added at varying concentration in a 96-well microplate at room temperature. Contact activation is then initiated by the addition of 25% (2.5% final) APTT-XL reagent (dilute ellagic acid; Pacific Haemostasis), the microplate mixed by gentle shaking, and allowed to proceed for 2 minutes at room temperature, whereby 100 nM of CTI is added. 10 ul of this mixture is then removed to a replicate microplate containing 80ul of assay buffer at pre-equilibrated at 30C. This dilution plate is then incubated a further 5 minutes at 30C, and proteolysis of PFR-AMC assessed as above, but with back-calculated concentrations of inhibitor used in the X-axis for curve-fitting to a standard IC50 equation and/or modified Morrison equation (plasma is diluted 1:400 in final assay read).

[0176] The activities of 559C-X211-A01 observed in the above-noted assays are summarized in Table 5 below:

Table 5: Activities of 559C-X211-A01

Isolate	Plasma IC50 (nM)	APTT (fold delay) using 1uM X211-A01 in human plasma	APTT (fold delay) using 1uM X211-A01 in mouse plasma	Ki ^{app} (pM)
559C-X211-A01	289	3.4	2.8	5

[0177] According to SPR analysis, this antibody binds to FXIIa and does not bind the FXII zymogen. Enzyme inhibition experiments with FXIIa-beta show specific targeting to the catalytic domain.

[0178] 559C-X211-A01 is expected to reduce fibrin generation and deposition, to prolong the time it takes for blood to occlude a collagen-coated capillary tube in a flow model/capillary occlusion assay, and /or to reduce platelet aggregation and the incidence of arterial inclusion. 559C-211-A01 is expected to reduce the incidence of thrombus formation in both a mouse model of thrombosis and a non-human primate model of thrombosis.

Example *4:In vivo* Thrombosis Studies Using DX-4012

Pharmacokinetic studies

[0179] The anticoagulant effect of a single dose of DX-4012 was assessed in healthy baboons as a pharmacodynamic marker of the presence of the antibody in the circulation. The animals were mildly sedated, and blood was drawn from the antecubital vein for baseline coagulation values. The antibody was then administered, at a saturating dose. A single dose of the antibody was delivered intravenously, and blood samples were drawn at various time points as long as anticoagulation can be verified in the APTT assay relative to baseline. Plasma samples were also frozen for additional testing.

[0180] Following completion of the pharmacokinetic studies and determination of the effective half-life of the antibody in baboons, pharmacodynamic thrombosis and hemostasis studies were pursued.

Pharmacodynamic and thrombosis studies

[0181] Acute thrombosis and hemostasis experiments were performed before and during anticoagulation to determine the efficacy and safety of the antibody at a single saturating dose compared to a positive and negative control. The thrombosis experiments were performed in trained baboons by deploying a vascular graft segment into a chronic exteriorized femoral AV shunt. Each day of the study, 1 mL of citrated blood was taken from the loop of the shunt at various time points for serial bleeding time, volume measurements, and anticoagulation assays. Sample times include pre drug (on the day the animal received enoxaparin or DX-4012 prior to administration of enoxaparin or DX-4012), pre study (5 minutes prior to the start of the study), and post study (5 minutes prior to the end of the study, when the shunt loop was removed). Prior to the thrombosis and hemostasis experiments, the baboons received ^{111}In-radiolabeled autologous platelets and ^{125}I-radiolabeled fibrinogen. The antithrombotic activity of DX-4012 was compared to that of enoxaparin (low molecular weight heparin).

[0182] After baseline blood samples were drawn, the thrombosis experiments were initiated. The chronic AV shunt was temporally (60 min) extended using silicon tubing, and a short collagen ePTFE graft segment or tissue factor-coated ePTFE vascular graft segment (20 mm) was deployed within the external AV loop (Figure 10). A thrombus formed in the graft (head thrombus) and extended on the distal side (thrombus tail). Accumulation of radiolabeled platelets in the graft was monitored in real time using gamma camera imaging for 60 minutes and calculated as the number of platelets residing in the graft at 5 minute intervals. After 60 min of graft perfusion, the graft was removed, the shunt reconnected, and the amount of deposited fibrin in the graft by 60 min (end-point) was determined after decay of ^{111}In (30+ days - approximately 10 half-lives later). Fibrin and platelet numbers represent the size of the thrombus.

Results

[0183] APTT and PT assays were performed as described in Examples 2 and 3. APTT was measured using citrated plasma, SynthA Sil® (Instrumentation Laboratories) reagent, and a KC-4 coagulometer. PT was measured using citrated plasma, Dade® Innovin® reagent (Siemens), and a KC-4 coagulometer. Both APTT and PT were measured immediately following blood draw and centrifugation of the blood sample to generate platelet-poor plasma. At 1 hour post administration of DX-4012, the measured fold change in APTT was 2.2 fold (>2.2 fold expected). At 24-48 hrs, the measured fold change in APTT was 1.8 fold (1.8-2.0 fold expected); and at 1 week, the measured fold change in APTT was 1.3 fold

(1.3-1.5 fold expected). There were no observed changes to PT.

Collagen Graft

[0184] For the thrombosis studies, the animals received 10 mg/kg of antibody DX-4012, and the studies were performed at 1 hr, 24 hr, 48 hr, 168 hr, and 192 hrs following antibody administration. For analysis, data from the 24 and 48 hr time points were grouped together, and data from the 168 and 192 hr time points were grouped together. Control animals formed a collagen-initiated baseline thrombus head as expected. The thrombus head was significantly reduced in animals that received enoxaparin. At time points up to 48 hrs post antibody administration, the thrombus head size was reduced relative to the control animals and was comparable to that of animals that received enoxaparin. At one week following antibody administration (168-192 hrs), the thrombus head size was comparable to that of control animals (Figure 11A). At time points up to 48 hrs post antibody administration, the thrombus tail was nearly abolished and comparable to that of animals that received enoxaparin. At one week following antibody administration (168-192 hrs), the thrombus tail remained reduced, but to a lesser extent than post-enoxaparin treatment (Figure 11B).

[0185] In another experiment, at time points up to 48 hrs post antibody administration, the thrombus head size was reduced relative to the control animals and was comparable to that of animals that received enoxaparin. At one week following antibody administration (168-192 hrs), the thrombus head size was still reduced but to a lesser extent than post-enoxaparin treatment (Figure 12). Figure 13 presents thrombus formation (total thrombus), indicating that thrombus formation was comparable at 1 hr, 24 hr and 48 hrs post antibody administration. The thrombus growth rate (platelet deposition at 5 minute intervals) is shown in Figure 14. Control thrombi had increasing growth rate in the first 30 minutes before reaching a plateau. The growth rate after enoxaparin treatment reached a lower plateau at 20 minutes, began to decline after 40 minutes, and lysed at 60 minutes (negative growth rate). Within the first 48 hrs after DX-4012 administration, the growth rate plateaued at 15-20 minutes and had a reduced rate of growth. One week after antibody administration, thrombi were between the early time points (1 hr and 24-48 hrs) and the control.

[0186] Overall, administration of 10 mg/kg DX-4012 reduced the size of the thrombus formed by collagen initiation, the effects of which were seen on both the thrombus head size and the thrombus tail. The reduction in thrombus size was observed up to 48 hrs post antibody administration and was comparable to enoxaparin treatment (1 mg/kg). After one week, the head thrombus returned to baseline in animals that received the antibody, but thrombus tail size remained reduced.

Tissue Factor Graft

[0187] In general, thrombi sizes tend to vary more in tissue factor-coated shunts compared to collagen-coated shunts. Wide variation was observed in the described experiments (7.4, 14.0 and 2.3 billion platelets). Control animals formed a tissue factor-initiated baseline thrombus head as expected. The thrombus head was significantly reduced in animals that received enoxaparin. At time points up to 48 hrs post antibody administration, the thrombus head size was comparable to that of the control animals (Figure 15A). At one week following antibody administration (168-192 hrs), the thrombus head size varied greatly. Enoxaparin treatment nearly abolished tissue factor-initiated thrombus tail formation. There was no significant thrombus tail reduction in animals that received antibody treatment, though the data suggested that there was a slight trend toward a smaller thrombus tail (Figure 15B).

[0188] In another experiment, enoxaparin treatment resulted in a reduction in the thrombus size by >50%, largely due to nearly abolishing the thrombus tail. There was no significant thrombus tail reduction in animals that received antibody treatment, though the data suggested that there was a slight trend toward a smaller thrombus tail (Figure 16). Figure 17 presents thrombus formation (total thrombus). Performing RM ANOVA analysis of the data indicated there was a reduction in the total thrombus size at 1 hr following antibody treatment as compared to the control. The thrombus growth rate (platelet deposition at 5 minute intervals) is shown in Figure 18. Control thrombi had increasing growth rate in the first 30 minutes before reaching a plateau. The growth rate after enoxaparin treatment reached a plateau at 20 minutes, began to decline after 40 minutes, and lysed at 60 minutes (negative growth rate). Within the first 48 hrs after DX-4012 administration, the thrombus growth rate was similar to the observed rate in the control animal (Figure 18).

[0189] Overall, administration of 10 mg/kg DX-4012 had no effect on the thrombus head size and little effect on the thrombus tail size in tissue factor-initiated thrombus formation.

Terminal fibrin content and platelet deposition

[0190] At the end of the experiment, the shunts were flushed with saline and images were captured to assess the final platelet count. The loop was also preserved for fibrin content analysis of the thrombus head and tail.

[0191] Terminal thrombus size was reduced in animals that received enoxaparin. Thrombus size was also significantly reduced, as measured by both platelet deposition and fibrin content, in collagen-coated shunts in the animals that

received DX-4012, but was not reduced in tissue factor-coated shunts (Figures 19A and 19B).

Hemostasis studies

[0192] Hemostasis was measured using the FDA-approved Surgicutt™ bleeding time device and protocol. Blood volume was also measured. One bleeding time and bleeding volume measurement was performed per study day. As shown in Figures 22A and 22B, DX-4012 was effective in reducing the bleeding time and bleeding volume as compared with Enoxaparin.

Example 5:Identification of Critical Residues in the Catalytic Domain of FXIIa Based on the Crystal Structure of DX-4012-FXIIa Complex

[0193] A recombinant Fab fragment of DX-4012 was prepared via routine recombinant technology in *E. coli* and purified. The FXIIa was produced and purified via routine methods.

[0194] The DX-4012 Fab fragment and the FXIIa were mixed at various concentrations under suitable conditions allowing of Fab-FXIIa complexes. The complexes were purified using SEC and visualized in the traces shown in Figure 20.

[0195] The Fab-FXIIa complex was kept under various conditions allowing for crystallization. Diffraction analysis was performed on the crystallized complex. The crystal structures (2.6 Å and 2.25 Å) were determined based on the diffraction statistics.

[0196] According to the crystal structures, residues in the C chain of FXIIa that are involved in the interaction with the Fab fragment of DX-4012 were identified: L390, Y391, W392, G393, H394, S395, F396, C397, H412, C413, L414, Q415, D416, R432, N433, V456, Y458, H507, F509, E510, G511, A512, E513, Y515, D557, A558, C559, Q560, G561, D562, S563, 1584, S585, W586, G587, S588, G589, C590, G591, D592, G597.

[0197] In addition, residues in the Fab of DX-4012 that interact with FXIIa were also identified based on the crystal structure, including T28, S30, Q31, W52, P53, S54, G55, G56, H57, R59, N74, R100, Y101, R102, G103, P104, K105, Y106, Y107, and Y108 in the heavy chain variable region, and H31, N33, Y35, Y54, L55, N58, and T99 in the light chain variable region.

[0198] These results indicate that the heavy chain of DX-4012 is the main region that interacts with FXIIa and a couple of residues in the LC CDR1 were found to contribute to the interaction.

[0199] The crystal structures also revealed that residue R102 of the anti-FXIIa Fab fragment binds the S1 pocket of FXIIa and interacts with residue D557 via water-mediated interactions. This positions the backbone of residues Fab residues R102-G103-P104 near the catalytic triad of FXIIa but in a catalytically incompetent orientation. P104 may contribute to holding this orientation rigidly, thereby inhibiting the activity of FXIIa.

[0200] Complexes of other anti-FXIIa antibodies and Fab fragments thereof with FXIIa are also formed and co-purified in order to determine additional crystal structures.

SEQUENCE LISTING

[0201]

<110> Dyax Corp.

<120> A MONOCLONAL ANTIBODY INHIBITOR OF FACTOR XIIA

<130> D0617.70084WO00

<140> Not Yet Assigned
<141> 2016-07-21

<150> US 62/316,310
<151> 2016-03-31

<150> US 62/273,657
<151> 2015-12-31

<150> US 62/194,957
<151> 2015-07-21

<160> 128

<170> PatentIn version 3.5

<210> 1
<211> 123
<212> PRT
<213> Artificial Sequence

<220>
<223> Synthetic Polypeptide

<400> 1

```
Glu Val Gln Leu Leu Glu Ser Gly Gly Gly Leu Val Gln Pro Gly Gly
1               5                   10                  15

Ser Leu Arg Leu Ser Cys Ala Ala Ser Gly Phe Thr Phe Ser Trp Tyr
            20                  25                  30

Ser Met His Trp Val Arg Gln Ala Pro Gly Lys Gly Leu Glu Trp Val
        35                  40                  45

Ser Val Ile Tyr Pro Ser Gly Gly Lys Thr Arg Tyr Ala Asp Ser Val
    50                  55                  60

Lys Gly Arg Phe Thr Ile Ser Arg Asp Asn Ser Lys Asn Thr Leu Tyr
65                  70                  75                  80

Leu Gln Met Asn Ser Leu Arg Ala Glu Asp Thr Ala Val Tyr Tyr Cys
                85                  90                  95

Thr Arg Gln Arg Tyr Arg Gly Pro Lys Tyr Tyr Tyr Tyr Met Asp Val
            100                 105                 110

Trp Gly Lys Gly Thr Thr Val Thr Val Ser Ser


                    115                 120
```

<210> 2
<211> 123
<212> PRT
<213> Artificial Sequence

<220>
<223> Synthetic Polypeptide

<400> 2

Glu Val Gln Leu Leu Glu Ser Gly Gly Gly Leu Val Gln Pro Gly Gly
1   5   10   15

Ser Leu Arg Leu Ser Cys Ala Ala Ser Gly Phe Thr Phe Ser Gln Tyr
            20   25   30

Val Met His Trp Val Arg Gln Ala Pro Gly Lys Gly Leu Glu Trp Val
        35   40   45

Ser Ser Ile Trp Pro Ser Gly Gly His Thr Arg Tyr Ala Asp Ser Val
    50   55   60

Lys Gly Arg Phe Thr Ile Ser Arg Asp Asn Ser Lys Asn Thr Leu Tyr
65   70   75   80

Leu Gln Met Asn Ser Leu Arg Ala Glu Asp Thr Ala Val Tyr Tyr Cys
                85   90   95

Thr Arg Gln Arg Tyr Arg Gly Pro Lys Tyr Tyr Tyr Tyr Met Asp Val
            100   105   110

Trp Gly Lys Gly Thr Thr Val Thr Val Ser Ser
        115   120

<210> 3
<211> 123
<212> PRT
<213> Artificial Sequence

<220>
<223> Synthetic Polypeptide

<400> 3

Glu Val Gln Leu Leu Glu Ser Gly Gly Gly Leu Val Gln Pro Gly Gly
1   5   10   15

Ser Leu Arg Leu Ser Cys Ala Ala Ser Gly Phe Thr Phe Ser Trp Tyr
            20   25   30

```
Val Met His Trp Val Arg Gln Ala Pro Gly Lys Gly Leu Glu Trp Val
        35                  40                  45

Ser Gly Ile Trp Pro Ser Gly Gly Arg Thr Lys Tyr Ala Asp Ser Val
    50                  55                  60

Lys Gly Arg Phe Thr Ile Ser Arg Asp Asn Ser Lys Asn Thr Leu Tyr
65                  70                  75                  80

Leu Gln Met Asn Ser Leu Arg Ala Glu Asp Thr Ala Val Tyr Tyr Cys
                85                  90                  95

Thr Arg Gln Arg Tyr Arg Gly Pro Lys Tyr Tyr Tyr Tyr Met Asp Val
            100                 105                 110

Trp Gly Lys Gly Thr Thr Val Thr Val Ser Ser
        115                 120
```

<210> 4
<211> 123
<212> PRT
<213> Artificial Sequence

<220>
<223> Synthetic Polypeptide

<400> 4

```
Glu Val Gln Leu Leu Glu Ser Gly Gly Gly Leu Val Gln Pro Gly Gly
1               5                   10                  15

Ser Leu Arg Leu Ser Cys Ala Ala Ser Gly Phe Thr Phe Ser Asn Tyr
            20                  25                  30

Val Met His Trp Val Arg Gln Ala Pro Gly Lys Gly Leu Glu Trp Val
        35                  40                  45

Ser Ser Ile Trp Pro Ser Gly Gly Lys Thr Lys Tyr Ala Asp Ser Val
    50                  55                  60

Lys Gly Arg Phe Thr Ile Ser Arg Asp Asn Ser Lys Asn Thr Leu Tyr
65                  70                  75                  80

Leu Gln Met Asn Ser Leu Arg Ala Glu Asp Thr Ala Val Tyr Tyr Cys
                85                  90                  95

Thr Arg Gln Arg Tyr Arg Gly Pro Lys Tyr Tyr Tyr Tyr Met Asp Ala
            100                 105                 110

Trp Gly Gln Gly Thr Thr Val Thr Val Ser Ser


                    115                 120
```

<210> 5
<211> 123
<212> PRT
<213> Artificial Sequence

<220>
<223> Synthetic Polypeptide

<400> 5

Glu Val Gln Leu Leu Glu Ser Gly Gly Gly Leu Val Gln Pro Gly Gly
1 5 10 15

Ser Leu Arg Leu Ser Cys Ala Ala Ser Gly Phe Thr Phe Ser Met Tyr
20 25 30

Thr Met Asn Trp Val Arg Gln Ala Pro Gly Lys Gly Leu Glu Trp Val
35 40 45

Ser Arg Ile Tyr Pro Ser Gly Gly Lys Thr Leu Tyr Ala Asp Ser Val
50 55 60

Lys Gly Arg Phe Thr Ile Ser Arg Asp Asn Ser Lys Asn Thr Leu Tyr
65 70 75 80

Leu Gln Met Asn Ser Leu Arg Ala Glu Asp Thr Ala Val Tyr Tyr Cys
85 90 95

Thr Arg Gln Arg Tyr Arg Gly Pro Lys Tyr Tyr Tyr Tyr Met Asp Val
100 105 110

Trp Gly Lys Gly Thr Thr Val Thr Val Ser Ser
115 120

<210> 6
<211> 123
<212> PRT
<213> Artificial Sequence

<220>
<223> Synthetic Polypeptide

<400> 6

Glu Val Gln Leu Leu Glu Ser Gly Gly Gly Leu Val Gln Pro Gly Gly
1 5 10 15

Ser Leu Arg Leu Ser Cys Ala Ala Ser Gly Phe Thr Phe Ser Gln Tyr
20 25 30

Val Met Ser Trp Val Arg Gln Ala Pro Gly Lys Gly Leu Glu Trp Val
35 40 45

Ser Arg Ile Tyr Pro Ser Gly Gly Val Thr Lys Tyr Ala Asp Ser Val
50 55 60

Lys Gly Arg Phe Thr Ile Ser Arg Asp Asn Ser Lys Asn Thr Leu Tyr
65 70 75 80

Leu Gln Met Asn Ser Leu Arg Ala Glu Asp Thr Ala Val Tyr Tyr Cys
85 90 95

Thr Arg Gln Arg Tyr Arg Gly Pro Lys Tyr Tyr Tyr Tyr Met Asp Val
100 105 110

Trp Gly Lys Gly Thr Thr Val Thr Val Ser Ser
115 120

<210> 7
<211> 123
<212> PRT
<213> Artificial Sequence

<220>
<223> Synthetic Polypeptide

<400> 7

Glu Val Gln Leu Leu Glu Ser Gly Gly Gly Leu Val Gln Pro Gly Gly
1   5   10   15

Ser Leu Arg Leu Ser Cys Ala Ala Ser Gly Phe Thr Phe Ser Trp Tyr
            20   25   30

Asn Met His Trp Val Arg Gln Ala Pro Gly Lys Gly Leu Glu Trp Val
        35   40   45

Ser Tyr Ile Ser Pro Ser Gly Gly Lys Thr Lys Tyr Thr Asp Ser Val
    50   55   60

Lys Gly Arg Phe Thr Ile Ser Arg Asp Asn Ser Lys Asn Thr Leu Tyr
65   70   75   80

Leu Gln Met Asn Ser Leu Arg Ala Glu Asp Thr Ala Val Tyr Tyr Cys
                85   90   95

Thr Arg Gln Arg Tyr Arg Gly Pro Lys Tyr Tyr Tyr Tyr Met Asp Val
            100   105   110

Trp Gly Lys Gly Thr Thr Val Thr Val Ser Ser

                    115   120

<210> 8
<211> 123
<212> PRT
<213> Artificial Sequence

<220>
<223> Synthetic Polypeptide

<400> 8

Glu Val Gln Leu Leu Glu Ser Gly Gly Gly Leu Val Gln Pro Gly Gly
1 5 10 15

Ser Leu Arg Leu Ser Cys Ala Ala Ser Gly Phe Thr Phe Ser Arg Tyr
20 25 30

Ile Met His Trp Val Arg Gln Ala Pro Gly Lys Gly Leu Glu Trp Val
35 40 45

Ser Ser Ile Tyr Pro Ser Gly Gly Val Thr Lys Tyr Ala Asp Ser Val
50 55 60

Lys Gly Arg Phe Thr Ile Ser Arg Asp Asn Ser Lys Asn Thr Leu Tyr
65 70 75 80

Leu Gln Met Asn Ser Leu Arg Ala Glu Asp Thr Ala Val Tyr Tyr Cys
85 90 95

Thr Arg Gln Arg Tyr Arg Gly Pro Lys Tyr Tyr Tyr Tyr Met Asp Val
100 105 110

Trp Gly Lys Gly Thr Thr Val Thr Val Ser Ser
115 120

<210> 9
<211> 123
<212> PRT
<213> Artificial Sequence

<220>
<223> Synthetic Polypeptide

<400> 9

Glu Val Gln Leu Leu Glu Ser Gly Gly Gly Leu Val Gln Pro Gly Gly
1 5 10 15

Ser Leu Arg Leu Ser Cys Ala Ala Ser Gly Phe Thr Phe Ser Arg Tyr
20 25 30

Ile Met Gly Trp Val Arg Gln Ala Pro Gly Lys Gly Leu Glu Trp Val
35 40 45

Ser Ser Ile Tyr Pro Ser Gly Gly Val Thr Arg Tyr Ala Asp Ser Val
50 55 60

Lys Gly Arg Phe Thr Ile Ser Arg Asp Asn Ser Lys Asn Thr Leu Tyr
65 70 75 80

Leu Gln Met Asn Ser Leu Arg Ala Glu Asp Thr Ala Val Tyr Tyr Cys
85 90 95

Thr Arg Gln Arg Tyr Arg Gly Pro Lys Tyr Tyr Tyr Tyr Met Asp Val
100 105 110

Trp Gly Lys Gly Thr Thr Val Thr Val Ser Ser
115 120

<210> 10
<211> 123
<212> PRT
<213> Artificial Sequence

<220>
<223> Synthetic Polypeptide

<400> 10

```
Glu Val Gln Leu Leu Glu Ser Gly Gly Gly Leu Val Gln Pro Gly Gly
1               5                   10                  15

Ser Leu Arg Leu Ser Cys Ala Ala Ser Gly Phe Thr Phe Ser Gln Tyr
            20                  25                  30

Asn Met Val Trp Val Arg Gln Ala Pro Gly Lys Gly Leu Glu Trp Val
        35                  40                  45

Ser Arg Ile Trp Pro Ser Gly Gly Lys Thr Thr Tyr Ala Asp Ser Val
    50                  55                  60

Lys Gly Arg Phe Thr Ile Ser Arg Asp Asn Ser Lys Asn Thr Leu Tyr
65                  70                  75                  80

Leu Gln Met Asn Ser Leu Arg Ala Glu Asp Thr Ala Val Tyr Tyr Cys
                85                  90                  95

Thr Arg Gln Arg Tyr Arg Gly Pro Lys Tyr Tyr Tyr Tyr Met Asp Val
            100                 105                 110

Trp Gly Lys Gly Thr Thr Val Thr Val Ser Ser


                    115                 120
```

<210> 11
<211> 123
<212> PRT
<213> Artificial Sequence

<220>
<223> Synthetic Polypeptide

<400> 11

Glu Val Gln Leu Leu Glu Ser Gly Gly Gly Leu Val Gln Pro Gly Gly
1 5 10 15

Ser Leu Arg Leu Ser Cys Ala Ala Ser Gly Phe Thr Phe Ser Arg Tyr
20 25 30

Val Met Val Trp Val Arg Gln Ala Pro Gly Lys Gly Leu Glu Trp Val
35 40 45

Ser Arg Ile Tyr Pro Ser Gly Gly Met Thr Gln Tyr Ala Asp Ser Val
50 55 60

Lys Gly Arg Phe Thr Ile Ser Arg Asp Asn Ser Lys Asn Thr Leu Tyr
65 70 75 80

Leu Gln Met Asn Ser Leu Arg Ala Glu Asp Thr Ala Val Tyr Tyr Cys
85 90 95

Thr Arg Gln Arg Tyr Arg Gly Pro Lys Tyr Tyr Tyr Tyr Met Asp Val
100 105 110

Trp Gly Lys Gly Thr Thr Val Thr Val Ser Ser
115 120

<210> 12
<211> 123
<212> PRT
<213> Artificial Sequence

<220>
<223> Synthetic Polypeptide

<400> 12

Glu Val Gln Leu Leu Glu Ser Gly Gly Gly Leu Val Gln Pro Gly Gly
1 5 10 15

Ser Leu Arg Leu Ser Cys Ala Ala Ser Gly Phe Thr Phe Ser Trp Tyr
20 25 30

```
Asn Met Ala Trp Val Arg Gln Ala Pro Gly Lys Gly Leu Glu Trp Val
        35                  40                  45

Ser Arg Ile Tyr Pro Ser Gly Gly Met Thr Gln Tyr Ala Asp Ser Val
    50                  55                  60

Lys Gly Arg Phe Thr Ile Ser Arg Asp Asn Ser Lys Asn Thr Leu Tyr
65                  70                  75                  80

Leu Gln Met Asn Ser Leu Arg Ala Glu Asp Thr Ala Val Tyr Tyr Cys
                85                  90                  95

Thr Arg Gln Arg Tyr Arg Gly Pro Lys Tyr Tyr Tyr Tyr Met Asp Val
            100                 105                 110

Trp Gly Lys Gly Thr Thr Val Thr Val Ser Ser
        115                 120
```

<210> 13
<211> 123
<212> PRT
<213> Artificial Sequence

<220>
<223> Synthetic Polypeptide

<400> 13

Glu Val Gln Leu Leu Glu Ser Gly Gly Gly Leu Val Gln Pro Gly Gly
1 5 10 15

Ser Leu Arg Leu Ser Cys Ala Ala Ser Gly Phe Thr Phe Ser Gln Tyr
20 25 30

Ile Met His Trp Val Arg Gln Ala Pro Gly Lys Gly Leu Glu Trp Val
35 40 45

Ser Ser Ile Tyr Pro Ser Gly Gly Asn Thr Lys Tyr Ala Asp Ser Val
50 55 60

Lys Gly Arg Phe Thr Ile Ser Arg Asp Asn Ser Lys Asn Thr Leu Tyr
65 70 75 80

Leu Gln Met Asn Ser Leu Arg Ala Glu Asp Thr Ala Val Tyr Tyr Cys
85 90 95

Thr Arg Gln Arg Tyr Arg Gly Pro Lys Tyr Tyr Tyr Tyr Met Asp Val
100 105 110

Trp Gly Lys Gly Thr Thr Val Thr Val Ser Ser

115 120

<210> 14
<211> 123
<212> PRT
<213> Artificial Sequence

<220>
<223> Synthetic Polypeptide

<400> 14

Glu Val Gln Leu Leu Glu Ser Gly Gly Gly Leu Val Gln Pro Gly Gly
1 5 10 15

Ser Leu Arg Leu Ser Cys Ala Ala Ser Gly Phe Thr Phe Ser His Tyr
20 25 30

Val Met His Trp Val Arg Gln Ala Pro Gly Lys Gly Leu Glu Trp Val
35 40 45

Ser Ser Ile Tyr Pro Ser Gly Gly Leu Thr Lys Tyr Ala Asp Ser Val
50 55 60

Lys Gly Arg Phe Thr Ile Ser Arg Asp Asn Ser Lys Asn Thr Leu Tyr
65 70 75 80

Leu Gln Met Asn Ser Leu Arg Ala Glu Asp Thr Ala Val Tyr Tyr Cys
85 90 95

Thr Arg Gln Arg Tyr Arg Gly Pro Lys Tyr Tyr Tyr Tyr Met Asp Val
100 105 110

Trp Gly Lys Gly Thr Thr Val Thr Val Ser Ser
115 120

<210> 15
<211> 123
<212> PRT
<213> Artificial Sequence

<220>
<223> Synthetic Polypeptide

<400> 15

Glu Val Gln Leu Leu Glu Ser Gly Gly Gly Leu Val Gln Pro Gly Gly
1 5 10 15

Ser Leu Arg Leu Ser Cys Ala Ala Ser Gly Phe Thr Phe Ser Trp Tyr
20 25 30

Thr Met His Trp Val Arg Gln Ala Pro Gly Lys Gly Leu Glu Trp Val
35 40 45

Ser Ser Ile Tyr Pro Ser Gly Gly Phe Thr Arg Tyr Ala Asp Ser Val
50 55 60

Lys Gly Arg Phe Thr Ile Ser Arg Asp Asn Ser Lys Asn Thr Leu Tyr
65 70 75 80

Leu Gln Met Asn Ser Leu Arg Ala Glu Asp Thr Ala Val Tyr Tyr Cys
85 90 95

Thr Arg Gln Arg Tyr Arg Gly Pro Lys Tyr Tyr Tyr Tyr Met Asp Val
100 105 110

Trp Gly Lys Gly Thr Thr Val Thr Val Ser Ser
115 120

<210> 16
<211> 123
<212> PRT
<213> Artificial Sequence

<220>
<223> Synthetic Polypeptide

<400> 16

Glu Val Gln Leu Leu Glu Ser Gly Gly Gly Leu Val Gln Pro Gly Gly
1   5   10   15

Ser Leu Arg Leu Ser Cys Ala Ala Ser Gly Phe Thr Phe Ser Phe Tyr
            20   25   30

His Met His Trp Val Arg Gln Ala Pro Gly Lys Gly Leu Glu Trp Val
        35   40   45

Ser Arg Ile Val Pro Ser Gly Gly Met Thr Arg Tyr Ala Asp Ser Val
    50   55   60

Lys Gly Arg Phe Thr Ile Ser Arg Asp Asn Ser Lys Asn Thr Leu Tyr
65   70   75   80

Leu Gln Met Asn Ser Leu Arg Ala Glu Asp Thr Ala Val Tyr Tyr Cys
                85   90   95

Thr Arg Gln Arg Tyr Arg Gly Pro Lys Tyr Tyr Tyr Tyr Met Asp Val
            100   105   110

Trp Gly Lys Gly Thr Thr Val Thr Val Ser Ser

115 120

<210> 17
<211> 123
<212> PRT
<213> Artificial Sequence

<220>
<223> Synthetic Polypeptide

<400> 17

Glu Val Gln Leu Leu Glu Ser Gly Gly Gly Leu Val Gln Pro Gly Gly
1   5   10   15

Ser Leu Arg Leu Ser Cys Ala Ala Ser Gly Phe Thr Phe Ser Phe Tyr
            20   25   30

Ser Met His Trp Val Arg Gln Ala Pro Gly Lys Gly Leu Glu Trp Val
        35   40   45

Ser Arg Ile Tyr Pro Ser Gly Gly Val Thr Lys Tyr Ala Asp Ser Val
    50   55   60

Lys Gly Arg Phe Thr Ile Ser Arg Asp Asn Ser Lys Asn Thr Leu Tyr
65   70   75   80

Leu Gln Met Asn Ser Leu Arg Ala Glu Asp Thr Ala Val Tyr Tyr Cys
                85   90   95

Thr Arg Gln Arg Tyr Arg Gly Pro Lys Tyr Tyr Tyr Tyr Met Asp Val
            100   105   110

Trp Gly Lys Gly Thr Thr Val Thr Val Ser Ser
        115   120

<210> 18
<211> 123
<212> PRT
<213> Artificial Sequence

<220>
<223> Synthetic Polypeptide

<400> 18

Glu Val Gln Leu Leu Glu Ser Gly Gly Gly Leu Val Gln Pro Gly Gly
1   5   10   15

Ser Leu Arg Leu Ser Cys Ala Ala Ser Gly Phe Thr Phe Ser Gln Tyr
            20   25   30

Val Met His Trp Val Arg Gln Ala Pro Gly Lys Gly Leu Glu Trp Val
35 40 45

Ser Ser Ile Trp Pro Ser Gly Gly Lys Thr Thr Tyr Ala Asp Ser Val
50 55 60

Lys Gly Arg Phe Thr Ile Ser Arg Asp Asn Ser Lys Asn Thr Leu Tyr
65 70 75 80

Leu Gln Met Asn Ser Leu Arg Ala Glu Asp Thr Ala Val Tyr Tyr Cys
85 90 95

Thr Arg Gln Arg Tyr Arg Gly Pro Lys Tyr Tyr Tyr Tyr Met Asp Val
100 105 110

Trp Gly Lys Gly Thr Thr Val Thr Val Ser Ser
115 120

<210> 19
<211> 123
<212> PRT
<213> Artificial Sequence

<220>
<223> Synthetic Polypeptide

<400> 19

```
Glu Val Gln Leu Leu Glu Ser Gly Gly Gly Leu Val Gln Pro Gly Gly
1               5                   10                  15

Ser Leu Arg Leu Ser Cys Ala Ala Ser Gly Phe Thr Phe Ser Gln Tyr
            20                  25                  30

Val Met His Trp Val Arg Gln Ala Pro Gly Lys Gly Leu Glu Trp Val
        35                  40                  45

Ser Ser Ile Trp Pro Ser Gly Gly Phe Thr Lys Tyr Ala Asp Ser Val
    50                  55                  60

Lys Gly Arg Phe Thr Ile Ser Arg Asp Asn Ser Lys Asn Thr Leu Tyr
65                  70                  75                  80

Leu Gln Met Asn Ser Leu Arg Ala Glu Asp Thr Ala Val Tyr Tyr Cys
                85                  90                  95

Thr Arg Gln Arg Tyr Arg Gly Pro Lys Tyr Tyr Tyr Tyr Met Asp Val
            100                 105                 110

Trp Gly Lys Gly Thr Thr Val Thr Val Ser Ser

                    115                 120
```

<210> 20
<211> 123
<212> PRT
<213> Artificial Sequence

<220>
<223> Synthetic Polypeptide

<400> 20

```
Glu Val Gln Leu Leu Glu Ser Gly Gly Gly Leu Val Gln Pro Gly Gly
1               5                   10                  15

Ser Leu Arg Leu Ser Cys Ala Ala Ser Gly Phe Thr Phe Ser Phe Tyr
            20                  25                  30

Asn Met His Trp Val Arg Gln Ala Pro Gly Lys Gly Leu Glu Trp Val
        35                  40                  45

Ser Ser Ile Tyr Pro Ser Gly Gly Val Thr Arg Tyr Ala Asp Ser Val
    50                  55                  60

Lys Gly Arg Phe Thr Ile Ser Arg Asp Asn Ser Lys Asn Thr Leu Tyr
65                  70                  75                  80

Leu Gln Met Asn Ser Leu Arg Ala Glu Asp Thr Ala Val Tyr Tyr Cys
                85                  90                  95

Thr Arg Gln Arg Tyr Arg Gly Pro Lys Tyr Tyr Tyr Tyr Met Asp Val
            100                 105                 110

Trp Gly Lys Gly Thr Thr Val Thr Val Ser Ser
        115                 120
```

<210> 21
<211> 123
<212> PRT
<213> Artificial Sequence

<220>
<223> Synthetic Polypeptide

<400> 21

```
Glu Val Gln Leu Leu Glu Ser Gly Gly Gly Leu Val Gln Pro Gly Gly
1               5                   10                  15

Ser Leu Arg Leu Ser Cys Ala Ala Ser Gly Phe Thr Phe Ser Trp Tyr
            20                  25                  30
```

```
Val Met His Trp Val Arg Gln Ala Pro Gly Lys Gly Leu Glu Trp Val
        35                  40                  45

Ser Ser Ile Tyr Pro Ser Gly Gly Lys Thr Ser Tyr Ala Asp Ser Val
    50                  55                  60

Lys Gly Arg Phe Thr Ile Ser Arg Asp Asn Ser Lys Asn Thr Leu Tyr
65                  70                  75                  80

Leu Gln Met Asn Ser Leu Arg Ala Glu Asp Thr Ala Val Tyr Tyr Cys
                85                  90                  95

Thr Arg Gln Arg Tyr Arg Gly Pro Lys Tyr Tyr Tyr Tyr Met Asp Val
            100                 105                 110

Trp Gly Lys Gly Thr Thr Val Thr Val Ser Ser
        115                 120
```

<210> 22
<211> 123
<212> PRT
<213> Artificial Sequence

<220>
<223> Synthetic Polypeptide

<400> 22

Glu Val Gln Leu Leu Glu Ser Gly Gly Gly Leu Val Gln Pro Gly Gly
1   5   10   15

Ser Leu Arg Leu Ser Cys Ala Ala Ser Gly Phe Thr Phe Ser Pro Tyr
            20   25   30

Ile Met His Trp Val Arg Gln Ala Pro Gly Lys Gly Leu Glu Trp Val
        35   40   45

Ser Val Ile Tyr Pro Ser Gly Ser Lys Thr Asn Tyr Ala Asp Ser Val
    50   55   60

Lys Gly Arg Phe Thr Ile Ser Arg Asp Asn Ser Lys Asn Thr Leu Tyr
65   70   75   80

Leu Gln Met Asn Ser Leu Arg Ala Glu Asp Thr Ala Val Tyr Tyr Cys
                85   90   95

Thr Arg Gln Arg Tyr Arg Gly Pro Lys Tyr Tyr Tyr Tyr Met Asp Val
            100   105   110

Trp Gly Lys Gly Thr Thr Val Thr Val Ser Ser

                    115   120

<210> 23
<211> 123
<212> PRT
<213> Artificial Sequence

<220>
<223> Synthetic Polypeptide

<400> 23

```
Glu Val Gln Leu Leu Glu Ser Gly Gly Gly Leu Val Gln Pro Gly Gly
1               5                   10                  15

Ser Leu Arg Leu Ser Cys Ala Ala Ser Gly Phe Thr Phe Ser Arg Tyr
            20                  25                  30

Thr Met Arg Trp Val Arg Gln Ala Pro Gly Lys Gly Leu Glu Trp Val
        35                  40                  45

Ser Ser Ile Trp Pro Ser Gly Gly Met Thr Arg Tyr Ala Asp Ser Val
    50                  55                  60

Lys Gly Arg Phe Thr Ile Ser Arg Asp Asn Ser Lys Asn Thr Leu Tyr
65                  70                  75                  80

Leu Gln Met Asn Ser Leu Arg Ala Glu Asp Thr Ala Val Tyr Tyr Cys
                85                  90                  95

Thr Arg Gln Arg Tyr Arg Gly Pro Lys Tyr Tyr Tyr Tyr Met Asp Val
            100                 105                 110

Trp Gly Gln Gly Thr Thr Val Thr Val Ser Ser
        115                 120
```

<210> 24
<211> 123
<212> PRT
<213> Artificial Sequence

<220>
<223> Synthetic Polypeptide

<400> 24

```
Glu Val Gln Leu Leu Glu Ser Gly Gly Gly Leu Val Gln Pro Gly Gly
1               5                   10                  15

Ser Leu Arg Leu Ser Cys Ala Ala Ser Gly Phe Thr Phe Ser Gln Tyr
            20                  25                  30
```

Val Met His Trp Val Arg Gln Ala Pro Gly Lys Gly Leu Glu Trp Val
35 40 45

Ser Ser Ile Tyr Pro Ser Gly Gly Leu Thr Arg Tyr Ala Asp Ser Val
50 55 60

Lys Gly Arg Phe Thr Ile Ser Arg Asp Asn Ser Lys Asn Thr Leu Tyr
65 70 75 80

Leu Gln Met Asn Ser Leu Arg Ala Glu Asp Thr Ala Val Tyr Tyr Cys
85 90 95

Thr Arg Gln Arg Tyr Arg Gly Pro Lys Tyr Tyr Tyr Tyr Met Asp Val
100 105 110

Trp Gly Gln Gly Thr Thr Val Thr Val Ser Ser
115 120

<210> 25
<211> 123
<212> PRT
<213> Artificial Sequence

<220>
<223> Synthetic Polypeptide

<400> 25

Glu Val Gln Leu Leu Glu Ser Gly Gly Gly Leu Val Gln Pro Gly Gly
1 5 10 15

Ser Leu Arg Leu Ser Cys Ala Ala Ser Gly Phe Thr Phe Ser Trp Tyr
20 25 30

Ile Met Gly Trp Val Arg Gln Ala Pro Gly Lys Gly Leu Glu Trp Val
35 40 45

Ser Tyr Ile Tyr Pro Ser Gly Gly Asn Thr Arg Tyr Ala Asp Ser Val
50 55 60

Lys Gly Arg Phe Thr Ile Ser Arg Asp Asn Ser Lys Asn Thr Leu Tyr
65 70 75 80

Leu Gln Met Asn Ser Leu Arg Ala Glu Asp Thr Ala Val Tyr Tyr Cys
85 90 95

Thr Arg Gln Arg Tyr Arg Gly Pro Lys Tyr Tyr Tyr Tyr Met Asp Val
100 105 110

Trp Gly Lys Gly Thr Thr Val Thr Val Ser Ser

115 120

<210> 26
<211> 123
<212> PRT
<213> Artificial Sequence

<220>
<223> Synthetic Polypeptide

<400> 26

```
Glu Val Gln Leu Leu Glu Ser Gly Gly Gly Leu Val Gln Pro Gly Gly
1               5                   10                  15

Ser Leu Arg Leu Ser Cys Ala Ala Ser Gly Phe Thr Phe Ser Arg Tyr
            20                  25                  30

Val Met His Trp Val Arg Gln Ala Pro Gly Lys Gly Leu Glu Trp Val
        35                  40                  45

Ser Ser Ile Trp Pro Ser Gly Gly Met Thr Lys Tyr Ala Asp Ser Val
    50                  55                  60

Lys Gly Arg Phe Thr Ile Ser Arg Asp Asn Ser Lys Asn Thr Leu Tyr
65                  70                  75                  80

Leu Gln Met Asn Ser Leu Arg Ala Glu Asp Thr Ala Val Tyr Tyr Cys
                85                  90                  95

Thr Arg Gln Arg Tyr Arg Gly Pro Lys Tyr Tyr Tyr Tyr Met Asp Val
            100                 105                 110

Trp Gly Lys Gly Thr Thr Val Thr Val Ser Ser
        115                 120
```

<210> 27
<211> 123
<212> PRT
<213> Artificial Sequence

<220>
<223> Synthetic Polypeptide

<400> 27

```
Glu Val Gln Leu Leu Glu Ser Gly Gly Gly Leu Val Gln Pro Gly Gly
1               5                   10                  15

Ser Leu Arg Leu Ser Cys Ala Ala Ser Gly Phe Thr Phe Ser Phe Tyr
            20                  25                  30
```

```
Ile Met Gly Trp Val Arg Gln Ala Pro Gly Lys Gly Leu Glu Trp Val
        35                  40                  45

Ser Arg Ile Tyr Pro Ser Gly Gly Ala Thr Gln Tyr Ala Asp Ser Val
    50                  55                  60

Lys Gly Arg Phe Thr Ile Ser Arg Asp Asn Ser Lys Asn Thr Leu Tyr
65                  70                  75                  80

Leu Gln Met Asn Ser Leu Arg Ala Glu Asp Thr Ala Val Tyr Tyr Cys
                85                  90                  95

Thr Arg Gln Arg Tyr Arg Gly Pro Lys Tyr Tyr Tyr Tyr Met Asp Val
            100                 105                 110

Trp Gly Lys Gly Thr Thr Val Thr Val Ser Ser
        115                 120
```

<210> 28
<211> 123
<212> PRT
<213> Artificial Sequence

<220>
<223> Synthetic Polypeptide

<400> 28

Glu Val Gln Leu Leu Glu Ser Gly Gly Gly Leu Val Gln Pro Gly Gly
1 5 10 15

Ser Leu Arg Leu Ser Cys Ala Ala Ser Gly Phe Thr Phe Ser Phe Tyr
20 25 30

Val Met Gly Trp Val Arg Gln Ala Pro Gly Lys Gly Leu Glu Trp Val
35 40 45

Ser Arg Ile Tyr Pro Ser Gly Gly Leu Thr Gln Tyr Ala Asp Ser Val
50 55 60

Lys Gly Arg Phe Thr Ile Ser Arg Asp Asn Ser Lys Asn Thr Leu Tyr
65 70 75 80

Leu Gln Met Asn Ser Leu Arg Ala Glu Asp Thr Ala Val Tyr Tyr Cys
85 90 95

Thr Arg Gln Arg Tyr Arg Gly Pro Lys Tyr Tyr Tyr Tyr Met Asp Val
100 105 110

Trp Gly Lys Gly Thr Thr Val Thr Val Ser Ser

115 120

<210> 29
<211> 123
<212> PRT
<213> Artificial Sequence

<220>
<223> Synthetic Polypeptide

<400> 29

```
Glu Val Gln Leu Leu Glu Ser Gly Gly Gly Leu Val Gln Pro Gly Gly
1               5                   10                  15

Ser Leu Arg Leu Ser Cys Ala Ala Ser Gly Phe Thr Phe Ser Phe Tyr
            20                  25                  30

Ser Met His Trp Val Arg Gln Ala Pro Gly Lys Gly Leu Glu Trp Val
        35                  40                  45

Ser Arg Ile Tyr Pro Ser Gly Gly Ile Thr Ser Tyr Ala Asp Ser Val
    50                  55                  60

Lys Gly Arg Phe Thr Ile Ser Arg Asp Asn Ser Lys Asn Thr Leu Tyr
65                  70                  75                      80

Leu Gln Met Asn Ser Leu Arg Ala Glu Asp Thr Ala Val Tyr Tyr Cys
                85                  90                  95

Thr Arg Gln Arg Tyr Arg Gly Pro Lys Tyr Tyr Tyr Tyr Met Asp Val
            100                 105                 110

Trp Gly Lys Gly Thr Thr Val Thr Val Ser Ser
        115                 120
```

<210> 30
<211> 123
<212> PRT
<213> Artificial Sequence

<220>
<223> Synthetic Polypeptide

<400> 30

```
Glu Val Gln Leu Leu Glu Ser Gly Gly Gly Leu Val Gln Pro Gly Gly
1               5                   10                  15

Ser Leu Arg Leu Ser Cys Ala Ala Ser Gly Phe Thr Phe Ser Met Tyr
            20                  25                  30
```

```
Ile Met His Trp Val Arg Gln Ala Pro Gly Lys Gly Leu Glu Trp Val
        35                  40                  45

Ser Ser Ile Tyr Pro Ser Gly Gly Met Thr Lys Tyr Ala Asp Ser Val
    50                  55                  60

Lys Gly Arg Phe Thr Ile Ser Arg Asp Asn Ser Lys Asn Thr Leu Tyr
65                  70                  75                  80

Leu Gln Met Asn Ser Leu Arg Ala Glu Asp Thr Ala Val Tyr Tyr Cys
                85                  90                  95

Thr Arg Gln Arg Tyr Arg Gly Pro Lys Tyr Tyr Tyr Tyr Met Asp Val
            100                 105                 110

Trp Gly Lys Gly Thr Thr Val Thr Val Ser Ser
        115                 120
```

<210> 31
<211> 123
<212> PRT
<213> Artificial Sequence

<220>
<223> Synthetic Polypeptide

<400> 31

Glu Val Gln Leu Leu Glu Ser Gly Gly Gly Leu Val Gln Pro Gly Gly
1   5   10   15

Ser Leu Arg Leu Ser Cys Ala Ala Ser Gly Phe Thr Phe Ser Met Tyr
            20   25   30

Val Met His Trp Val Arg Gln Ala Pro Gly Lys Gly Leu Glu Trp Val
        35   40   45

Ser Ser Ile Tyr Pro Ser Gly Gly Leu Thr Lys Tyr Ala Asp Ser Val
    50   55   60

Lys Gly Arg Phe Thr Ile Ser Arg Asp Asn Ser Lys Asn Thr Leu Tyr
65   70   75   80

Leu Gln Met Asn Ser Leu Arg Ala Glu Asp Thr Ala Val Tyr Tyr Cys
                85   90   95

Thr Arg Gln Arg Tyr Arg Gly Pro Lys Tyr Tyr Tyr Tyr Met Asp Val
            100   105   110

Trp Gly Lys Gly Thr Thr Val Thr Val Ser Ser

                    115   120

<210> 32
<211> 123
<212> PRT
<213> Artificial Sequence

<220>
<223> Synthetic Polypeptide

<400> 32

```
Glu Val Gln Leu Leu Glu Ser Gly Gly Gly Leu Val Gln Pro Gly Gly
1               5                   10                  15

Ser Leu Arg Leu Ser Cys Ala Ala Ser Gly Phe Thr Phe Ser Gln Tyr
            20                  25                  30

Val Met His Trp Val Arg Gln Ala Pro Gly Lys Gly Leu Glu Trp Val
        35                  40                  45

Ser Arg Ile Tyr Pro Ser Gly Gly Leu Thr Asn Tyr Ala Asp Ser Val
    50                  55                  60

Lys Gly Arg Phe Thr Ile Ser Arg Asp Asn Ser Lys Asn Thr Leu Tyr
65                  70                  75                  80

Leu Gln Met Asn Ser Leu Arg Ala Glu Asp Thr Ala Val Tyr Tyr Cys
                85                  90                  95

Thr Arg Gln Arg Tyr Arg Gly Pro Lys Tyr Tyr Tyr Tyr Met Asp Val
            100                 105                 110

Trp Gly Lys Gly Thr Thr Val Thr Val Ser Ser
        115                 120
```

<210> 33
<211> 123
<212> PRT
<213> Artificial Sequence

<220>
<223> Synthetic Polypeptide

<400> 33

```
Glu Val Gln Leu Leu Glu Ser Gly Gly Gly Leu Val Gln Pro Gly Gly
1               5                   10                  15

Ser Leu Arg Leu Ser Cys Ala Ala Ser Gly Phe Thr Phe Ser Trp Tyr
            20                  25                  30
```

```
Val Met Gln Trp Val Arg Gln Ala Pro Gly Lys Gly Leu Glu Trp Val
        35                  40                  45

Ser Ser Ile Tyr Pro Ser Gly Gly Met Thr Lys Tyr Ala Asp Ser Val
    50                  55                  60

Lys Gly Arg Phe Thr Ile Ser Arg Asp Asn Ser Lys Asn Thr Leu Tyr
65                  70                  75                  80

Leu Gln Met Asn Ser Leu Arg Ala Glu Asp Thr Ala Val Tyr Tyr Cys
                85                  90                  95

Thr Arg Gln Arg Tyr Arg Gly Pro Lys Tyr Tyr Tyr Tyr Met Asp Val
            100                 105                 110

Trp Gly Lys Gly Thr Thr Val Thr Val Ser Ser
        115                 120
```

<210> 34
<211> 123
<212> PRT
<213> Artificial Sequence

<220>
<223> Synthetic Polypeptide

<400> 34

```
Glu Val Gln Leu Leu Glu Ser Gly Gly Gly Leu Val Gln Pro Gly Gly
1               5                   10                  15

Ser Leu Arg Leu Ser Cys Ala Ala Ser Gly Phe Thr Phe Ser Trp Tyr
            20                  25                  30

Ile Met Gly Trp Val Arg Gln Ala Pro Gly Lys Gly Leu Glu Trp Val
        35                  40                  45

Ser Arg Ile Tyr Pro Ser Gly Gly Ser Thr His Tyr Ala Asp Ser Val
    50                  55                  60

Lys Gly Arg Phe Thr Ile Ser Arg Asp Asn Ser Lys Asn Thr Leu Tyr
65                  70                  75                  80

Leu Gln Met Asn Ser Leu Arg Ala Glu Asp Thr Ala Val Tyr Tyr Cys
                85                  90                  95

Thr Arg Gln Arg Tyr Arg Gly Pro Arg Tyr Tyr Tyr Tyr Ile Asp Ala
            100                 105                 110

Trp Gly Gln Gly Thr Thr Val Thr Val Ser Ser


                    115                 120
```

<210> 35
<211> 123
<212> PRT
<213> Artificial Sequence

<220>
<223> Synthetic Polypeptide

<400> 35

Glu Val Gln Leu Leu Glu Ser Gly Gly Gly Leu Val Gln Pro Gly Gly
1   5   10   15

Ser Leu Arg Leu Ser Cys Ala Ala Ser Gly Phe Thr Phe Ser Gln Tyr
            20   25   30

Thr Met Val Trp Val Arg Gln Ala Pro Gly Lys Gly Leu Glu Trp Val
        35   40   45

Ser Arg Ile Tyr Pro Ser Gly Gly Val Thr Gln Tyr Ala Asp Ser Val
    50   55   60

Lys Gly Arg Phe Thr Ile Ser Arg Asp Asn Ser Lys Asn Thr Leu Tyr
65   70   75   80

Leu Gln Met Asn Ser Leu Arg Ala Glu Asp Thr Ala Val Tyr Tyr Cys
                85   90   95

Thr Arg Gln Arg Tyr Arg Gly Pro Lys Tyr Tyr Tyr Tyr Met Asp Val
            100   105   110

Trp Gly Lys Gly Thr Thr Val Thr Val Ser Ser
        115   120

<210> 36
<211> 123
<212> PRT
<213> Artificial Sequence

<220>
<223> Synthetic Polypeptide

<400> 36

Glu Val Gln Leu Leu Glu Ser Gly Gly Gly Leu Val Gln Pro Gly Gly
1   5   10   15

Ser Leu Arg Leu Ser Cys Ala Ala Ser Gly Phe Thr Phe Ser Trp Tyr
            20   25   30

Val Met Tyr Trp Val Arg Gln Ala Pro Gly Lys Gly Leu Glu Trp Val
35 40 45

Ser Arg Ile Tyr Pro Ser Gly Gly Ile Thr His Tyr Ala Asp Ser Val
50 55 60

Lys Gly Arg Phe Thr Ile Ser Arg Asp Asn Ser Lys Asn Thr Leu Tyr
65 70 75 80

Leu Gln Met Asn Ser Leu Arg Ala Glu Asp Thr Ala Val Tyr Tyr Cys
85 90 95

Thr Arg Gln Arg Tyr Arg Gly Pro Lys Tyr Tyr Tyr Tyr Met Asp Val
100 105 110

Trp Gly Lys Gly Thr Thr Val Thr Val Ser Ser
115 120

<210> 37
<211> 123
<212> PRT
<213> Artificial Sequence

<220>
<223> Synthetic Polypeptide

<400> 37

```
Glu Val Gln Leu Leu Glu Ser Gly Gly Gly Leu Val Gln Pro Gly Gly
1               5                   10                  15

Ser Leu Arg Leu Ser Cys Ala Ala Ser Gly Phe Thr Phe Ser Phe Tyr
            20                  25                  30

Val Met Leu Trp Val Arg Gln Ala Pro Gly Lys Gly Leu Glu Trp Val
        35                  40                  45

Ser Ser Ile Trp Pro Ser Gly Gly Val Thr Lys Tyr Ala Asp Ser Val
    50                  55                  60

Lys Gly Arg Phe Thr Ile Ser Arg Asp Asn Ser Lys Asn Thr Leu Tyr
65                  70                  75                  80

Leu Gln Met Asn Ser Leu Arg Ala Glu Asp Thr Ala Val Tyr Tyr Cys
                85                  90                  95

Thr Arg Gln Arg Tyr Arg Gly Pro Lys Tyr Tyr Tyr Tyr Met Asp Val
            100                 105                 110

Trp Gly Lys Gly Thr Thr Val Thr Val Ser Ser

                    115                 120
```

<210> 38
<211> 123
<212> PRT
<213> Artificial Sequence

<220>
<223> Synthetic Polypeptide

<400> 38

Glu Val Gln Leu Leu Glu Ser Gly Gly Gly Leu Val Gln Pro Gly Gly
1 5 10 15

Ser Leu Arg Leu Ser Cys Ala Ala Ser Gly Phe Thr Phe Ser Trp Tyr
20 25 30

Val Met Gln Trp Val Arg Gln Ala Pro Gly Lys Gly Leu Glu Trp Val
35 40 45

Ser Tyr Ile Tyr Pro Ser Gly Gly His Thr Lys Tyr Ala Asp Ser Val
50 55 60

Lys Gly Arg Phe Thr Ile Ser Arg Asp Asn Ser Lys Asn Thr Leu Tyr
65 70 75 80

Leu Gln Met Asn Ser Leu Arg Ala Glu Asp Thr Ala Val Tyr Tyr Cys
85 90 95

Thr Arg Gln Arg Tyr Arg Gly Pro Lys Tyr Tyr Tyr Tyr Met Asp Val
100 105 110

Trp Gly Lys Gly Thr Thr Val Thr Val Ser Ser
115 120

<210> 39
<211> 123
<212> PRT
<213> Artificial Sequence

<220>
<223> Synthetic Polypeptide

<400> 39

Glu Val Gln Leu Leu Glu Ser Gly Gly Gly Leu Val Gln Pro Gly Gly
1 5 10 15

Ser Leu Arg Leu Ser Cys Ala Ala Ser Gly Phe Thr Phe Ser Arg Tyr
20 25 30

```
Ser Met Asn Trp Val Arg Gln Ala Pro Gly Lys Gly Leu Glu Trp Val
        35                  40                  45

Ser Gly Ile Tyr Pro Ser Gly Gly Lys Thr Lys Tyr Ala Asp Ser Val
    50                  55                  60

Lys Gly Arg Phe Thr Ile Ser Arg Asp Asn Ser Lys Asn Thr Leu Tyr
65                  70                  75                  80

Leu Gln Met Asn Ser Leu Arg Ala Glu Asp Thr Ala Val Tyr Tyr Cys
                85                  90                  95

Thr Arg Gln Arg Tyr Arg Gly Pro Lys Tyr Tyr Tyr Tyr Met Asp Val
            100                 105                 110

Trp Gly Lys Gly Thr Thr Val Thr Val Ser Ser
        115                 120
```

<210> 40
<211> 112
<212> PRT
<213> Artificial Sequence

<220>
<223> Synthetic Polypeptide

<400> 40

```
Asp Ile Gln Met Thr Gln Ser Pro Leu Ser Leu Pro Val Thr Pro Gly
1               5                   10                  15

Glu Pro Ala Ser Ile Ser Cys Arg Ser Ser Gln Ser Leu Leu His Ser
            20                  25                  30

Asn Gly Tyr Asn Tyr Leu Asp Trp Tyr Leu Gln Lys Pro Gly Gln Ser
        35                  40                  45

Pro Gln Leu Leu Ile Tyr Leu Gly Ser Asn Arg Ala Ser Gly Val Pro
    50                  55                  60

Asp Arg Phe Ser Gly Ser Gly Ser Gly Thr Asp Phe Thr Leu Lys Ile
65                  70                  75                  80

Ser Arg Val Glu Ala Glu Asp Val Gly Val Tyr Tyr Cys Met Gln Ala
                85                  90                  95

Leu Gln Thr Pro Trp Thr Phe Gly Gln Gly Thr Lys Val Glu Ile Lys
            100                 105                 110
```

<210> 41
<211> 5
<212> PRT
<213> Artificial Sequence

<220>
<223> Synthetic Polypeptide

<400> 41

```
Trp Tyr Ser Met His
1               5
```

<210> 42
<211> 5
<212> PRT
<213> Artificial Sequence

<220>
<223> Synthetic Polypeptide

<400> 42

```
Gln Tyr Val Met His
1               5
```

<210> 43
<211> 5
<212> PRT
<213> Artificial Sequence

<220>
<223> Synthetic Polypeptide

<400> 43

```
Trp Tyr Val Met His
1               5
```

<210> 44
<211> 5
<212> PRT
<213> Artificial Sequence

<220>
<223> Synthetic Polypeptide

<400> 44

```
Asn Tyr Val Met His
1               5
```

<210> 45
<211> 5
<212> PRT
<213> Artificial Sequence

<220>

<223> Synthetic Polypeptide

<400> 45

Met Tyr Thr Met Asn
1 5

<210> 46
<211> 5
<212> PRT
<213> Artificial Sequence

<220>
<223> Synthetic Polypeptide

<400> 46

Gln Tyr Val Met Ser
1 5

<210> 47
<211> 5
<212> PRT
<213> Artificial Sequence

<220>
<223> Synthetic Polypeptide

<400> 47

Trp Tyr Asn Met His
1 5

<210> 48
<211> 5
<212> PRT
<213> Artificial Sequence

<220>
<223> Synthetic Polypeptide

<400> 48

Arg Tyr Ile Met His
1 5

<210> 49
<211> 5
<212> PRT
<213> Artificial Sequence

<220>
<223> Synthetic Polypeptide

<400> 49

Arg Tyr Ile Met Gly
1 5

<210> 50
<211> 5
<212> PRT
<213> Artificial Sequence

<220>
<223> Synthetic Polypeptide

<400> 50

Gln Tyr Asn Met Val
1 5

<210> 51
<211> 5
<212> PRT
<213> Artificial Sequence

<220>
<223> Synthetic Polypeptide

<400> 51

Arg Tyr Val Met Val
1 5

<210> 52
<211> 5
<212> PRT
<213> Artificial Sequence

<220>
<223> Synthetic Polypeptide

<400> 52

Trp Tyr Asn Met Ala
1 5

<210> 53
<211> 5
<212> PRT
<213> Artificial Sequence

<220>
<223> Synthetic Polypeptide

<400> 53

Gln Tyr Ile Met His
1 5

<210> 54
<211> 5
<212> PRT
<213> Artificial Sequence

<220>

<223> Synthetic Polypeptide

<400> 54

His Tyr Val Met His
1 5

<210> 55
<211> 5
<212> PRT
<213> Artificial Sequence

<220>
<223> Synthetic Polypeptide

<400> 55

Trp Tyr Thr Met His
1 5

<210> 56
<211> 5
<212> PRT
<213> Artificial Sequence

<220>
<223> Synthetic Polypeptide

<400> 56

Phe Tyr His Met His
1 5

<210> 57
<211> 5
<212> PRT
<213> Artificial Sequence

<220>
<223> Synthetic Polypeptide

<400> 57

Phe Tyr Ser Met His
1 5

<210> 58
<211> 5
<212> PRT
<213> Artificial Sequence

<220>
<223> Synthetic Polypeptide

<400> 58

Gln Tyr Val Met His
1 5

<210> 59
<211> 5
<212> PRT
<213> Artificial Sequence

<220>
<223> Synthetic Polypeptide

<400> 59

Phe Tyr Asn Met His
1 5

<210> 60
<211> 5
<212> PRT
<213> Artificial Sequence

<220>
<223> Synthetic Polypeptide

<400> 60

Pro Tyr Ile Met His
1 5

<210> 61
<211> 5
<212> PRT
<213> Artificial Sequence

<220>
<223> Synthetic Polypeptide

<400> 61

Arg Tyr Thr Met Arg
1 5

<210> 62
<211> 5
<212> PRT
<213> Artificial Sequence

<220>
<223> Synthetic Polypeptide

<400> 62

Trp Tyr Ile Met Gly
1 5

<210> 63
<211> 5
<212> PRT
<213> Artificial Sequence

<220>

<223> Synthetic Polypeptide

<400> 63

Arg Tyr Val Met His
1 5

<210> 64
<211> 5
<212> PRT
<213> Artificial Sequence

<220>
<223> Synthetic Polypeptide

<400> 64

Phe Tyr Ile Met Gly
1 5

<210> 65
<211> 5
<212> PRT
<213> Artificial Sequence

<220>
<223> Synthetic Polypeptide

<400> 65

Phe Tyr Val Met Gly
1 5

<210> 66
<211> 5
<212> PRT
<213> Artificial Sequence

<220>
<223> Synthetic Polypeptide

<400> 66

Phe Tyr Ser Met His
1 5

<210> 67
<211> 5
<212> PRT
<213> Artificial Sequence

<220>
<223> Synthetic Polypeptide

<400> 67

Met Tyr Ile Met His
1 5

<210> 68
<211> 5
<212> PRT
<213> Artificial Sequence

<220>
<223> Synthetic Polypeptide

<400> 68

```
Met Tyr Val Met His
1               5
```

<210> 69
<211> 5
<212> PRT
<213> Artificial Sequence

<220>
<223> Synthetic Polypeptide

<400> 69

```
Trp Tyr Val Met Gln
1               5
```

<210> 70
<211> 5
<212> PRT
<213> Artificial Sequence

<220>
<223> Synthetic Polypeptide

<400> 70

```
Gln Tyr Thr Met Val
1               5
```

<210> 71
<211> 5
<212> PRT
<213> Artificial Sequence

<220>
<223> Synthetic Polypeptide

<400> 71

```
Trp Tyr Val Met Tyr
1               5
```

<210> 72
<211> 5
<212> PRT
<213> Artificial Sequence

<220>

<223> Synthetic Polypeptide

<400> 72

Phe Tyr Val Met Leu
1 5

<210> 73
<211> 5
<212> PRT
<213> Artificial Sequence

<220>
<223> Synthetic Polypeptide

<400> 73

Arg Tyr Ser Met Asn
1 5

<210> 74
<211> 17
<212> PRT
<213> Artificial Sequence

<220>
<223> Synthetic Polypeptide

<400> 74

Val Ile Tyr Pro Ser Gly Gly Lys Thr Arg Tyr Ala Asp Ser Val Lys
1 5 10 15

Gly

<210> 75
<211> 17
<212> PRT
<213> Artificial Sequence

<220>
<223> Synthetic Polypeptide

<400> 75

Ser Ile Trp Pro Ser Gly Gly His Thr Arg Tyr Ala Asp Ser Val Lys
1 5 10 15

Gly

<210> 76
<211> 17
<212> PRT
<213> Artificial Sequence

<220>

<223> Synthetic Polypeptide

<400> 76

```
Gly Ile Trp Pro Ser Gly Gly Arg Thr Lys Tyr Ala Asp Ser Val Lys
1               5                   10                  15

Gly
```

<210> 77
<211> 17
<212> PRT
<213> Artificial Sequence

<220>
<223> Synthetic Polypeptide

<400> 77

```
Ser Ile Trp Pro Ser Gly Gly Lys Thr Lys Tyr Ala Asp Ser Val Lys
1               5                   10                  15

Gly
```

<210> 78
<211> 17
<212> PRT
<213> Artificial Sequence

<220>
<223> Synthetic Polypeptide

<400> 78

```
Arg Ile Tyr Pro Ser Gly Gly Lys Thr Leu Tyr Ala Asp Ser Val Lys
1               5                   10                  15

Gly
```

<210> 79
<211> 17
<212> PRT
<213> Artificial Sequence

<220>
<223> Synthetic Polypeptide

<400> 79

```
Arg Ile Tyr Pro Ser Gly Gly Val Thr Lys Tyr Ala Asp Ser Val Lys
1               5                   10                  15

Gly
```

<210> 80
<211> 17
<212> PRT
<213> Artificial Sequence

<220>
<223> Synthetic Polypeptide

<400> 80

```
Tyr Ile Ser Pro Ser Gly Gly Lys Thr Lys Tyr Thr Asp Ser Val Lys
1               5                   10                  15


Gly
```

<210> 81
<211> 17
<212> PRT
<213> Artificial Sequence

<220>
<223> Synthetic Polypeptide

<400> 81

```
Ser Ile Tyr Pro Ser Gly Gly Val Thr Lys Tyr Ala Asp Ser Val Lys
1               5                   10                  15


Gly
```

<210> 82
<211> 17
<212> PRT
<213> Artificial Sequence

<220>
<223> Synthetic Polypeptide

<400> 82

```
Ser Ile Tyr Pro Ser Gly Gly Val Thr Arg Tyr Ala Asp Ser Val Lys
1               5                   10                  15


Gly
```

<210> 83
<211> 17
<212> PRT
<213> Artificial Sequence

<220>
<223> Synthetic Polypeptide

<400> 83

Arg Ile Trp Pro Ser Gly Gly Lys Thr Thr Tyr Ala Asp Ser Val Lys
1 5 10 15

Gly

<210> 84
<211> 17
<212> PRT
<213> Artificial Sequence

<220>
<223> Synthetic Polypeptide

<400> 84

Arg Ile Tyr Pro Ser Gly Gly Met Thr Gln Tyr Ala Asp Ser Val Lys
1 5 10 15

Gly

<210> 85
<211> 17
<212> PRT
<213> Artificial Sequence

<220>
<223> Synthetic Polypeptide

<400> 85

Ser Ile Tyr Pro Ser Gly Gly Asn Thr Lys Tyr Ala Asp Ser Val Lys
1 5 10 15

Gly

<210> 86
<211> 17
<212> PRT
<213> Artificial Sequence

<220>
<223> Synthetic Polypeptide

<400> 86

Ser Ile Tyr Pro Ser Gly Gly Leu Thr Lys Tyr Ala Asp Ser Val Lys
1 5 10 15

Gly

<210> 87
<211> 17
<212> PRT
<213> Artificial Sequence

<220>
<223> Synthetic Polypeptide

<400> 87

Ser Ile Tyr Pro Ser Gly Gly Phe Thr Arg Tyr Ala Asp Ser Val Lys
1 5 10 15

Gly

<210> 88
<211> 17
<212> PRT
<213> Artificial Sequence

<220>
<223> Synthetic Polypeptide

<400> 88

Arg Ile Val Pro Ser Gly Gly Met Thr Arg Tyr Ala Asp Ser Val Lys
1 5 10 15

Gly

<210> 89
<211> 17
<212> PRT
<213> Artificial Sequence

<220>
<223> Synthetic Polypeptide

<400> 89

Arg Ile Tyr Pro Ser Gly Gly Val Thr Lys Tyr Ala Asp Ser Val Lys
1 5 10 15

Gly

<210> 90
<211> 17
<212> PRT
<213> Artificial Sequence

<220>
<223> Synthetic Polypeptide

<400> 90

Ser Ile Trp Pro Ser Gly Gly Lys Thr Thr Tyr Ala Asp Ser Val Lys
1 5 10 15

Gly

<210> 91
<211> 17
<212> PRT
<213> Artificial Sequence

<220>
<223> Synthetic Polypeptide

<400> 91

```
Ser Ile Trp Pro Ser Gly Gly Phe Thr Lys Tyr Ala Asp Ser Val Lys
1               5                   10                  15

Gly
```

<210> 92
<211> 17
<212> PRT
<213> Artificial Sequence

<220>
<223> Synthetic Polypeptide

<400> 92

```
Ser Ile Tyr Pro Ser Gly Gly Val Thr Arg Tyr Ala Asp Ser Val Lys
1               5                   10                  15

Gly
```

<210> 93
<211> 17
<212> PRT
<213> Artificial Sequence

<220>
<223> Synthetic Polypeptide

<400> 93

```
Ser Ile Tyr Pro Ser Gly Gly Lys Thr Ser Tyr Ala Asp Ser Val Lys
1               5                   10                  15

Gly
```

<210> 94
<211> 17
<212> PRT
<213> Artificial Sequence

<220>
<223> Synthetic Polypeptide

<400> 94

Val Ile Tyr Pro Ser Gly Ser Lys Thr Asn Tyr Ala Asp Ser Val Lys
1 5 10 15

Gly

<210> 95
<211> 17
<212> PRT
<213> Artificial Sequence

<220>
<223> Synthetic Polypeptide

<400> 95

Ser Ile Trp Pro Ser Gly Gly Met Thr Arg Tyr Ala Asp Ser Val Lys
1 5 10 15

Gly

<210> 96
<211> 17
<212> PRT
<213> Artificial Sequence

<220>
<223> Synthetic Polypeptide

<400> 96

Ser Ile Tyr Pro Ser Gly Gly Leu Thr Arg Tyr Ala Asp Ser Val Lys
1 5 10 15

Gly

<210> 97
<211> 17
<212> PRT
<213> Artificial Sequence

<220>
<223> Synthetic Polypeptide

<400> 97

Tyr Ile Tyr Pro Ser Gly Gly Asn Thr Arg Tyr Ala Asp Ser Val Lys
1 5 10 15

Gly

<210> 98
<211> 17
<212> PRT
<213> Artificial Sequence

<220>
<223> Synthetic Polypeptide

<400> 98

```
Ser Ile Trp Pro Ser Gly Gly Met Thr Lys Tyr Ala Asp Ser Val Lys
1               5                   10                  15

Gly
```

<210> 99
<211> 17
<212> PRT
<213> Artificial Sequence

<220>
<223> Synthetic Polypeptide

<400> 99

```
Arg Ile Tyr Pro Ser Gly Gly Ala Thr Gln Tyr Ala Asp Ser Val Lys
1               5                   10                  15

Gly
```

<210> 100
<211> 17
<212> PRT
<213> Artificial Sequence

<220>
<223> Synthetic Polypeptide

<400> 100

```
Arg Ile Tyr Pro Ser Gly Gly Leu Thr Gln Tyr Ala Asp Ser Val Lys
1               5                   10                  15

Gly
```

<210> 101
<211> 17
<212> PRT
<213> Artificial Sequence

<220>
<223> Synthetic Polypeptide

<400> 101

```
Arg Ile Tyr Pro Ser Gly Gly Ile Thr Ser Tyr Ala Asp Ser Val Lys
1               5                   10                  15

Gly
```

<210> 102
<211> 17
<212> PRT
<213> Artificial Sequence

<220>
<223> Synthetic Polypeptide

<400> 102

Ser Ile Tyr Pro Ser Gly Gly Met Thr Lys Tyr Ala Asp Ser Val Lys
1 5 10 15

Gly

<210> 103
<211> 17
<212> PRT
<213> Artificial Sequence

<220>
<223> Synthetic Polypeptide

<400> 103

Ser Ile Tyr Pro Ser Gly Gly Leu Thr Lys Tyr Ala Asp Ser Val Lys
1 5 10 15

Gly

<210> 104
<211> 17
<212> PRT
<213> Artificial Sequence

<220>
<223> Synthetic Polypeptide

<400> 104

Arg Ile Tyr Pro Ser Gly Gly Leu Thr Asn Tyr Ala Asp Ser Val Lys
1 5 10 15

Gly

<210> 105
<211> 17
<212> PRT
<213> Artificial Sequence

<220>
<223> Synthetic Polypeptide

<400> 105

Arg Ile Tyr Pro Ser Gly Gly Ser Thr His Tyr Ala Asp Ser Val Lys
1 5 10 15

Gly

<210> 106
<211> 17
<212> PRT
<213> Artificial Sequence

<220>
<223> Synthetic Polypeptide

<400> 106

Arg Ile Tyr Pro Ser Gly Gly Val Thr Gln Tyr Ala Asp Ser Val Lys
1 5 10 15

Gly

<210> 107
<211> 17
<212> PRT
<213> Artificial Sequence

<220>
<223> Synthetic Polypeptide

<400> 107

Arg Ile Tyr Pro Ser Gly Gly Ile Thr His Tyr Ala Asp Ser Val Lys
1 5 10 15

Gly

<210> 108
<211> 17
<212> PRT
<213> Artificial Sequence

<220>
<223> Synthetic Polypeptide

<400> 108

Ser Ile Trp Pro Ser Gly Gly Val Thr Lys Tyr Ala Asp Ser Val Lys
1 5 10 15

Gly

<210> 109
<211> 17
<212> PRT
<213> Artificial Sequence

<220>
<223> Synthetic Polypeptide

<400> 109

Tyr Ile Tyr Pro Ser Gly Gly His Thr Lys Tyr Ala Asp Ser Val Lys
1 5 10 15

Gly

<210> 110
<211> 17
<212> PRT
<213> Artificial Sequence

<220>
<223> Synthetic Polypeptide

<400> 110

Gly Ile Tyr Pro Ser Gly Gly Lys Thr Lys Tyr Ala Asp Ser Val Lys
1 5 10 15

Gly

<210> 111
<211> 14
<212> PRT
<213> Artificial Sequence

<220>
<223> Synthetic Polypeptide

<400> 111

Gln Arg Tyr Arg Gly Pro Lys Tyr Tyr Tyr Tyr Met Asp Val
1 5 10

<210> 112
<211> 14
<212> PRT
<213> Artificial Sequence

<220>
<223> Synthetic Polypeptide

<400> 112

Gln Arg Tyr Arg Gly Pro Lys Tyr Tyr Tyr Tyr Met Asp Ala
1 5 10

<210> 113
<211> 14
<212> PRT
<213> Artificial Sequence

<220>
<223> Synthetic Polypeptide

<400> 113

```
Gln Arg Tyr Arg Gly Pro Arg Tyr Tyr Tyr Tyr Ile Asp Ala
1               5                   10
```

<210> 114
<211> 16
<212> PRT
<213> Artificial Sequence

<220>
<223> Synthetic Polypeptide

<400> 114

```
Arg Ser Ser Gln Ser Leu Leu His Ser Asn Gly Tyr Asn Tyr Leu Asp
1               5                   10                  15
```

<210> 115
<211> 7
<212> PRT
<213> Artificial Sequence

<220>
<223> Synthetic Polypeptide

<400> 115

```
Leu Gly Ser Asn Arg Ala Ser
1               5
```

<210> 116
<211> 9
<212> PRT
<213> Artificial Sequence

<220>
<223> Synthetic Polypeptide

<400> 116

```
Met Gln Ala Leu Gln Thr Pro Trp Thr
1               5
```

<210> 117
<211> 5
<212> PRT
<213> Artificial Sequence

<220>
<223> Synthetic Polypeptide

<220>
<221> misc_feature
<222> (1)..(1)

<223> Xaa is Arg, Gln, Trp, His, Phe, Pro, Met, or Asn

<220>
<221> misc_feature
<222> (3)..(3)
<223> Xaa is Ile, Val, Thr, His, Ser, or Asn

<220>
<221> misc_feature
<222> (5)..(5)
<223> Xaa is His, Gly, Val, Ala, Arg, Gln, Tyr, Leu, Asn, or Ser

<400> 117

```
Xaa Tyr Xaa Met Xaa
1               5
```

<210> 118
<211> 17
<212> PRT
<213> Artificial Sequence

<220>
<223> Synthetic Polypeptide

<220>
<221> misc_feature
<222> (1)..(1)
<223> Xaa is Ser, Arg, Val, Tyr, or Gly

<220>
<221> misc_feature
<222> (3)..(3)
<223> Xaa is Tyr, Trp, Val, or Ser

<220>
<221> misc_feature
<222> (7)..(7)
<223> Xaa is Gly or Ser

<220>
<221> misc_feature
<222> (8)..(8)
<223> Xaa is Val, Lys, Met, Asn, Leu, Phe, Ala, Ile, Ser, His, or Arg

<220>
<221> misc_feature
<222> (10)..(10)
<223> Xaa is Lys, Arg, Thr, Gln, Ser, Asn, His, or Leu

<220>
<221> misc_feature
<222> (12)..(12)
<223> Xaa is Ala or Thr

<400> 118

Xaa Ile Xaa Pro Ser Gly Xaa Xaa Thr Xaa Tyr Xaa Asp Ser Val Lys
1 5 10 15

Gly

<210> 119
<211> 329
<212> PRT
<213> Homo sapiens

<400> 119

Ala Ser Thr Lys Gly Pro Ser Val Phe Pro Leu Ala Pro Ser Ser Lys
1 5 10 15

Ser Thr Ser Gly Gly Thr Ala Ala Leu Gly Cys Leu Val Lys Asp Tyr
20 25 30

Phe Pro Glu Pro Val Thr Val Ser Trp Asn Ser Gly Ala Leu Thr Ser
35 40 45

Gly Val His Thr Phe Pro Ala Val Leu Gln Ser Ser Gly Leu Tyr Ser
50 55 60

Leu Ser Ser Val Val Thr Val Pro Ser Ser Ser Leu Gly Thr Gln Thr
65 70 75 80

Tyr Ile Cys Asn Val Asn His Lys Pro Ser Asn Thr Lys Val Asp Lys
85 90 95

Arg Val Glu Pro Lys Ser Cys Asp Lys Thr His Thr Cys Pro Pro Cys
100 105 110

```
Pro Ala Pro Glu Leu Leu Gly Gly Pro Ser Val Phe Leu Phe Pro Pro
        115                 120                 125

Lys Pro Lys Asp Thr Leu Met Ile Ser Arg Thr Pro Glu Val Thr Cys
    130                 135                 140

Val Val Val Asp Val Ser His Glu Asp Pro Glu Val Lys Phe Asn Trp
145                 150                 155                 160

Tyr Val Asp Gly Val Glu Val His Asn Ala Lys Thr Lys Pro Arg Glu
                165                 170                 175

Glu Gln Tyr Asn Ser Thr Tyr Arg Val Val Ser Val Leu Thr Val Leu
            180                 185                 190

His Gln Asp Trp Leu Asn Gly Lys Glu Tyr Lys Cys Lys Val Ser Asn
        195                 200                 205

Lys Ala Leu Pro Ala Pro Ile Glu Lys Thr Ile Ser Lys Ala Lys Gly
    210                 215                 220

Gln Pro Arg Glu Pro Gln Val Tyr Thr Leu Pro Pro Ser Arg Glu Glu
225                 230                 235                 240

Met Thr Lys Asn Gln Val Ser Leu Thr Cys Leu Val Lys Gly Phe Tyr
                245                 250                 255

Pro Ser Asp Ile Ala Val Glu Trp Glu Ser Asn Gly Gln Pro Glu Asn
            260                 265                 270

Asn Tyr Lys Thr Thr Pro Pro Val Leu Asp Ser Asp Gly Ser Phe Phe
        275                 280                 285

Leu Tyr Ser Lys Leu Thr Val Asp Lys Ser Arg Trp Gln Gln Gly Asn
    290                 295                 300

Val Phe Ser Cys Ser Val Met His Glu Ala Leu His Asn His Tyr Thr
305                 310                 315                 320

Gln Lys Ser Leu Ser Leu Ser Pro Gly
                325
```

<210> 120
<211> 329
<212> PRT
<213> Artificial Sequence

<220>

<223> Synthetic Polypeptide

<400> 120

Ala Ser Thr Lys Gly Pro Ser Val Phe Pro Leu Ala Pro Ser Ser Lys
1   5   10   15

Ser Thr Ser Gly Gly Thr Ala Ala Leu Gly Cys Leu Val Lys Asp Tyr
            20   25   30

Phe Pro Glu Pro Val Thr Val Ser Trp Asn Ser Gly Ala Leu Thr Ser
        35   40   45

Gly Val His Thr Phe Pro Ala Val Leu Gln Ser Ser Gly Leu Tyr Ser
    50   55   60

Leu Ser Ser Val Val Thr Val Pro Ser Ser Ser Leu Gly Thr Gln Thr
65   70   75   80

Tyr Ile Cys Asn Val Asn His Lys Pro Ser Asn Thr Lys Val Asp Lys
                85   90   95

Arg Val Glu Pro Lys Ser Cys Asp Lys Thr His Thr Cys Pro Pro Cys
            100   105   110

Pro Ala Pro Glu Leu Leu Gly Gly Pro Ser Val Phe Leu Phe Pro Pro
        115   120   125

Lys Pro Lys Asp Thr Leu Tyr Ile Thr Arg Glu Pro Glu Val Thr Cys
    130   135   140

Val Val Val Asp Val Ser His Glu Asp Pro Glu Val Lys Phe Asn Trp
145   150   155   160

Tyr Val Asp Gly Val Glu Val His Asn Ala Lys Thr Lys Pro Arg Glu
                165   170   175

Glu Gln Tyr Asn Ser Thr Tyr Arg Val Val Ser Val Leu Thr Val Leu
            180   185   190

His Gln Asp Trp Leu Asn Gly Lys Glu Tyr Lys Cys Lys Val Ser Asn
        195   200   205

Lys Ala Leu Pro Ala Pro Ile Glu Lys Thr Ile Ser Lys Ala Lys Gly
    210   215   220

Gln Pro Arg Glu Pro Gln Val Tyr Thr Leu Pro Pro Ser Arg Glu Glu
225   230   235   240

```
Met Thr Lys Asn Gln Val Ser Leu Thr Cys Leu Val Lys Gly Phe Tyr
                245                 250                 255

Pro Ser Asp Ile Ala Val Glu Trp Glu Ser Asn Gly Gln Pro Glu Asn
            260                 265                 270

Asn Tyr Lys Thr Thr Pro Pro Val Leu Asp Ser Asp Gly Ser Phe Phe
        275                 280                 285

Leu Tyr Ser Lys Leu Thr Val Asp Lys Ser Arg Trp Gln Gln Gly Asn
    290                 295                 300

Val Phe Ser Cys Ser Val Met His Glu Ala Leu His Asn His Tyr Thr
305                 310                 315                 320

Gln Lys Ser Leu Ser Leu Ser Pro Gly
                325
```

<210> 121
<211> 5
<212> PRT
<213> Artificial Sequence

<220>
<223> Synthetic Polypeptide

<400> 121

```
Gln Tyr Ser Met His
1               5
```

<210> 122
<211> 17
<212> PRT
<213> Artificial Sequence

<220>
<223> Synthetic Polypeptide

<400> 122

```
Ser Ile Tyr Pro Ser Gly Gly Lys Thr Arg Tyr Ala Asp Ser Val Lys
1               5                   10                  15

Gly
```

<210> 123
<211> 17
<212> PRT
<213> Artificial Sequence

<220>
<223> Synthetic Polypeptide

<400> 123

Val Ile Trp Pro Ser Gly Gly Lys Thr Arg Tyr Ala Asp Ser Val Lys
1 5 10 15

Gly

<210> 124
<211> 17
<212> PRT
<213> Artificial Sequence

<220>
<223> Synthetic Polypeptide

<400> 124

Val Ile Tyr Pro Ser Gly Gly His Thr Arg Tyr Ala Asp Ser Val Lys
1 5 10 15

Gly

<210> 125
<211> 123
<212> PRT
<213> Artificial Sequence

<220>
<223> Synthetic Polypeptide

<400> 125

Glu Val Gln Leu Leu Glu Ser Gly Gly Gly Leu Val Gln Pro Gly Gly
1 5 10 15

Ser Leu Arg Leu Ser Cys Ala Ala Ser Gly Phe Thr Phe Ser Gln Tyr
20 25 30

Val Met His Trp Val Arg Gln Ala Pro Gly Lys Gly Leu Glu Trp Val
35 40 45

Ser Ser Ile Trp Pro Ser Gly Gly His Thr Arg Tyr Ala Asp Ser Val
50 55 60

Lys Gly Arg Phe Thr Ile Ser Arg Asp Asn Ser Lys Asn Thr Leu Tyr
65 70 75 80

Leu Gln Met Asn Ser Leu Arg Ala Glu Asp Thr Ala Val Tyr Tyr Cys
85 90 95

Thr Arg Gln Arg Tyr Arg Gly Pro Lys Tyr Tyr Tyr Tyr Met Asp Val
100 105 110

Trp Gly Lys Gly Thr Thr Val Thr Val Ser Ser
115 120

<210> 126
<211> 112
<212> PRT
<213> Artificial Sequence

<220>
<223> Synthetic Polypeptide

<400> 126

Asp Ile Val Met Thr Gln Ser Pro Leu Ser Leu Pro Val Thr Pro Gly
1 5 10 15

Glu Pro Ala Ser Ile Ser Cys Arg Ser Ser Gln Ser Leu Leu His Ser
20 25 30

Asn Gly Tyr Asn Tyr Leu Asp Trp Tyr Leu Gln Lys Pro Gly Gln Ser
35 40 45

Pro Gln Leu Leu Ile Tyr Leu Gly Ser Asn Arg Ala Ser Gly Val Pro
50 55 60

Asp Arg Phe Ser Gly Ser Gly Ser Gly Thr Asp Phe Thr Leu Lys Ile
65 70 75 80

Ser Arg Val Glu Ala Glu Asp Val Gly Val Tyr Tyr Cys Met Gln Ala
85 90 95

Leu Gln Thr Pro Trp Thr Phe Gly Gln Gly Thr Lys Val Glu Ile Lys
100 105 110

<210> 127
<211> 17
<212> PRT
<213> Artificial Sequence

<220>
<223> Synthetic Polypeptide

<400> 127

Ser Ile Trp Pro Ser Gly Gly His Thr Arg Tyr Ala Asp Ser Val His
1 5 10 15

Gly

<210> 128
<211> 615
<212> PRT
<213> Homo sapiens

<400> 128

```
Met Arg Ala Leu Leu Leu Leu Gly Phe Leu Leu Val Ser Leu Glu Ser
1               5                   10                  15

Thr Leu Ser Ile Pro Pro Trp Glu Ala Pro Lys Glu His Lys Tyr Lys
            20                  25                  30

Ala Glu Glu His Thr Val Val Leu Thr Val Thr Gly Glu Pro Cys His
        35                  40                  45

Phe Pro Phe Gln Tyr His Arg Gln Leu Tyr His Lys Cys Thr His Lys
    50                  55                  60

Gly Arg Pro Gly Pro Gln Pro Trp Cys Ala Thr Thr Pro Asn Phe Asp
65                  70                  75                  80

Gln Asp Gln Arg Trp Gly Tyr Cys Leu Glu Pro Lys Lys Val Lys Asp
                85                  90                  95

His Cys Ser Lys His Ser Pro Cys Gln Lys Gly Gly Thr Cys Val Asn
            100                 105                 110

Met Pro Ser Gly Pro His Cys Leu Cys Pro Gln His Leu Thr Gly Asn
        115                 120                 125

His Cys Gln Lys Glu Lys Cys Phe Glu Pro Gln Leu Leu Arg Phe Phe
    130                 135                 140

His Lys Asn Glu Ile Trp Tyr Arg Thr Glu Gln Ala Ala Val Ala Arg
145                 150                 155                 160

Cys Gln Cys Lys Gly Pro Asp Ala His Cys Gln Arg Leu Ala Ser Gln
                165                 170                 175

Ala Cys Arg Thr Asn Pro Cys Leu His Gly Gly Arg Cys Leu Glu Val
            180                 185                 190

Glu Gly His Arg Leu Cys His Cys Pro Val Gly Tyr Thr Gly Ala Phe
        195                 200                 205

Cys Asp Val Asp Thr Lys Ala Ser Cys Tyr Asp Gly Arg Gly Leu Ser
```

```
        210                 215                 220

Tyr Arg Gly Leu Ala Arg Thr Thr Leu Ser Gly Ala Pro Cys Gln Pro
225                 230                 235                 240

Trp Ala Ser Glu Ala Thr Tyr Arg Asn Val Thr Ala Glu Gln Ala Arg
                245                 250                 255

Asn Trp Gly Leu Gly Gly His Ala Phe Cys Arg Asn Pro Asp Asn Asp
            260                 265                 270

Ile Arg Pro Trp Cys Phe Val Leu Asn Arg Asp Arg Leu Ser Trp Glu
        275                 280                 285

Tyr Cys Asp Leu Ala Gln Cys Gln Thr Pro Thr Gln Ala Ala Pro Pro
    290                 295                 300

Thr Pro Val Ser Pro Arg Leu His Val Pro Leu Met Pro Ala Gln Pro
305                 310                 315                 320

Ala Pro Pro Lys Pro Gln Pro Thr Thr Arg Thr Pro Pro Gln Ser Gln
                325                 330                 335

Thr Pro Gly Ala Leu Pro Ala Lys Arg Glu Gln Pro Pro Ser Leu Thr
            340                 345                 350

Arg Asn Gly Pro Leu Ser Cys Gly Gln Arg Leu Arg Lys Ser Leu Ser
        355                 360                 365

Ser Met Thr Arg Val Val Gly Gly Leu Val Ala Leu Arg Gly Ala His
    370                 375                 380

Pro Tyr Ile Ala Ala Leu Tyr Trp Gly His Ser Phe Cys Ala Gly Ser
385                 390                 395                 400

Leu Ile Ala Pro Cys Trp Val Leu Thr Ala Ala His Cys Leu Gln Asp
                405                 410                 415

Arg Pro Ala Pro Glu Asp Leu Thr Val Val Leu Gly Gln Glu Arg Arg
            420                 425                 430

Asn His Ser Cys Glu Pro Cys Gln Thr Leu Ala Val Arg Ser Tyr Arg
        435                 440                 445

Leu His Glu Ala Phe Ser Pro Val Ser Tyr Gln His Asp Leu Ala Leu
    450                 455                 460
```

```
Leu Arg Leu Gln Glu Asp Ala Asp Gly Ser Cys Ala Leu Leu Ser Pro
465                 470                 475                 480

Tyr Val Gln Pro Val Cys Leu Pro Ser Gly Ala Ala Arg Pro Ser Glu
                485                 490                 495

Thr Thr Leu Cys Gln Val Ala Gly Trp Gly His Gln Phe Glu Gly Ala
            500                 505                 510

Glu Glu Tyr Ala Ser Phe Leu Gln Glu Ala Gln Val Pro Phe Leu Ser
        515                 520                 525

Leu Glu Arg Cys Ser Ala Pro Asp Val His Gly Ser Ser Ile Leu Pro
    530                 535                 540

Gly Met Leu Cys Ala Gly Phe Leu Glu Gly Gly Thr Asp Ala Cys Gln
545                 550                 555                 560

Gly Asp Ser Gly Gly Pro Leu Val Cys Glu Asp Gln Ala Ala Glu Arg
                565                 570                 575

Arg Leu Thr Leu Gln Gly Ile Ile Ser Trp Gly Ser Gly Cys Gly Asp
            580                 585                 590

Arg Asn Lys Pro Gly Val Tyr Thr Asp Val Ala Tyr Tyr Leu Ala Trp
        595                 600                 605

Ile Arg Glu His Thr Val Ser
    610                 615
```

Claims

1. A monoclonal antibody that binds to Factor XIIa (FXIIa) and does not bind to Factor XII (FXII), wherein the antibody comprises a heavy chain variable region which comprises an amino acid sequence of SEQ ID NOs:125 and a light chain variable region which comprises an amino acid sequence of SEQ ID NO:126.

2. The antibody of claim 1, wherein the antibody is a full length antibody or an antigen-binding fragment thereof, or a Fab.

3. The antibody of any one of claims 1-2, wherein the heavy chain further comprises a heavy chain constant region, preferably

   wherein the heavy chain constant region comprises at least one mutation that enhances half-life of the antibody as compared to the wild-type counterpart, more preferred
   wherein the at least one mutation is at a position corresponding to position 145, 147, or 149 of SEQ ID NO:119, even more preferred
   wherein the at least one mutation is an amino acid substitution selected from the group consisting ofM145Y, S147T, and T149E, and/or
   wherein the heavy chain constant region comprises amino acid substitutions at positions corresponding to positions 145, 147, and 149 of SEQ ID NO:119, preferably
   wherein the heavy chain constant region comprises amino acid substitutions M145Y, S147T, and T149E, and/or

wherein the heavy chain constant region comprises the amino acid sequence of SEQ ID NO: 120.

4. An isolated nucleic acid or a set of nucleic acids, which collectively encode the antibody of any one of claims 1-3.

5. A vector or vector set, comprising the nucleic acid or the set of nucleic acids of claim 4, wherein the vector(s) is (are) preferably an expression vector(s).

6. A host cell or host cell set, comprising the vector or vector set of claim 5, preferably

wherein the cell(s) is a bacterial cell, a yeast cell, an insect cell, a plant cell, or a mammalian cell, and more preferred
wherein the cell(s) is a CHO cell.

7. A method of producing an antibody that binds to Factor XIIa (FXIIa), comprising:

culturing the host cell or host cell set of claim 6 in a culture medium; and
collecting the cultured host cells or the culture medium for isolation of the antibody,
wherein the method further comprises isolating the antibody from the cultured host cells or the culture medium.

8. A pharmaceutical composition, comprising

(a) the antibody of any one of claims 1-3, the nucleic acid or the set of nucleic acids of claim 4, or the vector or vector set of claim 5; and
(b) a pharmaceutically acceptable carrier.

9. The antibody of any one of claims 1-3, the nucleic acid or set of nucleic acids of claim 4, the vector or vector set of claim 5, or the pharmaceutical composition of claim 8, for use in a method of treating thrombosis, hereditary angioedema (HAE) or an ocular disease, which are associated with contact system activation.

10. The antibody, the nucleic acid (set), vector (set) or pharmaceutical composition for the use of claim 9, wherein the ocular disease is selected from the group consisting of macular edema, diabetic retinopathy, hypertensive retinopathy, age-related macular degeneration, and retinal vein occlusion.

11. The antibody, the nucleic acid (set), vector (set) or pharmaceutical composition for the use of claim 9 or 10, wherein the thrombosis is associated with atrial fibrillation, deep vein thrombosis (DVT), pulmonary embolism, stroke, or an arterial or venous thrombotic event.

12. The antibody, the nucleic acid (set), vector (set) or pharmaceutical composition for the use of any one of claims 9-11, wherein the subject has, is suspected of having, or is at risk for hereditary angioedema (HAE), in particular wherein the HAE is Type 1, Type 2, or Type 3 HAE.

Patentansprüche

1. Monoklonaler Antikörper, der an Faktor XIIa (FXIIa) bindet und nicht an Faktor XII (FXII) bindet,
wobei der Antikörper eine variable Region der schweren Kette umfasst, die eine Aminosäuresequenz von SEQ ID NO: 125 umfasst, und eine variable Region der leichten Kette umfasst, die eine Aminosäuresequenz von SEQ ID NO: 126 umfasst.

2. Antikörper gemäß Anspruch 1, wobei der Antikörper ein Antikörper in voller Länge oder ein Antigen-bindendes Fragment davon oder ein Fab ist.

3. Antikörper gemäß irgendeinem der Ansprüche 1-2, wobei die schwere Kette weiter eine konstante Region der schweren Kette umfasst, bevorzugt

wobei die konstante Region der schweren Kette mindestens eine Mutation umfasst, die die Halbwertszeit des Antikörpers verglichen mit dem Wild-Typ Gegenstück erhöht, bevorzugter
wobei die mindestens eine Mutation an einer Position entsprechend Position 145, 147 oder 149 von SEQ ID

NO: 119 liegt, noch bevorzugter
wobei die mindestens eine Mutation eine Aminosäuresubstitution ausgewählt aus der Gruppe bestehend aus M145Y, S147T und T149E ist, und/oder
wobei die konstante Region der schweren Kette Aminosäuresubstitutionen an Positionen entsprechend Positionen 145, 147 und 149 von SEQ ID NO: 119 umfasst, bevorzugt
wobei die konstante Region der schweren Kette Aminosäuresubstitutionen M145Y, S147T und T149E umfasst, und/oder
wobei die konstante Region der schweren Kette die Aminosäuresequenz von SEQ ID NO: 120 umfasst.

4. Isolierte Nukleinsäure oder Satz von Nukleinsäuren, welche gemeinsam den Antikörper gemäß irgendeinem der Ansprüche 1-3 kodieren.

5. Vektor oder Satz von Vektoren, umfassend die Nukleinsäure oder den Satz von Nukleinsäuren gemäß Anspruch 4, wobei der Vektor (die Vektoren) bevorzugt Expressionsvektor(en) ist(sind).

6. Wirtszelle oder Satz von Wirtszellen, umfassend den Vektor oder den Satz von Vektoren gemäß Anspruch 5, bevorzugt

wobei die Zelle(n) eine Bakterienzelle, eine Hefezelle, eine Insektenzelle, eine Pflanzenzelle oder eine Säugetierzelle ist, und bevorzugter
wobei die Zelle(n) eine CHO-Zelle ist.

7. Herstellungsverfahren für einen Antikörper, der an Faktor XIIa (FXIIa) bindet, umfassend:

Kultivieren der Wirtszelle oder des Satzes von Wirtszellen gemäß Anspruch 6 in einem Kulturmedium; und
Sammeln der kultivierten Wirtszellen oder des Kulturmediums zur Isolation des Antikörpers,
wobei das Verfahren weiter Isolieren des Antikörpers aus den kultivierten Wirtszellen oder dem Kulturmedium umfasst.

8. Pharmazeutische Zusammensetzung, umfassend:

(a) den Antikörper gemäß irgendeinem der Ansprüche 1-3, die Nukleinsäure oder den Satz von Nukleinsäuren gemäß Anspruch 4, oder den Vektor oder den Satz von Vektoren gemäß Anspruch 5; und
(b) einen pharmazeutisch akzeptablen Träger.

9. Antikörper gemäß irgendeinem der Ansprüche 1-3, Nukleinsäure oder Satz von Nukleinsäuren gemäß Anspruch 4, Vektor oder Satz von Vektoren gemäß Anspruch 5 oder pharmazeutische Zusammensetzung gemäß Anspruch 8, zur Verwendung in einem Verfahren für die Behandlung von Thrombose, hereditärem Angioödem (HAE) oder einer Augenerkrankung, welche mit Kontaktsystemaktivierung ("contact system activation") im Zusammenhang stehen.

10. Antikörper, Nukleinsäure (Satz), Vektor (Satz) oder pharmazeutische Zusammensetzung zur Verwendung gemäß Anspruch 9, wobei die Augenerkrankung ausgewählt ist aus der Gruppe bestehend aus Makulaödem, diabetischer Retinopathie, hypertensiver Retinopathie, altersbedingter Makuladegeneration und Netzhautvenenverschluss.

11. Antikörper, Nukleinsäure (Satz), Vektor (Satz) oder pharmazeutische Zusammensetzung zur Verwendung gemäß Anspruch 9 oder 10, wobei die Thrombose mit Vorhofflimmern, tiefer Venenthrombose (DVT), Lungenembolie, Schlaganfall oder einem arteriellen oder venösen thrombotischen Ereignis im Zusammenhang steht.

12. Antikörper, Nukleinsäure (Satz), Vektor (Satz) oder pharmazeutische Zusammensetzung zur Verwendung gemäß irgendeinem der Ansprüche 9-11, wobei das Subjekt hereditäres Angioödem (HAE) hat, vermutet wird, dass das Subjekt dieses hat, oder das Subjekt einem Risiko dieses zu bekommen ausgesetzt ist, insbesondere wobei das HAE Typ 1, Typ 2 oder Typ 3 HAE ist.

Revendications

1. Anticorps monoclonal qui se lie au facteur XIIa (FXIIa), mais qui ne se lie pas au facteur XII (FXII),

dans lequel l'anticorps comprend une région variable de chaîne lourde qui comprend une séquence d'acides aminés de SEQ ID NO: 125 et une région variable de chaîne légère qui comprend une séquence d'acides aminés de SEQ ID NO: 126.

2. Anticorps selon la revendication 1, dans lequel l'anticorps est un anticorps pleine longueur ou un fragment de celui-ci se liant à un antigène, ou un Fab.

3. Anticorps selon l'une quelconque des revendications 1-2, dans lequel la chaîne lourde comprend en outre une région constante de chaîne lourde, de préférence

dans lequel la région constante de chaîne lourde comprend au moins une mutation qui améliore la demi-vie de l'anticorps par rapport à son homologue de type sauvage, plus préférentiellement
dans lequel la au moins une mutation se situe à une position correspondant à la position 145, 147 ou 149 de SEQ ID NO: 119, encore plus préférentiellement
dans lequel la au moins une mutation est une substitution d'acide aminé choisie parmi le groupe consistant en M145Y, S147T et T149E, et/ou
dans lequel la région constante de chaîne lourde comprend des substitutions d'acide aminé situées à des positions correspondant aux positions 145, 147 et 149 de SEQ ID NO: 119, de préférence
dans lequel la région constante de chaîne lourde comprend des substitutions d'acide aminé M145Y, S147T et T149E, et/ou
dans lequel la région constante de chaîne lourde comprend la séquence d'acides aminés de SEQ ID NO: 120.

4. Acide nucléique isolé ou ensemble d'acides nucléiques, qui codent ensemble pour l'anticorps selon l'une quelconque des revendications 1-3.

5. Vecteur ou ensemble de vecteurs, comprenant l'acide nucléique ou l'ensemble d'acides nucléiques selon la revendication 4, dans lequel le(s) vecteur(s) est(sont) de préférence un(des) vecteur(s) d'expression.

6. Cellule hôte ou ensemble de cellules hôtes, comprenant le vecteur ou l'ensemble de vecteurs selon la revendication 5, de préférence

dans laquelle/lequel la(les) cellule(s) est(sont) une cellule bactérienne, une cellule de levure, une cellule d'insecte, une cellule végétale ou une cellule de mammifère, et plus préférentiellement
dans laquelle/lequel la(les) cellule(s) est(sont) une cellule CHO.

7. Procédé de production d'un anticorps qui se lie au facteur XIIa (FXIIa), comprenant :

la mise en culture de la cellule hôte ou de l'ensemble de cellules hôtes selon la revendication 6 dans un milieu de culture ; et
le prélèvement des cellules hôtes mises en culture ou du milieu de culture pour isoler l'anticorps,
dans lequel le procédé comprend en outre l'isolement de l'anticorps à partir des cellules hôtes mises en culture ou du milieu de culture.

8. Composition pharmaceutique, comprenant

(a) l'anticorps selon l'une quelconque des revendications 1-3, l'acide nucléique ou l'ensemble d'acides nucléiques selon la revendication 4, ou le vecteur ou l'ensemble de vecteurs selon la revendication 5 ; et
(b) un support pharmaceutiquement acceptable.

9. Anticorps selon l'une quelconque des revendications 1-3, acide nucléique ou ensemble d'acides nucléiques selon la revendication 4, vecteur ou ensemble de vecteurs selon la revendication 5, ou composition pharmaceutique selon la revendication 8, destiné(e) à être utilisé(e) dans un procédé de traitement d'une thrombose, d'un angiœdème héréditaire (AOH) ou d'une maladie oculaire, qui sont associés à l'activation du système de contact.

10. Anticorps, (ensemble des) acide(s) nucléique(s), (ensemble des) vecteur(s) ou composition pharmaceutique destiné(e) à être utilisé(e) selon la revendication 9, dans lequel(laquelle) la maladie oculaire est choisie parmi le groupe consistant en œdème maculaire, rétinopathie diabétique, rétinopathie hypertensive, dégénérescence maculaire liée à l'âge et occlusion veineuse rétinienne.

11. Anticorps, (ensemble des) acide(s) nucléique(s), (ensemble des) vecteur(s) ou composition pharmaceutique destiné(e) à être utilisé(e) selon la revendication 9 ou 10, dans lequel(laquelle) la thrombose est associée à une fibrillation auriculaire, à une thrombose veineuse profonde (TVP), à une embolie pulmonaire, à un accident vasculaire cérébral, ou à un événement thrombotique artériel ou veineux.

12. Anticorps, (ensemble des) acide(s) nucléique(s), (ensemble des) vecteur(s) ou composition pharmaceutique destiné(e) à être utilisé(e) selon l'une quelconque des revendications 9-11, dans lequel(laquelle) le sujet présente, est suspecté de présenter, ou présente un risque de développer un angiœdème héréditaire (AOH), en particulier dans lequel(laquelle) l'AOH est un AOH de type 1, de type 2 ou de type 3.

FIGURE 1

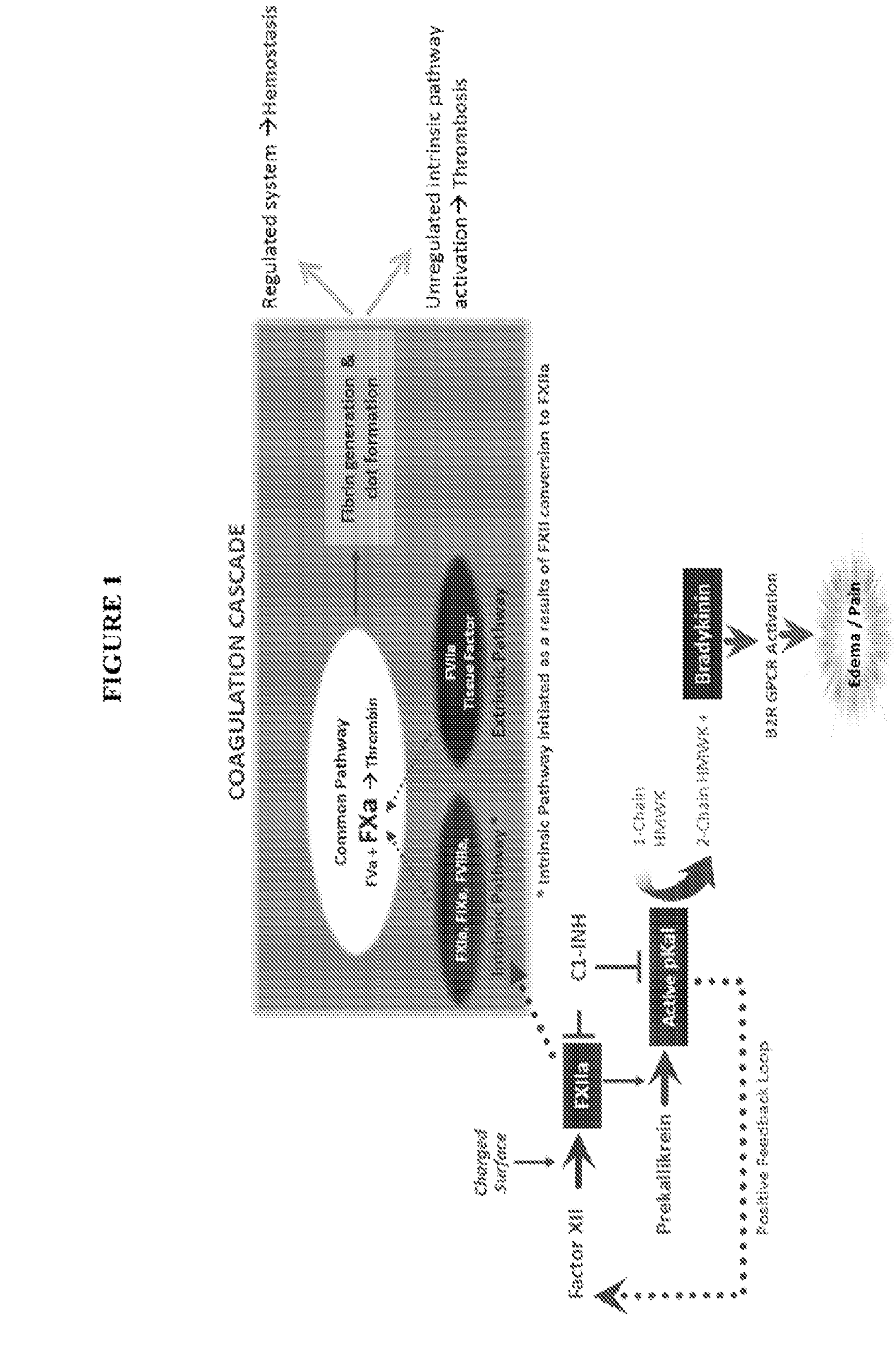

COAGULATION CASCADE
Common Pathway
FVa + FXa → Thrombin
Fibrin generation & clot formation
FXIa, FIXa, FVIIIa
Intrinsic Pathway *
FVIIa
Tissue Factor
Extrinsic Pathway
* Intrinsic Pathway initiated as a results of FXII conversion to FXIIa
Regulated system → Hemostasis
Unregulated intrinsic pathway activation → Thrombosis
Charged Surface
Factor XII
FXIIa
C1-INH
Prekallikrein
Active pKal
1-Chain HMWK
2-Chain HMWK +
Bradykinin
B2R GPCR Activation
Edema / Pain
Positive Feedback Loop

FIGURE 2

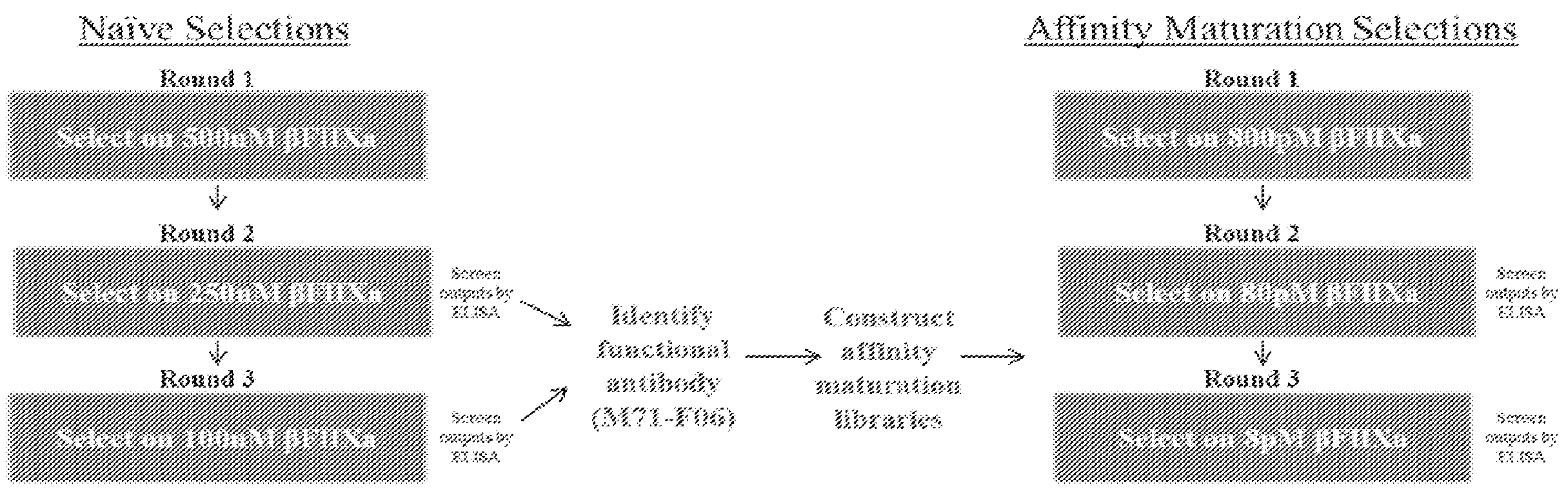
Naïve Selections
Round 1
Select on 500nM βFIIXa
Round 2
Select on 250nM βFIIXa
Round 3
Select on 100nM βFIIXa
Screen outputs by ELISA
Screen outputs by ELISA
Identify functional antibody (M71-F06)
Construct affinity maturation libraries
Affinity Maturation Selections
Round 1
Round 2
Round 3
Screen outputs by ELISA
Screen outputs by ELISA

FIGURE 3

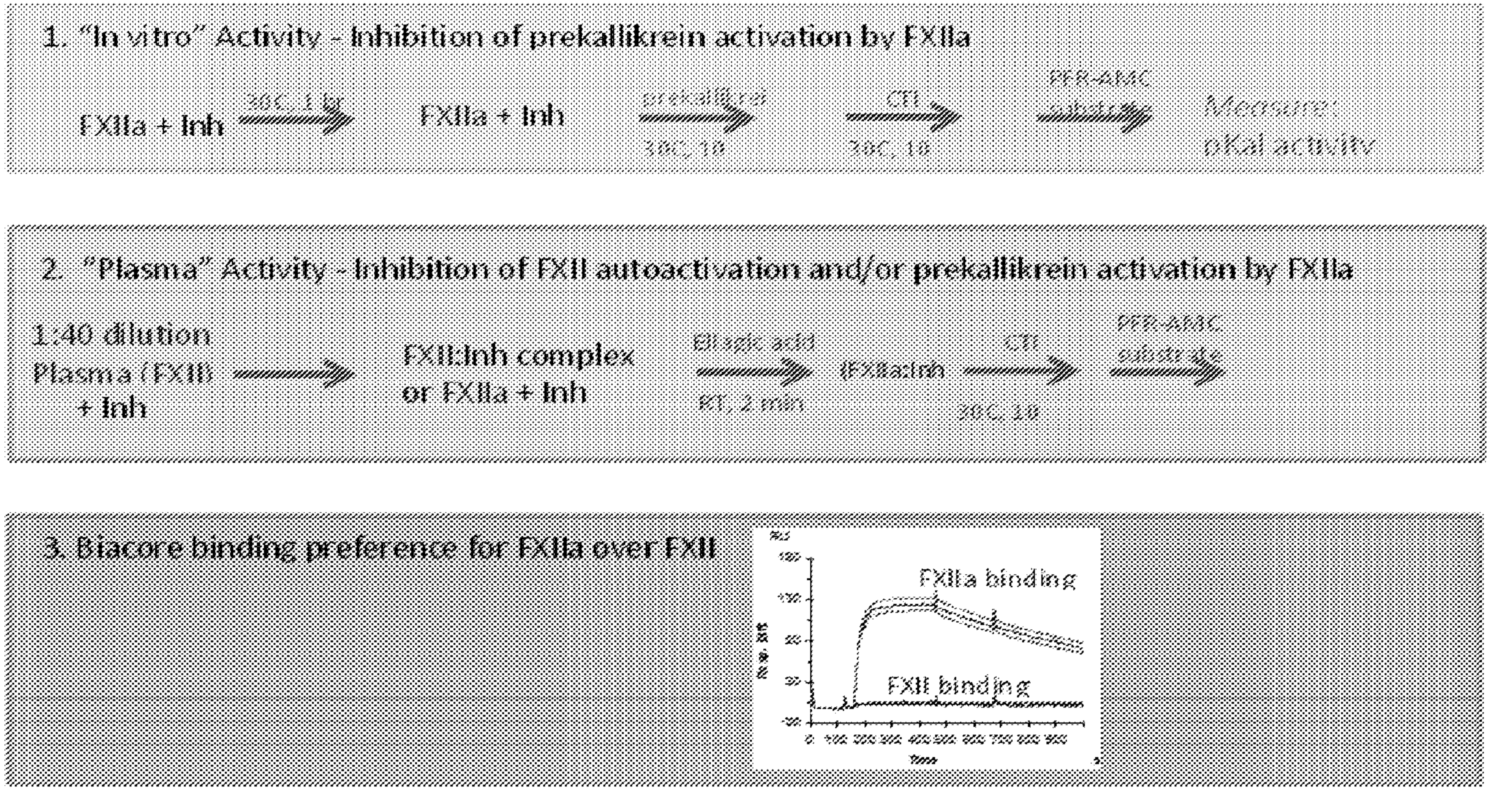
1. "In vitro" Activity - Inhibition of prekallikrein activation by FXIIa
FXIIa + Inh
38C, 1 hr
FXIIa + Inh
prekallikrein
38C, 1B
CTI
38C, 1B
PFR-AMC
substrate
Measure:
pKal activity
2. "Plasma" Activity - Inhibition of FXII autoactivation and/or prekallikrein activation by FXIIa
1:40 dilution
Plasma (FXII)
+ Inh
FXII:Inh complex
or FXIIa + Inh
Ellagic acid
RT, 2 min
(FXIIa:Inh
CTI
38C, 1B
PFR-AMC
substrate
3. Biacore binding preference for FXIIa over FXII
FXIIa binding
FXII binding
Time

FIGURE 4

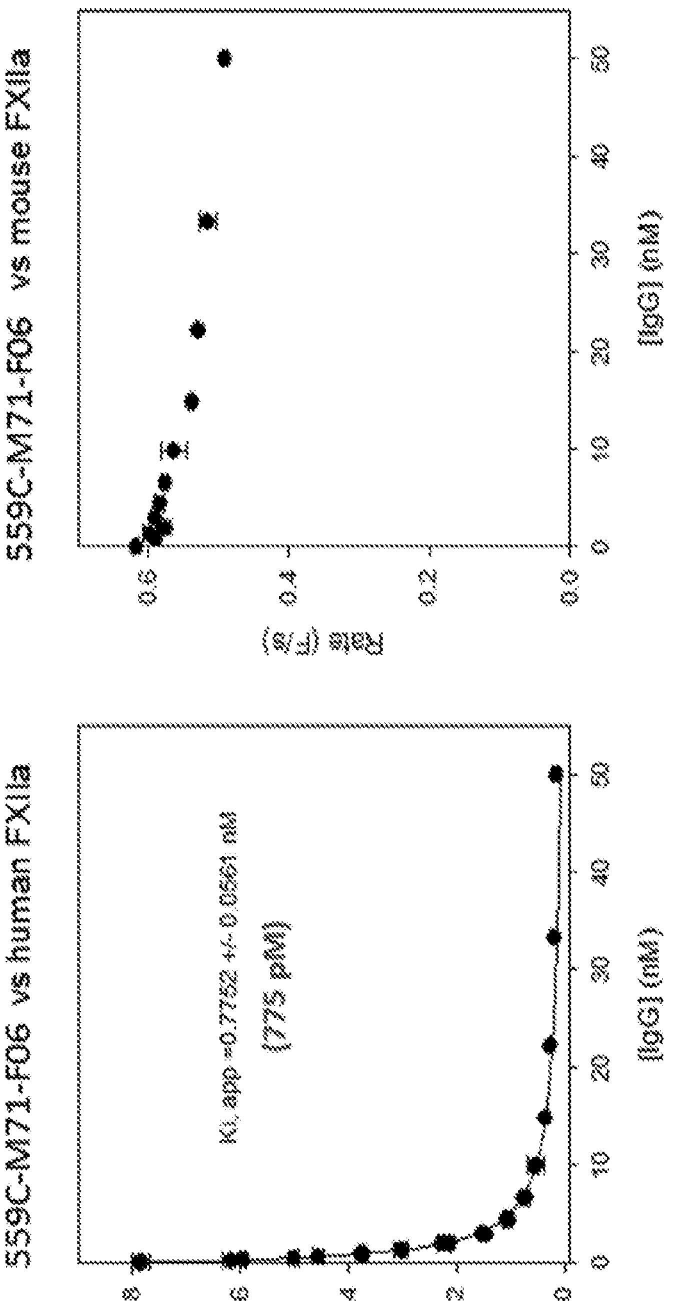
559C-M71-F06 vs human FXIIa
Ki, app =0.7752 +/- 0.0561 nM
(775 pM)
Rate (F/s)
[IgG] (nM)
559C-M71-F06 vs mouse FXIIa
Rate (F/s)
[IgG] (nM)

FIGURE 5

APTT

- 1 µM IgG or PBS pre-incubated with plasma ~ 15 min, FXII activation initiated (IgG now ~0.5uM) 3 minutes
- At t = 0 secs $CaCl_2$ added and coagulation time recorded
- Times normalized to changes vs PBS addition (t^{exp} / t_{avg}^{PBS}) due to differences in base coagulation times for different plasma

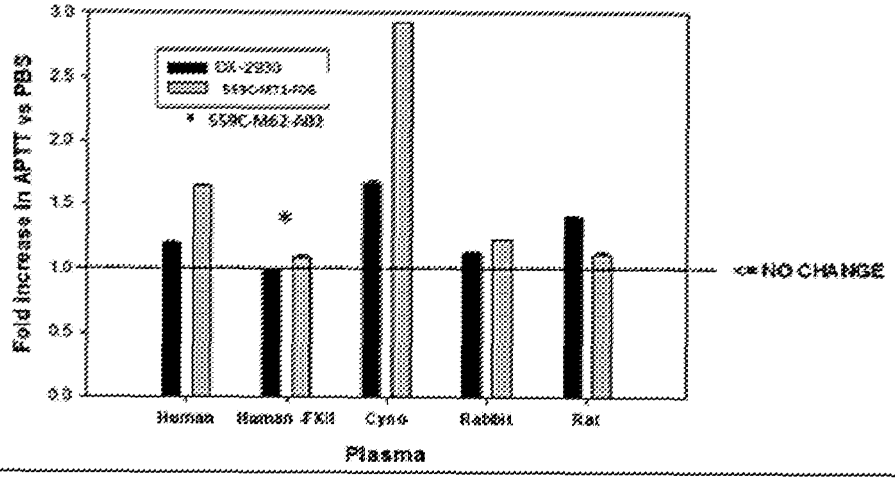

- Some coagulation delay in FXII deficient plasma -- off target inhibition, or residual FXII?
- Measureable coagulation delay in all species - excellent Cyno inhibition
- M71-F06 poor activity against rat (expected due to poor mouse cross-reactivity)

FIGURE 6

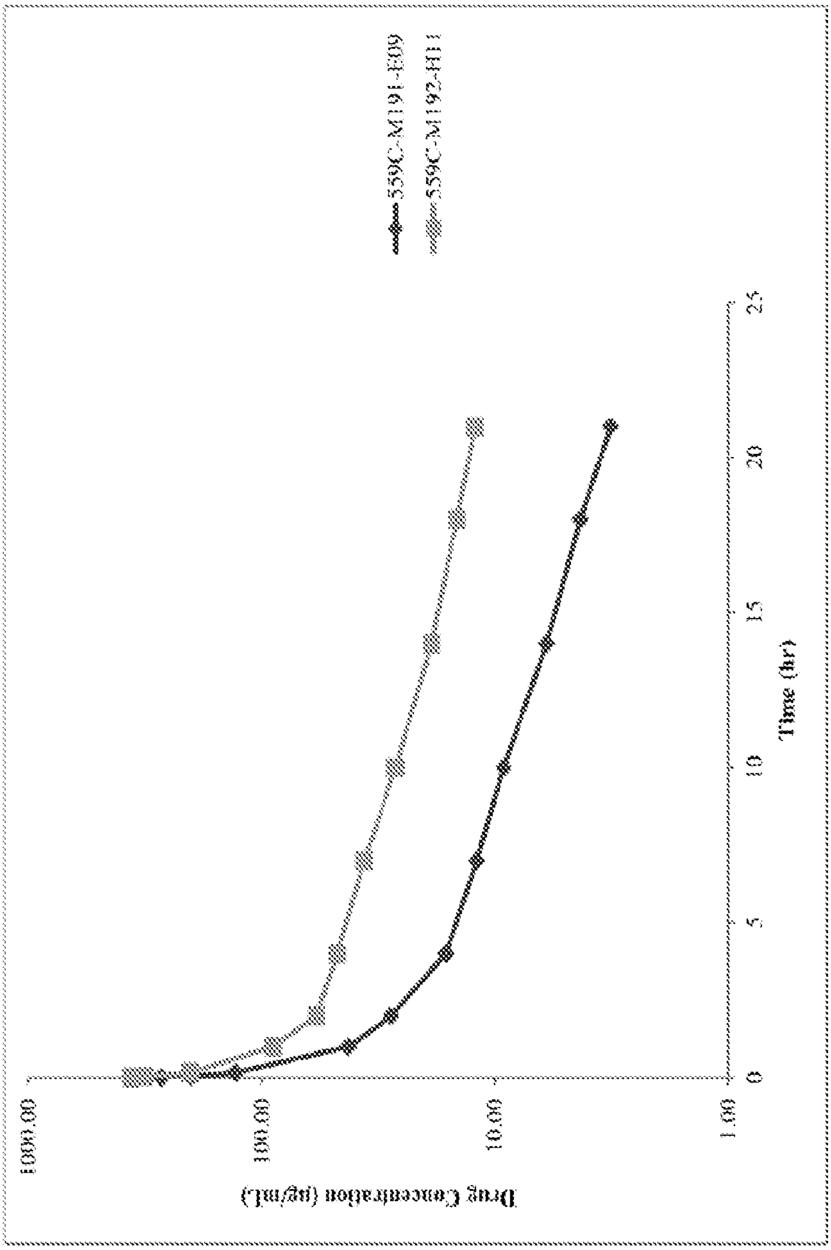

Drug Concentration (µg/mL)
1000.00
100.00
10.00
1.00
Time (hr)
0
5
10
15
20
25
559C-M191-E09
559C-M192-H11

FIGURE 7

A.

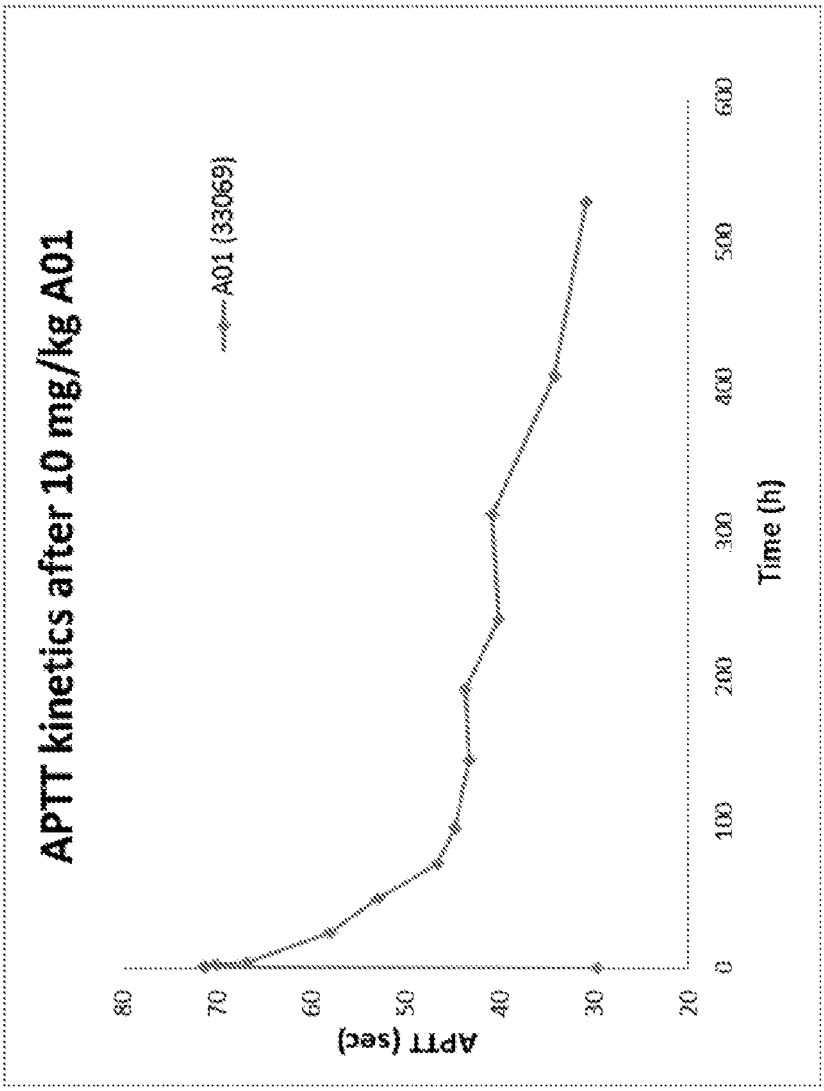
APTT kinetics after 10 mg/kg A01
A01 (33069)
APTT (sec)
80
70
60
50
40
30
20
0
100
200
300
400
500
600
Time (h)

FIGURE 7 (Cont'd)

B.

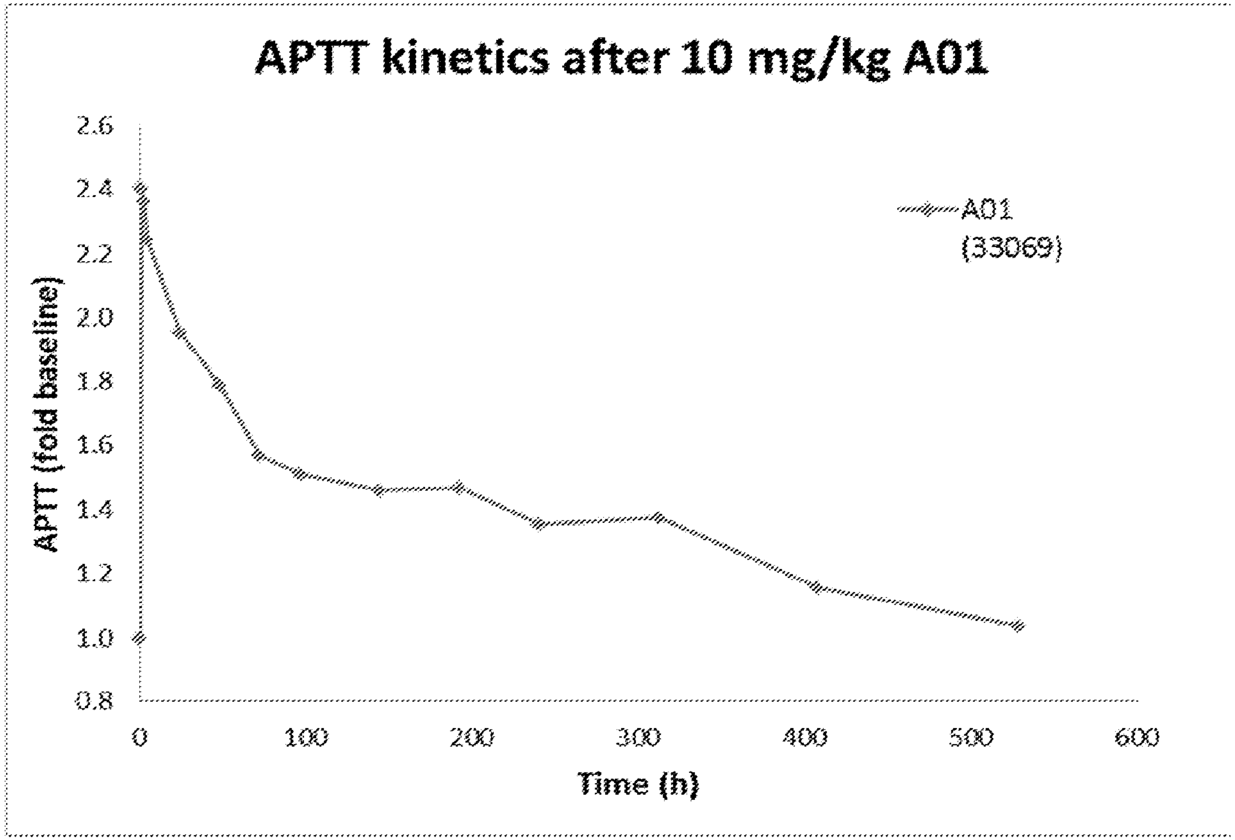

FIGURE 7 (Cont'd)

C.

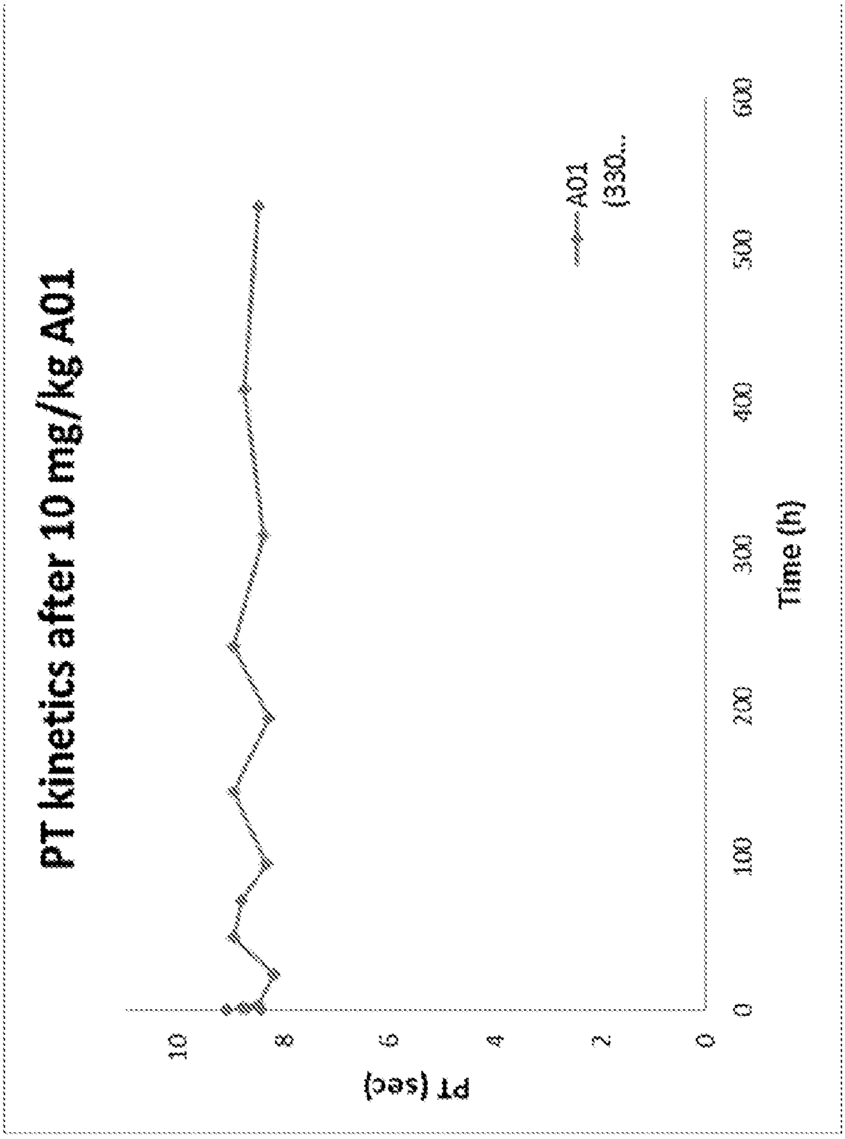

FIGURE 8

A.

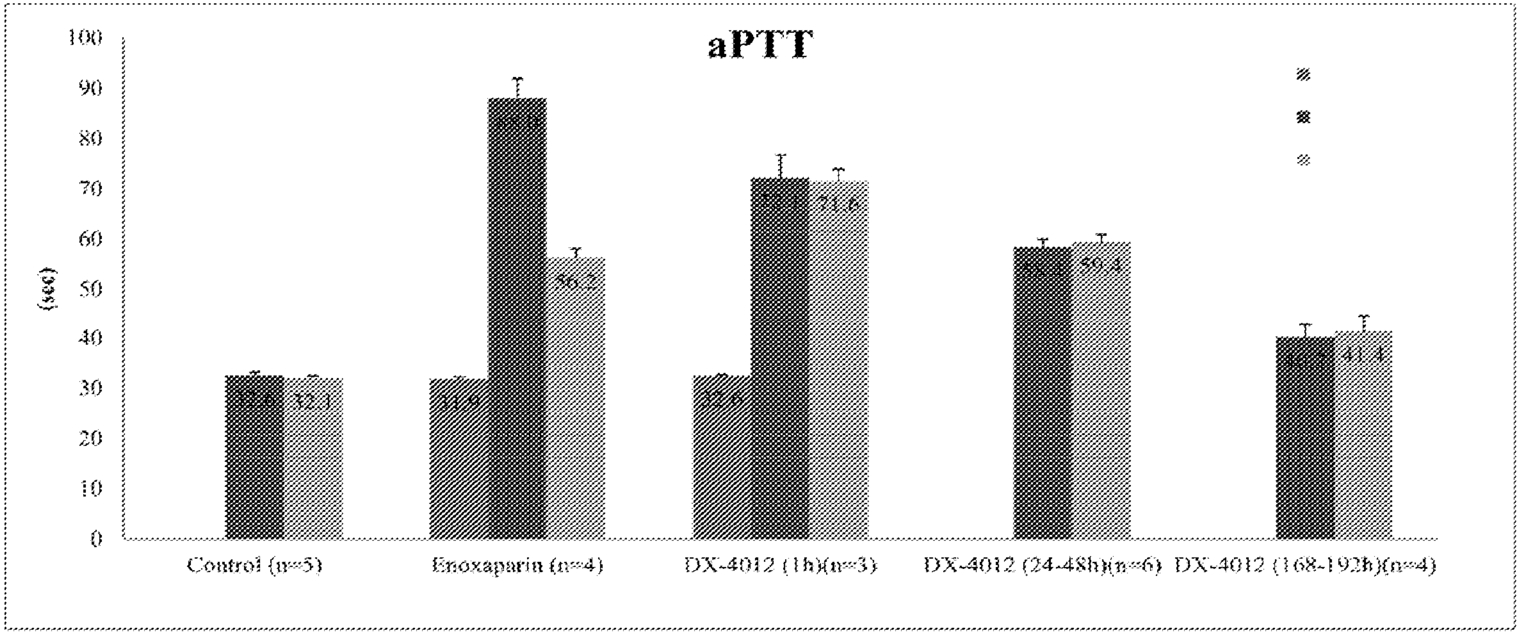
aPTT
(sec)
100
90
80
70
60
50
40
30
20
10
0
32.1
56.2
32.6
71.6
59.4
41.4
Control (n=5)
Enoxaparin (n=4)
DX-4012 (1h)(n=3)
DX-4012 (24-48h)(n=6)
DX-4012 (168-192h)(n=4)

FIGURE 8 (Cont'd)

B.

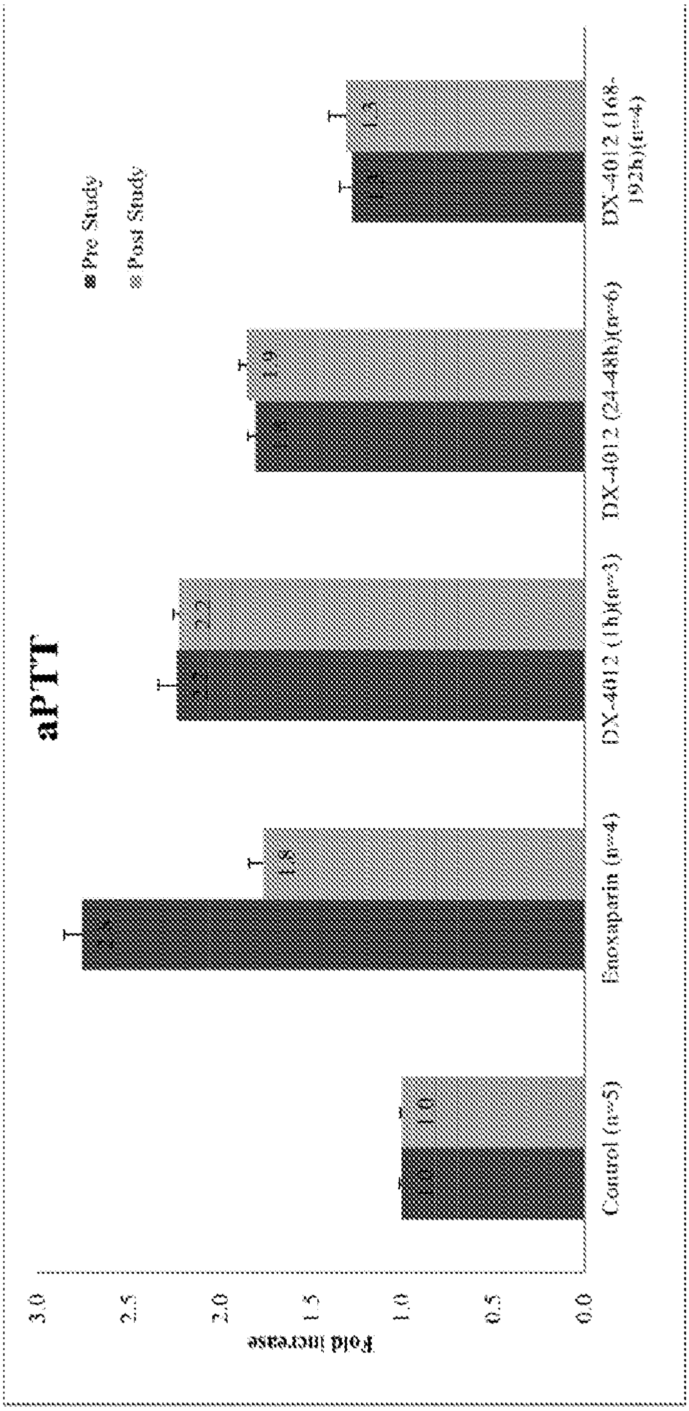

FIGURE 9

A.

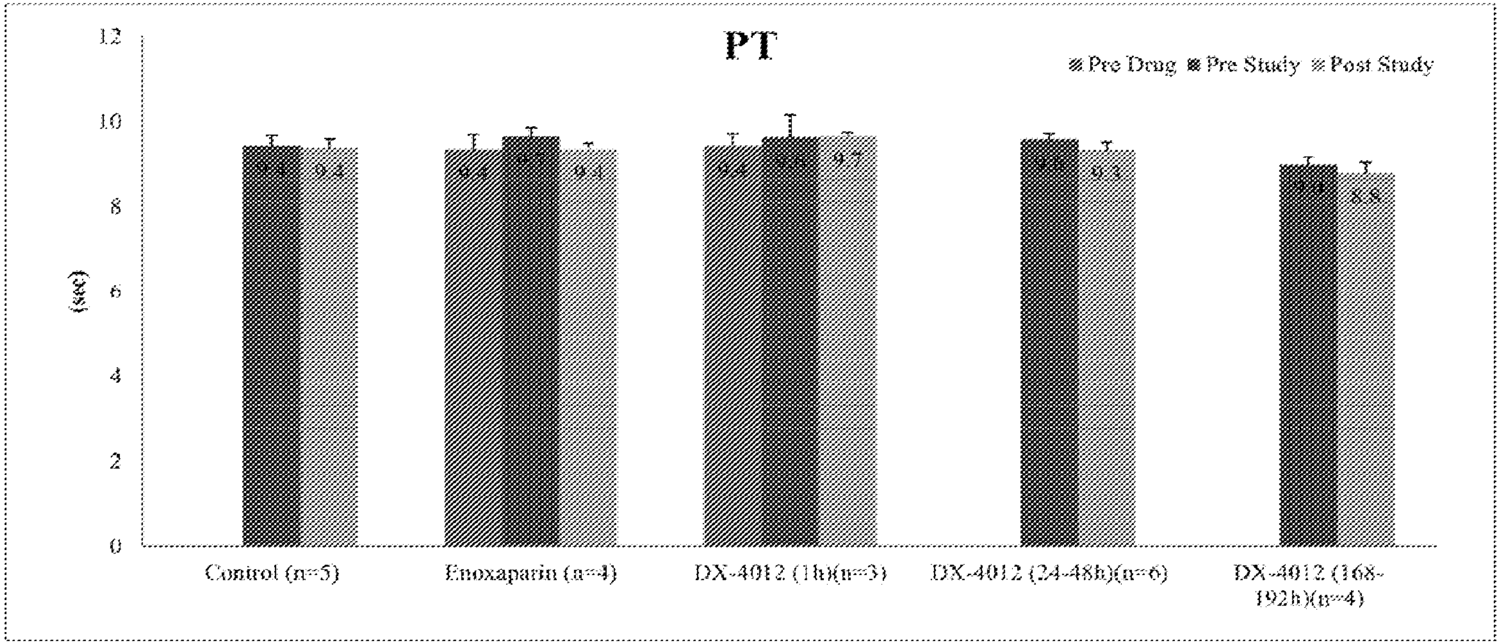
PT
Pre Drug
Pre Study
Post Study
(sec)
0
2
4
6
8
10
12
Control (n=5)
Enoxaparin (n=4)
DX-4012 (1h)(n=3)
DX-4012 (24-48h)(n=6)
DX-4012 (168-192h)(n=4)
9.4
9.4
9.4
9.4
9.4
9.7
9.3
8.8

FIGURE 9 (Cont'd)

B.

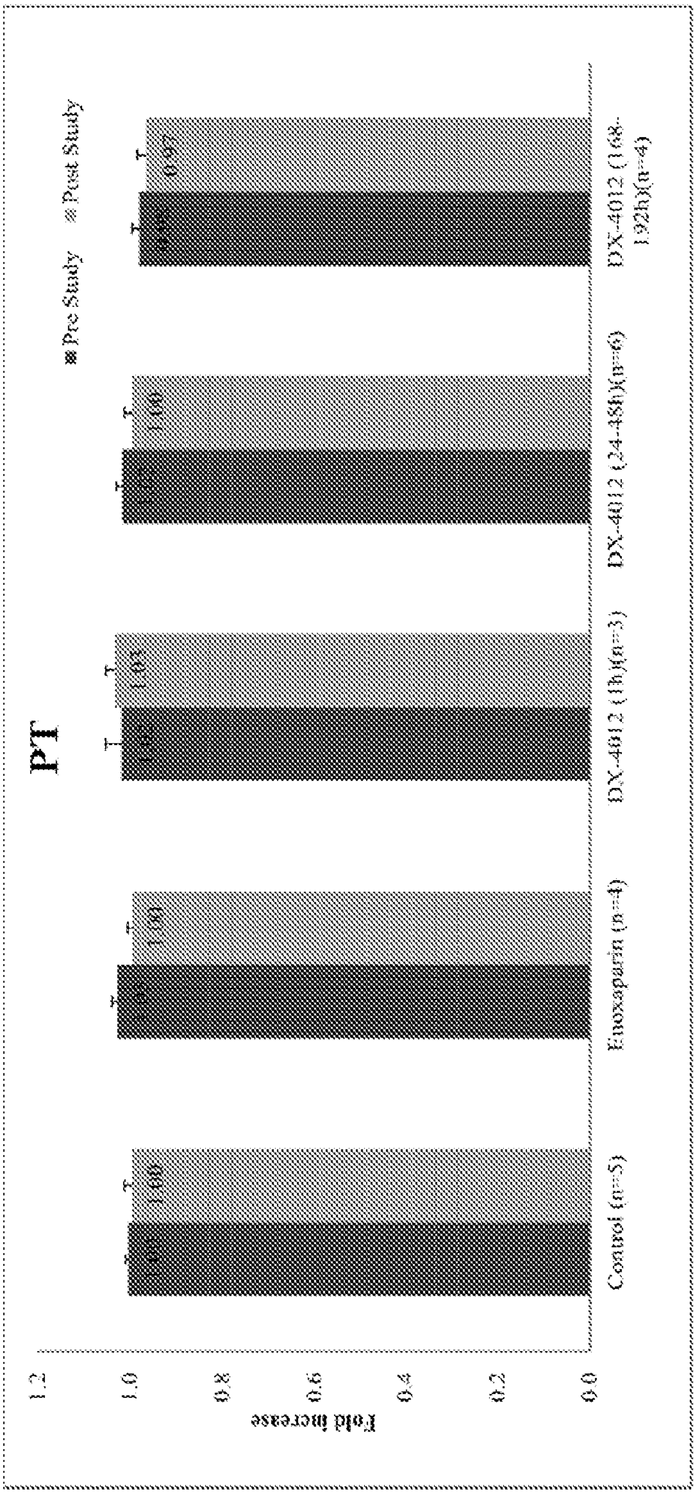

FIGURE 10

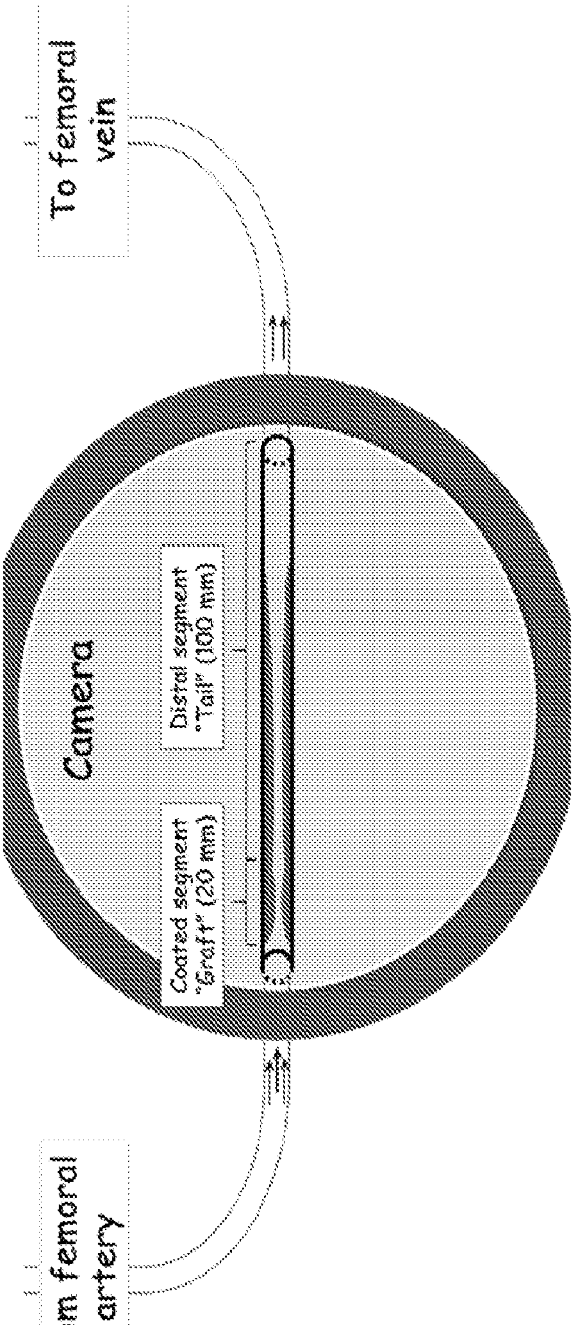
To femoral vein
Camera
Distal segment "Tail" (100 mm)
Coated segment "Graft" (20 mm)
From femoral artery

FIGURE 11

A.

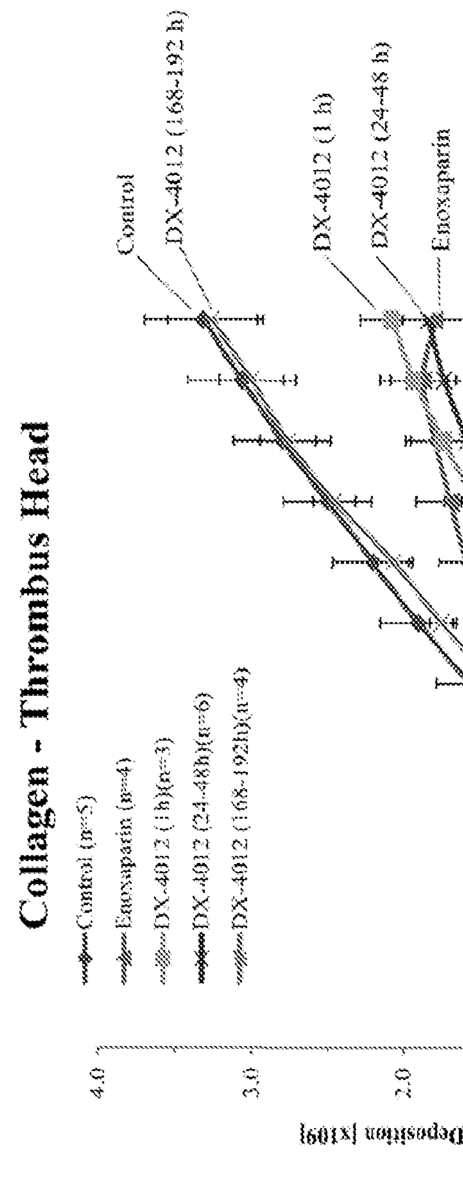
Collagen - Thrombus Head
Control (n=5)
Enoxaparin (n=4)
DX-4012 (1h)(n=3)
DX-4012 (24-48h)(n=6)
DX-4012 (168-192h)(n=4)
Control
DX-4012 (168-192 h)
DX-4012 (1 h)
DX-4012 (24-48 h)
Enoxaparin
Platelet Deposition [x109]
0.0
1.0
2.0
3.0
4.0
Time [min]
0
10
20
30
40
50
60

FIGURE 11 (Cont'd)

B.

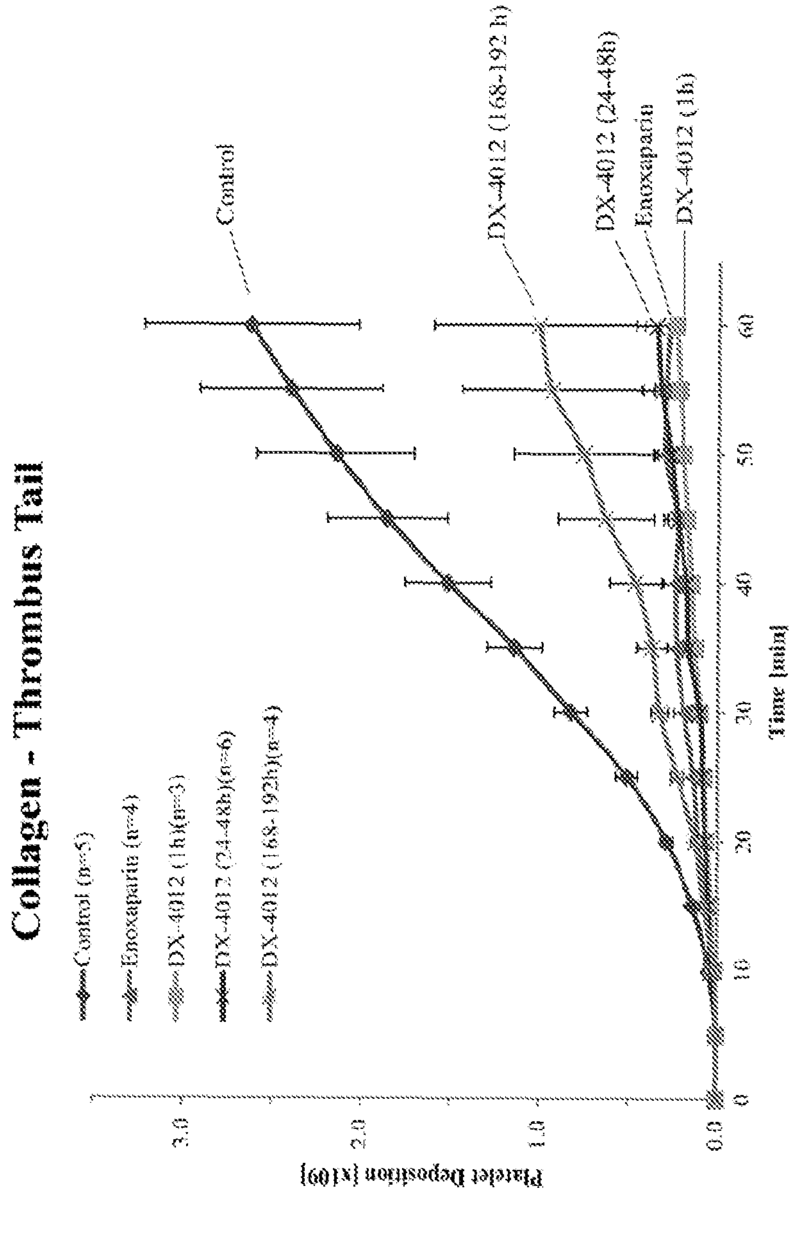

FIGURE 12

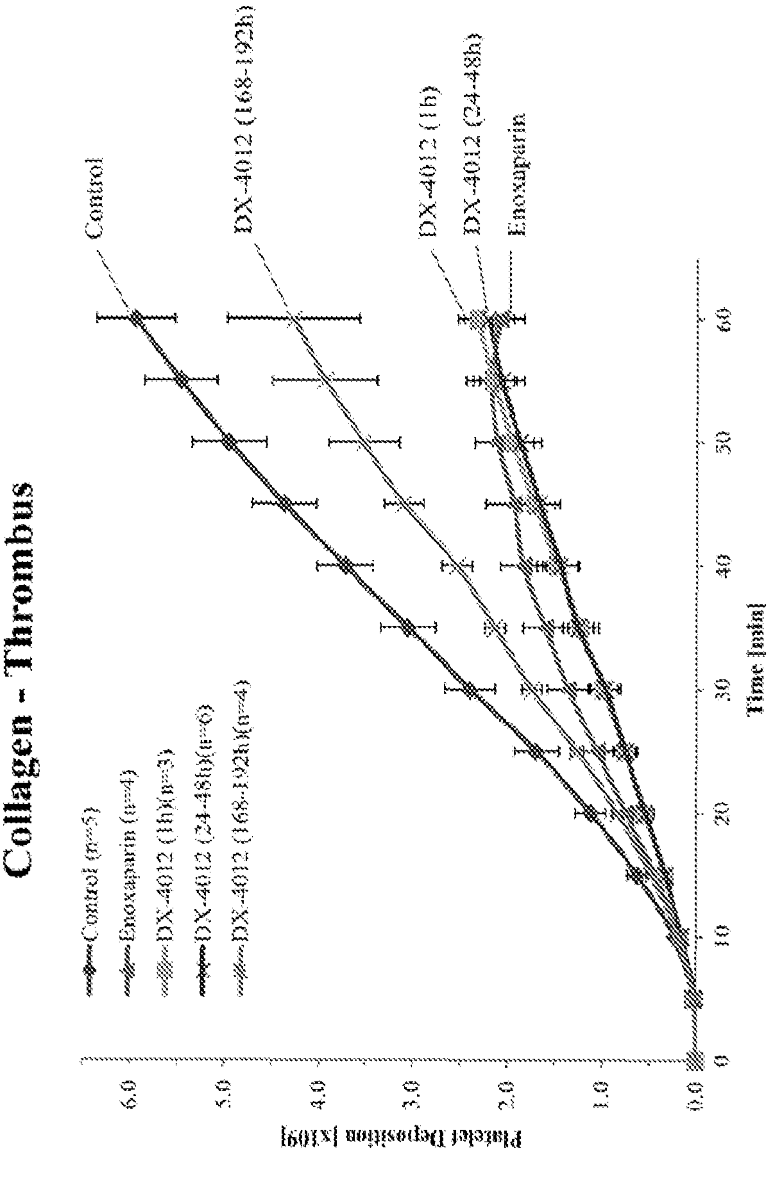
Collagen - Thrombus
Control (n=5)
Enoxaparin (n=4)
DX-4012 (1h)(n=3)
DX-4012 (24-48h)(n=6)
DX-4012 (168-192h)(n=4)
Platelet Deposition [x109]
0.0
1.0
2.0
3.0
4.0
5.0
6.0
0
10
20
30
40
50
60
Time [min]
Control
DX-4012 (168-192h)
DX-4012 (1h)
DX-4012 (24-48h)
Enoxaparin

FIGURE 13

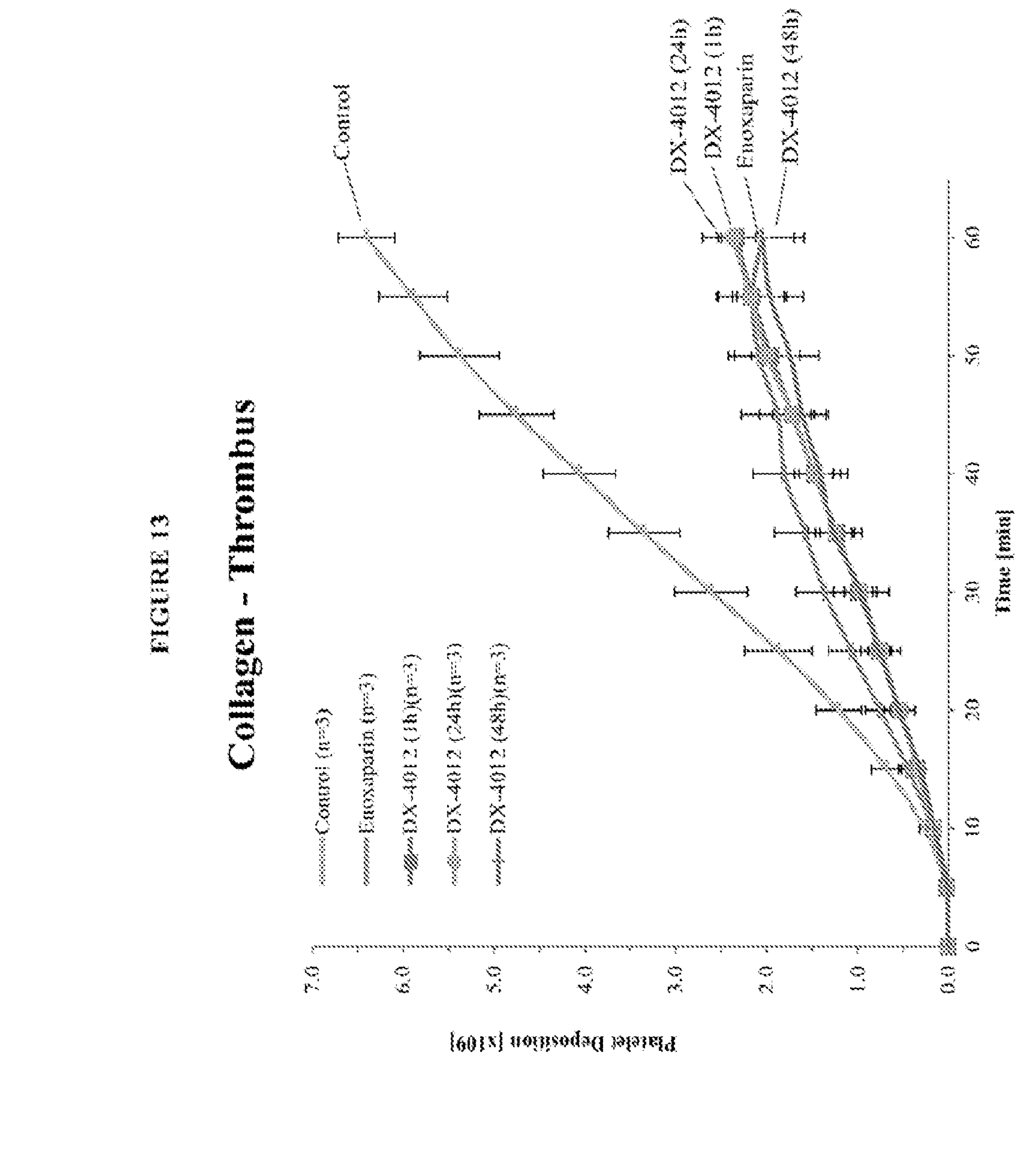
Collagen - Thrombus
Control (n=3)
Enoxaparin (n=3)
DX-4012 (1h)(n=3)
DX-4012 (24h)(n=3)
DX-4012 (48h)(n=3)
Control
DX-4012 (24h)
DX-4012 (1h)
Enoxaparin
DX-4012 (48h)
Platelet Deposition [x109]
Time [min]
0.0
1.0
2.0
3.0
4.0
5.0
6.0
7.0
0
10
20
30
40
50
60

FIGURE 14

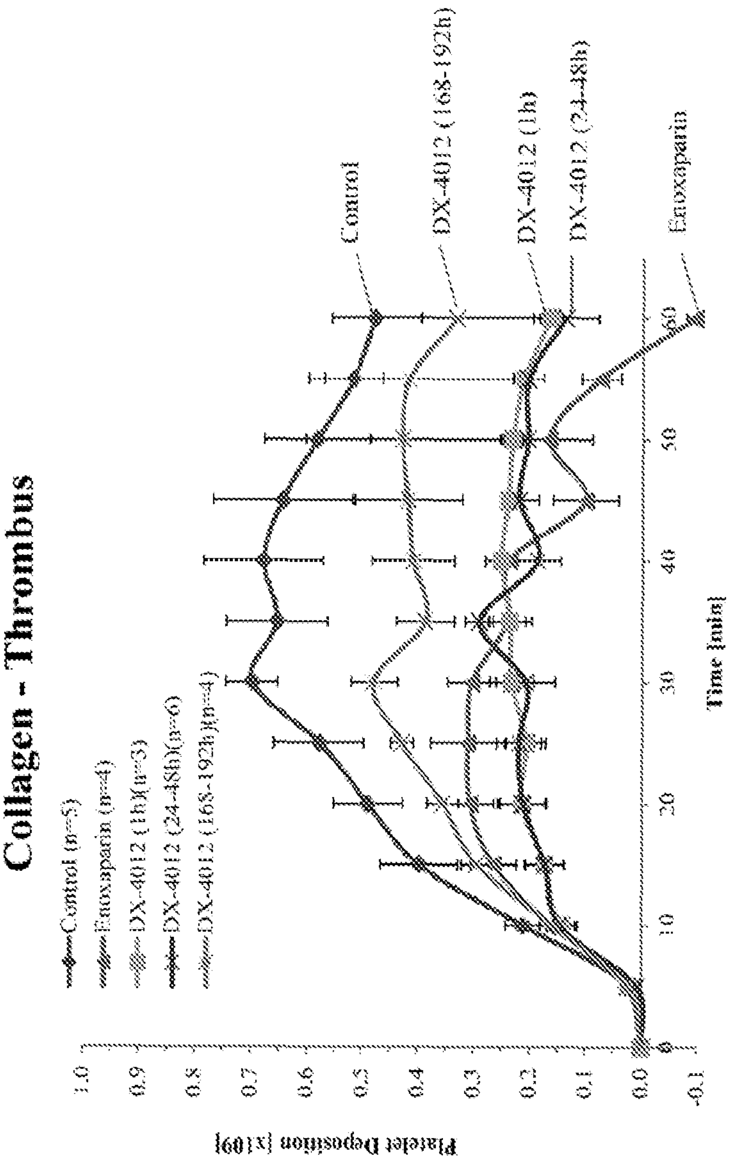
Collagen - Thrombus
Control (n=5)
Enoxaparin (n=4)
DX-4012 (1h)(n=3)
DX-4012 (24-48h)(n=6)
DX-4012 (168-192h)(n=4)
Platelet Deposition [x109]
1.0
0.9
0.8
0.7
0.6
0.5
0.4
0.3
0.2
0.1
0.0
-0.1
0
10
20
30
40
50
60
Time [min]
Control
DX-4012 (168-192h)
DX-4012 (1h)
DX-4012 (24-48h)
Enoxaparin

FIGURE 15

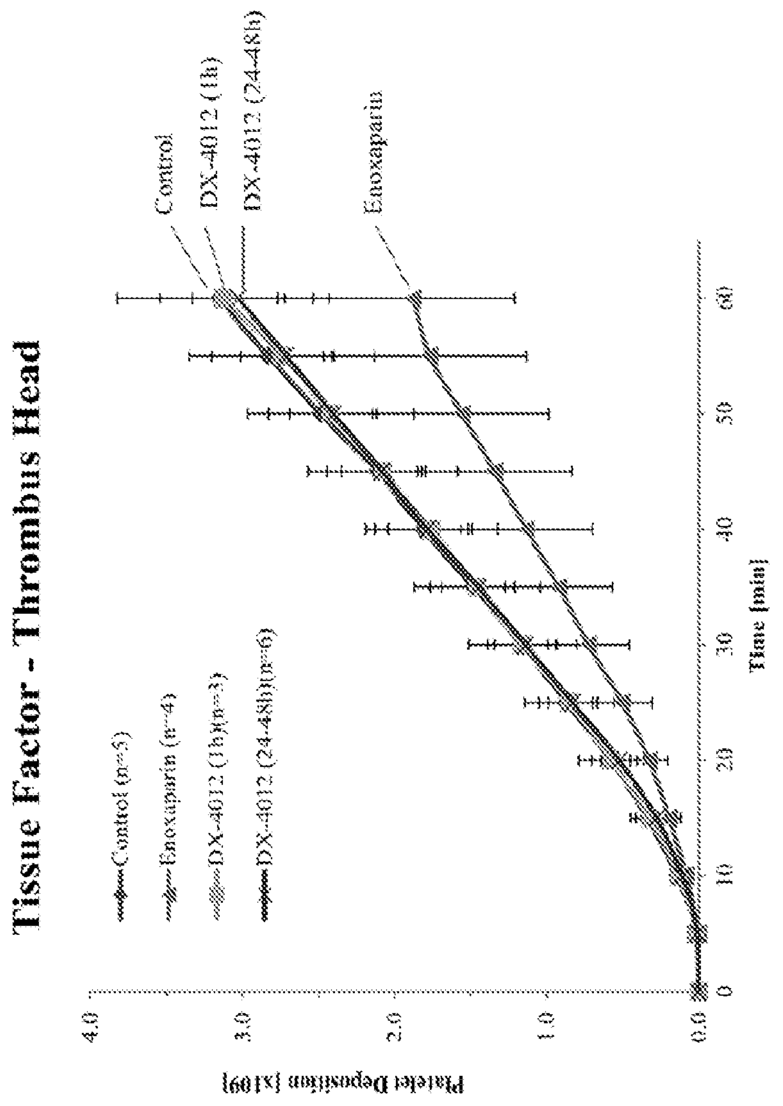
Tissue Factor - Thrombus Head
Control (n=5)
Enoxaparin (n=4)
DX-4012 (1h)(n=3)
DX-4012 (24-48h)(n=6)
Control
DX-4012 (1h)
DX-4012 (24-48h)
Enoxaparin
Platelet Deposition [x109]
Time [min]
0.0
1.0
2.0
3.0
4.0
0
10
20
30
40
50
60

A.

FIGURE 15 (Cont'd)

B.

Tissue Factor - Thrombus Tail

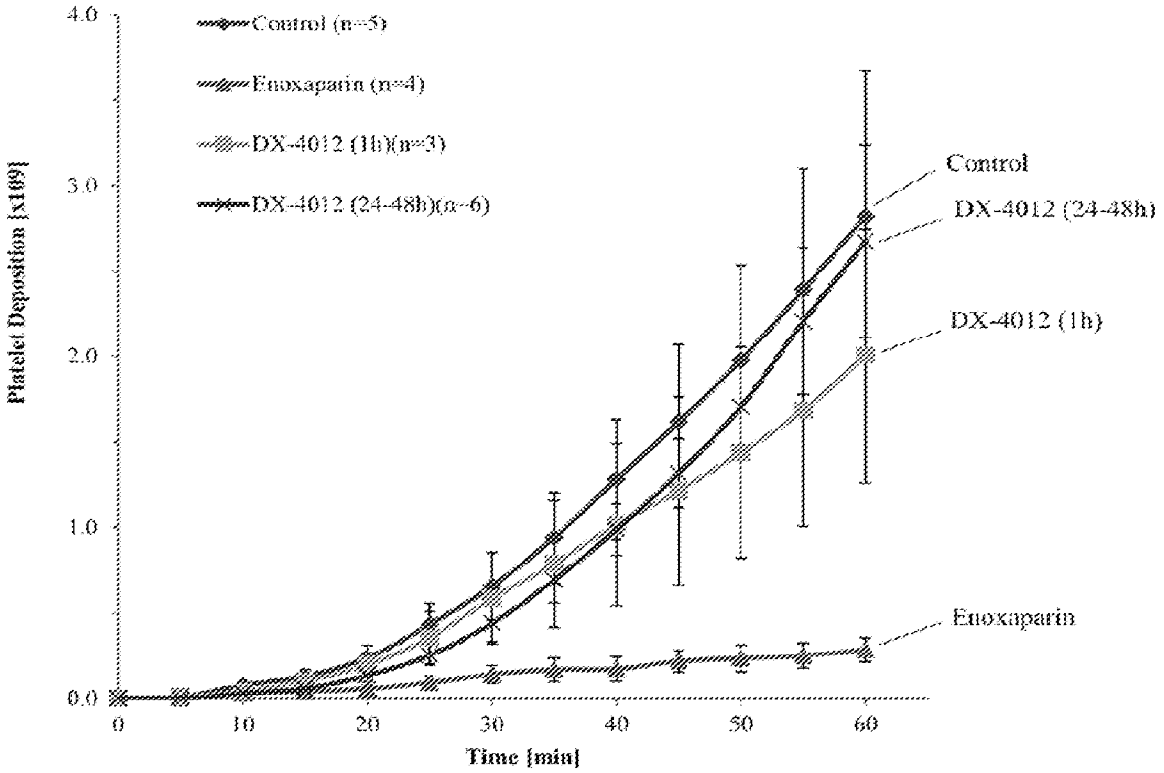

FIGURE 16

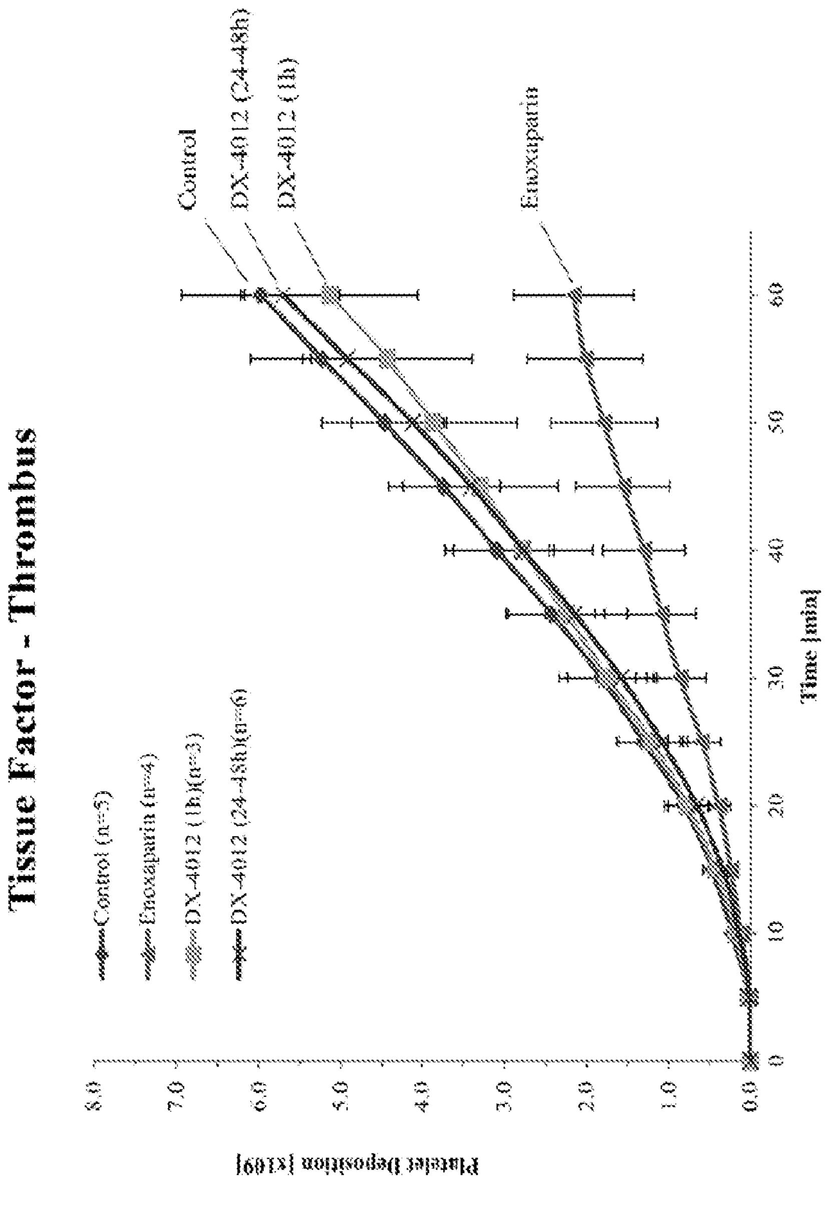
Tissue Factor - Thrombus
Control (n=5)
Enoxaparin (n=4)
DX-4012 (1h)(n=3)
DX-4012 (24-48h)(n=6)
Control
DX-4012 (24-48h)
DX-4012 (1h)
Enoxaparin
Platelet Deposition [x109]
Time [min]

FIGURE 17

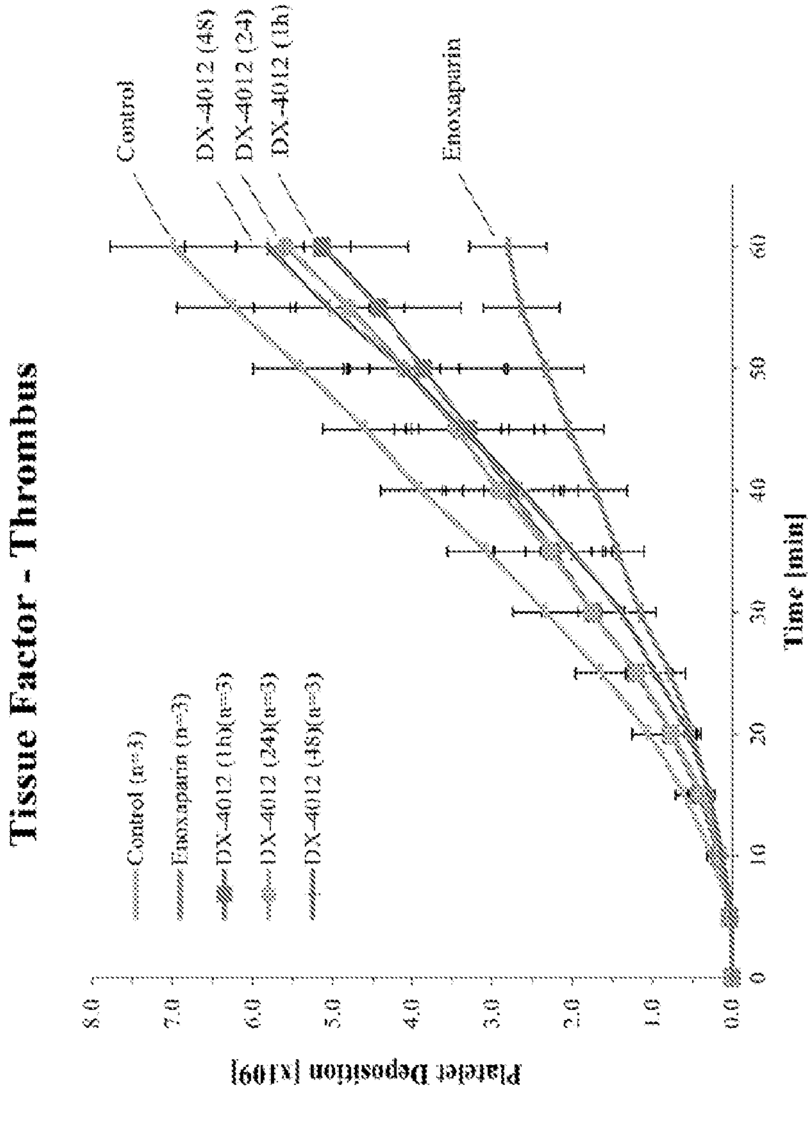
Tissue Factor - Thrombus
Control (n=3)
Enoxaparin (n=3)
DX-4012 (1h)(n=3)
DX-4012 (24)(n=3)
DX-4012 (48)(n=3)
Control
DX-4012 (48)
DX-4012 (24)
DX-4012 (1h)
Enoxaparin
Platelet Deposition [x109]
Time [min]
0.0
1.0
2.0
3.0
4.0
5.0
6.0
7.0
8.0
0
10
20
30
40
50
60

FIGURE 18

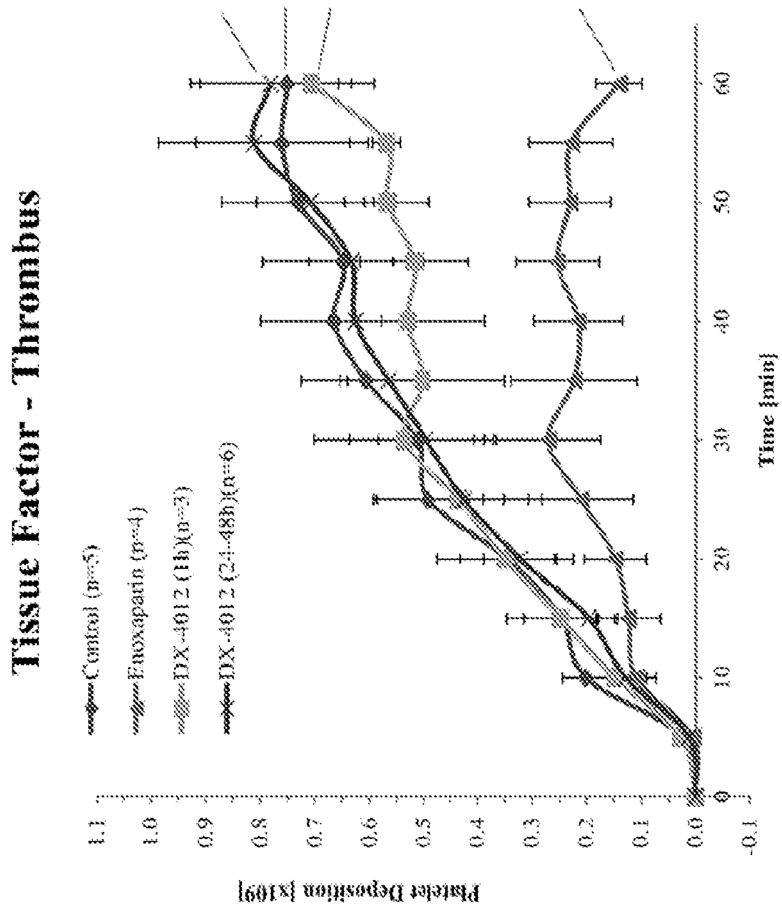
Tissue Factor - Thrombus
Control (n=5)
Enoxaparin (n=4)
DX-4012 (1h)(n=3)
DX-4012 (24-48h)(n=6)
Platelet Deposition [x109]
1.1
1.0
0.9
0.8
0.7
0.6
0.5
0.4
0.3
0.2
0.1
0.0
-0.1
0
10
20
30
40
50
60
Time [min]

FIGURE 19

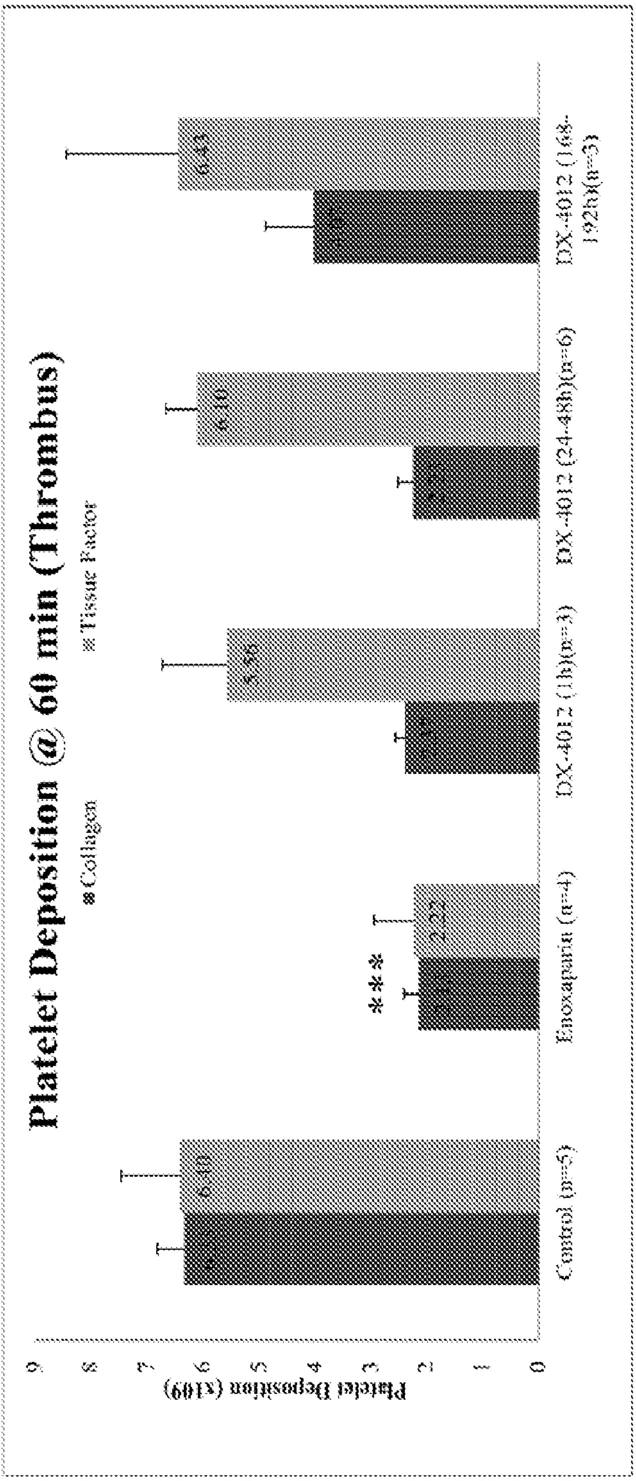
Platelet Deposition @ 60 min (Thrombus)
Collagen
Tissue Factor
Platelet Deposition (x109)
0
1
2
3
4
5
6
7
8
9
6.40
***
2.22
5.56
6.10
6.43
Control (n=5)
Enoxaparin (n=4)
DX-4012 (1h)(n=3)
DX-4012 (24-48h)(n=6)
DX-4012 (168-192h)(n=3)

A.

FIGURE 19 (Cont'd)

B.

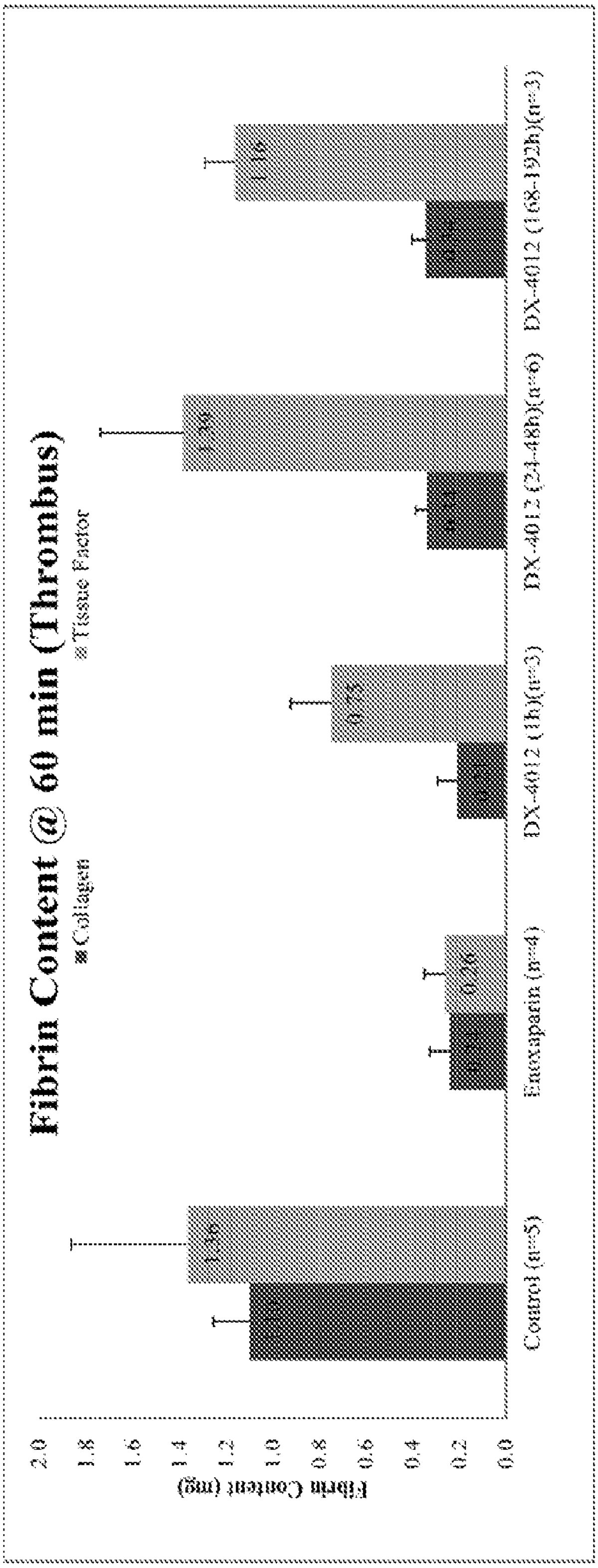

FIGURE 20

A.

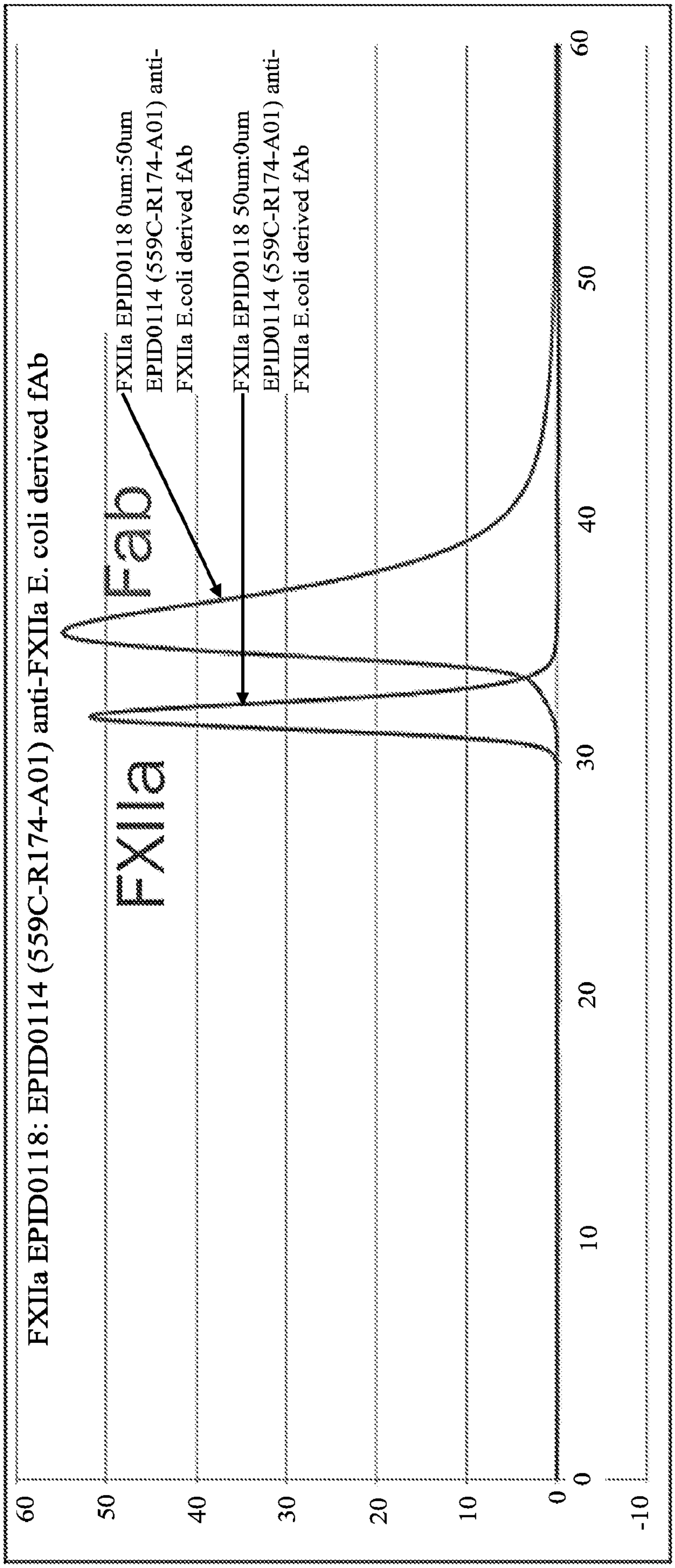
FXIIa EPID0118: EPID0114 (559C-R174-A01) anti-FXIIa E. coli derived fAb
FXIIa
Fab
FXIIa EPID0118 0um:50um EPID0114 (559C-R174-A01) anti-FXIIa E.coli derived fAb
FXIIa EPID0118 50um:0um EPID0114 (559C-R174-A01) anti-FXIIa E.coli derived fAb
60
50
40
30
20
10
0
-10
0
10
20
30
40
50
60

FIGURE 20 (Cont'd)

B.

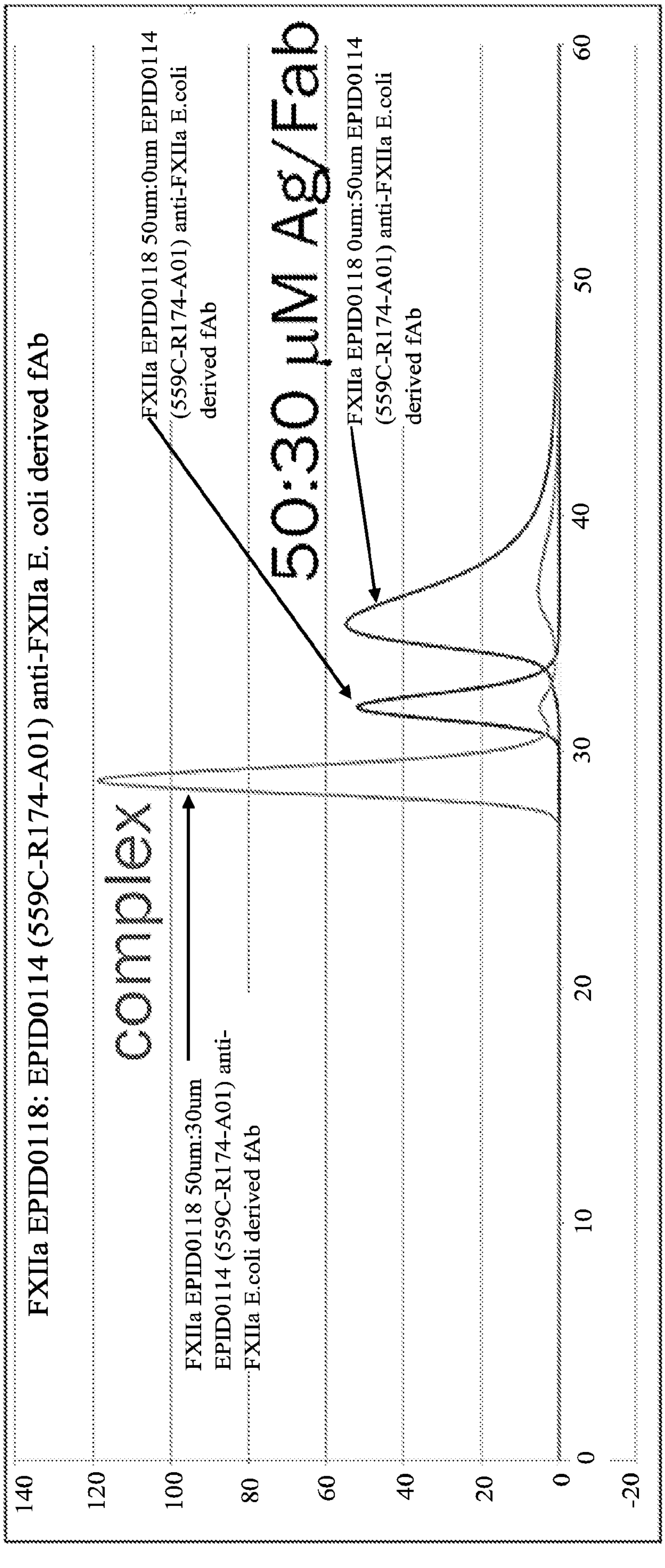

FIGURE 20 (Cont'd)

C.

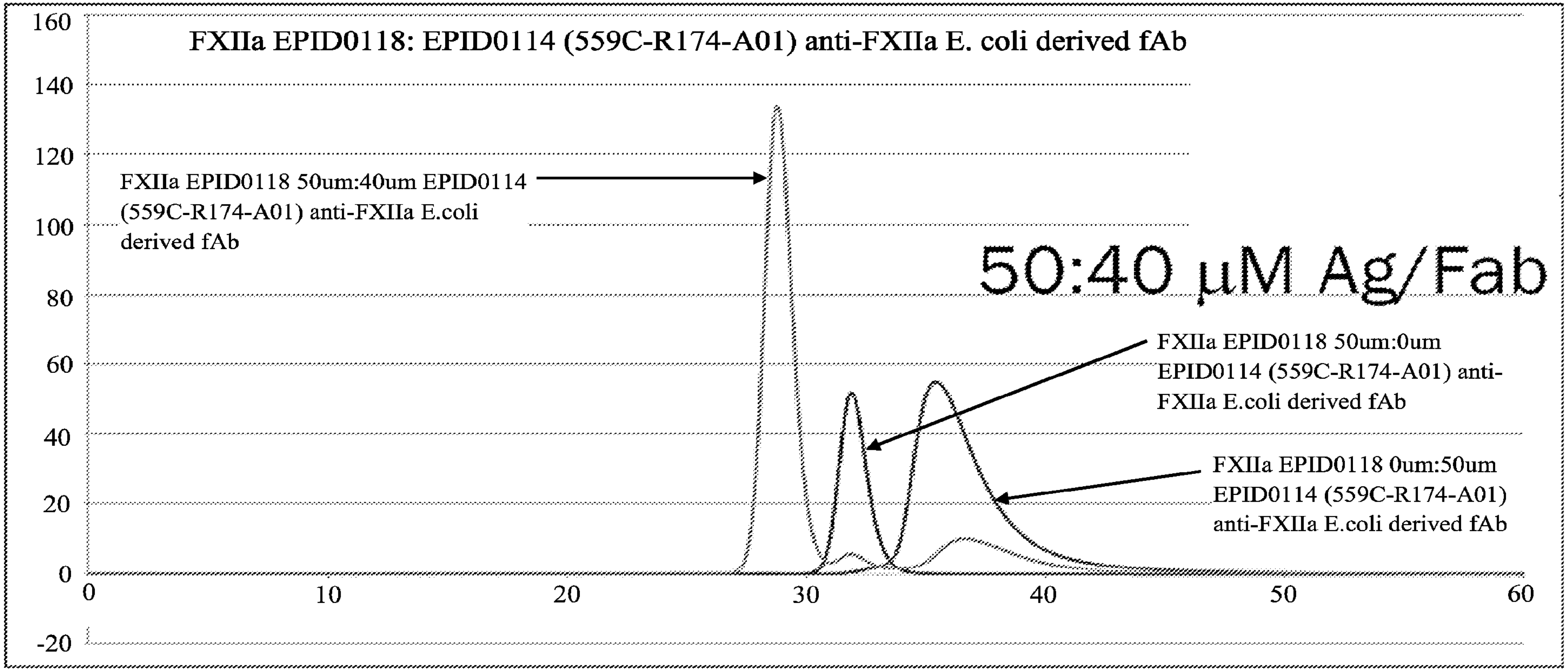

FIGURE 20 (Cont'd)

D.

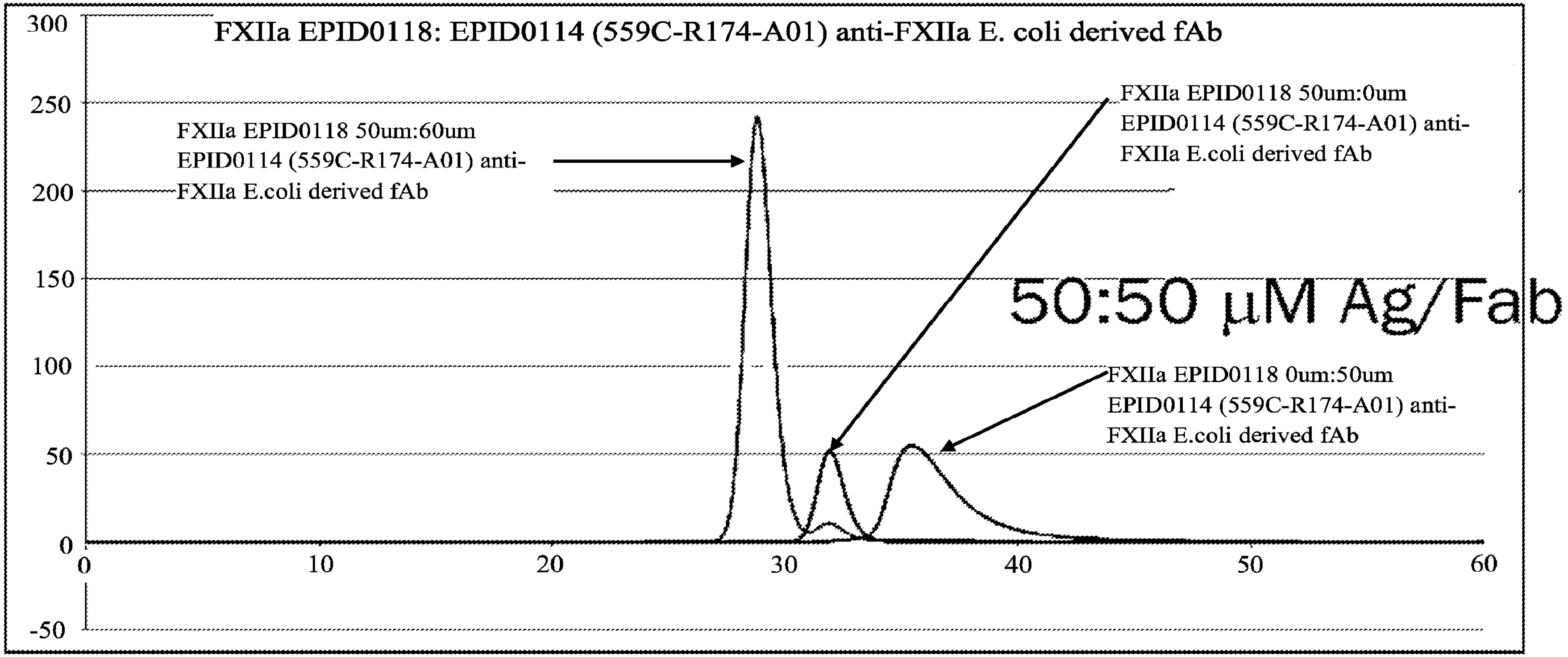

FIGURE 20 (Cont'd)

E.

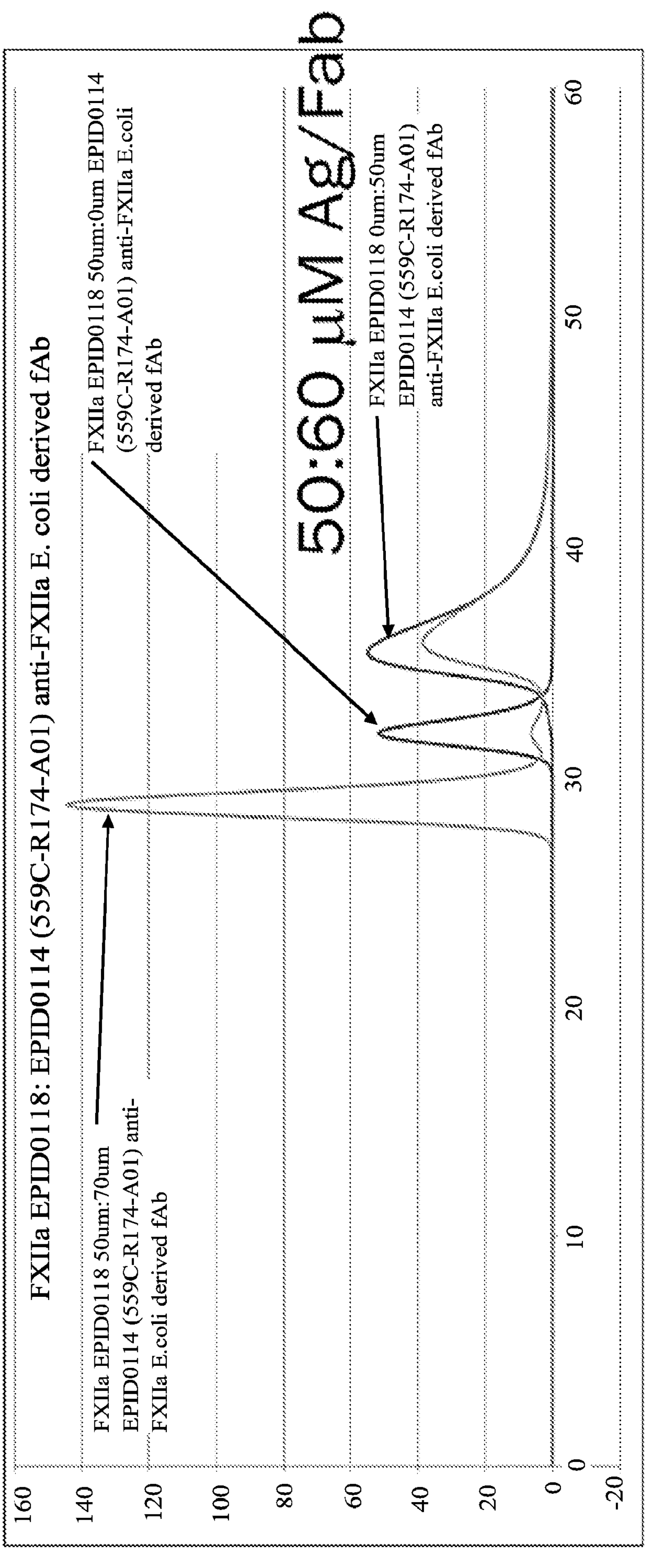

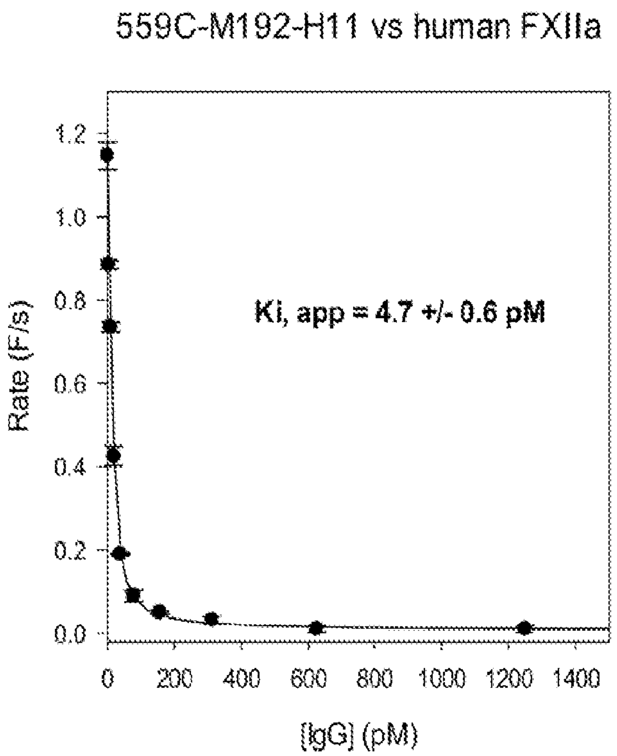
559C-M192-H11 vs human FXIIa
Ki, app = 4.7 +/- 0.6 pM
Rate (F/s)
1.2
1.0
0.8
0.6
0.4
0.2
0.0
0
200
400
600
800
1000
1200
1400
[IgG] (pM)

FIGURE 22

A.

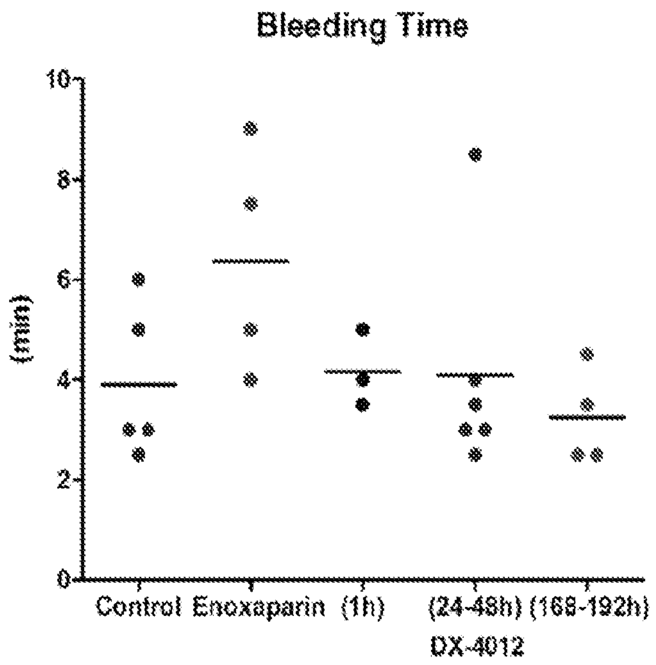
Bleeding Time
(min)
10
8
6
4
2
0
Control
Enoxaparin
(1h)
(24-48h)
(168-192h)
DX-4012

B.

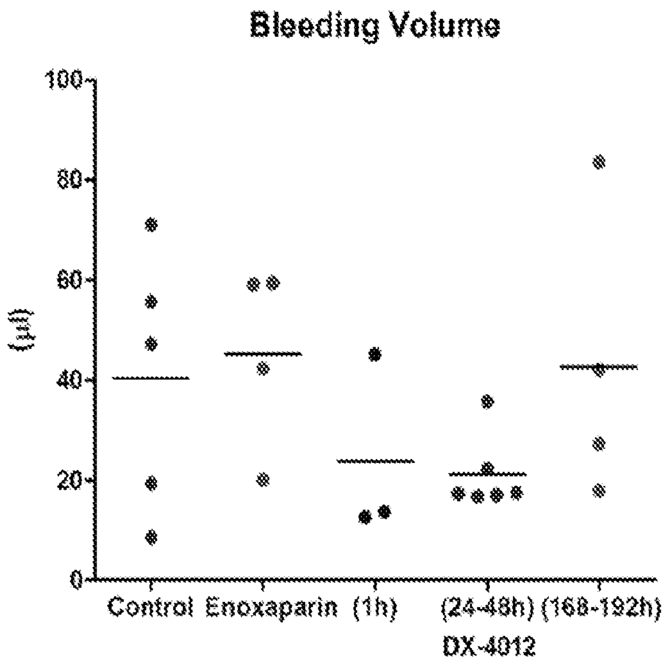
Bleeding Volume
(µl)
100
80
60
40
20
0
Control
Enoxaparin
(1h)
(24-48h)
(168-192h)
DX-4012

REFERENCES CITED IN THE DESCRIPTION

This list of references cited by the applicant is for the reader's convenience only. It does not form part of the European patent document. Even though great care has been taken in compiling the references, errors or omissions cannot be excluded and the EPO disclaims all liability in this regard.

Patent documents cited in the description

- US 62194957 **[0001]**
- US 62273657 **[0001]**
- US 62316310 **[0001]**
- WO 2013014092 A **[0003]**
- WO 9958572 A **[0031]**
- US 5500362 A **[0045]**
- GB 9901441 W **[0045]**
- GB 9809951 A **[0045]**
- US 5565332 A **[0071]**
- US 5580717 A **[0071]**
- US 5733743 A **[0071]**
- US 6265150 B **[0071]**
- WO 8704462 A **[0073]**
- US 4946778 A **[0077]**
- US 4704692 A **[0077]**
- US 4485045 A **[0096]**
- US 4544545 A **[0096]**
- US 5013556 A **[0096]**
- US 3773919 A **[0098]**
- WO 0053211 A **[0134]**
- US 5981568 A **[0134]**
- WO 9007936 A **[0138]**
- WO 9403622 A **[0138]**
- WO 9325698 A **[0138]**
- WO 9325234 A **[0138]**
- WO 9311230 A **[0138]**
- WO 9310218 A **[0138]**
- WO 9102805 A **[0138]**
- US 5219740 A **[0138]**
- US 4777127 A **[0138]**
- GB 2200651 A **[0138]**
- EP 0345242 A **[0138]**
- WO 9412649 A **[0138]**
- WO 9303769 A **[0138]**
- WO 9319191 A **[0138]**
- WO 9428938 A **[0138]**
- WO 9511984 A **[0138]**
- WO 9500655 A **[0138]**
- US 5814482 A **[0139]**
- WO 9507994 A **[0139]**
- WO 9617072 A **[0139]**
- WO 9530763 A **[0139]**
- WO 9742338 A **[0139]**
- WO 9011092 A **[0139]**
- US 5580859 A **[0139]**
- US 5422120 A **[0139]**
- WO 9513796 A **[0139]**
- WO 9423697 A **[0139]**
- WO 9114445 A **[0139]**
- EP 0524968 A **[0139]**
- US 62316310 B **[0201]**
- US 62273657 B **[0201]**
- US 62194957 B **[0201]**

Non-patent literature cited in the description

- **ESNOUF et al.** *Thrombosis and Haemostasis,* 2000, vol. 83 (6), 874-881 **[0003]**
- *Eur. J. Immunol.,* 1999, vol. 29, 2613-2624 **[0045]**
- **KABAT et al.** Sequences of Proteins of Immunological Interest. U.S. Public Health Service, National Institutes of Health, 1991 **[0046]**
- **DALL'ACQUA et al.** *J.B.C.,* 2006, vol. 281, 23514-23524 **[0046] [0047]**
- **ROBBIE et al.** *Antimicrob. Agents Chemother,* 2013, vol. 57 (12), 6147 **[0046] [0047]**
- **DALL'ACQUA et al.** *J. Immunol.,* 2002, vol. 169, 5171-5180 **[0046] [0047]**
- **J. SAMBROOK et al.** Molecular Cloning: A Laboratory Manual. Cold Spring Harbor Laboratory Press, 1989 **[0056]**
- Current Protocols in Molecular Biology. John Wiley & Sons, Inc, **[0056]**
- **KARLIN ; ALTSCHUL.** *Proc. Natl. Acad. Sci. USA,* 1990, vol. 87, 2264-68 **[0058]**
- **ALTSCHUL.** *Proc. Natl. Acad. Sci. USA,* 1993, vol. 90, 5873-77 **[0058]**
- **ALTSCHUL et al.** *J. Mol. Biol.,* 1990, vol. 215, 403-10 **[0058]**
- **ALTSCHUL et al.** *Nucleic Acids Res.,* 1997, vol. 25 (17), 3389-3402 **[0058]**
- **HARLOW ; LANE.** Antibodies: A Laboratory Manual. Cold Spring Harbor Laboratory, 1998 **[0066]**
- **KOHLER, B. ; MILSTEIN, C.** *Nature,* 1975, vol. 256, 495-497 **[0068]**
- **BUCK, D. W. et al.** *In Vitro,* 1982, vol. 18, 377-381 **[0068]**
- **WINTER et al.** *Annu. Rev. Immunol.,* 1994, vol. 12, 433-455 **[0071]**
- **MCCAFFERTY et al.** *Nature,* 1990, vol. 348, 552-553 **[0071]**

- **MORRISON et al.** *Proc. Nat. Acad. Sci.,* 1984, vol. 81, 6851 **[0073]**
- **MORRISON et al.** *Proc. Natl. Acad. Sci. USA,* 1984, vol. 81, 6851 **[0074]**
- **NEUBERGER et al.** *Nature,* 1984, vol. 312, 604 **[0074]**
- **TAKEDA et al.** *Nature,* 1984, vol. 314, 452 **[0074]**
- **QUEEN et al.** *Proc. Natl. Acad. Sci. USA,* 1989, vol. 86, 10029-10033 **[0075]**
- **HARLOW ; LANE.** Using Antibodies, a Laboratory Manual. Cold Spring Harbor Laboratory Press, 1999 **[0078]**
- **BROWN, M. et al.** *Cell,* 1987, vol. 49, 603-612 **[0085]**
- **GOSSEN, M. ; BUJARD, H.** *Proc. Natl. Acad. Sci. USA,* 1992, vol. 89, 5547-5551 **[0085]**
- **YAO, F. et al.** *Human Gene Therapy,* 1998, vol. 9, 1939-1950 **[0085]**
- **SHOCKELT, P. et al.** *Proc. Natl. Acad. Sci. USA,* 1995, vol. 92, 6522-6526 **[0085]**
- **M. BROWN et al.** *Cell,* 1987, vol. 49, 603-612 **[0086]**
- **M. GOSSEN et al.** *Natl. Acad. Sci. USA,* 1992, vol. 89, 5547-5551 **[0086]**
- **YAO et al.** *Human Gene Therapy* **[0086]**
- **GOSSEN et al.** *Natl. Acad. Sci. USA,* 1992, vol. 89, 5547-5551 **[0086]**
- **SHOCKETT et al.** *Proc. Natl. Acad. Sci. USA,* 1995, vol. 92, 6522-6526 **[0086]**
- Remington: The Science and Practice of Pharmacy. Lippincott Williams and Wilkins, 2000 **[0094]** **[0095]**
- **EPSTEIN et al.** *Proc. Natl. Acad. Sci. USA,* 1985, vol. 82, 3688 **[0096]**
- **HWANG et al.** *Proc. Natl. Acad. Sci. USA,* 1980, vol. 77, 4030 **[0096]**
- Remington, The Science and Practice of Pharmacy. Mack Publishing, 2000 **[0097]**
- **MCMILLAN, C.V. et al.** *Patient,* 2012, vol. 5 (2), 113-26 **[0121]**
- **FINDEIS et al.** *Trends Biotechnol,* 1993, vol. 11, 202 **[0135]**
- **CHIOU et al.** Gene Therapeutics: Methods And Applications Of Direct Gene Transfer. 1994 **[0135]**
- **WU et al.** *J. Biol. Chem.,* 1988, vol. 263, 621 **[0135]**
- **WU et al.** *J. Biol. Chem.,* 1994, vol. 269, 542 **[0135]**
- **ZENKE et al.** *Proc. Natl. Acad. Sci. USA,* 1990, vol. 87, 3655 **[0135]**
- **WU et al.** *J. Biol. Chem.,* 1991, vol. 266, 338 **[0135]**
- **JOLLY.** *Cancer Gene Therapy,* 1994, vol. 1, 51 **[0137]**
- **KIMURA.** *Human Gene Therapy,* 1994, vol. 5, 845 **[0137]**
- **CONNELLY.** *Human Gene Therapy,* 1995, vol. 1, 185 **[0137]**
- **KAPLITT.** *Nature Genetics,* 1994, vol. 6, 148 **[0137]**
- **CURIEL.** *Hum. Gene Ther.,* 1992, vol. 3, 147 **[0138]** **[0139]**
- **WU.** *J. Biol. Chem.,* 1989, vol. 264, 16985 **[0139]**
- **PHILIP.** *Mol. Cell. Biol.,* 1994, vol. 14, 2411 **[0139]**
- **WOFFENDIN.** *Proc. Natl. Acad. Sci.,* 1994, vol. 91, 1581 **[0139]**
- **SAMBROOK et al.** Molecular Cloning: A Laboratory Manual. Cold Spring Harbor Press, 1989 **[0149]**
- Oligonucleotide Synthesis. 1984 **[0149]**
- Methods in Molecular Biology. Humana Press **[0149]**
- Cell Biology: A Laboratory Notebook. Academic Press, 1998 **[0149]**
- Animal Cell Culture. 1987 **[0149]**
- **J. P. MATHER ; P. E. ROBERTS.** Introduction to Cell and Tissue Culture. Plenum Press, 1998 **[0149]**
- Cell and Tissue Culture: Laboratory Procedures. J. Wiley and Sons, 1993 **[0149]**
- Methods in Enzymology. Academic Press, Inc, **[0149]**
- Handbook of Experimental Immunology **[0149]**
- Gene Transfer Vectors for Mammalian Cells. 1987 **[0149]**
- Current Protocols in Molecular Biology. 1987 **[0149]**
- PCR: The Polymerase Chain Reaction. 1994 **[0149]**
- Current Protocols in Immunology. 1991 **[0149]**
- Short Protocols in Molecular Biology. Wiley and Sons, 1999 **[0149]**
- **C. A. JANEWAY ; P. TRAVERS.** *Immunobiology,* 1997 **[0149]**
- **P. FINCH.** *Antibodies,* 1997 **[0149]**
- Antibodies: a practical approach. IRL Press, 1988 **[0149]**
- Monoclonal antibodies: a practical approach. Oxford University Press, 2000 **[0149]**
- **E. HARLOW ; D. LANE.** Using antibodies: a laboratory manual. Cold Spring Harbor Laboratory Press, 1999 **[0149]**
- The Antibodies. Harwood Academic Publishers, 1995 **[0149]**

Arrowhead Pharmaceuticals Presents New Data on ARC-F12 and ARC-LPA Using DPCsqTM Subcutaneous RNAi Delivery Vehicle

Investors

Overview
Press Releases
Events & Presentations
Corporate Governance
Financials & Filings
Stock Information
Investor FAQs
Contact Us

<< Back

Arrowhead Pharmaceuticals Presents New Data on ARC-F12 and ARC-LPA Using DPCsqTM Subcutaneous RNAi Delivery Vehicle

Nov 14, 2016 at 3:00 PM EST

Arrowhead Pharmaceuticals Presents New Data on ARC-F12 and ARC-LPA Using DPCsqTM Subcutaneous RNAi Delivery Vehicle

PASADENA, Calif.--(BUSINESS WIRE)-- Arrowhead Pharmaceuticals, Inc. (NASDAQ: ARWR) today delivered oral presentations on ARC-LPA, its preclinical development program targeting lipoprotein (a), for the treatment of cardiovascular disease, and ARC-F12, its preclinical development program targeting factor XII (F12) for the prophylactic treatment of thromboembolism and hereditary angioedema, at the American Heart Association's Scientific Sessions 2016 in New Orleans. These data show advancements being made in the ARC-LPA and ARC-F12 programs and in the development of Arrowhead's proprietary DPC_{sq}^{TM}delivery vehicle designed for subcutaneous administration of RNAi therapeutics.

Chris Anzalone, Ph.D., president and CEO of Arrowhead Pharmaceuticals, said: "We have made rapid advancements to our DPC_{sq}^{TM} subcutaneous RNAi delivery platform. F12 and Lp(a) are both very attractive targets for RNAi, and the data presented today on ARC-F12 and ARC-LPA show substantial improvements in activity over previous generations. In addition, through multiple rounds of SAR studies, substantial gains in potency continue to be made by Arrowhead scientists. Data shown today are from the first and second generation triggers. Studies that are ongoing in NHPs that use our fourth generation RNAi triggers and the DPC_{sq}^{TM} platform, are achieving even higher levels of target knockdown with longer duration using lower and less frequent doses (data not shown)."

The oral poster titled, "**Targeting factor XII (F12) with a novel RNAi delivery platform as a prophylactic treatment for thromboembolism**", describes advancements in the preclinical development of ARC-F12. Key details of the presentation include the following:

- F12 is an attractive target for an RNAi therapeutic
  - It is key component at the top of the contact (intrinsic) coagulation cascade
  - It is predominantly expressed in the liver and circulates in blood
  - Inhibition is genetically validated and deficiency protects from thromboembolic disease but is not associated with either bleeding or thrombotic disorders
- Arrowhead developed RNAi triggers that gave greater than 95% reductions of serum F12 levels after a single subcutaneous injection
- Dose dependent reductions in serum F12 levels were observed with single injections of ARC-F12 of 1 mg/kg and 3 mg/kg leading to mean reductions of 86% and 96% respectively
- A statistically significant reduction (p=0.002) in thrombus weight was observed at greater than 95% F12 knockdown in a rat arterio-venous shunt model
- There was no increased bleeding risk in ARC-F12 treated mice, even with greater than 99% knockdown of F12 levels

The oral presentation titled, "**Targeting apolipoprotein(a) with a novel RNAi delivery platform as a prophylactic treatment to reduce risk of cardiovascular events in individuals with elevated lipoprotein (a)**", describes advancements in the preclinical development of ARC-LPA, which is part of a recently announced license and collaboration agreement with Amgen. Key details of the presentation include the following:

- Lipoprotein (a) [Lp(a)] is an attractive target for an RNAi therapeutic
  - Lp(a) is an independent risk factor for cardiovascular disease through its atherogenic potential
  - Higher levels of Lp(a) correlate with increased risk of cardiovascular disease
  - Lp(a) levels in humans are genetically defined and do not change significantly with diet and exercise
  - Approximately 25% of the U.S. population has greater than 30 mg/dL of Lp(a) (normal levels: 0.1 - 25 mg/dL)
  - It is predominantly expressed in the liver and circulates in blood
- Screening of LPA RNAi triggers in Lp(a) transgenic (Tg) mice identified several that induced substantial and sustained knockdown of serum Lp(a) levels
- Structure activity relationship (SAR) studies assessing structure and chemical modifications identified triggers that achieved greater than 98% maximum knockdown after a single 3 mg/kg SQ dose in Tg mice
- In NHPs, 85-90% reduction of serum Lp(a) levels was observed after three weekly 3 mg/kg SQ doses
- In an atherosclerosis model, data suggest that RNAi triggers can be effectively delivered to a fatty liver using the DPC_{sq}^{TM} platform

A copy of presentation materials will be made available on the Events and Presentations page under the Investors section of the Arrowhead website.

About ARC-F12

Arrowhead's RNAi-based candidate ARC-F12 is in preclinical development as a potential treatment for factor XII (F12) mediated diseases. Arrowhead sees clear unmet need in hereditary angioedema (HAE) and thromboembolic diseases. The biology of factor 12 as part of the coagulation cascade and the kinin-kallikrein system suggest that its reduction through RNAi may present opportunities in both disease areas. The company is currently conducting studies in order to advance ARC-F12 into clinical trials.

About ARC-LPA

Arrowhead's RNAi-based candidate ARC-LPA is in preclinical development as a potential treatment for cardiovascular diseases. ARC-LPA is designed to reduce production of apolipoprotein(a), a key component of lipoprotein(a), or Lp(a). Lp(a) levels in humans are genetically defined and higher levels correlate with increased risk of cardiovascular diseases, independent of cholesterol and LDL levels. ARC-LPA is Arrowhead's first drug candidate to use a subcutaneously administered delivery construct.

About Arrowhead Pharmaceuticals

Arrowhead Pharmaceuticals develops medicines that treat intractable diseases by silencing the genes that cause them. Using a broad portfolio of RNA chemistries and efficient modes of delivery, Arrowhead therapies trigger the RNA interference mechanism to induce rapid, deep, and durable knockdown of target genes. RNA interference, or RNAi, is a mechanism present in living cells that inhibits the expression of a specific gene, thereby affecting the production of a specific protein. Arrowhead's RNAi-based therapeutics leverage this natural pathway of gene silencing. The company's pipeline includes ARC-520 and ARC-521 for chronic hepatitis B virus infection, ARC-AAT for liver disease associated with alpha-1 antitrypsin deficiency, ARC-F12 for hereditary angioedema and thromboembolic disorders, ARC-LPA for cardiovascular disease, and ARC-HIF2 for renal cell carcinoma.

For more information, please visit www.arrowheadpharma.com, or follow us on Twitter @ArrowheadPharma. To be added to the Company's email list and receive news directly, please visit http://ir.arrowheadpharma.com/alerts.cfm.

Safe Harbor Statement under the Private Securities Litigation Reform Act:

This news release contains forward-looking statements within the meaning of the "safe harbor" provisions of the Private Securities Litigation Reform Act of 1995. These statements are based upon our current expectations and speak only as of the date hereof. Our actual results may differ materially and adversely from those expressed in any forward-looking statements as a result of various factors and uncertainties, including the safety and efficacy of our product candidates, the duration and impact of regulatory delays in our clinical programs, our ability to finance our operations, the future success of our scientific studies, our ability to successfully develop drug candidates, the timing for starting and completing clinical trials, rapid technological change in our markets, and the enforcement of our intellectual property rights. Our most recent Annual Report on Form 10-K and subsequent Quarterly Reports on Form 10-Q discuss some of the important risk factors that may affect our business, results of operations and financial condition. We assume no obligation to update or revise forward-looking statements to reflect new events or circumstances.

DYNAMIC POLYCONJUGATES is a trademark of Arrowhead Pharmaceuticals, Inc.

Source: Arrowhead Pharmaceuticals, Inc.

View source version on businesswire.com: http://www.businesswire.com/news/home/20161114005602/en/

Arrowhead Pharmaceuticals, Inc.
Vince Anzalone, CFA
626-304-3400
ir@arrowheadpharma.com
or
Investor Relations:
The Trout Group
Chad Rubin
646-378-2947
ir@arrowheadpharma.com
or
Media:
Russo Partners
Matt Middleman, M.D.
212-845-4272
matt.middleman@russopartnersllc.com

Source: Arrowhead Pharmaceuticals, Inc.

News Provided by Acquire Media

$36.81
-0.11 (-0.30%)
NASDAQ (ARWR)
10:58 AM EDT - 06.29.23
Data Provided by Refinitiv. Minimum 15 minutes delayed.

Shareholder Tools

Email Alerts Subscription
Download Library
RSS News Feeds
Print
Share
Search Investors

Copyright © Arrowhead Pharmaceuticals, Inc.
WEBSITE DESIGN BY MENTUS

Factor XIIa

About KalVista

Products & Pipeline

For Patients

- Clinical Studies
- HAE Resources
- Sebetralstat
- **Factor XIIa**
- DME Resources

For Healthcare Providers

For Investors

We recently introduced an oral Factor XIIa (FXIIa) inhibitor program which we believe represents the next generation of therapies that may further improve the treatment of HAE for patients.

There is a strong scientific rationale and positive clinical evidence for FXIIa inhibition in HAE prophylaxis. We believe that inhibition of Factor XIIa can be designed to block the most upstream mechanism in the contact system. Clinical studies of Factor XIIa antibodies have demonstrated efficacy in preventing HAE attacks, and there are no known safety implications of long-term inhibition of this enzyme.

While injectable Factor XIIa antibody therapies are currently in clinical studies for HAE prophylaxis and other indications, developing oral Factor XIIa inhibitors has been a significant scientific challenge, and our newly announced program represents a major breakthrough in this area. Our internal research team has discovered multiple series of low nanomolar potency Factor XIIa inhibitors with high degrees of selectivity and oral bioavailability. We are pursuing comprehensive IP protection for this advanced medicinal chemistry program that is currently in lead optimization. We anticipate advancing the first candidate in this program to clinical studies in 2023.

+1 (857) 999 0075
info@kalvista.com
medinfo@kalvista.com

Copyright © 2023 | All rights reserved.

Press Release Details

BioCryst Announces FDA Approval of ORLADEYO™ (berotralstat), First Oral, Once-daily Therapy to Prevent Attacks in Hereditary Angioedema Patients

December 3, 2020 PDF Version

—Significant and sustained reduction in HAE attacks—

—Oral, once-daily prophylactic option enables HAE patients to reduce burden of therapy[1] —

—ORLADEYO approved for adult and pediatric patients 12 years and older—

RESEARCH TRIANGLE PARK, N.C., Dec. 03, 2020 (GLOBE NEWSWIRE) -- BioCryst Pharmaceuticals, Inc. (Nasdaq: BCRX) today announced that the U.S. Food and Drug Administration (FDA) has approved oral, once-daily ORLADEYO™ (berotralstat) for prophylaxis to prevent attacks of hereditary angioedema (HAE) in adults and pediatric patients 12 years and older.

"ORLADEYO offers people with HAE and their physicians the first orally administered non-steroidal option for preventing HAE attacks and represents an important and welcome step in making more treatment options available to physicians and patients," said Anthony J. Castaldo, president and chief executive officer of the US Hereditary Angioedema Association (HAEA).

In the pivotal Phase 3 APeX-2 trial, ORLADEYO significantly reduced attacks at 24 weeks, and this reduction was sustained through 48 weeks. HAE patients who completed 48 weeks of treatment (150 mg) saw reductions in their HAE attack rates, from a mean of 2.9 attacks per month at baseline to a mean of 1.0 attacks per month after 48 weeks of therapy. In the long-term open label APeX-S trial, patients completing 48 weeks of therapy (150 mg) had a mean attack rate of 0.8 attacks per month.

ORLADEYO was safe and well tolerated in both trials. The most frequently reported adverse reactions in patients receiving ORLADEYO compared with placebo were gastrointestinal reactions. These reactions generally occurred early after initiation of treatment with ORLADEYO, became less frequent with time and typically self-resolved.

"Patients and physicians acknowledge that HAE treatments can add a burden to patients' lives. As an oral, once-daily option, ORLADEYO can provide significant attack reduction and lessen the burden associated with injections and infusions," said Marc Riedl, M.D., professor of medicine and clinical director, U.S. Hereditary Angioedema Association Center at the University of California, San Diego, and an investigator in the APeX-2 trial.

"With this new treatment option, physicians and patients can continue to have collaborative discussions to choose the treatment that meets each patient's needs, life circumstances and preferences," Riedl added.

HAE patients note a significant treatment burden associated with existing prophylactic therapy. In addition to reducing HAE attack rate, data from APeX-2 show that patients reported meaningful improvements in both quality of life and overall patient-reported satisfaction, and significant reductions in their monthly use of standard of care on-demand medicine, while taking oral, once-daily ORLADEYO (150 mg).[2,3]

"The FDA approval of ORLADEYO fulfills a promise BioCryst made to HAE patients that we were committed to helping them achieve the dream of an oral, once-daily medicine to prevent and reduce the burden of their attacks," said Jon Stonehouse, president and chief executive officer of BioCryst.

"Thank you to the HAE patients who participated in our clinical trials, to the investigators around the world who conducted these trials, to the HAEA for their patient advocacy and to our employees who never forgot that patients were waiting. We will stay focused on enabling access and providing personalized support to HAE patients and physicians," Stonehouse added.

Commitment to Patient Access

BioCryst is committed to supporting HAE patients taking ORLADEYO through a new program designed to streamline access to therapy. Through EMPOWER Patient Services, each HAE patient and their healthcare provider will have a single point of contact for access to ORLADEYO. A dedicated care coordinator will support access for each patient with comprehensive financial support tools and reimbursement support.

EMPOWER Patients Services is administered by Optime Care Inc., the exclusive specialty pharmacy provider for ORLADEYO. Physicians can begin writing prescriptions for ORLADEYO immediately, with direct to patient shipments from Optime Care expected to begin by the end of December 2020.

Additional information is available at www.ORLADEYO.com and 1-866-5-EMPOWER (1-866-536-7693).

About ORLADEYO™ (berotralstat)
ORLADEYO™ (berotralstat) is the first and only oral therapy designed specifically to prevent attacks of hereditary angioedema (HAE) in adults and pediatric patients 12 years and older. One capsule of ORLADEYO per day works to prevent HAE attacks by decreasing the activity of plasma kallikrein.

U.S. Indication and Important Safety Information

INDICATION
ORLADEYO™ (berotralstat) is a plasma kallikrein inhibitor indicated for prophylaxis to prevent attacks of hereditary angioedema (HAE) in adults and pediatric patients 12 years and older.

Limitations of use
The safety and effectiveness of ORLADEYO for the treatment of acute HAE attacks have not been established. ORLADEYO should not be used for the treatment of acute HAE attacks. Additional doses or dosages of ORLADEYO higher than 150 mg once daily are not recommended due to the potential for QT prolongation.

IMPORTANT SAFETY INFORMATION
An increase in QT prolongation was observed at dosages higher than the recommended 150 mg once-daily dosage and was concentration dependent.

The most common adverse reactions (≥10% and higher than placebo) in patients receiving ORLADEYO were abdominal pain, vomiting, diarrhea, back pain, and gastroesophageal reflux disease.

A reduced dosage of 110 mg taken orally once daily with food is recommended in patients with moderate or severe hepatic impairment (Child-Pugh B or C) and in patients taking chronically administered P-glycoprotein (P-gp) or breast cancer resistance protein (BCRP) inhibitors (eg, cyclosporine).

Berotralstat is a substrate of P-gp and BCRP. P-gp inducers (eg, rifampin, St. John's wort) may decrease berotralstat plasma concentration, leading to reduced efficacy of ORLADEYO. The use of P-gp inducers is not recommended with ORLADEYO.

ORLADEYO at a dose of 150 mg is a moderate inhibitor of CYP2D6 and CYP3A4. For concomitant medications with a narrow therapeutic index that are predominantly metabolized by CYP2D6 or CYP3A4, appropriate monitoring and dose titration is recommended. ORLADEYO at a dose of 300 mg is a P-gp inhibitor. Appropriate monitoring and dose titration is recommended for P-gp substrates (eg, digoxin) when coadministering with ORLADEYO.

The safety and effectiveness of ORLADEYO in pediatric patients <12 years of age have not been established.

There are insufficient data available to inform drug-related risks with ORLADEYO use in pregnancy. There are no data on the presence of berotralstat in human milk, its effects on the breastfed infant, or its effects on milk production.

To report SUSPECTED ADVERSE REACTIONS, contact BioCryst Pharmaceuticals, Inc. at 1-833-633-2279 or FDA at 1-800-FDA-1088 or www.fda.gov/medwatch.

Please see full Prescribing Information.

About BioCryst Pharmaceuticals
BioCryst Pharmaceuticals discovers novel, oral, small-molecule medicines that treat rare diseases in which significant unmet medical needs exist and an enzyme plays a key role in the biological pathway of the disease. Oral, once-daily ORLADEYO™ (berotralstat) is approved in the United States for the prevention of HAE attacks in adults and pediatric patients 12 years and older, and under regulatory review for approval in Japan and the European Union. BioCryst has several ongoing development programs including, an oral Factor D inhibitor for the treatment of complement-mediated diseases, galidesivir, a potential treatment for COVID-19, Marburg virus disease and Yellow Fever, and BCX9250, an ALK-2 inhibitor for the treatment of fibrodysplasia ossificans progressiva. RAPIVAB® (peramivir injection), a viral neuraminidase inhibitor for the treatment of influenza, is BioCryst's first approved product and has received regulatory approval in the U.S., Canada, Australia, Japan, Taiwan, Korea and the European Union. Post-marketing commitments for RAPIVAB are ongoing. For more information, please visit the Company's website at www.biocryst.com.

Forward-Looking Statements

This press release contains forward-looking statements, including statements regarding BioCryst's plans and expectations for ORLADEYO. These statements involve known and unknown risks, uncertainties and other factors which may cause BioCryst's actual results, performance or achievements to be materially different from any future results, performances or achievements expressed or implied by the forward-looking statements. These statements reflect our current views with respect to future events and are based on assumptions and are subject to risks and uncertainties. Given these uncertainties, you should not place undue reliance on these forward-looking statements. Some of the factors that could affect the forward-looking statements contained herein include: the ongoing COVID-19 pandemic, which could create challenges in all aspects of BioCryst's business, including without limitation delays, stoppages, difficulties and increased expenses with respect to BioCryst's and its partners' development, regulatory processes and supply chains, negatively impact BioCryst's ability to access the capital or credit markets to finance its operations, or have the effect of heightening many of the risks described below or in the documents BioCryst files periodically with the Securities and Exchange Commission; BioCryst's ability to successfully implement its commercialization plans for, and to commercialize, ORLADEYO, which could take longer or be more expensive than planned; the commercial viability of ORLADEYO, including its ability to achieve market acceptance; the FDA, EMA, PMDA or other applicable regulatory agency may require additional studies beyond the studies planned for product candidates, including to support the continued commercialization of ORLADEYO, may not provide regulatory clearances which may result in delay of planned clinical trials, may impose certain restrictions, warnings, or other requirements on product candidates, including ORLADEYO, may impose a clinical hold with respect to such product candidates, or may withhold or withdraw market approval for such product candidates; BioCryst's ability to successfully manage its growth and compete effectively; risks related to the international expansion of BioCryst's business; and actual financial results may not be consistent with expectations, including that 2020 operating expenses and cash usage may not be within management's expected ranges. Please refer to the documents BioCryst files periodically with the Securities and Exchange Commission, specifically BioCryst's most recent Annual Report on Form 10-K, Quarterly Reports on Form 10-Q, and Current Reports on Form 8-K, all of which identify important factors that could cause the actual results to differ materially from those contained in BioCryst's forward-looking statements.

BCRXW

Contacts:
John Bluth
+1 919 859 7910
jbluth@biocryst.com

Catherine Collier Kyroulis
+1 917 886 5586
ckyroulis@biocryst.com

---

[1] Radojicic, C, *et al. Patient Perspectives on the Treatment Burden of Injectable Medication Administration for Hereditary Angioedema. Annual Scientific Meeting of the American College of Allergy, Asthma & Immunology;* ***Poster 160, Nov (2020)***

[2] Johnston, D.T., *et al. Berotralstat Improves Patient-Reported Quality of Life Through 48 Weeks in the Phase 3 APeX-2 Trial; Annual Scientific Meeting of the American College of Allergy, Asthma & Immunology;* ***Poster 154, Nov (2020)***

[3] Jacobs, J, *et al. Berotralstat Positively Impacts Patient-Reported Satisfaction: Results from the Phase 3 APeX-2 trial; Annual Scientific Meeting of the American College of Allergy, Asthma & Immunology;* ***Poster 158, Nov (2020)***

A photo accompanying this announcement is available at https://www.globenewswire.com/NewsRoom/AttachmentNg/bf57a925-5da4-4c72-8a93-02e9c4308123

© BioCryst Pharmaceuticals, Inc. All Rights Reserved. 2023

Sebetralstat for HAE

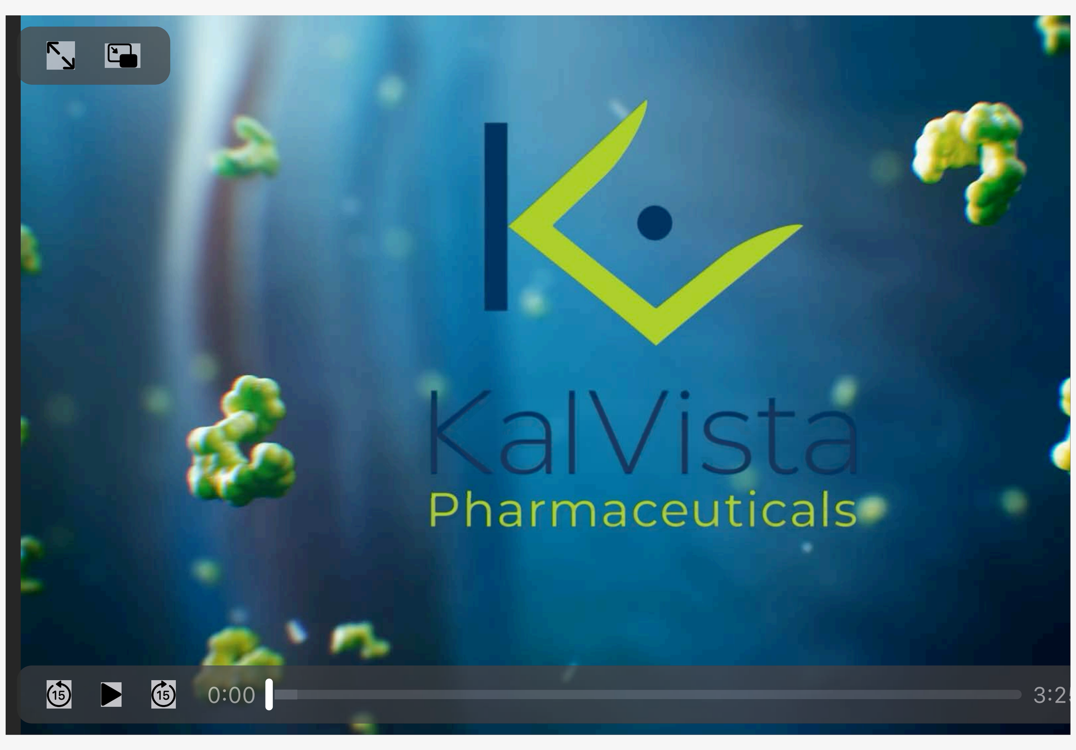

Sebetralstat is an oral plasma kallikrein inhibitor and the most advanced compound in our portfolio of candidates for the treatment of hereditary angioedema (HAE). Early treatment of HAE attacks has been shown to be key in maximizing treatment efficacy. Dosing with approved injectable treatments is often delayed undermining treatment outcomes. As a result of its straightforward oral administration combined with its rapid uptake and encouraging safety profile, sebetralstat has the potential to offer HAE patients an option to treat attacks at the earliest stages, before the symptoms develop more fully.

In March, 2022 we announced the initiation of the sebetralstat Phase 3 KONFIDENT clinical trial, evaluating the safety and efficacy of sebetralstat as an on-demand therapy for HAE attacks. KONFIDENT is a worldwide clinical study being conducted at approximately 60 sites in 20 countries. The trial is intended to enroll a minimum of 84 HAE adolescent and adult patients who will complete treatment of three attacks: one each with 300 mg sebetralstat, 600 mg sebetralstat and placebo in a double-blinded, randomized sequence. The primary endpoint of the trial is time to the beginning of symptom relief, evaluated on a Patient Global Impression of Change (PGI-C) scale, and additional endpoints will evaluate other measures of patient response and attack progression, as well as safety. We anticipate data from this trial in the second half of 2023 and, based upon the results of the trial, we expect to be able to file a NDA with the US FDA in the first half of 2024.

More information on the KONFIDENT study can be found at: **www.konfidentstudy.com**

In February 2021, we reported positive results for a Phase 2 clinical trial demonstrating statistically and clinically significant responses for sebetralstat as an oral on-demand treatment for HAE attacks.

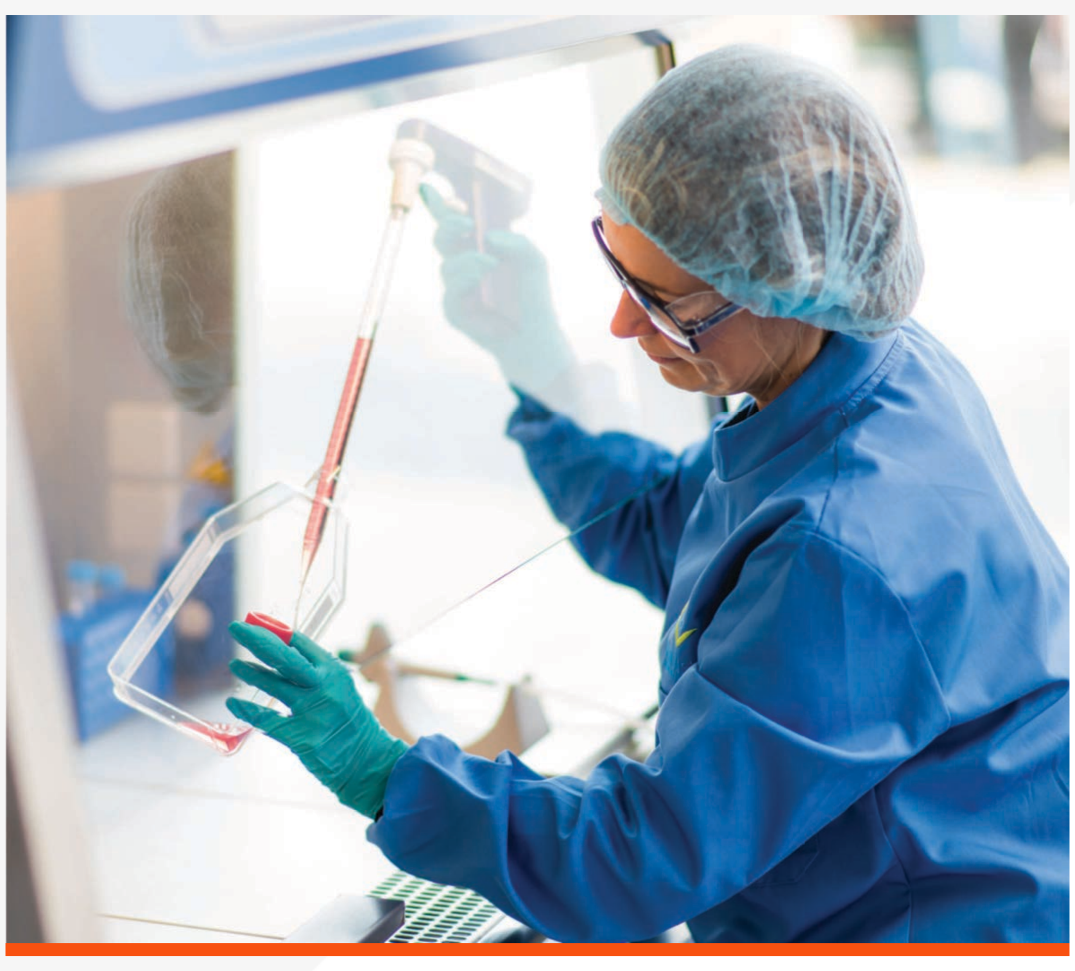

The sebetralstat Phase 2 was a randomized, double-blind, placebo-controlled, crossover clinical trial evaluating the efficacy and safety of sebetralstat as an on-demand treatment for hereditary angioedema (HAE) attacks. The trial completed 53 adult HAE patients from 25 clinical sites in the United States and Europe. The trial included type 1 and type 2 HAE patients who had three attacks in 90 days prior to enrollment. During the first part of the two-part trial, patients received a single, open label 600 mg dose of sebetralstat to evaluate pharmacokinetic and pharmacodynamic properties. All patients then entered part two of the trial, which was a double-blind investigation to assess the efficacy of sebetralstat compared to placebo in a two-attack, crossover design. During part two of the trial, patients took a single dose of 600 mg of sebetralstat or placebo within one hour of the start of the first attack. The second attack was dosed with the alternative crossover treatment. Patients were able to use their conventional rescue treatment, as required.

Topline Phase 2 Results

- Time to use of conventional treatment within 12 h of study drug administration was significantly longer with sebetralstat versus placebo; (p=0·0010)
- Sebetralstat significantly reduced time to onset of symptom relief (p=<0.0001) on the Patient Global Impression of Change scale (PGI-C), with a median time of 1.6 hours versus 9 hours for attacks treated with placebo
- Time to conventional attack treatment use or worsening in severity by 1 level or more on the PGI-S, whichever came first, within 12 h of study administration was significantly longer after treatment with sebetralstat than after treatment with placebo (p<0.001)
- The median time to attack resolution, defined as a PGI-S rating of "none" within 24 h after study drug administration, was significantly shorter with sebetralstat than with placebo (p=0·0021)
- Sebetralstat was well-tolerated and no serious adverse events were reported. The proportion of drug-related treatment-emergent adverse events was similar in attacks treated with sebetralstat and placebo

+1 (857) 999 0075
info@kalvista.com
medinfo@kalvista.com

Copyright © 2024 | All rights reserved.

News Release

About KalVista
Products & Pipeline
For Patients
For Healthcare Providers
For Investors
Annual Meeting
Stock Information
Stock Quote & Chart
Historic Price Lookup
Investment Calculator
News Releases
Event Calendar
Presentations
SEC Filings
Analyst Coverage
Corporate Governance

KalVista Pharmaceuticals Announces Termination of KVD824 Phase 2 KOMPLETE Trial for Prophylactic Treatment of Hereditary Angioedema

October 4, 2022 at 6:30 AM EDT

PDF Version

- Safety signals observed will not yield targeted product profile –

- No impact on Sebetralstat expected safety profile or Phase 3 KONFIDENT trial –

- Oral Factor XIIa program to become primary focus for HAE prophylaxis -

CAMBRIDGE, Mass. & SALISBURY, England--(BUSINESS WIRE)--Oct. 4, 2022-- KalVista Pharmaceuticals, Inc. (NASDAQ: KALV), a clinical stage pharmaceutical company focused on the discovery, development, and commercialization of oral, small molecule protease inhibitors, today announced that it has terminated the KOMPLETE phase 2 clinical trial for KVD824 for the prevention of attacks in people with hereditary angioedema (HAE). This decision was based on the observation of liver enzyme (ALT/AST) elevations in multiple patients in all treatment groups of the trial. No patients had concomitant elevation of bilirubin levels and all were asymptomatic.

"The health and safety of participants in our clinical trials is of utmost importance to us," said Andrew Crockett, Chief Executive Officer of KalVista. "We made the difficult decision to terminate KOMPLETE because we concluded that the emerging safety profile of the current formulation will not meet our requirements for a best-in-class oral prophylactic therapy. This termination conserves our financial resources and allows us to focus on continuing to advance sebetralstat through the ongoing phase 3 program and towards a planned 2024 NDA filing, as well as on our emerging oral Factor XIIa inhibitor program as a potential once daily prophylactic therapy for people with HAE."

The KOMPLETE trial is a phase 2 clinical trial evaluating KVD824, an investigational oral plasma kallikrein inhibitor designed for the prevention of attacks in adults living with HAE. Patients in the trial were randomized to one of three treatment groups, each placebo controlled: 300 mg, 600 mg, 900 mg KVD824 (or placebo), all dosed twice daily. A total of 33 patients were enrolled in the trial, of which 7 patients experienced either Grade 3 or Grade 4 elevations of liver enzymes at timeframes ranging from two to twelve weeks. The elevations were noted in all treatment groups. One additional Grade 4 elevation was recorded in a patient at the baseline visit, prior to receiving study drug. KalVista will proceed to finalize the database of the trial and assess the unblinded data for efficacy and safety to determine the potential for any further development.

KalVista continues to recruit the phase 3 KONFIDENT trial assessing sebetralstat (formerly KVD900) as a potential oral, on-demand therapy for HAE attacks, with data anticipated in the second half of 2023. Sebetralstat is a distinct compound from KVD824, and no treatment related liver enzyme elevations in patients have been observed in any sebetralstat clinical studies, including in the ongoing Phase 3 KONFIDENT trial.

About KalVista Pharmaceuticals, Inc.

KalVista Pharmaceuticals, Inc. is a pharmaceutical company focused on the discovery, development, and commercialization of oral, small molecule protease inhibitors for diseases with significant unmet need. KalVista has developed a proprietary portfolio of novel, small molecule plasma kallikrein inhibitors initially targeting hereditary angioedema (HAE) and diabetic macular edema (DME). KalVista is developing sebetralstat as an oral on-demand therapy for HAE attacks and is enrolling the Phase 3 KONFIDENT clinical trial. In addition, KalVista's oral Factor XIIa inhibitor program represents a new generation of therapies that may further improve treatment for people living with HAE. In DME, an intravitreally administered plasma kallikrein inhibitor, called KVD001, has completed a Phase 2 clinical trial.

For more information about KalVista, please visit www.kalvista.com.

For more information on the sebetralstat HAE on-demand Phase 3 KONFIDENT trial, please visit www.konfidentstudy.com.

Forward-Looking Statements

This press release contains "forward-looking" statements within the meaning of the safe harbor provisions of the U.S. Private Securities Litigation Reform Act of 1995. Forward-looking statements can be identified by words such as: "anticipate," "intend," "plan," "goal," "seek," "believe," "project," "estimate," "expect," "strategy," "future," "likely," "may," "should," "will" and similar references to future periods. These statements are subject to numerous risks and uncertainties, including the potential impact of COVID-19, that could cause actual results to differ materially from what we expect. Examples of forward-looking statements include, among others, timing or outcomes of communications with the FDA, our expectations about safety and efficacy of our product candidates and timing of clinical trials and its results, our ability to commence clinical studies or complete ongoing clinical studies, including our Phase 3 KONFIDENT trial, and to obtain regulatory approvals for sebetralstat and other candidates in development, the ability of sebetralstat and other candidates in development to treat HAE or DME, and the future progress and potential success of our oral Factor XIIa program. Further information on potential risk factors that could affect our business and financial results are detailed in our filings with the Securities and Exchange Commission, including in our annual report on Form 10-K for the year ended April 30, 2022, our quarterly reports on Form 10-Q, and our other reports that we may make from time to time with the Securities and Exchange Commission. We undertake no obligation to publicly update any forward-looking statement, whether written or oral, that may be made from time to time, whether as a result of new information, future developments or otherwise.

View source version on businesswire.com: https://www.businesswire.com/news/home/20221004005432/en/

KalVista Pharmaceuticals, Inc.
Jarrod Aldom
Vice President, Corporate Communications
(201) 705-0254
jarrod.aldom@kalvista.com

Source: KalVista Pharmaceuticals, Inc.

Copyright © 2023 | All rights reserved. Kalvista Terms | Privacy

Attune Pharmaceuticals

Attune Pharmaceuticals Announces Positive Phase 1 Results for ATN-249, an Oral Plasma Kallikrein Inhibitor for the Treatment of HAE

NEW YORK, Jan. 22, 2019 --(GLOBE NEWSWIRE) -- Attune Pharmaceuticals, a biotechnology company focused on the discovery and development of novel oral small molecule therapeutics for treatment of rare diseases, today announced positive results from the first in human studies evaluating ATN-249, a novel orally administered plasma kallikrein inhibitor for the treatment of Hereditary Angioedema (HAE). The data were presented by Ira Kalfus, M.D., chief medical officer of Attune, as a poster presentation at the Western Society of Allergy, Asthma and Immunology (WSAAI) 2019 Annual Scientific Session.

“We are very pleased that our first-in-human study showed orally administered ATN-249 achieves high blood plasma levels, with dose-dependent pharmacokinetics and a favorable safety profile," said Andrew McDonald, Ph.D., CEO of Attune Pharmaceuticals. “We expect to complete the multiple ascending dose trial shortly and plan to rapidly advance the program to a Phase 2 trial.”

The randomized, placebo-controlled, Phase 1 single ascending dose clinical trial of ATN-249 in healthy volunteers successfully met all its objectives in assessing safety, tolerability and pharmacokinetics (PK) with results supporting further development as an oral plasma kallikrein inhibitor for the prophylactic treatment of HAE. Plasma levels of ATN-249 increased in approximate proportion to dose, and drug exposure was not affected by dosing with food. Once-daily dosing of ATN-249 was safe and well tolerated at all studied doses (50mg to 800 mg) in healthy volunteers. There were no moderate or severe adverse events with all adverse effects being mild and deemed not related to the drug, as well as no dose limiting toxicities.

Ira Kalfus, M.D., said, “The positive outcome of this Phase 1 trial is an important milestone in the development of ATN-249 for the treatment of HAE, a rare and potentially life-threatening disease. Current treatment options for HAE are limited to intravenous and subcutaneous options and are often associated with adverse events. ATN-249, as a potential effective and well-tolerated oral treatment, has the potential to significantly improve disease management and patient quality of life.”

About the Study

The first in human study of ATN-249 was a double blind, placebo-controlled safety, tolerably, and pharmacokinetic single ascending dose study in 48 healthy volunteers. A total of 30 subjects received a single dose of ATN-249 ranging from 50 mg to 800 mg and 12 subjects received placebo. In the food-effect pharmacokinetic study, 6 subjects in the 100 mg cohort received ATN-249 under fasted conditions (period 1) and after a 7-day

washout received a second 100mg dose 30 minutes after the start of a high fat, high caloric meal (period 2).

About Hereditary Angioedema

Hereditary angioedema (HAE) is a rare, potentially life-threatening disease characterized by acute skin and mucosal edema. It is caused by an autosomal dominant mutation of the SERPING1 or F12 genes, resulting in diminished C1 inhibitor levels and/or function. Dysregulation of the contact-kallikrein pathway mediated by dysfunctional C1 inhibitor causes upregulation of bradykinin production, leading to increased vascular permeability, recurrent abdominal pain, and mucosal swelling, which can be fatal with laryngeal involvement. Current treatments are limited by route of administration and adverse events, since all leading HAE drugs are administered intravenously or subcutaneously and may be associated with drug-specific adverse effects.

About ATN-249's Clinical Development Program

ATN-249 is a novel, potent, selective, and orally-administered plasma kallikrein inhibitor for the treatment of Hereditary Angioedema (HAE). Preclinical studies in both biochemical and contact activation assays have demonstrated that ATN-249 is highly selective and potent at plasma kallikrein inhibition. Upon conclusion of the Phase I study in healthy volunteers, a Phase 2, four-week dose ranging trial to evaluate the safety, tolerability, pharmacokinetics, pharmacodynamics, and efficacy of ATN-249 as a preventative treatment to reduce the frequency of attacks in HAE patients is expected to begin.

About Attune Pharmaceuticals

Attune Pharmaceuticals is a clinical-stage biotechnology company focused on the discovery and development of novel once-daily small molecule therapeutics for treatment of rare diseases. Attune Pharmaceuticals is currently advancing two programs, one in Hereditary Angioedema (HAE) and a second in complement-mediated diseases.

Attune Contacts

Corporate Contact:
Attune Pharmaceuticals
(646) 597-6987
info@attunepharma.com

Media Contact:
Allison Blum, Ph.D.
LifeSci Public Relations
(646) 627-8383
Allison@lifescipublicrelations.com

NTLA-2002 in Adults With Hereditary Angioedema (HAE) (NTLA-2002)

The safety and scientific validity of this study is the responsibility of the study sponsor and investigators. Listing a study does not mean it has been evaluated by the U.S. Federal Government. Know the risks and potential benefits of clinical studies and talk to your health care provider before participating. Read our disclaimer for details.

ClinicalTrials.gov Identifier: NCT05120830

Recruitment Status : Recruiting
First Posted : November 15, 2021
Last Update Posted : June 18, 2023

See **Contacts and Locations**

View this study on the modernized ClinicalTrials.gov

Sponsor:
Intellia Therapeutics

Information provided by (Responsible Party):
Intellia Therapeutics

Study Description

Brief Summary:
This study will be conducted to evaluate the safety, tolerability, activity, pharmacokinetics, and pharmacodynamics of **NTLA-2002** in adults with Hereditary Angioedema (HAE).

Condition or disease	Intervention/treatment	Phase
Hereditary Angioedema	Biological: Biological **NTLA-2002**<br>Other: Normal Saline IV Administration	Phase 1<br>Phase 2

Study Design

Study Type : Interventional (Clinical Trial)
Estimated Enrollment : 55 participants
Allocation: Non-Randomized
Intervention Model: Sequential Assignment
Masking: None (Open Label)
Primary Purpose: Treatment
Official Title: Phase 1/2 Study to Evaluate Safety, Tolerability, Pharmacokinetics, and Pharmacodynamics of **NTLA-2002** in Adults With Hereditary Angioedema (HAE)
Actual Study Start Date : December 10, 2021
Estimated Primary Completion Date : April 15, 2024
Estimated Study Completion Date : December 15, 2025

Resource links provided by the National Library of Medicine

MedlinePlus Genetics related topics: Hereditary angioedema

Genetic and Rare Diseases Information Center resources: Hereditary Angioedema

U.S. FDA Resources

Arms and Interventions

Arm	Intervention/treatment
Experimental: Phase 1 Study Arm<br>Participants assigned to 1 of 3 dose-escalation cohorts will receive a single dose of **NTLA-2002** on Day 1 and will then be followed for 104 weeks. Primary observation period is 16 weeks.	Biological: Biological **NTLA-2002**<br>CRISPR/Cas9 gene editing system delivered by LNP for IV administration
Experimental: Phase 2 Experimental Study Arm<br>Participants randomized to **NTLA-2002** (2 dose levels), will receive a single dose of **NTLA-2002** on Day 1 and will then be followed for 104 weeks. Primary observation period is 16 weeks.	Biological: Biological **NTLA-2002**<br>CRISPR/Cas9 gene editing system delivered by LNP for IV administration
Placebo Comparator: Phase 2 Placebo Comparator Study Arm<br>Participants randomized to placebo will receive IV normal saline on Day 1 and will then be followed for up to 104 weeks. Primary observation period is 16 weeks.	Other: Normal Saline IV Administration<br>The administration of IV normal saline

Outcome Measures

Primary Outcome Measures :

1. Safety and tolerability of **NTLA-2002** as determined by adverse events (AEs) and dose limiting toxicities (DLTs) [Time Frame: From NTLA-2002 infusion up to week 104 post-infusion]
   (Phase 1 only)
2. Number of HAE attacks per month (Weeks 1-16) [Time Frame: From study drug infusion up to week 16 post-infusion]
   (Phase 2 only)

Secondary Outcome Measures :

1. Change from baseline in total plasma kallikrein protein level [Time Frame: From NTLA-2002 infusion up to week 104 post-infusion]
   (Phase 1 & 2)
2. Plasma and urine concentrations for DMG-PEG2k, LP000001, Cas9 mRNA, and sgRNA [Time Frame: From NTLA-2002 infusion up to week 104 post-infusion]
   (Phase 1 & 2)
3. Safety and tolerability of **NTLA-2002** as determined by AEs [Time Frame: From study drug infusion up to week 104 post-infusion]
   (Phase 2 only)
4. Number of HAE attacks per month (Weeks 5-16) [Time Frame: From week 6 post-infusion up to week 16 post-infusion]
   (Phase 2 only)
5. Number of HAE attacks per month requiring acute therapy (Weeks 1-16, Weeks 5-16) [Time Frame: From study drug infusion up to week 16 post-infusion]
   (Phase 2 only)

Eligibility Criteria

Information from the National Library of Medicine

Choosing to participate in a study is an important personal decision. Talk with your doctor and family members or friends about deciding to join a study. To learn more about this study, you or your doctor may contact the study research staff using the contacts provided below. For general information, Learn About Clinical Studies.

Ages Eligible for Study: 18 Years and older (Adult, Older Adult)
Sexes Eligible for Study: All
Accepts Healthy Volunteers: No

Criteria

Inclusion Criteria:

1. Age >18 years
2. Diagnosis of HAE Types I or II
3. Ability to provide evidence of HAE attacks to meet the screening requirement
4. Subjects must have access to, and the ability to use, ≥ 1 acute medication(s) to treat angioedema attacks.
5. Adequate chemistry and hematology measures at screening
6. Subjects must agree not to participate in another interventional study for the duration of this trial.
7. Subjects must be capable of providing signed informed consent

Exclusion Criteria:

1. Concurrent diagnosis of any other type of recurrent angioedema
2. Subjects who have known negative reaction or hypersensitivity to any lipid nanoparticles (LNP) component.
3. Any condition that, in the Investigator's opinion, could adversely affect the safety of the subject.
4. Unwilling to comply with study procedures.

Contacts and Locations

Information from the National Library of Medicine

To learn more about this study, you or your doctor may contact the study research staff using the contact information provided by the sponsor.

*Please refer to this study by its ClinicalTrials.gov identifier (NCT number): **NCT05120830***

Contacts

Contact: Trial Manager at Intellia 833-888-0387 clinicalscience@intelliatx.com

Locations

France
Clinical Trial Site **Recruiting**
Grenoble, France
Clinical Trial Site **Recruiting**
Lille, France
Clinical Trial Site **Recruiting**
Paris, France

Germany
Clinical Trial Site **Recruiting**
Berlin, Germany

Netherlands
Clinical Trial Site **Recruiting**
Amsterdam, Netherlands

New Zealand
Clinical Trial Site **Recruiting**
Auckland, New Zealand

United Kingdom
Clinical Trial Site **Recruiting**
Cambridge, United Kingdom

Sponsors and Collaborators

Intellia Therapeutics

More Information

Responsible Party: Intellia Therapeutics
ClinicalTrials.gov Identifier: NCT05120830 History of Changes
Other Study ID Numbers: ITL-2002-CL-001
First Posted: November 15, 2021 Key Record Dates
Last Update Posted: June 18, 2023
Last Verified: June 2023

Individual Participant Data (IPD) Sharing Statement:
Plan to Share IPD: No

Studies a U.S. FDA-regulated Drug Product: No
Studies a U.S. FDA-regulated Device Product: No

Additional relevant MeSH terms:

Angioedema	Hypersensitivity, Immediate
Angioedemas, Hereditary	Hypersensitivity
Vascular Diseases	Immune System Diseases
Cardiovascular Diseases	Hereditary Complement Deficiency Diseases
Urticaria	Primary Immunodeficiency Diseases
Skin Diseases, Vascular	Genetic Diseases, Inborn
Skin Diseases	Immunologic Deficiency Syndromes

Press Release

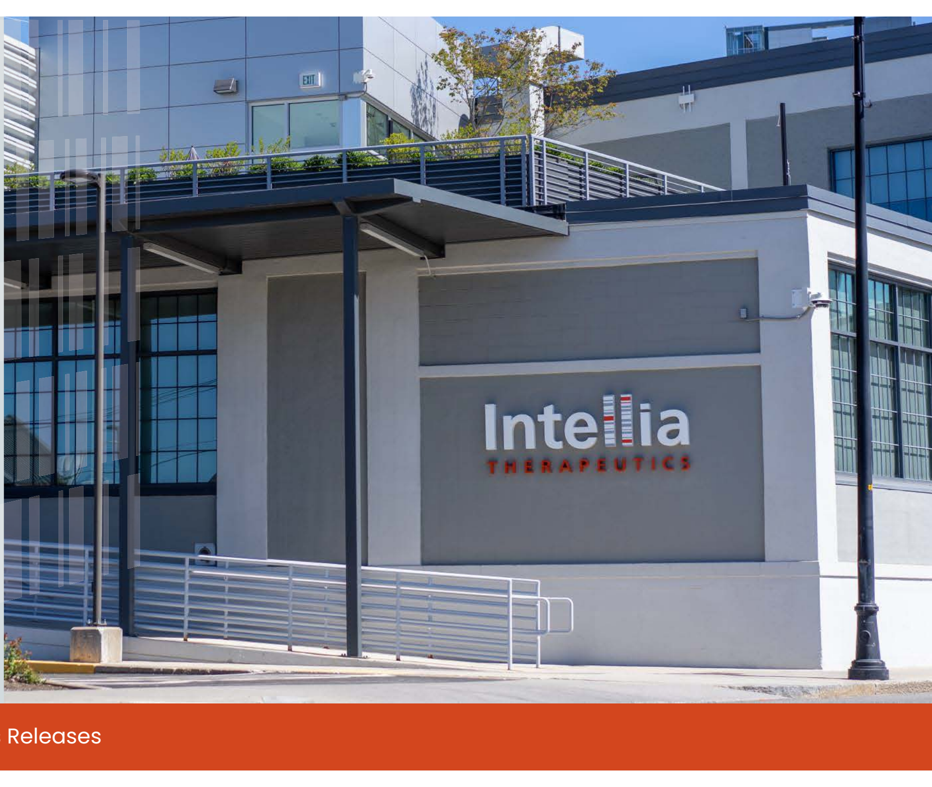

Back to Press Releases

Intellia Therapeutics Announces Positive Interim Clinical Data for its Second Systemically Delivered Investigational CRISPR Candidate, NTLA-2002 for the Treatment of Hereditary Angioedema (HAE)

September 16, 2022 at 7:00 AM EDT

PDF Version

- *Positive interim clinical data further validate the modularity of Intellia's industry-leading genome editing platform and its potential to target a multitude of genetic diseases*
- *A single dose of NTLA-2002 led to a 65% and 92% mean plasma kallikrein reduction at 25 mg and 75 mg doses, respectively, at week eight*
- *HAE attacks were reduced by 91% in the 25 mg dose cohort through week 16; two of three patients remain attack free since treatment with third patient attack free since week 10 through latest follow-up*
- *NTLA-2002 was generally well-tolerated at both dose levels*
- *Intellia plans to initiate the Phase 2 dose-expansion portion of the study in 1H 2023*
- *Intellia to host investor event today, Friday, September 16, at 8:00 a.m. ET*

CAMBRIDGE, Mass., Sept. 16, 2022 (GLOBE NEWSWIRE) -- Intellia Therapeutics, Inc. (NASDAQ:NTLA), a leading clinical-stage genome editing company focused on developing potentially curative therapeutics leveraging CRISPR-based technologies, today announced positive interim results from an ongoing Phase 1/2 clinical study of NTLA-2002, its second *in vivo* genome editing candidate. NTLA-2002 is a systemically administered CRISPR candidate being developed for hereditary angioedema (HAE) and is designed to knock out the *KLKB1* gene in liver cells, thereby reducing the production of kallikrein protein. Uncontrolled activity of kallikrein is responsible for the overproduction of bradykinin, which leads to the recurring, debilitating and potentially fatal swelling attacks that occur in people living with HAE. The interim data were shared today in an oral presentation at the 2022 Bradykinin Symposium held in Berlin, Germany.

The data presented are from the initial six adult patients with HAE in the ongoing dose-escalation study with a data cut-off date of July 27, 2022. Single doses of 25 mg (n=3) and 75 mg (n=3) of NTLA-2002 were administered via intravenous infusion, and changes from baseline values of plasma kallikrein protein were measured for each patient. Administration of NTLA-2002 led to dose-dependent reductions in plasma kallikrein and achieved maximal reductions by week eight, with mean reductions of 65% and 92% in the 25 mg and 75 mg dose cohorts, respectively. Furthermore, these reductions were sustained through at least 16 weeks in the 25 mg cohort and eight weeks in the 75 mg cohort for which complete cohort biomarker data were available.

In addition to plasma kallikrein levels, HAE attack rates are also being measured in the study, with the first analysis occurring at the end of the pre-specified 16-week primary observation period. To date, all three patients in the 25 mg dose cohort have reached the end of this initial observation period. Patients in this group had a baseline HAE attack rate ranging from 1.1 to 7.2 attacks per month, as confirmed by the investigator. Treatment with a single dose of 25 mg of NTLA-2002 resulted in a mean reduction in HAE attacks of 91% throughout the 16-week observation period. Additionally, two of the three patients have not had a single HAE attack since treatment, and all three patients have been attack free since week 10 (follow-up through weeks 24 - 32). Patients in the 75 mg cohort have not completed the primary 16-week observation period. Attack-rate data for this cohort will be presented at the American College of Allergy, Asthma & Immunology (ACAAI) Annual Scientific Meeting, November 10 – 14 in Louisville, Kentucky.

Prophylaxis medications are permitted in the Phase 1 part of the study. Two of the three patients in the 25 mg cohort were actively receiving prophylaxis therapy prior to administration of NTLA-2002. For these two patients, the study protocol permitted investigators to withdraw the patient's prophylaxis therapy after completion of the 16-week primary observation period. This treatment approach was implemented for the two applicable patients in this cohort, and neither patient has had an HAE attack since discontinuing their prophylaxis therapy through the latest follow-up.

"These initial data represent a significant milestone for both Intellia and people around the world suffering from genetic diseases, such as HAE," said Intellia President and Chief Executive Officer John Leonard, M.D. "We are strongly encouraged by the greater than 90% reduction in HAE attacks observed in the 25 mg dose cohort, as these interim results support our belief that a single dose of NTLA-2002 has the potential to permanently prevent the debilitating swelling attacks associated with HAE. Additionally, today's announcement continues to validate our genome editing approach and the modular platform we have built. This is now the second time in history clinical data have been generated suggesting we can precisely edit target cells within the human body to potentially treat genetic diseases with a single, systemic administration of a CRISPR-based therapy. We plan to move as quickly and judiciously as possible on behalf of people living with HAE and a number of additional genetic diseases in the months and years ahead."

At both dose levels, NTLA-2002 was generally well-tolerated, and the majority of adverse events were mild in severity. The most frequent adverse events were infusion-related reactions, which were mostly Grade 1 and resolved within one day. There have been no dose-limiting toxicities, no serious adverse events and no adverse events of Grade 3 or higher observed to date. No clinically significant laboratory abnormalities were observed, including any significant elevation in liver enzymes.

"Many people living with HAE continue to experience breakthrough attacks despite currently available treatments and often find the burden of untreated attacks, frequent infusions or injections to be tremendously disruptive to their lives," said Hilary Longhurst, M.D., Ph.D., Faculty of Medical and Health Sciences, University of Auckland, New Zealand, and the trial's principal investigator in New Zealand. "These early data support NTLA-2002 as a potential one-time treatment capable of producing profound reductions in HAE attacks. While the clinical data are still emerging, I am highly optimistic that NTLA-2002 could become a new treatment option for the HAE community."

Based on the interim data presented today, Intellia selected a third dose of 50 mg to be evaluated in the ongoing dose-escalation portion of the Phase 1/2 study. Dosing at this level has recently completed, and Intellia expects to select up to two doses to further evaluate in the Phase 2, placebo-controlled, dose-expansion portion of the study, which is expected to begin in the first half of 2023. Intellia anticipates expanding country and site participation, including U.S. clinical sites, as part of the Phase 2 study.

Intellia Therapeutics Investor Event and Webcast Information
Intellia will host a live webcast today, Friday, September 16, 2022, at 8:00 a.m. ET, to provide a clinical update from its *in vivo* portfolio, during which the company will review the presented clinical data at the 2022 Bradykinin Symposium alongside interim results from NTLA-2001. To join the webcast, please visit this link, or the Events and Presentations page of the Investors & Media section of the company's website at www.intelliatx.com. A replay of the webcast will be available on Intellia's website for at least 30 days following the call.

About the NTLA-2002 Clinical Program
Intellia's multi-national Phase 1/2 study is evaluating the safety, tolerability, pharmacokinetics and pharmacodynamics of NTLA-2002 in adults with Type I or Type II hereditary angioedema (HAE). This includes the measurement of plasma kallikrein protein levels and activity as determined by HAE attack rate measures. The Phase 1 portion of the study is an open-label, single-ascending dose design used to identify up to two dose levels of NTLA-2002 that will be further evaluated in the randomized, placebo-controlled Phase 2 portion of the study. This Phase 1/2 study will identify the dose of NTLA-2002 for use in future studies. Visit clinicaltrials.gov (NCT05120830) for more details.

About NTLA-2002

Based on Nobel Prize-winning CRISPR/Cas9 technology, NTLA-2002 is the first single-dose investigational treatment being explored in clinical trials for the potential to continuously reduce kallikrein activity and prevent attacks in people living with hereditary angioedema (HAE). NTLA-2002 is a wholly owned investigational CRISPR therapeutic candidate designed to inactivate the *kallikrein B1 (KLKB1*) gene, which encodes for prekallikrein, the kallikrein precursor protein. NTLA-2002 is Intellia's second investigational CRISPR therapeutic candidate to be administered systemically, by intravenous infusion, to edit disease-causing genes inside the human body with a single dose of treatment. Intellia's proprietary non-viral platform deploys lipid nanoparticles to deliver to the liver a two-part genome editing system: guide RNA specific to the disease-causing gene and messenger RNA that encodes the Cas9 enzyme, which together carry out the precision editing.

About Hereditary Angioedema

Hereditary angioedema (HAE) is a rare, genetic disorder characterized by severe, recurring and unpredictable inflammatory attacks in various organs and tissues of the body, which can be painful, debilitating and life-threatening. It is estimated that one in 50,000 people are affected by HAE, and current treatment options often include life-long therapies, which may require chronic intravenous (IV) or subcutaneous (SC) administration as often as twice per week, or daily oral administration to ensure constant pathway suppression for disease control. Despite chronic administration, breakthrough attacks still occur. Kallikrein inhibition is a clinically validated strategy for the preventive treatment of HAE attacks.

About Intellia Therapeutics
Intellia Therapeutics, a leading clinical-stage genome editing company, is developing novel, potentially curative therapeutics leveraging CRISPR-based technologies. To fully realize the transformative potential of CRISPR-based technologies, Intellia is pursuing two primary approaches. The company's *in vivo* programs use intravenously administered CRISPR as the therapy, in which proprietary delivery technology enables highly precise editing of disease-causing genes directly within specific target tissues. Intellia's *ex vivo* programs use CRISPR to create the therapy by using engineered human cells to treat cancer and autoimmune diseases. Intellia's deep scientific, technical and clinical development experience, along with its robust intellectual property portfolio, have enabled the company to take a leadership role in harnessing the full potential of genome editing to create new classes of genetic medicine. Learn more at intelliatx.com. Follow us on Twitter @intelliatx.

Forward-Looking Statements

This press release contains "forward-looking statements" of Intellia Therapeutics, Inc. ("Intellia" or the "Company") within the meaning of the Private Securities Litigation Reform Act of 1995. These forward-looking statements include, but are not limited to, express or implied statements regarding Intellia's beliefs and expectations regarding: its ability to conduct and complete clinical studies for NTLA-2002 for the treatment of hereditary angioedema (HAE); its ability to generate data to demonstrate NTLA-2002 as a potential single-dose treatment for HAE, including safety, kallikrein reduction and attack rate data including permanently preventing debilitating swelling attacks; its ability to develop its modular platform and full-spectrum approach to advance its complex genome editing capabilities, including to apply its proprietary cell engineering platform to additional product candidates; the advancement and expansion of its CRISPR/Cas9 technology to develop human therapeutic products; its ability to maintain and expand its related intellectual property portfolio, and avoid or acquire rights to valid intellectual property of third parties; its ability to demonstrate its platform's modularity and replicate or apply results achieved in preclinical studies, including those in its NTLA-2002 program, in any future studies, including human clinical trials; its ability to develop other in vivo or ex vivo cell therapeutics of all types, and NTLA-2002 in particular, using CRISPR/Cas9 technology; and the timing of regulatory filings and clinical trial execution, including enrollment and dosing of patients.

Any forward-looking statements in this press release are based on management's current expectations and beliefs of future events, and are subject to a number of risks and uncertainties that could cause actual results to differ materially and adversely from those set forth in or implied by such forward-looking statements. These risks and uncertainties include, but are not limited to: risks related to the successful enrollment of patients in the Phase 1/2 study for NTLA-2002 for the treatment of HAE; risks related to Intellia's ability to protect and maintain its intellectual property position; risks related to the authorization, initiation and conduct of studies and other development requirements, including manufacturing, for its in vivo and ex vivo product candidates, including NTLA-2002; the risk that any one or more of Intellia's product candidates, including NTLA-2002, will not be successfully developed and commercialized; the risk that the results of preclinical studies or clinical studies, including for NTLA-2002, will not be predictive of future results in connection with future studies; and the risk that Intellia's will not be able to demonstrate its platform's modularity and replicate or apply results achieved in preclinical studies to develop additional product candidates, including to apply its proprietary CRISPR/Cas9 technology platform successfully to additional product candidates. For a discussion of these and other risks and uncertainties, and other important factors, any of which could cause Intellia's actual results to differ from those contained in the forward-looking statements, see the section entitled "Risk Factors" in Intellia's most recent annual report on Form 10-K and quarterly report on Form 10-Q, as well as discussions of potential risks, uncertainties and other important factors in Intellia's other filings with the Securities and Exchange Commission (SEC). All information in this press release is as of the date of the release, and Intellia undertakes no duty to update this information unless required by law.

Intellia Contacts:

Investors:
Ian Karp
Senior Vice President, Investor Relations and Corporate Communications
+1-857-449-4175
ian.karp@intelliatx.com

Lina Li
Senior Director, Investor Relations and Corporate Communications
+1-857-706-1612
lina.li@intelliatx.com

Media:
Rebecca Spalding
Ten Bridge Communications
+1-646-509-3831
media@intelliatx.com
rebecca@tenbridgecommunications.com

Intellia THERAPEUTICS

Source: Intellia Therapeutics, Inc.

SHARE | TWEET | SHARE | COPY

Programs & Pipeline

We believe our modular approach to selecting our in vivo and ex vivo programs positions us to build a full-spectrum genome editing company, with a pipeline across a range of indications.

See our progress

Contacts | E-mail Alerts | RSS Feeds | E-mail Page | Print Page

© Intellia Therapeutics, Inc. 2023
Intellia Therapeutics and the Intellia Therapeutics logo are registered trademarks with the U.S. Patent & Trademark Office.

This is the classic website, which will be retired eventually. Please visit the modernized ClinicalTrials.gov instead.

U.S. National Library of Medicine
ClinicalTrials.gov

Find Studies ▾ About Studies ▾ Submit Studies ▾ Resources ▾ About Site ▾ PRS Login

Efficacy and Safety of IONIS-PKKRx for Preventive Treatment of Chronic Migraine

The safety and scientific validity of this study is the responsibility of the study sponsor and investigators. Listing a study does not mean it has been evaluated by the U.S. Federal Government. Read our disclaimer for details.

ClinicalTrials.gov Identifier: NCT03108469

Recruitment Status : Completed
First Posted : April 11, 2017
Results First Posted : February 5, 2020
Last Update Posted : February 12, 2020

View this study on the modernized ClinicalTrials.gov

Sponsor:
Smith, Timothy R., M.D.

Collaborators:
Clinvest Research, LLC
Ionis Pharmaceuticals, Inc.

Information provided by (Responsible Party):
Timothy Smith, Smith, Timothy R., M.D.

Study Description

Go to ▾

Brief Summary:
The purpose of this study is to evaluate the safety, tolerability, and changes in the number of migraine and headache days with repeated subcutaneous administration of **IONIS-PKKRx** (ISIS 546254) or placebo in subjects with chronic migraine.

Condition or disease	Intervention/treatment	Phase
Chronic Migraine	Drug: **IONIS-PKKRx** (ISIS 546254)<br>Other: Placebo	Phase 2

Detailed Description:

This is a double-blind, placebo-controlled, randomized, multi-center study in subjects with chronic migraine. The study will consist of 7 office visits, 6 Sample collection visits and 3 phone call assessments. Subjects agreeing to participate in the study and meeting the entry criteria assessed at the screening visit, will begin a 28 day baseline period to confirm their diagnosis, and establish a baseline frequency of migraine and headache days. During the baseline period, subjects will continue treating their migraines in their usual manner. They will monitor headache activity, migraine related symptoms, and medication usage with an electronic daily headache diary.

Subjects who, after completing the baseline, continue to meet entrance criteria will be eligible to enter into the 4 month treatment phase. They will be randomized according to the Clinvest generated randomization schedule. A total of 30 randomized subjects will enter the treatment phase receiving IONIS-PKKRx (ISIS 546254; SC) or placebo in a 1:1 design. Study drug or placebo will be administered weekly for 16 weeks. A short phone call to assess any treatment related adverse events will take place 1 and 2 days after randomization. Daily electronic diary assessments will collect headache frequency and severity, associated migraine symptoms, acute medication usage, and the emergence of unusual symptoms and adverse events. Subjects will return to the site at weeks 4, 8, and 12 for investigational product (IP) accountability/dispensing, medication and medication updates, biomarker/lab sample collection, and assessment of adverse events. An end of treatment visit will take place 16 weeks after randomization.

Subjects will have a follow-up safety visit one month after their last dosage of IP) for assessment of any adverse events (AE) and satisfaction and a final safety phone call 2 months following their last office visit (3 months after last dose of IP) for assessment of any adverse events (AE). Subjects will continue to complete headache diaries through Visit 7. Subjects will also have hematology samples collected every other week starting after Visit 2 through Day 154.

Safety and tolerability will be monitored by the Investigators. Patients who discontinue study treatment prematurely should complete any follow-up visits associated with the most recent dose and should move into and complete the Follow-up Phase.

Subjects will undergo sampling for (pharmacokinetics) PK, coagulation, chemistry, hematology, and optional future biomedical research, as specified in the schedule of procedures.

Study Design

Go to ▾

Study Type :	Interventional (Clinical Trial)
Actual Enrollment :	30 participants
Allocation:	Randomized
Intervention Model:	Parallel Assignment
Masking:	Double (Participant, Investigator)
Masking Description:	At randomization (Visit 2), neither the subject nor the investigator will be aware to which treatment group the subject has been assigned. Investigational product will be drawn and administered in a double blind format. If needed, for safety and proper treatment of the subject, the investigator can unblind the subject's treatment assignment to determine which treatment has been assigned and institute appropriate follow-up care. When possible, Sponsor and Clinvest should be notified prior to unblinding study drug.
Primary Purpose:	Treatment
Official Title:	A Multicenter, Randomized, Double-Blind, Placebo-Controlled Study Evaluating the Efficacy and Safety of ISIS 546254 for Preventive Treatment of Chronic Migraine
Actual Study Start Date :	August 1, 2017
Actual Primary Completion Date :	April 26, 2019
Actual Study Completion Date :	April 26, 2019

Resource links provided by the National Library of Medicine NIH NLM

MedlinePlus Genetics related topics: Migraine

MedlinePlus related topics: Migraine

Genetic and Rare Diseases Information Center resources: Chronic Graft Versus Host Disease

U.S. FDA Resources

Arms and Interventions

Go to ▾

Arm	Intervention/treatment
Active Comparator: **IONIS-PKKRx** (ISIS 546254)<br>Those randomized to **IONIS-PKKRx** (ISIS 546254) will receive subcutaneous injections containing 1.00 mL (200mg) weekly for weeks 1-16.	Drug: **IONIS-PKKRx** (ISIS 546254)<br>Those randomized to **IONIS-PKKRx** (ISIS 546254) will receive subcutaneous injections containing 1.00 mL (200mg) of **IONIS- PKKRx** (ISIS 546254) weekly for weeks 1-16.
Placebo Comparator: Placebo<br>Those randomized to placebo will receive subcutaneous injections containing 1.00 mL (200mg) weekly for weeks 1-16.	Other: Placebo<br>Those randomized to placebo will receive subcutaneous injections 1.00 mL (200mg) weekly for weeks 1-16.

Outcome Measures

Go to ▾

Primary Outcome Measures :

1. Migraine Days [Time Frame: Comparing baseline to treatment period, up to 16 weeks]

   Compare the efficacy of ISIS 546254 in the preventive treatment of chronic migraine, measured by the number of monthly migraine days comparing baseline to the final month of the 4-month treatment period for subjects treated with ISIS 546254 vs. placebo.

Secondary Outcome Measures :

1. Headache Severity [Time Frame: Comparing baseline to treatment period, up to 16 weeks]

   Evaluate the efficacy of ISIS 546254 in the preventive treatment of chronic migraine, measured by the monthly headache severity comparing baseline to the final month of the 4-month treatment period for subjects treated with ISIS 546254 vs. placebo. Headache severity scale ranges from 1 (Mild) to 3 (Severe).

2. Headache Days [Time Frame: Comparing baseline to treatment period, up to 16 weeks]

   Compare the efficacy of ISIS 546254 in the preventive treatment of chronic migraine, measured by the number of monthly headache days comparing baseline to the final month of the 4-month treatment period for subjects treated with ISIS 546254 vs. placebo.

3. Number of Subjects Reporting a ≥ 50% Reduction in the Number of Migraine Headaches [Time Frame: Comparing baseline to treatment period, up to 16 weeks]

   Compare the number of patients meeting 50% response criteria, response defined as a ≥ 50% reduction in the number of headaches from baseline to the final month of the 4-month treatment period for subjects treated with ISIS 546254 vs. placebo.

4. Count of Migraine Headache Days Requiring Use of Migraine Medication [Time Frame: Comparing baseline to treatment period, up to 16 weeks]

   Compare the count of the number of headache days requiring use of medication for the treatment of migraine or headache pain (i.e., acute and rescue or breakthrough medication use) from baseline to the final month of the 4-month treatment period for subjects treated with ISIS 546254 vs. placebo.

5. Migraine Specific Quality of Life (MSQ) Questionnaire Score [Time Frame: Comparing baseline to treatment period, up to 16 weeks]

   Compare scores from baseline to the final month of the 4-month treatment period in the Migraine Specific Quality of Life (MSQ) Questionnaire for subjects treated with ISIS 546254 vs. placebo.

   The MSQ total score is derived from the summation of fourteen questions on the MSQ. Answer options for the MSQ are coded as: 1 = None of the time, 2 = A little bit of the time, 3 = Some of the time, 4 = A good bit of the time, 5 = Most of the time, 6 = All of the time. The range of total MSQ score for each time point is 14 to 84, with higher scores indicating more effects of migraine on the subject's daily activities.

6. Physician Global Impress of Change (PGIC) Score [Time Frame: Evaluating the treatment period, but collected at the end of treatment, up to 16 weeks]

   Compare the end of treatment month 4 in the physician global impress of change (PGIC) for subjects treated with ISIS 546254 vs. placebo.

   The PGIC is a one item questionnaire asking, "Since the beginning of treatment in this study, how would you describe this patient's change (if any) in their overall status?". The answer option for the PGIC is coded as: 3 = Very Much Improved, 2 = Much Improved, 1 = Minimally Improved, 0 = No Change, -1 = Minimally Worse, -2 = Much Worse, -3 = Very Much Worse, with negative scores indicating the subject's overall status has decreased while in the study and positive scores indicating the subject's overall status has increased while in the study.

7. Subject Global Impress of Change (SGIC) Score [Time Frame: Evaluating the treatment period, but collected at the end of treatment, up to 16 weeks]

   Compare the end of treatment month 4 in the subjects' global impression of change (SGIC) for subjects treated with ISIS 546254 vs. placebo.

   The SGIC is a one item questionnaire asking, "Since the beginning of treatment in this study, how would you describe the change (if any) in your overall status?". The answer option for the SGIC is coded as: 3 = Very Much Improved, 2 = Much Improved, 1 = Minimally Improved, 0 = No Change, -1 = Minimally Worse, -2 = Much Worse, -3 = Very Much Worse, with negative scores indicating the subject's overall status has decreased while in the study and positive scores indicating the subject's overall status has increased while in the study.

Eligibility Criteria

Go to ▾

Information from the National Library of Medicine NIH NLM

Choosing to participate in a study is an important personal decision. Talk with your doctor and family members or friends about deciding to join a study. To learn more about this study, you or your doctor may contact the study research staff using the contacts provided below. For general information, Learn About Clinical Studies.

Ages Eligible for Study:	18 Years to 65 Years (Adult, Older Adult)
Sexes Eligible for Study:	All
Accepts Healthy Volunteers:	No

Criteria

Inclusion Criteria:

Potential subjects must meet the following criteria at the screening visit to enter this study:

1. male or female, in otherwise good health, 18 to 65 years of age.
2. history of chronic migraine meeting the diagnostic criteria listed in the International Classification of Headache Disorders (ICHD-III beta version, 2013), as follows:

   a. History of frequent headaches suggestive of chronic migraine (15 or greater days of qualifying headaches per month) for at least three months prior to screening b. Verification of headache frequency through prospectively collected baseline information during the 28-day run-in phase demonstrating headaches on at least 15 days, with at least 8 days per month fulfilling any ONE of the following; i. Qualify as being a migraine attack ii. Relieved by migraine specific acute medications
3. onset of migraine before age 50.
4. stable pattern of migraine pattern for at least 3 months prior to screening.
5. not currently taking a migraine preventive OR has been taking a stable dose of a preventive for at least 30 days prior to screening and agrees to not start, stop, or change medication and/or dosage during the study period.

   i. Subjects on migraine preventative should have stable headache pattern ii. Injections of onabotulinumtoxinA are allowable if subject has completed at least 2 injection cycles and agrees to maintain a regular injection cycle for the duration of the study

Exclusion Criteria:

Potential subjects meeting any of the following criteria will be excluded from entering this study:

1. unable to understand the study requirements, the informed consent, or complete headache records as required per protocol.
2. pregnant, actively trying to become pregnant, or breast-feeding.
3. history of medication overuse (MO) of opioids, or butalbital, as defined by ICHD-3 beta criteria and/or MO during baseline period.
4. history of substance abuse and/or dependence, in the opinion of the Investigator.
5. unstable neurological condition or a significantly abnormal neurological examination with focal signs or signs of increased intracranial pressure.

Contacts and Locations

Go to ▾

Information from the National Library of Medicine NIH NLM

To learn more about this study, you or your doctor may contact the study research staff using the contact information provided by the sponsor.

Please refer to this study by its ClinicalTrials.gov identifier (NCT number): ***NCT03108469***

Locations

United States, Missouri
StudyMetrix
Saint Peters, Missouri, United States, 63303
Clinvest Research, LLC
Springfield, Missouri, United States, 65807

Sponsors and Collaborators
Smith, Timothy R., M.D.
Clinvest Research, LLC
Ionis Pharmaceuticals, Inc.

Investigators
Principal Investigator: Timothy R Smith, MD Clinvest Research, LLC

Study Documents (Full-Text)

Documents provided by Timothy Smith, Smith, Timothy R., M.D.:

Study Protocol [PDF] June 12, 2017

Statistical Analysis Plan [PDF] January 17, 2018

More Information

Go to ▾

Responsible Party:	Timothy Smith, Principal Investigator, Smith, Timothy R., M.D.
ClinicalTrials.gov Identifier:	NCT03108469 History of Changes
Other Study ID Numbers:	15-001IS
First Posted:	April 11, 2017 Key Record Dates
Results First Posted:	February 5, 2020
Last Update Posted:	February 12, 2020
Last Verified:	February 2020

Studies a U.S. FDA-regulated Drug Product: Yes
Studies a U.S. FDA-regulated Device Product: No

Keywords provided by Timothy Smith, Smith, Timothy R., M.D.:
migraine

Additional relevant MeSH terms:

Migraine Disorders	Brain Diseases
Headache Disorders, Primary	Central Nervous System Diseases
Headache Disorders	Nervous System Diseases

Copyright | Privacy | Accessibility | Viewers and Players | Freedom of Information Act | USA.gov | HHS Vulnerability Disclosure
U.S. National Library of Medicine | U.S. National Institutes of Health | U.S. Department of Health and Human Services

This is the classic website, which will be retired eventually. Please visit the modernized ClinicalTrials.gov instead.

Efficacy and Safety of IONIS-PKKRx for Preventive Treatment of Chronic Migraine

The safety and scientific validity of this study is the responsibility of the study sponsor and investigators. Listing a study does not mean it has been evaluated by the U.S. Federal Government. Read our disclaimer for details.

ClinicalTrials.gov Identifier: NCT03108469

Recruitment Status : Completed
First Posted : April 11, 2017
Results First Posted : February 5, 2020
Last Update Posted : February 12, 2020

View this study on the modernized ClinicalTrials.gov

Sponsor:
Smith, Timothy R., M.D.

Collaborators:
Clinvest Research, LLC
Ionis Pharmaceuticals, Inc.

Information provided by (Responsible Party):
Timothy Smith, Smith, Timothy R., M.D.

Study Details | Tabular View | Study Results | Disclaimer | How to Read a Study Record

Study Type	Interventional
Study Design	Allocation: Randomized; Intervention Model: Parallel Assignment; Masking: Double (Participant, Investigator); Primary Purpose: Treatment
Condition	Chronic Migraine
Interventions	Drug: IONIS-PKKRx (ISIS 546254)<br>Other: Placebo
Enrollment	30

Participant Flow

Recruitment Details	
Pre-assignment Details	

Arm/Group Title	IONIS-PKKRx (ISIS 546254)	Placebo
Arm/Group Description	Those randomized to IONIS-PKKRx (ISIS 546254) received subcutaneous injections containing 1.00 mL (200mg/mL) weekly for weeks 1-16.	Those randomized to placebo will receive subcutaneous injections containing 1.00 mL weekly for weeks 1-16.
Period Title: **Overall Study**		
Started	15	15
Completed	11	11
Not Completed	4	4

Baseline Characteristics

Arm/Group Title		IONIS-PKKRx (ISIS 546254)	Placebo	Total
Arm/Group Description		Those randomized to IONIS-PKKRx (ISIS 546254) received subcutaneous injections containing 1.00 mL (200mg/mL) weekly for weeks 1-16.	Those randomized to placebo will receive subcutaneous injections containing 1.00 mL weekly for weeks 1-16.	Total of all reporting groups
Overall Number of Baseline Participants		15	15	30
Baseline Analysis Population Description		[Not Specified]		
Age, Continuous<br>Mean (Standard Deviation)<br>Unit of measure: Years				
	Number Analyzed	15 participants	15 participants	30 participants
		35.6 (13.04)	38.4 (10.83)	37.16 (12.08)
Sex: Female, Male<br>Measure Type: Count of Participants<br>Unit of measure: Participants				
	Number Analyzed	15 participants	15 participants	30 participants
	Female	13 86.7%	12 80.0%	25 83.3%
	Male	2 13.3%	3 20.0%	5 16.7%
Ethnicity (NIH/OMB)<br>Measure Type: Count of Participants<br>Unit of measure: Participants				
	Number Analyzed	15 participants	15 participants	30 participants
	Hispanic or Latino	1 6.7%	0 0.0%	1 3.3%
	Not Hispanic or Latino	14 93.3%	15 100.0%	29 96.7%
	Unknown or Not Reported	0 0.0%	0 0.0%	0 0.0%
Race (NIH/OMB)<br>Measure Type: Count of Participants<br>Unit of measure: Participants				
	Number Analyzed	15 participants	15 participants	30 participants
	American Indian or Alaska Native	0 0.0%	1 6.7%	1 3.3%
	Asian	0 0.0%	0 0.0%	0 0.0%
	Native Hawaiian or Other Pacific Islander	0 0.0%	0 0.0%	0 0.0%
	Black or African American	0 0.0%	0 0.0%	0 0.0%
	White	15 100.0%	14 93.3%	29 96.7%
	More than one race	0 0.0%	0 0.0%	0 0.0%
	Unknown or Not Reported	0 0.0%	0 0.0%	0 0.0%
Region of Enrollment<br>Measure Type: Number<br>Unit of measure: Participants				
United States	Number Analyzed	15 participants	15 participants	30 participants
		15	15	30

Outcome Measures

1. Primary Outcome

Title	Migraine Days
Description	Compare the efficacy of ISIS 546254 in the preventive treatment of chronic migraine, measured by the number of monthly migraine days comparing baseline to the final month of the 4-month treatment period for subjects treated with ISIS 546254 vs. placebo.
Time Frame	Comparing baseline to treatment period, up to 16 weeks

Outcome Measure Data

Analysis Population Description
[Not Specified]

Arm/Group Title	IONIS-PKKRx (ISIS 546254)	Placebo
Arm/Group Description:	Those randomized to IONIS-PKKRx (ISIS 546254) received subcutaneous injections containing 1.00 mL (200mg/mL) weekly for weeks 1-16.	Those randomized to placebo will receive subcutaneous injections containing 1.00 mL weekly for weeks 1-16.
Overall Number of Participants Analyzed	15	15
Mean (Standard Error)<br>Unit of Measure: Days		
Baseline	14.267 (1.48)	14.133 (1.17)
Treatment Month	11.067 (2.08)	9.867 (1.80)

2. Secondary Outcome

Title	Headache Severity
Description	Evaluate the efficacy of ISIS 546254 in the preventive treatment of chronic migraine, measured by the monthly headache severity comparing baseline to the final month of the 4-month treatment period for subjects treated with ISIS 546254 vs. placebo. Headache severity scale ranges from 1 (Mild) to 3 (Severe).
Time Frame	Comparing baseline to treatment period, up to 16 weeks

Outcome Measure Data

Analysis Population Description
[Not Specified]

Arm/Group Title	IONIS-PKKRx (ISIS 546254)	Placebo
Arm/Group Description:	Those randomized to IONIS-PKKRx (ISIS 546254) received subcutaneous injections containing 1.00 mL (200mg/mL) weekly for weeks 1-16.	Those randomized to placebo will receive subcutaneous injections containing 1.00 mL weekly for weeks 1-16.
Overall Number of Participants Analyzed	15	15
Mean (Standard Error)<br>Unit of Measure: units on a scale		
Baseline	1.605 (0.106)	1.538 (0.084)
Treatment Month	1.202 (0.146)	0.994 (0.170)

3. Secondary Outcome

Title	Headache Days
Description	Compare the efficacy of ISIS 546254 in the preventive treatment of chronic migraine, measured by the number of monthly headache days comparing baseline to the final month of the 4-month treatment period for subjects treated with ISIS 546254 vs. placebo.
Time Frame	Comparing baseline to treatment period, up to 16 weeks

Outcome Measure Data

Analysis Population Description
[Not Specified]

Arm/Group Title	IONIS-PKKRx (ISIS 546254)	Placebo
Arm/Group Description:	Those randomized to IONIS-PKKRx (ISIS 546254) received subcutaneous injections containing 1.00 mL (200mg/mL) weekly for weeks 1-16.	Those randomized to placebo will receive subcutaneous injections containing 1.00 mL weekly for weeks 1-16.
Overall Number of Participants Analyzed	15	15
Mean (Standard Error)<br>Unit of Measure: Days		
Baseline	22.267 (1.27)	23.733 (0.86)
Treatment Month	17.733 (2.11)	14.333 (1.76)

4. Secondary Outcome

Title	Number of Subjects Reporting a ≥ 50% Reduction in the Number of Migraine Headaches
Description	Compare the number of patients meeting 50% response criteria, response defined as a ≥ 50% reduction in the number of headaches from baseline to the final month of the 4-month treatment period for subjects treated with ISIS 546254 vs. placebo.
Time Frame	Comparing baseline to treatment period, up to 16 weeks

Outcome Measure Data

Analysis Population Description
[Not Specified]

Arm/Group Title	IONIS-PKKRx (ISIS 546254)	Placebo
Arm/Group Description:	Those randomized to IONIS-PKKRx (ISIS 546254) received subcutaneous injections containing 1.00 mL (200mg/mL) weekly for weeks 1-16.	Those randomized to placebo will receive subcutaneous injections containing 1.00 mL weekly for weeks 1-16.
Overall Number of Participants Analyzed	15	15
Measure Type: Count of Participants<br>Unit of Measure: Participants		
	2 13.3%	6 40.0%

5. Secondary Outcome

Title	Count of Migraine Headache Days Requiring Use of Migraine Medication
Description	Compare the count of the number of headache days requiring use of medication for the treatment of migraine or headache pain (i.e., acute and rescue or breakthrough medication use) from baseline to the final month of the 4-month treatment period for subjects treated with ISIS 546254 vs. placebo.
Time Frame	Comparing baseline to treatment period, up to 16 weeks

Outcome Measure Data

Analysis Population Description
[Not Specified]

Arm/Group Title	IONIS-PKKRx (ISIS 546254)	Placebo
Arm/Group Description:	Those randomized to IONIS-PKKRx (ISIS 546254) received subcutaneous injections containing 1.00 mL (200mg/mL) weekly for weeks 1-16.	Those randomized to placebo will receive subcutaneous injections containing 1.00 mL weekly for weeks 1-16.
Overall Number of Participants Analyzed	15	15
Mean (Standard Error)<br>Unit of Measure: Days		
Baseline	7.2 (1.64)	14.733 (2.09)
Treatment Month	4.733 (1.64)	7.733 (2.00)

6. Secondary Outcome

Title	Migraine Specific Quality of Life (MSQ) Questionnaire Score
Description	Compare scores from baseline to the final month of the 4-month treatment period in the Migraine Specific Quality of Life (MSQ) Questionnaire for subjects treated with ISIS 546254 vs. placebo.<br>The MSQ total score is derived from the summation of fourteen questions on the MSQ. Answer options for the MSQ are coded as: 1 = None of the time, 2 = A little bit of the time, 3 = Some of the time, 4 = A good bit of the time, 5 = Most of the time, 6 = All of the time. The range of total MSQ score for each time point is 14 to 84, with higher scores indicating more effects of migraine on the subject's daily activities.
Time Frame	Comparing baseline to treatment period, up to 16 weeks

Outcome Measure Data

Analysis Population Description
[Not Specified]

Arm/Group Title	IONIS-PKKRx (ISIS 546254)	Placebo
Arm/Group Description:	Those randomized to IONIS-PKKRx (ISIS 546254) received subcutaneous injections containing 1.00 mL (200mg/mL) weekly for weeks 1-16.	Those randomized to placebo will receive subcutaneous injections containing 1.00 mL weekly for weeks 1-16.
Overall Number of Participants Analyzed	15	15
Mean (Standard Error)<br>Unit of Measure: score on a scale		
Baseline	50.133 (2.40)	50.133 (2.63)
Treatment Month	41.133 (3.46)	40.733 (3.16)

7. Secondary Outcome

Title	Physician Global Impress of Change (PGIC) Score
Description	Compare the end of treatment month 4 in the physician global impress of change (PGIC) for subjects treated with ISIS 546254 vs. placebo.<br>The PGIC is a one item questionnaire asking, "Since the beginning of treatment in this study, how would you describe this patient's change (if any) in their overall status?". The answer option for the PGIC is coded as: 3 = Very Much Improved, 2 = Much Improved, 1 = Minimally Improved, 0 = No Change, -1 = Minimally Worse, -2 = Much Worse, -3 = Very Much Worse, with negative scores indicating the subject's overall status has decreased while in the study and positive scores indicating the subject's overall status has increased while in the study.
Time Frame	Evaluating the treatment period, but collected at the end of treatment, up to 16 weeks

Outcome Measure Data

Analysis Population Description
[Not Specified]

Arm/Group Title	IONIS-PKKRx (ISIS 546254)	Placebo
Arm/Group Description:	Those randomized to IONIS-PKKRx (ISIS 546254) received subcutaneous injections containing 1.00 mL (200mg/mL) weekly for weeks 1-16.	Those randomized to placebo will receive subcutaneous injections containing 1.00 mL weekly for weeks 1-16.
Overall Number of Participants Analyzed	15	15
Mean (Standard Error)<br>Unit of Measure: units on a scale		
	0.867 (0.215)	1.00 (0.258)

8. Secondary Outcome

Title	Subject Global Impress of Change (SGIC) Score
Description	Compare the end of treatment month 4 in the subjects' global impression of change (SGIC) for subjects treated with ISIS 546254 vs. placebo.<br>The SGIC is a one item questionnaire asking, "Since the beginning of treatment in this study, how would you describe the change (if any) in your overall status?". The answer option for the SGIC is coded as: 3 = Very Much Improved, 2 = Much Improved, 1 = Minimally Improved, 0 = No Change, -1 = Minimally Worse, -2 = Much Worse, -3 = Very Much Worse, with negative scores indicating the subject's overall status has decreased while in the study and positive scores indicating the subject's overall status has increased while in the study.
Time Frame	Evaluating the treatment period, but collected at the end of treatment, up to 16 weeks

Outcome Measure Data

Analysis Population Description
[Not Specified]

Arm/Group Title	IONIS-PKKRx (ISIS 546254)	Placebo
Arm/Group Description:	Those randomized to IONIS-PKKRx (ISIS 546254) received subcutaneous injections containing 1.00 mL (200mg/mL) weekly for weeks 1-16.	Those randomized to placebo will receive subcutaneous injections containing 1.00 mL weekly for weeks 1-16.
Overall Number of Participants Analyzed	15	15
Mean (Standard Error)<br>Unit of Measure: units on a scale		
	0.867 (0.291)	1.13 (0.350)

Adverse Events

Time Frame	196 days
Adverse Event Reporting Description	[Not Specified]

Arm/Group Title	IONIS-PKKRx (ISIS 546254)	Placebo
Arm/Group Description	Those randomized to IONIS-PKKRx (ISIS 546254) received subcutaneous injections containing 1.00 mL (200mg/mL) weekly for weeks 1-16.	Those randomized to placebo will receive subcutaneous injections containing 1.00 mL weekly for weeks 1-16.

All-Cause Mortality

	IONIS-PKKRx (ISIS 546254)		Placebo	
	Affected / at Risk (%)		Affected / at Risk (%)	
Total	0/15 (0.00%)		0/15 (0.00%)	

Serious Adverse Events

	IONIS-PKKRx (ISIS 546254)		Placebo	
	Affected / at Risk (%)	# Events	Affected / at Risk (%)	# Events
Total	0/15 (0.00%)		2/15 (13.33%)	
Hepatobiliary disorders				
Cholecystitis *1	0/15 (0.00%)	0	1/15 (6.67%)	1
Surgical and medical procedures				
Gastrectomy *1	0/15 (0.00%)	0	1/15 (6.67%)	1

1 Term from vocabulary, MedDRA (Unspecified)
* Indicates events were collected by non-systematic assessment

Other (Not Including Serious) Adverse Events

Frequency Threshold for Reporting Other Adverse Events: 5%

	IONIS-PKKRx (ISIS 546254)		Placebo	
	Affected / at Risk (%)	# Events	Affected / at Risk (%)	# Events
Total	15/15 (100.00%)		8/15 (53.33%)	
Gastrointestinal disorders				
Nausea *1	1/15 (6.67%)	1	3/15 (20.00%)	3
General disorders				
Injection site erythema *1	15/15 (100.00%)	96	0/15 (0.00%)	0
Injection site induration *1	9/15 (60.00%)	31	0/15 (0.00%)	0
Injection site pain *1	11/15 (73.33%)	51	0/15 (0.00%)	0
Injection site pruritus *1	5/15 (33.33%)	23	0/15 (0.00%)	0
Pain *1	0/15 (0.00%)	0	4/15 (26.67%)	4
Respiratory, thoracic and mediastinal disorders				
Sinus congestion *1	1/15 (6.67%)	1	4/15 (26.67%)	4

1 Term from vocabulary, MedDRA (Unspecified)
* Indicates events were collected by non-systematic assessment

Limitations and Caveats

[Not Specified]

More Information

Certain Agreements

Principal Investigators are NOT employed by the organization sponsoring the study.

There is NOT an agreement between Principal Investigators and the Sponsor (or its agents) that restricts the PI's rights to discuss or publish trial results after the trial is completed.

Results Point of Contact

Name/Title: Jim Sly
Organization: Clinvest Research
Phone: 4178413673
EMail: jsly@clinvest.com

Responsible Party: Timothy Smith, Smith, Timothy R., M.D.
ClinicalTrials.gov Identifier: NCT03108469 History of Changes
Other Study ID Numbers: 15-001IS
First Submitted: March 27, 2017
First Posted: April 11, 2017
Results First Submitted: January 20, 2020
Results First Posted: February 5, 2020
Last Update Posted: February 12, 2020

Ionis Innovation

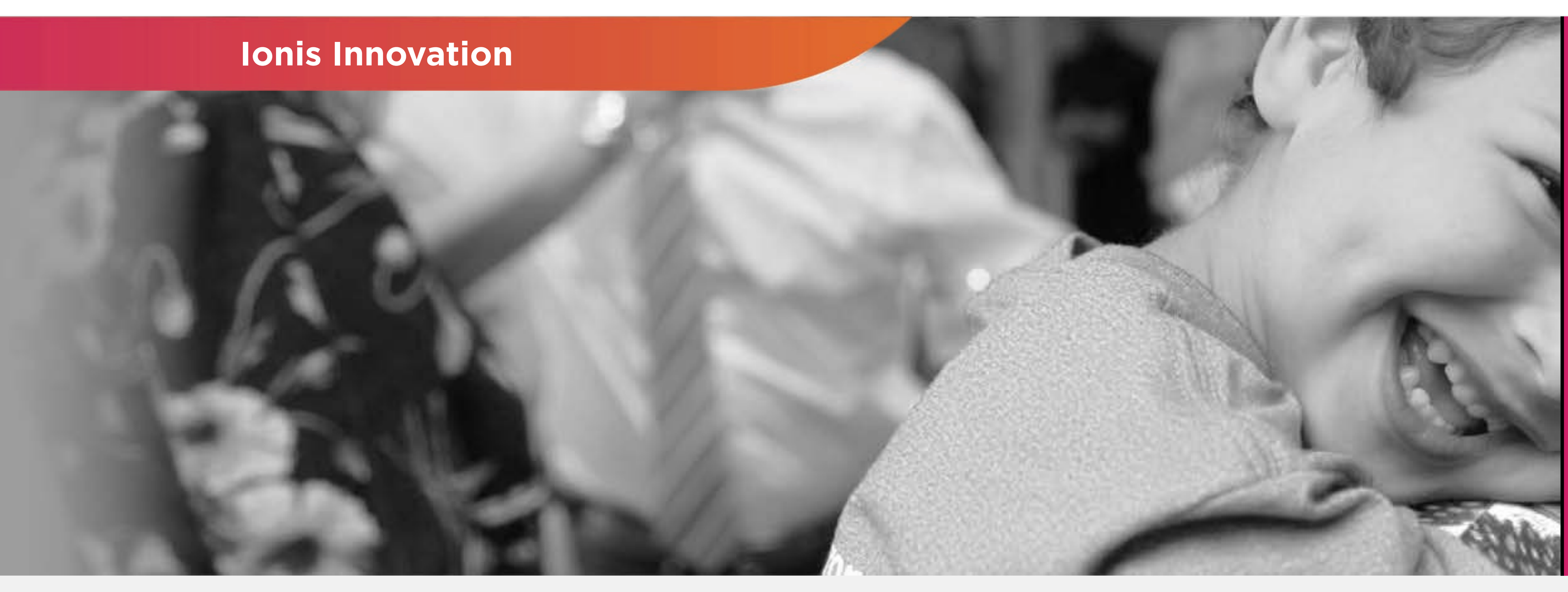

The Ionis antisense pipeline

Our **antisense technology platform** has served as a springboard for drug discovery and realized hope for patients with unmet needs. Our broad, diverse pipeline has more than 40 potential first-in-class and/or best-in-class medicines designed to treat a broad range of diseases.

The result of 30 years of perfecting and advancing **RNA targeted drug discovery and development**, our proprietary platform now provides a fast and efficient path from identification of potential causes of human diseases to the discovery of potentially life-saving medicines.

Search Filter

Hide Tools »

keyword search

Partnered | Ionis-Owned

Therapeutic Areas

Neurological | Cardiovascular | Rare | Specialty Rare | Other Medicines

Phase

Phase 1 | Phase 2 | Phase 3

Apply | Clear

Neurological

	Partner	Phase 1	Phase 2	Phase 3
**Eplontersen**<br>(TTR)<br>Hereditary ATTR Polyneuropathy	AstraZeneca**			REGISTRATION
**Ulefnersen**<br>(FUS)<br>Amyotrophic Lateral Sclerosis	Ionis-Owned			
**Tofersen**<br>(SOD1)<br>Amyotrophic Lateral Sclerosis	Biogen			
**Zilganersen***<br>(GFAP)<br>Alexander Disease	Ionis-Owned			
**IONIS-MAPT$_{Rx}$**<br>(TAU)<br>Alzheimer's Disease	Biogen			
**ION859**<br>(LRRK2)<br>Parkinson's Disease	Biogen			
**ION464**<br>(SNCA)<br>Multiple System Atrophy & Parkinson's Disease	Biogen			
**ION541***<br>(ATXN2)<br>Amyotrophic Lateral Sclerosis	Biogen			
**ION582***<br>(UBE3A-ATS)<br>Angelman Syndrome	Biogen			
**Tominersen**<br>(HTT)<br>Huntington's Disease	Roche			
**ION260**<br>(ATXN3)<br>Spinocerebellar Ataxia Type 3	Ionis-Owned			
**ION306**<br>(SMN2)<br>Spinal Muscular Atrophy	Biogen			

Cardiovascular

	Partner	Phase 1	Phase 2	Phase 3
**Eplontersen**<br>(TTR)<br>Transthyretin Amyloid Cardiomyopathy	AstraZeneca**			
**Olezarsen**<br>(ApoC-III)<br>FCS	Ionis-Owned			
**Olezarsen**<br>(ApoC-III)<br>Severe Hypertriglyceridemia	Ionis-Owned			
**Pelacarsen**<br>(Apo(a))<br>CVD	Novartis			
**Fesomersen**<br>(Factor XI)<br>Thrombotic Disorders	Ionis-Owned			
**IONIS-AGT-L$_{Rx}$**<br>(Angiotensinogen)<br>Treatment-Resistant Hypertension	Ionis-Owned			
**IONIS-AGT-L$_{Rx}$**<br>(Angiotensinogen)<br>Chronic Heart Failure with Reduced Ejection Fraction	Ionis-Owned			
**ION904**<br>(Angiotensinogen)<br>Treatment-Resistant Hypertension	Ionis-Owned			

Specialty Rare

	Partner	Phase 1	Phase 2	Phase 3
**Donidalorsen**<br>(PKK)<br>Hereditary Angioedema	Ionis-Owned			
**Sapablursen**<br>(TMPRSS6)<br>Polycythemia Vera	Ionis-Owned			

Other Medicines

	Partner	Phase 1	Phase 2	Phase 3
**Bepirovirsen**<br>(Hepatitis B Virus)<br>Hepatitis B Virus Infection	GSK			
**IONIS-FB-L$_{Rx}$**<br>(Complement Factor B)<br>IgA Nephropathy	Roche			
**IONIS-FB-L$_{Rx}$**<br>(Complement Factor B)<br>Geographic Atrophy/AMD	Roche			
**ION224**<br>(DGAT2)<br>Non-Alcoholic Steatohepatitis	Ionis-Owned			
**ION839**<br>(PNPLA3)<br>Non-Alcoholic Steatohepatitis	AstraZeneca			
**ION455**<br>(Undisclosed)<br>Non-Alcoholic Steatohepatitis	AstraZeneca			
**ION532**<br>(APOL1)<br>Chronic Kidney Disease	AstraZeneca			

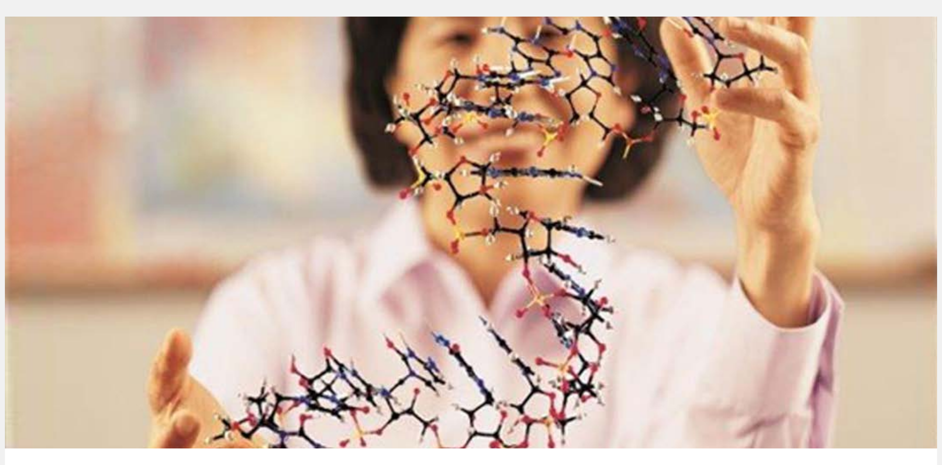

Future-creating, antisense medicines

Our antisense technology has allowed us to create treatments that disrupt the disease process, may change its course, and we hope make a positive difference in patients' lives.

Learn about our medicines »

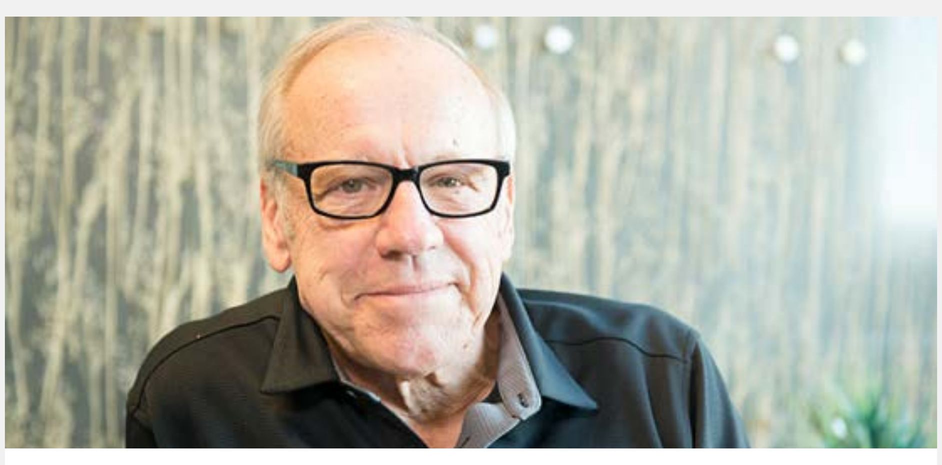

Once in a lifetime breakthroughs again and again

Meet Chuck. Active throughout his life, hATTR, a rare disease, started to rob him of the things he loved: riding his bicycle, dancing, racing motorcycles, and picking up his grandchildren. Just about to give up, news of a clinical trial renewed his hope.

See who inspires Ionis »

©1989 – 2023 Ionis Pharmaceuticals® is a registered trademark of Ionis Pharmaceuticals, Inc.
Akcea Therapeutics® is a wholly owned subsidiary of Ionis Pharmaceuticals, Inc.

We are using cookies to give you the best experience on our website.
You can find out more about which cookies we are using or switch them off in settings.
Accept

This is the classic website, which will be retired eventually. Please visit the modernized ClinicalTrials.gov instead.

Safety, Tolerability, PK, PD of ADX-324 in Healthy Volunteers and Hereditary Angioedema Patients

The safety and scientific validity of this study is the responsibility of the study sponsor and investigators. Listing a study does not mean it has been evaluated by the U.S. Federal Government. Know the risks and potential benefits of clinical studies and talk to your health care provider before participating. Read our disclaimer for details.

ClinicalTrials.gov Identifier: NCT05691361

Recruitment Status : Recruiting
First Posted : January 20, 2023
Last Update Posted : January 20, 2023

See **Contacts and Locations**

View this study on the modernized ClinicalTrials.gov

Sponsor:
ADARx Pharmaceuticals, Inc.

Information provided by (Responsible Party):
ADARx Pharmaceuticals, Inc.

Study Description

Brief Summary:
The first-in-human Phase 1 study will evaluate the safety, tolerability, pharmacokinetics (PK) and pharmacodynamics (PD) of **ADX-324** in healthy volunteers (HV) and in patients with Hereditary Angioedema (HAE).

Condition or disease	Intervention/treatment	Phase
Hereditary Angioedema	Drug: **ADX-324**<br>Drug: Placebo	Phase 1

Detailed Description:

The clinical study described in this protocol is a Phase 1, single-center study evaluating safety, tolerability, PK, and PD of ADX-324.

The study consists of 2 parts:

- Randomized, double-blind, placebo-controlled, parallel group, single ascending dose (SAD) in HV with up to 6 dose cohorts. For SAD cohorts and planned dosing; and,
- Expansion cohort in participants with Hereditary Angioedema (HAE) at selected dose from Part A and will be open label.

Study Design

Study Type : Interventional (Clinical Trial)
Estimated Enrollment : 53 participants
Allocation: Randomized
Intervention Model: Sequential Assignment
Intervention Model Description: Phase 1, Single-Center, Randomized, Placebo-Controlled, Double Blind Single Ascending Dose Study in HV with expansion into HAE
Masking: Quadruple (Participant, Care Provider, Investigator, Outcomes Assessor)
Masking Description: Double blinded
Primary Purpose: Treatment
Official Title: A Phase 1, Randomized, Placebo-Controlled, Double Blind Single Ascending Dose Study in Healthy Volunteers and an Expansion Cohort in Patients With Hereditary Angioedema to Evaluate the Safety, Tolerability, PK and PD of **ADX-324**
Actual Study Start Date : December 14, 2022
Estimated Primary Completion Date : January 2, 2024
Estimated Study Completion Date : November 26, 2024

Resource links provided by the National Library of Medicine NIH NLM

MedlinePlus Genetics related topics: Hereditary angioedema

MedlinePlus related topics: Safety

Genetic and Rare Diseases Information Center resources: Hereditary Angioedema

U.S. FDA Resources

Arms and Interventions

Arm	Intervention/treatment
Experimental: PART A - Active **ADX-324** administered to HV<br>For each cohort in Part A (SAD), 8 participants will be randomized in a 3:1 ratio; 6 participants to active (**ADX-324**): 2 participants to control (matched placebo). Randomization will be on Day 1. Initially, 2 sentinel participants (1 active and 1 placebo) will be randomized and dosed. The sentinel participants will be evaluated for safety. The investigator's assessment and the independent medical monitor will decide upon the randomization and dosing of the 6 remaining participants (5 active and 1 placebo) according to the randomization schedule.	Drug: **ADX-324**<br>siRNA duplex oligonucleotide<br>Other Name: siRNA
Placebo Comparator: PART A- Placebo administered to HV<br>For each cohort in Part A (SAD), 8 participants will be randomized in a 3:1 ratio; 6 participants to active (**ADX-324**): 2 participants to control (matched placebo). Randomization will be on Day 1. Initially, 2 sentinel participants (1 active and 1 placebo) will be randomized and dosed. The sentinel participants will be evaluated for safety. The investigator's assessment and the independent medical monitor will decide upon the randomization and dosing of the 6 remaining participants (5 active and 1 placebo) according to the randomization schedule.	Drug: Placebo<br>saline<br>Other Name: Saline
Experimental: PART B - **ADX-324** administered to HAE participants<br>This will be initiated at the dose level determined by the Safety Review Committee from SAD in HVs. The treatment of HAE participants is an open-label study.	Drug: **ADX-324**<br>siRNA duplex oligonucleotide<br>Other Name: siRNA

Outcome Measures

Primary Outcome Measures :

1. Safety in Healthy Volunteers [Time Frame: 183 days]

   To evaluate the safety and tolerability of **ADX-324** in HVs by incidence, relationship, and severity of adverse events and serious adverse events

2. Safety in Healthy Volunteers [Time Frame: 183 days]

   To evaluate the safety and tolerability of **ADX-324** in HVs by change in baseline electrocardiogram (ECG) parameters (PR, QRS, QT, and QTcF intervals)

3. Safety in Hereditary Angioedema [Time Frame: 183 days]

   To evaluate the safety and tolerability of **ADX-324** in HAE by incidence, relationship, and severity of adverse events and serious adverse events

Secondary Outcome Measures :

1. Pharmacokinetics in Healthy Volunteers [Time Frame: 183 days]

   To characterize the Pharmacokinetics of **ADX-324** in HVs by measuring the Maximum observed concentration (Cmax)

2. Pharmacokinetics in Healthy Volunteers [Time Frame: 183 days]

   To characterize the Pharmacokinetics of **ADX-324** in HVs by measuring the Time to Cmax (Tmax)

3. Pharmacokinetics in Healthy Volunteers [Time Frame: 183 days]

   To characterize the Pharmacokinetics of **ADX-324** in HVs by measuring the Area under the concentration-time curve from 0 to time of last quantifiable concentration (AUC0-last)

4. Pharmacokinetics in Healthy Volunteers [Time Frame: 183 days]

   To characterize the Pharmacokinetics of **ADX-324** in HVs by measuring the Area under the concentration-time curve from 0 to infinity (AUC0-∞)

5. Pharmacokinetics in Healthy Volunteers [Time Frame: 183 days]

   To characterize the Pharmacokinetics of **ADX-324** in HVs by measuring the Apparent terminal half-life (t½)

6. Pharmacokinetics in Healthy Volunteers [Time Frame: 183 days]

   To characterize the Pharmacokinetics of **ADX-324** in HVs by measuring the Terminal elimination rate constant (λz)

7. Pharmacokinetics in Healthy Volunteers [Time Frame: 183 days]

   To characterize the Pharmacokinetics of **ADX-324** in HVs by measuring the Total apparent body clearance (CL/F)

8. Pharmacokinetics in Healthy Volunteers [Time Frame: 183 days]

   To characterize the Pharmacokinetics of **ADX-324** in HVs by measuring the Apparent volume of distribution (Vz/F)

9. Pharmacodynamics in Healthy Volunteers [Time Frame: 30 hours]

   To characterize the PD of **ADX-324** in HVs by the Change from base in plasma concentrations over time of pre Kallikrein (PKK)

10. Pharmacodynamics in Healthy Volunteers [Time Frame: 30 hours]

    To characterize the PD of **ADX-324** in HVs by the Change from base in plasma concentrations over time of Kallikrein (KK)

11. Pharmacokinetics in Hereditary Angioedema [Time Frame: 30 hours]

    To characterize the PD of **ADX-324** in HAE by Maximum observed concentration (Cmax) of **ADX-324**

12. Pharmacokinetics in Hereditary Angioedema [Time Frame: 30 hours]

    To characterize the PD of **ADX-324** in HAE by Time to Cmax (Tmax) of **ADX-324**

13. Pharmacokinetics in Hereditary Angioedema [Time Frame: 30 hours]

    To characterize the PD of **ADX-324** in HAE by Area under the concentration-time curve from 0 to time of last quantifiable concentration (AUC0-last) of **ADX-324**

14. Pharmacokinetics in Hereditary Angioedema [Time Frame: 30 hours]

    To characterize the PD of **ADX-324** in HAE by Area under the concentration-time curve from 0 to infinity (AUC0-∞) of **ADX-324**

15. Pharmacokinetics in Hereditary Angioedema [Time Frame: 30 hours]

    To characterize the PD of **ADX-324** in HAE by Apparent terminal half-life (t½)

16. Pharmacokinetics in Hereditary Angioedema [Time Frame: 30 hours]

    To characterize the PD of **ADX-324** in HAE by Terminal elimination rate constant (λz)

17. Pharmacokinetics in Hereditary Angioedema [Time Frame: 30 hours]

    To characterize the PD of **ADX-324** in HAE by Total apparent body clearance (CL/F)

18. Pharmacokinetics in Hereditary Angioedema [Time Frame: 30 hours]

    To characterize the PD of **ADX-324** in HAE by Apparent volume of distribution (Vz/F)

19. Pharmacodynamics in Hereditary Angioedema [Time Frame: 183 days]

    To characterize the PD of **ADX-324** in HV by Change from base in plasma concentrations over time pre-kallikrein (PKK)

20. Pharmacodynamics in Hereditary Angioedema [Time Frame: 183 days]

    To characterize the PD of **ADX-324** in HAE by Change from base in plasma concentrations over time kallikren (KK)

Eligibility Criteria

Information from the National Library of Medicine NIH NLM

Choosing to participate in a study is an important personal decision. Talk with your doctor and family members or friends about deciding to join a study. To learn more about this study, you or your doctor may contact the study research staff using the contacts provided below. For general information, Learn About Clinical Studies.

Ages Eligible for Study: 18 Years to 55 Years (Adult)
Sexes Eligible for Study: All
Accepts Healthy Volunteers: Yes

Criteria

Part A - HV

Inclusion Criteria:

1. Male and female adults 18 to 55 years old
2. Body mass index (BMI) between 18 and 30 kg/m2
3. Contraception use by men or women should be consistent with local regulations regarding the methods of contraception for those participating in clinical studies
4. Willing and able to provide informed consent and comply with all study visits

Exclusion Criteria:

1. Any significant medical history
2. Active malignancy and/or history of malignancy in the past 5 years
3. History of liver disease, Gilbert's syndrome, or abnormal liver function test
4. Estimated creatinine clearance <60 mL/min or serum creatinine > 1.5-fold upper limit of normal.
5. Any active infection or acute illness
6. Major surgery or significant traumatic injury occurring within 3 months
7. Have any other conditions that, in the opinion of the Investigator or Sponsor, would make the participant unsuitable for inclusion, or could interfere with the participant participating in or completing the study.
8. Positive serology tests (HepB, Hep C, HIV)
9. Use of any prescription, vaccines, supplements/vitamins, or over-the counter medication
10. Treatment with another investigational product within 30 days prior to the first study drug administration
11. Known any clinically significant allergic reactions which, in the opinion of the Investigator, would interfere with the volunteer's ability to participate in the study
12. Known hypersensitivity to any of the study drug ingredients.
13. Pregnancy, intent to become pregnant during the course of the study, or lactating women

Part B - HAE

Inclusion Criteria:

1. Male and female ≥18 years old, inclusive, at the time of signing the PICF
2. Confirmed diagnosis of HAE Types I or II
3. Evidence of an average of (at least) one HAE attack per month
4. Participants must have access to, and the ability to use, acute medication(s) to treat angioedema attacks.
5. Body mass index (BMI) between 18 and 30 kg/m2
6. Contraception use by men or women should be consistent with local regulations regarding the methods of contraception for those participating in clinical studies
7. Willing and able to provide informed consent and comply with all study visits

Exclusion Criteria:

1. Concurrent diagnosis of any other type of chronic angioedema
2. History of clinically significant arterial or venous thrombosis, or current history of a clinically significant prothrombotic risk.
3. Any significant medical history
4. Active malignancy and/or history of malignancy in the past 5 years
5. Any active infection or acute illness, inclusive of cold/flu or COVID-19, within 30 days prior to the first study drug administration.
6. Major surgery or significant traumatic injury occurring within 3 months prior to signature of the PICF
7. Have any other conditions that, in the opinion of the Investigator or Sponsor, would make the participant unsuitable for inclusion, or could interfere with the participant participating in or completing the study.
8. Positive serology tests for human immunodeficiency virus (HIV), hepatitis B surface antigen (HBsAg), or hepatitis C virus (HCV).
9. Use of C1-INH products, androgens, antifibrinolytics or other small molecule medications for routine prophylaxis within four half-lives prior to screening
10. Must have documented evidence of medical history of HAE attacks
11. Use of any prescription, vaccines, supplements/vitamins, or over-the counter medication (with the exception of oral contraceptives) within 7 days prior to the first study drug administration.
12. Treatment with another investigational product or biologic agent within 30 days prior to the study drug administration
13. History or presence of alcohol abuse or drug use within 30 days prior to the first study drug administration and throughout the study.
14. Blood donation of 50 to 499 mL within 30 days prior to the first study drug administration or of >499 mL within 60 days prior to the first study drug administration.
15. Pregnancy, intent to become pregnant during the course of the study, or lactating women.

Contacts and Locations

Information from the National Library of Medicine NIH NLM

To learn more about this study, you or your doctor may contact the study research staff using the contact information provided by the sponsor.

Please refer to this study by its ClinicalTrials.gov identifier (NCT number): ***NCT05691361***

Contacts

Contact: CMAX Reception +610870887900 Jane.kelly@cmax.com.au

Locations

Australia, South Australia

CMAX Clinical Research — **Recruiting**
Adelaide, South Australia, Australia, 5000
Contact: CMAX Reception +61 0870887900 jane.kelly@cmax.com.au

Sponsors and Collaborators

ADARx Pharmaceuticals, Inc.

Investigators

Principal Investigator: Nicholas Farinola, MD CMAX Clinical Research Pty Ltd

More Information

Responsible Party: ADARx Pharmaceuticals, Inc.
ClinicalTrials.gov Identifier: NCT05691361 History of Changes
Other Study ID Numbers: **ADX-324-**101
First Posted: January 20, 2023 Key Record Dates
Last Update Posted: January 20, 2023
Last Verified: January 2023

Individual Participant Data (IPD) Sharing Statement:
Plan to Share IPD: Undecided

Studies a U.S. FDA-regulated Drug Product: No
Studies a U.S. FDA-regulated Device Product: No

Additional relevant MeSH terms:

Angioedema	Hypersensitivity, Immediate
Angioedemas, Hereditary	Hypersensitivity
Vascular Diseases	Immune System Diseases
Cardiovascular Diseases	Hereditary Complement Deficiency Diseases
Urticaria	Primary Immunodeficiency Diseases
Skin Diseases, Vascular	Genetic Diseases, Inborn
Skin Diseases	Immunologic Deficiency Syndromes

News Release

About KalVista
Products & Pipeline
For Patients
For Healthcare Providers
For Investors
Annual Meeting
Stock Information
Stock Quote & Chart
Historic Price Lookup
Investment Calculator
News Releases
Event Calendar
Presentations
SEC Filings
Analyst Coverage
Corporate Governance

KalVista Pharmaceuticals Reports Phase 2 Clinical Trial Results in Patients with Diabetic Macular Edema

December 9, 2019 at 7:00 AM EST

PDF Version

– KVD001 Does Not Meet Primary Endpoint –

– Pre-Planned Analyses Show Clinical Benefit on Vision –

– KVD001 Generally Safe and Well Tolerated –

CAMBRIDGE, Mass. & SALISBURY, England--(BUSINESS WIRE)--Dec. 9, 2019-- KalVista Pharmaceuticals, Inc. (NASDAQ: KALV), a clinical stage pharmaceutical company focused on the discovery, development, and commercialization of small molecule protease inhibitors, today announced results of the Phase 2 clinical trial evaluating the use of KVD001 in patients with diabetic macular edema (DME).

The KVD001 Phase 2 clinical trial study was designed to evaluate patients who were poor responders to previous treatment with anti-VEGF therapy. The primary efficacy endpoint in the trial was change in best corrected visual acuity (BCVA) at 16 weeks compared to sham. The 6µg dose showed a difference of +2.6 letters versus sham, which was not statistically significant (p=0.223), and the 3µg dose showed a difference of +1.5 letters (p=0.465). No significant differences were observed in the secondary endpoints of central subfield thickness (CST) or the diabetic retinopathy severity scale (DRSS). KVD001 was generally safe and well tolerated with no drug-related serious adverse events.

"This was the first study to evaluate the efficacy of a plasma kallikrein inhibitor in DME," said Andrew Crockett, Chief Executive Officer of KalVista. "There is a significant population of DME patients who do not respond sufficiently to anti-VEGF therapies, and we want to express our gratitude to these patients as well as the healthcare providers and others who participated. Although the study did not meet the primary endpoint, KVD001 demonstrated what we believe is an important dose responsive clinical benefit on vision in the overall population. In addition, we identified a substantial proportion of patients who experienced a more robust response to treatment, that we believe warrants further study. These data and the safety profile also support continued evaluation of oral plasma kallikrein inhibitors as a treatment for DME."

In the overall study population, KVD001 demonstrated a protection against vision loss. In the sham treated group 54.5% of patients experienced a reduction in vision compared to 32.5% in the 6µg dose (p=0.042). The study also included a pre-specified subgroup analysis investigating the impact of baseline visual acuity on response. After excluding those patients with the most severe vision loss (visual acuity of <55 letters at baseline), the remaining 70% of the total patient population showed a difference in BCVA compared to sham of 4.9 letters (p=0.056) at the 6µg dose.

"This trial studied a challenging DME patient population with significant persistent vision loss despite prior therapies," said Lloyd Paul Aiello, MD, PhD, Professor of Ophthalmology, Harvard Medical School. "These study data support the possibility that plasma kallikrein inhibition prevents worsening vision in patients with DME and that KVD001 warrants additional study in this regard as a method to treat this disease."

KVD001 is a small molecule plasma kallikrein inhibitor administered by intravitreal injection. The Phase 2 trial consisted of 129 patients who had previously been treated with anti-VEGF therapy, and still had significant edema and reduced visual acuity. The sham-controlled, double-masked clinical trial evaluated two dose levels of KVD001, 3µg and 6µg. Four intravitreal injections or sham were administered over three months with a three month follow up period. The study was conducted at 38 sites in the United States.

KVD001 is subject to an option agreement with Merck, signed in 2017. Under the terms of the agreement, KalVista will provide to Merck a package of clinical and other data, after which Merck will have a specified period of time to determine whether to exercise the option. Exercise of the option by Merck will result in a payment due to KalVista, as well as future potential milestone and royalty payments. KalVista will provide further public updates once Merck has notified KalVista of its decision or the option exercise period has expired.

A summary presentation of the KVD001 data findings has been posted to the investors section of the KalVista website, and will be available for the next 30 days.

About KalVista Pharmaceuticals, Inc.

KalVista Pharmaceuticals, Inc. is a pharmaceutical company focused on the discovery, development, and commercialization of small molecule protease inhibitors for diseases with significant unmet need. The initial focus is on inhibitors of plasma kallikrein, which is an important component of the body's inflammatory response and which, in excess, can lead to increased vascular permeability, edema and inflammation. KalVista has developed a proprietary portfolio of novel, small molecule plasma kallikrein inhibitors initially targeting hereditary angioedema (HAE) and diabetic macular edema (DME). The Company has created a structurally diverse portfolio of oral plasma kallikrein inhibitors and is advancing multiple drug candidates for HAE as well as DME. The Company has selected KVD900 as its program to be advanced as an on-demand therapy for acute HAE attacks and commenced a Phase 2 proof-of-concept study in HAE patients in late 2018. In DME, an intravitreally administered plasma kallikrein inhibitor known as KVD001, completed a Phase 2 clinical trial in 2019.

For more information, please visit www.kalvista.com.

Forward-Looking Statements

This press release contains "forward-looking" statements within the meaning of the safe harbor provisions of the U.S. Private Securities Litigation Reform Act of 1995. Forward-looking statements can be identified by words such as: "anticipate," "intend," "plan," "goal," "seek," "believe," "project," "estimate," "expect," "strategy," "future," "likely," "may," "should," "will" and similar references to future periods. These statements are subject to numerous risks and uncertainties that could cause actual results to differ materially from what we expect. Examples of forward-looking statements include, among others, available funding, our cash runway, potential future clinical trial timing and results. Further information on potential risk factors that could affect our business and its financial results are detailed in the annual report on Form 10-K filed on July 15, 2019 and other reports as filed from time to time with the Securities and Exchange Commission. We undertake no obligation to publicly update any forward-looking statement, whether written or oral, that may be made from time to time, whether as a result of new information, future developments or otherwise.

View source version on businesswire.com: https://www.businesswire.com/news/home/20191209005372/en/

Source: KalVista Pharmaceuticals, Inc.

KalVista Pharmaceuticals, Inc.
Leah Monteiro
Senior Director, Corporate Communications & Investor Relations
857-999-0808
leah.monteiro@kalvista.com

Copyright © 2023 | All rights reserved.

TVST/IOVS Special Issue Call for Papers
Anti-VEGF
Submission deadline: Oct. 1

June 2020
Volume 61, Issue 7
ISSUE

FREE

ARVO Annual Meeting Abstract | June 2020

Nonclinical safety and pharmacology of RZ402, a plasma kallikrein inhibitor, for the treatment of diabetic macular edema as a daily oral therapy

Sankaram Mantripragada; Sharon Xueyan Wang; Tamie Chilcote; Sukanto Sinha

Author Affiliations & Notes

Investigative Ophthalmology & Visual Science June 2020, Vol.61, 307. doi:

891 Views
View Metrics
Advertisement
SHARE TOOLS

Abstract

Purpose : Excessive retinal vascular permeability (VP) is the key defining pathology underlying the development of diabetic macular edema (DME). The serine protease plasma kallikrein (PK) generates the vasoactive peptide bradykinin, a known mediator of VP. PK-null mice are protected from increased retinal VP induced by diabetes, or by VEGF, and we previously showed that systemic administration of the selective small-molecule PK inhibitor (PKI) RZ402 (formerly ASP440) normalized the elevated retinal VP in rodent models of diabetes (Diabetes 60:1590–1598, 2011.) We are developing RZ402 as a potential treatment for DME.

Methods : Single and repeat-dose toxicology, respiratory safety, cardiovascular safety, neurobehavioral and toxicokinetic studies were performed in Sprague-Dawley (SD) rats, cynomolgus monkeys, or both. Oral dosing was carried out via gavage of a suspension of RZ402 in 0.4% hydroxymethyl cellulose. Administered p.o. doses ranged from 0, 70, 100, 200, 300, 400, 800 and 1000 mg/kg. Drug exposure was determined by analyzing plasma samples by LC-MS/MS.

Results : RZ402 exhibited excellent ADME properties in rat and monkey, with moderate clearance, excreted renally as the unchanged parent drug. Plasma levels of drug were maintained above the previously established EC50 level of 50 nM for up to 24h after oral dosing. No adverse effects were observed following seven-day repeat oral dosing in rats at up to 1000 mg/kg, at therapeutic exposure multiples >500-fold. RZ402 was well-tolerated and exhibited dose-proportional exposures up to 800 mg/kg when dosed orally in monkeys, and repeat oral dosing for seven days at up to 400 mg/kg did not result in adverse clinical observations nor any meaningful changes in hematology and clinical chemistry measures. No histopathological changes were observed in any organs. Additionally, no adverse effects were observed in respiratory and CNS safety pharmacology studies in rats (dosed orally at up to 1000 mg/kg) or in a cardiovascular safety pharmacology study in monkeys (dosed orally at up to 400 mg/kg).

Conclusions : In summary, RZ402 is a safe and effective preclinical drug candidate for DME, with an ADME and safety profile in rodent and non-rodent species supporting the potential for once-daily oral dosing in the clinic.

This is a 2020 ARVO Annual Meeting abstract.

This work is licensed under a Creative Commons Attribution-NonCommercial-NoDerivatives 4.0 International License.

This site uses cookies. By continuing to use our website, you are agreeing to our privacy policy. Accept

PRESS RELEASES

REZOLUTE ANNOUNCES INITIATION OF A PHASE 2 STUDY OF RZ402 IN PATIENTS WITH DIABETIC MACULAR EDEMA

December 15, 2022 7:00am EST

Download as PDF

RZ402 IS AN ORAL THERAPY BEING DEVELOPED AS A POTENTIAL ALTERNATIVE TO INVASIVE AND SUBOPTIMAL INJECTIONS INTO THE EYE

REDWOOD CITY, Calif., Dec. 15, 2022 (GLOBE NEWSWIRE) -- Rezolute, Inc. (Nasdaq: RZLT), a clinical-stage biopharmaceutical company dedicated to developing transformative therapies with the potential to shift the treatment paradigms of devastating metabolic diseases, today announced that the Company has initiated a Phase 2 proof-of-concept study for RZ402, a plasma kallikrein inhibitor (PKI) being developed as an oral therapy for the treatment of Diabetic Macular Edema (DME). The current standard of care for DME is anti-vascular growth factor (anti-VEGF) injections into the eye, requiring repeated administration over recurring periods of time to preserve vision, with significant treatment burden and occasional serious side effects. The invasive route of administration, coupled with inadequate responsiveness in some patients, leads to overall undertreatment and suboptimal vision outcomes in DME patients.

“The initiation of this Phase 2 study is an important milestone in our mission to address the serious unmet need in the current DME treatment paradigm,” said Raj Agrawal, M.D., Vice President, and Head of Ophthalmological Clinical Development at Rezolute. “By targeting an alternative pathway and route of administration to the current standard of care, we believe that orally-administered RZ402 has the potential to be a less burdensome and more beneficial treatment option for all patients suffering with DME, including the approximately 50% of patients that don’t adequately respond to anti-VEGFs.”

“We believe that RZ402 represents the potential for a significant change in the treatment paradigm of DME,” said Brian Roberts, M.D., Chief Medical Officer at Rezolute. “By targeting this microvascular diabetes complication with an oral-systemic PKI, analogous to the management of other diabetes complications, RZ402 has the potential to improve overall clinical outcomes by driving earlier treatment intervention and preventing disease progression and vision loss for patients with DME. We look forward to building upon the positive data generated in our Phase 1b trial and remain excited by the broad clinical potential of RZ402 to more effectively treat diseases associated with excessive kallikrein-kinin activity.”

The Phase 2 study is a multi-center, randomized, double-masked, placebo-controlled, parallel-arm study to evaluate the safety, efficacy, and pharmacokinetics of RZ402 administered as monotherapy over a 12-week treatment period in participants with DME who are naïve to or have received limited anti-VEGF injections. The study population will include DME patients with mild to moderate Non-Proliferative Diabetic Retinopathy (NPDR), Central Subfield Thickness (CST) of ≥320 µm (or corresponding values), and Best-Corrected Visual Acuity (BCVA) of ≤78 Early Treatment Diabetic Retinopathy Study (ETDRS) letters (≤20/25 on Snellen chart). Patients who have previously received more than three anti-VEGF injections prior to the study will be excluded.

Eligible participants will be randomized equally, to one of three RZ402 active treatment arms at doses of 50, 200, and 400 mg, or a placebo control arm, and will receive study drug once daily for 12 weeks, before completing a four-week follow-up. The study is expected to enroll approximately 100 patients overall, across approximately 25 investigational sites in the United States. The principal endpoints of the trial include the change in CST, as measured by Spectral Domain Ocular Coherence Tomography (SD-OCT), the change in visual acuity by ETDRS scale, the repeat dose pharmacokinetics of RZ402 in patients with DME, and the safety and tolerability of RZ402.

About Diabetic Macular Edema (DME)
Diabetic retinopathy (DR) affects approximately one third of adults with diabetes and is the leading cause of vision loss in the working age population. DME is a severe vision-threatening complication of DR characterized by swelling of the retina and thickening of the macula, the part of the eye that is responsible for high-resolution vision. Anti-vascular growth factor (anti-VEGF) injections into the eye are the current standard of care for DME, requiring repeated administration over recurring periods of time to preserve vision. Due to their invasive route of administration and occasional serious side effects, there is a tendency to delay treatment until later in the disease course, and long-term compliance with eye injection regimens can be difficult for patients. Coupled with inadequate responsiveness in some patients, this leads to overall undertreatment and suboptimal vision outcomes in DME patients.

About RZ402
RZ402 is a selective and potent PKI being developed as a potential once-daily oral therapy for the chronic treatment of DME. By inhibiting the formation of kallikrein, RZ402 is designed to block downstream bradykinin production and the pro-inflammatory, pro-coagulant and fluid leaking contact-activation cascade.

Results from the Phase 1b multiple ascending dose (MAD) study showed that RZ402 was readily bioavailable with dose-dependent increases in systemic exposures. Results at both peak and 24-hour trough substantially exceeded target concentrations based on a combination of in-vitro and in-vivo profiling. RZ402 was generally safe and well-tolerated, including at higher doses than previously tested in the single ascending dose (SAD) study. There were no serious adverse events, adverse drug reactions, or identified risks.

RZ402 has been shown to reduce and prevent retinal vascular leakage in animal models by 80-90%.

About the Contact Activation Kallikrein-Kinin System
The contact-activation kallikrein-kinin system promotes increased vascular permeability and inflammation via key downstream mediators, including bradykinin, and activation of the intrinsic pathway of coagulation. Pathophysiologic upregulation of this system has been linked to a variety of diseases which are characterized by vascular dysfunction, including diabetic macular edema.

About Rezolute, Inc.
Rezolute strives to disrupt current treatment paradigms by developing transformative therapies for devastating rare and chronic metabolic diseases. Its novel therapies hold the potential to both significantly improve outcomes and reduce the treatment burden for patients, the treating physician, and the healthcare system. Patient, clinician, and advocate voices are integrated in the Company’s drug development process, enabling Rezolute to boldly address a range of severe conditions. Rezolute is steadfast in its mission to create profound, positive, and lasting impact on patients’ lives. The Company’s lead clinical asset, RZ358, is in late-stage development for the treatment of congenital hyperinsulinism, a rare pediatric endocrine disorder. Rezolute is also developing RZ402, an orally available plasma kallikrein inhibitor, for the treatment of diabetic macular edema. For more information, visit www.rezolutebio.com or follow us on Twitter.

Forward-Looking Statements
This release, like many written and oral communications presented by Rezolute and our authorized officers, may contain certain forward-looking statements regarding our prospective performance and strategies within the meaning of Section 27A of the Securities Act and Section 21E of the Securities Exchange Act of 1934, as amended. We intend such forward-looking statements to be covered by the safe harbor provisions for forward-looking statements contained in the Private Securities Litigation Reform Act of 1995 and are including this statement for purposes of said safe harbor provisions. Forward-looking statements, which are based on certain assumptions and describe future plans, strategies, and expectations of Rezolute, are generally identified by use of words such as "anticipate," "believe," "estimate," "expect," "intend," "plan," "project," "seek," "strive," "try," or future or conditional verbs such as "could," "may," "should," "will," "would," or similar expressions. Our ability to predict results or the actual effects of our plans or strategies is inherently uncertain. Accordingly, actual results may differ materially from anticipated results. Readers are cautioned not to place undue reliance on these forward-looking statements, which speak only as of the date of this release. Except as required by applicable law or regulation, Rezolute undertakes no obligation to update these forward-looking statements to reflect events or circumstances that occur after the date on which such statements were made. Important factors that may cause such a difference include any other factors discussed in Rezolute’s filings with the SEC, including the Risk Factors contained in the Rezolute’s Annual Report on Form 10-K and Quarterly Reports on Form 10-Q, which are available at the SEC’s website at www.sec.gov. You are urged to consider these factors carefully in evaluating the forward-looking statements in this release and are cautioned not to place undue reliance on such forward-looking statements, which are qualified in their entirety by this cautionary statement.

Investor:

Chiti Chopra
Director, Corporate Strategy
investor-relations@rezolutebio.com
408-606-0789

Kimberly Minarovich/Carrie McKim
Argot Partners
rezolute@argotpartners.com
212-600-1902

Media:

Ingrid Mezo
Canale Communications, Inc.
ingrid.mezo@canalecomm.com
301-473-2881

REZOLUTE

Source: Rezolute, Inc.
Released December 15, 2022

©2023 Rezolute, Inc.

This is the classic website, which will be retired eventually. Please visit the modernized ClinicalTrials.gov instead.

Notice to API users: Read about imminent changes impacting usage of the ClinicalTrials.gov API.

NIH U.S. National Library of Medicine
ClinicalTrials.gov

Find Studies About Studies Submit Studies Resources About Site PRS Login

Hemodialysis.-Induced Hypotension Therapy for End Stage Kidney Disease (Hinder)

The safety and scientific validity of this study is the responsibility of the study sponsor and investigators. Listing a study does not mean it has been evaluated by the U.S. Federal Government. Know the risks and potential benefits of clinical studies and talk to your health care provider before participating. Read our disclaimer for details.

ClinicalTrials.gov Identifier: NCT05297786

Recruitment Status: Recruiting
First Posted: March 28, 2022
Last Update Posted: August 5, 2022
See Contacts and Locations

View this study on the modernized ClinicalTrials.gov

Sponsor:
Vanderbilt University Medical Center

Information provided by (Responsible Party):
Jorge Gamboa, Vanderbilt University Medical Center

Study Details | Tabular View | No Results Posted | Disclaimer | How to Read a Study Record

Study Description

Brief Summary:
Bradykinin is a potent vasodilator that is formed by the activation of the kallikrein-kinin system. We and others have shown that bradykinin increased during hemodialysis; however, the role of bradykinin in dialysis-induced hypotension (DIH) has not been evaluated. Preliminary results from a pilot clinical trial showed that bradykinin B2 receptor blockade with icatibant prevents excessive blood pressure during hemodialysis. Thus, in this study, we will test the overarching hypothesis that blockade plasma kallikrein with **lanadelumab** would ameliorate the reduction of blood pressure during hemodialysis in patients who are prone to DIH. For this purpose, we will conduct a parallel arm, double-blind placebo-controlled trial, using **lanadelumab** to evaluate the occurrence of

Condition or disease	Intervention/treatment	Phase
Hemodialysis Complication<br>Hypotension of Hemodialysis	Drug: **Lanadelumab** Injection [**Takhzyro**]<br>Drug: Placebo	Phase 2

Study Design

Study Type :	Interventional (Clinical Trial)
Estimated Enrollment :	32 participants
Allocation:	Randomized
Intervention Model:	Parallel Assignment
Masking:	Quadruple (Participant, Care Provider, Investigator, Outcomes Assessor)
Primary Purpose:	Prevention
Official Title:	Prevention of Dialysis-Induced Hypotension by Inhibiting Plasma Kallikrein
Actual Study Start Date :	July 27, 2022
Estimated Primary Completion Date :	June 2024
Estimated Study Completion Date :	December 2024

Resource links provided by the National Library of Medicine NIH NLM

MedlinePlus related topics: Dialysis Low Blood Pressure

U.S. FDA Resources

Arms and Interventions

Arm	Intervention/treatment
Experimental: **Lanadelumab**<br>Subjects will receive **lanadelumab** (300 mg) subcutaneously at the beginning of the study and 14 days later	Drug: **Lanadelumab** Injection [**Takhzyro**]<br>**Lanadelumab** 300 mg subcutaneous injection
Placebo Comparator: Placebo<br>Subjects will receive placebo subcutaneously at the beginning of the study and 14 days later	Drug: Placebo<br>Placebo injection

Outcome Measures

Primary Outcome Measures :

1. Blood Pressure during hemodialysis [Time Frame: From the second week of the study until the end (week 6)]
   Average blood pressure during hemodialysis of three hemodialysis sessions after the second week of treatment.

Secondary Outcome Measures :

1. Hypotensive episodes [Time Frame: From the second week of the study until the end (week 6)]
   Number of hypotensive episodes during hemodialysis after the second week of treatment

Eligibility Criteria

Information from the National Library of Medicine NIH NLM

Choosing to participate in a study is an important personal decision. Talk with your doctor and family members or friends about deciding to join a study. To learn more about this study, you or your doctor may contact the study research staff using the contacts provided below. For general information, Learn About Clinical Studies.

Ages Eligible for Study:	18 Years to 85 Years (Adult, Older Adult)
Sexes Eligible for Study:	All
Accepts Healthy Volunteers:	No

Criteria

Inclusion Criteria:

- Subjects age 18 to 85 years
- On thrice-weekly hemodialysis for at least six months
- Clinically stable, adequately dialyzed (single-pool Kt/V > 1.2), with polysulphone membrane for at least three consecutive months before the study
- Subjects with pre-dialytic systolic blood pressure between 110 and 170 mmHg.
- Subjects with a reduction of systolic blood pressure during hemodialysis equal to or greater than 30 mmHg, with associated symptoms such as nausea, vomiting, muscle cramps, dizziness, or anxiety.
- Hypotensive episodes should occur four times or more in four weeks (12 hemodialysis sessions).

Exclusion Criteria:

- Subjects with intradialytic hypotension that require the use of pharmacological intervention such as midodrine or vasopressin
- Subjects with pre-dialytic systolic blood pressure greater than 170 mmHg or diastolic blood pressure greater than 110 mmHg
- History of myocardial infarction or cerebrovascular event within 3 months
- History of serious hemorrhage (including cerebral hemorrhage) in the past 6 months
- Advanced liver disease
- Ejection fraction less than 30%
- Anticipated live donor kidney transplant
- A history of poor adherence to hemodialysis or medical regimen
- Severe anemia (hemoglobin less than 8 g/dl) requiring blood transfusions
- Use of immunosuppressive drugs within one month before study enrollment
- Active connective tissue disease
- History of acute infections disease within one month before study enrollment
- Inability to provide consent
- Pregnancy

Contacts and Locations

Information from the National Library of Medicine NIH NLM

To learn more about this study, you or your doctor may contact the study research staff using the contact information provided by the sponsor.

Please refer to this study by its ClinicalTrials.gov identifier (NCT number): ***NCT05297786***

Contacts

Contact: Jorge L Gamboa, M.D. Ph.D. 6153434176 jorge.gamboa@vumc.org

Locations

United States, Tennessee

Vanderbilt University Medical Center **Recruiting**
Nashville, Tennessee, United States, 37232
Contact: Jorge L Gamboa, M.D. Ph.D. 615-343-4176 jorge.gamboa@vanderbilt.edu
Principal Investigator: Jorge L Gamboa, MD Ph.D
Principal Investigator: Talat Alp Ikizler, MD

Sponsors and Collaborators

Vanderbilt University Medical Center

More Information

Responsible Party:	Jorge Gamboa, Research Assistant Professor, Vanderbilt University Medical Center
ClinicalTrials.gov Identifier:	NCT05297786 History of Changes
Other Study ID Numbers:	210633
First Posted:	March 28, 2022 Key Record Dates
Last Update Posted:	August 5, 2022
Last Verified:	August 2022

Individual Participant Data (IPD) Sharing Statement:
Plan to Share IPD: Yes

Studies a U.S. FDA-regulated Drug Product: Yes
Studies a U.S. FDA-regulated Device Product: No

Additional relevant MeSH terms:
Hypotension
Vascular Diseases
Cardiovascular Diseases

This is the classic website, which will be retired eventually. Please visit the modernized ClinicalTrials.gov instead.

Notice to API users: Read about imminent changes impacting usage of the ClinicalTrials.gov API.

U.S. National Library of Medicine
ClinicalTrials.gov

Find Studies About Studies Submit Studies Resources About Site PRS Login

Inhibition of Plasma Kallikrein as a New Therapy for Lung Injury

The safety and scientific validity of this study is the responsibility of the study sponsor and investigators. Listing a study does not mean it has been evaluated by the U.S. Federal Government. Know the risks and potential benefits of clinical studies and talk to your health care provider before participating. Read our disclaimer for details.

ClinicalTrials.gov Identifier: NCT04848272

Recruitment Status : Recruiting
First Posted : April 19, 2021
Last Update Posted : May 6, 2022

See Contacts and Locations

View this study on the modernized ClinicalTrials.gov

Sponsor:
St Vincent's Institute of Medical Research

Information provided by (Responsible Party):
Duncan Campbell, St Vincent's Institute of Medical Research

Study Details | Tabular View | No Results Posted | Disclaimer | How to Read a Study Record

Study Description

Go to

Brief Summary:
Phase 1 study investigating safety of **lanadelumab** administration to patients with lung injury

Condition or disease	Intervention/treatment	Phase
Lung Injury	Drug: **Lanadelumab**<br>Other: Saline control	Phase 1

Detailed Description:
This study will investigate the safety of inhibition of plasma kallikrein by lanadelumab administration in patients with lung injury.

Study Design

Go to

Study Type :	Interventional (Clinical Trial)
Estimated Enrollment :	48 participants
Allocation:	Non-Randomized
Intervention Model:	Sequential Assignment
Intervention Model Description:	Open-label placebo-controlled dose-escalation Phase 1 study
Masking:	None (Open Label)
Primary Purpose:	Treatment
Official Title:	Inhibition of Plasma Kallikrein as a New Therapy for Lung Injury
Actual Study Start Date :	August 9, 2021
Estimated Primary Completion Date :	December 2022
Estimated Study Completion Date :	June 2023

Arms and Interventions

Go to

Arm	Intervention/treatment
Placebo Comparator: Saline control<br>Saline control	Other: Saline control<br>Saline control
Experimental: **Lanadelumab** 30 mg<br>**Lanadelumab** 30 mg	Drug: **Lanadelumab**<br>Monoclonal antibody that targets active plasma kallikrein<br>Other Name: **Takhzyro**
Experimental: **Lanadelumab** 100 mg<br>**Lanadelumab** 100 mg	Drug: **Lanadelumab**<br>Monoclonal antibody that targets active plasma kallikrein<br>Other Name: **Takhzyro**
Experimental: **Lanadelumab** 300 mg<br>**Lanadelumab** 300 mg	Drug: **Lanadelumab**<br>Monoclonal antibody that targets active plasma kallikrein<br>Other Name: **Takhzyro**

Outcome Measures

Go to

Primary Outcome Measures :

1. Number of Participants With Treatment-Related Adverse Events [Time Frame: 28 days]

   Blood gases, pH, bicarbonate, carbon dioxide and lactic acid levels. If the participant is receiving mechanical ventilation, the ventilator settings will be constantly monitored.

   Chest X-ray, as indicated. Constant monitoring of haemodynamics (pulse rate, blood pressure) via an arterial cannula, use of vasoactive medications, and constant monitoring of ECG. Fluid balance, including urine output. Serum creatinine, electrolytes, liver function, creatinine kinase, FBE and coagulation will be measured daily or less frequently as decided by the treating team.

   If there is suspicion of infection, then appropriate samples will be taken for microbiological examination.

   Neurological function will be assessed several times per day.

Secondary Outcome Measures :

1. Blood bradykinin concentration [Time Frame: 0, 6, 12, and 24 hours]

   Blood bradykinin concentration

Eligibility Criteria

Go to

Information from the National Library of Medicine NIH NLM

Choosing to participate in a study is an important personal decision. Talk with your doctor and family members or friends about deciding to join a study. To learn more about this study, you or your doctor may contact the study research staff using the contacts provided below. For general information, Learn About Clinical Studies.

Ages Eligible for Study:	18 Years to 90 Years (Adult, Older Adult)
Sexes Eligible for Study:	All
Accepts Healthy Volunteers:	No

Criteria

Inclusion Criteria:

- Age >18 years admitted to the ICU with primary diagnosis of lung injury. The criteria for the diagnosis of lung injury include five principal elements: hypoxemia (blood oxygen saturation (PaO2) ≤92% when breathing ambient air, ratio of PaO2 to the fraction of inspired oxygen (FiO2) ≤300), diffuse pulmonary infiltrates on chest radiographs, decreased lung compliance, and the absence of congestive heart failure.
- Both males and females will be recruited in the proportion in which they are admitted to the ICU with lung injury.
- Arterial cannula in place as part of usual care for the measurement of blood gases.
- Patients may or may not be intubated and mechanically ventilated.
- Able to provide informed consent, or if unable to do so, a responsible person:

medical treatment decision maker is available (by telephone if necessary) who can be approached to seek consent.

Exclusion Criteria:

- Other causes of lung infiltrates: pulmonary oedema, alveolar haemorrhage, adverse drug reactions, radiation injury and the idiopathic pneumonitis syndrome.
- Significant dysfunction of non-pulmonary organs in the opinion of the treating ICU consultant.
- Death is deemed imminent or inevitable or there is underlying disease with a life expectancy of less than 90 days.
- Previously enrolled in this study.
- Enrolled in another study.
- Usually receives home oxygen.
- Usually receives any type of assisted ventilation at home. e.g. continuous positive airway pressure for obstructive sleep apnoea.
- Pregnant or might be pregnant. Women aged 18 to 49 are excluded unless there is documented menopause, hysterectomy or surgical sterilisation, or a pregnancy test is negative.
- Objection from the treating clinician.
- Consent refused by the patient or substitute decision maker.

Contacts and Locations

Go to

Information from the National Library of Medicine NIH NLM

To learn more about this study, you or your doctor may contact the study research staff using the contact information provided by the sponsor.

*Please refer to this study by its ClinicalTrials.gov identifier (NCT number): **NCT04848272***

Locations

Australia, Victoria

St Vincent's Hospital Melbourne	**Recruiting**
Melbourne, Victoria, Australia, 3065	
Contact: Duncan J Campbell, MB.BS., PhD 61 429959601 dcampbell@svi.edu.au	
Contact: Barry Dixon, MB.BS., PhD 61 439618815 barry.dixon@svha.org.au	

Sponsors and Collaborators

St Vincent's Institute of Medical Research

More Information

Go to

Responsible Party:	Duncan Campbell, Associate Professor, St Vincent's Institute of Medical Research
ClinicalTrials.gov Identifier:	NCT04848272 History of Changes
Other Study ID Numbers:	SVI-01
First Posted:	April 19, 2021 Key Record Dates
Last Update Posted:	May 6, 2022
Last Verified:	May 2022

Individual Participant Data (IPD) Sharing Statement:

Plan to Share IPD:	Yes
Plan Description:	Anonymised individual participant data (IPD) available to other researchers
Supporting Materials:	Study Protocol<br>Statistical Analysis Plan (SAP)<br>Informed Consent Form (ICF)<br>Clinical Study Report (CSR)<br>Analytic Code
Time Frame:	After completion of study
Access Criteria:	To be determined

Studies a U.S. FDA-regulated Drug Product:	No
Studies a U.S. FDA-regulated Device Product:	No
Product Manufactured in and Exported from the U.S.:	No

Additional relevant MeSH terms:

Lung Injury
Wounds and Injuries
Lung Diseases
Respiratory Tract Diseases
Thoracic Injuries

ePDS: 348

Oral plasma kallikrein inhibitor KV998052 improves arterial blood oxygenation in a murine model of acute respiratory distress syndrome (ARDS)

Nivetha Murugesan, Allen C. Clermont and Edward P. Feener
KalVista Pharmaceuticals, Inc., Cambridge, MA, USA

Background/Objective

- Acute respiratory distress syndrome (ARDS) is the acute onset of noncardiogenic pulmonary edema, hypoxemia and widespread inflammation in the lungs
- ARDS is a serious complication associated with the infection with SARS-CoV-2 virus that causes COVID-19
- Recent reports have implicated the kallikrein-kinin system in ARDS caused by SARS-CoV-2[1-4]
- Pharmacological inhibition of plasma kallikrein (PKa) may provide a therapeutic target for treating ARDS symptoms
- The aim of this study is to evaluate effects of two plasma kallikrein inhibitors, subcutaneously implanted KV999272 (14.4 mg/kg/day) and oral KV998052 (45 mg/kg bid) on an acid-aspiration lung injury mouse model of ARDS

Methods

- Tracheostomy was performed on anesthetized C57BL/6 mice and 0.1N HCl (pH 1.25) instilled to induce ARDS; control animals received saline (S).
- Bronchoalveolar lavage fluid (BALF) was collected via a blunt cannula inserted into the trachea
- Protein concentrations in the BALF were measured using a BCA protein assay
- Plasma prekallikrein (PKa) and high molecular weight kininogen (HK) were measured by immunoassay using the WES system (ProteinSimple)
- Arterial blood oxygenation (oxygen saturation, sO_2,%) was measured using a paw pulse oximeter (Kent Scientific)
- Contact system activation was triggered with dextran sulphate (DXS, Sigma, 6.25 μg/ml) in undiluted citrated mouse plasma in the absence or presence of PKa inhibitor KV998052

Results

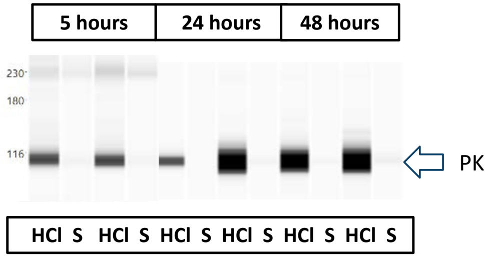

Acid instillation in mice increased PK levels in BALF at 5,24,48 hours.

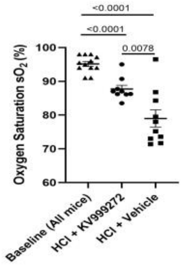

Systemic administration of KV999272 improves oxygen saturation in acid instilled mice compared with vehicle controls.

Results

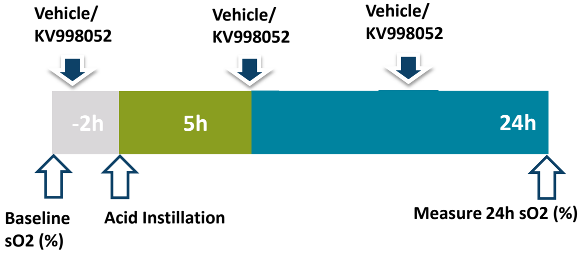

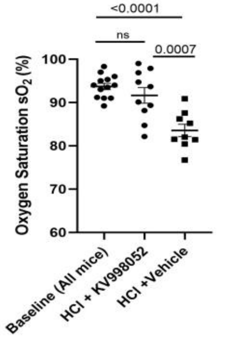

Pretreatment with orally administered KV998052 was associated with significantly improved blood oxygenation.

Results

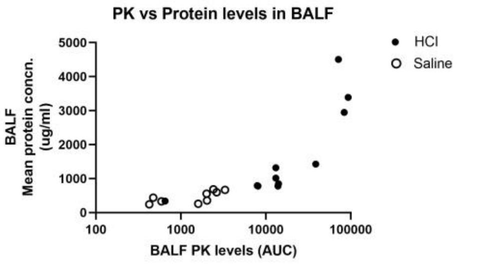

PK levels correlates with protein concentrations in BALF in acid instilled ARDS mice.

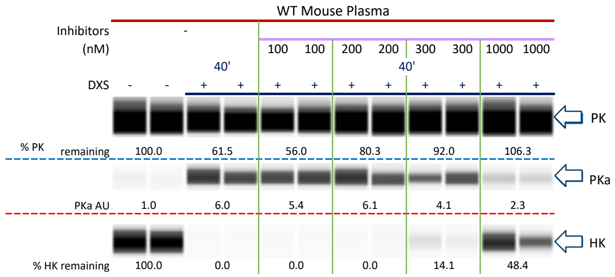

KV998052 protects against DXS stimulated PKa generation and HK cleavage in mouse plasma, in vitro.

Results

BALF Protein levels

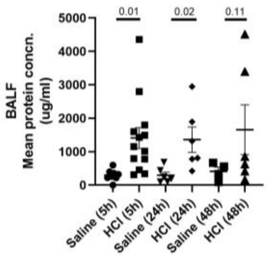

Oxygen Saturation

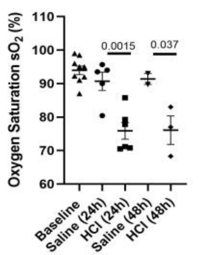

Acid-instillation increased BALF protein concentration and impaired blood oxygenation in mice.

Results

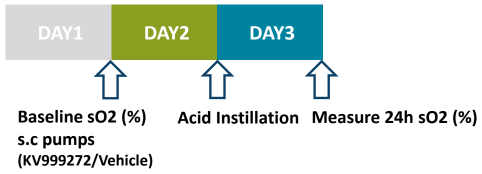

Experimental design for subcutaneous administration of KV999272 in acid instilled ARDS mice.

Summary and Conclusion

- ARDS in mice was associated with high levels of BALF protein concentration at 5h and 24h post surgery. This correlated with a significant reduction in sO_2 levels in acid instilled ARDS mice at both 24h and 48h
- PK levels were increased in the BALF fluid at 5h, 24h and 48h in HCl-instilled ARDS mice compared to saline-instilled controls. Increase in PK correlated with increased BALF protein concentration
- Systemic administration of a PKa inhibitor, KV999272, was associated with higher sO_2 in HCl instilled mice compared with vehicle treated mice
- KV998052, an orally available PKa inhibitor, protected against DXS stimulated HK cleavage in mouse plasma, *in vitro*
- Pretreatment of mice with orally administered KV998052 was associated with significantly improved blood oxygenation in mice with HCl induced ARDS compared with mice receiving vehicle
- Pharmacological inhibition of PKa may provide a therapeutic opportunity for the treatment of ARDS

References

1. de Maat S et al., Semin. Thromb. Hemost. 2020;46(7):835-7 2. van de Veerdonk et al., JAMA Network Open. 2020;3(8):e2017708; 3. Lipsey et al., Front. Immunol., 2021; 4. Mansour et al., Viruses, 2021;13(2):309.

Conflicts: Authors are or were employees of KalVista Pharmaceuticals during the execution of this work

Aprotinin Injection (marketed as Trasylol) Information

Share Tweet Linkedin Email Print

Postmarket Drug Safety Information for Patients and Providers

Index to Drug-Specific Information

This product is not currently available for purchase in the U.S.

Content current as of:
02/05/2018

[UPDATE 5/14/2008]: Following publication of the *Blood conservation using antifibrinolytics: A randomized trial in a cardiac surgery population* (BART) study in the May 14, 2008 online issue of *The New England Journal of Medicine*, Bayer Pharmaceuticals, the manufacturer of Trasylol (aprotinin), notified the FDA of their intent to remove all remaining supplies of Trasylol from hospital pharmacies and warehouses. [See FDA Statement]. Because Trasylol has been shown to decrease the need for red blood cell transfusions in patients undergoing coronary artery bypass surgery, future supplies of Trasylol will continue to be available through the company as an investigational drug under a special treatment protocol. In November 2007, Bayer suspended the marketing of this drug until final results of the BART study became available. The BART study showed an increase in the risk of death with Trasylol compared with aminocaproic acid and tranexamic acid, consistent with findings from other recent studies. FDA will be reviewing these data and will reassess the status and access to the product once the review is completed.

Historical Information

- Manufacturer Removes Remaining Stocks of Trasylol
- FDA Requests Marketing Suspension of Trasylol
- November 5, 2007 Transcript of FDA Press Conference on Trasylol
- November 5, 2007 Second Transcript of FDA Press Conference on Trasylol
- FDA Revises Labeling for Trasylol (Aprotinin Injection) to Strengthen Safety Warnings and Limit Usage of Drug to Specific Situations
- FDA Statement Regarding New Trasylol Data
- FDA Issues Public Health Advisory for Trasylol

Related Information

- Early Communication about an Ongoing Safety Review on Aprotinin Injection (marketed as Trasylol)
- Information for Healthcare Professionals Aprotinin Injection (marketed as Trasylol) text version
- Information for Healthcare Professionals Aprotinin (marketed as Trasylol) 9/2006 text version
- Aprotinin Injection (marketed as Trasylol)
- Aprotinin Injection (marketed as Trasylol) 5/2008
- Questions and Answers on Aprotinin (marketed as Trasylol)
- Regulatory History of Aprotinin from Drugs@FDA

This is the classic website, which will be retired eventually. Please visit the modernized ClinicalTrials.gov instead.

Phase II Study to Compare MDCO-2010 vs Placebo and Tranexamic Acid in Patients Undergoing Cardiac Surgery

The safety and scientific validity of this study is the responsibility of the study sponsor and investigators. Listing a study does not mean it has been evaluated by the U.S. Federal Government. Read our disclaimer for details.

ClinicalTrials.gov Identifier: NCT01530399

Recruitment Status : Terminated (Safety)
First Posted : February 9, 2012
Results First Posted : December 10, 2015
Last Update Posted : December 10, 2015

View this study on the modernized ClinicalTrials.gov

Sponsor:
The Medicines Company

Information provided by (Responsible Party):
The Medicines Company

Study Description

Brief Summary:
The objective of this study is to determine the dose response relationship regarding blood loss, PK, PD and clinical outcomes of **MDCO-2010** in comparison to placebo and tranexamic acid in patients undergoing primary cardiac surgery with cardiopulmonary bypass. The aim of the study is to define minimally effective dose of **MDCO-2010.**

Condition or disease	Intervention/treatment	Phase
Bleeding	Drug: MDCO 1<br>Drug: MDCO 2<br>Drug: MDCO 3<br>Drug: MDCO 4<br>Drug: Tranexamic Acid<br>Drug: Saline	Phase 2

Detailed Description:

This was a two-stage, double-blind, randomized, parallel-group, multicenter Phase II dose-selection study to compare antifibrinolytic MDCO-2010 vs tranexamic acid and placebo in reducing blood loss.

This study was designed to examine a broad range of doses to fully characterize the dose-response relationship between MDCO-2010 dose, plasma PK, PD, and clinical effects.

In Stage 1, 90 patients were to be enrolled into one of six treatment groups with 15 patients per group: four groups were to receive MDCO-2010, one group was to receive tranexamic acid, and one group was to receive placebo. Stage 2 was to be an expansion of Stage 1.

The study was terminated after 49 patients were enrolled into Stage 1.

Study Design

Study Type : Interventional (Clinical Trial)
Actual Enrollment : 44 participants
Allocation: Randomized
Intervention Model: Parallel Assignment
Masking: Triple (Participant, Investigator, Outcomes Assessor)
Primary Purpose: Treatment
Official Title: A Phase II, Double-blind, Parallel Group, Dose-selection Study to Compare Antifibrinolytic **MDCO-2010** vs. Placebo and Tranexamic Acid in Reducing Blood Loss in Patients Undergoing Primary Cardiac Surgery
Study Start Date : March 2012
Actual Primary Completion Date : November 2012
Actual Study Completion Date : November 2012

Resource links provided by the National Library of Medicine NIH NLM

MedlinePlus related topics: Heart Surgery

Drug Information available for: Tranexamic acid

U.S. FDA Resources

Arms and Interventions

Arm	Intervention/treatment
Experimental: MDCO 1<br>**MDCO-2010**: load 15 µg/kg; infusion 30 µg/kg/h; CPB prime 0.11 µg/mL priming volume	Drug: MDCO 1<br>**MDCO-2010** will be administered as a loading dose followed by a continuous infusion until sternal closure. In addition, the CPB reservoir will be primed with **MDCO-2010.** MDCO 1: load 15 µg/kg; infusion 30 µg/kg/h; CPB prime 0.11 µg/mL priming volume<br>Other Name: **MDCO-2010**
Experimental: MDCO 2<br>**MDCO-2010**: load 30 µg/kg; infusion 60 µg/kg/h; CPB prime 0.22 µg/mL priming volume	Drug: MDCO 2<br>**MDCO-2010** will be administered as a loading dose followed by a continuous infusion until sternal closure. In addition, the CPB reservoir will be primed with **MDCO-2010.** MDCO 2: load 30 µg/kg; infusion 60 µg/kg/h; CPB prime 0.22 µg/mL priming volume<br>Other Name: **MDCO-2010**
Experimental: MDCO 3<br>**MDCO-2010**: load 60 µg/kg ; infusion 120 µg/kg/h; CPB prime 0.44 µg/mL priming volume	Drug: MDCO 3<br>**MDCO-2010** will be administered as a loading dose followed by a continuous infusion until sternal closure. In addition, the CPB reservoir will be primed with **MDCO-2010.** MDCO 3: load 60 µg/kg ; infusion 120 µg/kg/h; CPB prime 0.44 µg/mL priming volume<br>Other Name: **MDCO-2010**
Experimental: MDCO 4<br>**MDCO-2010**: load 90 µg/kg; infusion 180 µg/kg/h; CPB prime 0.65 µg/mL priming volume	Drug: MDCO 4<br>**MDCO-2010** will be administered as a loading dose followed by a continuous infusion until sternal closure. In addition, the CPB reservoir will be primed with **MDCO-2010.** MDCO 4: load 90 µg/kg; infusion 180 µg/kg/h; CPB prime 0.65 µg/mL priming volume<br>Other Name: **MDCO-2010**
Placebo Comparator: Saline<br>Commercially available saline (0.9% NaCl solution)	Drug: Tranexamic Acid<br>Tranexamic acid will be administered as a loading dose followed by a continuous infusion until sternal closure. In addition, the CPB reservoir will be primed with tranexamic acid. The flow rates will be the same as for **MDCO-2010.**<br>Other Name: TXA
Active Comparator: Tranexamic acid<br>Tranexamic acid: 12 mg/kg loading dose; 5 mg/kg/h infusion; 0.556 mg/mL CPB priming	Drug: Saline<br>A loading dose of saline will be followed by a continuous infusion of saline until sternal closure. In addition, the CPB reservoir will be primed with saline. The flow rates will be the same as for **MDCO-2010.**<br>Other Name: NaCl 0.9%

Outcome Measures

Primary Outcome Measures :

1. Chest Tube Drainage at 12 Hours After Surgery [Time Frame: 12 hours post CABG]

Eligibility Criteria

Information from the National Library of Medicine NIH NLM

Choosing to participate in a study is an important personal decision. Talk with your doctor and family members or friends about deciding to join a study. To learn more about this study, you or your doctor may contact the study research staff using the contacts provided below. For general information, Learn About Clinical Studies.

Ages Eligible for Study: 18 Years to 85 Years (Adult, Older Adult)
Sexes Eligible for Study: All
Accepts Healthy Volunteers: No

Criteria

Inclusion Criteria:

- For Stage 1: Planned primary isolated CABG surgery
- For Stage 2: Planned primary isolated CABG surgery, OR Planned primary combined CABG and aortic valve replacement surgery
- Men, aged 18 to 85 years, or Women, aged 18 to 85 years, either of postmenopausal status, defined as ≥1 year since last menstrual period AND if less than 65 years old with a negative pre-operative pregnancy test within 24 hours of surgery OR with medical history of hysterectomy or bilateral oophorectomy
- Written informed consent

Exclusion Criteria:

- Off-pump surgery or hybrid procedures
- Patients undergoing repeat sternotomy
- Planned deep hypothermic circulatory arrest (<28°C)
- Known allergy, sensitivity, or contraindications to tranexamic acid
- Epileptiform disorders, history of seizure activity, or anticonvulsive medication
- Administration of clopidogrel, ticagrelor or ticlopidine within 5 days prior to surgery or prasugrel within 7 days prior to surgery.
- Administration of low molecular weight heparin, glycoprotein IIb/IIIa inhibitors, or fondaparinux within 12 hours prior to surgery
- Known history of coronary stent thrombosis within the last three months
- History of stroke or transient ischemic attack within 3 months prior to surgery
- History of deep venous thrombosis or pulmonary embolism
- LVEF ≤35% or Grade III or IV
- Body mass index <20 or >35
- Known active gastrointestinal (GI) or other non-catheterization bleeding within 7 days prior to surgery
- Preoperative coagulation abnormalities defined as:
- Platelet count <100,000/L or >350,000/L, or
- International normalized ration (INR) >1.5, or
- Hematocrit <36%, or
- aPTT >1.5 x ULN
- Major surgical procedures within 30 days prior to surgery
- Patient inability or refusal to receive donor blood products if necessary
- Creatinine >2 mg/dL or estimated glomerular filtration rate (eGFR) (calculated using Modification of Diet in Renal Disease [MDRD] equation <30 mL/min)
- Known heparin-induced thrombocytopenia type II
- Known history of thrombophilia, such as AT-III, Protein C or Protein S deficiency, Factor V Leiden, anti-phospholipid syndrome
- Active liver disease defined as any known current infectious, neoplastic or metabolic pathology of the liver OR ALT or AST elevation >2x ULN or total bilirubin elevation >1.5x ULN at Screening
- Any condition requiring ongoing chronic immunosuppressive medication
- Malignancy within 5 years prior to surgery
- Receipt of an investigational drug or device within 60 days prior to surgery Any other condition which, in the opinion of the Principal Investigator, would put the subject at increased risk from participating in the study or otherwise prevent a patient"s participation in the study

Contacts and Locations

Information from the National Library of Medicine NIH NLM

To learn more about this study, you or your doctor may contact the study research staff using the contact information provided by the sponsor.

Please refer to this study by its ClinicalTrials.gov identifier (NCT number): ***NCT01530399***

Locations

Germany
Charité Berlin
Berlin, Germany
Universitätsklinikum Bonn
Bonn, Germany
Universität Heidelberg
Heidelberg, Germany
Universität Leipzig - Herzzentrum
Leipzig, Germany

Switzerland
University Hospital/Inselspital Bern
Bern, Switzerland

Sponsors and Collaborators
The Medicines Company

Investigators
Principal Investigator: Lars Englberger, PD DR. med University Hospital Inselspital, Bern

More Information

Responsible Party: The Medicines Company
ClinicalTrials.gov Identifier: NCT01530399 History of Changes
Other Study ID Numbers: TMC-MDC-11-01
First Posted: February 9, 2012 Key Record Dates
Results First Posted: December 10, 2015
Last Update Posted: December 10, 2015
Last Verified: November 2015

Additional relevant MeSH terms:

Tranexamic Acid	Molecular Mechanisms of Pharmacological Action
Antifibrinolytic Agents	Hemostatics
Fibrin Modulating Agents	Coagulants

This is the classic website, which will be retired eventually. Please visit the modernized ClinicalTrials.gov instead.

Phase II Study to Compare MDCO-2010 vs Placebo and Tranexamic Acid in Patients Undergoing Cardiac Surgery

The safety and scientific validity of this study is the responsibility of the study sponsor and investigators. Listing a study does not mean it has been evaluated by the U.S. Federal Government. Read our disclaimer for details.

ClinicalTrials.gov Identifier: NCT01530399

Recruitment Status : Terminated (Safety)
First Posted : February 9, 2012
Results First Posted : December 10, 2015
Last Update Posted : December 10, 2015

View this study on the modernized ClinicalTrials.gov

Sponsor:
The Medicines Company

Information provided by (Responsible Party):
The Medicines Company

**Study Type**	Interventional
**Study Design**	Allocation: Randomized; Intervention Model: Parallel Assignment; Masking: Triple (Participant, Investigator, Outcomes Assessor); Primary Purpose: Treatment
**Condition**	Bleeding
**Interventions**	Drug: MDCO 1<br>Drug: MDCO 2<br>Drug: MDCO 3<br>Drug: MDCO 4<br>Drug: Tranexamic Acid<br>Drug: Saline
**Enrollment**	44

Participant Flow

Recruitment Details	
Pre-assignment Details	

Arm/Group Title	MDCO 1	MDCO 2	MDCO 3	MDCO 4	Saline	Tranexamic Acid
Arm/Group Description	MDCO-2010 Dose 1: MDCO-2010 will be administered as a loading dose followed by a continuous infusion until sternal closure. In addition, the CPB reservoir will be primed with MDCO-2010. MDCO 1: load 15 µg/kg; infusion 30 µg/kg/h; CPB prime 0.11 µg/mL priming volume	MDCO-2010 Dose 2: MDCO-2010 will be administered as a loading dose followed by a continuous infusion until sternal closure. In addition, the CPB reservoir will be primed with MDCO-2010. MDCO 2: load 30 µg/kg; infusion 60 µg/kg/h; CPB prime 0.22 µg/mL priming volume	MDCO-2010 Dose 3: MDCO-2010 will be administered as a loading dose followed by a continuous infusion until sternal closure. In addition, the CPB reservoir will be primed with MDCO-2010. MDCO 3: load 60 µg/kg ; infusion 120 µg/kg/h; CPB prime 0.44 µg/mL priming volume	MDCO-2010 Dose 4: MDCO-2010 will be administered as a loading dose followed by a continuous infusion until sternal closure. In addition, the CPB reservoir will be primed with MDCO-2010. MDCO 4: load 90 µg/kg; infusion 180 µg/kg/h; CPB prime 0.65 µg/mL priming volume	Tranexamic Acid: Tranexamic acid will be administered as a loading dose followed by a continuous infusion until sternal closure. In addition, the CPB reservoir will be primed with tranexamic acid. The flow rates will be the same as for MDCO-2010.	saline: A loading dose of saline will be followed by a continuous infusion of saline until sternal closure. In addition, the CPB reservoir will be primed with saline. The flow rates will be the same as for MDCO-2010.
Period Title: **Overall Study**						
Started	8	10	8	10	6	7
Completed	7	8	7	10	6	6
Not Completed	1	2	1	0	0	1

Baseline Characteristics

Arm/Group Title		MDCO 1	MDCO 2	MDCO 3	MDCO 4	Saline	Tranexamic Acid	Total
Arm/Group Description		MDCO-2010 Dose 1: MDCO-2010 will be administered as a loading dose followed by a continuous infusion until sternal closure. In addition, the CPB reservoir will be primed with MDCO-2010. MDCO 1: load 15 µg/kg; infusion 30 µg/kg/h; CPB prime 0.11 µg/mL priming volume	MDCO-2010 Dose 2: MDCO-2010 will be administered as a loading dose followed by a continuous infusion until sternal closure. In addition, the CPB reservoir will be primed with MDCO-2010. MDCO 2: load 30 µg/kg; infusion 60 µg/kg/h; CPB prime 0.22 µg/mL priming volume	MDCO-2010 Dose 3: MDCO-2010 will be administered as a loading dose followed by a continuous infusion until sternal closure. In addition, the CPB reservoir will be primed with MDCO-2010. MDCO 3: load 60 µg/kg ; infusion 120 µg/kg/h; CPB prime 0.44 µg/mL priming volume	MDCO-2010 Dose 4: MDCO-2010 will be administered as a loading dose followed by a continuous infusion until sternal closure. In addition, the CPB reservoir will be primed with MDCO-2010. MDCO 4: load 90 µg/kg; infusion 180 µg/kg/h; CPB prime 0.65 µg/mL priming volume	Tranexamic Acid: Tranexamic acid will be administered as a loading dose followed by a continuous infusion until sternal closure. In addition, the CPB reservoir will be primed with tranexamic acid. The flow rates will be the same as for MDCO-2010.	saline: A loading dose of saline will be followed by a continuous infusion of saline until sternal closure. In addition, the CPB reservoir will be primed with saline. The flow rates will be the same as for MDCO-2010.	Total of all reporting groups
Overall Number of Baseline Participants		8	10	8	10	6	7	49
Baseline Analysis Population Description		[Not Specified]						
Age, Continuous<br>Median (Inter-Quartile Range)<br>Unit of measure: Years								
	Number Analyzed	8 participants	10 participants	8 participants	10 participants	6 participants	7 participants	49 participants
		71<br>(64 to 75)	70<br>(57 to 73)	66<br>(60 to 79)	73<br>(68 to 73)	66<br>(60 to 71)	58<br>(52 to 62)	69<br>(61 to 73)
Sex: Female, Male<br>Measure Type: Count of Participants<br>Unit of measure: Participants								
	Number Analyzed	8 participants	10 participants	8 participants	10 participants	6 participants	7 participants	49 participants
	Female	1 12.5%	2 20.0%	1 12.5%	2 20.0%	2 33.3%	0 0.0%	8 16.3%
	Male	7 87.5%	8 80.0%	7 87.5%	8 80.0%	4 66.7%	7 100.0%	41 83.7%
Region of Enrollment<br>Measure Type: Number<br>Unit of measure: Participants	Number Analyzed	8 participants	10 participants	8 participants	10 participants	6 participants	7 participants	49 participants
Germany		6	7	5	7	4	4	33
Switzerland		2	3	3	3	2	3	16

Outcome Measures

1. Primary Outcome

Title	Chest Tube Drainage at 12 Hours After Surgery
Description	[Not Specified]
Time Frame	12 hours post CABG

Outcome Measure Data

Analysis Population Description
[Not Specified]

Arm/Group Title	MDCO 1	MDCO 2	MDCO 3	MDCO 4	Saline	Tranexamic Acid
Arm/Group Description:	MDCO-2010 Dose 1: MDCO-2010 will be administered as a loading dose followed by a continuous infusion until sternal closure. In addition, the CPB reservoir will be primed with MDCO-2010. MDCO 1: load 15 µg/kg; infusion 30 µg/kg/h; CPB prime 0.11 µg/mL priming volume	MDCO-2010 Dose 2: MDCO-2010 will be administered as a loading dose followed by a continuous infusion until sternal closure. In addition, the CPB reservoir will be primed with MDCO-2010. MDCO 2: load 30 µg/kg; infusion 60 µg/kg/h; CPB prime 0.22 µg/mL priming volume	MDCO-2010 Dose 3: MDCO-2010 will be administered as a loading dose followed by a continuous infusion until sternal closure. In addition, the CPB reservoir will be primed with MDCO-2010. MDCO 3: load 60 µg/kg ; infusion 120 µg/kg/h; CPB prime 0.44 µg/mL priming volume	MDCO-2010 Dose 4: MDCO-2010 will be administered as a loading dose followed by a continuous infusion until sternal closure. In addition, the CPB reservoir will be primed with MDCO-2010. MDCO 4: load 90 µg/kg; infusion 180 µg/kg/h; CPB prime 0.65 µg/mL priming volume	Tranexamic Acid: Tranexamic acid will be administered as a loading dose followed by a continuous infusion until sternal closure. In addition, the CPB reservoir will be primed with tranexamic acid. The flow rates will be the same as for MDCO-2010.	saline: A loading dose of saline will be followed by a continuous infusion of saline until sternal closure. In addition, the CPB reservoir will be primed with saline. The flow rates will be the same as for MDCO-2010.
Overall Number of Participants Analyzed	7	7	6	10	6	6
Median (Inter-Quartile Range)<br>Unit of Measure: mL						
	600<br>(300 to 970)	580<br>(420 to 880)	480<br>(350 to 700)	453<br>(415 to 645)	645<br>(400 to 1040)	593<br>(530 to 750)

Adverse Events

Time Frame	30 (+5) days post treatment
Adverse Event Reporting Description	[Not Specified]

Arm/Group Title	MDCO 1	MDCO 2	MDCO 3	MDCO 4	Saline	Tranexamic Acid
Arm/Group Description	MDCO-2010 Dose 1: MDCO-2010 will be administered as a loading dose followed by a continuous infusion until sternal closure. In addition, the CPB reservoir will be primed with MDCO-2010. MDCO 1: load 15 µg/kg; infusion 30 µg/kg/h; CPB prime 0.11 µg/mL priming volume	MDCO-2010 Dose 2: MDCO-2010 will be administered as a loading dose followed by a continuous infusion until sternal closure. In addition, the CPB reservoir will be primed with MDCO-2010. MDCO 2: load 30 µg/kg; infusion 60 µg/kg/h; CPB prime 0.22 µg/mL priming volume	MDCO-2010 Dose 3: MDCO-2010 will be administered as a loading dose followed by a continuous infusion until sternal closure. In addition, the CPB reservoir will be primed with MDCO-2010. MDCO 3: load 60 µg/kg ; infusion 120 µg/kg/h; CPB prime 0.44 µg/mL priming volume	MDCO-2010 Dose 4: MDCO-2010 will be administered as a loading dose followed by a continuous infusion until sternal closure. In addition, the CPB reservoir will be primed with MDCO-2010. MDCO 4: load 90 µg/kg; infusion 180 µg/kg/h; CPB prime 0.65 µg/mL priming volume	Tranexamic Acid: Tranexamic acid will be administered as a loading dose followed by a continuous infusion until sternal closure. In addition, the CPB reservoir will be primed with tranexamic acid. The flow rates will be the same as for MDCO-2010.	saline: A loading dose of saline will be followed by a continuous infusion of saline until sternal closure. In addition, the CPB reservoir will be primed with saline. The flow rates will be the same as for MDCO-2010.

All-Cause Mortality

	MDCO 1	MDCO 2	MDCO 3	MDCO 4	Saline	Tranexamic Acid
	Affected / at Risk (%)	Affected / at Risk (%)	Affected / at Risk (%)	Affected / at Risk (%)	Affected / at Risk (%)	Affected / at Risk (%)
Total	--/--	--/--	--/--	--/--	--/--	--/--

Serious Adverse Events

	MDCO 1	MDCO 2	MDCO 3	MDCO 4	Saline	Tranexamic Acid
	Affected / at Risk (%)	Affected / at Risk (%)	Affected / at Risk (%)	Affected / at Risk (%)	Affected / at Risk (%)	Affected / at Risk (%)
Total	2/7 (28.57%)	2/8 (25.00%)	1/7 (14.29%)	2/10 (20.00%)	0/6 (0.00%)	0/6 (0.00%)
Blood and lymphatic system disorders						
Haemorrhagic anaemia †1	1/7 (14.29%)	0/8 (0.00%)	0/7 (0.00%)	0/10 (0.00%)	0/6 (0.00%)	0/6 (0.00%)
Cardiac disorders						
Cardiac tamponade †1	0/7 (0.00%)	1/8 (12.50%)	0/7 (0.00%)	0/10 (0.00%)	0/6 (0.00%)	0/6 (0.00%)
Coronary artery occlusion †1	0/7 (0.00%)	1/8 (12.50%)	0/7 (0.00%)	0/10 (0.00%)	0/6 (0.00%)	0/6 (0.00%)
Low cardiac output syndrome †1	0/7 (0.00%)	1/8 (12.50%)	1/7 (14.29%)	0/10 (0.00%)	0/6 (0.00%)	0/6 (0.00%)
Injury, poisoning and procedural complications						
Vascular graft thrombosis †1	0/7 (0.00%)	0/8 (0.00%)	0/7 (0.00%)	1/10 (10.00%)	0/6 (0.00%)	0/6 (0.00%)
Nervous system disorders						
Ischaemic stroke †1	1/7 (14.29%)	0/8 (0.00%)	0/7 (0.00%)	0/10 (0.00%)	0/6 (0.00%)	0/6 (0.00%)
Renal and urinary disorders						
Renal failure †1	1/7 (14.29%)	0/8 (0.00%)	0/7 (0.00%)	0/10 (0.00%)	0/6 (0.00%)	0/6 (0.00%)
Respiratory, thoracic and mediastinal disorders						
Respiratory failure †1	1/7 (14.29%)	0/8 (0.00%)	0/7 (0.00%)	0/10 (0.00%)	0/6 (0.00%)	0/6 (0.00%)
Vascular disorders						
Arterial thrombosis limb †1	0/7 (0.00%)	0/8 (0.00%)	0/7 (0.00%)	1/10 (10.00%)	0/6 (0.00%)	0/6 (0.00%)
Extremity necrosis †1	1/7 (14.29%)	0/8 (0.00%)	0/7 (0.00%)	0/10 (0.00%)	0/6 (0.00%)	0/6 (0.00%)
Jugular vein thrombosis †1	0/7 (0.00%)	0/8 (0.00%)	0/7 (0.00%)	1/10 (10.00%)	0/6 (0.00%)	0/6 (0.00%)

† Indicates events were collected by systematic assessment
1 Term from vocabulary, MedDRA (Unspecified)

Other (Not Including Serious) Adverse Events

Frequency Threshold for Reporting Other Adverse Events	5%

	MDCO 1	MDCO 2	MDCO 3	MDCO 4	Saline	Tranexamic Acid
	Affected / at Risk (%)	Affected / at Risk (%)	Affected / at Risk (%)	Affected / at Risk (%)	Affected / at Risk (%)	Affected / at Risk (%)
Total	5/7 (71.43%)	7/8 (87.50%)	4/7 (57.14%)	7/10 (70.00%)	5/6 (83.33%)	4/6 (66.67%)
Blood and lymphatic system disorders						
Troponin T increased †1	1/7 (14.29%)	2/8 (25.00%)	1/7 (14.29%)	1/10 (10.00%)	0/6 (0.00%)	0/6 (0.00%)
Cardiac disorders						
Atrial fibrillation †1	1/7 (14.29%)	2/8 (25.00%)	1/7 (14.29%)	2/10 (20.00%)	1/6 (16.67%)	2/6 (33.33%)
Gastrointestinal disorders						
Nausea †1	2/7 (28.57%)	1/8 (12.50%)	0/7 (0.00%)	1/10 (10.00%)	2/6 (33.33%)	0/6 (0.00%)
AST increased †1	0/7 (0.00%)	0/8 (0.00%)	0/7 (0.00%)	0/10 (0.00%)	0/6 (0.00%)	2/6 (33.33%)
Hepatobiliary disorders						
AST increased †1	0/7 (0.00%)	2/8 (25.00%)	0/7 (0.00%)	0/10 (0.00%)	0/6 (0.00%)	0/6 (0.00%)
Renal and urinary disorders						
Urinary tract infection †1	0/7 (0.00%)	0/8 (0.00%)	1/7 (14.29%)	2/10 (20.00%)	2/6 (33.33%)	0/6 (0.00%)
Respiratory, thoracic and mediastinal disorders						
Pleural effusion †1	3/7 (42.86%)	2/8 (25.00%)	1/7 (14.29%)	1/10 (10.00%)	1/6 (16.67%)	0/6 (0.00%)
Pneumothorax †1	0/7 (0.00%)	2/8 (25.00%)	1/7 (14.29%)	0/10 (0.00%)	0/6 (0.00%)	0/6 (0.00%)

† Indicates events were collected by systematic assessment
1 Term from vocabulary, MedDRA (Unspecified)

Limitations and Caveats

[Not Specified]

More Information

Certain Agreements

Principal Investigators are NOT employed by the organization sponsoring the study.

There IS an agreement between Principal Investigators and the Sponsor (or its agents) that restricts the PI's rights to discuss or publish trial results after the trial is completed.

After initial multicenter results communications are published, or after 12 months from study closure (whichever occurs first), sponsor can review results communications prior to submission and can embargo submissions for a period of 45 days from the time submitted to the sponsor for review, agreeing to resolve differences of opinion or interpretation through scientific debate. Sponsor can request further delay for an additional 90 days to file any patent applications if deemed necessary

Results Point of Contact

Name/Title: Peter Villiger
Organization: TMC
Phone: +19732906340
EMail: peter.villiger@themedco.com

Responsible Party: The Medicines Company
ClinicalTrials.gov Identifier: NCT01530399 History of Changes
Other Study ID Numbers: TMC-MDC-11-01
First Submitted: February 7, 2012
First Posted: February 9, 2012
Results First Submitted: November 4, 2015
Results First Posted: December 10, 2015
Last Update Posted: December 10, 2015

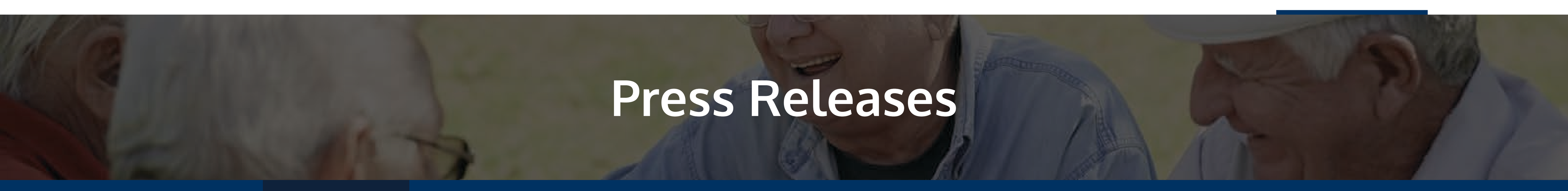

Press Releases

DiaMedica Therapeutics Announces Clinical Hold of its Phase 2/3 ReMEDy2 Clinical Trial for DM199

July 06, 2022 4:41pm EDT

Download as PDF

MINNEAPOLIS--(BUSINESS WIRE)-- DiaMedica Therapeutics Inc. (Nasdaq: DMAC), a clinical-stage biopharmaceutical company focused on developing novel treatments for neurological disorders and kidney diseases, today announced that the U.S. Food and Drug Administration (FDA) has placed a clinical hold on the Company's Phase 2/3 ReMEDy2 trial studying the use of the Company's product candidate, DM199, to treat acute ischemic stroke (AIS) patients. The clinical hold was initiated following the Company's pause in patient enrollment and submission of three serious adverse event reports to the FDA related to clinically significant, transient hypotension (low blood pressure) occurring shortly after initiation of the intravenous (IV) dose of DM199. The blood pressure levels of the three patients recovered back to their baseline blood pressure within minutes after IV infusion was stopped.

The Company believes that the adverse events resulted from switching to an IV bag formulated from different materials in the ReMEDy2 trial compared to the IV bag used in the prior Phase 2 ReMEDy1 trial. Due to supply issues, the type of IV bag used in the ReMEDy1 trial was not available in many U.S. hospitals, and accordingly after routine compatibility testing, a different type of IV bag was selected for use in the ReMEDy2 trial. As part of the Company's evaluation of the events that lead to these hypotensive events, the Company is confirming the differences in drug absorption in the IV bags used in the ReMEDy1 trial compared to the ReMEDy2 trial and plans to work with the FDA to modify the ReMEDy2 trial protocol to adjust the DM199 IV dosing to more closely match the dosing in the ReMEDy1 trial, taking into account these differences. The Company notes that no such hypotension issues were reported in its ReMEDy1 trial in which 46 stroke patients received DM199.

The Company may not enroll any additional patients in the ReMEDy2 trial until the Company provides the FDA with the Company's analysis of the events leading to or causing the hypotension, its suggested protocol modifications to address the mitigation of these events, its rationale and supporting data for the protocol modifications, and the FDA notifies the Company that it may resume enrollment in the clinical trial. Based on information received to date, DiaMedica believes that proportionate reductions in the DM199 dose level and IV infusion times will effectively mitigate the hypotension issue in ReMEDy2 patients. The Company plans to submit a revised ReMEDy2 trial protocol with the supporting rationale and data to the FDA for review upon completion of its compatibility analysis.

Kirsten Gruis, M.D., Chief Medical Officer of DiaMedica, noted that, "Patient safety is very important as we plan and conduct our clinical studies. Patient blood pressure is easily and routinely monitored in stroke patients which is why our study sites were able to quickly identify the issue and immediately stop the dosing of DM199, after which the patients then recovered within minutes and suffered no injuries. We are committed to working diligently with the FDA to resolve this issue and resume the trial as soon as reasonably practicable."

"While having to pause enrollment in the ReMEDy2 trial was not desirable, we remain confident about the future potential of DM199 and are committed to refining the dosing procedures and methods that will further enhance patient safety," commented Rick Pauls, DiaMedica's President and Chief Executive Officer. "We also take comfort in that the transient hypotension observed is yet another clinical indicator that DM199 may be biologically active in stroke patients and may provide a meaningful improvement in stroke outcomes."

About ReMEDy2 Trial

The ReMEDy2 trial is an adaptive design, randomized, double-blind, placebo-controlled trial studying the use of the Company's product candidate, DM199, to treat AIS patients. The trial is intended to enroll approximately 350 patients at 75 sites in the United States. Patients enrolled in the trial will be treated for three weeks with either DM199 or placebo, beginning within 24 hours of the onset of AIS symptoms, with the final follow-up at 90 days. The trial excludes patients treated with tissue plasminogen activator (tPA) and/or mechanical thrombectomy. The study population is representative of the approximately 80% of AIS patients who do not have treatment options today, primarily due to the limitations on treatment with tPA or mechanical thrombectomy. DiaMedica believes that the proposed trial has the potential to serve as a pivotal registration study of DM199 in this patient population.

The ReMEDy2 trial has two separate, independent, primary endpoints based upon both the results observed in the first ReMEDy1 phase 2 trial and published results from the urine-derived form of KLK1 used to successfully treat AIS in China. ReMEDy2 is powered for success with either endpoint: 1) physical recovery from stroke as measured by the well-established modified Rankin Scale (mRS) at day 90, and 2) the rate of ischemic stroke recurrence through day 90. Recurrent strokes represent 25% of all ischemic strokes, often occurring in the first few weeks after an initial stroke and are typically more disabling, costly, and fatal than initial strokes.

About DM199

DM199 is a recombinant (synthetic) form of human tissue kallikrein-1 (KLK1). KLK1 is a serine protease (protein) that plays an important role in the regulation of diverse physiological processes including blood flow, blood pressure, inflammation, fibrosis, oxidative stress and neurogenesis via a molecular mechanism that increases production of nitric oxide and prostaglandin. KLK1 deficiency may play a role in multiple vascular and fibrotic diseases such as stroke, chronic kidney disease, retinopathy, vascular dementia, and resistant hypertension where current treatment options are limited or ineffective. DiaMedica is the first company to have developed a recombinant form of the KLK1 protein. KLK1 protein, produced from porcine pancreas and human urine, has been used to treat patients in Japan, China and South Korea for decades. In September 2021, the FDA granted Fast Track Designation to DM199 for the treatment of acute ischemic stroke.

About DiaMedica Therapeutics Inc.

DiaMedica Therapeutics Inc. is a clinical stage biopharmaceutical company committed to improving the lives of people suffering serious diseases. Its lead candidate, DM199, is the first pharmaceutically active recombinant (synthetic) form of the KLK1 protein, an established therapeutic modality for the treatment of acute ischemic stroke and chronic kidney disease. For more information visit the Company's website at www.diamedica.com.

Cautionary Note Regarding Forward-Looking Statements

This press release contains forward-looking statements within the meaning of the U.S. Private Securities Litigation Reform Act of 1995 and forward-looking information that are based on the beliefs of management and reflect management's current expectations. When used in this press release, the words "estimate," "believe," "anticipate," "intend," "expect," "plan," "continue," "potential," "will," "may" or "should," the negative of these words or such variations thereon or comparable terminology and the use of future dates are intended to identify forward-looking statements and information. The forward-looking statements and information in this press release include statements regarding the Company's beliefs as to the issues raised in the FDA's clinical hold letter, and the causes of and its plans and ability to address the deficiencies identified in the clinical hold letter, the timeframe for doing so and future changes to the ReMEDy2 trial protocol; the anticipated clinical benefits and success of DM199, the timing and requirements of its clinical programs, including its Phase 2/3 trial for DM199 in patients with AIS, which DiaMedica believes has the potential to serve as a pivotal registration study of DM199 in that patient population; the potential for each of the two separate independent primary endpoints to be the basis for regulatory approval of DM199 for the treatment of AIS; anticipated clinical results and ability to achieve clinical and other milestones; and its goal of offering a treatment option for patients who suffer from AIS. Such statements and information reflect management's current view and DiaMedica undertakes no obligation to update or revise any of these statements or information. By their nature, forward-looking statements involve known and unknown risks, uncertainties and other factors which may cause actual results, performance or achievements, or other future events, to be materially different from any future results, performance or achievements expressed or implied by such forward-looking statements. Applicable risks and uncertainties include, among others, the risk that the Company may not be able to address successfully the concerns identified in the clinical hold letter or may require the Company to collect additional data or information beyond what it currently expects; the risk that the Company may not be able to lift the clinical hold or do so in a timely manner; uncertainties relating to regulatory applications and related filing and approval timelines, including the risk that FDA may not remove the clinical hold on the ReMEDy2 trial; the possibility of additional future adverse events associated with or unfavorable results from the ReMEDy2 trial; the possibility of unfavorable results from DiaMedica's clinical trials of DM199; the risk that existing preclinical and clinical data may not be predictive of the results of ongoing or later clinical trials; DiaMedica's plans to develop, obtain regulatory approval for and commercialize its DM199 product candidate for the treatment of AIS and chronic kidney disease and its expectations regarding the benefits of DM199; DiaMedica's ability to conduct successful clinical testing of DM199 and within its anticipated parameters, enrollment numbers, costs and timeframes; the adaptive design of the ReMEDy2 trial and the possibility that the targeted enrollment and other aspects of the trial could change depending upon certain factors, including additional input from the FDA and the blinded interim analysis; the perceived benefits of DM199 over existing treatment options; the potential direct or indirect impact of the COVID-19 pandemic, hospital and medical facility staffing shortages, and worldwide global supply chain shortages on DiaMedica's business and clinical trials, including its ability to meet its site activation and enrollment goals; DiaMedica's reliance on collaboration with third parties to conduct clinical trials; DiaMedica's ability to continue to obtain funding for its operations, including funding necessary to complete planned clinical trials and obtain regulatory approvals for DM199 for AIS and chronic kidney disease, and the risks identified under the heading "Risk Factors" in DiaMedica's annual report on Form 10-K for the fiscal year ended December 31, 2021 and subsequent U.S. Securities and Exchange Commission filings. The forward-looking information contained in this press release represents the expectations of DiaMedica as of the date of this press release and, accordingly, is subject to change after such date. Readers should not place undue importance on forward-looking information and should not rely upon this information as of any other date. While DiaMedica may elect to, it does not undertake to update this information at any particular time except as required in accordance with applicable laws.

View source version on businesswire.com:
https://www.businesswire.com/news/home/20220706005843/en/

Scott Kellen
Chief Financial Officer
Phone: (763) 496-5118
skellen@diamedica.com

Paul Papi
Corporate Communications
Phone: (508) 444-6790
ppapi@diamedica.com

Source: DiaMedica Therapeutics

Released July 6, 2022

© 2023 DiaMedica Therapeutics, Inc. All Rights Reserved.

DiaMedica Therapeutics Announces that the FDA Has Removed The Clinical Hold On DM199 Phase 2/3 Trial For Ischemic Stroke

June 21, 2023 8:15am EDT

Download as PDF

MINNEAPOLIS--(BUSINESS WIRE)-- DiaMedica Therapeutics Inc. (Nasdaq: DMAC), a clinical-stage biopharmaceutical company focused on developing novel treatments for neurological disorders and kidney diseases, announced today that the U.S. Food and Drug Administration (FDA) has removed the clinical hold placed on the investigational new drug application for its ReMEDy2 phase 2/3 clinical trial studying DM199 in the treatment of acute ischemic stroke (AIS) and that preparations are underway to resume the ReMEDy2 trial as soon as possible.

"We are pleased that the FDA has fully lifted the clinical hold. DM199, a synthetic formulation of the human tissue-1 kallikrein protein (KLK1), represents a novel approach to treating AIS patients, principally aimed at enhancing collateral blood flow in the brain tissues affected by the stroke," said Rick Pauls, DiaMedica's President and CEO.

"We look forward to re-engaging with our study sites and stroke expert principal investigators as we resume our ReMEDy2 trial as there is continued unmet need of new potential therapeutics for patients who have had an ischemic stroke," said Kirsten Gruis, MD, DiaMedica's Chief Medical Officer.

About the ReMEDy2 Trial

The ReMEDy2 trial is an adaptive design, randomized, double-blind, placebo-controlled trial studying the use of the Company's product candidate, DM199, to treat acute ischemic stroke (AIS) patients. The trial is intended to enroll approximately 350 patients at 75 sites in the United States. Patients enrolled in the trial will be treated for three weeks with either DM199 or placebo, beginning within 24 hours of the onset of AIS symptoms, with the final follow-up at 90 days. The trial excludes patients treated with tissue plasminogen activator (tPA) and/or mechanical thrombectomy. The study population is representative of the approximately 80% of AIS patients who do not have treatment options today, primarily due to the limitations on treatment with tPA or mechanical thrombectomy. DiaMedica believes that the proposed trial has the potential to serve as a pivotal registration study of DM199 in this patient population.

About DM199

DM199 is a recombinant (synthetic) form of human tissue kallikrein-1 (KLK1). KLK1 is a serine protease (protein) that plays an important role in the regulation of diverse physiological processes including blood flow, inflammation, fibrosis, oxidative stress and neurogenesis via a molecular mechanism that increases production of nitric oxide and prostaglandin. KLK1 deficiency may play a role in multiple vascular and fibrotic diseases such as stroke, chronic kidney disease, retinopathy, vascular dementia, and resistant hypertension where current treatment options are limited or ineffective. DiaMedica is the first company to have developed and clinically studied a recombinant form of the KLK1 protein. The KLK1 protein, produced from the pancreas of pigs and human urine, has been used to treat patients in Japan, China and South Korea for decades. DM199 is currently being studied in patients with acute ischemic stroke (AIS). In September 2021, the FDA granted Fast Track Designation to DM199 for the treatment of AIS.

About DiaMedica Therapeutics Inc.

DiaMedica Therapeutics Inc. is a clinical stage biopharmaceutical company committed to improving the lives of people suffering from serious diseases with a focus on acute ischemic stroke. DiaMedica's lead candidate DM199 is the first pharmaceutically active recombinant (synthetic) form of the KLK1 protein, an established therapeutic modality in Asia for the treatment of acute ischemic stroke and other vascular diseases. For more information visit the Company's website at www.diamedica.com.

Cautionary Note Regarding Forward-Looking Statements

This press release contains forward-looking statements within the meaning of the U.S. Private Securities Litigation Reform Act of 1995 and forward-looking information that are based on the beliefs of management and reflect management's current expectations. When used in this press release, the words "anticipates," "believes," "look forward," "continue," "could," "estimates," "expects," "intends," "may," "plans," "potential," "hope," "should," or "will," the negative of these words or such variations thereon or comparable terminology, and the use of future dates are intended to identify forward-looking statements and information. The forward-looking statements and information in this press release include statements regarding the Company's expectations regarding the resumption of the ReMEDy2 trial and the anticipated clinical benefits and success of DM199. Such statements and information reflect management's current view and DiaMedica undertakes no obligation to update or revise any of these statements or information. By their nature, forward-looking statements involve known and unknown risks, uncertainties and other factors which may cause actual results, performance or achievements, or other future events, to be materially different from any future results, performance or achievements expressed or implied by such forward-looking statements. Applicable risks and uncertainties include, among others, uncertainties relating to regulatory applications and related filing and approval timelines; the possibility of additional future adverse events associated with or unfavorable results from the ReMEDy2 trial; the possibility of unfavorable results from DiaMedica's ongoing or future clinical trials of DM199; the risk that existing preclinical and clinical data may not be predictive of the results of ongoing or later clinical trials; DiaMedica's plans to develop, obtain regulatory approval for and commercialize its DM199 product candidate for the treatment of acute ischemic stroke and chronic kidney disease and its expectations regarding the benefits of DM199; DiaMedica's ability to conduct successful clinical testing of DM199 and within its anticipated parameters, enrollment numbers, costs and timeframes; the adaptive design of the ReMEDy2 trial and the possibility that the targeted enrollment and other aspects of the trial could change depending upon certain factors, including additional input from the FDA and the blinded interim analysis; the perceived benefits of DM199 over existing treatment options; the potential direct or indirect impact of COVID-19, hospital and medical facility staffing shortages, and worldwide global supply chain shortages on DiaMedica's business and clinical trials, including its ability to meet its site activation and enrollment goals; DiaMedica's reliance on collaboration with third parties to conduct clinical trials; DiaMedica's ability to continue to obtain funding for its operations, including funding necessary to complete planned clinical trials and obtain regulatory approvals for DM199 for acute ischemic stroke and chronic kidney disease, and the risks identified under the heading "Risk Factors" in DiaMedica's annual report on Form 10-K for the fiscal year ended December 31, 2022 and subsequent U.S. Securities and Exchange Commission filings, including DiaMedica's quarterly report on Form 10-Q for the quarterly period ended March 31, 2023. The forward-looking information contained in this press release represents the expectations of DiaMedica as of the date of this press release and, accordingly, is subject to change after such date. Readers should not place undue importance on forward-looking information and should not rely upon this information as of any other date. While DiaMedica may elect to, it does not undertake to update this information at any particular time except as required in accordance with applicable laws.

View source version on businesswire.com:
https://www.businesswire.com/news/home/20230621362057/en/

Scott Kellen
Chief Financial Officer
Phone: (763) 496-5118
skellen@diamedica.com

Paul Papi
Corporate Communications
Phone: 617-899-5941
ppapi@diamedica.com

Source: DiaMedica Therapeutics Inc.

Released June 21, 2023

© 2024 DiaMedica Therapeutics, Inc. All Rights Reserved.

A Study to Evaluate the Safety and Effectiveness of DM199 in Healthy Subjects and Type 2 Diabetes Patients

The safety and scientific validity of this study is the responsibility of the study sponsor and investigators. Listing a study does not mean it has been evaluated by the U.S. Federal Government. Read our disclaimer for details.

ClinicalTrials.gov Identifier: NCT01845064

Recruitment Status : Completed
First Posted : May 3, 2013
Last Update Posted : December 19, 2014

View this study on the modernized ClinicalTrials.gov

Sponsor:
DiaMedica Therapeutics Inc

Information provided by (Responsible Party):
DiaMedica Therapeutics Inc

Study Details | Tabular View | No Results Posted | Disclaimer | How to Read a Study Record

Study Description

Go to

Brief Summary:

DM199 (recombinant human tissue kallikrein-1) is a new investigational compound that may eventually be used for the treatment of Diabetes Mellitus Type 2. This is the first time that this compound is being given to humans.

The purpose of the study is to investigate to what extent DM199 is safe and tolerated. Further, it will be investigated how quickly and to what extent DM99 is absorbed and eliminated from the body (this is called pharmacokinetics). In addition, the effect of the compound on the body will be investigated (this is called pharmacodynamics).

This study is not intended to improve anyone's health, but is necessary for the further development of DM199.

The study consists of 4 parts. Each part (A, B, C and D) will consist of one or several periods. The research will be conducted in healthy male and female volunteers (Part A and C) and in male and female type 2 diabetes mellitus patients (Part B and D).

Condition or disease	Intervention/treatment	Phase
Diabetes Type 2	Drug: DM199<br>Drug: Placebo	Phase 1<br>Phase 2

Detailed Description:

DM199 (recombinant human tissue kallikrein-1) is being developed as a new biological treatment for type 2 diabetes mellitus. This is a first-in-human study for DM199 and is a 4-part, single center study in healthy subjects and type 2 diabetes mellitus patients.

Part A will be a randomized, double-blinded, placebo-controlled, single ascending dose (SAD) study in healthy male and/or female subjects. Subjects will receive DM199 or placebo subcutaneously (sc).

Part B will be a randomized, partially double-blinded, placebo-controlled, sequential SAD study in male and/or female type 2 diabetes mellitus patients.

Part C will be a randomized, double-blinded, placebo-controlled, 14-day multiple ascending dose (MAD) study in healthy male and/or female subjects each. Subjects will receive sequential doses of DM-199 or placebo sc for 14 days.

Part D will be a randomized, double-blinded, placebo-controlled, 28-day multiple-dose proof of concept (POC) study in male and/or female type 2 diabetes mellitus patients. Subjects will receive parallel doses of DM199 or placebo sc for 28 days.

The primary objective is to evaluate the safety and tolerability of single and multiple subcutaneous doses of DM199 in healthy subjects and type 2 diabetes mellitus patients. Another objective is to determine the plasma pharmacokinetic profile of DM199 after administration of single and multiple doses of DM199 in healthy subjects and type 2 diabetes mellitus patients.

Secondary objectives include determining the effect of DM199 on glucose homeostasis (via fasting glucose and HbA1c levels), standardized meal tolerance test, C-peptide, fructosamine, GLP-1 (active and total), glucagon, adiponectin and lipids measurements, and homeostatic model assessment of insulin resistance/beta cell function (HOMA) determination in type 2 diabetes mellitus patients; assessing the formation of antibodies to DM199 after administration of multiple doses of DM199 in healthy subjects and type 2 diabetes mellitus patients; and determining changes in immune cell populations by fluorescence-activated cell sorting analysis following multiple doses of DM199 in healthy subjects and type 2 diabetes mellitus patients

Study Design

Go to

Study Type :	Interventional (Clinical Trial)
Actual Enrollment :	98 participants
Allocation:	Randomized
Intervention Model:	Parallel Assignment
Masking:	Quadruple (Participant, Care Provider, Investigator, Outcomes Assessor)
Primary Purpose:	Treatment
Official Title:	A Double-Blinded, Placebo-Controlled, Single-Dose and Multiple-Dose Study to Evaluate the Safety, Tolerability, Pharmacokinetics, Pharmacodynamics and Proof of Concept of DM199 in Healthy Subjects and Patients With Type 2 Diabetes Mellitus
Study Start Date :	April 2013
Actual Primary Completion Date :	September 2014
Actual Study Completion Date :	November 2014

Resource links provided by the National Library of Medicine

NIH NLM

MedlinePlus Genetics related topics: Type 2 diabetes

U.S. FDA Resources

Arms and Interventions

Go to

Arm	Intervention/treatment
Experimental: Part A - SAD in healthy subjects<br>A randomized, double-blinded, placebo-controlled, single ascending dose (SAD) study in healthy male and/or female subjects. Subjects will receive DM199 subcutaneously (sc).	Drug: DM199<br>Other Names:<br>• recombinant human tissue kallikrein-1<br>• tissue kallikrein-1
Experimental: Part B - SAD in type 2 diabetic patients<br>A randomized, partially double-blinded, placebo-controlled, sequential SAD study in male and/or female type 2 diabetes mellitus patients. Subjects will receive DM199 subcutaneously (sc).	Drug: DM199<br>Other Names:<br>• recombinant human tissue kallikrein-1<br>• tissue kallikrein-1
Experimental: Part C - MAD in healthy subjects<br>A randomized, double-blinded, placebo-controlled, 14-day multiple ascending dose (MAD) study in healthy male and/or female subjects each. Subjects will receive sequential doses of DM199 sc for 14 days.	Drug: DM199<br>Other Names:<br>• recombinant human tissue kallikrein-1<br>• tissue kallikrein-1
Experimental: Part D - POC in type 2 diabetes patients<br>A randomized, double-blinded, placebo-controlled, 28-day multiple-dose proof of concept (POC) study in male and/or female type 2 diabetes mellitus patients. Subjects will receive doses of DM199 sc for 28 days.	Drug: DM199<br>Other Names:<br>• recombinant human tissue kallikrein-1<br>• tissue kallikrein-1
Placebo Comparator: Part A - Healthy subjects SAD placebo<br>A randomized, double-blinded, placebo-controlled, single ascending dose (SAD) study in healthy male and/or female subjects. Subjects will receive placebo subcutaneously (sc).	Drug: Placebo
Placebo Comparator: Part B - Type 2 diabetic patients SAD placebo<br>A randomized, partially double-blinded, placebo-controlled, sequential SAD study in male and/or female type 2 diabetes mellitus patients. Subjects will receive placebo subcutaneously (sc).	Drug: Placebo
Placebo Comparator: Part C - Healthy subjects MAD placebo<br>A randomized, double-blinded, placebo-controlled, 14-day multiple ascending dose (MAD) study in healthy male and/or female subjects each. Subjects will receive placebo sc for 14 days.	Drug: Placebo
Placebo Comparator: Part D - Type 2 diabetic patients POC placebo<br>A randomized, double-blinded, placebo-controlled, 28-day multiple-dose proof of concept (POC) study in male and/or female type 2 diabetes mellitus patients. Subjects will receive placebo sc for 28 days.	Drug: Placebo

Outcome Measures

Go to

Primary Outcome Measures :

1. Safety and tolerability of single and multiple subcutaneous doses of DM199 [Time Frame: Up to 13 days after final dose]

   Number of participants with adverse events in the single and multiple ascending dose studies.

2. Determine the pharmacokinetic of DM199 after single and multiple doses [Time Frame: Up to 3 days after final dose]

   Determine the plasma pharmacokinetic profile of DM199 after administration of single and multiple doses of DM199 in healthy subjects and type 2 diabetes mellitus patients. Measure plasma DM199 levels in individual participants.

Secondary Outcome Measures :

1. Determine the effect of DM199 on glucose homeostasis in healthy volunteers and type 2 diabetes mellitus patients [Time Frame: Part C, Day -1 and 14; Part D, Days -1, 14 and 28]

   Determine the effect of DM199 on glucose homeostasis (via fasting glucose and HbA1c levels), standardized meal tolerance test, C-peptide, fructosamine, GLP-1 (active and total), glucagon, adiponectin and lipids measurements, and homeostatic model assessment of insulin resistance/beta cell function (HOMA) determination in type 2 diabetes mellitus patients

2. Assess formation of ADA to DM199 [Time Frame: Part C, Day -1 and 42; Part D, Day -1 and 35]

   Assess the formation of antibodies to DM199 after administration of multiple doses of DM199 in healthy subjects and type 2 diabetes mellitus patients

3. Determine changes in immune cell populations by FACS analysis. [Time Frame: Part C, Day -1 and 15; Part D, Day -1 and 29]

   Determine changes in immune cell populations by fluorescence-activated cell sorting analysis following multiple doses of DM199 in healthy subjects and type 2 diabetes mellitus patients

Eligibility Criteria

Go to

Information from the National Library of Medicine

NIH NLM

Choosing to participate in a study is an important personal decision. Talk with your doctor and family members or friends about deciding to join a study. To learn more about this study, you or your doctor may contact the study research staff using the contacts provided below. For general information, Learn About Clinical Studies.

Ages Eligible for Study:	18 Years to 75 Years (Adult, Older Adult)
Sexes Eligible for Study:	All
Accepts Healthy Volunteers:	Yes

Criteria

Inclusion Criteria:

1. Status : Parts A and C: healthy subjects
   - Parts B and D: type 2 diabetes mellitus patients :
2. Body Mass Index : Parts A and C: 18.0 - 30.0 kg/m2
   - Parts B and D: 25.0 - 35.0 kg/m2
3. HbA1c : Parts B and D: at screening between 6.5% and 9.0%, inclusive for patients using one oral anti-diabetic medication, and between 6.0% and 8.5%, inclusive for patients using two or more oral anti-diabetic medications
4. Fasting blood glucose : Parts B and D: within 7.5-13.5 mmol/L, inclusive at entry into the clinical research center (Day -1 for Part B or Day -2 for Part D)
5. Women of childbearing potential agree to use an appropriate contraceptive method (hormonal, IUD, or diaphragm) until 90 days after the follow-up visit. For males: willingness to use adequate contraception from entry in the clinical research center until 90 days after the follow-up visit
6. Medical history without clinically significant abnormalities
7. Parts B and D: Taking a stable dose of one or more oral anti-diabetic medications, such as metformin, sulphonylurea or any other orally administered glucose lowering medication (except for thiazolidinediones) for at least 3 months prior to screening. Receiving no other chronic medications, including dietary supplements, that alter blood glucose control.
8. Parts A and C: Resting supine blood pressure of 140/90 mmHg or lower and higher than 90/50mmHg at screening, and showing no clinically relevant deviations as judged by the Principal Investigator
9. Parts B and D: Resting supine blood pressure of 160/100 mmHg or lower and higher than 90/50mmHg at screening, and showing no clinically relevant deviations as judged by the Principal Investigator

Exclusion Criteria:

1. Evidence of clinically relevant pathology
2. Pregnancy or lactation
3. For healthy volunteers: use of concomitant medication, except for acetaminophen (paracetamol), which is allowed up to 3 days before entry into the clinical research center (after that time the use of a limited amount of acetaminophen is permitted after consultation with the Principal Investigator). Multivitamins and vitamin C are allowed up to 7 days before entry into the clinical research center. All other medication (including over the counter medication, health supplements, and herbal remedies such as St. John's Wort extract) must have been stopped at least 14 days prior to entry into the clinical research center.
4. Participation in a drug study within 60 days prior to drug administration. Participation in more than 3 other drug studies (for men) / more than 2 other drug studies (for women) in the 10 months preceding the start of this study)
5. Positive drug screen (opiates, methadone, cocaine, amphetamines, cannabinoids, barbiturates, benzodiazepines, tricyclic antidepressants and alcohol)
6. Intake of more than 24 units of alcohol per week (one unit of alcohol equals approximately 250 mL of beer, 100 mL of wine or 35 mL of spirits)
7. Positive screen on HBsAg, anti-HCV or anti-HIV 1/2
8. Illness within 7 days prior to (the first) drug administration
9. Serum creatinine > upper limit of the normal (ULN) range

   Additional Exclusion Criteria Specific to Type 2 Diabetes Mellitus Patients (Part B and Part D)

10. The use of insulin and thiazolidinediones for type 2 diabetes mellitus 3 months prior to screening is not allowed.
11. The use of angiotensin converting enzyme (ACE) inhibitors 1 month prior to screening is not allowed.
12. History of diabetic ketoacidosis or hyperosmolar coma
13. Advanced diabetic complications, including neuropathy, nephropathy, retinopathy or other symptoms

Contacts and Locations

Go to

Information from the National Library of Medicine

NIH NLM

To learn more about this study, you or your doctor may contact the study research staff using the contact information provided by the sponsor.

Please refer to this study by its ClinicalTrials.gov identifier (NCT number): ***NCT01845064***

Locations

Netherlands

PRA
Zuidlaren, Netherlands, 9471 GP

Sponsors and Collaborators

DiaMedica Therapeutics Inc

Investigators

Principal Investigator: Salah Hadi, MD, MSc PRA

More Information

Go to

Responsible Party:	DiaMedica Therapeutics Inc
ClinicalTrials.gov Identifier:	NCT01845064 History of Changes
Other Study ID Numbers:	DMA-Clin-199-2013-001<br>2013-000225-30 (EudraCT Number)
First Posted:	May 3, 2013 Key Record Dates
Last Update Posted:	December 19, 2014
Last Verified:	December 2014

Keywords provided by DiaMedica Therapeutics Inc:

type 2 diabetes	DiaMedica
diabetes mellitus	DM-199
type 2 diabetes mellitus	DM199
HbA1c	recombinant human tissue kallikrein-1
blood glucose	tissue kallikrein-1
glucose control	

Additional relevant MeSH terms:

Diabetes Mellitus	Coagulants
Diabetes Mellitus, Type 2	Fertility Agents, Male
Glucose Metabolism Disorders	Fertility Agents
Metabolic Diseases	Reproductive Control Agents
Endocrine System Diseases	Physiological Effects of Drugs
Kallikreins	

This is the classic website, which will be retired eventually. Please visit the modernized ClinicalTrials.gov instead.

Multiple Doses of DM199 in Patients With Chronic Kidney Disease (REDUX)

The safety and scientific validity of this study is the responsibility of the study sponsor and investigators. Listing a study does not mean it has been evaluated by the U.S. Federal Government. Read our disclaimer for details.

ClinicalTrials.gov Identifier: NCT04123613

Recruitment Status : Completed
First Posted : October 11, 2019
Last Update Posted : March 31, 2022

View this study on the modernized ClinicalTrials.gov

Sponsor:
DiaMedica Therapeutics Inc

Information provided by (Responsible Party):
DiaMedica Therapeutics Inc

Study Description

Brief Summary:
An open-label, Phase II, **multi-**center study evaluating **multiple doses of DM199** in participants with **chronic kidney disease**.

Condition or disease	Intervention/treatment	Phase
**Kidney Diseases**	Drug: **DM199**	Phase 2

Detailed Description:

This is an open-label, Phase II, multi-center study evaluating DM199 in approximately 90 Participants in three cohorts.

Cohort I: African Americans with CKD (Stage II or III), hypertension and non-diabetic Cohort II: Participants with IgA nephropathy diagnosis and CKD (Stage II or III) Cohort III: Diabetes Mellitus (Type II) with CKD (Stage II or III) and hypertension

Participants in each cohort will be enrolled in a parallel assignment to one of two doses:

Dose 1: DM199 2.0 µg/kg SC 2x week for 95 days Dose 2: DM199 5.0 µg/kg SC 2x week for 95 days

Study Design

Study Type :	Interventional (Clinical Trial)
Actual Enrollment :	79 participants
Allocation:	Non-Randomized
Intervention Model:	Parallel Assignment
Masking:	None (Open Label)
Primary Purpose:	Treatment
Official Title:	A **Multi-**Center Open-label Investigation to Assess the Safety and Efficacy of **Multiple Doses of DM199 in Patients With Chronic Kidney Disease**
Actual Study Start Date :	December 17, 2019
Actual Primary Completion Date :	March 16, 2022
Actual Study Completion Date :	March 16, 2022

Resource links provided by the National Library of Medicine NIH NLM

MedlinePlus related topics: Kidney Diseases

Genetic and Rare Diseases Information Center resources: Chronic Graft Versus Host Disease

U.S. FDA Resources

Arms and Interventions

Arm	Intervention/treatment
Experimental: 2.0 µg/kg, **multiple dose**<br>n=45 with 15 Participants from cohort 1, 15 from cohort 2, and 15 from cohort 3	Drug: **DM199**<br>A pharmaceutical formulation comprised of recombinant human tissue kallikrein-1 (rhKLK-1) that is being developed as an injectable protein drug
Experimental: 5.0 µg/kg, **multiple dose**<br>n=45 with 15 Participants from cohort 1, 15 from cohort 2 and 15 from cohort 3	Drug: **DM199**<br>A pharmaceutical formulation comprised of recombinant human tissue kallikrein-1 (rhKLK-1) that is being developed as an injectable protein drug

Outcome Measures

Primary Outcome Measures :

1. Incidence of treatment emergent adverse events [Time Frame: 12 weeks]
   Incidence, severity, and causality of adverse events
2. Change in **renal** function [Time Frame: 12 weeks]
   eGFR
3. Change in urine albumin to creatinine ratio [Time Frame: 12 weeks]
   UACR change from baseline
4. Plasma measurements of **DM199** [Time Frame: 12 weeks]
   Maximum plasma concentration of **DM199**

Secondary Outcome Measures :

1. Tumor necrosis factor receptor 1 (TNF R1) concentration in plasma, change from baseline [Time Frame: 12 weeks]
   TNF R1 change from baseline
2. C-reactive protein (CRP) concentration in plasma, change from baseline [Time Frame: 12 weeks]
   CRP change from baseline
3. Matrix metalloproteainase-9 (MMP-9) concentration in plasma, change from baseline [Time Frame: 12 weeks]
   MMP-9 change from baseline
4. Vascular endothelial growth factor (VEGF) concentration in plasma, change from baseline [Time Frame: 12 weeks]
   VEGF change from baseline
5. Cystatin C concentration in plasma, change from baseline [Time Frame: 12 weeks]
   Cystatin C change from baseline
6. Prostaglandin E2 concentration in plasma, change from baseline [Time Frame: 12 weeks]
   Prostaglandin E2 change from baseline
7. Prostacyclin concentration in plasma, change from baseline [Time Frame: 12 weeks]
   Prostacyclin change from baseline

Eligibility Criteria

Information from the National Library of Medicine NIH NLM

Choosing to participate in a study is an important personal decision. Talk with your doctor and family members or friends about deciding to join a study. To learn more about this study, you or your doctor may contact the study research staff using the contacts provided below. For general information, Learn About Clinical Studies.

Ages Eligible for Study:	18 Years and older (Adult, Older Adult)
Sexes Eligible for Study:	All
Accepts Healthy Volunteers:	No

Criteria

Inclusion Criteria:

Cohort I

- African American
- Hypertension as defined by the American Heart Association for Stage I hypertension where systolic blood pressure (BP) ≥130 mmHg or diastolic BP ≥ 80 mmHg or on medication for treatment of hypertension.

Cohort II

- IgA nephropathy confirmed by medical history with biopsy

Cohort III

- Diabetes Mellitus (Type 2) with hypertension where systolic blood pressure (BP) ≥130 mmHg or diastolic BP ≥ 80 mmHg or on medication for treatment of hypertension
- Hemoglobin A1c ≥7% at screening

Both Cohorts

- Participant is willing and able to provide informed consent for study participation
- Participant male or female ≥ 18 years of age
- Participant has CKD as defined by using CKD EPI for Stage II 60 to <90 mL/min/1.73 m2 or Stage III 30 to <60 mL/min/1.73 m2
- UACR >150 mg/g and <5000 mg/g at screening
- Participant is clinically stable with respect to underlying renal impairment as assessed by the Investigator's medical evaluation

Exclusion Criteria:

- Participant has positive drug test for drugs of abuse and/or positive alcohol breath test at screening and Day 1
- Participant has a current diagnosis and/or is taking medication or diet control for diabetes (cohort I and II only)
- Participant has an A1c > 7% at screening (cohort I and II only)
- Participant received corticosteroid therapy within last 3 months
- Participant is unable or unwilling to comply with protocol requirements, including assessments, tests, and follow-up visits
- Participant has a history of significant allergic diathesis such as urticaria, angioedema, or anaphylaxis
- Participant has been previously diagnosed with kidney disease other than for hypertension, IgA or Diabetes Mellitus (Type II)
- Participant has hypotension as defined by systolic blood pressure ≤ 90 mmHg and diastolic blood pressure ≤ 60 mmHg at screen
- ACEi or GLP-1 medication prescribed for and taken by Participant (must not be taking for 5 half-lives prior to study drug administration and for 10 days post study drug administration)
- Participant has a current malignancy or active malignancy ≤ 2 years prior to enrollment except basal cell or squamous cell carcinoma of the skin or in situ cervical cancer that has undergone potentially curative therapy and ≥ 6 months have elapsed since the procedure
- Participant has an active infection at the time of enrollment, and/or a history of clinically significant acute bacterial, viral, or fungal systemic infections that required systemic treatment with a completed therapy in the last 7 days prior to enrollment
- Participant has known medical history of alpha 1-antitrypsin deficiency (α1-antitrypsin deficiency)
- Participant is pregnant or nursing or is planning a pregnancy during the study period
- Participant is male or female of childbearing potential, is participating in sexual activity that could lead to pregnancy and is unable or unwilling to practice medically effective contraception during the study
- Participant has received any investigational drug or device within 14 days (or 5 half lives, whichever is longer) prior to study drug administration starting on Day 1
- Participant has renal artery stenosis as determined at screen with medical history
- Participant received a kidney transplant
- Participant does not have adequate venous access for blood sampling
- Participant has any other medical condition which, in the opinion of the Investigator, will make participation medically unsafe or interfere with the study results
- Participant has any other clinically significant abnormalities in laboratory test results at screening that would, in the opinion of the Investigator, increase the Participant's risk of participation, jeopardize complete participation in the study, or compromise interpretation of study data
- Participant has any significant arrhythmia or conduction abnormality, which in the opinion of the Investigators and Medical Monitor may interfere with the safety of the Participant

Contacts and Locations

Information from the National Library of Medicine NIH NLM

To learn more about this study, you or your doctor may contact the study research staff using the contact information provided by the sponsor.

Please refer to this study by its ClinicalTrials.gov identifier (NCT number): ***NCT04123613***

Locations

United States, Arizona
Aventiv Research
Mesa, Arizona, United States, 85210

United States, California
Amcis Research Center
Granada Hills, California, United States, 91334
IMD Clinical Trials Inc
Los Angeles, California, United States, 90033
Amicis Reserch Center
Northridge, California, United States, 91324

United States, Florida
Innovative Healthcare Institute
Coral Springs, Florida, United States, 33067
Elixia at Florida **Kidney** Physicians-SE
Fort Lauderdale, Florida, United States, 33308
Pines Clinical Research-Hollywood
Hollywood, Florida, United States, 33024
Elixia at Florida **Kidney** Physicians
Temple Terrace, Florida, United States, 33637

United States, Idaho
Boise **Kidney** & Hypertension Institute
Meridian, Idaho, United States, 83642

United States, Illinois
Research by Design LLC
Chicago, Illinois, United States, 60643

United States, Louisiana
New Orleans Center for Clinical Research, an AMR Company
New Orleans, Louisiana, United States, 70119

United States, Pennsylvania
Elixia At Clincal **Renal** Associates
Upland, Pennsylvania, United States, 19013

United States, Texas
Nephrotex Research Group, LLC
Dallas, Texas, United States, 75231
RDRI
DeSoto, Texas, United States, 75115

Sponsors and Collaborators
DiaMedica Therapeutics Inc

Investigators
Study Director: Harry Alcorn, Pharm.D. DiaMedica Inc.

More Information

Responsible Party:	DiaMedica Therapeutics Inc
ClinicalTrials.gov Identifier:	NCT04123613 History of Changes
Other Study ID Numbers:	**DM199-**2019-001
First Posted:	October 11, 2019 Key Record Dates
Last Update Posted:	March 31, 2022
Last Verified:	March 2022

Individual Participant Data (IPD) Sharing Statement:
Plan to Share IPD: No

Studies a U.S. FDA-regulated Drug Product: Yes
Studies a U.S. FDA-regulated Device Product: No

Additional relevant MeSH terms:

Kidney Diseases
Renal Insufficiency, **Chronic**
Urologic **Diseases**
Female Urogenital **Diseases**
Female Urogenital **Diseases** and Pregnancy Complications
Urogenital **Diseases**
Male Urogenital **Diseases**
Renal Insufficiency
Chronic Disease
Disease Attributes
Pathologic Processes

This is the classic website, which will be retired eventually. Please visit the modernized ClinicalTrials.gov instead.

Notice to API users: Read about imminent changes impacting usage of the ClinicalTrials.gov API.

A Multicenter Study to Evaluate the Efficacy and Safety of Cinryze® for the Treatment of Acute Antibody-mediated Rejection in Participants With Kidney Transplant

The safety and scientific validity of this study is the responsibility of the study sponsor and investigators. Listing a study does not mean it has been evaluated by the U.S. Federal Government. Read our disclaimer for details.

ClinicalTrials.gov Identifier: NCT02547220

Recruitment Status : Terminated (Following a pre-scheduled interim analysis performed by the DMC, it was determined that the study met the pre-specified criteria for futility.)
First Posted : September 11, 2015
Results First Posted : July 13, 2020
Last Update Posted : July 13, 2020

View this study on the modernized ClinicalTrials.gov

Sponsor:
Shire

Information provided by (Responsible Party):
Takeda (Shire)

Study Description

Brief Summary:
The main purpose of the study is to evaluate the efficacy of **CINRYZE** administered with plasmapheresis, plasma exchange, or immune adsorption treatments and sucrose-free immunoglobulin (IVIg) for the treatment of acute antibody-mediated rejection (AMR) of renal allograft in kidney transplant recipients as measured by the proportion of participants with new or worsening transplant glomerulopathy (TG) at 6 months after treatment initiation.

Condition or disease	Intervention/treatment	Phase
Acute Antibody-Mediated Rejection (AMR)	Biological: **Cinryze®**<br>Drug: Placebo	Phase 3

Study Design

Study Type : Interventional (Clinical Trial)
Actual Enrollment : 39 participants
Allocation: Randomized
Intervention Model: Parallel Assignment
Masking: Triple (Participant, Investigator, Outcomes Assessor)
Primary Purpose: Treatment
Official Title: A Randomized Double-blind Placebo-controlled Study to Evaluate the Efficacy and Safety of **Cinryze®** (**C1 Esterase Inhibitor** [Human]) for the Treatment of Acute Antibody-mediated Rejection in Kidney Transplant Patients
Actual Study Start Date : May 20, 2016
Actual Primary Completion Date : May 31, 2019
Actual Study Completion Date : May 31, 2019

Resource links provided by the National Library of Medicine

MedlinePlus related topics: Kidney Transplantation

Drug Information available for: SERPING1 protein, human

Genetic and Rare Diseases Information Center resources: Acute Graft Versus Host Disease

U.S. FDA Resources

Arms and Interventions

Arm	Intervention/treatment
Experimental: **Cinryze®**<br>Participants will receive 5000 Units of **CINRYZE** (50 millilitre [mL] of **CINRYZE**/ 50 mL of normal saline) on Day 1 and 2500 Units of **CINRYZE** (25 mL of **CINRYZE**/ 75 mL of normal saline) on Day 3, 5, 7, 9, 11, and 13 respectively.	Biological: **Cinryze®**<br>Participants will receive 5000 Units of **CINRYZE** (50 millilitre [mL] of **CINRYZE**/ 50 mL of normal saline) on Day 1 and 2500 Units of **CINRYZE** (25 mL of **CINRYZE**/ 75 mL of normal saline) on Day 3, 5, 7, 9, 11, and 13 respectively.
Placebo Comparator: Placebo<br>Participants will receive 7 doses of matched placebo over 13 days of treatment.	Drug: Placebo<br>Participants will receive 7 doses of matched placebo over 13 days of treatment.

Outcome Measures

Primary Outcome Measures :

1. Percentage of Participants With New or Worsening Transplant Glomerulopathy (TG) at Month 6 Post-Treatment [Time Frame: Month 6]

   New or worsening TG at month 6 by the standard score was defined as an increase in one or more between qualifying biopsy and 6-month biopsy. New or worsening TG was measured by Banff 2013 criteria (standard score) using allograft glomerulopathy (Cg0-Cg3): Cg0- No GBM double contours by light microscopy (LM) or electron microscopy (EM); Cg1- no GBM double contours by LM but GBM double contours in at least 3 glomerular capillaries by EM; Cg2- Double contours affecting 26 to 50% of peripheral capillary loops in the most affected of nonsclerotic glomeruli; Cg3- Double contours affecting more than 50% of peripheral capillary loops in the most affected of nonsclerotic glomeruli with a score range of 0 (no allograft glomerulopathy) and 3 (severe glomerulopathy). Percentage of participants with new or worsening TG at Month 6 post-treatment was reported.

Secondary Outcome Measures :

1. Number of Participants With All-Cause Graft Failure at Month 48 [Time Frame: Month 48]

   Graft failure was determined as the presence of one or more of the following criteria: institution of permanent dialysis (defined as dialysis treatment more than [>] 30 days), current transplant nephrectomy, and/or a clinical determination of cessation of kidney graft function and estimated glomerular filtration rate (eGFR) less than or equal to (<=) 15 milliliter (mL)/minute (min)/1.73 meter (m)^2.

2. Change From Baseline in Renal Function up to Month 48 [Time Frame: Baseline, up to Month 48]

   Renal function was measured as glomerular filtration rate calculated by the modification of diet in renal disease (eGFRMDRD).

3. Change From Baseline With Pre-Antibody-Mediated Rejection (AMR) in Renal Function up to Month 48 [Time Frame: Pre-AMR Baseline, up to Month 48]

   Renal function was measured as glomerular filtration rate calculated by the modification of diet in renal disease (eGFRMDRD). Pre-AMR baseline was the highest eGFRMDRD value obtained following the kidney transplant and within 30 days prior to the qualifying AMR episode.

4. Number of Participants With Proteinuria Levels at Month 48 [Time Frame: Month 48]

   Proteinuria included spot urine protein, urine creatinine, and urine protein/urine creatinine ratio.

5. Change From Pre-Antibody-Mediated Rejection (AMR) Baseline in Histopathology Per Banff Criteria at Month 6 [Time Frame: Pre-AMR Baseline, Month 6]

   Histopathological diagnosis of acute rejection was measured by Banff 2013 criteria: Glomerulitis score (g0-g3), allograft glomerulopathy (Cg0-cg3), Tubulitis score (T0-T3), Intimal arteritis score (V0-V3), peritubular capillaritis (PTC) (ptc0-ptc3) and Interstitial Inflammation score (i0-i3). The histopathology was a composite of the sub-scores. Each of the sub-scores or histopathology score ranges from 0 (no histopathology) to 3 (more severe histopathology).

6. Number of Participants With All-Cause Graft Failure at Month 6 [Time Frame: Month 6]

   Graft failure was determined by the presence of one or more of the following criteria: institution of permanent dialysis (defined as dialysis treatment more than [>] 30 days), current transplant nephrectomy, and/or a clinical determination of cessation of kidney graft function and estimated glomerular filtration rate (eGFR) less than or equal to (<=) 15 milliliter (mL)/minute (min)/1.73 meter (m)^2.

7. Number of Participants With Graft Failure Due to Antibody-Mediated Rejection (AMR) Episodes at Month 48 [Time Frame: Month 48]

   Graft failure was determined by the presence of one or more of the following criteria: institution of permanent dialysis (defined as dialysis treatment more than [>] 30 days), current transplant nephrectomy, and/or a clinical determination of cessation of kidney graft function and estimated glomerular filtration rate (eGFR) less than or equal to (<=) 15 milliliter (mL)/minute (min)/1.73 meter (m)^2.

8. Time to All-Cause Graft Failure up to Month 48 [Time Frame: Up to Month 48]

   Graft failure was determined by the presence of one or more of the following criteria: institution of permanent dialysis (defined as dialysis treatment more than [>] 30 days), current transplant nephrectomy, and/or a clinical determination of cessation of kidney graft function and estimated glomerular filtration rate (eGFR) less than or equal to (<=) 15 milliliter (mL)/minute (min)/1.73 meter (m)^2. Time to all-cause graft failure in months was calculated as (Date of graft failure - Date of first dose + 1)/30.25.

9. Time to Graft Failure Due to Antibody-Mediated Rejection (AMR) Episodes up to Month 48 [Time Frame: Up to Month 48]

   Graft failure was determined by the presence of one or more of the following criteria: institution of permanent dialysis (defined as dialysis treatment > 30 days), current transplant nephrectomy, and/or a clinical determination of cessation of kidney graft function and eGFR <=15 mL/ min/1.73m^2. Time to graft failure due to AMR episodes in months was calculated as (Date of graft failure due to AMR - Date of first dose + 1)/30.25.

10. Number of Participants With Resolution of the Qualifying Antibody-Mediated Rejection (AMR) Episodes at Month 48 [Time Frame: Month 48]

    Number of participants with resolution of the qualifying AMR episodes at Month 48.

11. Time to Resolution of Qualifying Antibody-Mediated Rejection (AMR) Episodes up to Month 48 [Time Frame: Up to Month 48]

    Time to resolution of qualifying AMR episodes was calculated as (Date of qualifying AMR resolution - Date of first dose + 1)/30.25. Participants who didn't had resolution of qualifying AMR episodes and still on-study were censored at the date of last visit; Participants who had completed the study without resolution of qualifying AMR were censored at the date of study completion; participants who discontinued from the study without resolution of qualifying AMR were censored at the date of early discontinuation.

12. Number of Participants Who Were Alive at Month 36 [Time Frame: Month 36]

    Number of participants who were alive at Month 36 (study terminated instead of Month 48) were reported.

13. Time to All-Cause Mortality up to Month 48 [Time Frame: Up to Month 48]

    Time to all-cause mortality was calculated as (Date of discontinuation due to death - Date of first dose + 1)/30.25. Participants who are alive and still on-study were censored at the date of last visit; Participants who had completed the study were censored at the date of study completion; Participants who discontinued from the study but not due to death were censored at the date of early discontinuation.

14. Number of Participants With Treatment-emergent Adverse Events (TEAEs) [Time Frame: From start of study drug administration up to study termination (Month 36)]

    An adverse event (AE) was any untoward, undesired, unplanned clinical event in the form of signs, symptoms, disease, or laboratory or physiological observations occurred in a participant participating in a clinical study with the sponsor's product, regardless of causal relationship. TEAEs were defined as events that started or worsened on or after the date of the first dose of investigational product, but no later than 30 days following the last dose of investigational product, within a treatment period.

Eligibility Criteria

Information from the National Library of Medicine

Choosing to participate in a study is an important personal decision. Talk with your doctor and family members or friends about deciding to join a study. To learn more about this study, you or your doctor may contact the study research staff using the contacts provided below. For general information, Learn About Clinical Studies.

Ages Eligible for Study: 18 Years to 70 Years (Adult, Older Adult)
Sexes Eligible for Study: All
Accepts Healthy Volunteers: No

Criteria

Inclusion Criteria:

1. Be greater than or equal to (>=) 18 and less than or equal to (<=) 70 years of age.
2. Weigh >= 45 kg with a body mass index (BMI) less than (<) 35 kilogram (kg)/meter (m)^2 at screening.
3. Have human leukocyte antigen (HLA) donor-specific antibody (DSA) identified at the time of diagnosis of AMR. If it is anticipated that the local DSA results will not be available within the screening period, previously obtained local DSA results can be used to assess eligibility, if obtained after kidney transplant and within 30 days prior to the qualifying AMR episode. In any instance, a local DSA test should still be performed at the time of AMR diagnosis.
4. Have a first qualifying episode of AMR in the participant's current renal allograft between 72 hours (h) and 12 months after transplant defined by a renal allograft biopsy demonstrating neutrophil and/or monocyte infiltration in the peritubular capillaries (PTC) and/or glomeruli with or without evidence of 4th complement protein degradation product (C4d) deposition by immunohistopathology according to 2013 Banff criteria.
5. Have achieved adequate renal function defined as: Pre-AMR baseline estimated glomerular filtration rate calculated by the Modification of Diet in Renal Disease (eGFRMDRD) >=20 millilitre (mL)/minute (min) /1.73m^2 for a qualifying AMR episode occurring <=21 days after transplant or pre-AMR baseline eGFRMDRD >=30 mL/min/1.7m^2 for a qualifying AMR episode occurring greater than (>) 21 days after transplant. The pre-AMR baseline is the highest eGFRMDRD value obtained following the kidney transplant and within 30 days prior to the qualifying AMR episode. If more than 1 eGFRMDRD value is available, a mean of the 2 highest values (at least 1 day apart and both prior to the AMR episode) will be used as the pre-AMR baseline value. If no eGFRMDRD was obtained within 30 days prior to biopsy, it can be evaluated within a 60 day period.
6. Receive first dose of investigational product after 7 days after the kidney transplant procedure and within 7 days after the qualifying renal allograft biopsy procedure that was positive for AMR.
7. Be informed of the nature of the study and provide written informed consent before any study-specific procedures are performed.
8. If female and of child-bearing potential, must have a negative urine pregnancy test confirmed by a negative serum beta human chorionic gonadotropin (beta-HCG) pregnancy test at the Screening Visit and must have a negative urine pregnancy test at the Day 1 visit.
9. Agree to comply with any applicable contraceptive requirements of the protocol.

Exclusion Criteria:

1. Have received pediatric en bloc kidney transplant.
2. Have primary Focal Segmental Glomerulosclerosis, rapidly progressive glomerulonephritis, membrano-proliferative glomerulonephritis type 1 (including C3 glomerulopathy), "dense deposit disease", or thrombotic microangiopathy as the cause of native kidney failure.
3. Have prior or concurrent non-renal solid organ transplant or hematopoietic stem cell transplant (HSCT) or have more than 2 completed kidney transplant procedures (note: 1 double kidney transplant procedure is considered to be 1 procedure).
4. Have a known neoplastic lesion in the transplanted allograft
5. Have, any ongoing infection that causes hemodynamic compromise or as determined by the investigator, any surgical or medical condition that could interfere with the administration of investigational product, interpretation of study results, or could compromise participant safety, including (as determined by the transplanting surgeon and documented in the operative report) any major technical complications of the renal artery, renal vein, or ureteral anastomosis
6. Have ongoing treatment for hepatitis C virus (HCV) infection.
7. Have had a recent myocardial infarction (MI) within the past 6 months and/or at the time of screening are treated with anticoagulants and/or antiplatelet agents (excluding aspirin) for a previous myocardial infarction.
8. Have a history of: abnormal bleeding, clotting events or disorders (excluding a history of clotted hemodialysis access or superficial thrombophlebitis in the absence of medically confirmed coagulopathy), any coagulopathy (documented or clinically suspected) For example, participants should be excluded if they have a history of renal allograft arterial or venous thrombosis, deep vein thrombosis, pulmonary embolism, ischemic cerebrovascular accident (stroke) or transient ischemic attack (TIA), any large vessel thrombosis.
9. Have a history of allergic reaction to CINRYZE or other blood products.
10. Have had any change in androgen therapy (example, danazol, oxandrolone, stanozolol, testosterone), tranexamic acid, epsilon-aminocaproic acid, or other fibrinolytics within 3 months before the first dose of investigational product.
11. Have participated in the active dosing phase of any other investigational drug study within 30 days prior to dosing with investigational product.
12. Have any of the following local laboratory values reported prior to dosing with investigational product: Within 24 h prior to participant dosing, white blood cell (WBC) count <0.5×109/litre (L) or >20×109/L (the value of >20×109/L should be excluded if obtained during steroid treatment), Within 24 h prior to participant dosing platelet count <25×109/L or >600×109/L
13. Be pregnant or breastfeeding.
14. Have received any of the following agents within 1 month prior to the first dose of investigational product: Sucrose-containing intravenous immunoglobulin (IVIg), Any C1 inhibitor (C1 INH) (plasma-derived [example, CINRYZE®, Berinert®, Cetor®] or recombinant [example, Rhucin®]), Eculizumab (Soliris®), Ecallantide (Kalbitor®).

Contacts and Locations

Information from the National Library of Medicine

To learn more about this study, you or your doctor may contact the study research staff using the contact information provided by the sponsor.

Please refer to this study by its ClinicalTrials.gov identifier (NCT number): ***NCT02547220***

Locations

Show 47 study locations

Sponsors and Collaborators

Shire

Investigators

Study Director: Study Director Shire

Study Documents (Full-Text)

Documents provided by Takeda (Shire):

Study Protocol [PDF] November 22, 2017

Statistical Analysis Plan [PDF] June 27, 2019

More Information

Publications automatically indexed to this study by ClinicalTrials.gov Identifier (NCT Number):

Karpman D, Bekassy Z, Grunenwald A, Roumenina LT. A role for complement blockade in kidney transplantation. Cell Mol Immunol. 2022 Jul;19(7):755-757. doi: 10.1038/s41423-022-00854-5. Epub 2022 Mar 24. No abstract available.

Responsible Party: Shire
ClinicalTrials.gov Identifier: NCT02547220 History of Changes
Other Study ID Numbers: SHP616-302
2015-000726-11 (EudraCT Number)
First Posted: September 11, 2015 Key Record Dates
Results First Posted: July 13, 2020
Last Update Posted: July 13, 2020
Last Verified: May 2020

Individual Participant Data (IPD) Sharing Statement:
Plan to Share IPD: Yes
Plan Description: Shire provides access to the de-identified individual participant data for eligible studies to aid qualified researchers in addressing legitimate scientific objectives. These IPDs will be provided following approval of a data sharing request, and under the terms of a data sharing agreement.
Supporting Materials: Study Protocol
Statistical Analysis Plan (SAP)
Informed Consent Form (ICF)
Clinical Study Report (CSR)
Access Criteria: IPD from eligible studies will be shared with qualified researchers according to the criteria and process described in the Data Sharing section of the www.shiretrials.com website. For approved requests, the researchers will be provided access to anonymized data (to respect patient privacy in line with applicable laws and regulations) and with information necessary to address the research objectives under the terms of a data sharing agreement.
URL: https://www.shiretrials.com/en/our-commitment-to-transparency/data-sharing-with-researchers

Studies a U.S. FDA-regulated Drug Product: Yes
Studies a U.S. FDA-regulated Device Product: No

Additional relevant MeSH terms:
Complement **C1 Inhibitor** Protein
Complement Inactivating Agents
Immunosuppressive Agents
Immunologic Factors
Physiological Effects of Drugs

This is the classic website, which will be retired eventually. Please visit the modernized ClinicalTrials.gov instead.

Notice to API users: Read about imminent changes impacting usage of the ClinicalTrials.gov API.

Recombinant Human C1 Esterase Inhibitor (Conestat Alfa) in the Prevention of Acute Ischemic Cerebral and Renal Events After Transcatheter Aortic Valve Implantation (PAIR-TAVI)

The safety and scientific validity of this study is the responsibility of the study sponsor and investigators. Listing a study does not mean it has been evaluated by the U.S. Federal Government. Know the risks and potential benefits of clinical studies and talk to your health care provider before participating. Read our disclaimer for details.

ClinicalTrials.gov Identifier: NCT05145283

Recruitment Status : Recruiting
First Posted : December 6, 2021
Last Update Posted : February 2, 2023

See **Contacts and Locations**

View this study on the modernized ClinicalTrials.gov

Sponsor:
University Hospital, Basel, Switzerland

Collaborators:
Swiss National Science Foundation
Pharming Technologies B.V.

Information provided by (Responsible Party):
University Hospital, Basel, Switzerland

Study Description

Brief Summary:
The aim of this trial is to assess the safety and efficacy of conestat alfa (Ruconest®, Pharming Technologies B.V.) on renal and cerebral ischemic events in patients undergoing TAVI for severe symptomatic aortic stenosis (AS) compared to placebo.

Condition or disease	Intervention/treatment	Phase
Acute Ischemic Stroke<br>Acute Renal Injury	Drug: Conestat alfa (Ruconest®)<br>Drug: NaCl 0.9%)	Phase 2

Detailed Description:
Severe aortic stenosis (AS) is a frequent valvular heart disease in the elderly with a prevalence of 4 to 10% and a mean survival of only 0.5 to 5 years if left untreated. Transcatheter aortic valve implantation (TAVI) has evolved as standard of care for high-, intermediate and potentially even low surgical risk candidates due to a lower perioperative risk compared to surgical aortic valve replacement (SAVR). Despite its relative safety compared to SAVR, embolic events originating from the calcified valve and leading to ischemic stroke and acute renal injury are major complications following TAVI in the acute and subacute period, and are associated with increased morbidity including cognitive decline and mortality. While cerebral embolic protection devices (CEPD) such as the Sentinel® CEPD (Boston Scientific) were designed to reduce the burden of cerebral embolic events, their impact on clinical events has yet to be determined, and other prophylactic options are currently not available. Ischemia/reperfusion injury (IRI) is a key pathophysiological mechanism involved in cerebral and renal embolic events after TAVI resulting in activation of endothelial cells, the contact activation and the complement system and attraction of neutrophils to the site of injury. In this regard, recombinant human C1 esterase inhibitor (rhC1INH, conestat alfa), a potent inhibitor of the complement and the contact system has been shown to reduce the size of cerebral ischemic damage and of renal injury in experimental IRI models, and has successfully been investigated in a pilot study of acute kidney injury following the administration of contrast media. The aim of the current trial is to assess the safety and efficacy of conestat alfa (Ruconest®, Pharming Technologies B.V.) on renal and cerebral ischemic events in patients undergoing TAVI for severe symptomatic AS compared to placebo.

Study Design

Study Type : Interventional (Clinical Trial)
Estimated Enrollment : 250 participants
Allocation: Randomized
Intervention Model: Parallel Assignment
Intervention Model Description: Patients will be randomized in two parallel groups to receive either conestat alfa or placebo.
Masking: Double (Participant, Investigator)
Primary Purpose: Prevention
Official Title: Recombinant Human **C1 Esterase Inhibitor** (Conestat Alfa) in the Prevention of Acute Ischemic Cerebral and Renal Events After Transcatheter Aortic Valve Implantation: a Multi-center, Randomized, Double-blind, Placebo-controlled Investigational Study (PAIR-TAVI).
Actual Study Start Date : March 16, 2022
Estimated Primary Completion Date : March 2025
Estimated Study Completion Date : March 2025

Resource links provided by the National Library of Medicine

Drug Information available for: Conestat alfa SERPING1 protein, human

Genetic and Rare Diseases Information Center resources: Aortic Valve Stenosis Acute Graft Versus Host Disease

U.S. FDA Resources

Arms and Interventions

Arm	Intervention/treatment
Active Comparator: Conestat alfa (Ruconest®) intervention group<br>The intervention group will receive conestat alfa (Ruconest®) as a 10-minute slow intravenous injection (up to 56 ml) once during the TAVI procedure followed by a second administration (up to 28 ml) again three hours later. The first administration will include a dosage of 100 U/kg (maximum 8400 U) conestat alfa. The dosing of the second administration will be 50 U/kg (maximum 4200 U).	Drug: Conestat alfa (Ruconest®)<br>In the current study, participants will receive two intravenous injections of conestat alfa (immediately during the TAVI procedure and again 3h later) at a dose of 100 U/kg (first dose) and of 50 U/kg (subsequent dose), for patients less than 84 kg; two intravenous injections (immediately during the TAVI procedure and again 4h later) of conestat at a dose of 8400 U (4 vials, first dose) and of 4200 U (2 vials, subsequent dose) for patients of 84 kg body weight or greater. The chosen regimen including repeated administration should increase and maintain serum C1INH levels above twice the serum concentration for six to eight hours in the majority of patients. The timeframe of therapeutic concentrations will cover the period of the TAVI procedure itself and the immediate postprocedural period during which reperfusion and additional ischemic events related to global hypoperfusion may occur.
Placebo Comparator: saline injection placebo group<br>Subjects randomized into the placebo group will receive an intravenous normal saline injection with corresponding volume over 10 minutes during the TAVI procedure and three hours later after the first administration.	Drug: NaCl 0.9%)<br>Normal saline (NaCl 0.9%) will serve as placebo treatment. The respective amount of saline (according to patient weight matching the volume of conestat alfa that would have been used for this patient) will be withdrawn in an opaque syringe for slow IV injection.

Outcome Measures

Primary Outcome Measures :

1. Total volume of new cerebral ischemic lesions as evaluated by magnetic resonance imaging (MRI) [Time Frame: on day 4 (+/-1 day) after transfemoral TAVI]
   Total volume of new cerebral ischemic lesions as evaluated by magnetic resonance imaging (MRI)

Secondary Outcome Measures :

1. Maximum new lesion volume as measured by MRI (i.e. volume of the largest new lesion) [Time Frame: on day 4 (+/-1 day) after transfemoral TAVI]
   Maximum new lesion volume as measured by MRI (i.e. volume of the largest new lesion)
2. Number of new cerebral ischemic lesions as measured by MRI [Time Frame: on day 4 (+/-1 day) after transfemoral TAVI]
   Number of new cerebral ischemic lesions as measured by MRI
3. Number (incidence) of clinically manifest ischemic stroke [Time Frame: within 48 hours after TAVI]
   Number (incidence) of clinically manifest ischemic stroke
4. Change in secondary brain atrophy at 3-months follow-up [Time Frame: at baseline and at 3-months follow-up]
   Secondary brain atrophy at 3-months follow-up related to the gradual cellular loss as measured by high resolution 3D T1-weighted MR images (defined as the difference between the brain volumes)
5. Change in secondary infarct growth at 3-months follow-up (defined as the difference between the infarct volumes) [Time Frame: at day 4 and at 3-months]
   Change in secondary infarct growth at 3-months follow-up (defined as the difference between the infarct volumes)
6. Total brain damage (defined as the sum of secondary brain atrophy and final infarct volume) [Time Frame: at 3 months]
   Total brain damage (defined as the sum of secondary brain atrophy and final infarct volume)
7. Change in National Institutes of Health Stroke Scale Score (NIHSS) [Time Frame: at baseline and at 3-months follow-up]
   The NIHSS is composed of 11 items, each of which scores a specific ability between a 0 and 4. For each item, a score of 0 typically indicates normal function in that specific ability, while a higher score is indicative of some level of impairment. The individual scores from each item are summed in order to calculate a patient's total NIHSS score. The maximum possible score is 42, with the minimum score being a 0.
8. Change in modified Rankin scale [Time Frame: at baseline and at 3-months follow-up]
   Change in modified Rankin scale; scale runs from 0-6, running from perfect health (0) without symptoms to death (6)
9. Change in trail making test [Time Frame: at baseline and at 3-months follow-up]
   Change in trail making test; scoring is based on time taken to complete the test (e.g. 35 seconds yielding a score of 35) with lower scores being better.
10. Change in Montreal Cognitive Assessment test (MOCA) [Time Frame: at baseline and at 3-months follow-up]
    Montreal Cognitive Assessment test scores range between 0 and 30. A score of 26 or over is considered to be normal
11. Incidence of acute kidney injury (AKI) defined according to the Kidney Disease: Improving Global Outcomes criteria (any stage) [Time Frame: within 3 days after TAVI]
    Incidence of AKI defined according to the Kidney Disease: Improving Global Outcomes criteria (any stage)
12. Peak increase of urinary Neutrophil Gelatinase-Associated Lipocalin (NGAL) [Time Frame: within 48 hours after TAVI]
    Peak increase of urinary NGAL (surrogate marker of acute renal injury)
13. Incidence of significant increase in serum cystatin C (>10%) [Time Frame: within 48 hours after TAVI]
    Incidence of significant increase in serum cystatin C (>10%)

Other Outcome Measures:

1. Persistent renal impairment after 3 months (defined as increase in serum creatinine of at least 50% from baseline at 3 months) [Time Frame: at 3-months follow-up]
   Persistent renal impairment after 3 months (defined as serum creatinine increase of at least 50% from baseline at 3 months)
2. Change in concentration of **C1-Esterase-Inhibitor** (**C1**INH) [Time Frame: during the first 24 hours after TAVI]
   Change in concentration of **C1**INH
3. Change in troponin T to assess myocardial injury following TAVI [Time Frame: within 72 hours after TAVI]
   Change in troponin T to assess myocardial injury following TAVI
4. Change in urinary biomarkers of renal injury (Kidney Injury Molecule-1 (KIM-1) and osteopontin) [Time Frame: within 48 hours after TAVI]
   Change in urinary biomarkers of renal injury (Kidney Injury Molecule-1 (KIM-1) and osteopontin)
5. Change in serum neurofilament light chain (marker of neuroaxonal damage) [Time Frame: within 3 months after TAVI]
   Change in serum neurofilament light chain (marker of neuroaxonal damage)
6. Number of adverse events [Time Frame: within 3 months after TAVI]
   Number of adverse events
7. Number of serious adverse events [Time Frame: within 3 months after TAVI]
   Number of serious adverse events
8. Number of major cardiovascular and renal events (cardiovascular death, non-fatal myocardial infarction, heart failure hospitalization, stroke, dialysis) [Time Frame: within 3 months after TAVI]
   Number of major cardiovascular and renal events (cardiovascular death, non-fatal myocardial infarction, heart failure hospitalization, stroke, dialysis)
9. Number of complications of transfemoral TAVI [Time Frame: within 3 months after TAVI]
   Number of complications of transfemoral TAVI such as conduction disturbance (including permanent pacemaker implantation) or aortic regurgitation according to the Valve Academic Research Consortium (VARC)-3 criteria, or bleeding according to the Bleeding Academic Research Consortium (BARC)-criteria)

Eligibility Criteria

Information from the National Library of Medicine

Choosing to participate in a study is an important personal decision. Talk with your doctor and family members or friends about deciding to join a study. To learn more about this study, you or your doctor may contact the study research staff using the contacts provided below. For general information, Learn About Clinical Studies.

Ages Eligible for Study: 18 Years and older (Adult, Older Adult)
Sexes Eligible for Study: All
Accepts Healthy Volunteers: No

Criteria

Inclusion Criteria:

- Informed consent as documented by signature
- Severe AS and scheduled for transfemoral TAVI

Exclusion Criteria:

- Contraindications to the class of drugs under study (C1INH), e.g., known hypersensitivity or allergy to class of drugs or the investigational product
- History of allergy to rabbits (as rhC1INH is derived from the breast milk of transgenic rabbits)
- Women who are pregnant or breast feeding
- Hemodynamic instability requiring emergency TAVI
- Valve-in-valve procedure
- Other access route than transfemoral
- Non-cardiac co-morbidity with expected survival <6 months
- Ischemic or hemorrhagic stroke within 30 days before TAVI
- Dialysis or estimated glomerular filtration rate (eGFR) <20 ml/min/1.73m2
- Contraindication for MRI such as a permanent non-MRI compatible pacemaker or severe claustrophobia
- Liver cirrhosis (any Child-Pugh score)
- Incapacity or inability to provide informed consent
- Participation in another study with investigational drug or medical device within the 30 days preceding and during the present study
- Previous enrolment into the current study
- Any uncontrolled or significant concurrent illness that would put the patient at a greater risk or limit compliance with the study requirements at the discretion of the investigator

Contacts and Locations

Information from the National Library of Medicine

To learn more about this study, you or your doctor may contact the study research staff using the contact information provided by the sponsor.

Please refer to this study by its ClinicalTrials.gov identifier (NCT number): ***NCT05145283***

Contacts

Contact: Michael Osthoff, Prof. Dr. med. +41 61 328 54 20 michael.osthoff@usb.ch
Contact: Stephan Moser, Dr. med. +41 61 265 25 25 stephan.moser@usb.ch

Locations

Switzerland

University Hospital Basel, Division of Internal Medicine — **Recruiting**
Basel, Switzerland, 4031
Contact: Michael Osthoff, Prof Dr. med. +41 61 328 54 20 michael.osthoff@usb.ch
Contact: Stephan Moser, Dr.med. +41 61 265 25 25 stephan.moser@usb.ch
Principal Investigator: Michael Osthoff, Prof Dr. med.

Stadtspital Triemli Zürich, Division of Cardiology — **Recruiting**
Zürich, Switzerland, 8063
Contact: Raban Jeger, Prof. Dr. med. +41 44 416 34 11 raban.jeger@stadtspital.ch
Principal Investigator: Raban Jeger, Prof. Dr. med.

Sponsors and Collaborators

University Hospital, Basel, Switzerland
Swiss National Science Foundation
Pharming Technologies B.V.

Investigators

Principal Investigator: Michael Osthoff, Prof. Dr. med. University Hospital Basel, Division of Internal Medicine
Principal Investigator: Raban Jeger, Prof. Dr. med. Stadtspital Triemli Zürich, Division of Cardiology

More Information

Responsible Party: University Hospital, Basel, Switzerland
ClinicalTrials.gov Identifier: NCT05145283 History of Changes
Other Study ID Numbers: 2021-02025; me19Osthoff
First Posted: December 6, 2021 Key Record Dates
Last Update Posted: February 2, 2023
Last Verified: February 2023

Studies a U.S. FDA-regulated Drug Product: No
Studies a U.S. FDA-regulated Device Product: No

Keywords provided by University Hospital, Basel, Switzerland:
Severe aortic stenosis (AS)
Valvular heart disease
Transcatheter aortic valve implantation (TAVI)
cerebral embolic events
renal embolic events
Ischemia/reperfusion injury (IRI)
recombinant human **C1 esterase inhibitor** (rhC1INH, conestat alfa)
complement system
contact activation system

Additional relevant MeSH terms:
Complement **C1 Inhibitor** Protein
Ischemic Stroke
Acute Kidney Injury
Ischemia
Pathologic Processes
Stroke
Cerebrovascular Disorders
Brain Diseases
Central Nervous System Diseases
Nervous System Diseases
Vascular Diseases
Cardiovascular Diseases
Renal Insufficiency
Kidney Diseases
Urologic Diseases
Female Urogenital Diseases
Female Urogenital Diseases and Pregnancy Complications
Urogenital Diseases
Male Urogenital Diseases
Complement Inactivating Agents
Immunosuppressive Agents
Immunologic Factors
Physiological Effects of Drugs

A Gene Therapy Study of BMN 331 in Subjects With Hereditary Angioedema (HAErmony-1)

The safety and scientific validity of this study is the responsibility of the study sponsor and investigators. Listing a study does not mean it has been evaluated by the U.S. Federal Government. Know the risks and potential benefits of clinical studies and talk to your health care provider before participating. Read our disclaimer for details.

ClinicalTrials.gov Identifier: NCT05121376

Recruitment Status : Recruiting
First Posted : November 16, 2021
Last Update Posted : April 11, 2023

See **Contacts and Locations**

View this study on the modernized ClinicalTrials.gov

Sponsor:
BioMarin Pharmaceutical

Information provided by (Responsible Party):
BioMarin Pharmaceutical

Study Details | **Tabular View** | **No Results Posted** | Disclaimer | How to Read a Study Record

Study Description

Brief Summary:

This is a Phase 1/2, single-arm, open-label, dose-escalation and dose-expansion study of BMN 331 for the treatment of hereditary angioedema (HAE) due to C1 Esterase Inhibitor (C1-INH) protein deficiency. The study drug BMN 331is identified as AAV5 hSERPING1, an adeno-associated virus (AAV5)-based gene therapy vector that expresses wild-type human C1 Esterase Inhibitor (hC1-INH), under the control of a liver-selective promoter, and is being developed for the treatment of HAE with C1-INH deficiency. The pharmaceutical form of BMN 331 is a solution for intravenous infusion.

Condition or disease	Intervention/treatment	Phase
Hereditary Angioedema<br>HAE	Genetic: Dose 1 of BMN 331<br>Genetic: Dose 2 of BMN 331<br>Genetic: Dose 3 of BMN 331<br>Genetic: Dose 4 of BMN 331<br>Genetic: Dose 5 of BMN 331	Phase 1<br>Phase 2

Detailed Description:

BMN 331 is an investigational, single administration gene therapy intended to modify the disease course of HAE. Preclinical studies have shown that BMN 331 can transduce hepatocytes resulting in restoration of the deficient circulating levels of hC1-INH that cause HAE.

Study 331-201 is a two-part (part A and part B), first-in-human, Phase 1/2 study designed to assess the safety and efficacy of BMN 331 in patients with HAE. Subjects will be followed for 5 years following BMN 331 infusion. Part A of the study is a dose escalation phase designed to assess the preliminary safety of a single IV administration of BMN 331 and to determine whether there is a dose-dependent increase in C1-INH protein expression following administration of BMN 331. Part B is a dose expansion phase designed to demonstrate that up to three safe doses of BMN 331 (as determined in Part A) sustains a clinically meaningful increase in C1-INH levels.

Study Design

Study Type : Interventional (Clinical Trial)
Estimated Enrollment : 34 participants
Allocation: N/A
Intervention Model: Sequential Assignment
Masking: None (Open Label)
Primary Purpose: Treatment
Official Title: A Phase 1/2 Open-Label, Dose-Escalation Study to Determine the Safety Tolerability & Efficacy of BMN 331 an AAV Vector-Mediated Gene Transfer of Human SERPING1 Gene in Subjects With HAE Due to Human C1-INH Deficiency
Actual Study Start Date : February 15, 2022
Estimated Primary Completion Date : November 2028
Estimated Study Completion Date : November 2028

Resource links provided by the National Library of Medicine

NIH NLM

MedlinePlus Genetics related topics: Hereditary angioedema

MedlinePlus related topics: Genes and Gene Therapy

Genetic and Rare Diseases Information Center resources: Hereditary Angioedema

U.S. FDA Resources

Arms and Interventions

Arm	Intervention/treatment
Experimental: BMN 331<br>AAV Gene Therapy Infusion	Genetic: Dose 1 of BMN 331<br>BMN 331 AAV Gene Therapy<br><br>Genetic: Dose 2 of BMN 331<br>BMN 331 AAV Gene Therapy<br><br>Genetic: Dose 3 of BMN 331<br>BMN 331 AAV Gene Therapy<br><br>Genetic: Dose 4 of BMN 331<br>BMN 331 AAV Gene Therapy<br><br>Genetic: Dose 5 of BMN 331<br>BMN 331 AAV Gene Therapy

Outcome Measures

Primary Outcome Measures :

1. Number of participants with treatment-emergent adverse events following a single IV administration of BMN 331 [Time Frame: At 5 years]

Secondary Outcome Measures :

1. Number and severity of investigator-confirmed HAE attacks [Time Frame: At 5 years]
2. Annualized use of HAE medication [Time Frame: At 5 years]
3. Plasma levels of functional C1-INH following BMN-331 infusion [Time Frame: At 5 years]
4. Plasma levels of C1-INH antigen following BMN 331 infusion [Time Frame: At 5 years]
5. Levels of total antibodies against AAV5 capsid following BMN 331 infusion [Time Frame: At 5 years]
6. Levels of total antibodies against C1-INH following BMN 331 infusion [Time Frame: At 5 years]
7. Levels of neutralizing antibodies against C1-INH following BMN 331 infusion [Time Frame: At 5 years]

Eligibility Criteria

Information from the National Library of Medicine

NIH NLM

Choosing to participate in a study is an important personal decision. Talk with your doctor and family members or friends about deciding to join a study. To learn more about this study, you or your doctor may contact the study research staff using the contacts provided below. For general information, Learn About Clinical Studies.

Ages Eligible for Study: 18 Years and older (Adult, Older Adult)
Sexes Eligible for Study: All
Accepts Healthy Volunteers: No

Criteria

Inclusion Criteria:

1. Female or male adults (≥ 18 years old)
2. Confirmed diagnosis of HAE due to C1-INH deficiency (Type I or II) confirmed by genotyping of SERPING1 gene
3. Currently using an HAE medication regimen that consists of a routine long-term prophylactic treatment for at least 6 months prior to enrollment or an on-demand therapy regimen for a documented attack frequency of at least 4 attacks within the last 12 months prior to enrollment or at least 2 attacks within the last 6 months prior to enrollment
4. Trained in self-administering acute attack treatment and is able to adequately manage acute attacks in a home setting
5. Willingness to abstain from consumption of alcohol for at least 52 weeks post BMN 331 infusion and to use highly effective contraception

Exclusion Criteria:

1. Evidence of active or chronic infection, including severe acute respiratory syndrome coronavirus 2 (SARS-CoV-2), or any immunosuppressive disorder
2. Contraindication to using glucocorticosteroids GCS, including a diagnosis of glaucoma or untreated osteoporosis
3. Active malignancy (except non-melanoma skin cancer) autoimmune, metabolic (i.e., diabetes), hematologic, cardiac, or renal disease that is of clinical significance defined as requiring regular medical attention and treatment
4. Prior gene therapy treatment
5. Prior use of high-dose attenuated androgens in the last 1 year prior to the study
6. History or current clinically relevant liver disease (eg, nonalcoholic steatohepatitis [NASH], or chronic viral hepatitis B or C [HBV or HCV] or autoimmune hepatitis)
7. Have a history or are at risk for clinically significant thromboembolic events (TEE) , or known underlying risk factor for thrombosis including thrombotic microangiopathy (TMA)

Contacts and Locations

Information from the National Library of Medicine

NIH NLM

To learn more about this study, you or your doctor may contact the study research staff using the contact information provided by the sponsor.

Please refer to this study by its ClinicalTrials.gov identifier (NCT number): ***NCT05121376***

Contacts

Contact: Trial Specialist 1-800-983-4587 medinfo@bmrn.com

Locations

United States, Alabama

AllerVie Clinical Research — **Recruiting**
Birmingham, Alabama, United States, 35209
Contact: Esther Lange, CRNP 205-209-4131 elange@allervie.com
Principal Investigator: John Anderson, MD

United States, California

University of California San Diego — **Recruiting**
San Diego, California, United States, 92122
Contact: Roxanne Lacy 858-657-5366 rlacy@health.ucsd.edu
Principal Investigator: Marc Riedl, MD MS

United States, Kansas

Dr. Henry J. Kanarek Allergy, Asthma & Immunology — **Recruiting**
Overland Park, Kansas, United States, 66211
Contact: Kristin Fravel 913-451-8555 kristinkresearch@gmail.com.com
Principal Investigator: Henry Kanarek, MD

United States, Maryland

Institute For Asthma & Allergy — **Recruiting**
Chevy Chase, Maryland, United States, 20815
Contact: Amie Koroma 301-986-9262 iaaresearchkoroma@gmail.com
Principal Investigator: Henry Li, MD

United States, Mississippi

Mississippi Center for Advanced Medicine — **Recruiting**
Madison, Mississippi, United States, 39110
Contact: Jesus Monico jmonico@msadvancedmedicine.com
Principal Investigator: Ray Rodriguez, MD

United States, Missouri

Washington University School of Medicine — **Recruiting**
Saint Louis, Missouri, United States, 63141
Contact: Tarisa Mantia 314-996-8339 tmantia@wustl.edu
Principal Investigator: James H Wedner, MD

United States, North Carolina

Duke Health — **Recruiting**
Durham, North Carolina, United States, 27710
Contact: Katherine Prince 919-681-8931 katherine.prince@duke.edu
Principal Investigator: Patricia Lugar, MD

United States, Ohio

University of Cincinnati (UC) Physicians Company, LLC — **Recruiting**
Cincinnati, Ohio, United States, 45267
Contact: Heather Kimbrough 513-558-6987 kimbrohb@ucmail.uc.edu
Principal Investigator: Jonathan Bernstein, MD

Optimed Research, LTD — **Recruiting**
Columbus, Ohio, United States, 43235
Contact: Katie Suchy 614-505-1967 katie@optimedresearch.com
Principal Investigator: Donald McNeil, MD

United States, Pennsylvania

The Pennsylvania State University (Penn State) Milton S. Hershey Medical Center — **Recruiting**
Hershey, Pennsylvania, United States, 16802
Contact: Kristina Richwine 717-531-4506 krichwine@pennstatehealth.psu.edu
Principal Investigator: Timothy Craig, MD

United States, Texas

AARA Research Center — **Recruiting**
Dallas, Texas, United States, 75231
Contact: Taufiq Sayibu 214-365-0365 SayibuMD@aararesearch.com
Principal Investigator: William Lumry, MD

Spain

Hospital Universitario Vall d'Hebron — **Recruiting**
Barcelona, Spain
Contact: Laura Company Sapina 617908767 laura.company@vhir.org
Principal Investigator: Mar Guilarte, MD PhD

Hospital Universitario La Paz — **Recruiting**
Madrid, Spain
Contact: Olga Roche 34 91 727 77 07 oroche@gmail.com
Principal Investigator: Teresa Caballero, MD, PhD

Sponsors and Collaborators

BioMarin Pharmaceutical

Investigators

Study Director: MD Medical Director BioMarin Pharmaceutical

More Information

Responsible Party: BioMarin Pharmaceutical
ClinicalTrials.gov Identifier: NCT05121376 History of Changes
Other Study ID Numbers: 331-201
First Posted: November 16, 2021 Key Record Dates
Last Update Posted: April 11, 2023
Last Verified: April 2023

Studies a U.S. FDA-regulated Drug Product: Yes
Studies a U.S. FDA-regulated Device Product: No

Keywords provided by BioMarin Pharmaceutical:

HAE	331-201
Hereditary Angioedema	331
Gene Therapy	Haermony

Additional relevant MeSH terms:

Angioedema	Hypersensitivity, Immediate
Angioedemas, Hereditary	Hypersensitivity
Vascular Diseases	Immune System Diseases
Cardiovascular Diseases	Hereditary Complement Deficiency Diseases
Urticaria	Primary Immunodeficiency Diseases
Skin Diseases, Vascular	Genetic Diseases, Inborn
Skin Diseases	Immunologic Deficiency Syndromes

An official website of the United States government Here's how you know

API users: Read about imminent changes impacting usage of the ClinicalTrials.gov API. (Return to the classic website)

NIH National Library of Medicine
National Center for Biotechnology Information

PRS Login

ClinicalTrials.gov

About This Site | Find Studies | Data About Studies | Study Basics | PRS Info

The U.S. government does not review or approve the safety and science of all studies listed on this website.
Read our full disclaimer for details.

NOT YET RECRUITING

Prevention of Intradialytic Hypotension by Inhibiting Bradykinin B2 Receptor

ClinicalTrials.gov ID NCT05834777
Sponsor Vanderbilt University Medical Center
Information provided by Jorge Gamboa, Vanderbilt University Medical Center (Responsible Party)
Last Update Posted 2023-08-01

Study record dates

Study Details | Table View | No Results Posted | Record History

On this page
- Study Overview
- Contacts and Locations
- Participation Criteria
- Study Plan
- Collaborators and Investigators
- Publications
- More Information

Download | Expand all content | Collapse all content

Study Overview

Brief Summary:
Currently, there is no medication available to adequately treat patients undergoing hemodialysis who are suffering from intradialytic hypotension (IDH). Medical interventions such as Trendelenburg positioning, saline bolus administration, reduction of ultrafiltration rate, interruption of the hemodialysis, and other medical treatments are the methods of choice to treat the hypotensive condition of these patients and thus to maintain the systolic blood pressure. Patients suffering from IDH have a higher reported mortality rate due to the given stress on their cardiovascular system. New treatments, therefore, would give clinicians an additional alternative to current existing approaches and might help patients to maintain their blood pressure during hemodialysis.

The main objective of the study is to evaluate the efficacy of icatibant in the prevention of systolic blood pressure (SBP) drop in patients on hemodialysis suffering from recurrent IDH episodes during hemodialysis.

— Show less

Detailed Description:
Aim 1 is to test the hypothesis that in patients prone to IDH, blockade of bradykinin B2 receptor with icatibant prevents the drop of blood pressure and maintains hemodynamic stability. For this purpose, heart rate and blood pressure during hemodialysis are monitored. Importantly, the study will be conducted in an outpatient clinic, using the patients' usual hemodialysis dose and settings as for a regular hemodialysis session.

Aim 2 is to test the hypothesis that in patients prone to IDH, inhibition of the plasma kallikrein system with icatibant prevents symptoms associated with IDH such as cramps, dizziness, and nausea, and improves the quality of life (QoL) and recovery time after hemodialysis. Hemodialysis is associated with many complications, including IDH, that negatively affect the QoL of patients and their families. Any intervention that prevents the occurrence of IDH will result in a faster recovery from hemodialysis. The present study will evaluate the impact of icatibant on preventing symptoms associated with IDH and reducing recovery time after hemodialysis.

— Show less

OFFICIAL TITLE
Prevention of Intradialytic Hypotension by Inhibiting Bradykinin B2 Receptor A Randomized, Double-blind, Placebo-controlled, Cross Over Explorative Study

CONDITIONS
Intradialytic Hypotension

INTERVENTION / TREATMENT
Drug: Icatibant
Drug: Placebo

STUDY START (ESTIMATED)
2023-09-01

PRIMARY COMPLETION (ESTIMATED)
2025-09-01

STUDY COMPLETION (ESTIMATED)
2025-12-01

ENROLLMENT (ESTIMATED)
26

STUDY TYPE
Interventional

PHASE
Phase 3

OTHER STUDY ID NUMBERS
222301

Resource links provided by the National Library of Medicine NIH NLM

MedlinePlus related topics: Dialysis Low Blood Pressure

Genetic and Rare Diseases Information Center resources: Oculocerebral Syndrome With Hypopigmentation

Drug Information available for: Icatibant Icatibant acetate

Other U.S. FDA Resources

Contacts and Locations

This section provides the contact details for those conducting the study, and information on where this study is being conducted.

STUDY CONTACT
Name: Delia M Woods, BSN/MSL
Phone Number:
615-327-7181
Email: delia.woods@vumc.org

No location data

Participation Criteria

Researchers look for people who fit a certain description, called eligibility criteria. Some examples of these criteria are a person's general health condition or prior treatments.

For general information about clinical research, read Learn About Studies.

Eligibility Criteria

DESCRIPTION

Inclusion Criteria:

- Patients ≥ 18 to ≤ 80 years of age
- Patients with end-stage renal disease on hemodialysis (including hemodiafiltration) for at least 6 months, receiving 3 dialysis sessions per week and who are in a stable clinical condition per investigator's judgement
- Patients on hemodialysis with at least 6 IDH episodes during the last 8 weeks based on medical record assessment
- Pre-dialysis systolic blood pressure ≥ 110 and ≤ 170 mmHg assessed by two consecutive and averaged pre-HD blood pressure measurments
- Patients adequately hemodialyzed with a Kt/V ≥ 1.2
- Patients whose treatment regimen remained unchanged within 14 days prior to dosing (diet, medication, dry weight, treatment time, dialysate composition and temperature, dialysis shift, blood flow, and dialysate flow, vascular access)
- Female subjects < 55 years of age to agree on effective contraception methods throughout the study period and who have a negative pregnancy test before initiating study activities
- Body weight ≤ 150 kg

Exclusion Criteria:

- Patients who have been hospitalized during the last 4 weeks before enrolment, except vascular access related hospitalization
- Patients with known clinically evident inflammatory or infectious disease per investigator's evaluation
- Severe anemia with a hemoglobin (Hb) < 8.0 g/dL at screening
- Platelet count < 50 x 109/L
- Hepatic disease associated with ALT > 3x ULN, or total bilirubin >2x ULN with direct bilirubin > 20% of the total bilirubin level
- Known bleeding disorders e.g., von-Willebrand disease or Hemophilia A, B, C, etc.
- Recent (<3 months before screening) thromboembolic event, e.g., acute coronary syndrome, stroke, or venous thrombosis embolism (except dialysis access thrombosis)
- Recent (<3 months before screening) major surgery or scheduled major surgery during study participation
- Scheduled living donor renal transplant during study participation
- Persistent heart failure as classified by the New York Heart Association (NYHA) classification of 3 or higher
- Receiving antiplatelet therapy except daily ASA ≤ 150 mg/day
- Receiving anticoagulation in therapeutic doses, other than standard anticoagulation during the hemodialysis procedure
- Patients with significant pre-dialysis overload as defined by > 5kg above dry weight estimated by bioimpedance spectroscopy
- Patient's life expectancy < 6 months per investigator's judgement

— Show less

AGES ELIGIBLE FOR STUDY
18 Years to 80 Years (Adult, Older Adult)

SEXES ELIGIBLE FOR STUDY
All

ACCEPTS HEALTHY VOLUNTEERS
No

Study Plan

This section provides details of the study plan, including how the study is designed and what the study is measuring.

Expand all / Collapse all

How is the study designed?

DESIGN DETAILS
Primary Purpose: Health Services Research
Allocation: Randomized
Interventional Model: Crossover Assignment
Interventional Model Description:
Randomized, placebo-controlled, cross-over study, with a pharmocokinetics evaluation phase.

Study duration for main cross-over study: Approximately 60 days including screening evaluation activities.
Masking: Quadruple (Participant, Care Provider, Investigator, Outcomes Assessor)
Masking Description: For the procedure of the cross-over main study, both participating sites will recruit patients that will be randomized through a centralized randomization key to ensuring an equally balanced allocation of patients to the respective cross-over groups (icatibant or placebo). All study personnel will be blinded except the IDS pharmacy.

ARMS AND INTERVENTIONS

Participant Group/Arm	Intervention/Treatment
Experimental: Icatibant<br>153 mL 0.9% saline bag containing 30 mg icatibant acetate (10 mg/ml) IV at each dialysis treatment for four weeks (12 hemodialysis sessions)	Drug: Icatibant<br>• Icatibant will be administered with an infusion rate of 100μg/kg/h for 15 minutes before their hemodialysis session (pre-infusion) and 50μg/kg/h during their routine hemodialysis session (maintenance infusion).
Placebo Comparator: Placebo<br>153 mL 0.9% saline bag IV at each dialysis treatment at each dialysis treatment for four weeks (12 hemodialysis sessions)	Drug: Placebo<br>• 0.9% sodium chloride will be administered as the same rate as icatibant

What is the study measuring?

PRIMARY OUTCOME MEASURES

Outcome Measure	Measure Description	Time Frame
Blood pressure during hemodialysis	Average systolic blood pressure	0-8 weeks

SECONDARY OUTCOME MEASURES

Outcome Measure	Measure Description	Time Frame
Hypotension events	We will record the number of hypotensive events during hemodialysis. We expect a decrease of event in the treatment arm compared to placebo	0-8 week
Symptomatology associated with hypotension	At the end of each hemodialysis session in the cross-over main study, the presence of any symptoms associated with hypotension using a standardized questionnaire will be assessed. This questionnaire will include symptoms such as cramps, dizziness, nausea and fatigue rating, and will be scored according to the severity from 0 to 4 (none to severe) for the first listed symptoms, and according to the SONG-HD Fatigue scoring for the last listed symptom, respectively.	0-8 weeks

Collaborators and Investigators

This is where you will find people and organizations involved with this study.

SPONSOR
Vanderbilt University Medical Center

COLLABORATORS
- Pharvaris Netherlands B.V.
- Renal Research Institute

INVESTIGATORS
- Principal Investigator: Jorge L Gamboa, MD/PhD, Vanderbilt University Medical Center
- Principal Investigator: Peter Kotanko, MD, FASN, Renal Research Institute
- Principal Investigator: Talat A Ikizler, MD, Vanderbilt University Medical Center

Publications

The person responsible for entering information about the study voluntarily provides these publications. These may be about anything related to the study.

GENERAL PUBLICATIONS
No publications available

*** Find Publications about Study Results and related Pubmed Publications in the "Results" section of the study record.**

More Information

Record History

Expand all / Collapse all

Terms related to this study

KEYWORDS PROVIDED BY JORGE GAMBOA, VANDERBILT UNIVERSITY MEDICAL CENTER
Hemodialysis
Intradialytic hypotension
Bradykinin
Kallikrein-Kinin System
Icatibant

ADDITIONAL RELEVANT MeSH TERMS
Vascular Diseases
Cardiovascular Diseases
Hypotension
Anti-Inflammatory Agents, Non-Steroidal
Analgesics, Non-Narcotic
Analgesics
Sensory System Agents
Peripheral Nervous System Agents
Physiological Effects of Drugs
Anti-Inflammatory Agents
Antirheumatic Agents
Bradykinin B2 Receptor Antagonists
Bradykinin Receptor Antagonists
Molecular Mechanisms of Pharmacological Action
Complement Inactivating Agents
Immunosuppressive Agents
Immunologic Factors
Icatibant

Plan for Individual participant data (IPD)

PLAN TO SHARE INDIVIDUAL PARTICIPANT DATA (IPD)?
No

Drug and device information, study documents, and helpful links

STUDIES A U.S. FDA-REGULATED DRUG PRODUCT
Yes

STUDIES A U.S. FDA-REGULATED DEVICE PRODUCT
No

PRODUCT MANUFACTURED IN AND EXPORTED FROM THE U.S.
No

STUDY DOCUMENTS
No study documents available

About
About ClinicalTrials.gov
Release Notes

Help
Give us feedback
Glossary
Customer Support

Legal
Disclaimer
Terms and Conditions

ClinicalTrials.gov
National Library of Medicine
8600 Rockville Pike, Bethesda, MD 20894

NIH National Library of Medicine
National Center for Biotechnology Information

ClinicalTrials.gov
An official website of the U.S. Department of Health and Human Services, National Institutes of Health, National Library of Medicine, and National Center for Biotechnology Information.

About HHS | About NCBI | No FEAR Act data | HHS Vulnerability Disclosure
About NIH | Accessibility support | Office of the Inspector General
About NLM | FOIA requests | Privacy policy

Looking for U.S. government information and services? Visit USA.gov

Feedback

Revision: v2.1.1

Release Details

Pharvaris Announces FDA Removal of Clinical Hold of Deucrictibant for the On-Demand Treatment of HAE

June 26, 2023

PDF Version

ZUG, Switzerland, June 26, 2023 (GLOBE NEWSWIRE) -- Pharvaris (Nasdaq: PHVS), a clinical-stage company developing novel, oral bradykinin-B2-receptor antagonists to treat and prevent hereditary angioedema (HAE) attacks, today announced the U.S. Food and Drug Administration (FDA) has lifted the clinical hold on the Investigational New Drug (IND) application for deucrictibant for the on-demand treatment of HAE following review of data from a preplanned interim analysis of the ongoing 26-week nonclinical study. Pharvaris expects to submit data from the 26-week nonclinical study by the end of 2023 to address the remaining hold on the IND application for deucrictibant for prophylactic treatment of HAE.

"The lift of the clinical hold on our on-demand clinical trials enables us to continue development of PHVS416 (deucrictibant immediate-release capsules) in the U.S., including resuming RAPIDe-2, our extension study for acute treatment of attacks," said Peng Lu, M.D., Ph.D., Chief Medical Officer of Pharvaris. "Our team plans to request an end of Phase 2 meeting with the agency and is preparing for RAPIDe-3, our global Phase 3 study of PHVS416 for the on-demand treatment of HAE, to include U.S. sites. The 26-week nonclinical study to address the remaining hold on prophylactic deucrictibant in the U.S. is still progressing and we plan to submit the data from that study to the FDA by the end of the year. Based on current enrollment, we confirm that top-line data from CHAPTER-1, our Phase 2 proof-of-concept study of PHVS416 for the prophylactic treatment of HAE, remains on track to be announced by the end of the year."

About Pharvaris
Building on its deep-seated roots in hereditary angioedema (HAE), Pharvaris is a clinical-stage company developing novel, oral bradykinin-B2-receptor antagonists to treat and prevent HAE attacks. By directly targeting this clinically proven therapeutic target with novel small molecules, the Pharvaris team aspires to offer people with all sub-types of HAE safe, effective, and convenient alternatives to treat attacks, both on-demand and prophylactically. The company brings together the best talent in the industry with deep expertise in rare diseases and HAE. For more information, visit https://pharvaris.com/.

Forward-Looking Statements
This press release contains certain forward-looking statements that involve substantial risks and uncertainties. All statements contained in this press release that do not relate to matters of historical fact should be considered forward-looking statements, including, without limitation, statements relating to the Offering and the use of proceeds therefrom, and any statements containing the words "believe," "anticipate," "expect," "estimate," "may," "could," "should," "would," "will," "intend" and similar expressions. These forward-looking statements are based on management's current expectations, are neither promises nor guarantees, and involve known and unknown risks, uncertainties and other important factors that may cause Pharvaris' actual results, performance or achievements to be materially different from its expectations expressed or implied by the forward-looking statements. Such risks include but are not limited to the following: uncertainty in the outcome of our interactions with regulatory authorities, including the FDA with respect to the clinical hold on prophylactic deucrictibant in the U.S.; the expected timing, progress, or success of our clinical development programs, especially for PHVS416 (immediate-release deucrictibant capsules) and PHVS719 (extended-release deucrictibant tablets), which are in mid-stage global clinical trials; risks associated with the COVID-19 pandemic, which may adversely impact our business, nonclinical studies, and clinical trials; the timing of regulatory approvals; the value of our ordinary shares; the timing, costs and other limitations involved in obtaining regulatory approval for our product candidates PHVS416 and PHVS719, or any other product candidate that we may develop in the future; our ability to establish commercial capabilities or enter into agreements with third parties to market, sell, and distribute our product candidates; our ability to compete in the pharmaceutical industry and with competitive generic products; our ability to market, commercialize and achieve market acceptance for our product candidates; our ability to raise capital when needed and on acceptable terms; regulatory developments in the United States, the European Union and other jurisdictions; our ability to protect our intellectual property and know-how and operate our business without infringing the intellectual property rights or regulatory exclusivity of others; our ability to manage negative consequences from changes in applicable laws and regulations, including tax laws, our ability to successfully remediate the material weakness in our internal control over financial reporting and to maintain an effective system of internal control over financial reporting; changes in general market, political and economic conditions, including as a result of the current conflict between Russia and Ukraine; and the other factors described under the headings "Cautionary Statement Regarding Forward-Looking Statements" and "Item 3. Key Information—D. Risk Factors" in our Annual Report on Form 20-F and other periodic filings with the U.S. Securities and Exchange Commission. These and other important factors could cause actual results to differ materially from those indicated by the forward-looking statements made in this press release. Any such forward-looking statements represent management's estimates as of the date of this press release. New risks and uncertainties may emerge from time to time, and it is not possible to predict all risks and uncertainties. While Pharvaris may elect to update such forward-looking statements at some point in the future, Pharvaris disclaims any obligation to do so, even if subsequent events cause its views to change. These forward-looking statements should not be relied upon as representing Pharvaris' views as of any date subsequent to the date of this press release.

Contact Maggie Beller Head of Public Relations and Communications maggie.beller@pharvaris.com

©2023 Pharvaris N.V. © All Rights Reserved

This is the classic website, which will be retired eventually. Please visit the modernized ClinicalTrials.gov instead.

Fasitibant Intra-articular Injection in Patients With Symptomatic Osteoarthritis of the Knee (ALBATROSS-3)

The safety and scientific validity of this study is the responsibility of the study sponsor and investigators. Listing a study does not mean it has been evaluated by the U.S. Federal Government. Read our disclaimer for details.

ClinicalTrials.gov Identifier: NCT02205814

Recruitment Status : Completed
First Posted : July 31, 2014
Results First Posted : September 16, 2015
Last Update Posted : November 3, 2015

View this study on the modernized ClinicalTrials.gov

Sponsor:
Menarini Group

Information provided by (Responsible Party):
Menarini Group

Study Description

Go to

Brief Summary:

This study is designed as a double-blind, randomised, placebo-controlled, four parallel arm, dose-finding study, to be conducted in approximately 26 sites, to evaluate the efficacy, safety, tolerability, and pharmacokinetics of single **intra-articular** (IA) **injections** of **fasitibant** in **patients with symptomatic osteoarthritis** (OA) of the **knee**.

Approximately 400 male and female **patients** 40-80 years old, with BMI < 30 kg/m² and with a clinical diagnosis of **symptomatic** primary **osteoarthritis of the knee** will be randomised to a total of 4 treatment arms. Each arm includes a single **intra-articular injection** of one of three dosages of **fasitibant** (low, intermediate and high dose) OR placebo. The randomisation ratio will be 1:1:1:1.

The primary efficacy variable will be the change of the Western Ontario and McMaster Universities Visual Analogue Scale 3.1 A (WOMAC VA 3.1 A) (total pain) subscore from baseline up to 2 weeks after randomisation. Safety will be assessed by monitoring adverse events and clinical laboratory tests; local tolerability at the **injection** site will also be assessed. In addition, the population pharmacokinetics and the exposure-response relationship will be evaluated.

The individual experimental clinical phase will last up to maximal 15 weeks encompassing 7 planned visits at site, including screening, randomisation, 4 follow-up visits and the End of study visit.

Condition or disease	Intervention/treatment	Phase
**Knee Osteoarthritis**	Drug: **Fasitibant-** low dose<br>Drug: **Fasitibant-** intermediate dose<br>Drug: **Fasitibant-** high dose<br>Drug: Placebo comparator	Phase 2

Study Design

Go to

Study Type : Interventional (Clinical Trial)
Actual Enrollment : 436 participants
Allocation: Randomized
Intervention Model: Parallel Assignment
Masking: Quadruple (Participant, Care Provider, Investigator, Outcomes Assessor)
Primary Purpose: Treatment
Official Title: A Double-blind, Randomised, Placebo-controlled, Four Parallel Arm, Dose-finding Study to Evaluate the Efficacy, Safety, Tolerability, and Pharmacokinetics of Single **Intra-articular Injections** of **Fasitibant** in **Patients With Symptomatic Osteoarthritis of the Knee**.
Study Start Date : April 2014
Actual Primary Completion Date : January 2015
Actual Study Completion Date : January 2015

Resource links provided by the National Library of Medicine NIH NLM

MedlinePlus Genetics related topics: Osteoarthritis

MedlinePlus related topics: Osteoarthritis

U.S. FDA Resources

Arms and Interventions

Go to

Arm	Intervention/treatment
Experimental: **Fasitibant** low dose<br>Drug: solution for **intra-articular injection**	Drug: **Fasitibant-** low dose<br>Single **intra-articular injection** of low dose of **fasitibant**
Experimental: **Fasitibant** intermediate dose<br>Drug: solution for **intra-articular injection**	Drug: **Fasitibant-** intermediate dose<br>Single **intra-articular injection** of intermediate dose of **fasitibant**
Experimental: **Fasitibant** high dose<br>Drug: solution for **intra-articular injection**	Drug: **Fasitibant-** high dose<br>Single **intra-articular injection** of high dose of **fasitibant**
Placebo Comparator: PLACEBO<br>Drug: solution for **intra-articular injection**	Drug: Placebo comparator<br>Single **intra-articular injection** of placebo

Outcome Measures

Go to

Primary Outcome Measures :

1. Change in WOMAC A [Time Frame: from baseline up to 2 weeks after randomisation]

   The validated Western Ontario and McMaster University questionnaire (WOMAC) was used to measure total **knee** pain choosing its visual analogue scale version (VAS). The WOMAC VA 3.1 A subscore (WOMAC A) ranges from 0 to 500 mm (summing up five VAS 0-100 mm) with higher scores indicating more pain.

Secondary Outcome Measures :

1. Change in WOMAC INDEX [Time Frame: from baseline up to 6 weeks after randomisation]

   The WOMAC VA 3.1 Index score (WOMAC INDEX) is the sum of WOMAC A (total pain), WOMAC B (stiffness) and WOMAC C (functional impairment) subscores. The WOMAC INDEX score ranges from 0 to 2400 mm, with higher scores indicating higher disease burden.

2. Responder Rate According to OMERACT-OARSI Criteria [Time Frame: from baseline up to 6 weeks after randomisation]

   Percentage of responders according to Outcome Measures in Rheumatology-**Osteoarthritis** Research Society International criteria (OMERACT-OARSI criteria). **Patients** with at least 50 % improvement in pain or in function scores are considered responders. Alternatively, **patients** are considered responders if they show at least 20% improvement in at least two of the following scores: pain, function and **Patients's** Global Assessment (PGA) scores.

3. Euro Quality of Life Questionnaire (EQ-5D-5L) Responder Rate [Time Frame: from baseline up to 6 weeks after randomisation]

   Response based on change ≥ 20 % from baseline for EQ-5D-5L index value

Eligibility Criteria

Go to

Information from the National Library of Medicine NIH NLM

Choosing to participate in a study is an important personal decision. Talk with your doctor and family members or friends about deciding to join a study. To learn more about this study, you or your doctor may contact the study research staff using the contacts provided below. For general information, Learn About Clinical Studies.

Ages Eligible for Study: 40 Years to 80 Years (Adult, Older Adult)
Sexes Eligible for Study: All
Accepts Healthy Volunteers: No

Criteria

Inclusion Criteria:

- Male or female patients aged 40 to 80 years with BMI < 30 kg/m²
- Patient with Kellgren-Lawrence Grade 2 to 3, symptomatic primary osteoarthritis at the index knee (ACR criteria), for which an IA treatment is indicated
- Pain of moderate to severe intensity, even if treated with chronic doses of non steroidal antinflammatory drugs

Exclusion Criteria:

- History of hypersensitivity/allergy to drugs including paracetamol and to disinfectants
- Any pharmacological treatment of concomitant disease(s) started or changed during 4 weeks prior to randomisation, or likely to be changed during the course of the study
- Use of systemic or topical corticosteroids > 10 mg prednisolone equivalent per day, or immunosuppressant drugs
- Current use of any pain or OA medication (e.g. NSAIDs, COX-2 inhibitors, analgesics, antidepressive agents), including topical treatments
- Viscosupplementation to the target knee administered < 4 months prior to randomisation and/or scheduled during the course of the study
- Evidence of clinically significant hepatic disease or of moderate or severe renal insufficiency
- Current use of any medications that are substrate of CYP3A4 and/or moderate or strong CYP3A4 inhibitors
- Patients with any clinically relevant or unstable disease, or malignant neoplasms that, in the opinion of the Investigator, may pose the patient at risk, or confound the efficacy and safety results of the study
- Patients with any clinically relevant abnormal safety laboratory test results, and/or abnormalities in vital signs, and/or ECG parameters
- Pregnant and breastfeeding women
- Any sign of significant immunodeficiency, systemic infection, knee infection or knee bursitis
- Patients with bleeding diathesis or on therapy with anticoagulants

Contacts and Locations

Go to

Information from the National Library of Medicine NIH NLM

To learn more about this study, you or your doctor may contact the study research staff using the contact information provided by the sponsor.

Please refer to this study by its ClinicalTrials.gov identifier (NCT number): ***NCT02205814***

Locations

Show 25 study locations

Sponsors and Collaborators

Menarini Group

Investigators

Study Chair: Karel Pavelka, Professor Institute of Rheumatology, Charles University Faculty Hospital, Na Slupi 4, 128 50 Prague 2, Czech Republic

More Information

Go to

Responsible Party: Menarini Group
ClinicalTrials.gov Identifier: NCT02205814 History of Changes
Other Study ID Numbers: BKOS-04
2013-004999-35 (EudraCT Number)
First Posted: July 31, 2014 Key Record Dates
Results First Posted: September 16, 2015
Last Update Posted: November 3, 2015
Last Verified: October 2015

Keywords provided by Menarini Group:

Osteoarthritis, **Knee**
Arthritis
WOMAC
Injections, **intra-articular**
Fasitibant
MEN 16132

Additional relevant MeSH terms:

Osteoarthritis
Osteoarthritis, **Knee**
Arthritis
Joint Diseases
Musculoskeletal Diseases
Rheumatic Diseases
(4-amino-5-(4-(4-(2,4-dichloro-3-(2,4-dimethyl-8-quinolyloxymethyl)phenylsulfonamido)tetrahydro-2H-4-pyranoylcarbonyl)piperazino)-5-oxopentyl)(trimethyl)ammonium
Bradykinin B2 Receptor Antagonists
Bradykinin Receptor Antagonists
Molecular Mechanisms of Pharmacological Action
Complement Inactivating Agents
Immunosuppressive Agents
Immunologic Factors
Physiological Effects of Drugs

This is the classic website, which will be retired eventually. Please visit the modernized ClinicalTrials.gov instead.

Fasitibant Intra-articular Injection in Patients With Symptomatic Osteoarthritis of the Knee (ALBATROSS-3)

The safety and scientific validity of this study is the responsibility of the study sponsor and investigators. Listing a study does not mean it has been evaluated by the U.S. Federal Government. Read our disclaimer for details.

ClinicalTrials.gov Identifier: NCT02205814

Recruitment Status : Completed
First Posted : July 31, 2014
Results First Posted : September 16, 2015
Last Update Posted : November 3, 2015

View this study on the modernized ClinicalTrials.gov

Sponsor:
Menarini Group

Information provided by (Responsible Party):
Menarini Group

**Study Type**	Interventional
**Study Design**	Allocation: Randomized; Intervention Model: Parallel Assignment; Masking: Quadruple (Participant, Care Provider, Investigator, Outcomes Assessor); Primary Purpose: Treatment
**Condition**	**Knee Osteoarthritis**
**Interventions**	Drug: **Fasitibant-** low dose<br>Drug: **Fasitibant-** intermediate dose<br>Drug: **Fasitibant-** high dose<br>Drug: Placebo comparator
**Enrollment**	436

Participant Flow

Go to

Recruitment Details	The first patient was screened on 28th April 2014. The first patient was randomised on 6th May 2014. The last patient completed the study on 6th January 2015.<br>The study was conducted in 25 study sites in Czech Republic, Germany, Italy and US.
Pre-assignment Details	A total of 645 patients entered a 2-week Screening period (including wash out); 209 of them were screen failed. One patient randomised to PLACEBO did not receive the study treatment (counted for ITT but not in safety population). Five patients received the study treatment without randomisation (not counted for ITT, but in safety population).

Arm/Group Title	Fasitibant Low Dose	Fasitibant Intermediate Dose	Fasitibant High Dose	PLACEBO
Arm/Group Description	Drug: solution for intra-articular injection<br>Fasitibant- low dose: Single intra-articular injection of low dose of fasitibant	Drug: solution for intra-articular injection<br>Fasitibant- intermediate dose: Single intra-articular injection of intermediate dose of fasitibant	Drug: solution for intra-articular injection<br>Fasitibant- high dose: Single intra-articular injection of high dose of fasitibant	Drug: solution for intra-articular injection<br>Placebo comparator: Single intra-articular injection of placebo
Period Title: **Overall Study**				
Started	110	110	108	108
Completed	108	109	103	104
Not Completed	2	1	5	4
Reason Not Completed				
Lost to Follow-up	0	0	0	1
Lack of Efficacy	1	0	1	1
Physician Decision	0	0	1	0
Protocol Violation	1	0	0	1
Withdrawal by Subject	0	1	2	1
not compliant with study procedures	0	0	1	0

Baseline Characteristics

Go to

Arm/Group Title	Fasitibant Low Dose	Fasitibant Intermediate Dose	Fasitibant High Dose	PLACEBO	Total
Arm/Group Description	Drug: solution for intra-articular injection<br>Fasitibant- low dose: Single intra-articular injection of low dose of fasitibant	Drug: solution for intra-articular injection<br>Fasitibant- intermediate dose: Single intra-articular injection of intermediate dose of fasitibant	Drug: solution for intra-articular injection<br>Fasitibant- high dose: Single intra-articular injection of high dose of fasitibant	Drug: solution for intra-articular injection<br>Placebo comparator: Single intra-articular injection of placebo	Total of all reporting groups
Overall Number of Baseline Participants	108	108	107	108	431
Baseline Analysis Population Description	Baseline analysis was performed on the ITT-population (n=431). The ITT-population included all patients randomised via IXRS.				
**Age, Continuous**<br>Mean (Standard Deviation)<br>Unit of measure: Years					
Age — Number Analyzed	108 participants	108 participants	107 participants	108 participants	431 participants
	65.3 (7.61)	63.2 (8.73)	64.7 (8.43)	64.4 (8.50)	64.4 (8.34)
**Sex: Female, Male**<br>Measure Type: Count of Participants<br>Unit of measure: Participants					
Number Analyzed	108 participants	108 participants	107 participants	108 participants	431 participants
Female	62 57.4%	71 65.7%	64 59.8%	66 61.1%	263 61.0%
Male	46 42.6%	37 34.3%	43 40.2%	42 38.9%	168 39.0%
**Ethnicity (NIH/OMB)**<br>Measure Type: Count of Participants<br>Unit of measure: Participants					
Number Analyzed	108 participants	108 participants	107 participants	108 participants	431 participants
Hispanic or Latino	1 0.9%	0 0.0%	1 0.9%	2 1.9%	4 0.9%
Not Hispanic or Latino	107 99.1%	108 100.0%	106 99.1%	106 98.1%	427 99.1%
Unknown or Not Reported	0 0.0%	0 0.0%	0 0.0%	0 0.0%	0 0.0%
**BMI**<br>Mean (Standard Deviation)<br>Unit of measure: Kg/m2					
Number Analyzed	108 participants	108 participants	107 participants	108 participants	431 participants
	27.1 (2.12)	26.5 (2.83)	27.1 (2.35)	27.0 (2.60)	26.9 (2.50)
**WOMAC A** [1]<br>Mean (Standard Deviation)<br>Unit of measure: Units on a scale					
Number Analyzed	108 participants	108 participants	107 participants	108 participants	431 participants
	286.5 (40.4)	282.7 (40.08)	278.3 (38.11)	275.5 (39.81)	280.8 (39.61)
	[1] Measure Description: The validated Western Ontario and McMaster University questionnaire (WOMAC) was used to measure total knee pain choosing its visual analogue scale version (VAS). The WOMAC VA 3.1 A subscore (WOMAC A) ranges from 0 to 500 mm (summing up five VAS 0-100 mm) with higher scores indicating more pain.				
**WOMAC INDEX** [1]<br>Mean (Standard Deviation)<br>Unit of measure: Units on a scale					
Number Analyzed	108 participants	108 participants	107 participants	108 participants	431 participants
	1321.5 (278.88)	1275.4 (283.60)	1282.6 (274.50)	1293.5 (239.73)	1293.3 (269.35)
	[1] Measure Description: The WOMAC VA 3.1 Index score (WOMAC INDEX) is the sum of WOMAC A (total pain), WOMAC B (stiffness) and WOMAC C (functional impairment) subscores. The WOMAC INDEX score ranges from 0 to 2400 mm, with higher scores indicating higher disease burden.				
**EQ VAS** [1]<br>Mean (Standard Deviation)<br>Unit of measure: Units on a scale					
Number Analyzed	108 participants	108 participants	107 participants	108 participants	431 participants
	63.1 (20.54)	64.3 (17.21)	67.4 (18.18)	65.7 (19.47)	65.1 (18.89)
	[1] Measure Description: The EQ visual analogue scale (EQ VAS) recorded the respondent's self-rated health on a 20 cm vertical VAS with endpoints labelled 'the best health you can imagine' on top (equal to 100) and 'the worst health you can imagine' at the bottom (equal to 0).				

Outcome Measures

Go to

1. Primary Outcome

Title	Change in WOMAC A
Description	The validated Western Ontario and McMaster University questionnaire (WOMAC) was used to measure total **knee** pain choosing its visual analogue scale version (VAS). The WOMAC VA 3.1 A subscore (WOMAC A) ranges from 0 to 500 mm (summing up five VAS 0-100 mm) with higher scores indicating more pain.
Time Frame	from baseline up to 2 weeks after randomisation

Outcome Measure Data

Analysis Population Description
The primary efficacy analysis was performed on the ITT-population (n=431).

Arm/Group Title	Fasitibant Low Dose	Fasitibant Intermediate Dose	Fasitibant High Dose	PLACEBO
Arm/Group Description:	Drug: solution for intra-articular injection<br>Fasitibant- low dose: Single intra-articular injection of low dose of fasitibant	Drug: solution for intra-articular injection<br>Fasitibant- intermediate dose: Single intra-articular injection of intermediate dose of fasitibant	Drug: solution for intra-articular injection<br>Fasitibant- high dose: Single intra-articular injection of high dose of fasitibant	Drug: solution for intra-articular injection<br>Placebo comparator: Single intra-articular injection of placebo
Overall Number of Participants Analyzed	108	108	107	108
Mean (Standard Deviation)<br>Unit of Measure: units on a scale				
Week 1 after randomisation	-91.8 (101.85)	-110.0 (99.48)	-109.8 (94.65)	-93.7 (94.15)
Week 2 after randomisation	-106.1 (101.88)	-131.5 (96.41)	-115.9 (104.61)	-117.2 (90.15)

Statistical Analysis 1

Statistical Analysis Overview	Comparison Group Selection	Fasitibant Low Dose, Fasitibant Intermediate Dose, Fasitibant High Dose, PLACEBO
	Comments	[Not Specified]
	Type of Statistical Test	Superiority or Other
	Comments	[Not Specified]
Statistical Test of Hypothesis	P-Value	<0.05
	Comments	Fourhundred evaluable patients were supposed to provide approximately 80% power in rejecting the null hypothesis of equality between any dose of fasitibant and placebo based on previous results and an overall significance level of 5% (two-sided).
	Method	mixed linear model for repeated measures
	Comments	[Not Specified]

2. Secondary Outcome

Title	Change in WOMAC INDEX
Description	The WOMAC VA 3.1 Index score (WOMAC INDEX) is the sum of WOMAC A (total pain), WOMAC B (stiffness) and WOMAC C (functional impairment) subscores. The WOMAC INDEX score ranges from 0 to 2400 mm, with higher scores indicating higher disease burden.
Time Frame	from baseline up to 6 weeks after randomisation

Outcome Measure Data

Analysis Population Description
The secondary efficacy analysis was performed on the ITT population (n=431).

Arm/Group Title	Fasitibant Low Dose	Fasitibant Intermediate Dose	Fasitibant High Dose	PLACEBO
Arm/Group Description:	Drug: solution for intra-articular injection<br>Fasitibant- low dose: Single intra-articular injection of low dose of fasitibant	Drug: solution for intra-articular injection<br>Fasitibant- intermediate dose: Single intra-articular injection of intermediate dose of fasitibant	Drug: solution for intra-articular injection<br>Fasitibant- high dose: Single intra-articular injection of high dose of fasitibant	Drug: solution for intra-articular injection<br>Placebo comparator: Single intra-articular injection of placebo
Overall Number of Participants Analyzed	108	108	107	108
Mean (Standard Deviation)<br>Unit of Measure: units on a scale				
Week 1 after randomisation	-396.0 (464.24)	-460.5 (469.68)	-445.2 (424.07)	-413.2 (452.04)
Week 2 after randomisation	-448.0 (477.95)	-563.0 (456.71)	-488.7 (480.39)	-517.7 (440.75)
week 4 after randomisation	-516.4 (513.70)	-628.4 (500.17)	-493.4 (513.94)	-562.3 (487.25)
Week 6 after randomisation	-566.3 (525.63)	-653.8 (516.31)	-547.6 (522.37)	-581.3 (503.37)

Statistical Analysis 1

Statistical Analysis Overview	Comparison Group Selection	Fasitibant Low Dose, Fasitibant Intermediate Dose, Fasitibant High Dose, PLACEBO
	Comments	All secondary efficacy variables were analysed on the ITT population only. Multiplicity was adjusted using the Hochberg procedure. The continuous secondary efficacy variables were analysed over time and were treated in the same way as the primary efficacy variable with respective output.
	Type of Statistical Test	Superiority or Other
	Comments	[Not Specified]
Statistical Test of Hypothesis	P-Value	<0.05
	Comments	Fourhundred evaluable patients were supposed to provide approximately 80% power in rejecting the null hypothesis of equality between any dose of fasitibant and placebo based on previous results and an overall significance level of 5% (two-sided).
	Method	mixed linear model for repeated measures
	Comments	[Not Specified]

3. Secondary Outcome

Title	Responder Rate According to OMERACT-OARSI Criteria
Description	Percentage of responders according to Outcome Measures in Rheumatology-**Osteoarthritis** Research Society International criteria (OMERACT-OARSI criteria). **Patients** with at least 50 % improvement in pain or in function scores are considered responders. Alternatively, **patients** are considered responders if they show at least 20% improvement in at least two of the following scores: pain, function and **Patients's** Global Assessment (PGA) scores.
Time Frame	from baseline up to 6 weeks after randomisation

Outcome Measure Data

Analysis Population Description
The secondary efficacy variables were analysed in the ITT population (n=431).

Arm/Group Title	Fasitibant Low Dose	Fasitibant Intermediate Dose	Fasitibant High Dose	PLACEBO
Arm/Group Description:	Drug: solution for intra-articular injection<br>Fasitibant- low dose: Single intra-articular injection of low dose of fasitibant	Drug: solution for intra-articular injection<br>Fasitibant- intermediate dose: Single intra-articular injection of intermediate dose of fasitibant	Drug: solution for intra-articular injection<br>Fasitibant- high dose: Single intra-articular injection of high dose of fasitibant	Drug: solution for intra-articular injection<br>Placebo comparator: Single intra-articular injection of placebo
Overall Number of Participants Analyzed	108	108	107	108
Measure Type: Number<br>Unit of Measure: percentage of responders				
Week 1 after randomisation	51.9	56.5	63.6	55.6
Week 2 after randomisation	59.3	72.2	62.6	68.5
Week 4 after randomisation	65.7	72.2	65.4	66.7
Week 6 after randomisation	71.3	74.1	67.3	67.6

4. Secondary Outcome

Title	Euro Quality of Life Questionnaire (EQ-5D-5L) Responder Rate
Description	Response based on change ≥ 20 % from baseline for EQ-5D-5L index value
Time Frame	from baseline up to 6 weeks after randomisation

Outcome Measure Data

Analysis Population Description
The secondary efficacy analysis was performed on the ITT-population (n=431).

Arm/Group Title	Fasitibant Low Dose	Fasitibant Intermediate Dose	Fasitibant High Dose	PLACEBO
Arm/Group Description:	Drug: solution for intra-articular injection<br>Fasitibant- low dose: Single intra-articular injection of low dose of fasitibant	Drug: solution for intra-articular injection<br>Fasitibant- intermediate dose: Single intra-articular injection of intermediate dose of fasitibant	Drug: solution for intra-articular injection<br>Fasitibant- high dose: Single intra-articular injection of high dose of fasitibant	Drug: solution for intra-articular injection<br>Placebo comparator: Single intra-articular injection of placebo
Overall Number of Participants Analyzed	108	108	107	108
Measure Type: Number<br>Unit of Measure: percentage of responders				
Week 2 after randomisation	18.5	18.5	20.6	24.1
Week 6 after randomisation	23.1	27.8	25.2	21.3

Adverse Events

Go to

Time Frame	For all patients receiving the study treatment (safety population, N=435), adverse event data were collected over a period of maximal 15 weeks.
Adverse Event Reporting Description	An Adverse Event (AE) is any untoward medical occurrence in a patient or clinical trial subject administered a medicinal product and which does not necessarily have a causal relationship with this treatment.

Arm/Group Title	Fasitibant Low Dose	Fasitibant Intermediate Dose	Fasitibant High Dose	PLACEBO
Arm/Group Description	Drug: solution for intra-articular injection<br>Fasitibant- low dose: Single intra-articular injection of low dose of fasitibant	Drug: solution for intra-articular injection<br>Fasitibant- intermediate dose: Single intra-articular injection of intermediate dose of fasitibant	Drug: solution for intra-articular injection<br>Fasitibant- high dose: Single intra-articular injection of high dose of fasitibant	Drug: solution for intra-articular injection<br>Placebo comparator: Single intra-articular injection of placebo

All-Cause Mortality

	Fasitibant Low Dose		Fasitibant Intermediate Dose		Fasitibant High Dose		PLACEBO	
	Affected / at Risk (%)		Affected / at Risk (%)		Affected / at Risk (%)		Affected / at Risk (%)	
Total	--/--		--/--		--/--		--/--	

Serious Adverse Events

	Fasitibant Low Dose		Fasitibant Intermediate Dose		Fasitibant High Dose		PLACEBO	
	Affected / at Risk (%)	# Events	Affected / at Risk (%)	# Events	Affected / at Risk (%)	# Events	Affected / at Risk (%)	# Events
Total	2/110 (1.82%)		2/110 (1.82%)		2/108 (1.85%)		4/107 (3.74%)	
Gastrointestinal disorders								
Inguinal hernia †[1]	0/110 (0.00%)	0	0/110 (0.00%)	0	1/108 (0.93%)	1	0/107 (0.00%)	0
Infections and infestations								
Urinary tract infection †[1]	0/110 (0.00%)	0	0/110 (0.00%)	0	1/108 (0.93%)	1	0/107 (0.00%)	0
Injury, poisoning and procedural complications								
Femur fracture †[1]	0/110 (0.00%)	0	0/110 (0.00%)	0	0/108 (0.00%)	0	1/107 (0.93%)	1
Humerus fracture †[1]	0/110 (0.00%)	0	1/110 (0.91%)	1	0/108 (0.00%)	0	0/107 (0.00%)	0
Thoracic vertebral fracture †[1]	0/110 (0.00%)	0	1/110 (0.91%)	1	0/108 (0.00%)	0	0/107 (0.00%)	0
Investigations								
Hepatic enzyme increased †[1]	0/110 (0.00%)	0	0/110 (0.00%)	0	0/108 (0.00%)	0	1/107 (0.93%)	1
Metabolism and nutrition disorders								
Hypercalcaemia †[1]	0/110 (0.00%)	0	0/110 (0.00%)	0	0/108 (0.00%)	0	1/107 (0.93%)	1
Musculoskeletal and connective tissue disorders								
Arthralgia †[1]	0/110 (0.00%)	0	0/110 (0.00%)	0	0/108 (0.00%)	0	1/107 (0.93%)	1
Neoplasms benign, malignant and unspecified (incl cysts and polyps)								
Breast cancer female †[1]	1/110 (0.91%)	1	0/110 (0.00%)	0	0/108 (0.00%)	0	0/107 (0.00%)	0
Respiratory, thoracic and mediastinal disorders								
Laryngeal oedema †[1]	1/110 (0.91%)	1	0/110 (0.00%)	0	0/108 (0.00%)	0	0/107 (0.00%)	0
Surgical and medical procedures								
Hernia repair †[1]	0/110 (0.00%)	0	0/110 (0.00%)	0	1/108 (0.93%)	1	0/107 (0.00%)	0
Knee arthroplasty †[1]	0/110 (0.00%)	0	0/110 (0.00%)	0	0/108 (0.00%)	0	1/107 (0.93%)	1
Malignant breast lump removal †[1]	1/110 (0.91%)	1	0/110 (0.00%)	0	0/108 (0.00%)	0	0/107 (0.00%)	0

† Indicates events were collected by systematic assessment
1 Term from vocabulary, MedDRA 16.1

Other (Not Including Serious) Adverse Events

Frequency Threshold for Reporting Other Adverse Events	1%

	Fasitibant Low Dose		Fasitibant Intermediate Dose		Fasitibant High Dose		PLACEBO	
	Affected / at Risk (%)	# Events	Affected / at Risk (%)	# Events	Affected / at Risk (%)	# Events	Affected / at Risk (%)	# Events
Total	28/110 (25.45%)		46/110 (41.82%)		36/108 (33.33%)		44/107 (41.12%)	
Gastrointestinal disorders								
Diarrhea †[1]	1/110 (0.91%)	1	1/110 (0.91%)	1	2/108 (1.85%)	2	3/107 (2.80%)	3
Toothache †[1]	1/110 (0.91%)	1	1/110 (0.91%)	2	1/108 (0.93%)	1	2/107 (1.87%)	3
Abdominal pain †[1]	0/110 (0.00%)	0	2/110 (1.82%)	2	0/108 (0.00%)	0	2/107 (1.87%)	2
General disorders								
Injection site haematoma †[1]	0/110 (0.00%)	0	2/110 (1.82%)	2	0/108 (0.00%)	0	1/107 (0.93%)	1
Infections and infestations								
Nasopharyngitis †[1]	4/110 (3.64%)	4	17/110 (15.45%)	18	11/108 (10.19%)	11	13/107 (12.15%)	13
Gastroenteritis †[1]	0/110 (0.00%)	0	2/110 (1.82%)	2	0/108 (0.00%)	0	0/107 (0.00%)	0
Rhinitis †[1]	2/110 (1.82%)	2	1/110 (0.91%)	1	2/108 (1.85%)	2	0/107 (0.00%)	0
Urinary tract infection †[1]	3/110 (2.73%)	3	1/110 (0.91%)	1	2/108 (1.85%)	2	3/107 (2.80%)	3
Injury, poisoning and procedural complications								
Contusion †[1]	2/110 (1.82%)	2	0/110 (0.00%)	0	0/108 (0.00%)	0	1/107 (0.93%)	1
Ligament sprain †[1]	0/110 (0.00%)	0	2/110 (1.82%)	2	0/108 (0.00%)	0	0/107 (0.00%)	0
Investigations								
Gamma-glutamyltransferase increased †[1]	0/110 (0.00%)	0	0/110 (0.00%)	0	2/108 (1.85%)	2	0/107 (0.00%)	0
Musculoskeletal and connective tissue disorders								
Arthralgia †[1]	4/110 (3.64%)	6	7/110 (6.36%)	7	5/108 (4.63%)	6	8/107 (7.48%)	8
Back pain †[1]	2/110 (1.82%)	2	4/110 (3.64%)	5	5/108 (4.63%)	5	6/107 (5.61%)	6
Joint swelling †[1]	1/110 (0.91%)	1	1/110 (0.91%)	1	1/108 (0.93%)	1	2/107 (1.87%)	2
Muscle spasms †[1]	1/110 (0.91%)	1	0/110 (0.00%)	0	2/108 (1.85%)	2	1/107 (0.93%)	1
Musculoskeletal pain †[1]	0/110 (0.00%)	0	2/110 (1.82%)	2	0/108 (0.00%)	0	0/107 (0.00%)	0
Pain in extremity †[1]	2/110 (1.82%)	2	0/110 (0.00%)	0	1/108 (0.93%)	1	0/107 (0.00%)	0
Nervous system disorders								
Headache †[1]	3/110 (2.73%)	4	3/110 (2.73%)	4	1/108 (0.93%)	1	2/107 (1.87%)	2
Vascular disorders								
Hypertension †[1]	2/110 (1.82%)	2	0/110 (0.00%)	0	1/108 (0.93%)	1	0/107 (0.00%)	0

† Indicates events were collected by systematic assessment
1 Term from vocabulary, MedDRA 16.1

Limitations and Caveats

Go to

[Not Specified]

More Information

Go to

Certain Agreements

Principal Investigators are NOT employed by the organization sponsoring the study.

There IS an agreement between Principal Investigators and the Sponsor (or its agents) that restricts the PI's rights to discuss or publish trial results after the trial is completed.

Prior to submitting the results of this study for publication or presentation, the Investigator will allow the sponsor at least 30 days time to review and comment upon the publication manuscript. It is agreed, that the results of the study will not be submitted for presentation, abstract, poster exhibition, or publication by the investigator until the sponsor has reviewed/commented and agreed to any publication.

Results Point of Contact

Name/Title: Angela Capriati, MD PhD - Corporate Director Clinical Research
Organization: MENARINI Group
Phone: +39 055 5680 ext 9990
EMail: acapriati@menarini-ricerche.it

Responsible Party: Menarini Group
ClinicalTrials.gov Identifier: NCT02205814 History of Changes
Other Study ID Numbers: BKOS-04
2013-004999-35 (EudraCT Number)
First Submitted: July 14, 2014
First Posted: July 31, 2014
Results First Submitted: July 6, 2015
Results First Posted: September 16, 2015
Last Update Posted: November 3, 2015

This is the classic website, which will be retired eventually. Please visit the modernized ClinicalTrials.gov instead.

A Study to Learn How the Study Treatment BAY2395840 Moves Into, Through and Out of the Body, How Safe it is, and How it Affects the Body in Participants With Moderate Reduced Kidney Function and in Healthy Male and Female Participants With Normal Kidney Function

ClinicalTrials.gov Identifier: NCT05406219

The safety and scientific validity of this study is the responsibility of the study sponsor and investigators. Listing a study does not mean it has been evaluated by the U.S. Federal Government. Read our disclaimer for details.

Recruitment Status : Withdrawn (Discontinuation of program.)
First Posted : June 6, 2022
Last Update Posted : April 19, 2023

View this study on the modernized ClinicalTrials.gov

Sponsor:
Bayer

Information provided by (Responsible Party):
Bayer

Study Details | Tabular View | No Results Posted | Disclaimer | How to Read a Study Record

Study Description

Go to ▾

Brief Summary:

Researchers are looking for a better way to treat people who have inflammatory conditions.

Inflammatory conditions may result from an increased activation of the body's natural defenses (the immune system) and are characterized by swelling, redness, heat, and pain.

The study treatment BAY2395840 is under development for use in inflammatory conditions. It works by blocking a protein, the B1 receptor, whose activation is involved in inflammatory responses.

The main purpose of this study is to learn how a single dose of BAY2395840 moves into, through and out of the body in participants with a moderate reduction of kidney function compared to matched participants with normal kidney function.

To answer this, the researchers will compare:

- the (average) total level of BAY2395840 in the blood (also known as AUC), and
- the (average) highest level of BAY2395840 in the blood (also known as Cmax) between the two groups of participants. The participants do not benefit from this study. However, the study will provide information on how to use BAY2395840 in later studies in people with inflammatory conditions. As some people with these conditions may also have kidney problems, this study is done in participants with moderate reduction of kidney function to characterize the use of BAY2395840 in this patient group.

All participants will take a single dose of BAY2395840 as tablets. Each participant will be in the study for up to 5 weeks. They will stay in-house for 4 days, including one treatment day. In addition, one visit before and one visit after the in-house phase to the study site is planned.

Condition or disease	Intervention/treatment	Phase
Diabetic Neuropathic Pain<br>Renal Impairment<br>Healthy Volunteers	Drug: BAY2395840	Phase 1

Study Design

Go to ▾

Study Type :	Interventional (Clinical Trial)
Actual Enrollment :	0 participants
Allocation:	Non-Randomized
Intervention Model:	Sequential Assignment
Masking:	None (Open Label)
Primary Purpose:	Basic Science
Official Title:	An Open-label, Single-dose Study to Evaluate the Pharmacokinetics, Safety, and Tolerability of BAY 2395840 in Participants With Moderate Renal Impairment and in Healthy Male and Female Participants With Normal Renal Function
Estimated Study Start Date :	May 15, 2023
Estimated Primary Completion Date :	August 17, 2023
Estimated Study Completion Date :	October 12, 2023

Arms and Interventions

Go to ▾

Arm	Intervention/treatment
Experimental: Group 1: moderately impaired renal function<br>Participants with moderately impaired renal function will receive a single dose of BAY2395840.	Drug: BAY2395840<br>Immediate release (IR) tablet, oral administration
Experimental: Group 2: normal renal function matched to Group 1<br>Participants with normal renal function matched to Group 1 will receive a single dose of BAY2395840.	Drug: BAY2395840<br>Immediate release (IR) tablet, oral administration
Experimental: Group 3: normal renal function aiming to balance out Group 2<br>Participants with normal renal function aiming to balance out Group 2 for the age and gender investigations will receive a single dose of BAY2395840.	Drug: BAY2395840<br>Immediate release (IR) tablet, oral administration

Outcome Measures

Go to ▾

Primary Outcome Measures :

1. Area under the concentration vs. time curve from zero to infinity (AUC) after single dose of BAY 2395840 [Time Frame: From pre-dose up to 72 hours post administration]
   AUC(0-tlast) will be used as the main parameter if AUC cannot be reliably determined
2. Maximum observed drug concentration in measured matrix (Cmax) after single dose of BAY 2395840 [Time Frame: From pre-dose up to 72 hours post administration]
3. Unbound area under the concentration vs. time curve from zero to infinity (AUCu) after single (first) dose of BAY 2395840 [Time Frame: From pre-dose up to 72 hours post administration]
   AUC(0-tlast)u will be used as the main parameter if AUC cannot be reliably determined
4. Unbound maximum observed drug concentration in measured matrix (Cmax,u) after single dose of BAY 2395840 [Time Frame: From pre-dose up to 72 hours post administration]

Secondary Outcome Measures :

1. Number of participants with treatment-emergent adverse events (TEAEs) [Time Frame: From application of study intervention until follow-up Day 9]

Eligibility Criteria

Go to ▾

Information from the National Library of Medicine

NIH NLM

Choosing to participate in a study is an important personal decision. Talk with your doctor and family members or friends about deciding to join a study. To learn more about this study, you or your doctor may contact the study research staff using the contacts provided below. For general information, Learn About Clinical Studies.

Ages Eligible for Study:	18 Years and older (Adult, Older Adult)
Sexes Eligible for Study:	All
Accepts Healthy Volunteers:	Yes

Criteria

Inclusion Criteria:

- Participants must be 18 years of age or older, at the time of signing the informed consent.
- Participants who are - apart from renal function - overtly healthy for their age as determined by medical evaluation including medical history, physical examination, laboratory tests, and cardiac monitoring.
- Participants with moderate renal impairment with an eGFR of 30 to 59 mL/min/1.73 m^2 (inclusive) according to the Chronic Kidney Disease Epidemiology Collaboration formula (CKD-EPI)
- Normal renal function, as assessed by estimated glomerular filtration rate (eGFR) based on serum creatinine at screening according to the modified Chronic Kidney Disease Epidemiology Collaboration formula (CKD-EPI) formula: eGFR ≥90 mL/min/1.73 m^2. For participants with an age of 65 years and older, an eGFR of ≥ 60 mL/min/1.73 m^2 is acceptable.
- Body mass index (BMI) within the range 18 - 32 kg/m^2 (inclusive).
- Male and female participants had to use safe contraception, as defined by guidelines for contraception in clinical studies.
- Capable of giving signed informed consent.

Exclusion Criteria:

- Medical disorder, condition or history of such that would impair the participant's ability to take part in or complete this study.
- Acute renal failure or acute nephritis within the past 2 years.
- Existing chronic diseases requiring medication (applicable only for Group 2 and Group 3 for those younger than 65 years).

Contacts and Locations

Go to ▾

No Contacts or Locations Provided

More Information

Go to ▾

Additional Information:

Click here to find information for studies related to Bayer products. To find this study enter the ClinicalTrials.gov identifier (NCT) number or Bayer Study Identifier (ID) in the search field.

Responsible Party:	Bayer
ClinicalTrials.gov Identifier:	NCT05406219 History of Changes
Other Study ID Numbers:	22044
First Posted:	June 6, 2022 Key Record Dates
Last Update Posted:	April 19, 2023
Last Verified:	April 2023

Individual Participant Data (IPD) Sharing Statement:

Plan to Share IPD:	No
Plan Description:	Availability of this study's data will later be determined according to Bayer's commitment to the EFPIA/PhRMA "Principles for responsible clinical trial data sharing". This pertains to scope, timepoint and process of data access. As such, Bayer commits to sharing upon request from qualified researchers patient-level clinical trial data, study-level clinical trial data, and protocols from clinical trials in patients for medicines and indications approved in the US and EU as necessary for conducting legitimate research. This applies to data on new medicines and indications that have been approved by the EU and US regulatory agencies on or after January 01, 2014.<br>Interested researchers can use www.vivli.org to request access to anonymized patient-level data and supporting documents from clinical studies to conduct research. Information on the Bayer criteria for listing studies and other relevant information is provided in the member section of the portal.

Studies a U.S. FDA-regulated Drug Product: Yes
Studies a U.S. FDA-regulated Device Product: No

Additional relevant MeSH terms:

Neuralgia	Kidney Diseases
Renal Insufficiency	Urologic Diseases
Peripheral Nervous System Diseases	Female Urogenital Diseases
Neuromuscular Diseases	Female Urogenital Diseases and Pregnancy Complications
Nervous System Diseases	Urogenital Diseases
Pain	Male Urogenital Diseases
Neurologic Manifestations	

Clinical Study Results Synopsis

This Clinical Study Results Synopsis is provided for patients and healthcare professionals to increase the transparency of Bayer's clinical research. This document is not intended to replace the advice of a healthcare professional and should not be considered as a recommendation. Patients should always seek medical advice before making any decisions on their treatment. Healthcare professionals should always refer to the specific labelling information approved for the patient's country or region. Data in this document or on the related website should not be considered as prescribing advice.
The study listed may include approved and non-approved formulations or treatment regimens. Overall data disclosed, may differ from published or presented data and are a reflection of the limited information provided here. The results from a single study need to be considered in the context of the totality of the available clinical research results for a drug. The results from a single study may not reflect the overall results for a drug.

The following information is the property of Bayer. Reproduction of all or part of this report is strictly prohibited without prior written permission from Bayer. Commercial use of the information is only possible with the written permission of the proprietor and is subject to a license fee. Please note that the General Conditions of Use and the Privacy Statement of bayer.com apply to the contents of this file.

Date of study report	01 JUN 2023
Study title	A randomized, double-blind, cross-over, placebo-controlled, multi-center, Phase 2a study to assess the safety and efficacy of BAY 2395840 in patients with diabetic neuropathic pain
Sponsor	Bayer
Sponsor's study ID	19636
NCT number	NCT05219812
EudraCT number	2021-001392-17
Study Phase	2a
Indication	Neuropathic pain associated with diabetic peripheral neuropathy<br>Peripheral neuropathic pain<br>Diabetes mellitus
Study objectives	Primary objective:<br>• To evaluate the efficacy of BAY2395840 on pain associated with diabetic neuropathic pain (DNP) as compared with placebo<br>Secondary objectives:<br>• To evaluate safety and tolerability of BAY2395840 given for the treatment of DNP as compared with placebo<br>• To further evaluate the efficacy of BAY2395840 on pain and associated symptoms
Test drug<br>Active ingredient(s)<br>Dose<br>Route of administration<br>Duration of treatment	BAY2395840<br>BAY2395840<br>Dosage A<br>Administration route A<br>Double-blind treatment with BAY2395840 in a period
Reference drug<br>Dose<br>Route of administration<br>Duration of treatment	Placebo<br>Not applicable<br>Administration route A<br>Double-blind treatment with placebo in a period<br>Single-blind placebo run-in : 21 days (3 weeks)
Main inclusion/exclusion criteria	• Had documented diagnosis of type 1 OR type 2 diabetes mellitus (DM)<br>• Had painful distal symmetrical sensorimotor diabetic neuropathy for at least 6 months prior to the screening and confirmed at screening via the modified Toronto Clinical Neuropathy Score with a score of at least 3.<br>• Weekly mean 24-hour average pain Numeric Rating Scale (NRS) ⩾ 4 with adequate variability (not the same score on all daily pain ratings) and

	compliance (non-missing pain score on at least 6 out of 7 consecutive days) in daily pain recording during the 7-day NRS baseline period.<br>• Neuropathic pain according to the Douleur Neuropathique 4 Questions (DN4) questionnaire at screening visit with a score of at least 4 out of 10.<br>• Participant should be on stable antidiabetic treatment for at least 3 months prior to the screening visit and there should be no pre-planned changes to antidiabetic treatment during this study.<br>• Participant was willing to use only rescue medication provided by the site and was willing to withdraw temporarily all other neuropathic pain medications which were in use before study participation.<br>• Participant was willing and able to use the electronic hand-held device on their own.
Study design	Multi-center, randomized, double-blind, placebo controlled, 2-treatment complete crossover study
Methodology	This study consisted of a screening phase (up to 21+2 days), 2 x 4 weeks intervention period, 3 weeks of single blind placebo run-in phase, and 14 ± 2 days of safety follow-up phase.<br>Patient reported outcome (PRO) assessments were collected using an electronic handheld device and site pads. Pain assessments based on the numeric rating scale (Pain Numeric Rating Scale [NRS]) were collected daily. All other PRO assessments were collected at Visits 2, 4, 6, 8, 10 and 12 using electronic site pads: Neuropathic Pain Symptom Inventory (NPSI), Patient Global Impression of Severity (PGI-S), Brief Pain Inventory-Short Form (BPI-SF), PROMIS Short Form - Sleep Disturbance, Hospital Anxiety and Depression Scale (HADS), Short Form Health Survey (SF-12).<br>Safety was monitored throughout the study (adverse events, clinical laboratory, vital signs, electrocardiogram). Blood samples for the measurement of plasma concentrations of BAY2395840 as well as for analyses of potential biomarkers were taken from all participants at dedicated time points (except optional pharmacokinetics [PK] sampling taken at optional Visits).
Statistical methods	For the primary endpoint analysis, the repeated measures of change from baseline in weekly mean 24-hour average pain intensity NRS scores over Weeks 1, 2, 3, and 4 were analyzed using a linear mixed model. The model was for treatment (BAY2395840 or placebo), period, timepoints (Weeks 1, 2, 3, and 4), treatment-timepoint interaction and period-timepoint interaction as factors having fixed effects and baseline pain NRS scores as between- and within-participant covariates. Specifically, the average baseline NRS scores from a participant were included as a between-participant covariate and difference of period-specific baseline scores from averaged baseline score as within-participant covariate.<br>Based on the primary analysis model, the estimated treatment difference on Week 4 (BAY2395840 vs. placebo) and associated 90% confidence interval (CI) was obtained for treatment comparisons. A negative placebo-corrected change from baseline indicates an improvement in pain symptoms after BAY2395840 treatment.<br>A graphical presentation of the estimated time-course of the treatment effect was also presented based on the model.<br>In addition to the aforementioned inferential analysis, a similar primary analysis approach using repeated measures analysis of covariance (ANCOVA) model were implemented under a Bayesian framework with non-informative priors on ‘true’ treatment effect. Based on the modeling, posterior probability distribution of the

	treatment effect was evaluated for aiding the decisions on efficacy of BAY2395840 on primary endpoint.<br>The weekly mean pain intensity scores were descriptively summarized by study intervention and for overall.
Early termination	Not applicable
Substantial protocol changes	Not applicable
Study period	Study Start Date: 16 FEB 2022<br>Study End Date: 21 NOV 2022
Study centers	Study was conducted at 29 centers in 6 countries. Germany (6), the United Kingdom (3), Spain (5), Czech Republic (7), Hungary (3), Slovakia (5)
Number of subjects	Planned: 75<br>Analyzed: 80
Criteria for evaluation<br>*Efficacy*	Primary endpoint:<br>• Change in weekly mean 24-hour average pain intensity score using the 11-point Numeric Rating Scale (NRS) from baseline to the end of intervention<br>Secondary endpoints:<br>• Change in Neuropathic Pain Symptom Inventory (NPSI) total score from baseline to the end of intervention<br>• Change in Patient Global Impression of Severity (PGI-S) from baseline to the end of intervention<br>• Number of subjects achieving a ⩾30% and a ⩾50% reduction in weekly mean 24-hour average pain intensity score (i.e. responder rates using NRS)
Safety	Secondary safety endpoint:<br>• Number of participants with treatment emergent adverse events
Clinical pharmacology	None

Subject disposition and baseline

- Subject Disposition

A total of 81 participants were randomized to 2 treatment sequences (41 to BAY2395840 - placebo, 40 to placebo - BAY2395840). Of these, 1 participant in the BAY2395840 - placebo treatment sequence never received study intervention. 78 participants were valid for the per protocol set (PPS). The PPS was the primary analysis set for efficacy. 3 participants (2 in BAY2395840 - placebo treatment sequence, 1 in placebo - BAY2395840 treatment sequence) did not complete the treatment of period 1. The reasons for non-completion of period 1 were adverse event (1 participant in BAY2395840 - placebo treatment sequence), participant decision (1 participant in BAY2395840 - placebo treatment sequence), and protocol deviation (1 participant in placebo - BAY2395840 treatment sequence). 3 participants (2 in BAY2395840 - placebo treatment sequence, 1 in placebo - BAY2395840 treatment sequence) did not complete the treatment of period 2. The reasons for non-completion of period 2 were death (1 participant in BAY2395840 - placebo treatment sequence), participant decision (1 participant in BAY2395840 - placebo treatment sequence), and adverse event (1 participant in placebo - BAY2395840 treatment sequence).

- Baseline Characteristics

Demographic characteristics were generally well balanced between the 2 treatment sequences. In the safety analysis set (SAF), 50.0% of all participants were female and all participants were white. The mean age of the participants was 64.8 years (standard deviation [SD]: 10.5), 58.8% of participants in the age group 65 to <85 years.

Efficacy

Primary endpoint:

- ***Change in weekly mean 24-hour average pain intensity score using the 11-point Numeric Rating Scale (NRS) from baseline to the end of intervention***

  At Week 4 in period 1 and period 2, the mean (SD) changes from baseline in weekly mean 24-hour average pain intensity score using the 11-point NRS were -0.93 (1.45) in the treatment of BAY2395840 and -1.23 (1.44) in the treatment of placebo (PPS). At Week 3, it was -0.67 (1.24) in the treatment of BAY2395840 and -1.06 (1.30) in the treatment of placebo. At Week 2 it was -0.44 (0.98) in the treatment of BAY2395840 and -0.75 (1.13) in the treatment of placebo (PPS). At Week 1, it was -0.33 (0.82) in the treatment of BAY2395840 and -0.39 (0.86) in the treatment of placebo (PPS).

  As the main analysis of the primary endpoint, a linear mixed model was applied to evaluate the repeated measures of change from baseline in weekly mean 24-hour average pain intensity NRS scores over Weeks 1, 2, 3, 4. At Week 4 in period 1 and period 2, the estimated mean (SE) treatment difference between BAY2395840 and placebo in the PPS was 0.07 (0.146) with 90% confidence interval (CI): -0.170; 0.314, i.e. no differences between BAY2395840 and placebo could be shown.

Secondary endpoints

- ***Change in Neuropathic Pain Symptom Inventory (NPSI) total score from baseline to the end of intervention***

  The mean (SD) change from baseline in the NPSI total score at Visit 6 / Visit 12 was -7.23 (15.10) during the treatment of BAY2395840 and -5.30 (11.44) during the treatment of placebo (PPS). At Visit 4 / Visit 10, it was -6.89 (10.53) during the treatment of BAY2395840 and -5.43 (10.56) during the treatment of placebo (PPS).

Table 1: NPSI: summary statistics and change from baseline by visit (per protocol set)

		Value at Visit				Change from Baseline			
Treatment	Visit	n	Mean (SD)	Median	Min, Max	n	Mean (SD)	Median	Min, Max
BAY2395840	Visit 2 / Visit 8	78	34.51 (19.82)	32.00	4.0, 81.0				
	Visit 4 / Visit 10	74	27.86 (17.20)	27.00	0.0, 69.0	74	-6.89 (10.53)	-5.50	-38.0, 18.0
	Visit 6 / Visit 12	77	26.82 (17.11)	25.00	0.0, 67.0	77	-7.23 (15.10)	-5.00	-53.0, 23.0
Placebo	Visit 2 / Visit 8	78	33.79 (17.06)	34.00	0.0, 78.0				
	Visit 4 / Visit 10	76	27.74 (16.52)	24.00	0.0, 72.0	76	-5.43 (10.56)	-6.00	-27.0, 20.0
	Visit 6 / Visit 12	77	28.52 (17.44)	26.00	0.0, 77.0	77	-5.30 (11.44)	-5.00	-34.0, 17.0

SD = standard deviation

An ANCOVA model was applied to evaluate the difference between BAY2395840 and placebo at Week 4 in period 1 and period 2. The estimated mean (SE) treatment difference based on NPSI total score between BAY2395840 and placebo was -1.19 (1.406), with a 90% CI between -3.528 and 1.157.

As a sensitivity analysis, an ANCOVA based on period 1 data only was applied. At Week 4 of period 1, the estimated mean (SE) treatment difference based on NPSI total score between BAY2395840 and placebo was -4.14 (3.018), with a 90% CI between -9.161 and 0.891.

- ***Change in Patient Global Impression of Severity (PGI-S) from baseline to the end of intervention***

The mean (SD) change from baseline in the PGI-S severity at Visit 6 / Visit 12 was -0.39 (0.88) during the treatment of BA2395840 and -0.43 (0.94) during the treatment of placebo (PPS). At Visit 4 / Visit 10, it was -0.27 (0.85) during the treatment of BA2395840 and -0.37 (0.83)during the treatment of placebo (PPS).

Table 2: PGI-S: summary statistics and change from baseline by visit (per protocol set)

		Value at Visit				Change from Baseline			
Treatment	Visit	n	Mean (SD)	Median	Min, Max	n	Mean (SD)	Median	Min, Max
BAY2395840	Visit 2 / Visit 8	78	4.17 (0.83)	4.00	2.0, 6.0				
	Visit 4 / Visit 10	73	3.88 (0.96)	4.00	1.0, 6.0	73	-0.27 (0.85)	0.00	-3.0, 1.0
	Visit 6 / Visit 12	77	3.77 (0.87)	4.00	2.0, 6.0	77	-0.39 (0.88)	0.00	-3.0, 1.0
Placebo	Visit 2 / Visit 8	78	4.14 (0.83)	4.00	2.0, 6.0				
	Visit 4 / Visit 10	76	3.76 (0.94)	4.00	1.0, 6.0	76	-0.37 (0.83)	0.00	-3.0, 2.0
	Visit 6 / Visit 12	77	3.71 (0.98)	4.00	1.0, 6.0	77	-0.43 (0.94)	0.00	-3.0, 2.0

SD = standard deviation

An ANCOVA model was applied to evaluate the difference between BAY2395840 and placebo at Week 4 in period 1 and period 2. The estimated mean (SE) treatment difference based on PGI-S severity score between BAY2395840 and placebo was 0.07 (0.121), with a 90% CI between 0.131 and 0.272.

As a sensitivity analysis, an ANCOVA based on period 1 data only was applied. At Week 4 of period 1, the estimated mean (SE) treatment difference based on PGI-S between BAY2395840 and placebo was -0.02 (0.184), with a 90% CI between -0.328 and 0.284.

- ***Number of subjects achieving a ⩾30% and a ⩾50% reduction in weekly mean 24-hour average pain intensity score (i.e. responder rates using NRS)***

Responder rates using NRS were defined as the proportion of participants with a ≥30% and a ≥50% reduction in weekly mean 24-hour average pain intensity score at Week 4 in period 1 and period 2. The number of participants achieving a ⩾30% reduction on weekly mean 24-hour average pain NRS in week 4 was 24 (32.0%) in treatment of BAY2395840 and 33 (47.1%) in treatment of placebo. The number of participants achieving a ⩾50% reduction on weekly mean 24-hour average pain NRS in week 4 was 10 (13.3%) in treatment of BAY2395840 and 13 (18.6%) in treatment of placebo. Based on a marginal responder rates analysis, the odds ratios for the treatment differences between BAY2395840 and placebo were 0.5143 (90% CI 0.2624, 1.0080, ≥30% reduction) and 0.6530 (90% CI 0.2681, 1.5904, ≥50% reduction).

Table 3: Number and proportion of participants achieving a ≥30% and a ≥50% reduction on weekly mean 24-hour average pain NRS in week 4 (per protocol set)

Category	Statistics	BAY2395840 (N = 78)	Placebo (N = 78)
Achieved >=30% reduction in NRS	Number of responders	24 (32.0%)	33 (47.1%)
Achieved >=50% reduction in NRS	Number of responders	10 (13.3%)	13 (18.6%)

N: number of subjects included in the analysis set

Safety

Secondary endpoints

- ***Number of participants with treatment emergent adverse events***

  The number and proportion of participants with TEAEs were 33 (41.8%) during the treatment BAY2395840, which was higher than 26 (32.9%) during the treatment of placebo. And, the number and proportion of participants with serious TEAEs were 2 (2.5%) during the treatment of BAY2395840 and 2 (2.5%) during the treatment of placebo.

Table 4: Treatment-emergent adverse events: overall summary of number of participants (SAF)

	BAY2395840 N=79 (100%) [a]	Placebo N=79 (100%)[b]
Number (%) of participants with treatment-emergent adverse events		
Any AE	33 (41.8%)	26 (32.9%)
Any SAE	2 (2.5%)	2 (2.5%)

a: 1 participant in BAY2395840 - placebo treatment sequence did not enter period 2.
b: 1 participant in placebo - BAY2395840 treatment sequence did not enter period 2.
AE = adverse event, N = number of participants, SAE = serious adverse event, SAF = safety analysis set

Other safety endpoints:

Overall, no safety signals of BAY2395840 were observed and BAY2395840 was generally well tolerated in this study. There was 1 death reported during the treatment placebo and 4 treatment-emergent SAEs were reported: 1 participant was hospitalized for treatment of anxiety disorder during the treatment BAY2395840; 2 participants (1 participant during the treatment placebo, another participant during the treatment BAY235840) were hospitalized for treatment of hyperglycaemia; 1 participant reported gastroenteritis with fatal outcome during the treatment placebo. None of these events were assessed as related to study intervention. The percentage of participants who permanently discontinued the study intervention due to TEAEs was low (2 participants during the treatment BAY2395840, 2.5%).

No pregnancies occurred during the study.

No relevant mean changes were observed in clinical laboratory parameters and vital signs (diastolic and systolic blood pressure, heart rate) or electrocardiogram (ECG) parameters.

Clinical Pharmacology

None

Overall conclusions

Participants randomized to 2 treatment sequences were comparable regarding baseline characteristics. For all primary and secondary efficacy endpoints, results for BAY2395840 treatment and placebo treatment were generally the same. There was no difference seen in the BAY2395840 treatment compared to the placebo treatment.

BAY2395840 was generally safe and well tolerated in this study.

Publication(s) based on the study

None at the time of report.

This is the classic website, which will be retired eventually. Please visit the modernized ClinicalTrials.gov instead.

Notice to API users: Read about imminent changes impacting usage of the ClinicalTrials.gov API.

Antisense Therapy to Block the Kallikrein-kinin Pathway in COVID-19 (ASKCOV)

The safety and scientific validity of this study is the responsibility of the study sponsor and investigators. Listing a study does not mean it has been evaluated by the U.S. Federal Government. Read our disclaimer for details.

ClinicalTrials.gov Identifier: NCT04549922

Recruitment Status : Completed
First Posted : **September 16, 2020**
Last Update Posted : September 22, 2022

View this study on the modernized ClinicalTrials.gov

Sponsor:
Hospital do Coracao

Collaborators:
Ionis Pharmaceuticals, Inc.
Hospital Moinhos de Vento

Information provided by (Responsible Party):
Hospital do Coracao

Study Details | Tabular View | No Results Posted | Disclaimer | How to Read a Study Record

Study Description

Go to

Brief Summary:

Up to 1/3 of all patients infected with COVID-19 can develop complications that require hospitalization. Severe pneumonia associated with acute respiratory distress syndrome (ARDS) is the most threatening and feared complication of COVID-19 infection, with mortality rates close to 50% in some groups.

Autopsies between these severe cases reveal severe capillary involvement, with signs of intense inflammatory changes, microvascular thrombosis, endothelial injury and abnormal tissue repair. The available evidence suggests that abnormal activation or imbalance in the counter-regulation of the kallikrein-kinin system may play a central role in a positive feedback cycle, leading to consequent diffuse microangiopathy. Blockade of the kallikrein-kinin system can therefore prevent deterioration of lung function by reducing inflammation, edema and microthrombosis.

The objective of this phase IIb study is to assess the preliminary effects on the oxygenation parameters of an antisense oligonucleotide that inhibits pre-kallikrein synthesis in patients with moderate to severe COVID-19.

Condition or disease	Intervention/treatment	Phase
**Covid19**	Drug: ISIS 721744<br>Drug: Normal Saline	Phase 2

Detailed Description:

The objective of this phase IIb study is to assess the preliminary effects on the oxygenation parameters of an antisense oligonucleotide that inhibits pre-kallikrein synthesis in patients with moderate to severe COVID-19.

This is a blind randomized pilot clinical study which aims to include 110 patients (55 per arm).

Study Design

Go to

Study Type : Interventional (Clinical Trial)
Actual Enrollment : 111 participants
Allocation: Randomized
Intervention Model: Parallel Assignment
Masking: Quadruple (Participant, Care Provider, Investigator, Outcomes Assessor)
Masking Description: The placebo used in the present study will consist of 0.9% NaCl Solution, which has the same physical characteristics as the ISIS 721744 drug. The placebo solution will be prepared by the nurse or pharmacist of the research team at the time of application. The nurse will be trained on the procedures for adequate blinding of the intervention. The pharmacist at the site level will not be blind, but the rest of the ream (nurse, physicians, patients, etc) will be blinded.
Primary Purpose: Treatment
Official Title: Antisense Therapy to Block the Kallikrein-kinin Pathway in **COVID-19**: A Phase II Randomized Controlled Trial
Actual Study Start Date : October 19, 2020
Actual Primary Completion Date : December 9, 2020
Actual Study Completion Date : December 9, 2021

Resource links provided by the National Library of Medicine

MedlinePlus related topics: COVID-19 (Coronavirus Disease 2019)

U.S. FDA Resources

Arms and Interventions

Go to

Arm	Intervention/treatment
Placebo Comparator: Placebo<br>1.2 mL Normal Saline, single dose subcutaneous, after randomization	Drug: Normal Saline<br>1.2 mL subcutaneous
Active Comparator: ISIS 721744<br>1.2 mL ISIS 721744, single dose subcutaneous, after randomization	Drug: ISIS 721744<br>1.2 mL of ISIS 721744 subcutaneous once after randomization

Outcome Measures

Go to

Primary Outcome Measures :

1. Days alive without respiratory support (any supplemental oxygen) after 15 days (DAFOR15) [Time Frame: 15 days]
   Number of days the patient is alive and not receiving any supplementary respiratory support (oxygen, non-invasive ventilation, high flow nasal catheter or mechanical ventilation) during 15 days

Secondary Outcome Measures :

1. SOFA - Sequential Organ Failure Assessment Score up to 15 days after randomization [Time Frame: 15 days]
   Sequential Organic Failure Assessment [SOFA]. This will be a primary secondary outcome. Analysis will check for trends in SOFA up to 15 days in a single model.
2. Need for mechanical ventilation [Time Frame: 30 days (or until hospital discharge)]
   Intubation and initiation of mechanical ventilation for any given reason
3. Duration of mechanical ventilation [Time Frame: 30 days (or until hospital discharge)]
   Number of days the patient remains in mechanical ventilation

Other Outcome Measures:

1. Oxygenation index [Time Frame: 14 days]
   Daily oxygenation levels assessed using the ROX index [(Oxygen Saturation/Inspired Fraction)/Respiratory Rate] from randomization to discharge or day 14, whichever comes first.
2. C-reactive protein levels during first 15 days after randomization [Time Frame: 15 days]
   C-reactive protein levels over time up to 15 days or hospital discharge.
3. Lymphocyte/neutrophil ratio during first 15 days after randomization [Time Frame: 15 days]
   Lymphocyte/neutrophil ratio over time up to 15 or until discharge discharge day.
4. D-dimer serum level during first 15 days after randomization [Time Frame: 15 days]
   D-dimer serum levels over time or until hospital discharge.
5. Fibrinogen serum levels during first 15 days after randomization [Time Frame: 15 days]
   Fibrinogen serum levels over time up to 15 days or until hospital discharge
6. Prothrombin Time levels during first 15 days after randomization [Time Frame: 15 days]
   Prothrombin Time over time up to 15 days or until hospital discharge.
7. Activated Partial Thromboplastin Time during first 15 days after randomization [Time Frame: 15 days]
   Activated Partial Thromboplastin Time over time up to 15 days or until hospital discharge.
8. Mortality [Time Frame: 1 year after randomization]
   One-year all cause mortality
9. Euroquol questionnaire for quality of life with 5 dimensions (EQ-5D) [Time Frame: 1 year after randomization]
   Quality of Life measured by EQ-5D from 11111-33333, lower values being better

Eligibility Criteria

Go to

Information from the National Library of Medicine

Choosing to participate in a study is an important personal decision. Talk with your doctor and family members or friends about deciding to join a study. To learn more about this study, you or your doctor may contact the study research staff using the contacts provided below. For general information, Learn About Clinical Studies.

Ages Eligible for Study: 18 Years to 80 Years (Adult, Older Adult)
Sexes Eligible for Study: All
Accepts Healthy Volunteers: No

Criteria

Inclusion Criteria:

1. Patients with COVID-19 who need supplemental oxygen
2. Women must not be pregnant or breastfeeding, and must be surgically sterile or in the postmenopausal stage (without risk of becoming pregnant).
3. Men must be surgically sterile or abstinent or, if they are having sex with risk of pregnancy, the subject must use an effective method of contraception from the moment they sign the informed consent form until at least 24 weeks after the dose of the study drug (ISIS 721744 or placebo).

Exclusion Criteria:

1. Patients on invasive mechanical ventilation or who may need mechanical ventilation for the next 24 hours. The use of non-invasive ventilation and/or a high-flow nasal catheter is permitted.
2. Patients with > 10 days since symptom onset or more than 48h of oxygen use
3. Pregnancy, breast-feeding or risk of becoming pregnant
4. Hemodynamically unstable (use of vasoconstrictors, such as norepinephrine, at any dose)
5. Previous diagnosis of heart failure at functional class III or IV
6. Previous uncontrolled hypertension (more than 3 drug classes use at home)
7. Severe lung disease (use of home oxygen)
8. Age < 18 and > 80 years
9. Physician and family not committed to full life support and/or with severe existing illness with a life expectancy of less than 12 months
10. Refusal to accept informed consent and/or unwillingness to comply with all requirements of the study procedure and security monitoring

Contacts and Locations

Go to

Information from the National Library of Medicine

To learn more about this study, you or your doctor may contact the study research staff using the contact information provided by the sponsor.

Please refer to this study by its ClinicalTrials.gov identifier (NCT number): ***NCT04549922***

Locations

Brazil

Hospital Naval Marcilio Dias
Rio De Janeiro, Brazil

BP-A Beneficiência Portuguesa de São Paulo
São Paulo, Brazil

Hospital São Paulo - UNIFESP
São Paulo, Brazil

Sponsors and Collaborators

Hospital do Coracao

Ionis Pharmaceuticals, Inc.

Hospital Moinhos de Vento

Investigators

Principal Investigator: Fernando G Zampieri, MD Research Coordinator

Study Chair: Alexandre B Cavalcanti Institute Director

More Information

Go to

Responsible Party: Hospital do Coracao
ClinicalTrials.gov Identifier: NCT04549922 History of Changes
Other Study ID Numbers: ASKCOV_Trial
First Posted: **September 16, 2020** Key Record Dates
Last Update Posted: September 22, 2022
Last Verified: September 2022

Individual Participant Data (IPD) Sharing Statement:
Plan to Share IPD: Yes
Plan Description: Data will be available under reasonable request after approval by the steering committee.
Supporting Materials: Study Protocol
Statistical Analysis Plan (SAP)
Time Frame: Protocol will be available in December, together with analysis plan
Access Criteria: Protocol and analysis plan will be uploaded to Clinicaltrials.gov

Studies a U.S. FDA-regulated Drug Product: No
Studies a U.S. FDA-regulated Device Product: No

Keywords provided by Hospital do Coracao:
antisense
kallikrein-kinin

Additional relevant MeSH terms:

**COVID-19**	Coronavirus Infections
Pneumonia, Viral	Coronaviridae Infections
Pneumonia	Nidovirales Infections
Respiratory Tract Infections	RNA Virus Infections
Infections	Lung Diseases
Virus Diseases	Respiratory Tract Diseases
